# Supplementary material for: Rbm24a dictates mRNA recruitment for germ granule assembly in zebrafish
Source: EMBO J. 2025 Apr 25;44(11):3121–49. doi: 10.1038/s44318-025-00442-z (PMC12130248; doi:10.1038/s44318-025-00442-z)
Supplement: Supplementary file 1 — Appendix [file 44318_2025_442_MOESM1_ESM.pdf]

Appendix for Rbm24a dictates mRNA recruitment for germ granule assembly in zebrafish

Yizhuang Zhang[1,2], Jiasheng Wang[1,2], Hailing Fang[1,2], Shuqi Hu[1], Boya Yang[1,2], Jiayi Zhou[1,2], Raphaëlle Grifone[3], Panfeng Li[1], Tong Lu[1,2], Zhengyang Wang[4], Chong Zhang[5], Yubin Huang[6], Dalei Wu[6], Qianqian Gong[1,2], De-Li Shi[3,7]*, Ang Li[1]*, Ming Shao[1,2,8]*

[1] Shandong Provincial Key Laboratory of Animal Cell and Developmental Biology, School of Life Sciences and Qilu Hospital (Qingdao), Cheeloo College of Medicine, Shandong University, Qingdao 266237, China

[2] Key Laboratory for Experimental Teratology of the Ministry of Education, Shandong University, Qingdao 266237, China

[3] Sorbonne Université, Institut de Biologie Paris-Seine (IBPS), UMR CNRS 8263, INSERM U1345, Development, Adaptation and Ageing, Paris-France

[4] Shandong University Taishan College, Qingdao 266237, China

[5] Zhanjiang Institute of Clinical Medicine, Central People's Hospital of Zhanjiang, Guangdong Medical University Zhanjiang Central Hospital, Zhanjiang 524045, China

[6] State Key Laboratory of Microbial Technology, Institute of Microbial Technology, Qingdao 266237, China

[7] Fang Zongxi Center, Key Laboratory of Marine Genetics and Breeding, College of Marine Life Sciences, Ocean University of China, Qingdao, China.

[8] Shandong University-Yuanchen Joint Biomedical Technology Laboratory, Qingdao, 266237, China

*Correspondence: de-li.shi@upmc.fr, angli41@sdu.edu.cn, shaoming@sdu.edu.cn

Table of Contents:

Appendix Fig. S1: 1
Appendix Fig. S2: 2
Appendix Fig. S3: 3
Appendix Fig. S4: 4
Appendix Fig. S5: 5
Appendix Fig. S6: 6
Appendix Fig. S7: 7
Appendix Fig. S8: 8
Appendix Fig. S9: 9
Appendix Fig. S10: 10
Appendix Fig. S11: 11
Appendix Fig. S12: 12
Appendix Fig. S13: 13
Appendix Table S1: 14-19
Appendix Table S2: 20-21

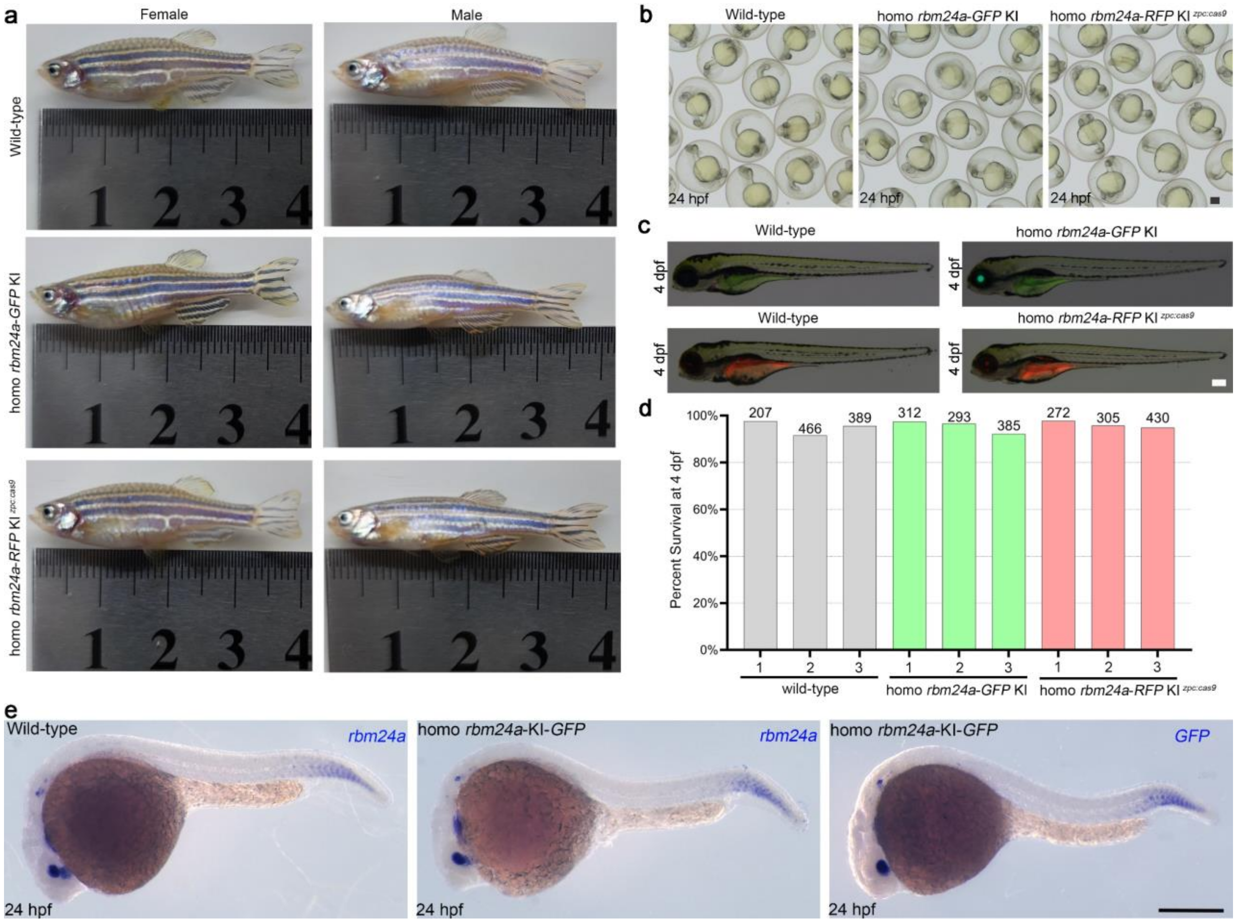

Appendix Figure S1. Rbm24a tagging does not affect its functionality.

(**a**) Homozygous *rbm24a-GFP* KI and *rbm24a-RFP* KI$^{zpc:cas9}$ fish can survive to adulthood. (**b**, **c**) Embryos spawned by homozygous *rbm24a-GFP* KI and *rbm24a-RFP* KI$^{zpc:cas9}$ fish show no developmental defect at 24 hpf (**b**) and 4 dpf (**c**). Scale bars, 200 µm. (**d**) Comparison of survival rate at 4 dpf between wild-type and homozygous knock-in fish. (**e**) In situ hybridization results showing identical expression patterns of wild-type *rbm24a* and *GFP*-tagged *rbm24a.* Scale bar, 200 µm. Source data are available online for this figure.

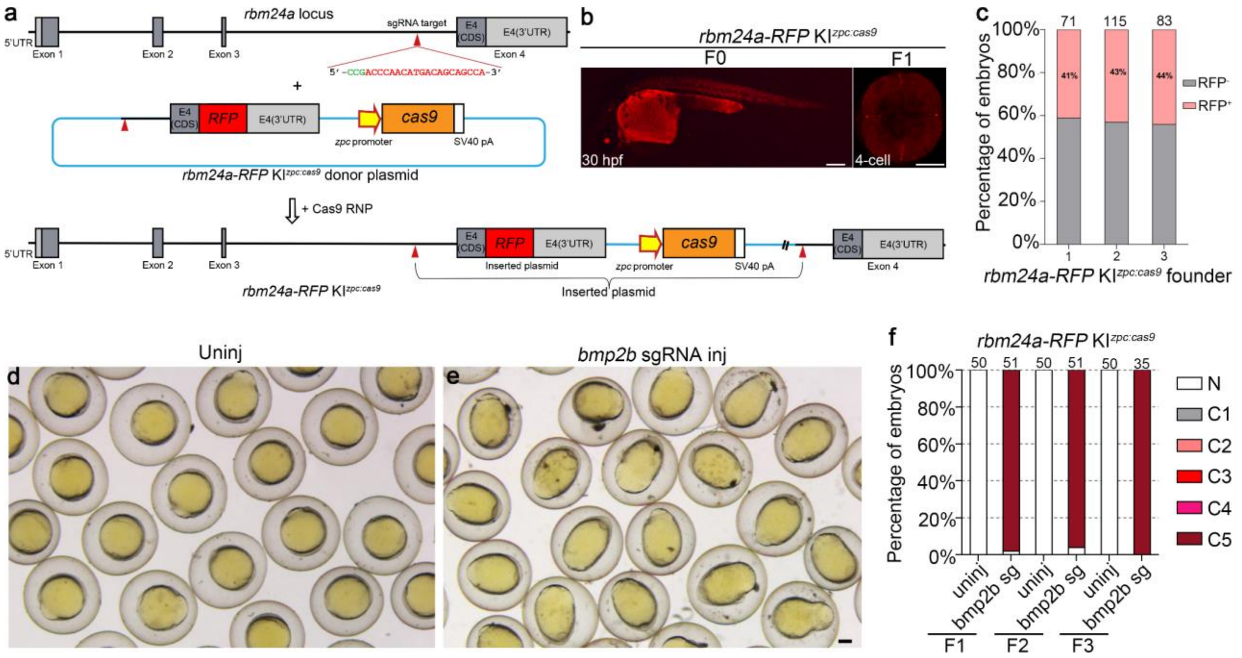

Appendix Figure S2: Construction of *cas9* knock-in line for maternal gene knockout.

(**a**) Knockin strategy to construct *rbm24a-RFP* KI$^{zpc:cas9}$. (**b**) Rbm24a-RFP localization in the KI embryos. Scale bars, 200 µm. (**c**) Germline transmission and percentage of knockin offspring from three founder *rbm24a-RFP* KI$^{zpc:cas9}$ fish with early integration. (**d**, **e**) Dorsalized phenotypes caused by injecting *bmp2b* sgRNA into KI embryos. Scale bar, 200 µm. (**f**) High percentage of dorsalized phenotypes caused by *bmp2b* sgRNA injection in embryos from different generations of KI fish. Source data are available online for this figure.

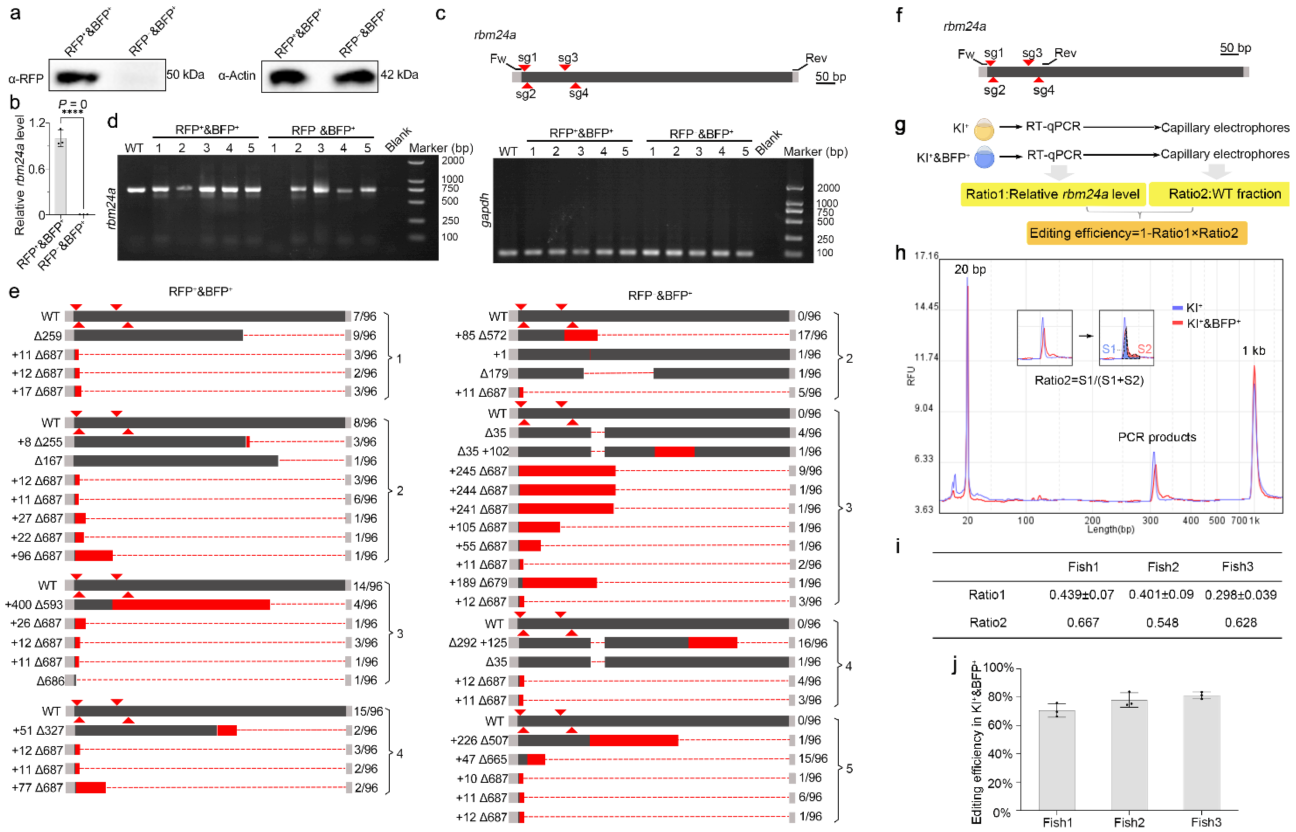

Appendix Figure S3. Genotyping M*rbm24a* embryos.

(**a**) Western blot analysis showing the absence of Rbm24a-RFP expression in RFP$^-$&BFP$^+$ embryos. (**b**) The transcript level of *rbm24a* is significantly reduced in RFP$^-$&BFP$^+$ embryos, as analyzed by qRT-PCR. (**c**) Primer design to amplify the entire ORF of *rbm24a* transcripts. (**d**) Agarose gel analysis of PCR products from RFP$^+$&BFP$^+$ and RFP$^-$&BFP$^+$ embryos. (**e**) Mutations detected by Sanger sequencing after cloning the PCR products into the pT2AL200R150G plasmid. (**f**) qPCR primer design amplifying the cDNA region containing the four sgRNA targeting sites. (**g**) Diagram illustrating the procedure to estimate the editing efficiency in *rbm24a-RFP* KI$^{zpc:cas9}$;Tg(*U6*:4xsgRNAs^{rbm24a};*ef1α*:*BFP*) double transgenic embryos (KI$^+$&BFP$^+$) at the 1-cell stage. (**h**) Electropherogram of high-resolution capillary electrophoresis and the principle to define the wild-type fraction. (**i**) Ratio1 and Ratio2 measured from KI$^+$&BFP$^+$ offspring from three independent double transgenic fish. (**j**) Histogram showing the final calculated editing rates (n = 3 independent biological samples). Data are presented as mean ± SD. Source data are available online for this figure.

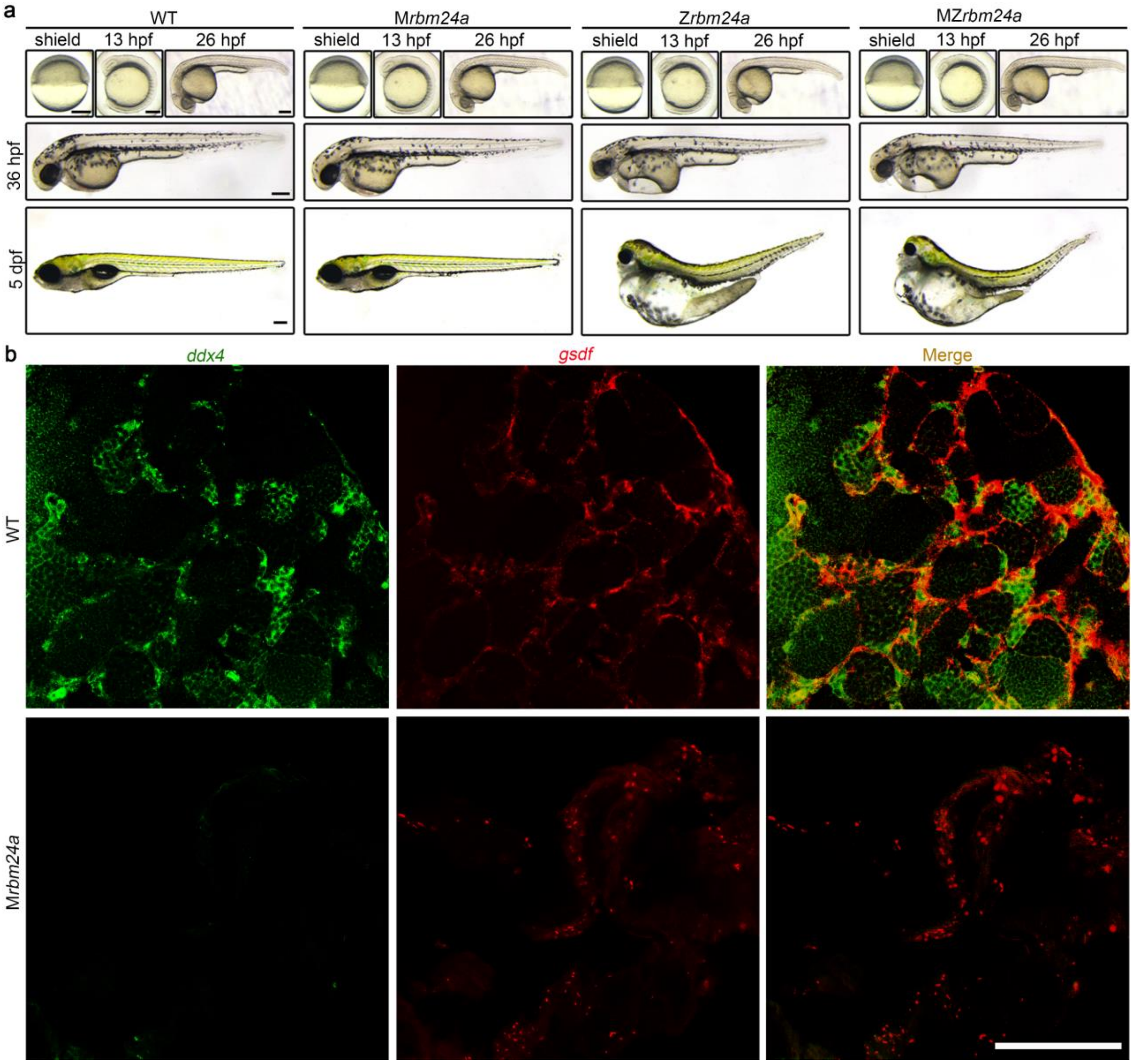

Appendix Figure S4. Phenotyping different *rbm24a* mutants.

(**a**) Characteristic phenotypes of *rbm24a* maternal mutant (M*rbm24a*), zygotic mutant (Z*rbm24a*) and MZ mutant (MZ*rbm24a*). Scale bars, 200 µm. (**b**) M*rbm24a* exhibits a specific germ cell loss in adult testis. Double color FISH was performed using *ddx4* probe to label developing germ cells and a *gsdf* probe to label somatic cells. Scale bar, 100 µm. Source data are available online for this figure.

a

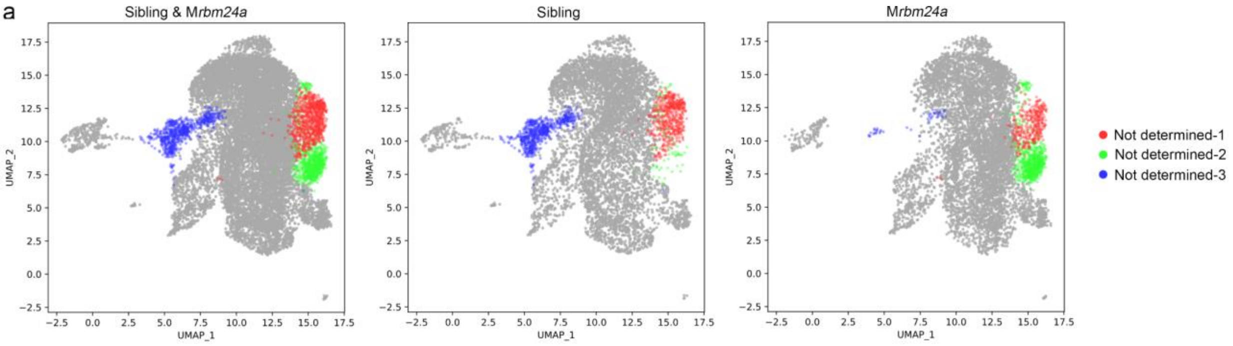

b

Not determined-1		
**Top DEGs**	**padj**	**log2FC**
si:ch211-246m6.4	0	3.053215
mt-rnr2	0	2.422381
rps11	0	1.433679
rpl17	0	1.019828
rpl18a	0	1.30973
prmt1	0	1.305479
ulk4	0	5.147068
rplp1	0	1.024048
zgc:153409	2.13E-302	1.618263
rpl23a	9.40E-302	1.456913

Not determined-2		
**Top DEGs**	**padj**	**log2FC**
marcksl1b	3.17E-123	0.431315
h2afvb	1.87E-117	0.400257
h3f3a	4.89E-114	0.464559
cbx3a	3.13E-112	0.507179
LOC110439626	2.52E-108	-3.662787
dynll1	4.24E-108	0.402867
hnrnpaba	1.64E-105	0.362786
hnrnpa0b_1	1.01E-97	0.767894
h2afx	2.66E-96	1.151767
hmgb2b	1.18E-94	0.520731

Not determined-3		
**Top DEGs**	**padj**	**log2FC**
LOC110439627	0	6.927536
LOC110439626	0	8.114172
mt-rnr1	0	3.612719
zgc:158463	6.43E-276	2.328873
mt-rnr2	1.42E-247	1.965721
lye	1.52E-187	3.86216
krt4_1	7.06E-135	4.628946
pfn1	3.47E-131	1.042445
cyt1	1.94E-125	4.714391
krt92	2.36E-120	4.741928

c

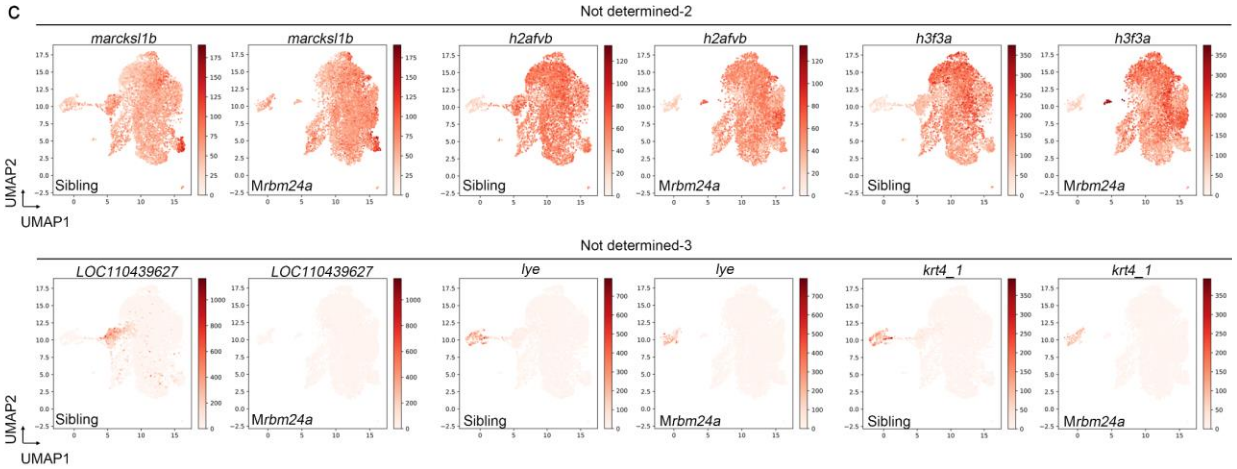

Appendix Figure S5. Characterization of three undetermined cell clusters in M*rbm24a* embryos at 6 hpf.
(**a**) Clustering map of scRNA-seq showing a decrease of “not determined-3” and an increase of “not determined-2” in M*rbm24a* embryos. (**b**) Top DEGs of “not determined-1, 2 and 3”. (**c**) UMAP map showing the expression of selected top DEGs in “not determined-2 and 3”. Source data are available online for this figure.

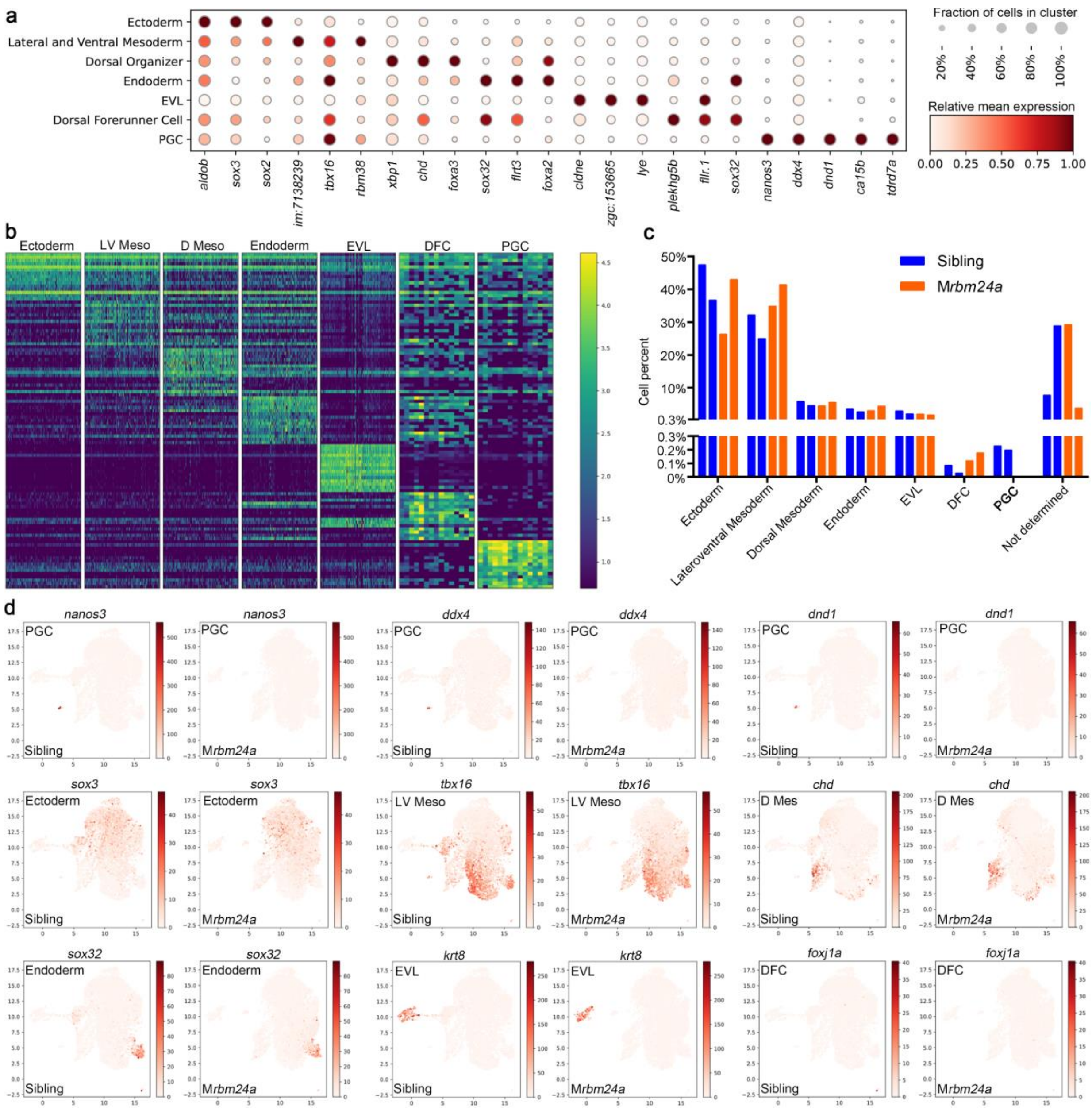

Appendix Figure S6. scRNA-seq analysis of gene expression in sibling embryos and M*rbm24a* mutants at 6 hpf.

(**a**) Bubble plot showing the expression of marker genes in different clusters of cells. (**b**) Heat map showing the top 15 genes expressed in each cluster. (**c**) Percentage of cells in each cluster from sibling and M*rbm24a* embryos. (**d**) UMAP map showing marker gene expression. Source data are available online for this figure.

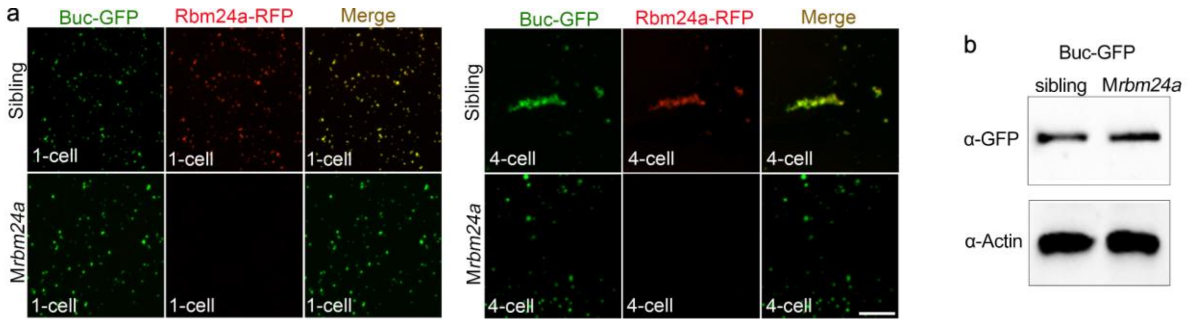

Appendix Figure S7. Expression of transgenic Buc-GFP in M*rbm24a* embryos.

(**a**) Distribution of Buc-GFP in sibling and M*rbm24a* embryos at 1-cell and 4-cell stages. Scale bar, 25 µm. (**b**) Western blot showing comparable expression of transgenic Buc-GFP in sibling and M*rbm24a* embryos. Source data are available online for this figure.

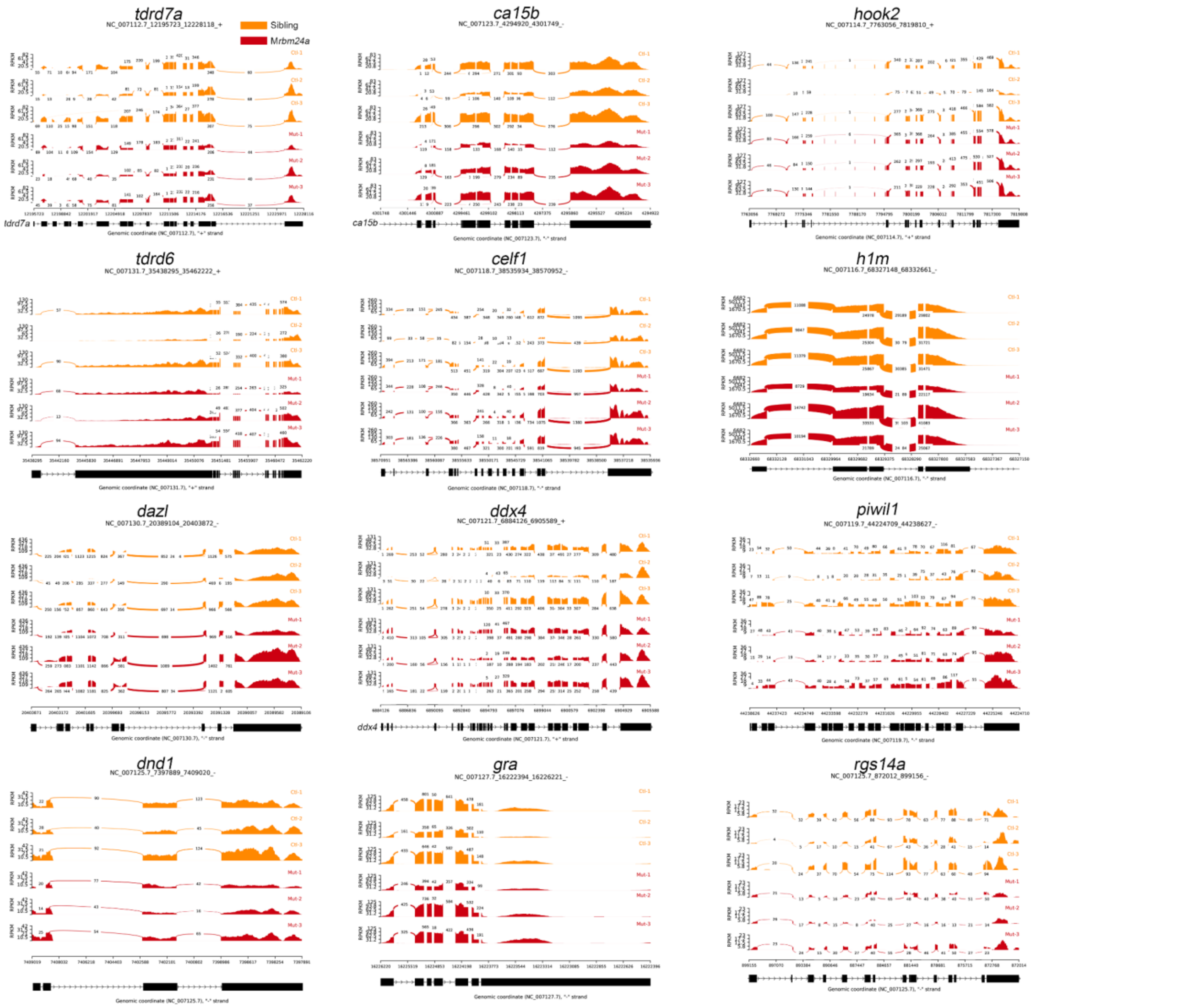

Appendix Figure S8. Mapping RNA-seq reads of germ plasm mRNAs at 4-cell stage.

No discernible splicing difference between siblings and M*rbm24a* embryos is observed for all germ plasm mRNAs. *nanos3* is absent from this analysis because it has only a single exon. Source data are available online for this figure.

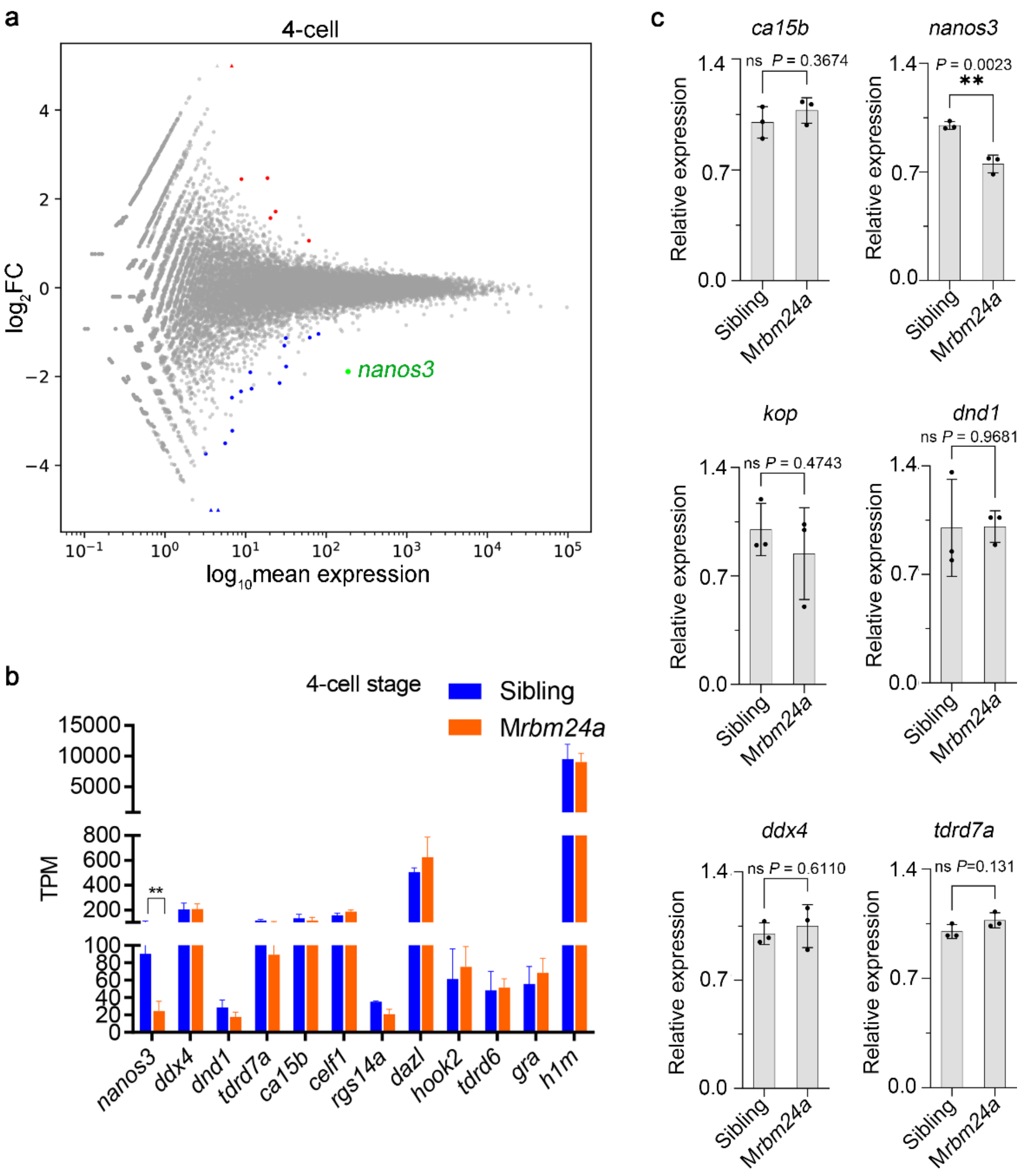

Appendix Figure S9. Expression of germ plasm mRNAs analyzed by RNA-seq and qRT-PCR.
(**a**) MA plot of RNA-seq showing an essentially similar transcriptome of sibling and M*rbm24a* embryos at the 4-cell stage. Among the germ plasm mRNAs, only *nanos3* is down-regulated. (**b**) Histogram showing the expression of twelve germ plasm transcripts in siblings and M*rbm24a* embryos (n = 3 independent biological samples). (**c**) qRT-PCR results showing a significant reduction in *nanos3* level in M*rbm24a*, but other germ plasm mRNAs are expressed normally (n = 3 independent biological samples). Data are presented as mean ± SD. ** $P < 0.01$, ns P > 0.05, unpaired Student's t test. Source data are available online for this figure.

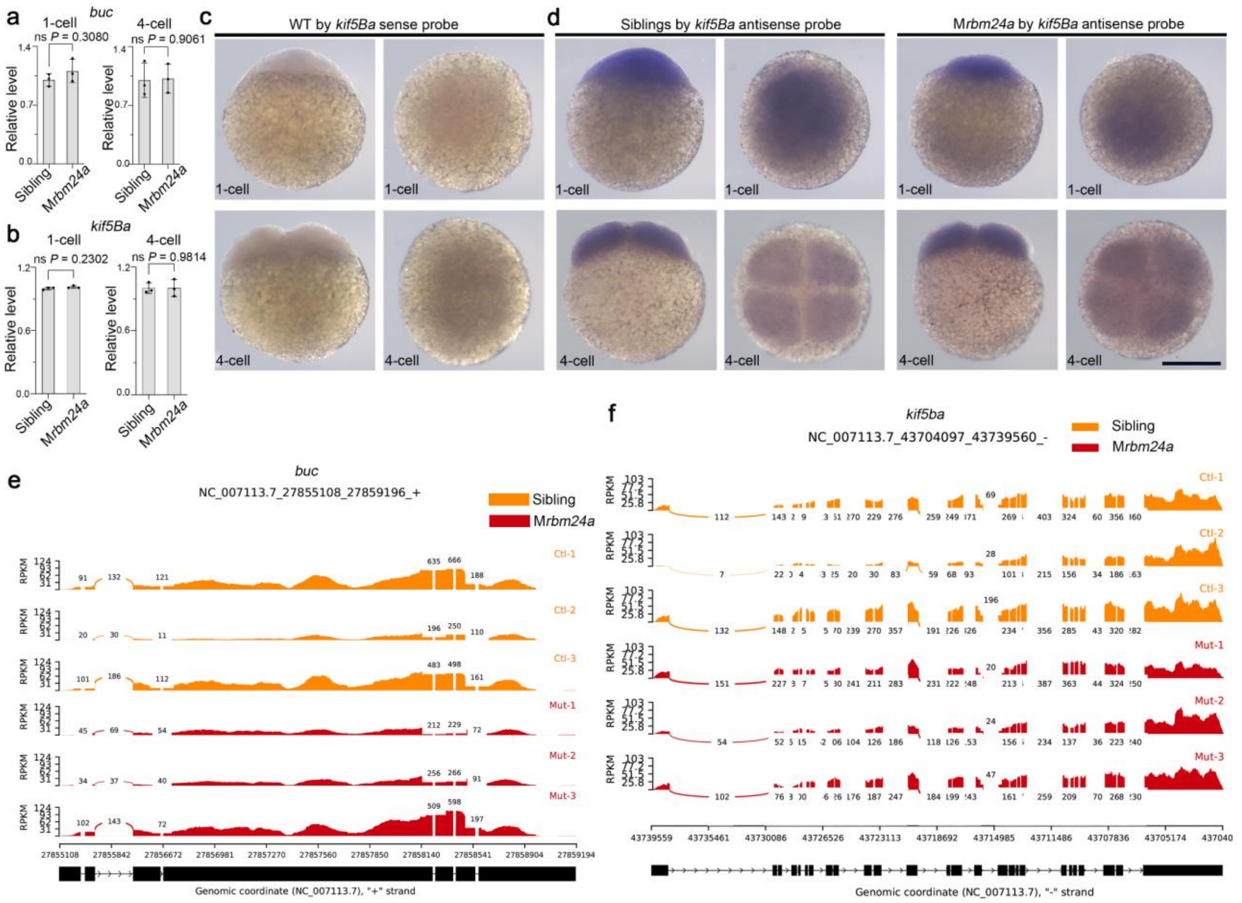

Appendix Figure S10. Expression and splicing of *buc* and *kif5Ba* are unaffected upon *rbm24a* mutation.

(**a**) qRT-PCR analysis shows comparable levels of *buc* transcripts between siblings and M*rbm24a* embryos at both 1-cell and 4-cell stages (n = 3 independent biological samples). Data are presented as mean ± SD. ns P > 0.05, unpaired Student's *t*-test. (**b**) Similar expression levels of *kif5Ba* between siblings and M*rbm24a* embryos (n = 3 independent biological samples). Data are presented as mean ± SD. ns P > 0.05, unpaired Student's *t*-test. (**c**) No signal is detected by in situ hybridization using a sense probe for *kif5Ba*. (**d**) *kif5Ba* exhibits a wide-spread expression with no difference between siblings and M*rbm24a* embryos. Scale bar, 200 µm for **c** and **d**. (**e**) Mapping of RNA-seq reads for buc and *kif5Ba* shows no change in splicing. Source data are available online for this figure.

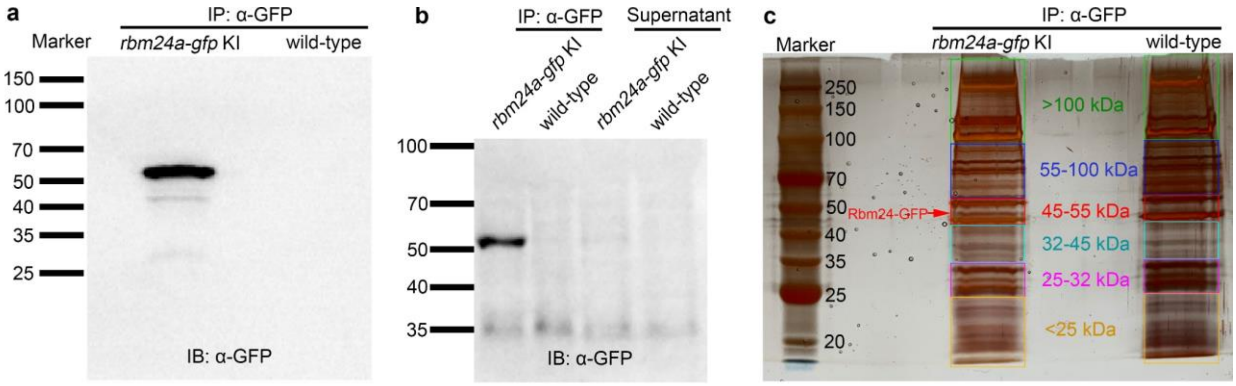

Appendix Figure S11. Quality control and fragmentation of samples for IP/MS analysis.

(**a**) Western blot showing the specific precipitation of maternal Rbm24a-GFP. (**b**) Highly efficient IP procedure as assayed by western blot analysis of the IP sample and the supernatant. (**c**) Protein samples were cut from the gel after silver staining and subjected individually to MS analysis. Source data are available online for this figure.

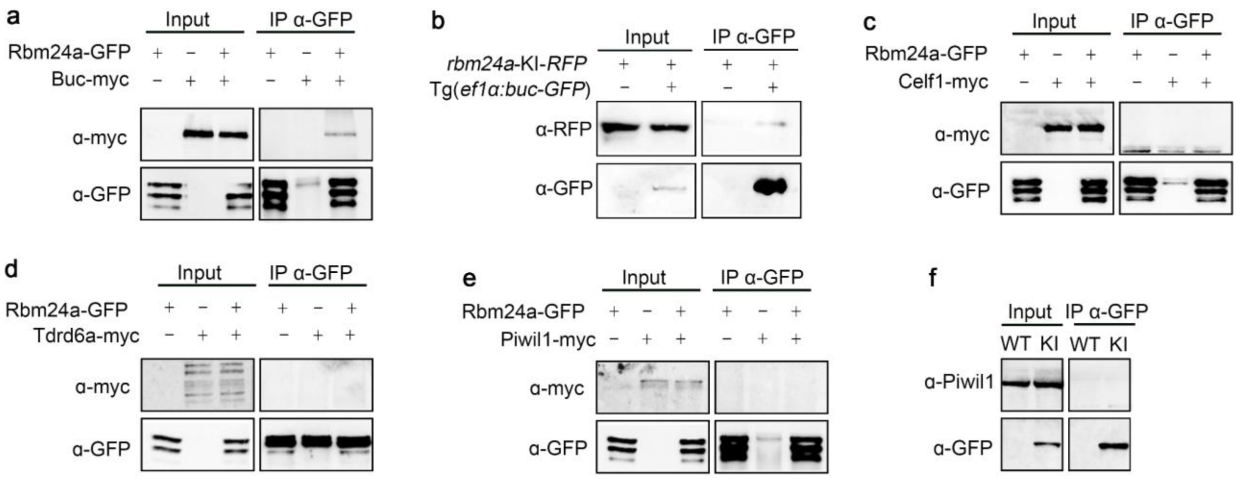

Appendix Figure S12. Rbm24a binds to Buc but not to other germ plasm proteins.

(**a**) Co-IP analysis of the interactions between overexpressed Rbm24a-GFP and myc-tagged Buc proteins. (**b**) Co-IP analysis showing the interaction between endogenous Rbm24a-RFP and transgenic Buc-GFP. (**c-e**) Co-IP analysis showing the absence of interaction between overexpressed Rbm24a-GFP and different myc-tagged germ plasm proteins. (**f**) No interaction is detected between endogenous Piwil1 and Rbm24a-GFP. Source data are available online for this figure.

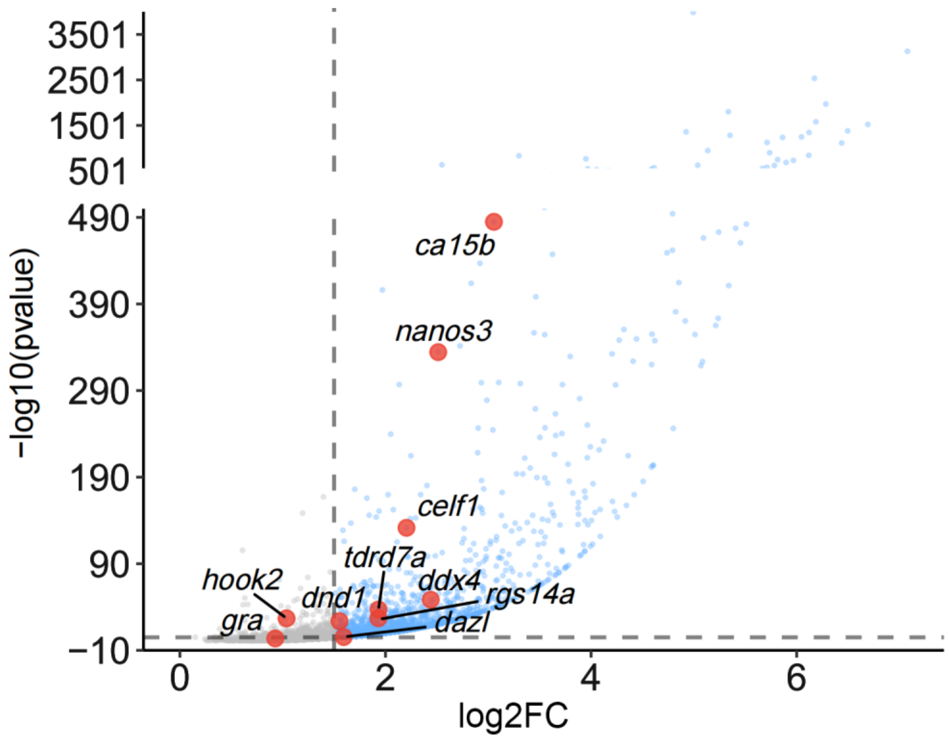

Appendix Figure S13. RIP-seq analysis of Rbm24a-binding transcripts using IgG as a negative control. The volcano plot illustrates mRNA enrichment by Rbm24a-GFP in knock-in embryos at the 4-cell stage, using IgG as a control. Genes with a log2FC > 1.5 and a *P*-value < 0.00001 are considered as statistically significant enrichment. Germ plasm transcripts are highlighted in red dots. Source data are available online for this figure.

Appendix Table S1. Primers and DNA sequences used in this study.

**primers and DNA sequences**	**Usage**	**Name**	**Sequence 5'-3'**	**note**
	**For generating sgRNA for knock in**	*rbm24a*-sgRNA65	TAATACGACTCACTATAGGGCTGCTGTCATGTTGGGTGTTTTAGAGCTAGAA	
	**Primers for sgRNA synthesis**	*rbm24a* Target_1	TAATACGACTCACTATAGGGGATGCATACCACGCAAAGTTTTAGAGCTAGAA	
		rbm24a Target_2	TAATACGACTCACTATAGGAAGTGGTGTCCTTTTGCGGTTTTAGAGCTAGAA	
		rbm24a Target_3	TAATACGACTCACTATAGGTGACGATGGCGGATCGCTGTTTTAGAGCTAGAA	
		rbm24a Target_4	TAATACGACTCACTATAGGCCGTCGATGATGGGGTTTGTTTTAGAGCTAGAA	
		bmp2b-Target	TAATACGACTCACTATAGGGAGGCTGAGAGCAACCGGGTTTTAGAGCTAGAA	
		buc-sgRNA59	TAATACGACTCACTATAGGGACGAAGAAAATCAGCACGTTTTAGAGCTAGAA	
		buc-sgRNA61a	TAATACGACTCACTATAGGAGGTTGCCCAACTCCCATGTTTTAGAGCTAGAA	
		buc-sgRNA61b	TAATACGACTCACTATAGGGATGCAACTGAGGGGTAGGTTTTAGAGCTAGAA	
		buc-sgRNA67	TAATACGACTCACTATAGGGACAGAGGAACTGTCAAGGTTTTAGAGCTAGAA	
	**Primers for genotyping CRISPants or KI line**	*rbm24a*-sg65-F	CTGCAAACCAGACTTTTGCAC	
		rbm24a-R	CGTGTGGAGTTGACAAATGAG	
		M13-F	GTAAAACGACGGCCAGT	
		rbm24a-sg65-R	CAAATGCTATGGAAGTCAACGG	
		rbm24a 1&2-F	GAGGAGCTCGACCCACCTGAT	
		rbm24a 1&2 -R	GTTTACACAACTCCACAAGAGG	
		rbm24a 3&4-F	GCTTGTACATGTAGGTCAACA	
		rbm24a 3&4-R	CGTTATTTGAGATGCCTGACTG	
		bmp2b-target-F	AGTTCTCGGAGATGCCAGTA	
		bmp2b-target-R	GCTTTTCTCGGTTCGAATGC	
		buc-sg59-F	GTTAGTCACTTCTGCTGTCG	

		buc-sg59-R	CCCAGGTTACCCAAACCATG	
		buc-sg61-F	CCTCATCTACCCTTGCTTGA	
		buc-sg61-R	CCCTTGTCACTGCCTAGTTC	
		buc-sg65-F	TGGGATGCATCGGGAAACTG	
		buc-sg65-R	CTGCAGCAGCATCCGCAGAT	
	**Primers to amplify fragments of probes**	*nanos3*-F	GCCTTGGAAGGACTACATGG	
		nanos3-R	CGCACACATGCATGCAAACTCGC	
		kop-F	GTGCACATCTCTGACAAGAC	
		kop-R	CATCACACCATTCTGTTCGGTC	
		ca15b-F	ACGGCACTCAACAGTCTCCAAT	
		ca15b-R	TGAAGACGGTCCAGATCACA	
		vasa-F	CTGGCCTCACACCTGTTATA	
		vasa-R	CCTACAAACAGTCACAATC	
		tdrd7a-F	GATGAGTGACGTGGAGTTGGTTA	
		tdrd7a-R	TGGCTGTATTTATTGAGCAGCTG	
		dnd1-F	ACTCCCCAGGAAGACAGCTG	
		dnd1-R	CAACAGGTGTTTATTAACAGTGC	
		kif5Ba.F1	ccaacagacatgtagctgtg	
		kif5Ba.F2	ccaggagtcacagtatcttc	
		kif5Ba.R	TAATACGACTCACTATAGGGatccagtgccaacacttctg	
		gsdf.F1	ATCGTGCCGTTCGTCTACATG	
		gsdf.F2	TCTGGTTCTGTCTACGGTGAG	
		gsdf.R	TAATACGACTCACTATAGGGCTGCCAGAGCCAAACCCGCAG	
		Sense probe-kif5Ba.F1	TAATACGACTCACTATAGGGccaacagacatgtagctgtg	
		Sense probe-kif5Ba.R	atccagtgccaacacttctg	
	**qPCR primers**	QN-*nanos3*-F	ATGGCTTTTTCTCTTCTCCA	Nested PCR 1st
		QN-*nanos3*-R	TGTTCTGCTCCGGTGAGTCG	
		Q-*dazl*-F	TTCTCGGTCAGTGTCATCTC	Tm=62 ℃
		Q-*dazl*-R	TTTGCTCTCCACACCACTGT	
		Q-*vasa*-F	TGAGGAAGCAGGACTTTGTGAC	
		Q-*vasa*-R	GTAGGATAGGCAGCAGGAAG	
		Q-*dnd*-F	AGGAGCTTCAGCAGATTCTG	
		Q-*dnd*-R	GTTCGGGATCTGACTGATGA	
		Q-*nanos3*-F	GGAGACTAGAAACCAGGACT	
		Q-*nanos3*-R	TGTTCTGCTCCGGTGAGTCG	
		Q-luciferase-F	TCGGTTGGCAGAAGCTATGA	

		Q-luciferase -R	ACGAACGTGTACATCGACTG	
		Q-kif5Ba-F	gactttgcacaacctcaggaaa	
		Q-kif5Ba-R	gcagacgcttctccagtttagg	
		Q-buc.F	caagttactggacctcaggatc	
		Q-buc.R	ggcagtaggtaaattcggtctc	
		Q-TMrbm.F1	GCACTTGAGCGTTTCGTGAG	
		Q-TMrbm.R1	CCGAGATAAGCCAGGTTAAC	
	**primers for construction of plasmids**	*rbm24a* left arm-F	CTCACTATAGGGCGAATTGGGTACCGGGCCCTGCAAACCAGACTTTTGC	*rbm24a-GFP* KI
		rbm24a left arm-R1704	GCCCTTGCTCACCATTTGCATGCGTTCGGC	
		EGFP-F	GCCGAACGCATGCAAATGGTGAGCAAGGGC	
		EGFP-R720	CCGCCTCTTTGGTTGTTACTTGTACAGCTCGTCCATG	
		*rbm24a-R*ight arm-F	GAGCTGTACAAGTAACAACCAAAGAGGCGGAC	
		*rbm24a-*Right arm-R2274	TTATCGATACCGTCGACCTCGAGGGGGGGCCGCAGCGTGTGCAATGA	
		zpc-zcas9-F	GATCCACTAGTTCTAGAGCGGCCGCGAAAATCCCCATGACATGC	*rbm24a-RFP* KI$^{zpc:cas9}$
		zpc-zcas9-R5003	CTCCACCGCGGTGGCAGATCTGATCTAGAGGATCATAATCA	
		rbm24a sg1-F	TTCGGGGATGCATACCACGCAAA	pGGDest EB-rbm24a-4sgRNA
		rbm24a sg1-R	AAACTTTGCGTGGTATGCATCCC	
		rbm24a sg2-F	TTCGGAAGTGGTGTCCTTTTGCG	
		rbm24a sg2-R	AAACCGCAAAAGGACACCACTTC	
		rbm24a sg3-F	TTCGGTGACGATGGCGGATCGCT	
		rbm24a sg3-R	AAACAGCGATCCGCCATCGTCAC	
		rbm24a sg4-F	TTCGGCCGTCGATGATGGGGTTT	
		rbm24a sg4-R	AAACAAACCCCATCATCGACGGC	
		BFP-F	CCGGTCGCCACCATGAGCGAGCTGATTAAGG	
		BFP-R	GGTGGCGGCCGCTACCTCCACCTTAATTAAGCTTG	
		Vector-F	TAATTAAGGTGGAGGTAGCGGCCGCCAC	

		Vector-R	TCAGCTCGCTCATGGTGGCGACCGGTG	
		pCS2-*buc*-F	GTTCTTTTTGCAGGATCCCATATGGAAGGAATAAATAACACTTCAC	pCS2-*buc-GFP*
		pCS3-*buc*-R	CCCTTGCTCACCATCCCTCCGCCACCGCCACCGGTCCGACCTCCACCGTATCTTGTGCCTCTTTTCTTCA	
		Bg:GFP-F	GAGGCACAAGATACGGTGGAGGTCGGACCGGTGGCGGTGGCGGAGGGATGGTGAGCAAGGGCGAG	
		Bg:GFP-R	TAGAGGCTCGAGAGGCCTTGAATTCGAATTTACTTGTACAGCTCGTCCAT	
		pCS2-*rbm24a*-F	GCTACTTGTTCTTTTTGCAGGATCCCATATGCATACCACGCAAAAGG	pCS2-*rbm24a-GFP*
		pCS2-*rbm24a*-R690	CCCTTGCTCACCATTTGCATGCGTTCGGCTTG	
		pCS2-GFP-F	CCGAACGCATGCAAATGGTGAGCAAGGGC	
		pCS2-GFP-R720	TAGAGGCTCGAGAGGCCTTGAATTCGAATTTACTTGTACAGCTCGTCCAT	
		buc-F	CATCATTTTGGCAAAGAATTCCTCGACGATGGAAGGAATAAATAACACTTCACA	pT2AL200R150G-*buc-GFP*
		buc-R2.7K	AATGTATCTTATCATGTCTGGATCATCATTTACTTGTACAGCTCGTCC	
		pCS2-*buc*-F	GTTCTTTTTGCAGGATCCCATATGGAAGGAATAAATAACACTTCAC	pCS2-*buc*-myc
		pCS2-*buc*-R	AATGAGCTTTTGCTCCATAGCAATCCCTCCGCCACCGCCACCG	
		pCS2-*celf1*-F	TTCTTTTTGCAGGATCCCATATGAATGGGTCTCTGGAC	pCS2-*celf1*-myc
		pCS2-celf1-R	TTTGCTCCATAGCTTTAAATGGCTTGCTGTCATTTTTAG	
		pCS2-*tdrd6*-F	TTCTTTTTGCAGGATCCCATATGTGCTCCATTCCGGGAC	pCS2-*tdrd6*-myc
		pCS2-*tdrd6*-R	TTTGCTCCATAGCTTTAAATTCACGCTTTTCTTTTTCACTCTC	
		pCS2-*piwil1*-F	TTCTTTTTGCAGGATCCCATATGACAGGACGAGCAAGAG	pCS2-*piwil1*-myc
		pCS2-*piwil1*-R	TTTGCTCCATAGCTTTAAATAGGTAATACAGGAAGTCATCC	
		pCS2-*bucΔ1*-F	GAAGGCCTGTGAACTAGGATCCCATCGATTTAAAGC	pCS2-*bucΔ1*-myc
		pCS2-*buc*Δ1-R	AATCGATGGGATCCTAGTTCACAGGCCTTCAGACTCTC	

		pCS2-*bucΔ2*-F	GAACCACAGACTGGATCCCATCGATTTAAAGC	pCS2-*bucΔ2*-myc
		pCS2-*buc*Δ2-R	ATCGATGGGATCCAGTCTGTGGTTCATCTTCC	
		pCS2-*bucΔ3*-F	CCACAGACTAGGTCTTGGAGGCAAGTTACTGGA	pCS2-*bucΔ3*-myc
		pCS2-*bucΔ*3-R	CCAAGACCTAGTCTGTGGTTCATCTTCCTCATTGAC	
		pCS2-*bucΔ4*-F	GCCAAACCGTACAATGACTATGATGAGACCGAAT	pCS2-*bucΔ4*-myc
		pCS2-*bucΔ4*-R	GTCATTGTACGGTTTGGCACCACAGCTCGCC	
		nanos 3utr-F	TCAACATGGTGAAGCGGACATTGATGCTCCGGTAG	pCS2-*nanos3*
		nanos3 3utr-R	CTTGAATTCGAATCGATGGTTTAAAGTCTAGAGAAAATGTTTATATTTTCCTCACATTTTTCACTCC	
		nanos3 CDS-F	TACTTGTTCTTTTTGCAGTTGTTTTGATCGCGCGCTCGGAGAGCAAC	
		nanos3 CDS-R	TCCGCTTCACCATGTTGATTTGGCGTACACCGAGC	
		pCS2-RFP-F	GCATGCAAATGGCCTCCTCCGAGGACGTC	pCS2-*rbm24a-RFP*
		pCS2-RFP-R	AATTCGAATTTAGGCGCCGGTGGAGTGGCG	
		pCS2-RVector-F	GGCGCCTAAATTCGAATTCAAGGCCTCTCG	
		pCS2-RVector-R	GAGGCCATTTGCATGCGTTCGGCTTGCAG	
		PIC9K-rbm24-BFP.F	GAAGCTTACGTAGAATTCCCTAGGGCtATGCATACCACGCAAAAGg	pPIC9K-*rbm24a-BFP-6xHis*
		PIC9K-rbm24-BFP.R	GTCATGTCTAAGGCGAATTAATTCGTTAGTGATGGTGGTGATGGTGATTAAGCTTGTGCCCCAG	
		PIC9K-Buc-GFP.F	GAAGCTTACGTAGAATTCCCTAGGGCtATGGAAGGAATAAATAACACTTCAC	pPIC9K-*buc-GFP-6xHis*
		PIC9K-Buc-GFP.R	GTCATGTCTAAGGCGAATTAATTCGCTTAGTGATGGTGGTGATGGTGCTTGTACAGCTCGTCCATG	
		HTABGH-Buc-GFP.F	CGCGGATCTCGGTCCGAAACCGCCGCCGCCACCATGGAAG	pFastBac-HTA2-buc-GFP-6xHis
		HTABGH-Buc-GFP.R	GATTAGTGGTGGTGGTGGTGGTGCTTGTACAGCTCGTCCATG	

		HTABGH-Vector.F	CAAGCACCACCACCACCACCACTAATCAGCCATACCACATTTG	
		HTABGH-Vector.R	GGCGGCGGTTTCGGACCGAGATCCGCGCCCGATG	
		HTARBH: RBH.F	GGATCTCGGTCCGAAACCATGCATACCACGCAAAAGG	pFastBac-HTA2-rbm24a-BFP-6xhis
		HTARBH: RBH.R1.4K	CTGATTAGTGGTGGTGGTGGTGGTGATTAAGCTTGTGCCCCAG	
		HTARBH: VEC.F	TAATCACCACCACCACCACCACTAATCAGCCATACCACATTTG	
		HTARBH: Vec.R4.7	GGTATGCATGGTTTCGGACCGAGATCCGCGCCCGATG	
		pETDuet1-Buc.F	TTTAAGAAGGAGATATACCGATGGAAGGAATAAATAACACTTCACAACCAAT	pETDuet1-buc-RFP-6xHis
		pETDuet1-Buc.R	CCGCCACCGGTCCGACCTCCACCGTATCTTGTGCCTCTTTTCT	
		pETDuet1-RFP.F	TCGGACCGGTGGCGGTGGCGGAGGGATGGCCTCCTCCGAGGACGT	
		pETDuet1-RFP.R	TTAGTGATGGTGGTGGTGATGGGCGCCGGTGGAGTGGCGG	
	***zGrad* sequence**	*zGrad* DNA sequence	ATGGAAACCGAAATGGAGGACAAGACACTGGAGCAGATGAACACGTCAGTGATGGACCCTCAGACCGCTGATCGGAGTCCGAAGATCACTCTCATCAAGTCAACATTCATCTGTCCACAGGTTTCTAACGGACCTCTGACCGGTTCTCGGAAGCGTCCAAGCGAAGGTAACTATGAGAAAGAGAAAGACGTGTGCATTCAGCTCTTCGACCAGTGGTCAGAGGCAGATCAGGTGGAGTTTGTTGAGCACCTGATTTCCAGGATGTGTCATTACCAACACGGACACATCAACTCTTATCTCAAACCTATGCTGCAAAGAGACTTCATTACCGCATTGCCAGCTCAGGGATTGGACCATATCGCCGAGAACATACTGTCTTTCTTGGATGCTAGAAGTCTGTGTAGTGCTGAACTGGTGTGCAAAGAGTGGCAGAGGGTCATCTCCGAGGGAATGCTGTGGAAGAAACTGATCGAACGCATGGTGAGAACCGATCCACTCTGGAAGGGACTGTCCGAGAGACATCAGTGGGAGAAGTACCTGTTCAAGAACAGAACCACTGAGGTGCCTCCAAACTCATATTACCGCTCACTGTATCCAAAGATCATCCAAGACATCGAGACTATTGAGGCCAACTGGAGGTGTGGCAGACACATGGATCAGGTTCAGCTCGTGGAGTCAGGCGGAGCATTGGTACAGCCAGGCGGATCACTGAGGCTGAGCTGCGCCGCATCTGGCTTTCCAGTGAACCGCTACAGCATGAGGTGGTATCGTCAAGCTCCAGGCAAAGAGAGAGAGTGGGTCGCTGGCATGAGCAGTGCTGGAGATAGGTCTAGTTACGAAGATTCCGTGAAAGGCAGATTCACCATTAGCAGGGATGATGCTCGCAACACTGTGTACCTGCAGATGAACTCACTGAAACCAGAAGATACTGCTGTGTACTACTGCAATGTGAACGTGGGATTCGAATACTGGGGACAGGGTACACAGGTGACCGTTTCTTCCATCGATTCGAATTCAAGGCCTCTCGAGCCTCTAGAACTATAG	

Appendix Table S2: Alternative splicing analysis of germ plasm-related mRNAs.

sibling vs M*rbm24a* at 4-cell

EventType	TotalEvents JC	TotalEvents JCEC	SignificantEvents JC	SignificantEvents JCEC
SE	7430	7466	214	220
A5SS	6624	6626	66	71
A3SS	8219	8222	147	145
MXE	634	642	16	17
RI	974	988	32	30

JC

gene_sym	SE	A5SS	A3SS	MXE	RI
buc	NA	NA	1.00000|0.36102	NA	NA
nanos3	NA	NA	NA	NA	NA
tdrd7a	NA	NA	NA	NA	NA
ddx4	1.00000|1.00000|1.00000	1.00000|1.00000|1.00000|1.00000|1.00000|1.00000|1.00000	1.00000|1.00000|1.00000|1.00000|1.00000|1.00000	NA	1
kif5ba	NA	1	1	NA	NA
dnd1	NA	NA	NA	NA	NA
ca15b	1.00000|1.00000|1.00000|1.00000	1	1.00000|1.00000|1.00000|1.00000|1.00000|1.00000|1.00000	NA	1
celf1	1.00000|0.22059	1.00000|1.00000|1.00000|1.00000	1.00000|1.00000|0.00102|1.00000|1.00000|1.00000	NA	0.013
rgs14a	NA	1	NA	NA	NA
dazl	1.00000|1.00000|1.00000|1.00000|1.00000|1.00000|1.00000	1.00000|1.00000|1.00000	1.00000|1.00000|1.00000	1	NA
hook2	1	1.00000|1.00000|1.00000|1.00000	1.00000|1.00000	NA	NA
tdrd6	1.00000|1.00000|0.56102|1.00000|1.00000|1.00000|1.00000	1.00000|1.00000|0.30090|1.00000|1.00000|1.00000|1.00000	1.00000|0.83661|1.00000|1.00000	1.00000|1.00000	0.78001|1.00000
gra	1	1	NA	NA	NA

h1m	1.00000|1.00000|1.00000|1.00000|0.50870|0.10053|1.00000|1.00000	1.00000|1.00000|1.00000|1.00000|1.00000|1.00000|1.00000	0.38262|0.33517|1.00000|1.00000|1.00000|1.00000|1.00000|1.00000|1.00000|1.00000|1.00000|1.00000|1.00000|1.00000	NA	1.00000|1.00000|1.00000

JCEC

gene_sym	SE	A5SS	A3SS	MXE	RI
buc	NA	NA	1.00000|0.36235	NA	NA
nanos3	NA	NA	NA	NA	NA
tdrd7a	NA	NA	NA	NA	NA
ddx4	1.00000|1.00000|1.00000	1.00000|1.00000|1.00000|1.00000|1.00000|1.00000|1.00000	1.00000|1.00000|1.00000|1.00000|1.00000|1.00000	NA	1
kif5ba	NA	1	1	NA	NA
dnd1	NA	NA	NA	NA	NA
ca15b	1.00000|1.00000|1.00000|1.00000	1	1.00000|1.00000|1.00000|1.00000|1.00000|1.00000|1.00000	NA	1
celf1	1.00000|0.21691	1.00000|1.00000|1.00000|1.00000	1.00000|1.00000|0.00106|1.00000|1.00000|1.00000	NA	1
rgs14a	NA	1	NA	NA	NA
dazl	1.00000|1.00000|1.00000|1.00000|1.00000|1.00000|1.00000	1.00000|1.00000|1.00000	1.00000|1.00000|1.00000	1	NA
hook2	1	1.00000|1.00000|1.00000|1.00000	1.00000|1.00000	NA	NA
tdrd6	1.00000|1.00000|0.54668|1.00000|1.00000|1.00000|1.00000	1.00000|1.00000|0.29934|1.00000|1.00000|1.00000|1.00000	1.00000|0.83337|1.00000|1.00000	1.00000|1.00000	0.77420|1.00000
gra	1	1	NA	NA	NA
h1m	1.00000|1.00000|1.00000|1.00000|0.49566|0.09799|1.00000|1.00000	1.00000|1.00000|1.00000|1.00000|1.00000|1.00000|1.00000	0.38400|0.33760|1.00000|1.00000|1.00000|1.00000|1.00000|1.00000|1.00000|1.00000|1.00000|1.00000|1.00000|1.00000	NA	1.00000|1.00000|1.00000
