# Supplementary material for: Rbm24a dictates mRNA recruitment for germ granule assembly in zebrafish
Source: EMBO J. 2025 Apr 25;44(11):3121–49. doi: 10.1038/s44318-025-00442-z (PMC12130248; doi:10.1038/s44318-025-00442-z)
Supplement: Supplementary file 3 — Data Set EV1 [file 44318_2025_442_MOESM3_ESM.zip › result/PD 2.4 表头说明.pdf]

附件 1_蛋白质鉴定列表

表头	定义	描述
Accession	蛋白质登录号	蛋白质序列数据库（FASTA database）中的蛋白质编号
Description	蛋白质信息描述	基于蛋白质序列的数据库中的蛋白质功能描述。搜库经常使用两类数据库：公共库（用的比较多的是 UniProtKB）与自建库（由转录组数据库翻译而来的蛋白质数据库）。UniProtKB 数据库（www.uniprot.org）的命名格式为：>db|UniqueIdentifier|EntryName ProteinName OS=OrganismName [GN=GeneName] PE=Protein Existence SV=SequenceVersion。自建库中若仅有蛋白质序列，没有功能注释，则查库结果的 Description 中没有蛋白质功能描述。
Coverage	肽段覆盖率	鉴定到的氨基酸数目占蛋白质总氨基酸数目的比例。
Unique Peptides	唯一肽段数	用于定量的唯一肽段数目
Peptides	肽段数	用于定性的肽段数目
PSMs	匹配到肽段的质谱图谱总数	全称是 peptide spectrum matches，为该蛋白质组的所有肽段匹配到全部质谱图的数量
AAs	氨基酸个数	蛋白质的氨基酸总数
MW [kDa]	分子量	蛋白质的理论分子量。该分子量是软件根据数据库中的蛋白质序列计算得到的。如果用以参加计算的蛋白质序列不是完整的全长序列，比如由转录组翻译而来的蛋白质序列，由此计算得到的分子量会小于完整蛋白质的分子量。
calc. pI	等电点	蛋白质的理论等电点
Abundances (Grouped)	蛋白相对表达量	根据质谱响应强度得到的蛋白定量值

附件 2_肽段鉴定列表

表头	定义	描述
Annotated Sequence	肽段氨基酸序列	描述肽段氨基酸的组成，其中小写字母为修饰氨基酸
Modifications	修饰	描述修饰氨基酸、位置及修饰方式
# Protein Groups	蛋白组个数	该肽段匹配到的蛋白组个数
Proteins	蛋白个数	该肽段匹配到的蛋白个数

PSMs	匹配到肽段的质谱图谱总数	全称是peptide spectrum matches，为该肽段匹配到全部质谱图的数量。
Master Protein Accessions	蛋白质登录号	蛋白质序列数据库（FASTA database）中的蛋白质编号
Positions in Master Proteins	在蛋白序列中的位置	鉴定到的肽段序列在蛋白序列中的位置
Modifications in Master Proteins	修饰	描述修饰氨基酸在蛋白序列的位置及修饰方式
# Missed Cleavages	漏切位点	肽段在酶解过程中有些位点可能发生漏切
Theo. MH+ [Da]	肽段分子量	肽段理论分子量
Abundances (Grouped)	相对表达量	肽段在质谱上的响应强度
Charge (by Search Engine): Mascot	电荷	肽段电荷数
DeltaM [ppm] (by Search Engine): Mascot	理论分子量和实验分子量的差值	肽段理论分子量和实验测得分子量的差异
RT [min] (by Search Engine): Mascot]	保留时间	全称为 retention time，指被分离样品组分从进样开始到柱后出现该组分浓度极大值时的时间，即从进样开始到出现某组分色谱峰的顶点时为止所经历的时间，称为此组分的保留时间，用 RT 表示，常以分（min）为时间单位。
Ions Score (by Search Engine): Mascot	肽段得分	Mascot 肽段得分
