# Supplementary figures and images for: Rbm24a dictates mRNA recruitment for germ granule assembly in zebrafish (part 1 of 3)
Source: EMBO J. 2025 Apr 25;44(11):3121–49. doi: 10.1038/s44318-025-00442-z (PMC12130248; doi:10.1038/s44318-025-00442-z)

RT: 0.00 - 65.00

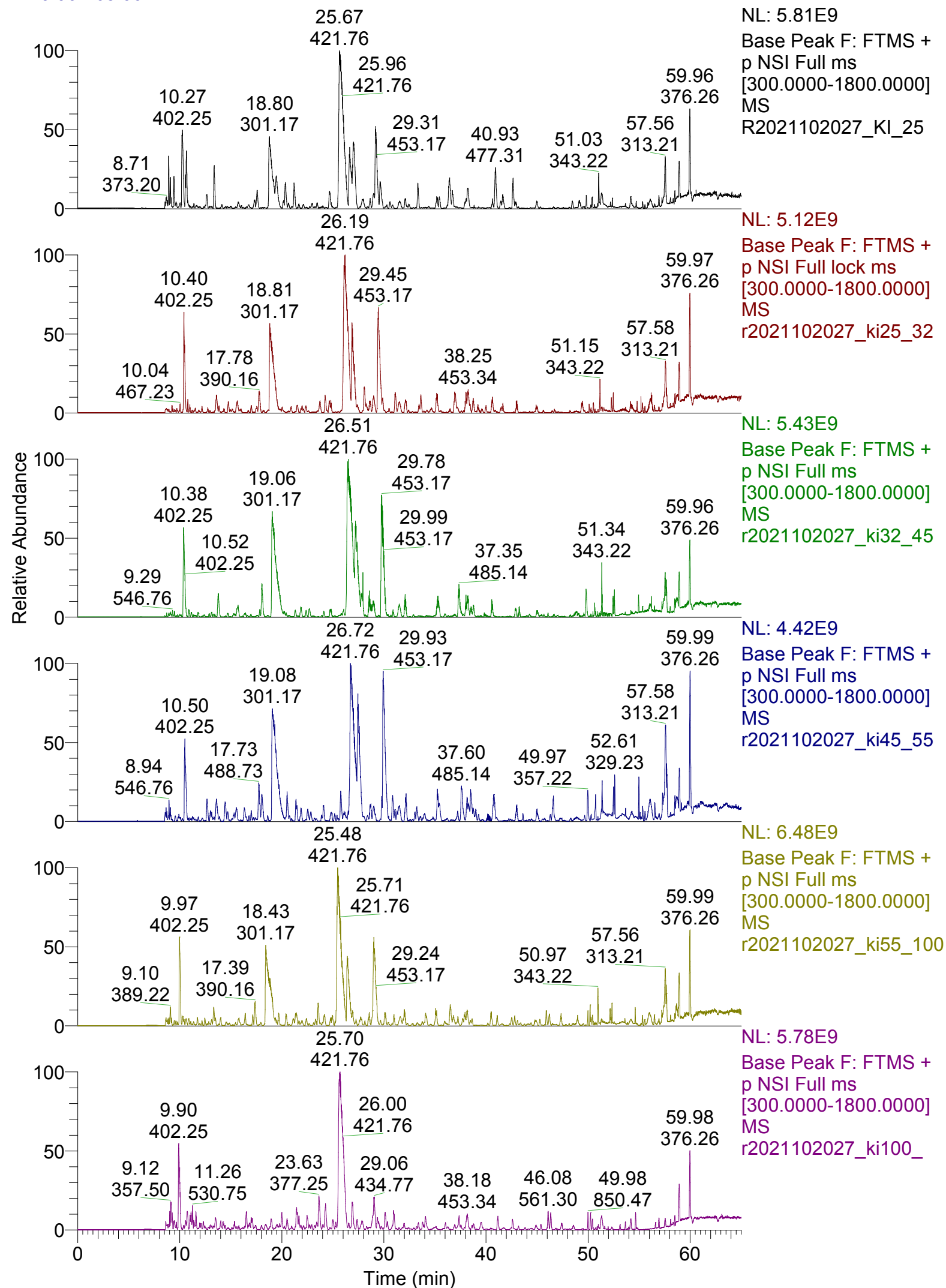

Supplement: Supplementary file 3 — Data Set EV1 [file 44318_2025_442_MOESM3_ESM.zip › Base Peak/basepeak1.pdf]

RT: 0.00 - 65.00

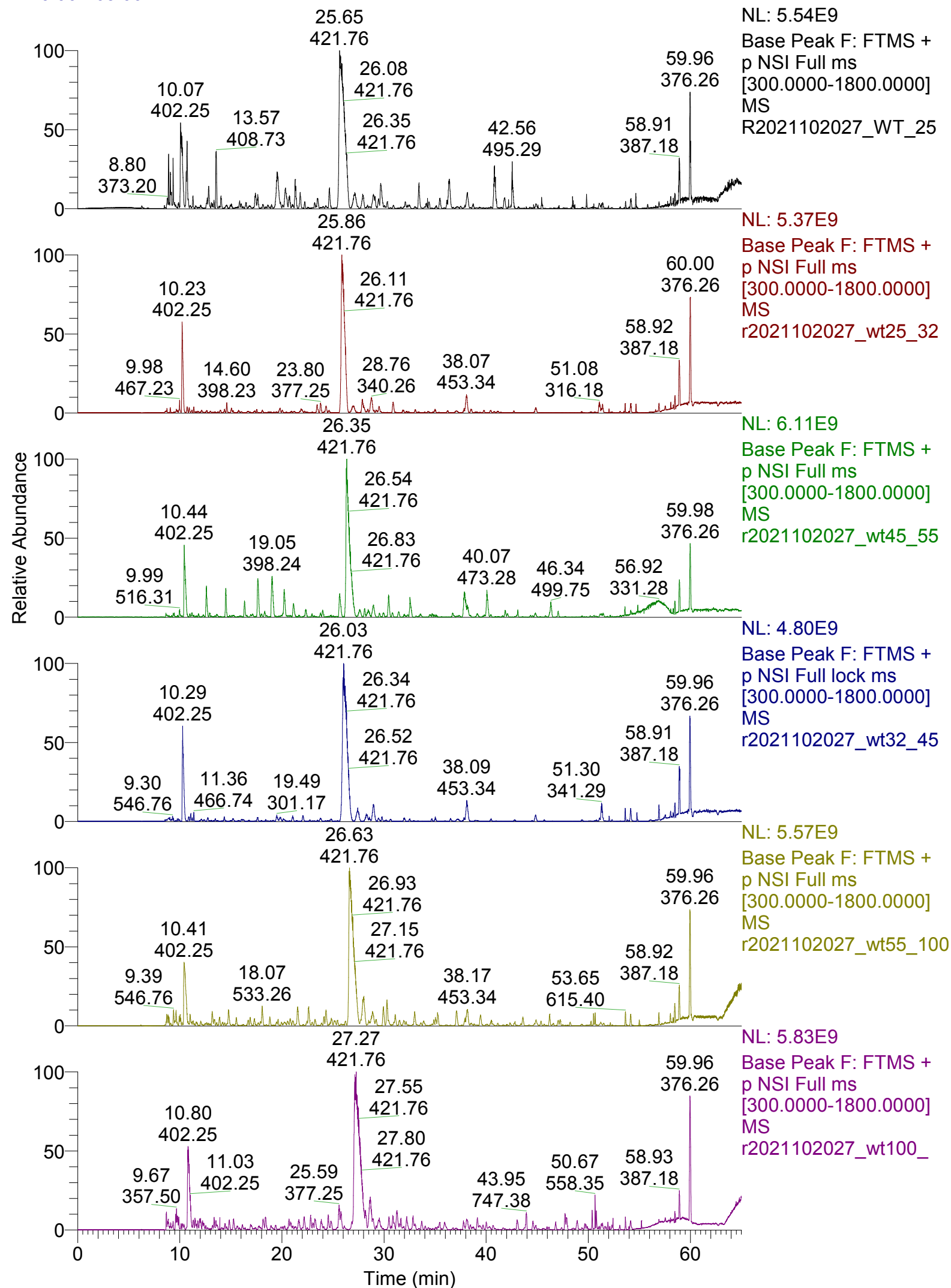

Supplement: Supplementary file 3 — Data Set EV1 [file 44318_2025_442_MOESM3_ESM.zip › Base Peak/basepeak2.pdf]

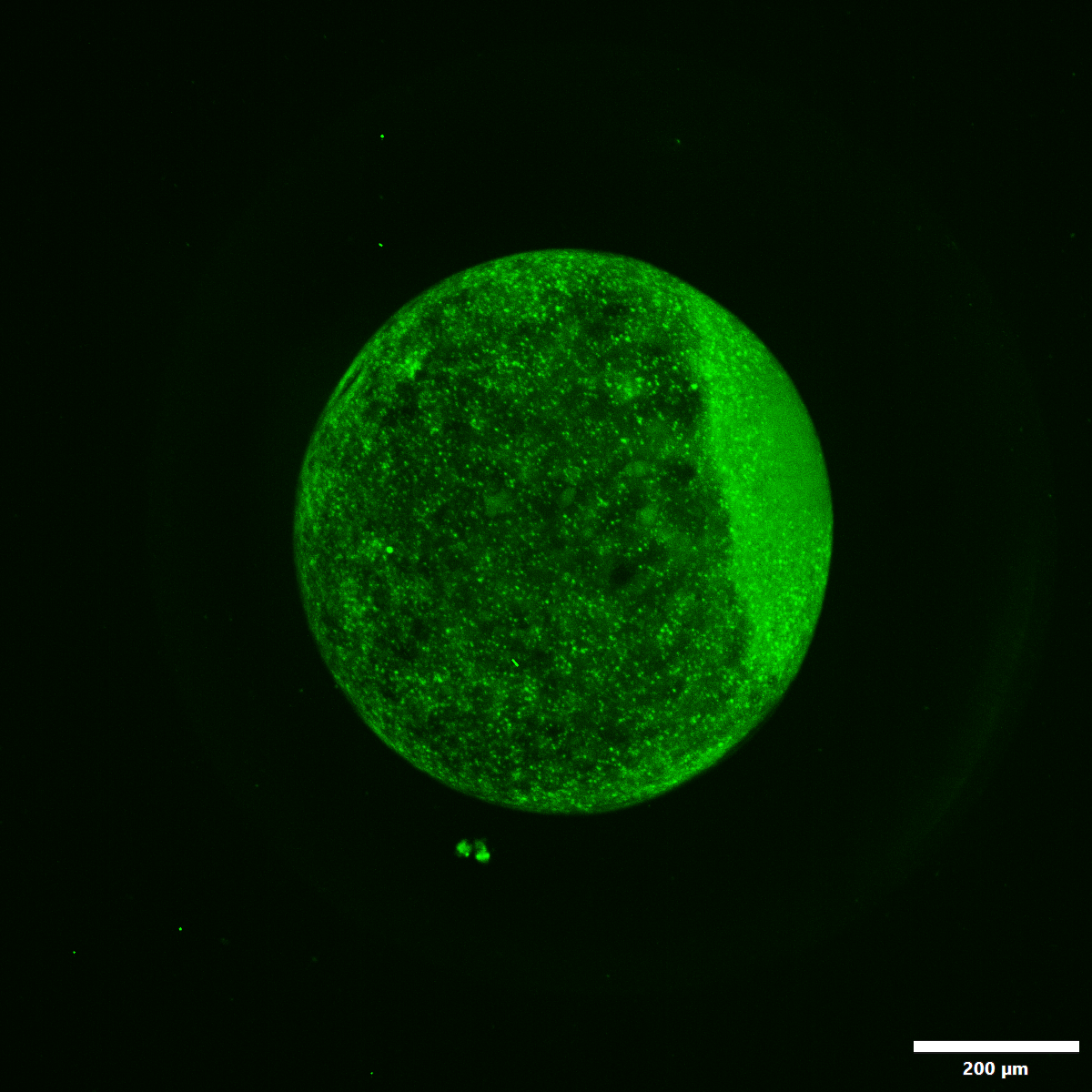

Supplement: Supplementary file 12 — Source data Fig. 1 [file 44318_2025_442_MOESM12_ESM.zip › Figure_1/Figure_1B/1-cell.tif]

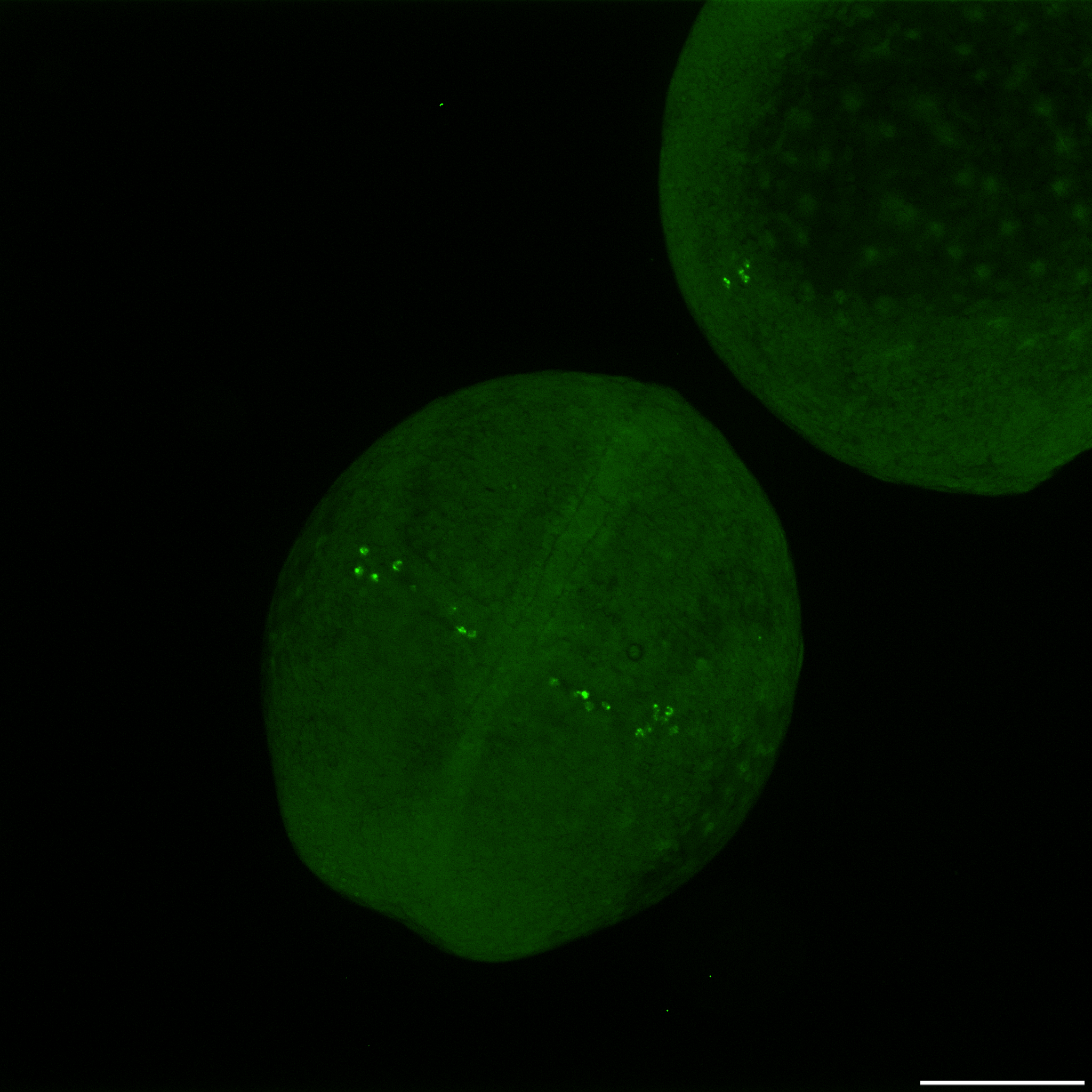

Supplement: Supplementary file 12 — Source data Fig. 1 [file 44318_2025_442_MOESM12_ESM.zip › Figure_1/Figure_1B/1-somite.tif]

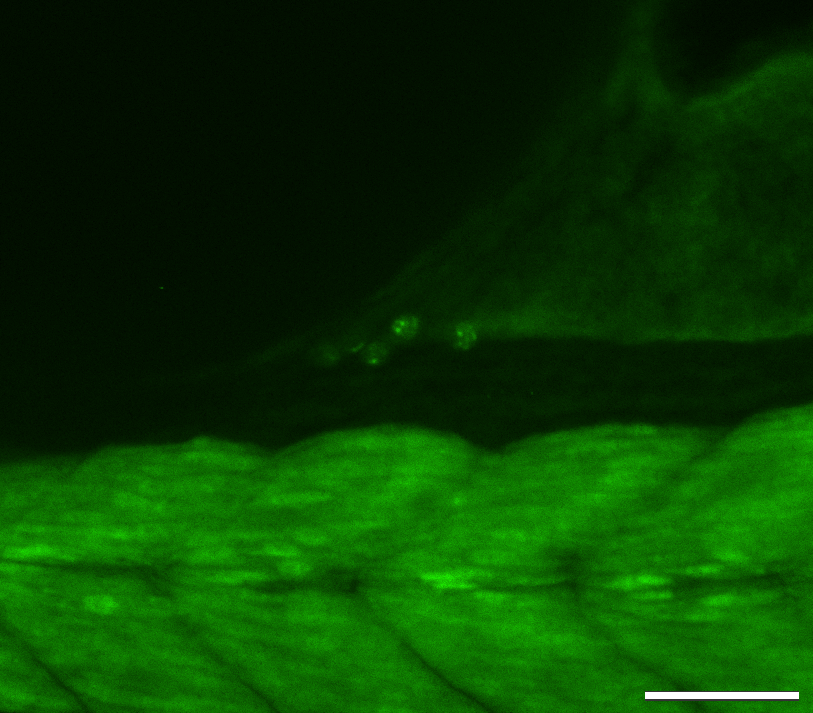

Supplement: Supplementary file 12 — Source data Fig. 1 [file 44318_2025_442_MOESM12_ESM.zip › Figure_1/Figure_1B/24 hpf enlarged .tif]

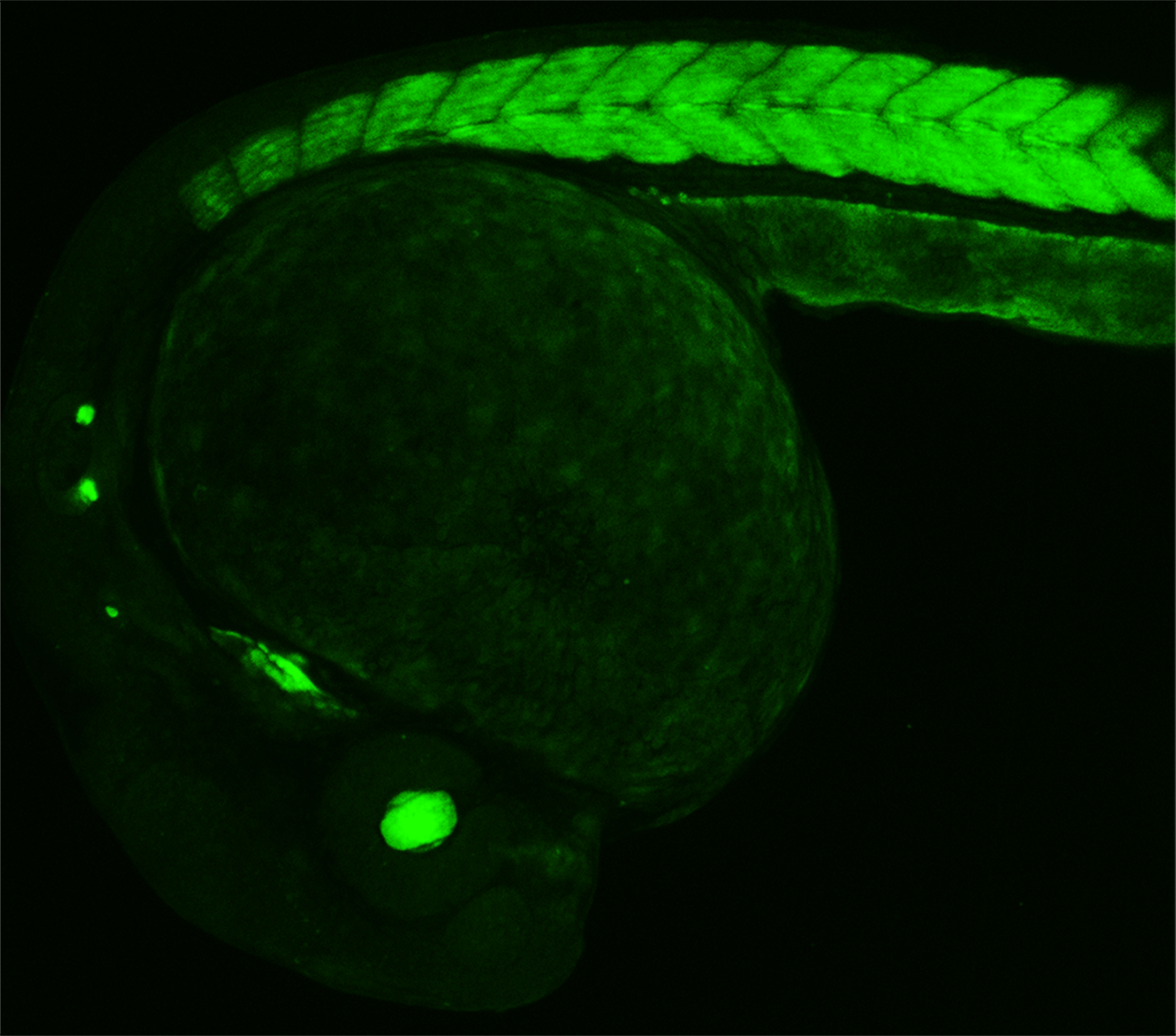

Supplement: Supplementary file 12 — Source data Fig. 1 [file 44318_2025_442_MOESM12_ESM.zip › Figure_1/Figure_1B/24 hpf.tif]

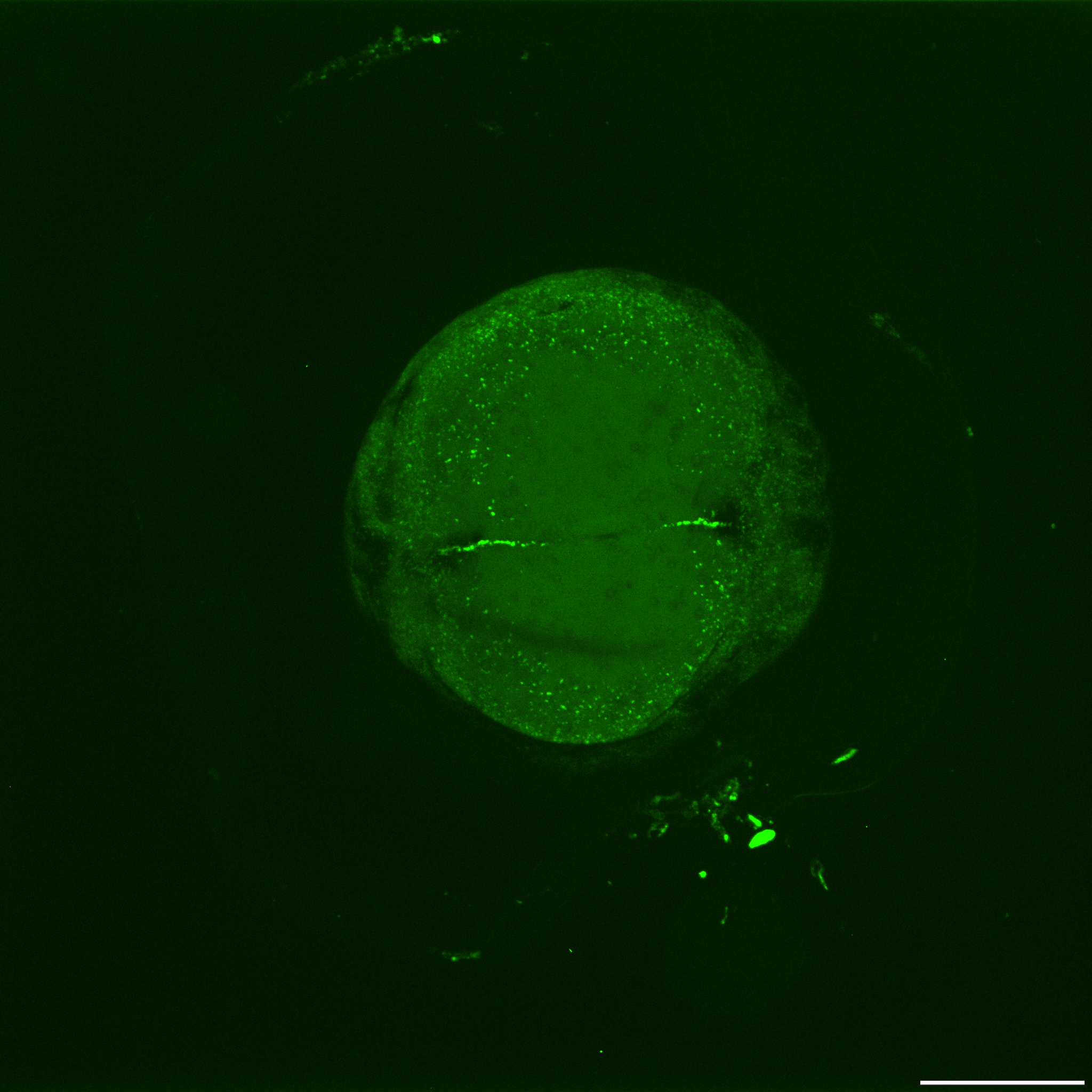

Supplement: Supplementary file 12 — Source data Fig. 1 [file 44318_2025_442_MOESM12_ESM.zip › Figure_1/Figure_1B/2-cell.tif]

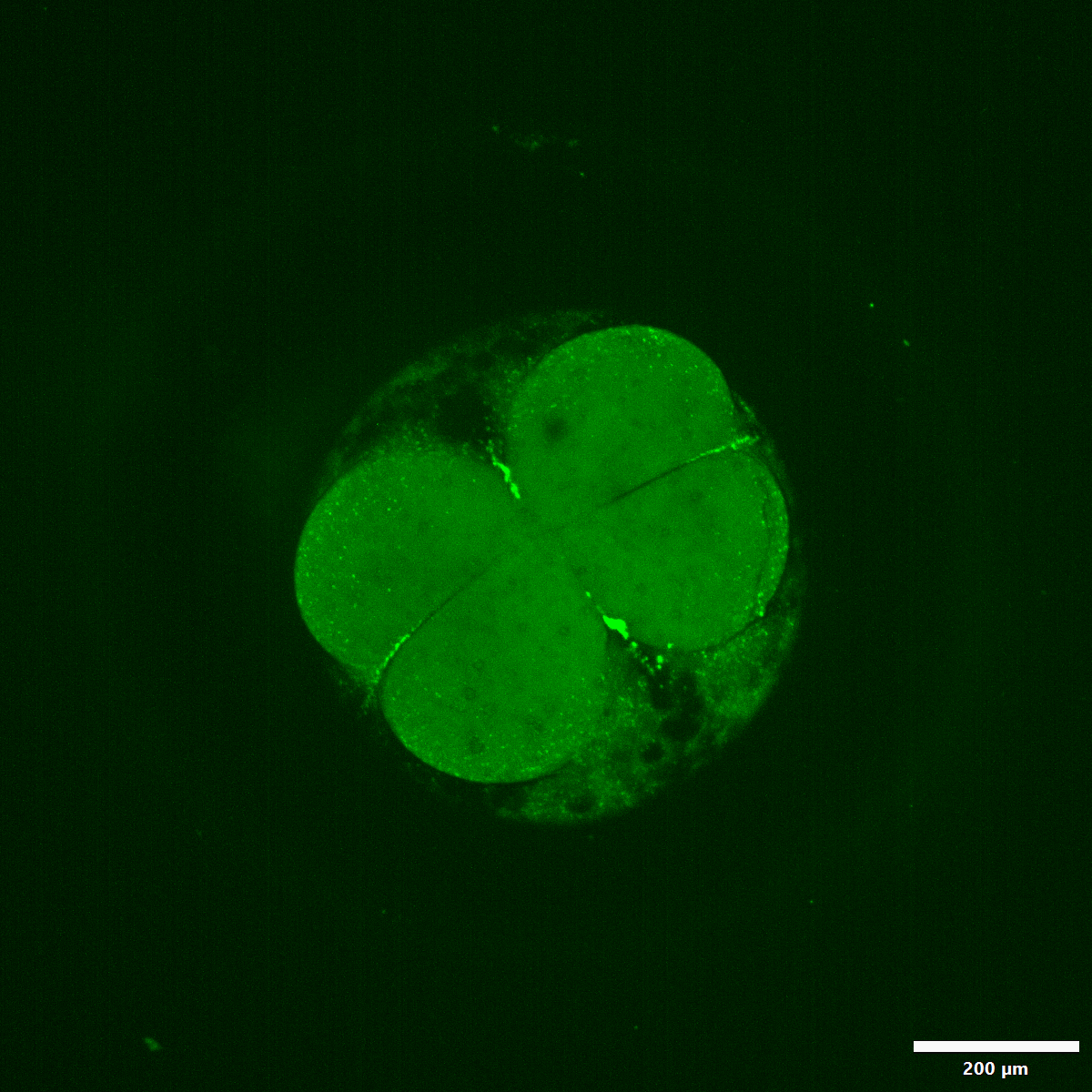

Supplement: Supplementary file 12 — Source data Fig. 1 [file 44318_2025_442_MOESM12_ESM.zip › Figure_1/Figure_1B/4-cell.tif]

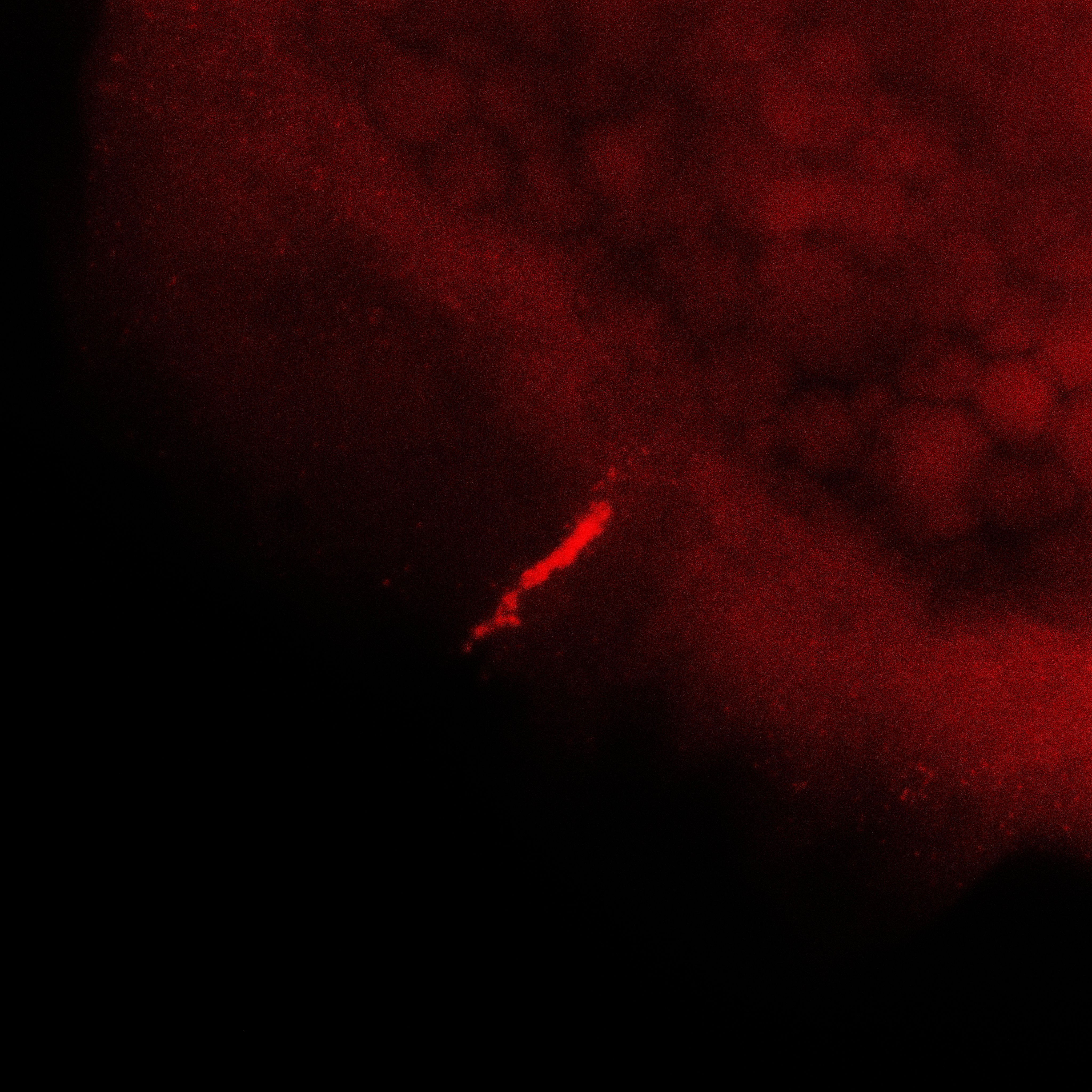

Supplement: Supplementary file 12 — Source data Fig. 1 [file 44318_2025_442_MOESM12_ESM.zip › Figure_1/Figure_1C/dazl.tif]

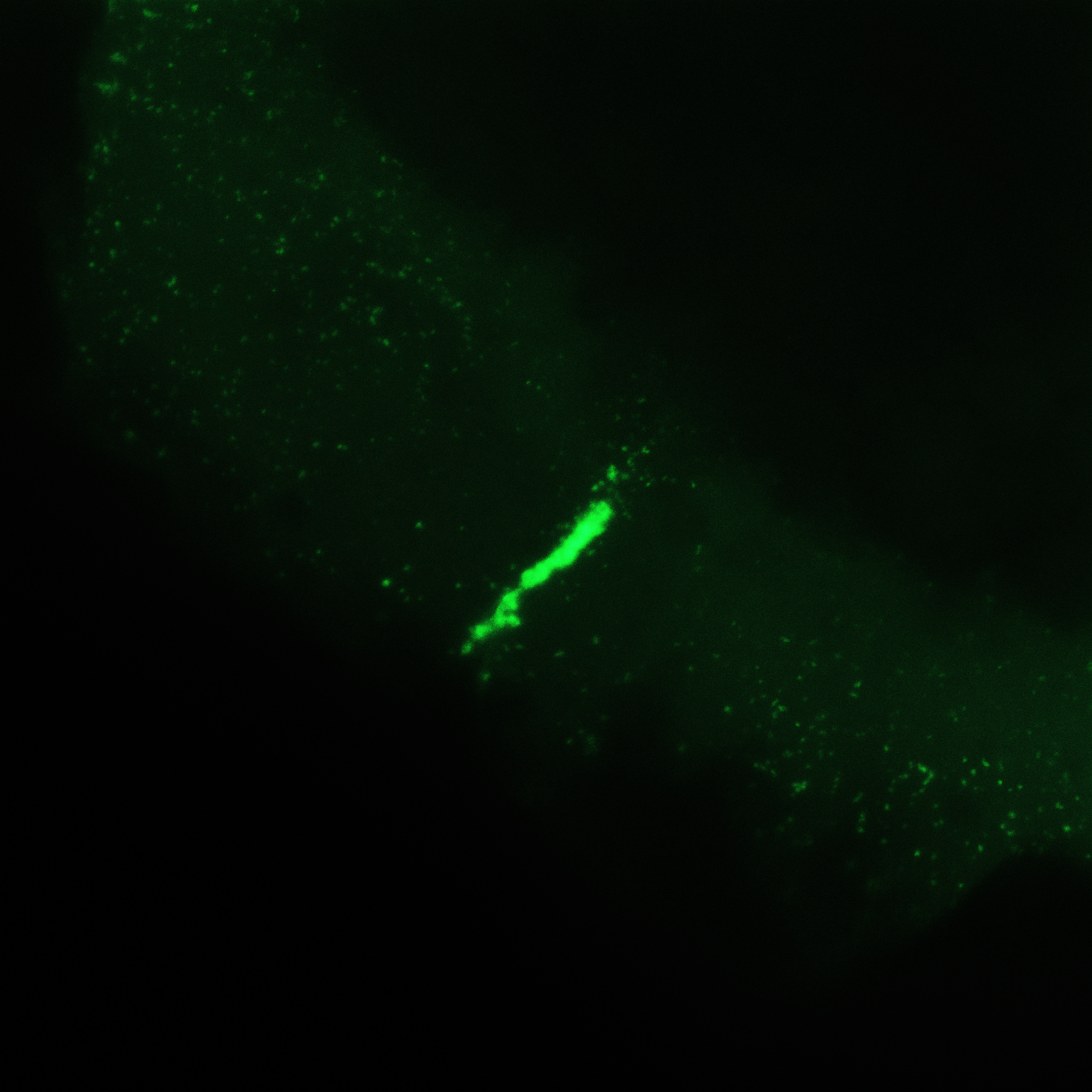

Supplement: Supplementary file 12 — Source data Fig. 1 [file 44318_2025_442_MOESM12_ESM.zip › Figure_1/Figure_1C/dazl-rbm24a-GFP .tif]

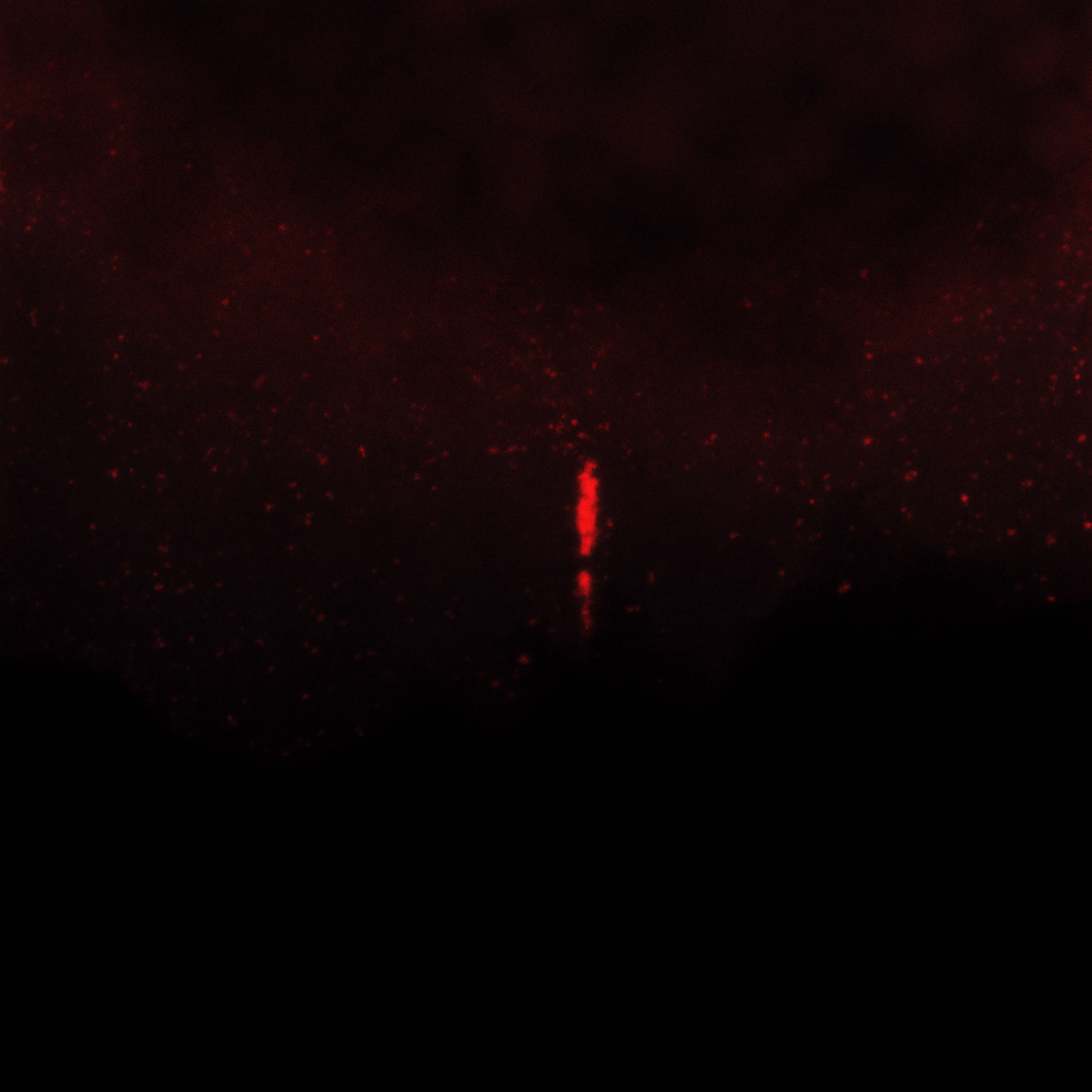

Supplement: Supplementary file 12 — Source data Fig. 1 [file 44318_2025_442_MOESM12_ESM.zip › Figure_1/Figure_1C/ddx4-FISH.tif]

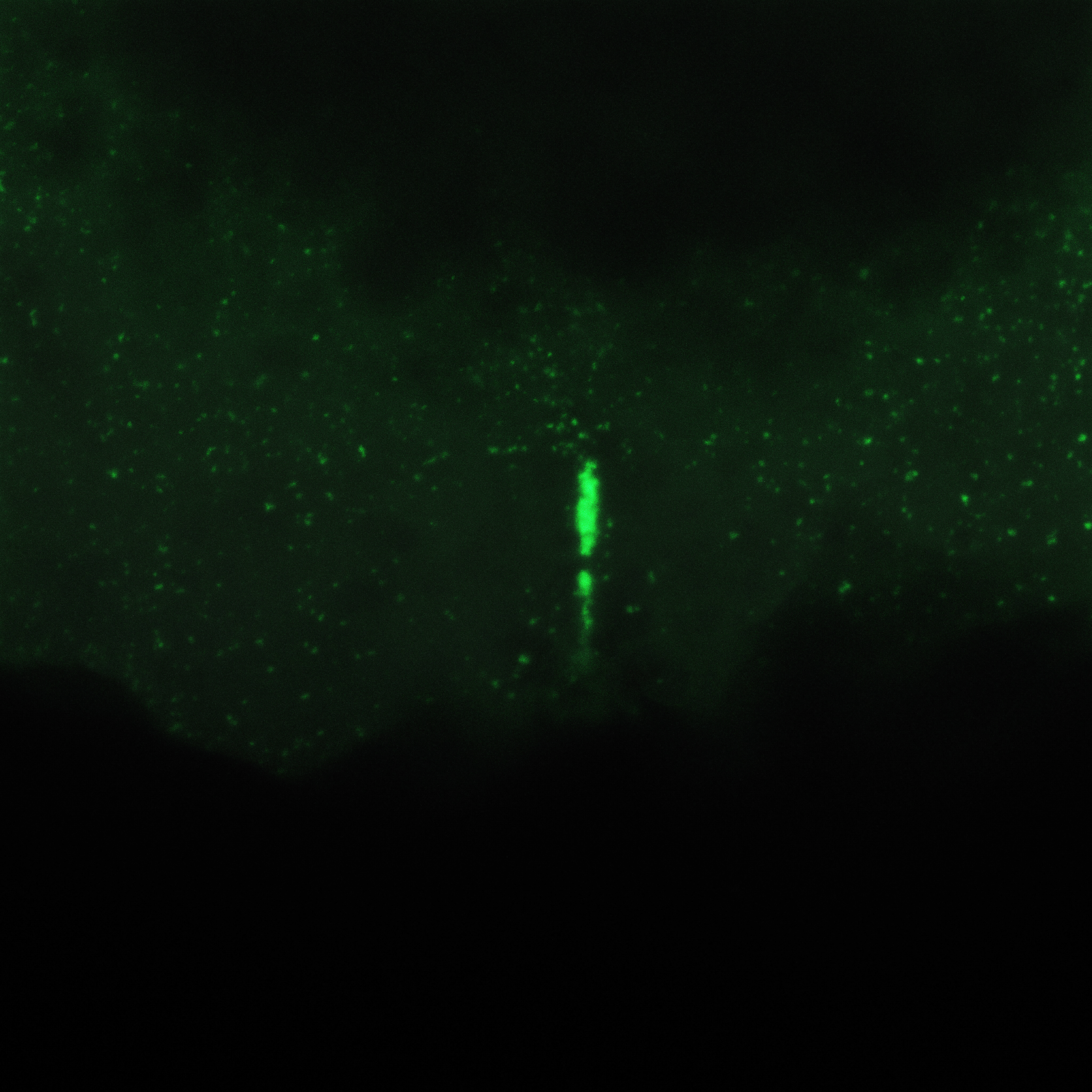

Supplement: Supplementary file 12 — Source data Fig. 1 [file 44318_2025_442_MOESM12_ESM.zip › Figure_1/Figure_1C/ddx4-rbm24a-GFP .tif]

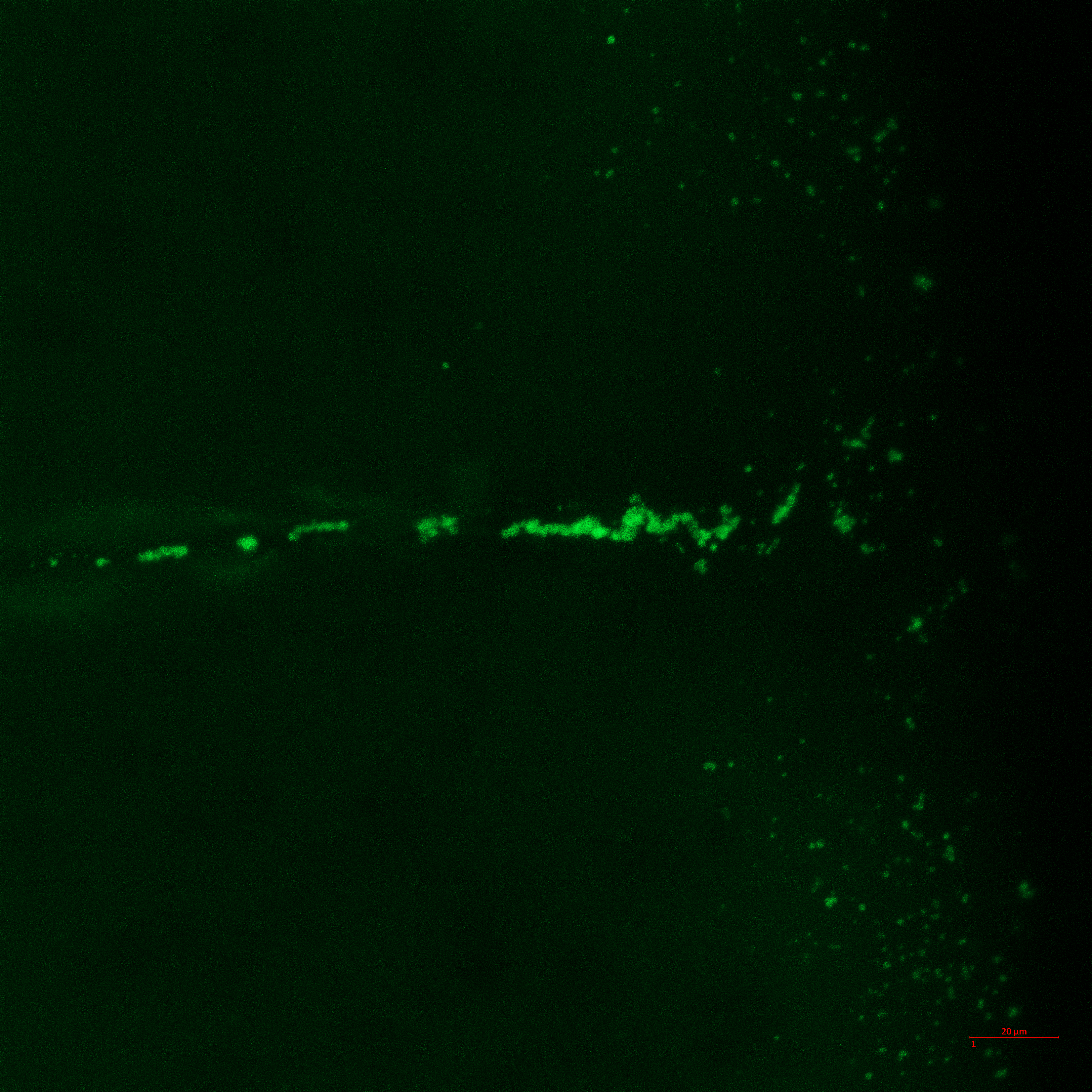

Supplement: Supplementary file 12 — Source data Fig. 1 [file 44318_2025_442_MOESM12_ESM.zip › Figure_1/Figure_1C/IF-rbm24a-GFP.tif]

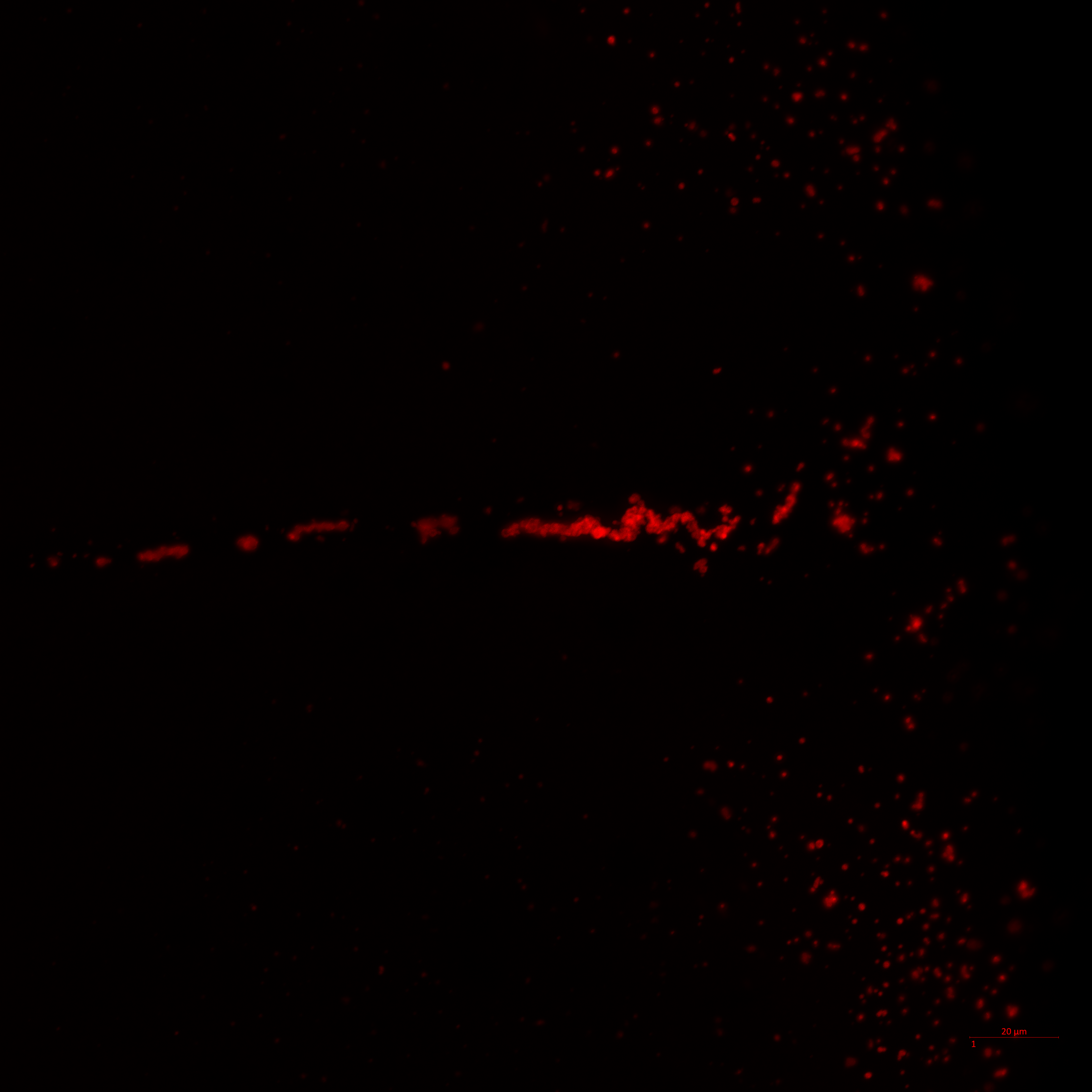

Supplement: Supplementary file 12 — Source data Fig. 1 [file 44318_2025_442_MOESM12_ESM.zip › Figure_1/Figure_1C/Piwil1.tif]

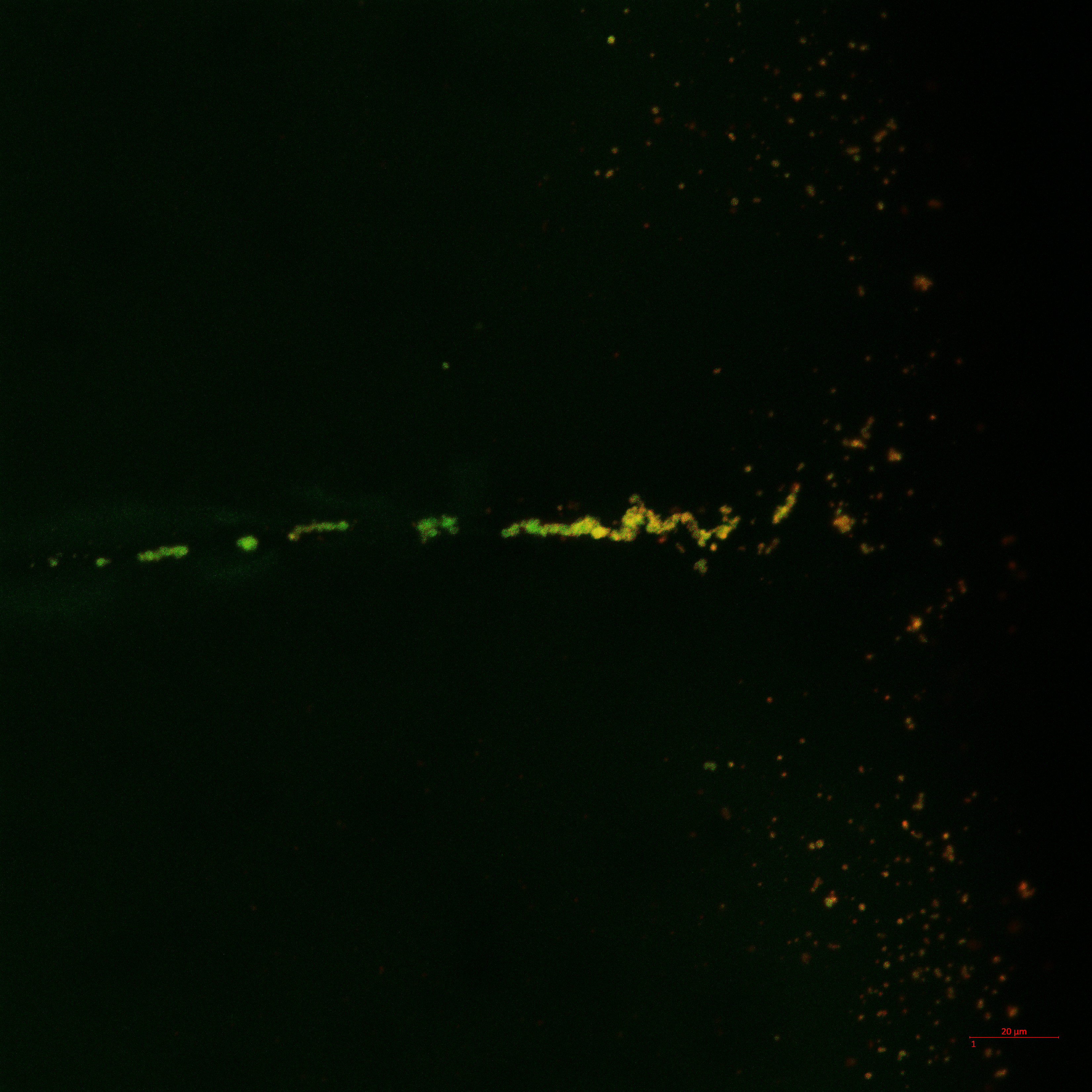

Supplement: Supplementary file 12 — Source data Fig. 1 [file 44318_2025_442_MOESM12_ESM.zip › Figure_1/Figure_1C/Piwil1-rbm24a-GFP merge.tif]

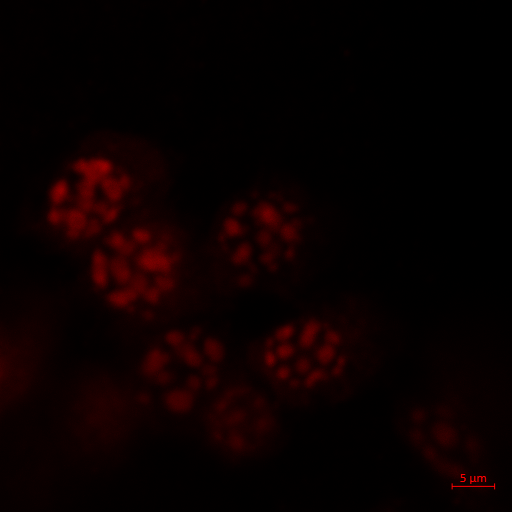

Supplement: Supplementary file 12 — Source data Fig. 1 [file 44318_2025_442_MOESM12_ESM.zip › Figure_1/Figure_1D/ddx4.tif]

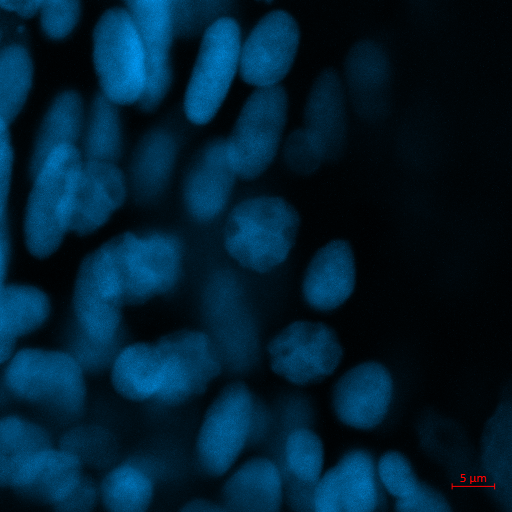

Supplement: Supplementary file 12 — Source data Fig. 1 [file 44318_2025_442_MOESM12_ESM.zip › Figure_1/Figure_1D/Hochest.tif]

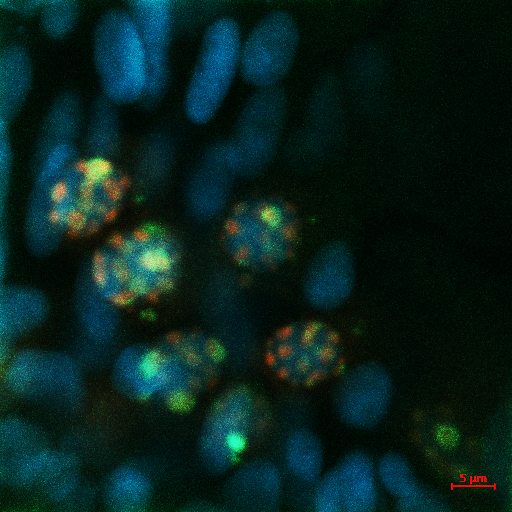

Supplement: Supplementary file 12 — Source data Fig. 1 [file 44318_2025_442_MOESM12_ESM.zip › Figure_1/Figure_1D/merge.tif]

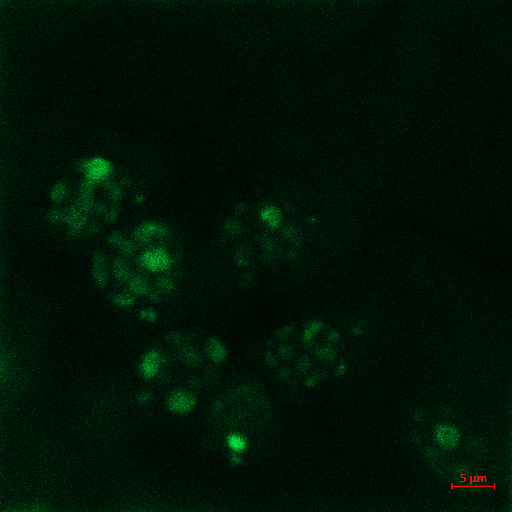

Supplement: Supplementary file 12 — Source data Fig. 1 [file 44318_2025_442_MOESM12_ESM.zip › Figure_1/Figure_1D/rbm24a-GFP.tif]

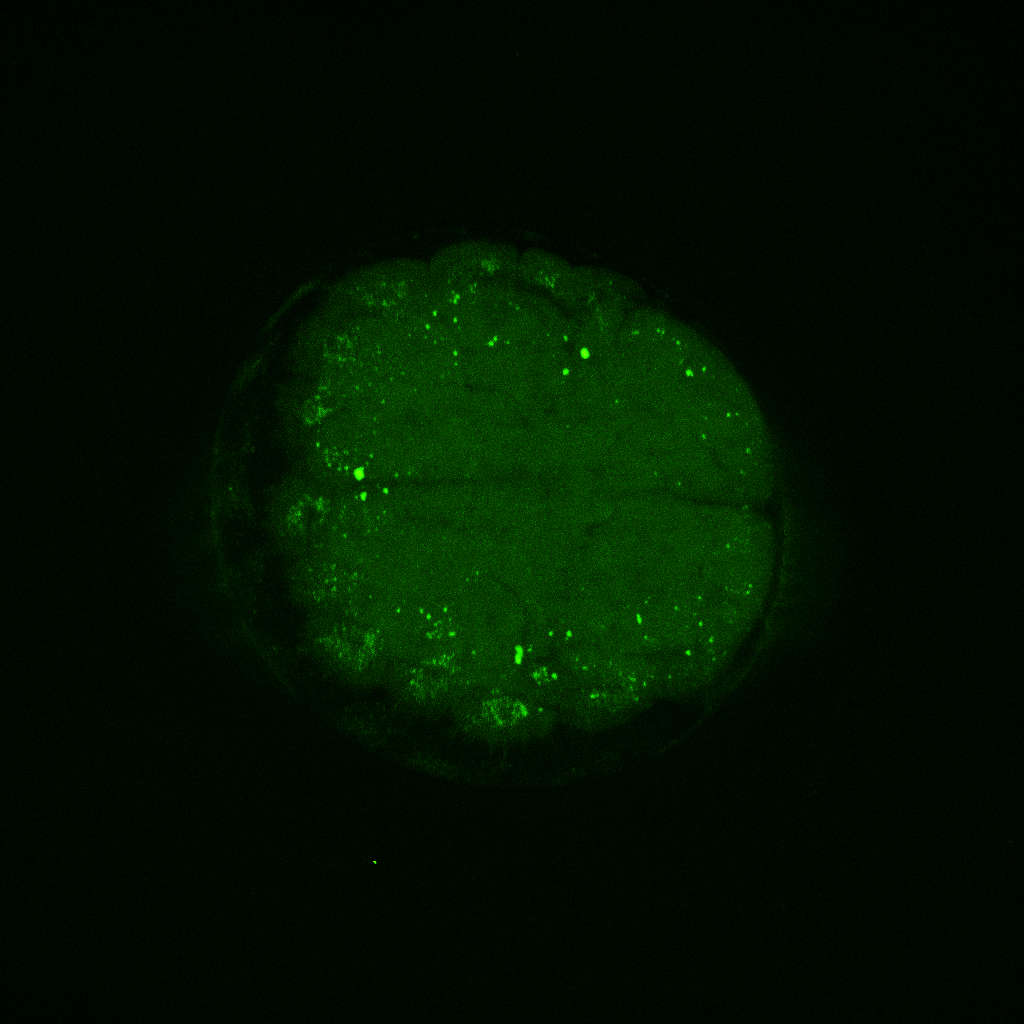

Supplement: Supplementary file 12 — Source data Fig. 1 [file 44318_2025_442_MOESM12_ESM.zip › Figure_1/Figure_1E/128-cell.tif]

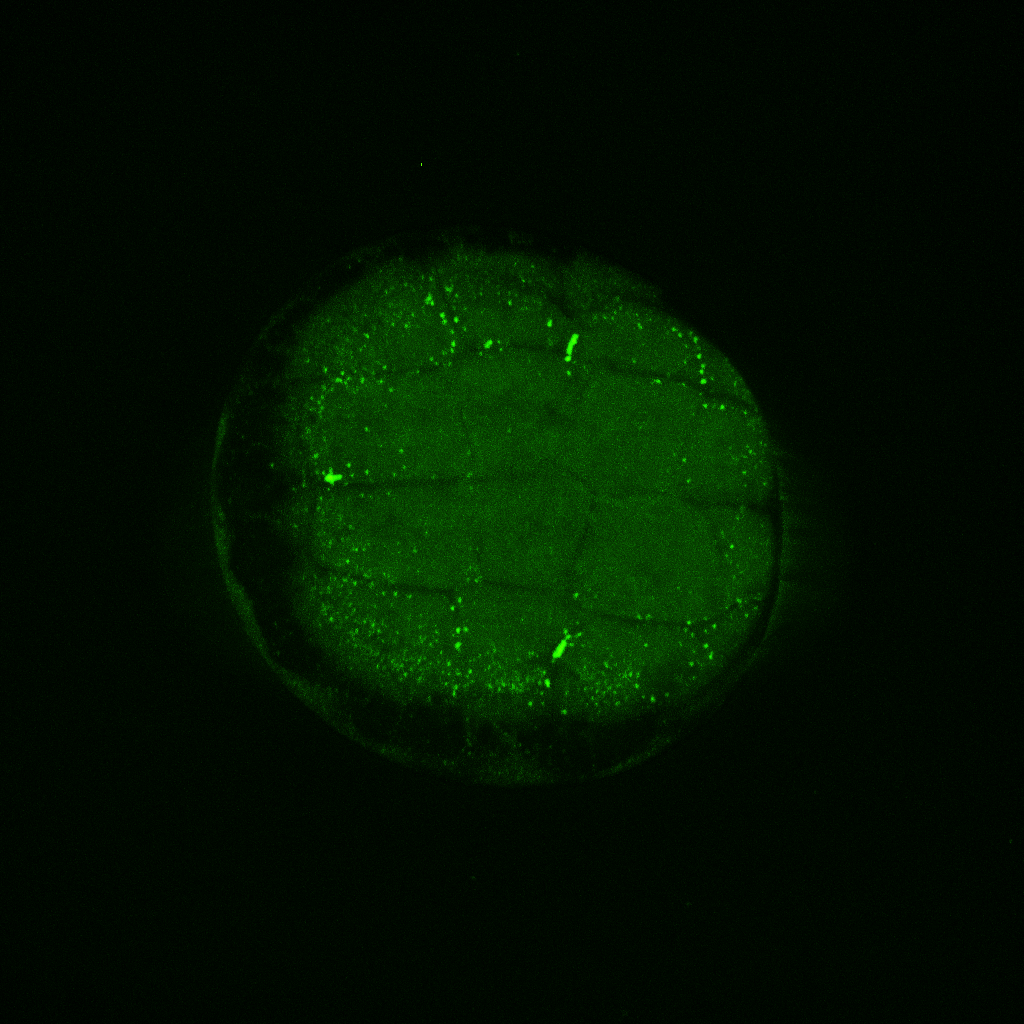

Supplement: Supplementary file 12 — Source data Fig. 1 [file 44318_2025_442_MOESM12_ESM.zip › Figure_1/Figure_1E/16-cell.tif]

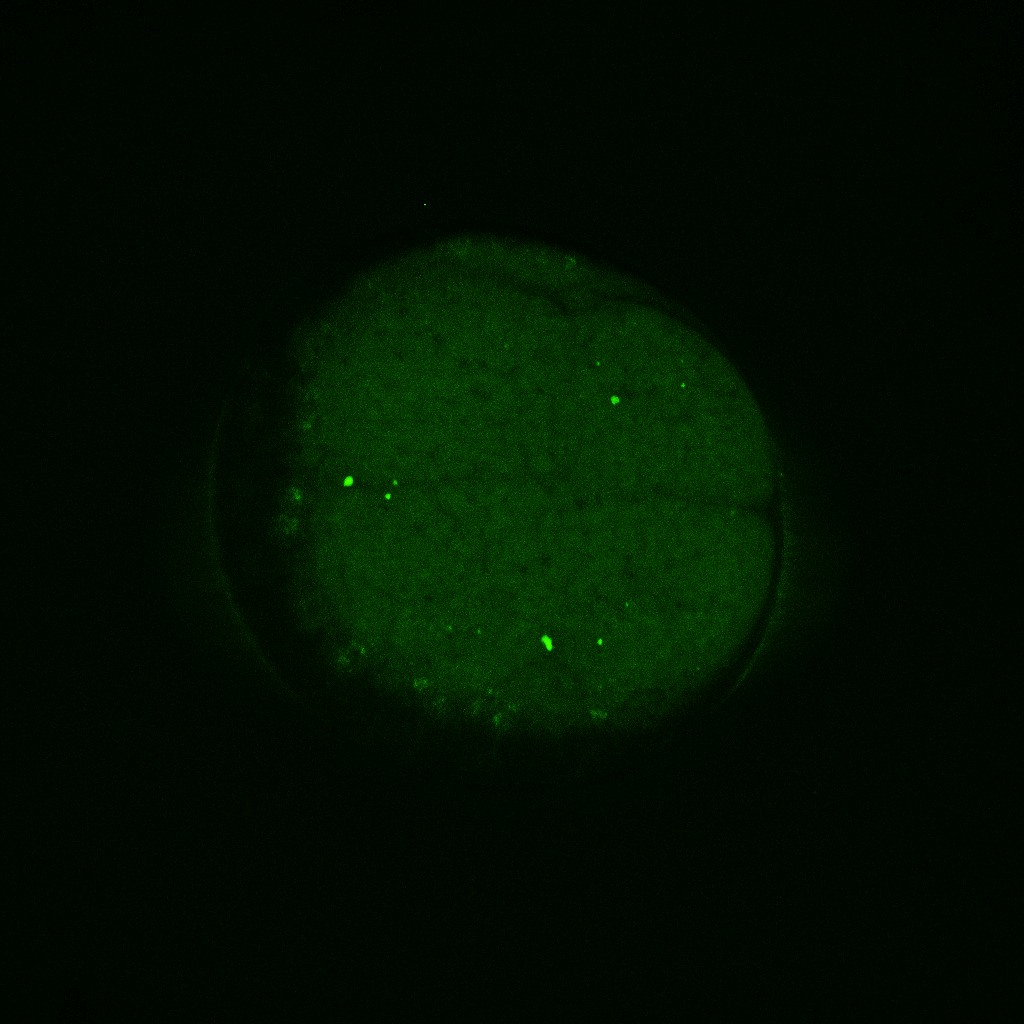

Supplement: Supplementary file 12 — Source data Fig. 1 [file 44318_2025_442_MOESM12_ESM.zip › Figure_1/Figure_1E/1k-cell.tif]

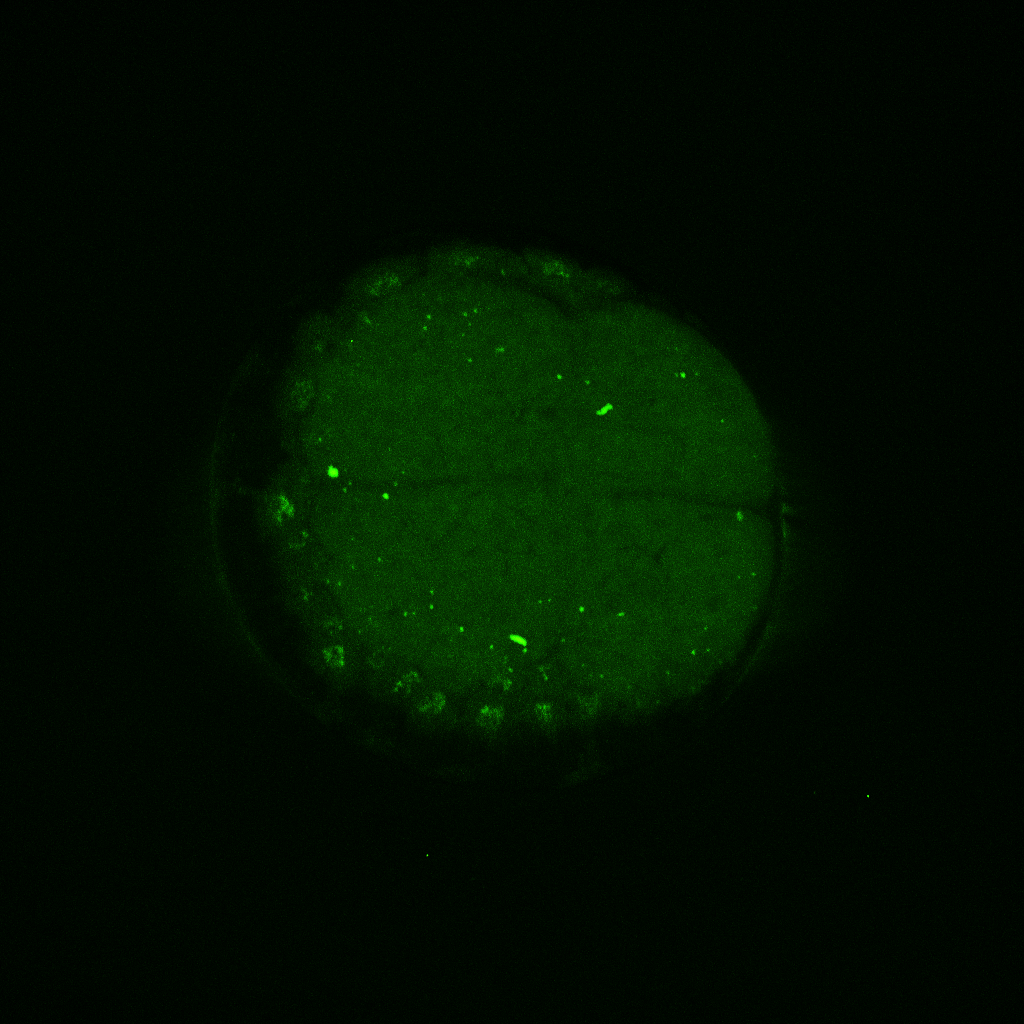

Supplement: Supplementary file 12 — Source data Fig. 1 [file 44318_2025_442_MOESM12_ESM.zip › Figure_1/Figure_1E/256-cell.tif]

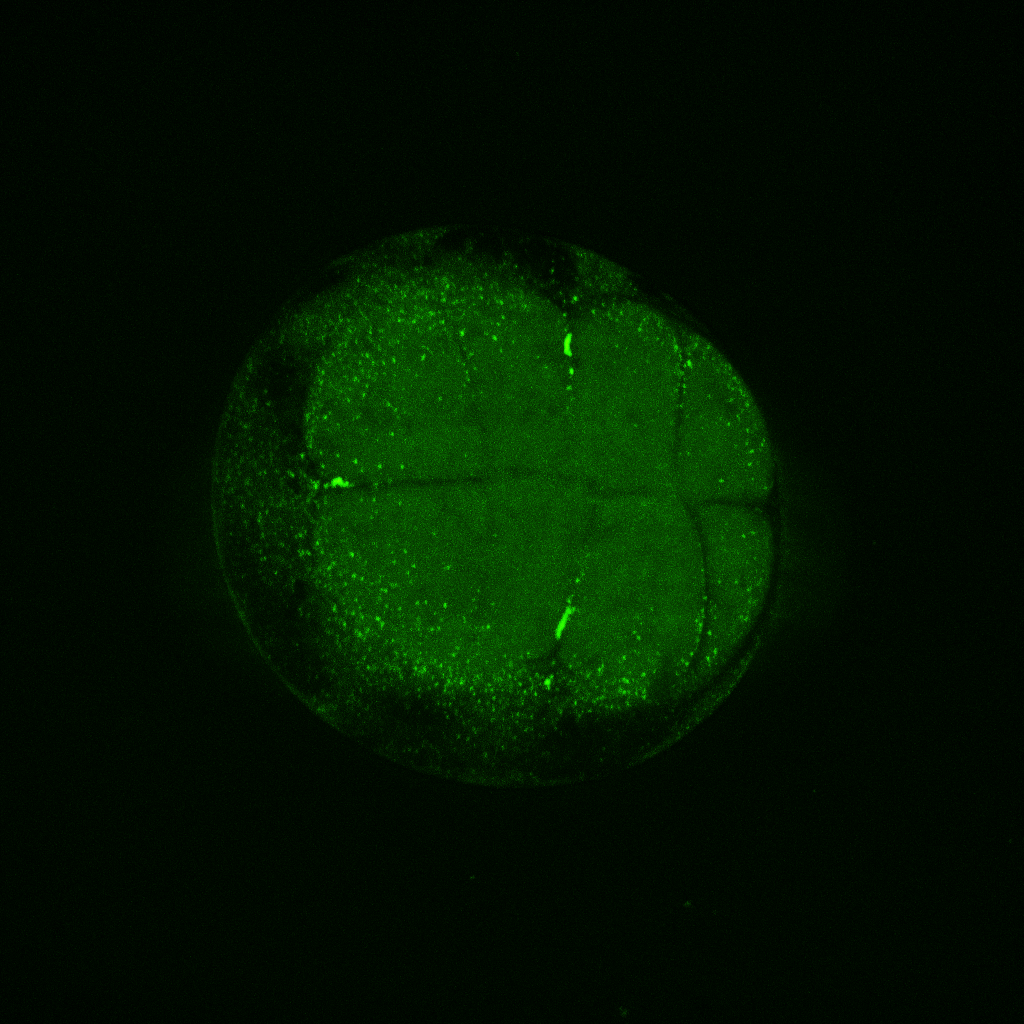

Supplement: Supplementary file 12 — Source data Fig. 1 [file 44318_2025_442_MOESM12_ESM.zip › Figure_1/Figure_1E/8-cell.tif]

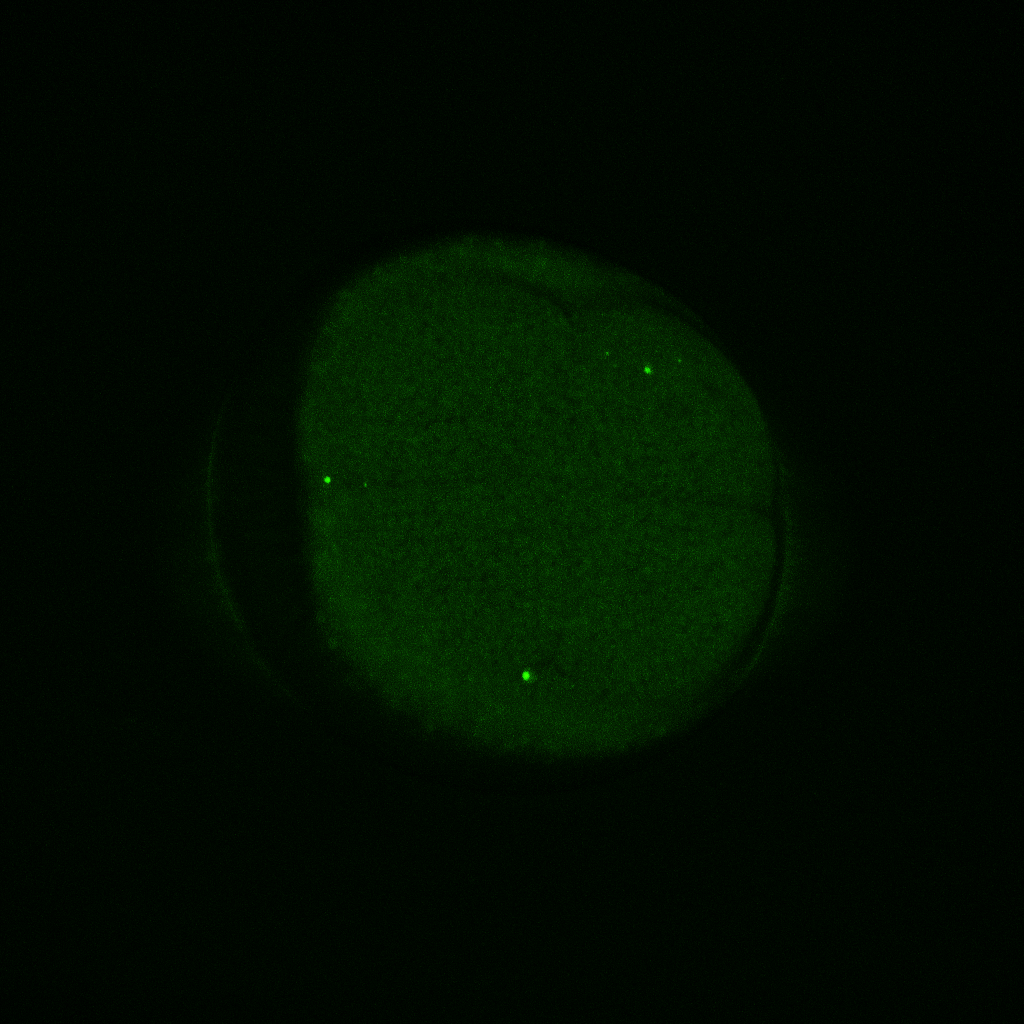

Supplement: Supplementary file 12 — Source data Fig. 1 [file 44318_2025_442_MOESM12_ESM.zip › Figure_1/Figure_1E/sphere.tif]

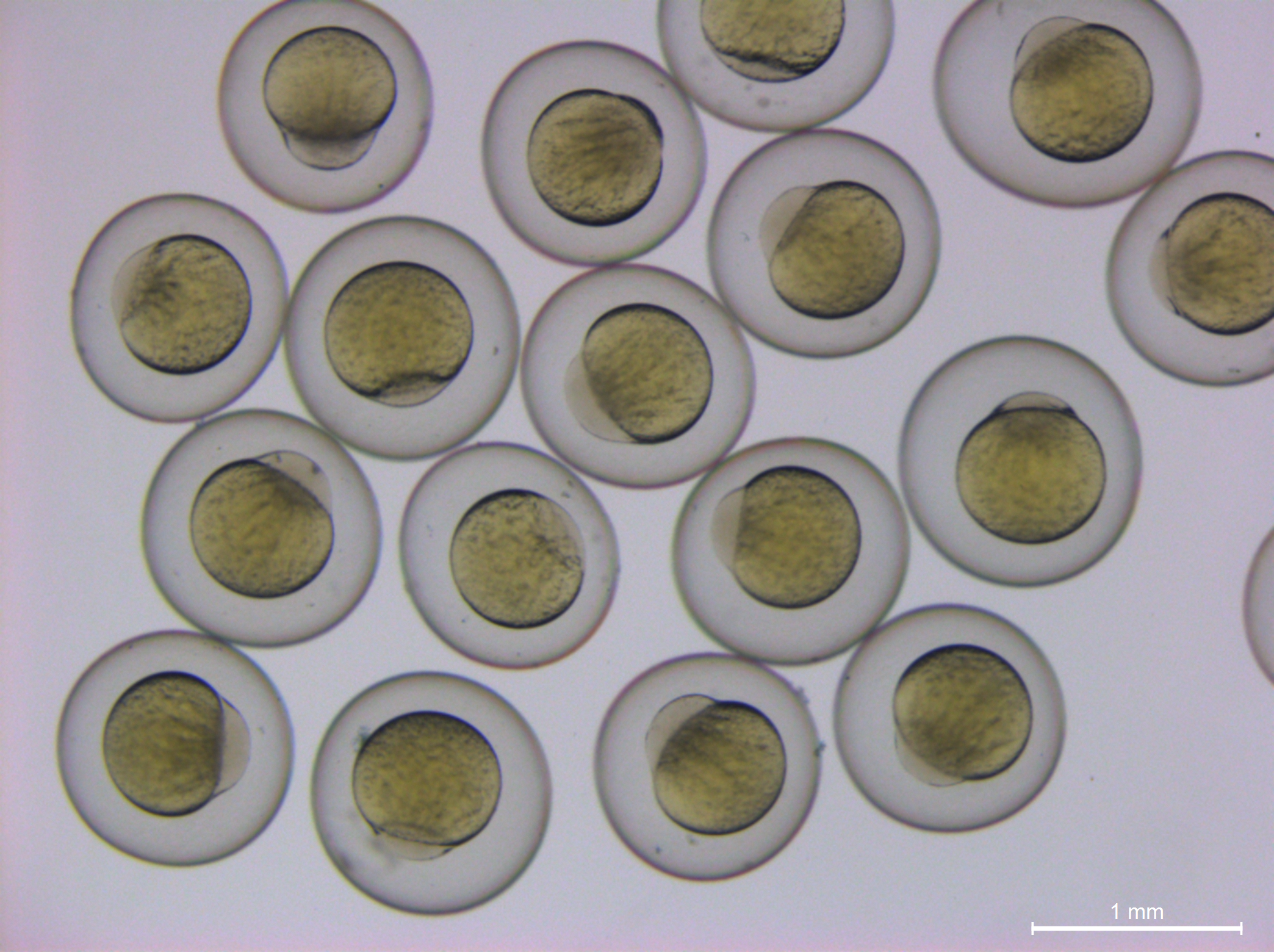

Supplement: Supplementary file 13 — Source data Fig. 2 [file 44318_2025_442_MOESM13_ESM.zip › Figure_2/Figure 2A/BF(bright field).tif]

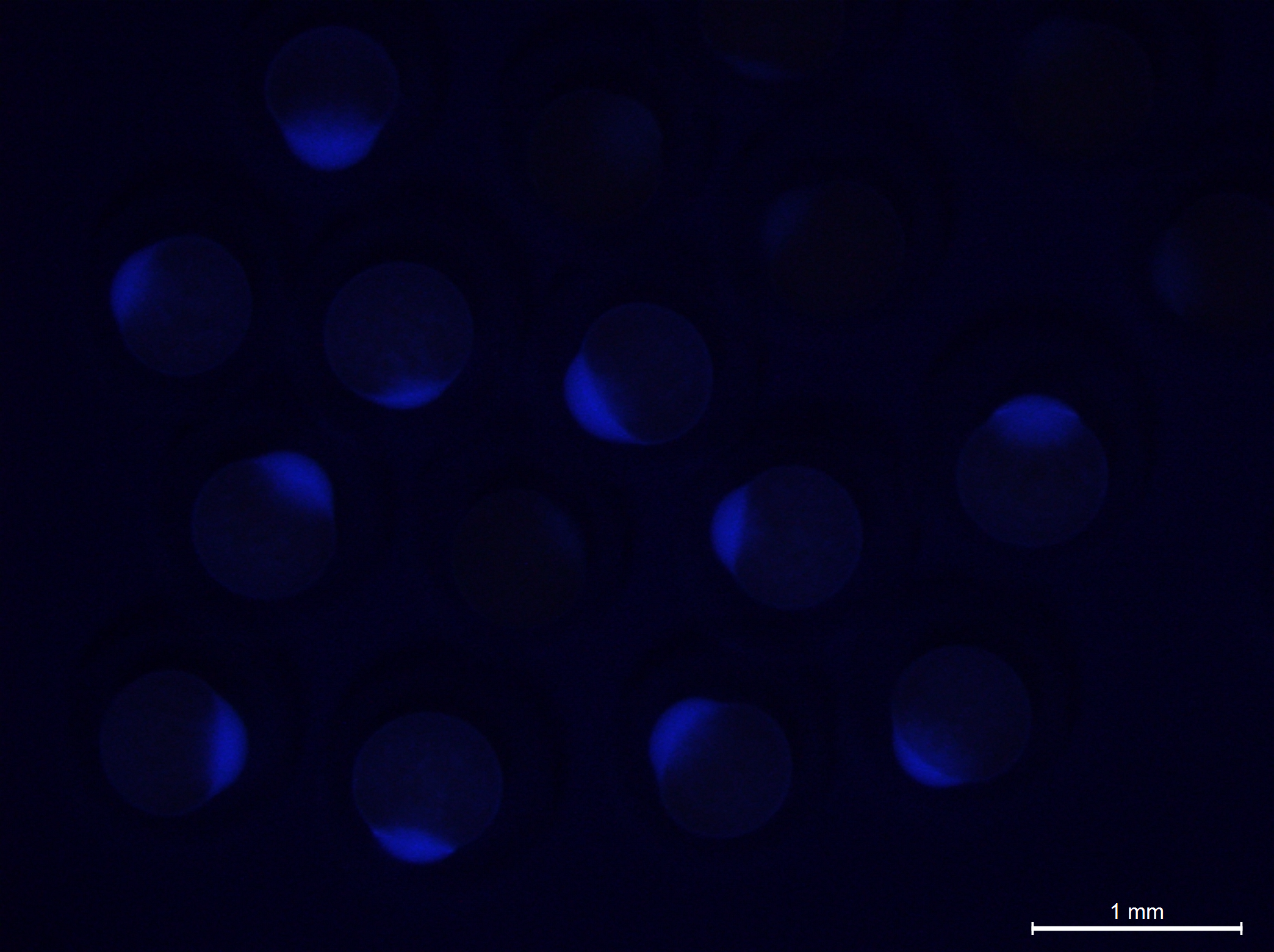

Supplement: Supplementary file 13 — Source data Fig. 2 [file 44318_2025_442_MOESM13_ESM.zip › Figure_2/Figure 2A/BFP.tif]

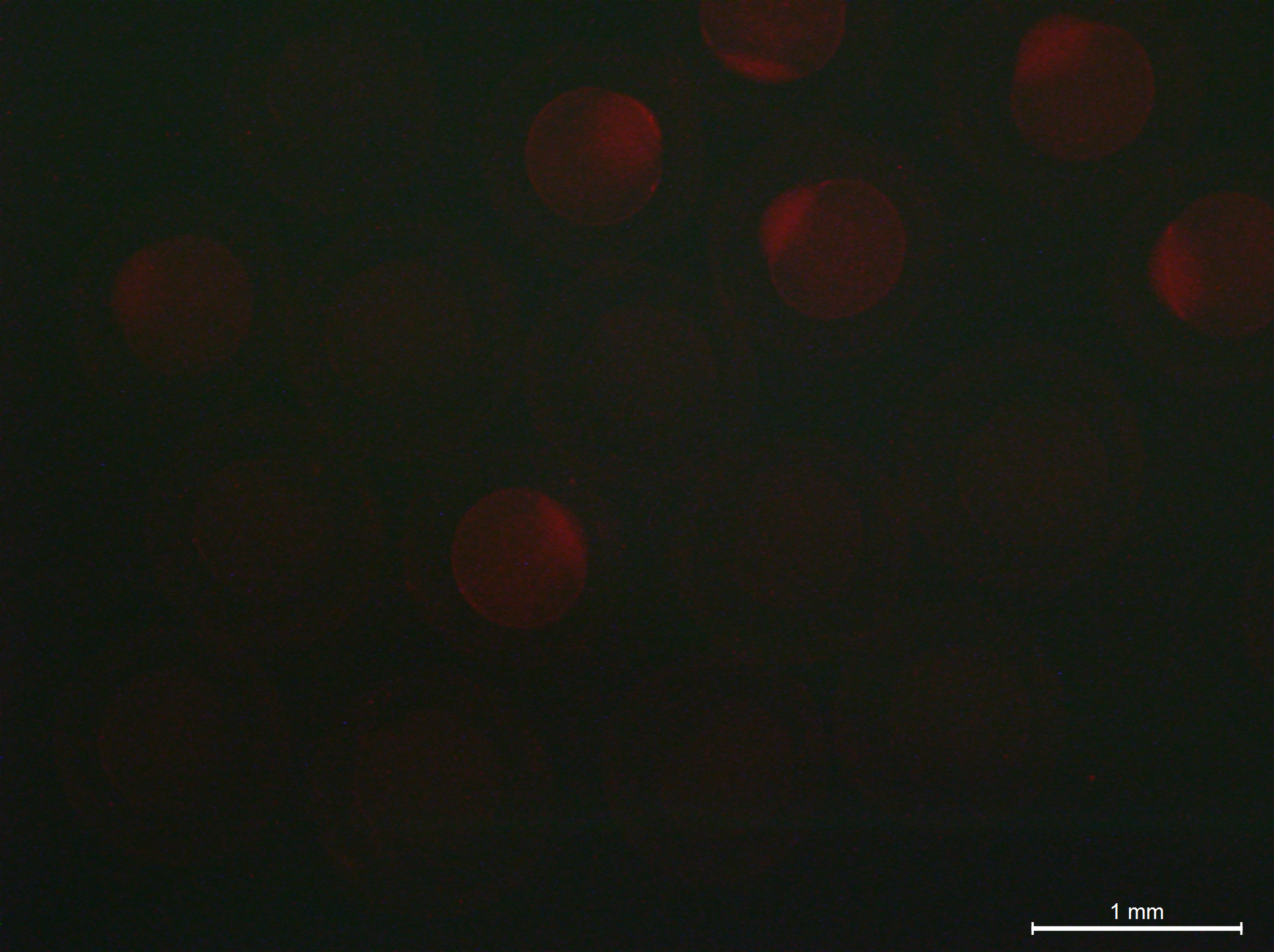

Supplement: Supplementary file 13 — Source data Fig. 2 [file 44318_2025_442_MOESM13_ESM.zip › Figure_2/Figure 2A/Rbm24a-RFP.tif]

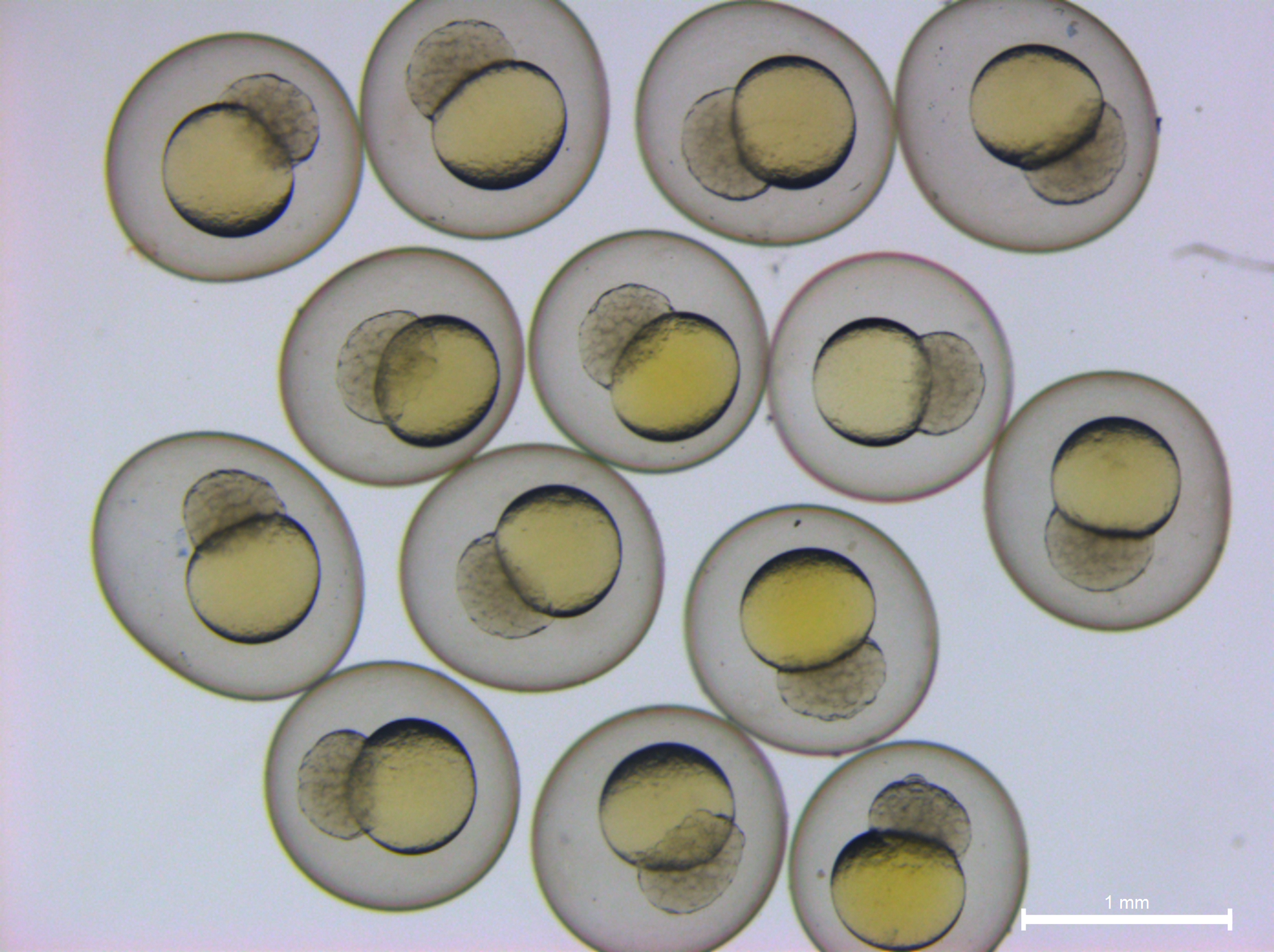

Supplement: Supplementary file 13 — Source data Fig. 2 [file 44318_2025_442_MOESM13_ESM.zip › Figure_2/Figure 2B/wt.tif]

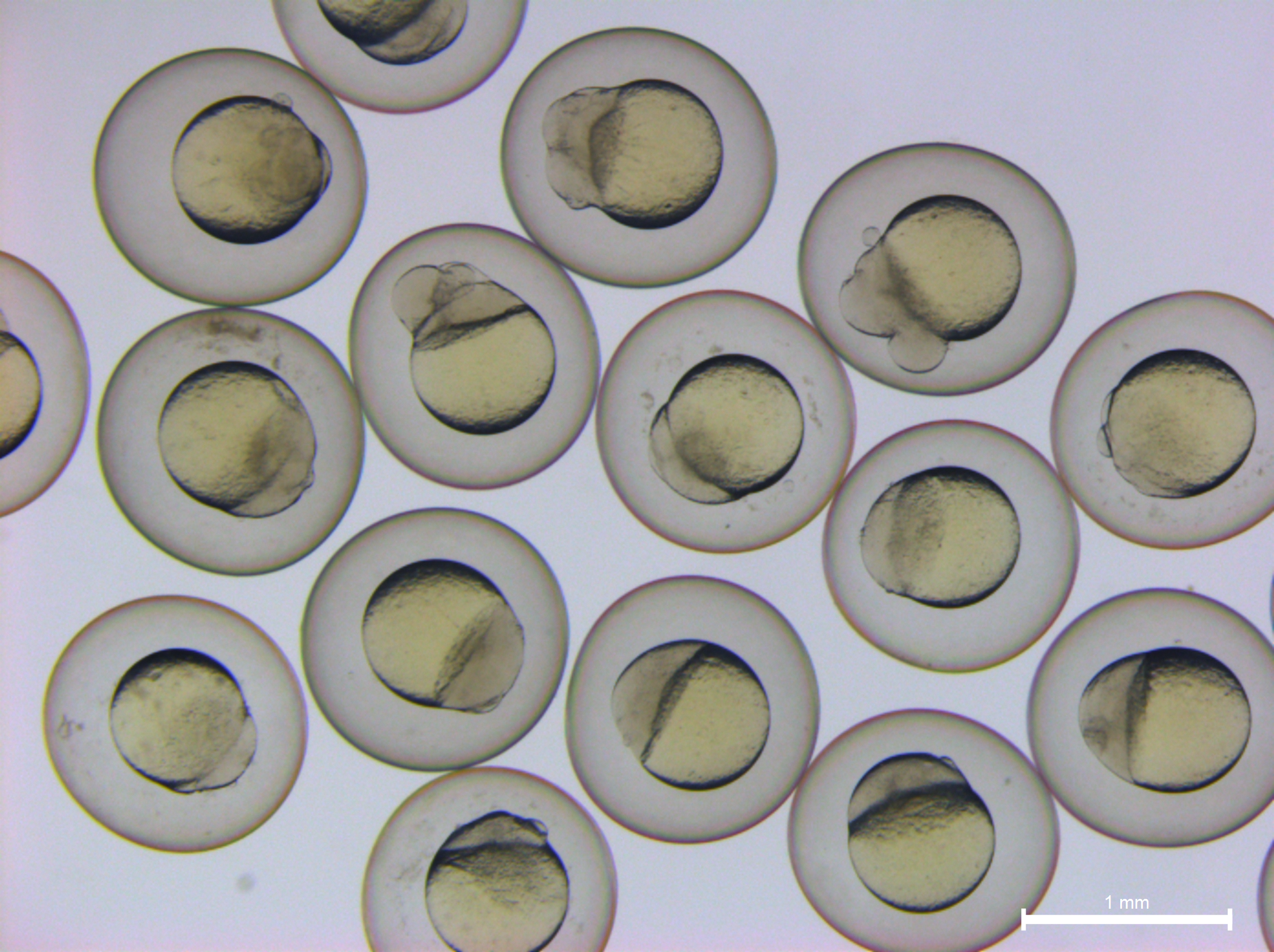

Supplement: Supplementary file 13 — Source data Fig. 2 [file 44318_2025_442_MOESM13_ESM.zip › Figure_2/Figure 2C/Mrbm24a.tif]

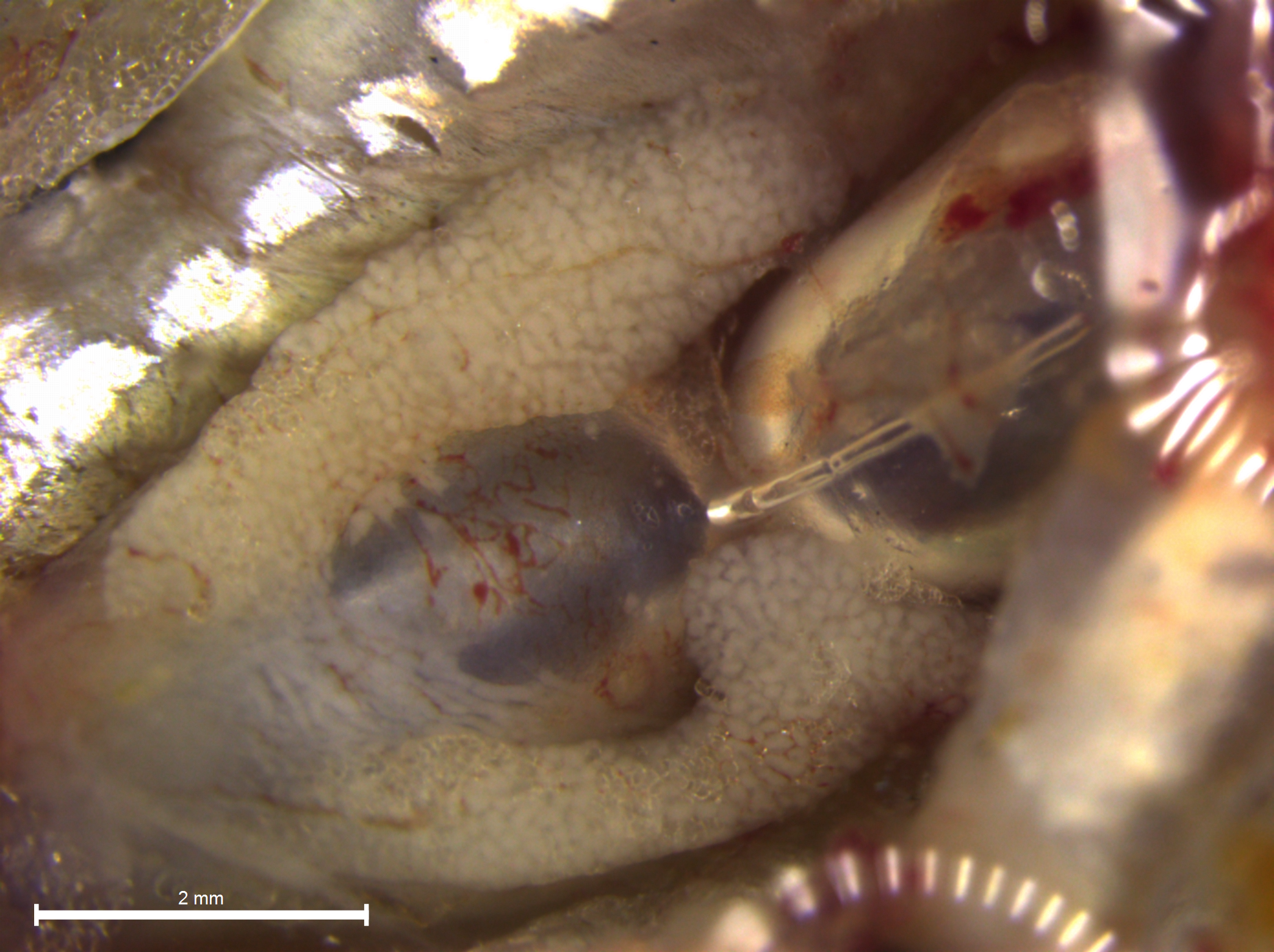

Supplement: Supplementary file 13 — Source data Fig. 2 [file 44318_2025_442_MOESM13_ESM.zip › Figure_2/Figure 2D/WT.tif]

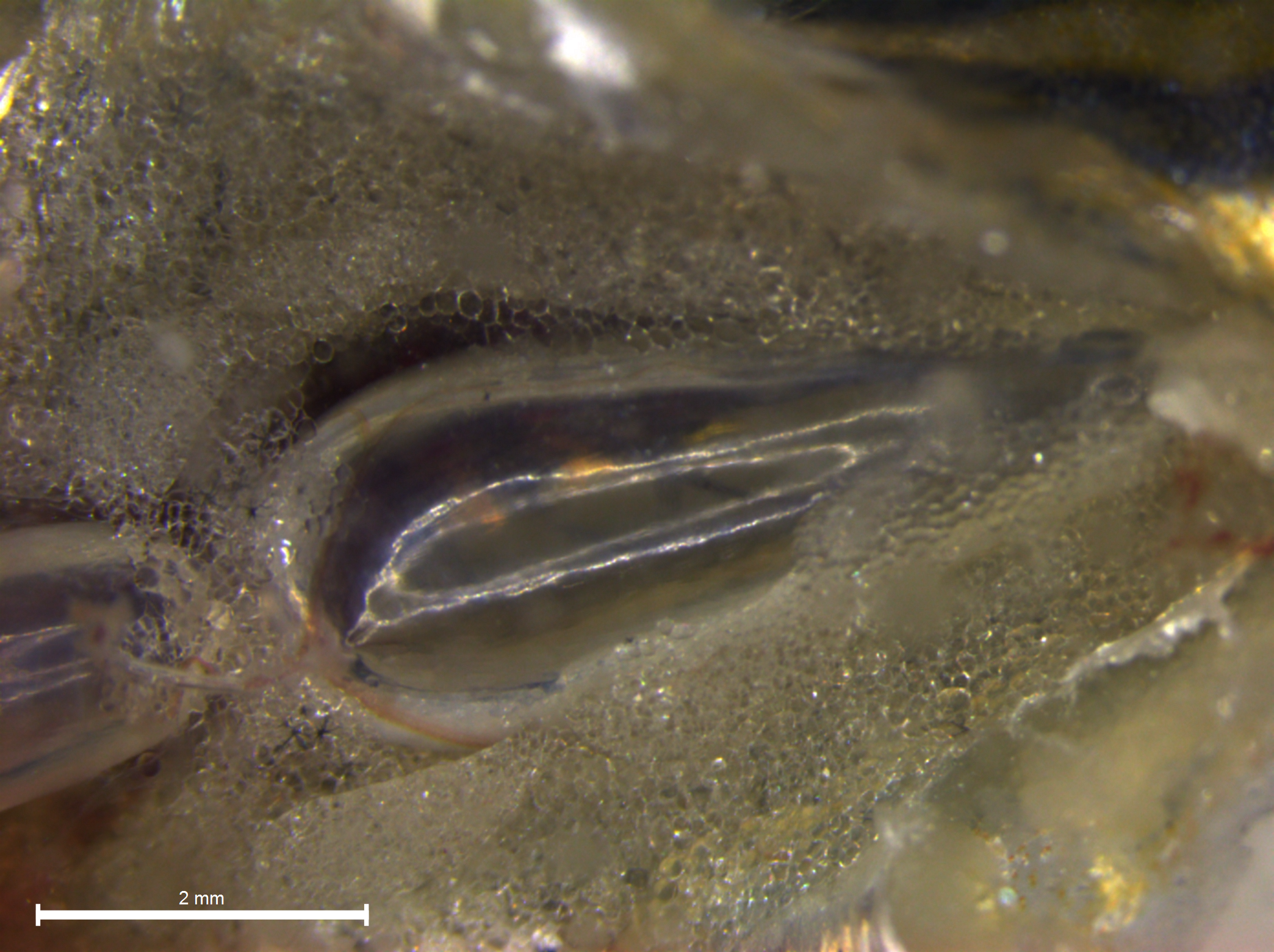

Supplement: Supplementary file 13 — Source data Fig. 2 [file 44318_2025_442_MOESM13_ESM.zip › Figure_2/Figure 2E/M.tif]

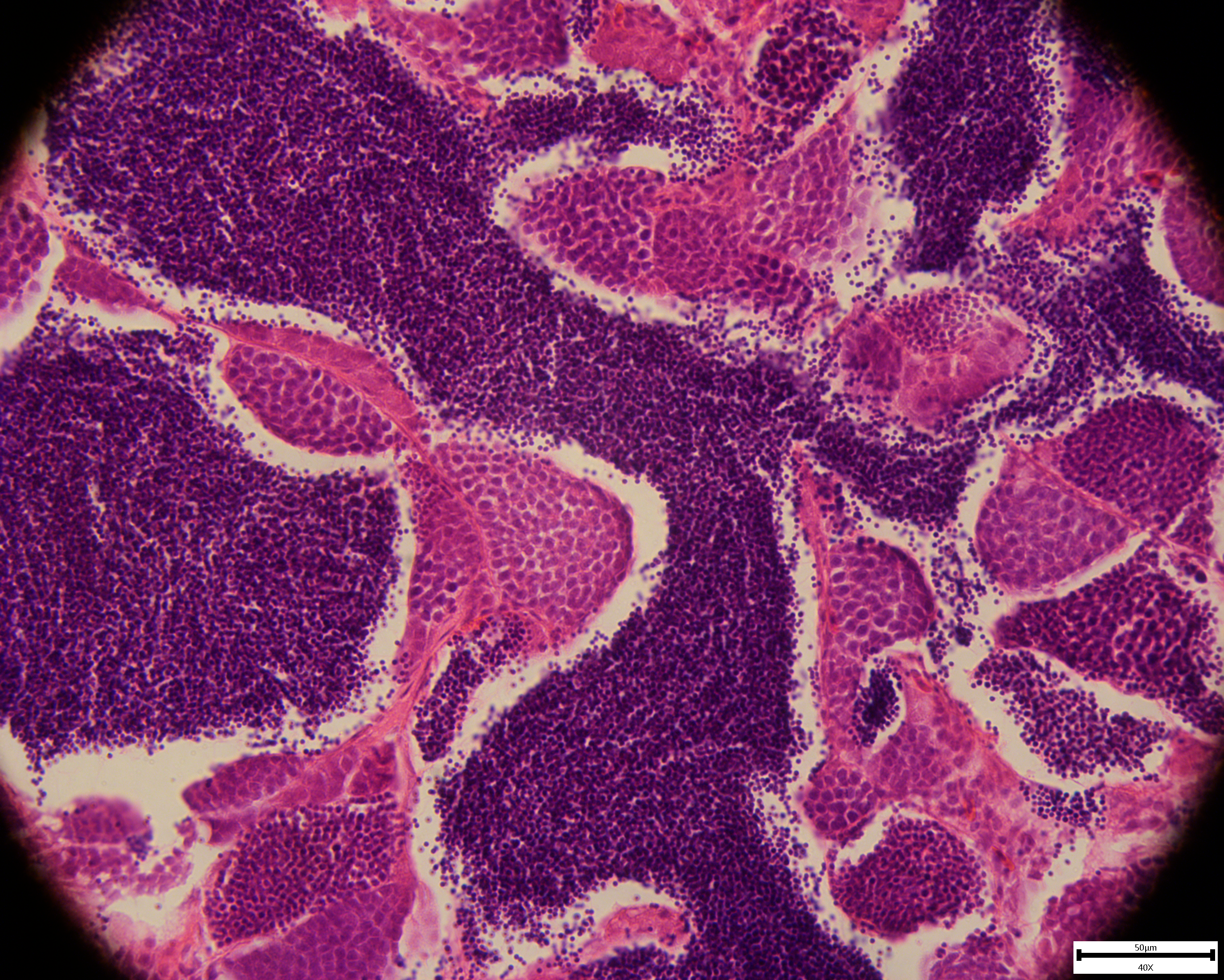

Supplement: Supplementary file 13 — Source data Fig. 2 [file 44318_2025_442_MOESM13_ESM.zip › Figure_2/Figure 2F/wt.tif]

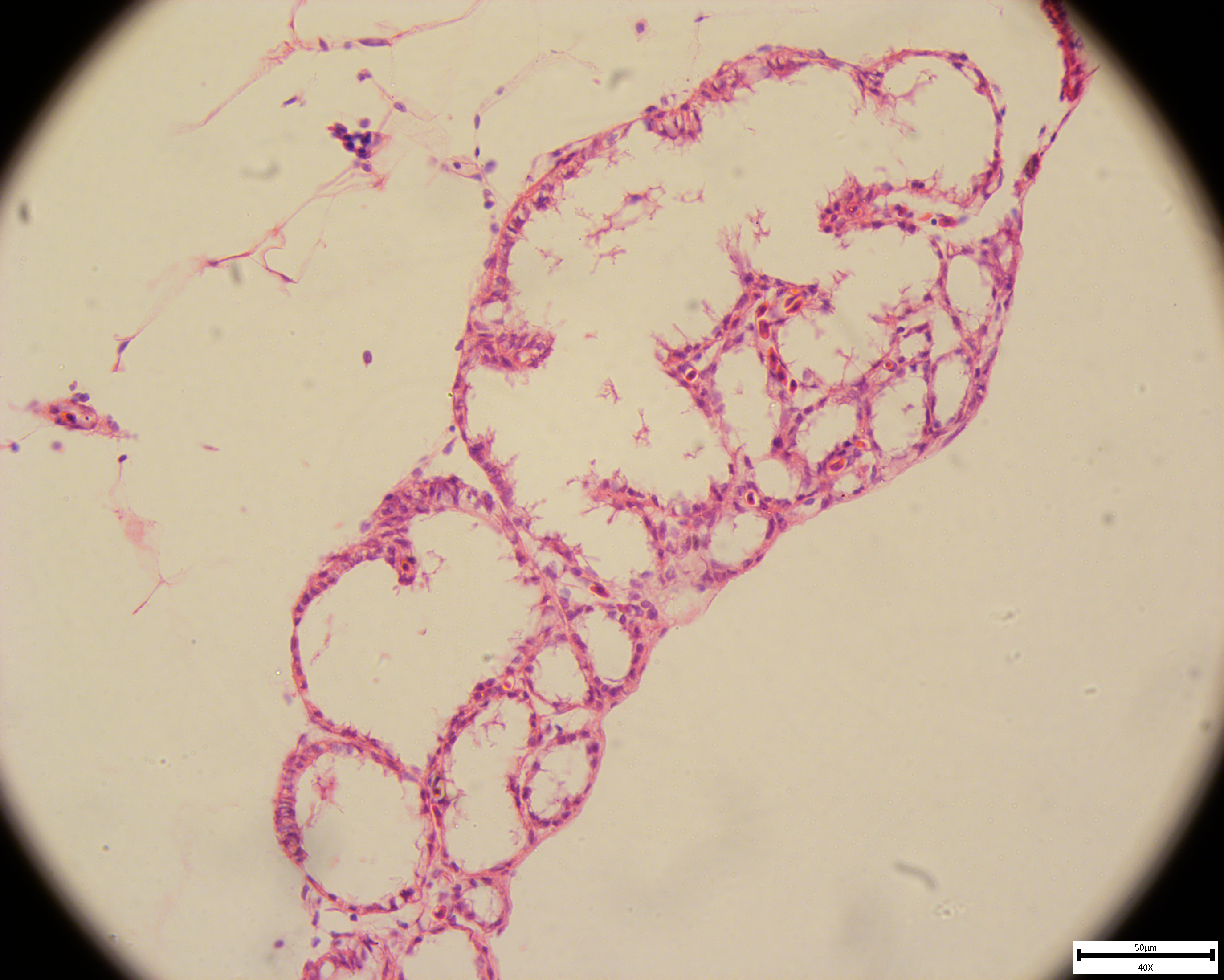

Supplement: Supplementary file 13 — Source data Fig. 2 [file 44318_2025_442_MOESM13_ESM.zip › Figure_2/Figure 2G/Mrbm24a.tif]

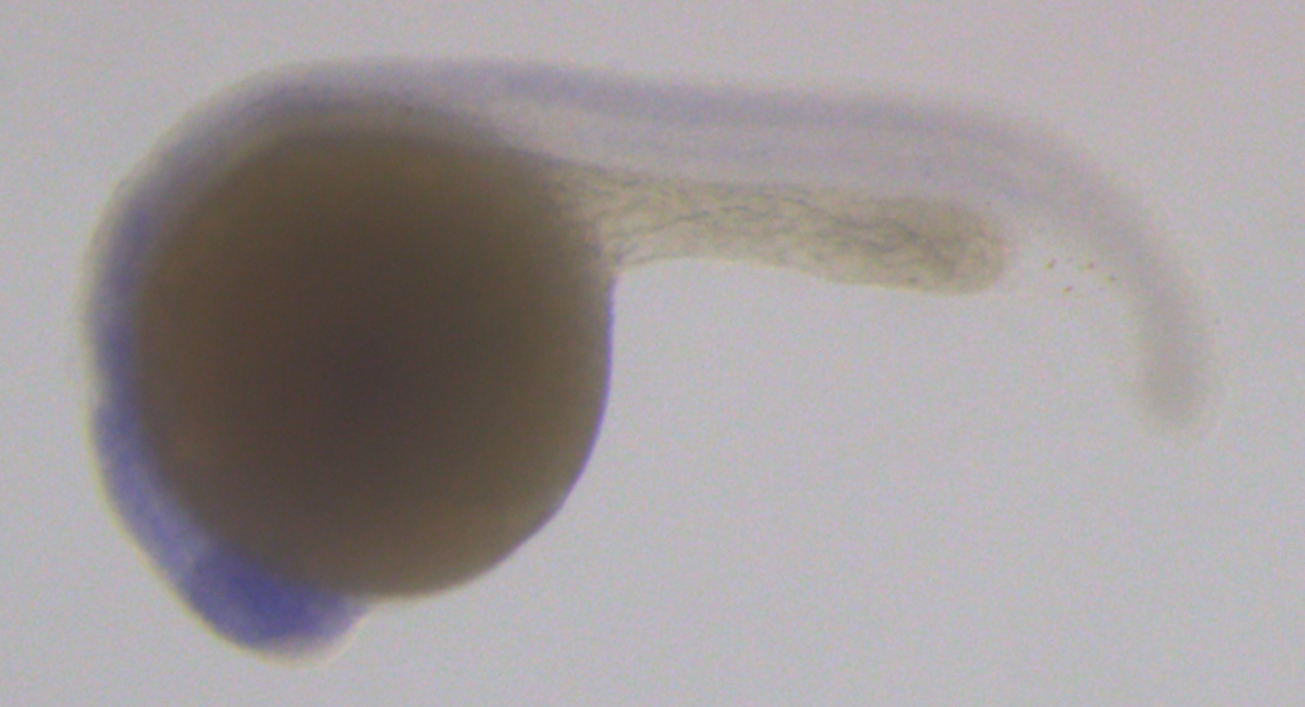

Supplement: Supplementary file 13 — Source data Fig. 2 [file 44318_2025_442_MOESM13_ESM.zip › Figure_2/Figure 2H/Mrbm24a ca15b.tif]

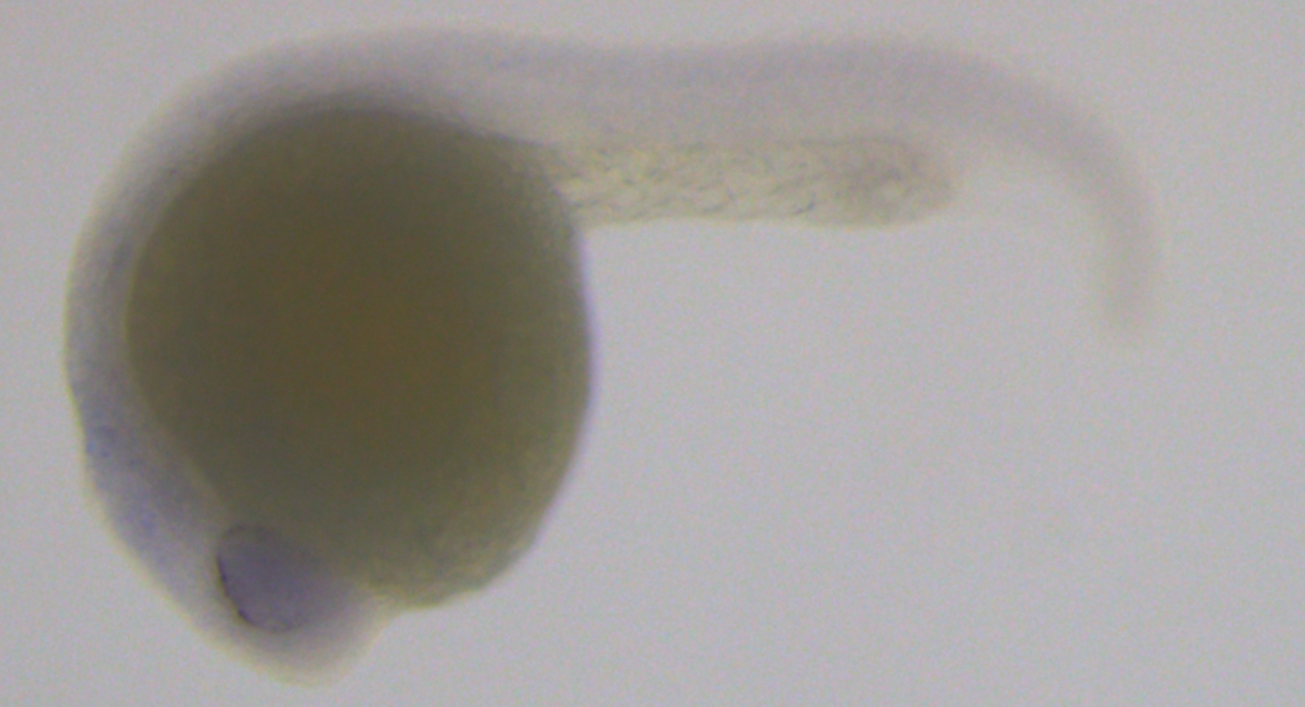

Supplement: Supplementary file 13 — Source data Fig. 2 [file 44318_2025_442_MOESM13_ESM.zip › Figure_2/Figure 2H/Mrbm24a ddx4.tif]

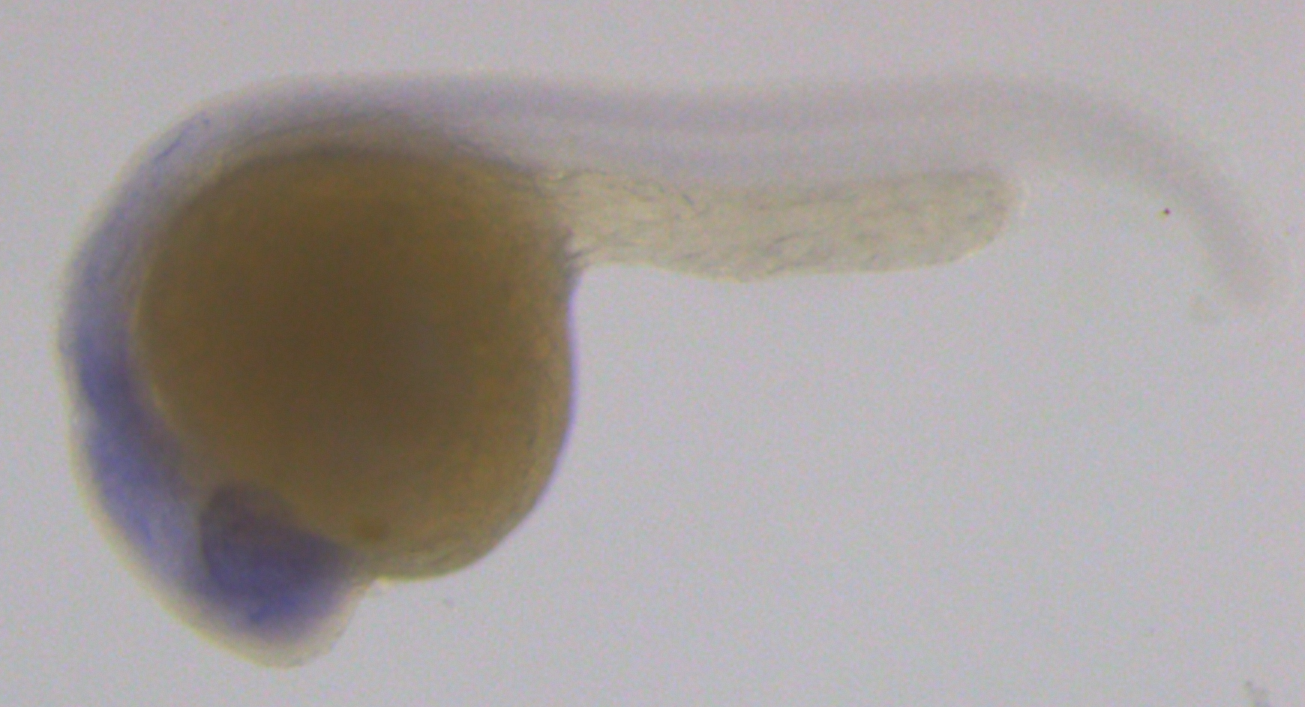

Supplement: Supplementary file 13 — Source data Fig. 2 [file 44318_2025_442_MOESM13_ESM.zip › Figure_2/Figure 2H/Mrbm24a kop.tif]

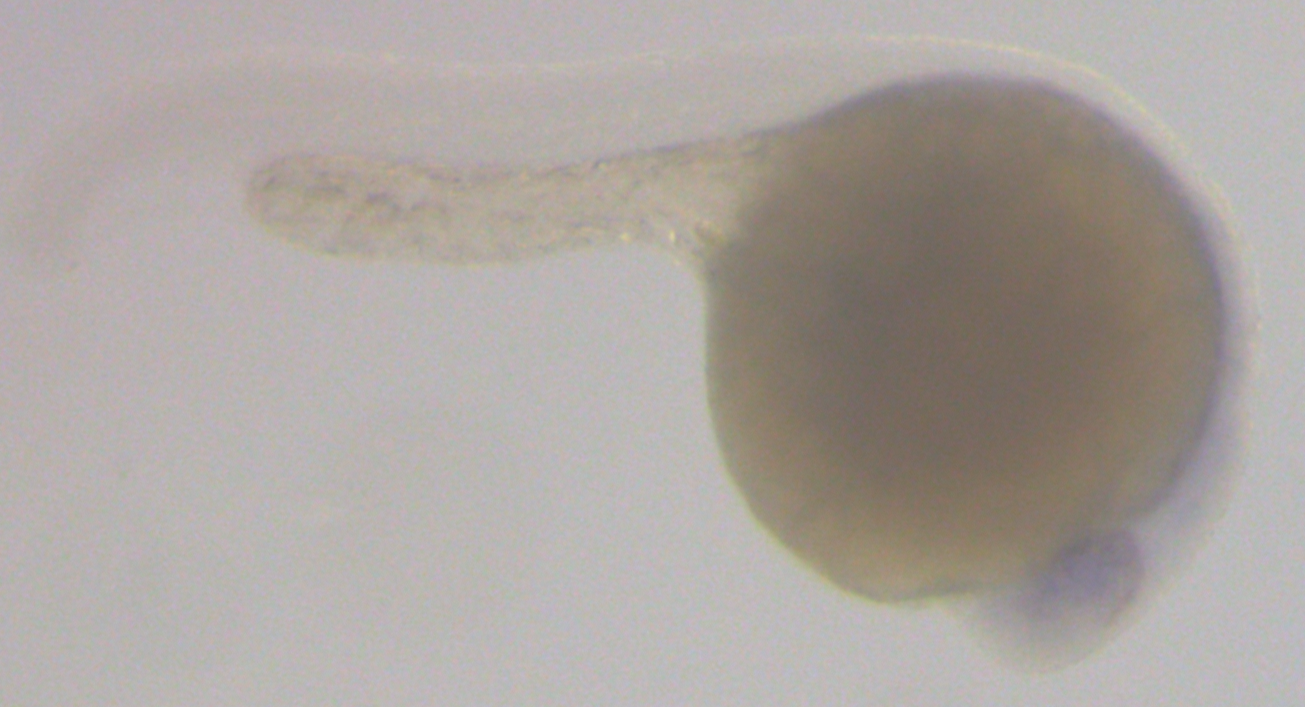

Supplement: Supplementary file 13 — Source data Fig. 2 [file 44318_2025_442_MOESM13_ESM.zip › Figure_2/Figure 2H/Mrbm24a nanos3.tif]

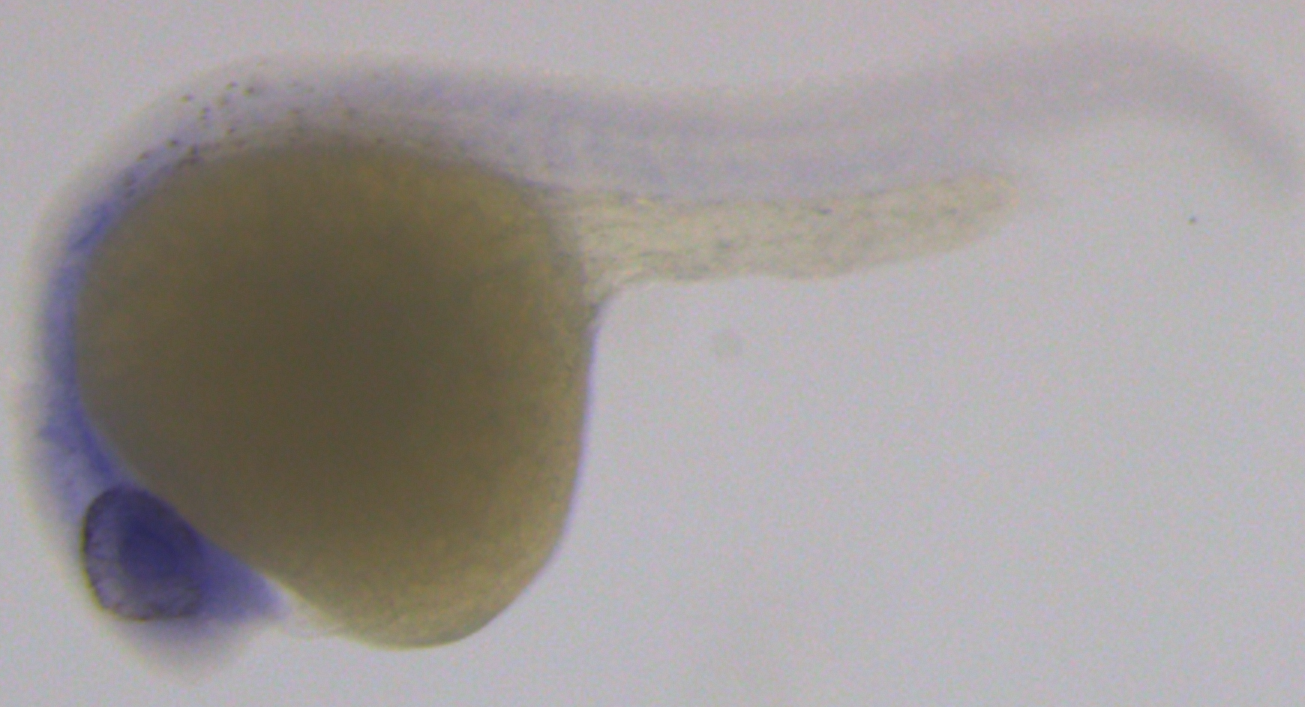

Supplement: Supplementary file 13 — Source data Fig. 2 [file 44318_2025_442_MOESM13_ESM.zip › Figure_2/Figure 2H/Mrbm24a.tif]

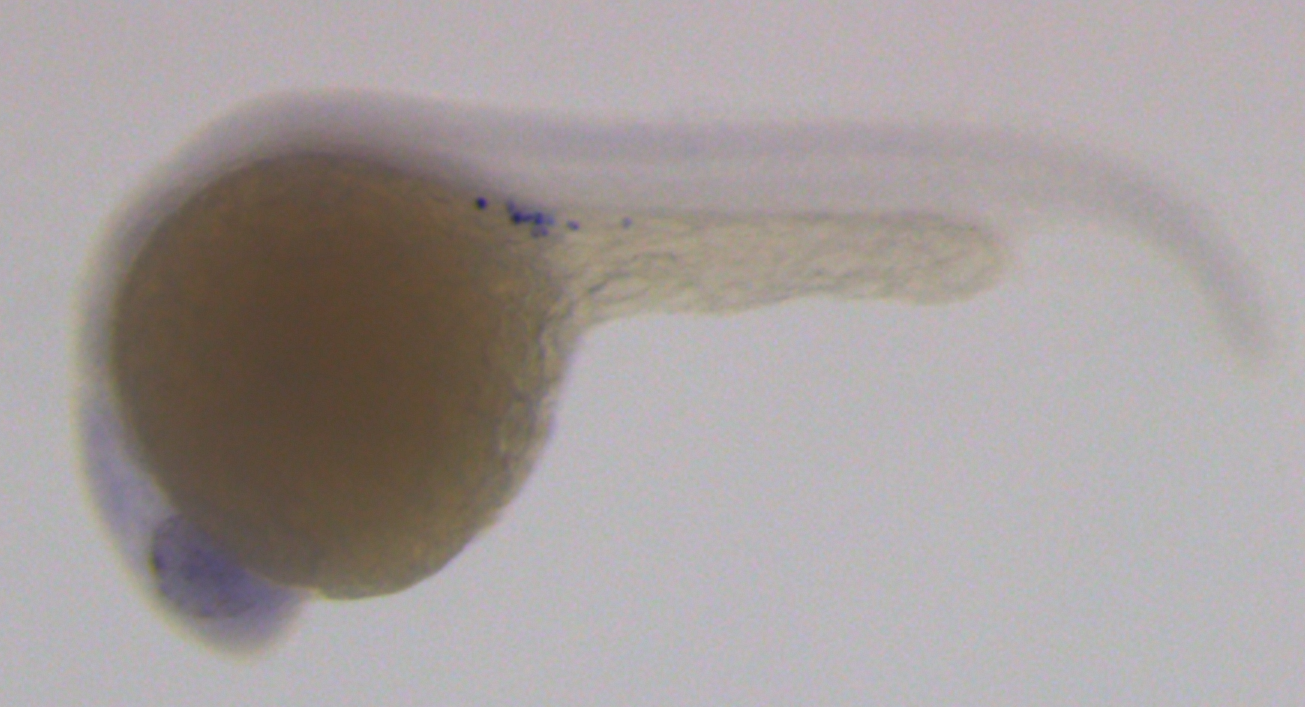

Supplement: Supplementary file 13 — Source data Fig. 2 [file 44318_2025_442_MOESM13_ESM.zip › Figure_2/Figure 2H/sibling ca15b .tif]

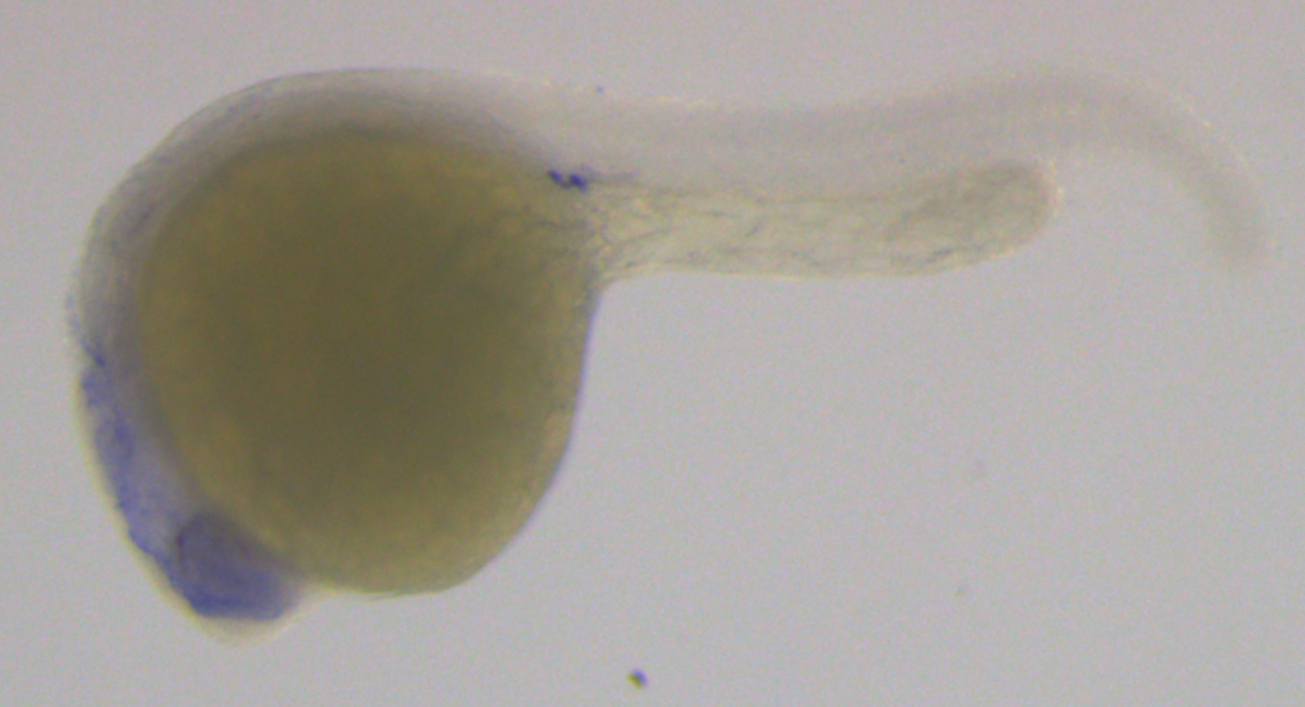

Supplement: Supplementary file 13 — Source data Fig. 2 [file 44318_2025_442_MOESM13_ESM.zip › Figure_2/Figure 2H/sibling ddx4.tif]

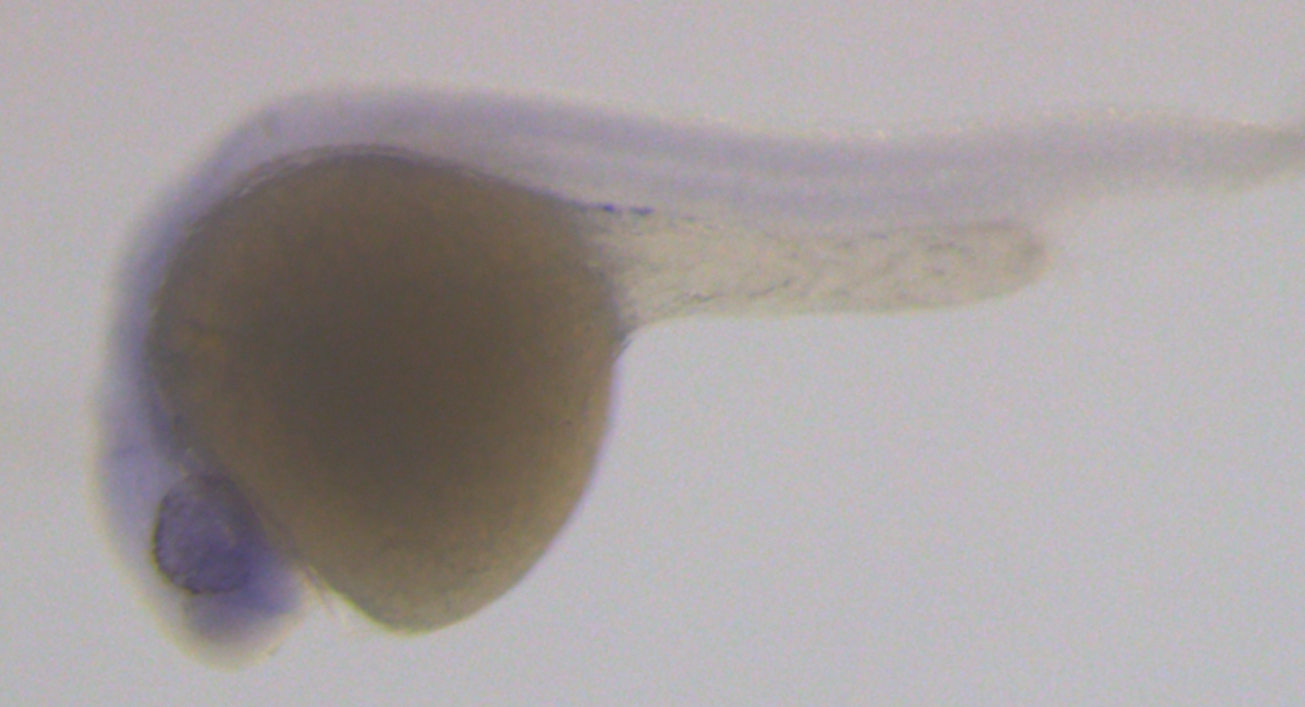

Supplement: Supplementary file 13 — Source data Fig. 2 [file 44318_2025_442_MOESM13_ESM.zip › Figure_2/Figure 2H/sibling kop .tif]

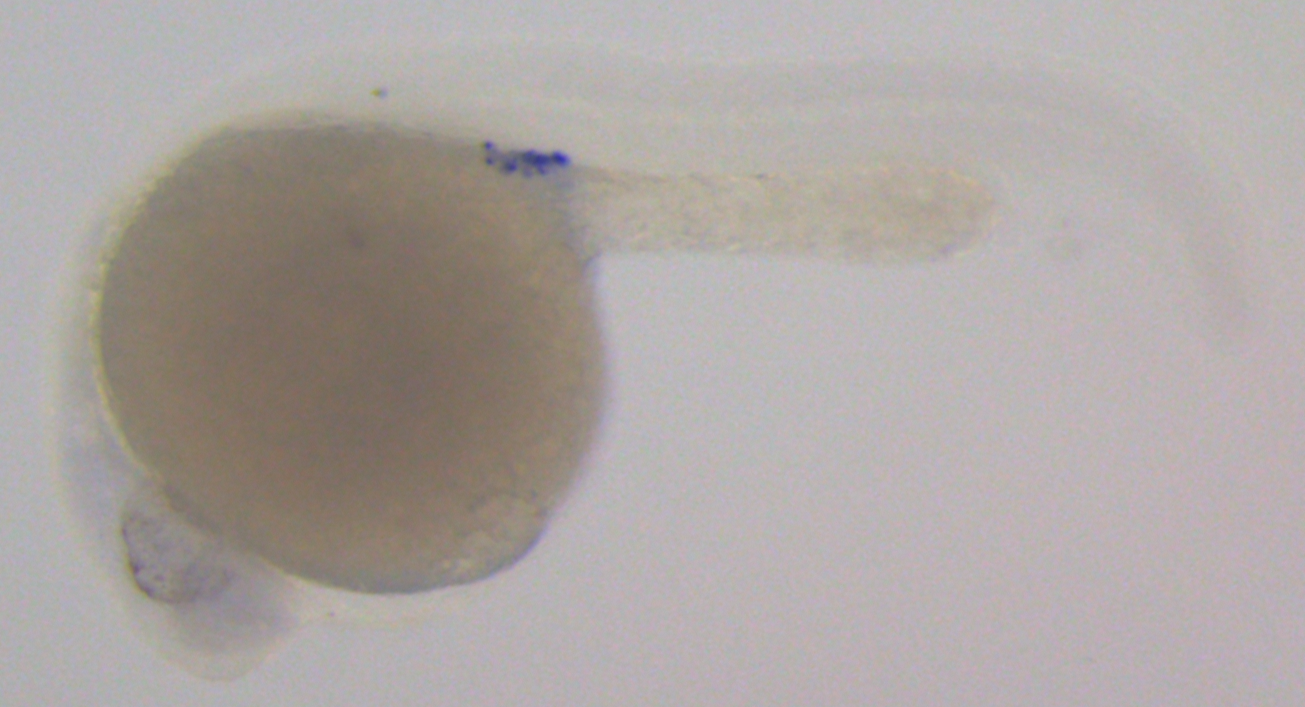

Supplement: Supplementary file 13 — Source data Fig. 2 [file 44318_2025_442_MOESM13_ESM.zip › Figure_2/Figure 2H/sibling nanos3.tif]

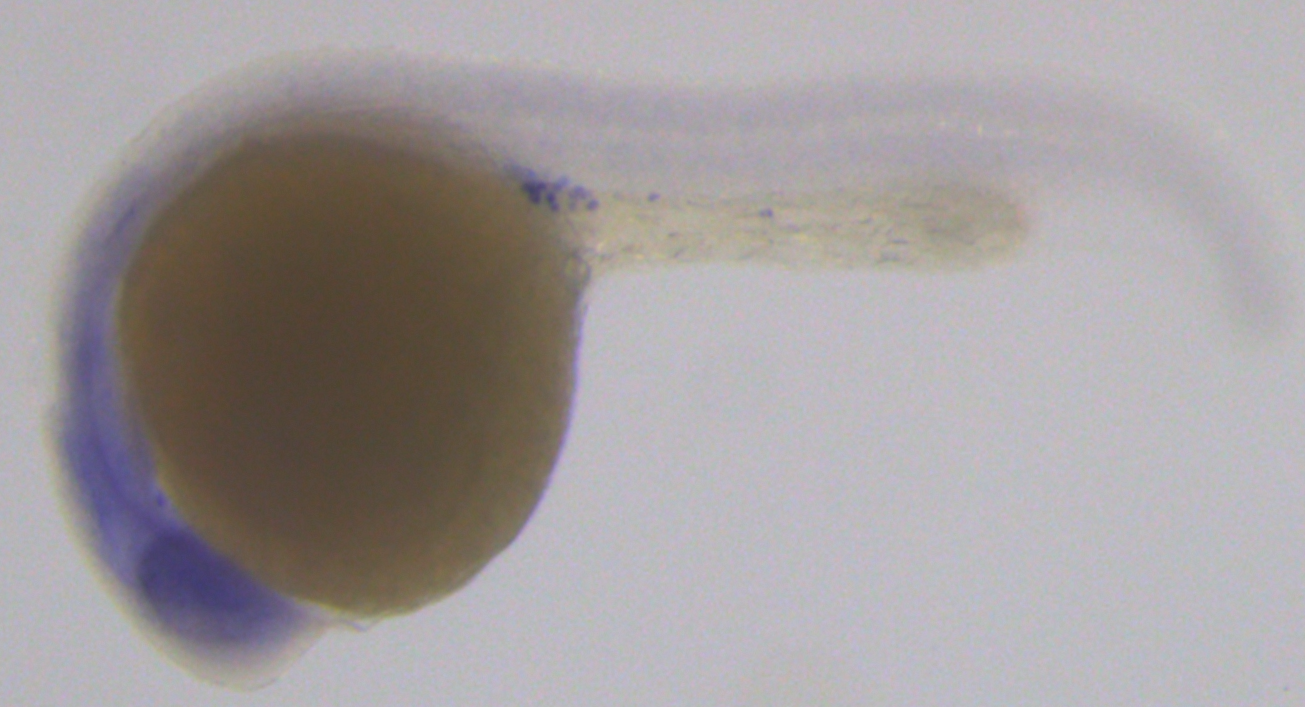

Supplement: Supplementary file 13 — Source data Fig. 2 [file 44318_2025_442_MOESM13_ESM.zip › Figure_2/Figure 2H/sibling tdrd7a.tif]

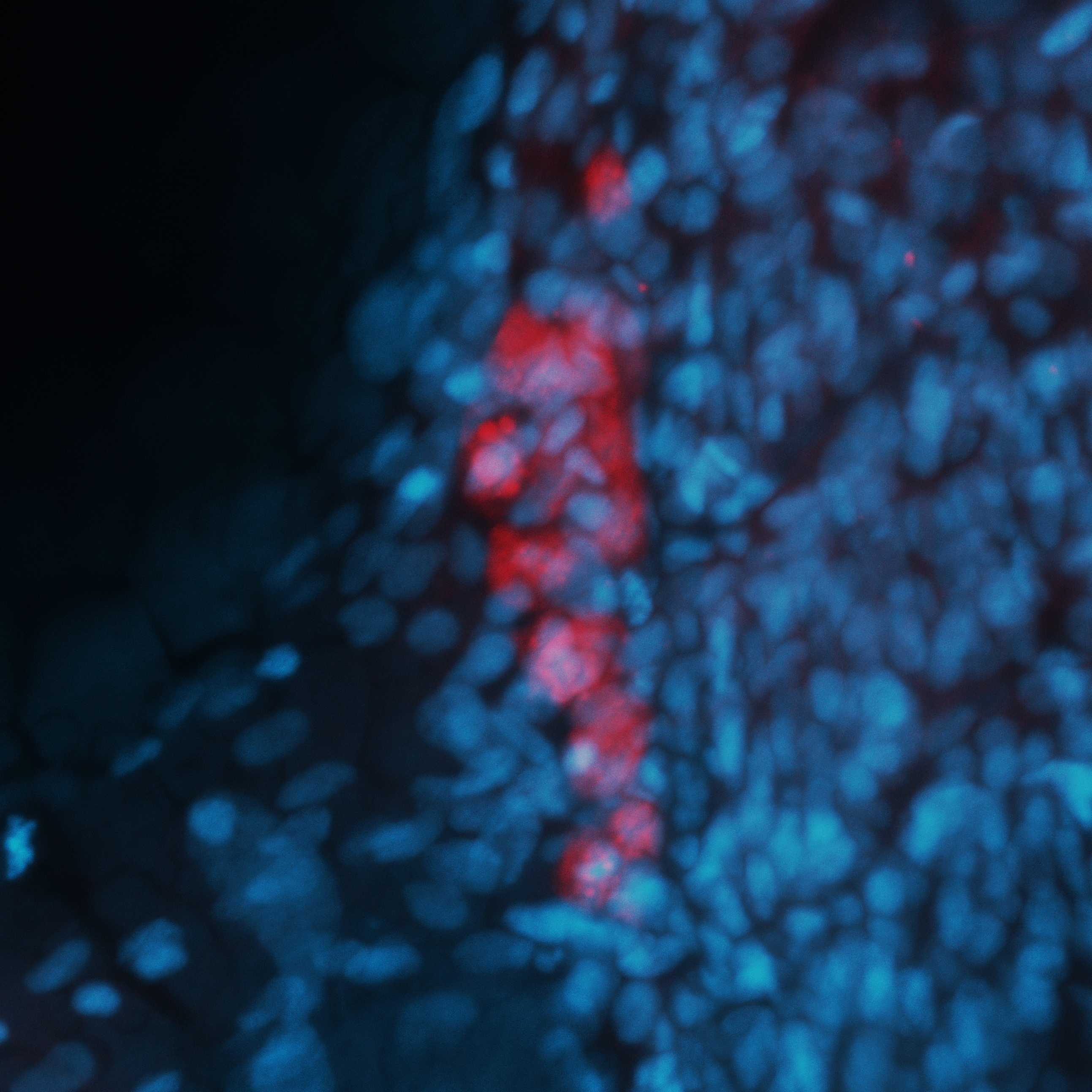

Supplement: Supplementary file 13 — Source data Fig. 2 [file 44318_2025_442_MOESM13_ESM.zip › Figure_2/Figure 2I/ddx4 merge.tif]

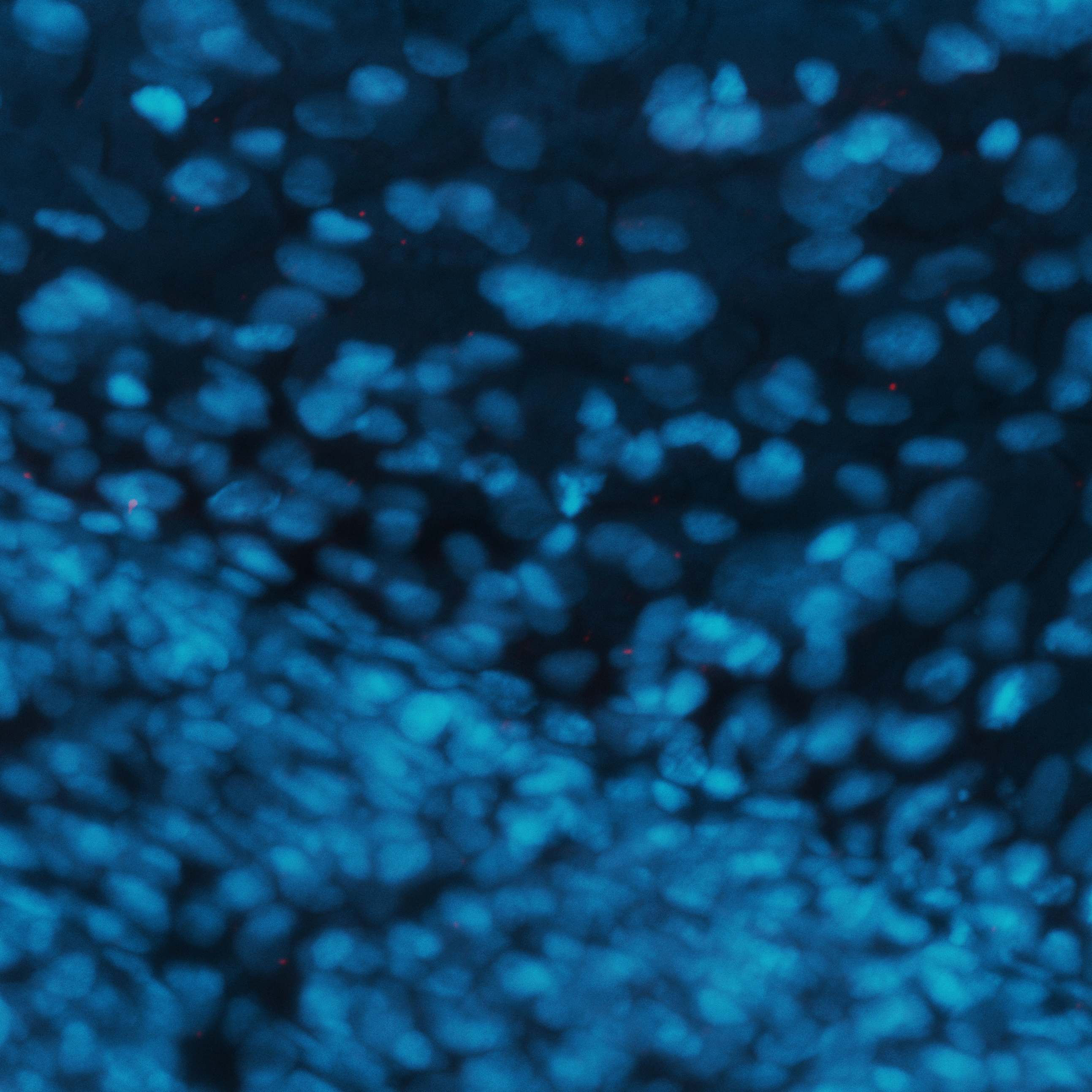

Supplement: Supplementary file 13 — Source data Fig. 2 [file 44318_2025_442_MOESM13_ESM.zip › Figure_2/Figure 2J/Hochest.tif]

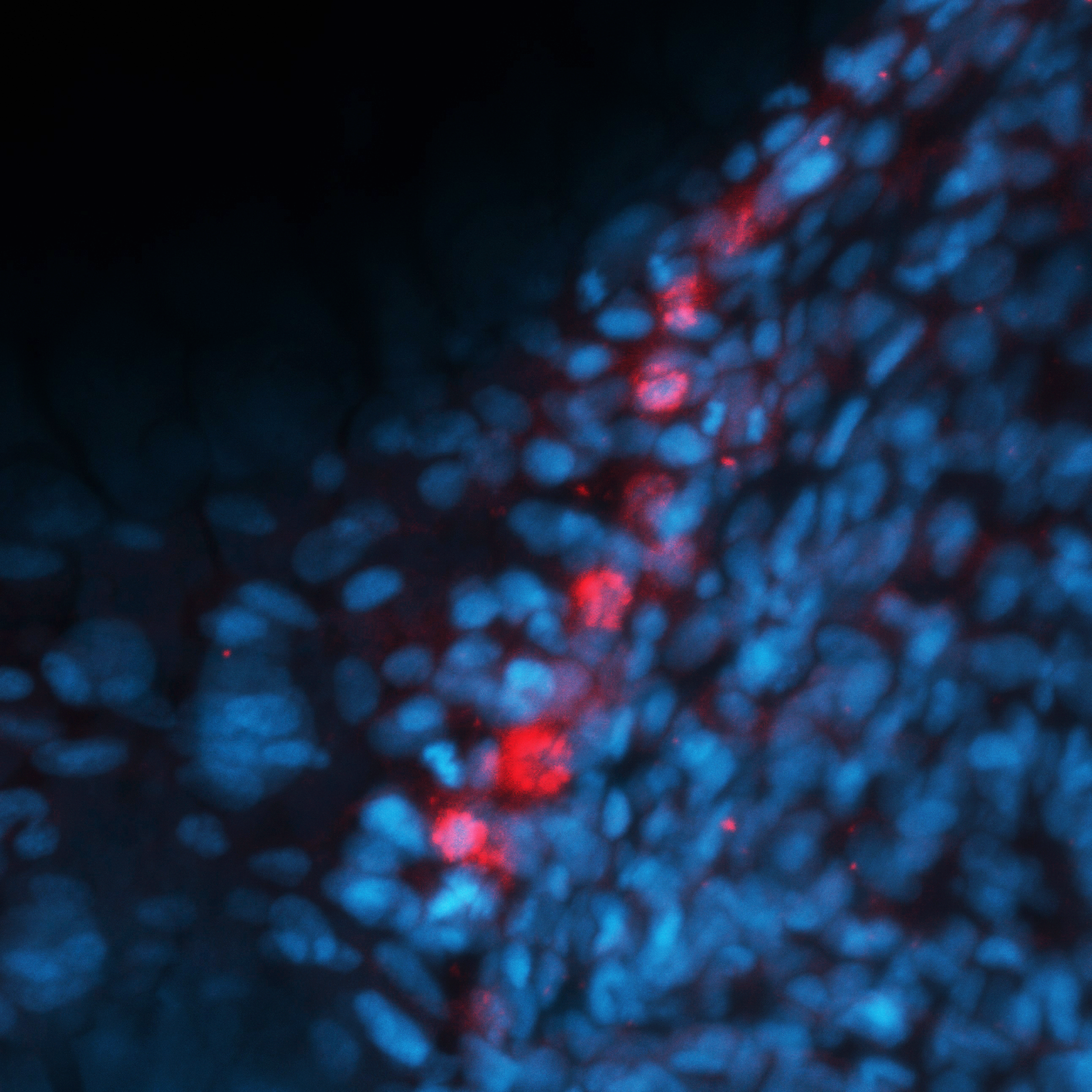

Supplement: Supplementary file 13 — Source data Fig. 2 [file 44318_2025_442_MOESM13_ESM.zip › Figure_2/Figure 2K/Piwil1.tif]

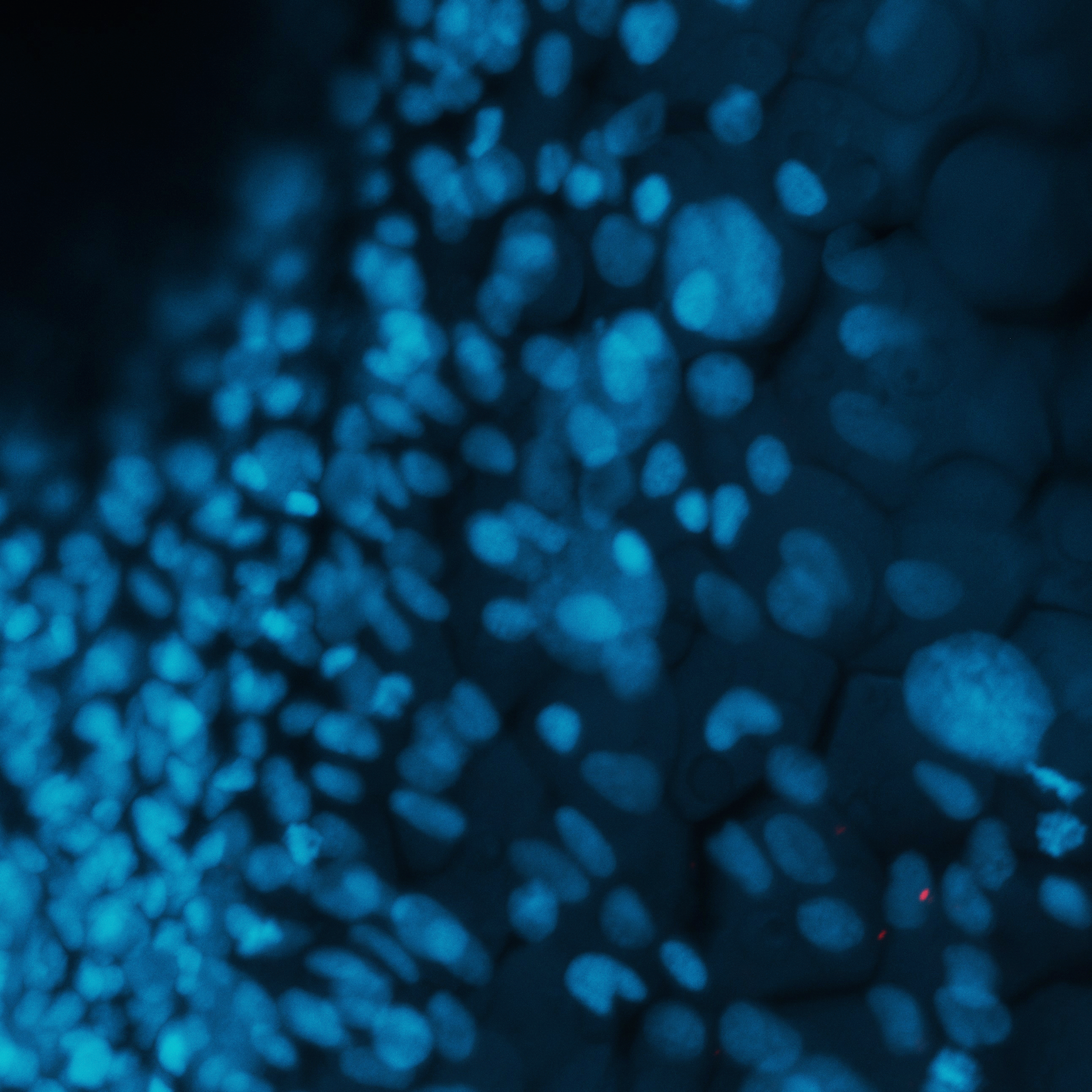

Supplement: Supplementary file 13 — Source data Fig. 2 [file 44318_2025_442_MOESM13_ESM.zip › Figure_2/Figure 2L/Hochest.tif]

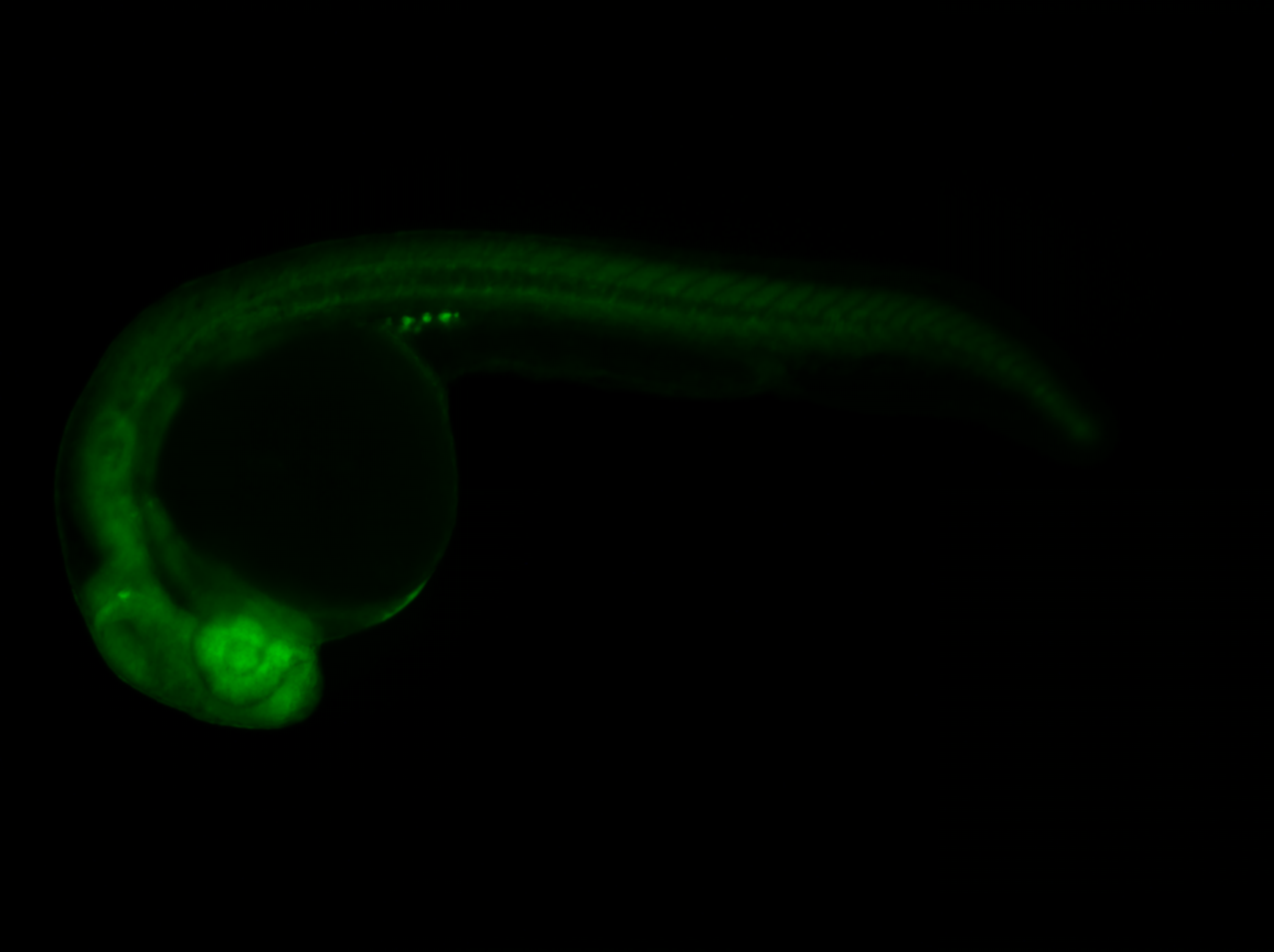

Supplement: Supplementary file 13 — Source data Fig. 2 [file 44318_2025_442_MOESM13_ESM.zip › Figure_2/Figure 2M/WT inj.tif]

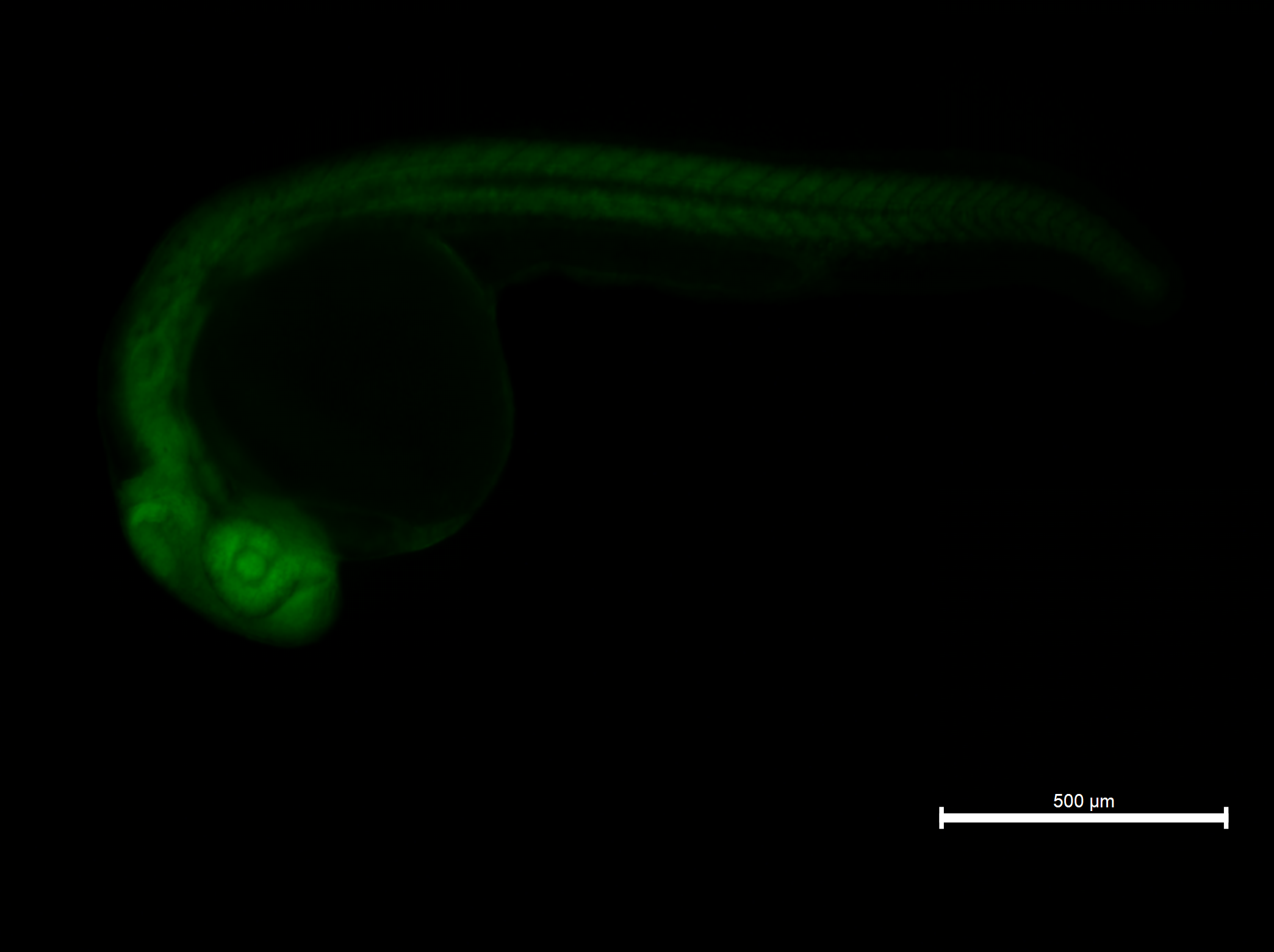

Supplement: Supplementary file 13 — Source data Fig. 2 [file 44318_2025_442_MOESM13_ESM.zip › Figure_2/Figure 2N/Mrbm24a inj.tif]

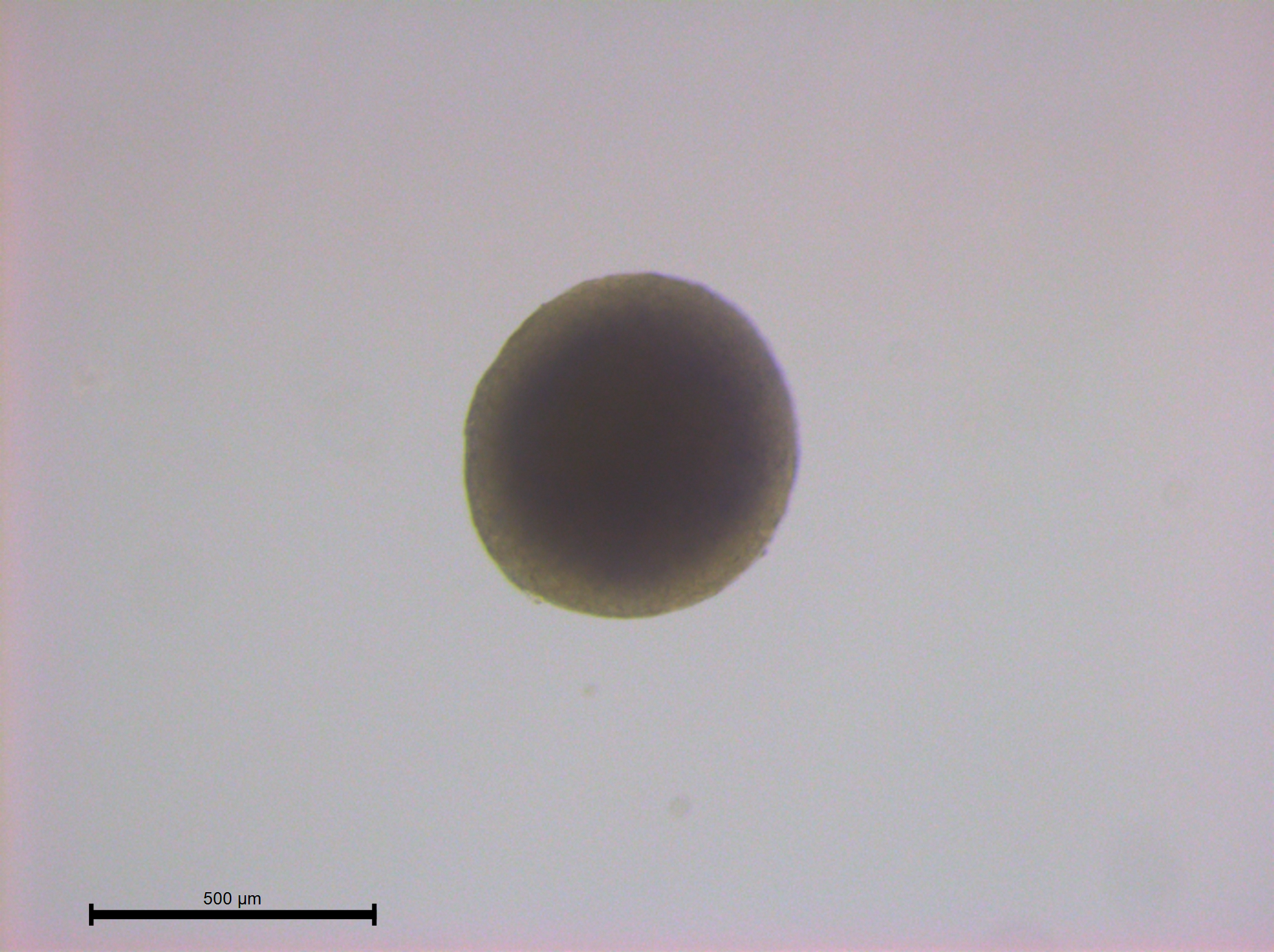

Supplement: Supplementary file 13 — Source data Fig. 2 [file 44318_2025_442_MOESM13_ESM.zip › Figure_2/Figure 2O/ca15b Mrbm24a.tif]

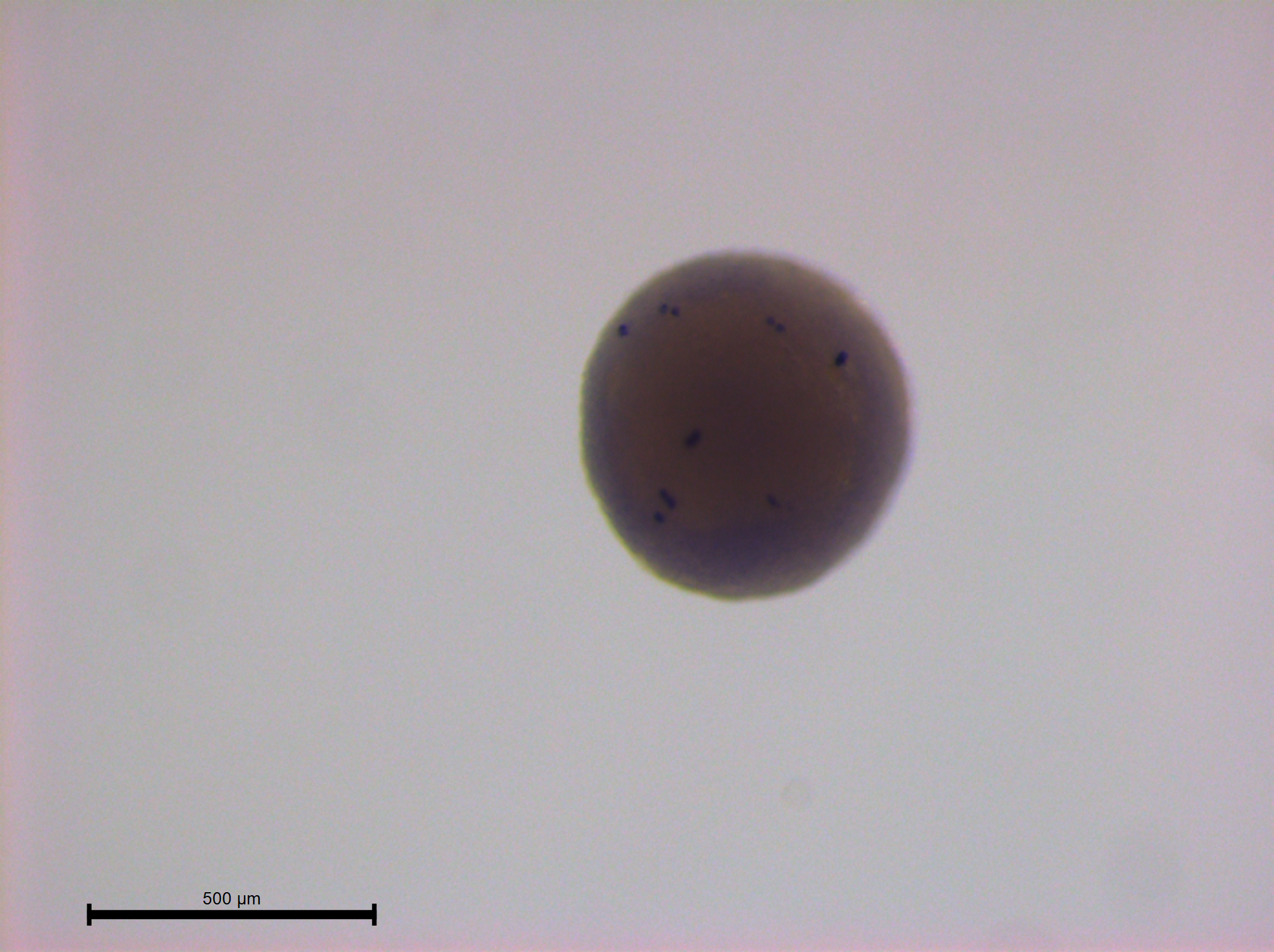

Supplement: Supplementary file 13 — Source data Fig. 2 [file 44318_2025_442_MOESM13_ESM.zip › Figure_2/Figure 2O/ca15b sibling.tif]

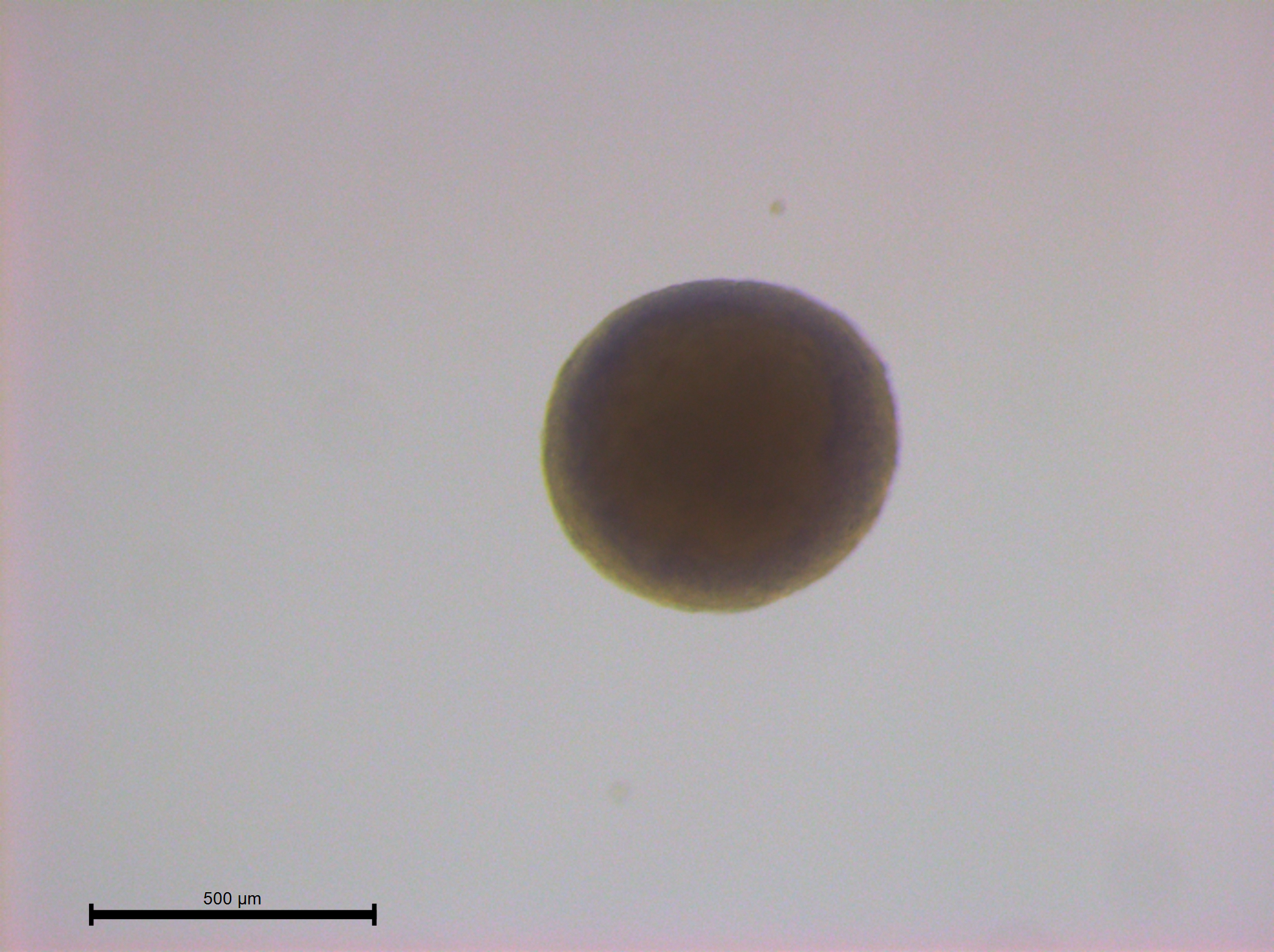

Supplement: Supplementary file 13 — Source data Fig. 2 [file 44318_2025_442_MOESM13_ESM.zip › Figure_2/Figure 2O/ddx4 Mrbm24a .tif]

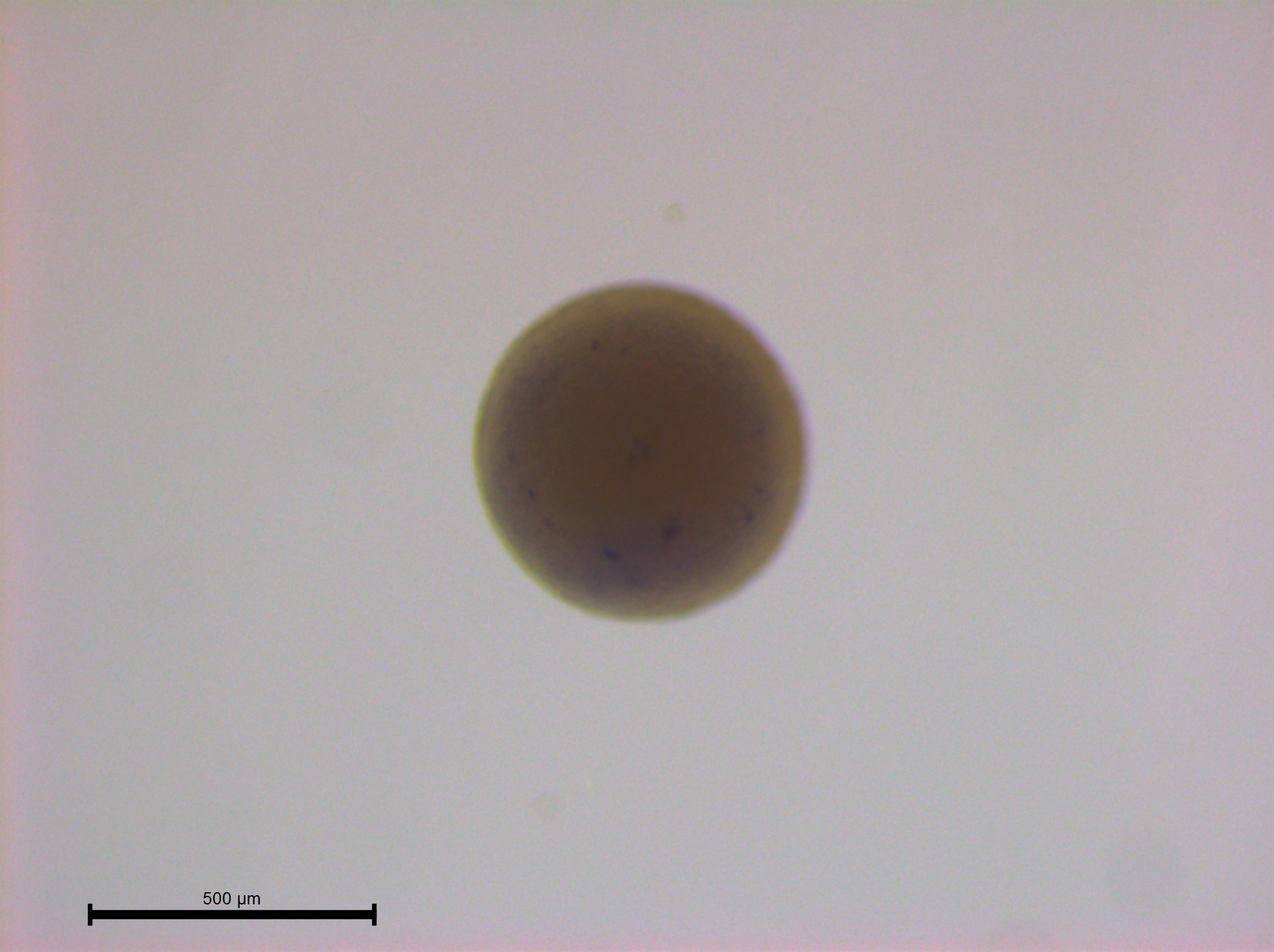

Supplement: Supplementary file 13 — Source data Fig. 2 [file 44318_2025_442_MOESM13_ESM.zip › Figure_2/Figure 2O/ddx4 sibling .tif]

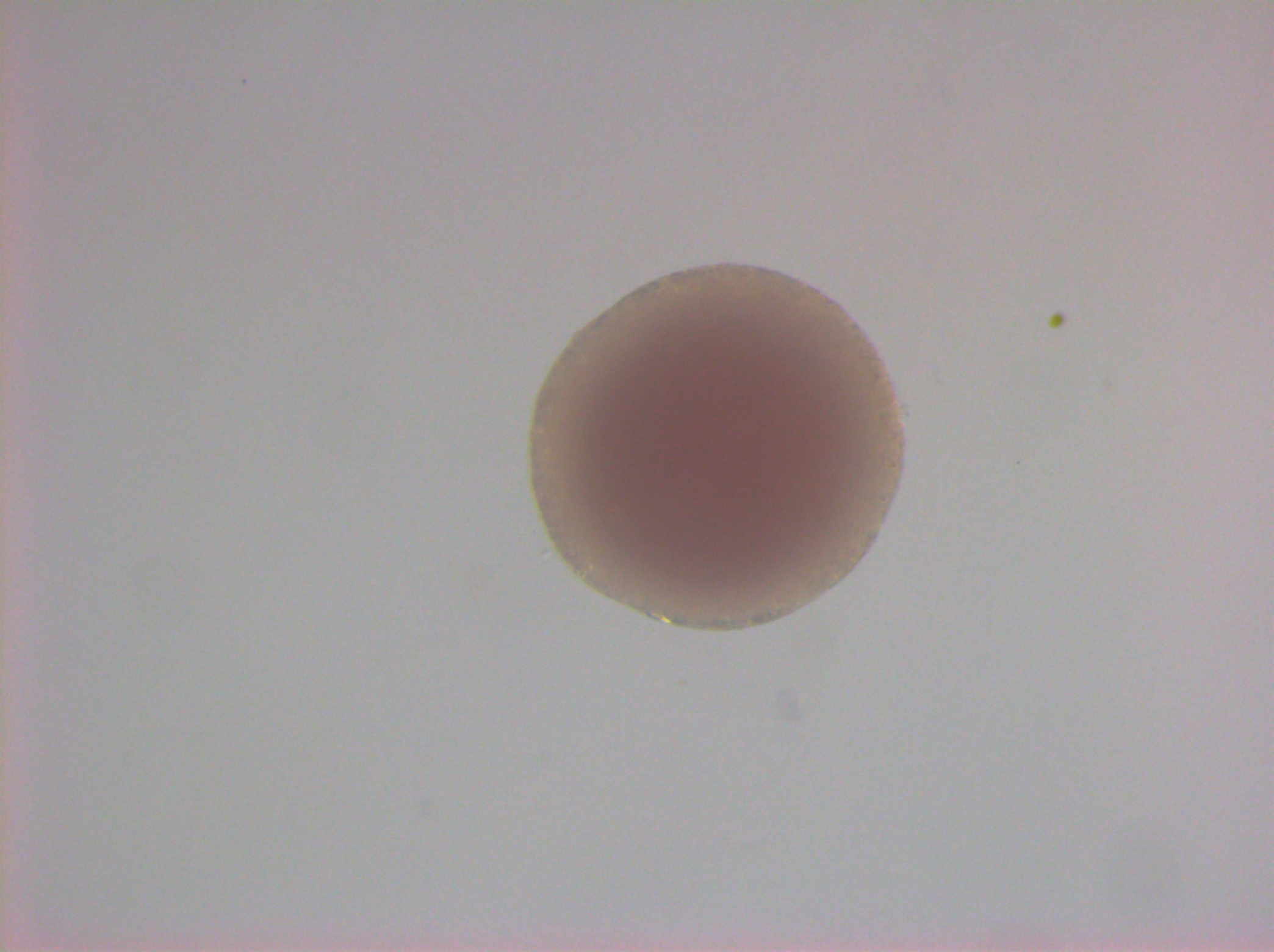

Supplement: Supplementary file 13 — Source data Fig. 2 [file 44318_2025_442_MOESM13_ESM.zip › Figure_2/Figure 2O/dnd1 Mrbm24a .tif]

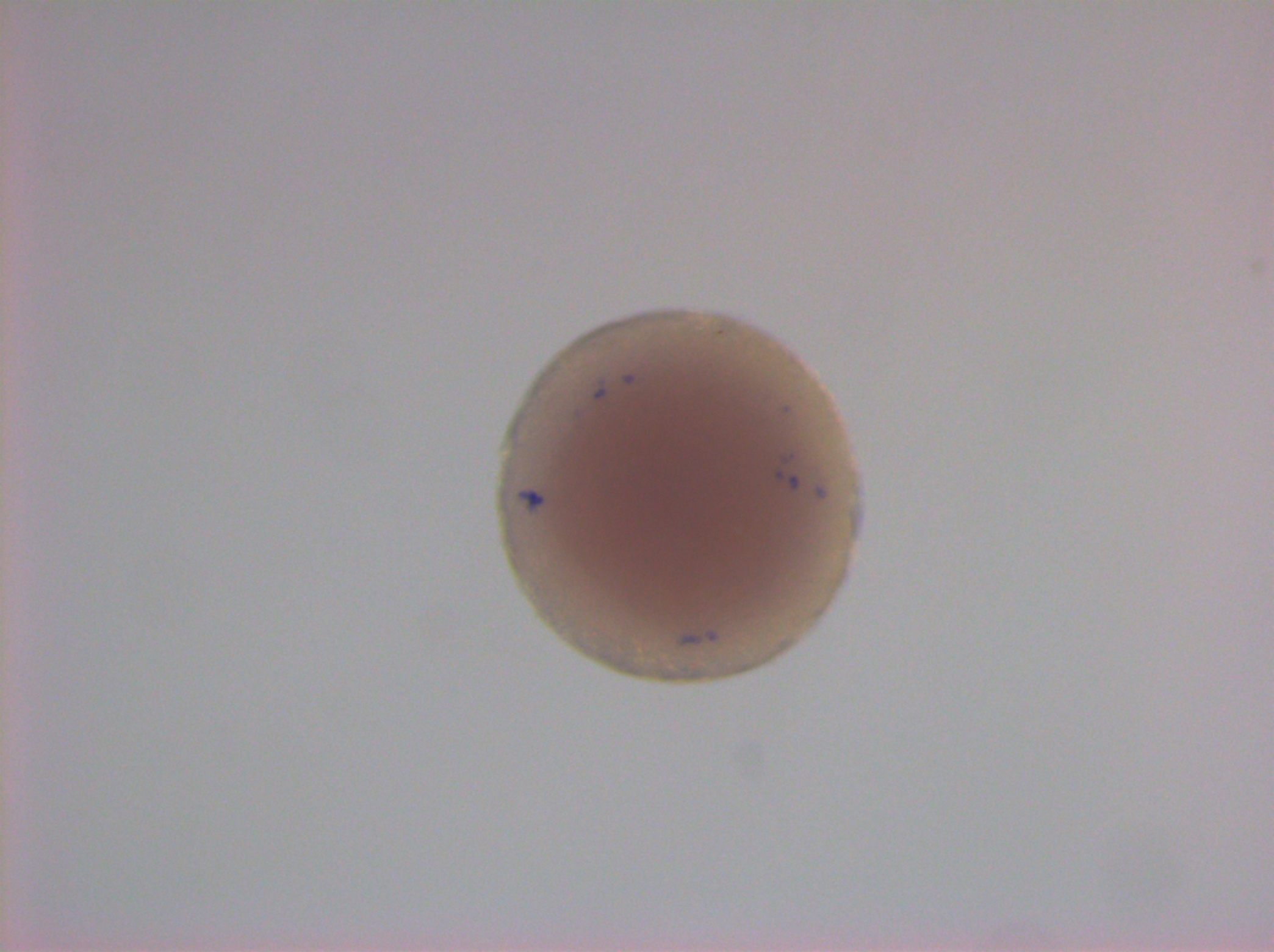

Supplement: Supplementary file 13 — Source data Fig. 2 [file 44318_2025_442_MOESM13_ESM.zip › Figure_2/Figure 2O/dnd1 sibling.tif]

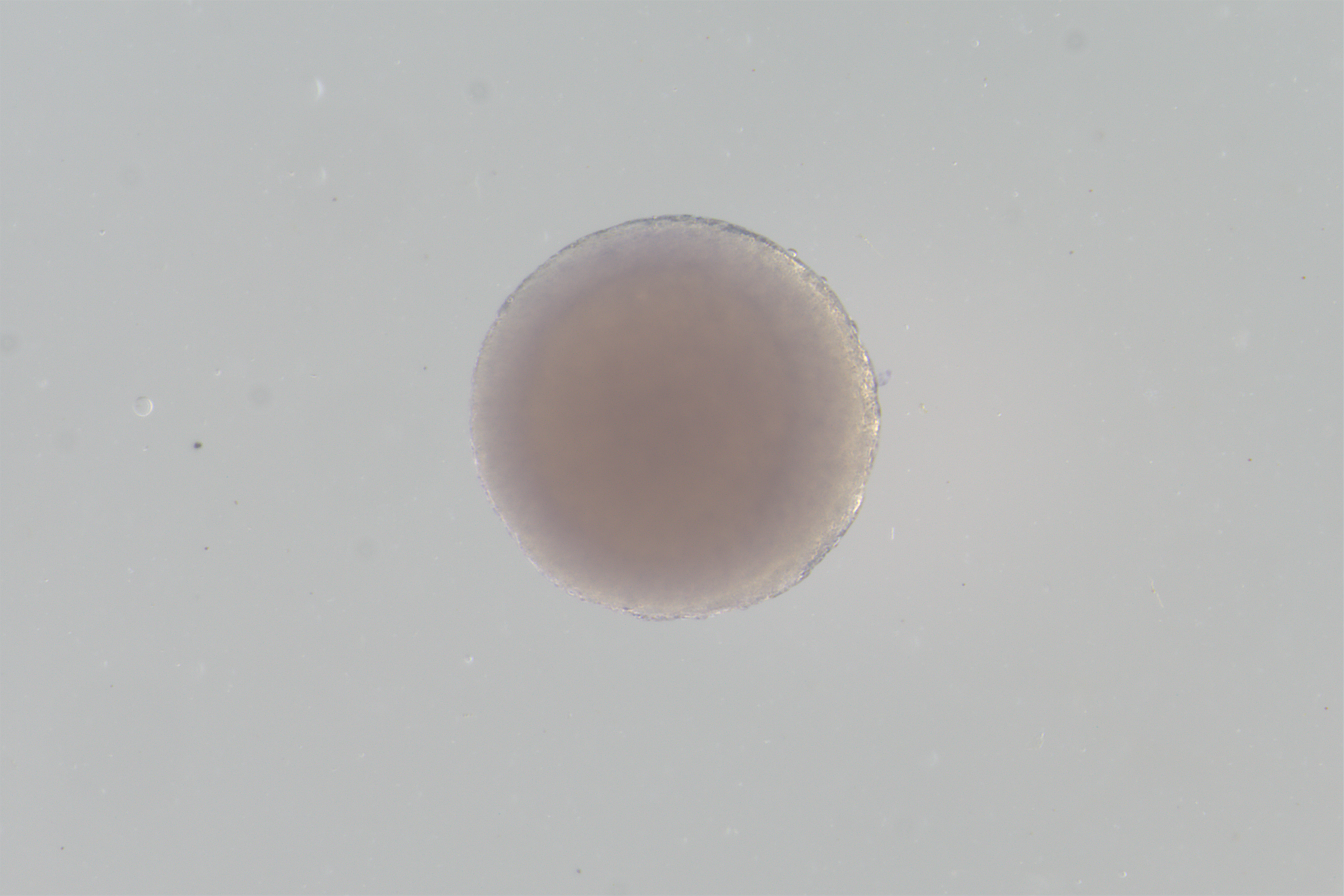

Supplement: Supplementary file 13 — Source data Fig. 2 [file 44318_2025_442_MOESM13_ESM.zip › Figure_2/Figure 2O/kop Mrbm24a.tif]

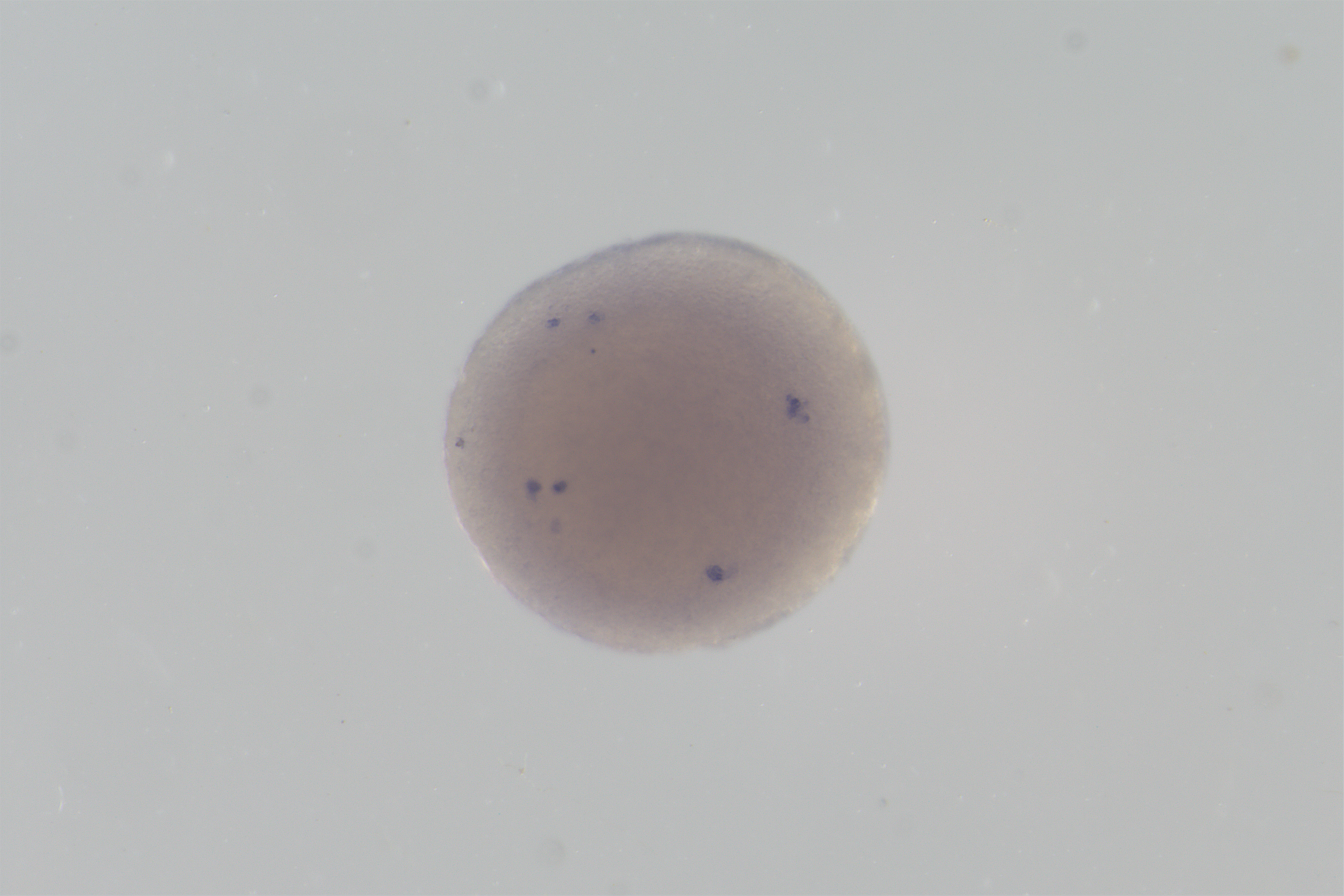

Supplement: Supplementary file 13 — Source data Fig. 2 [file 44318_2025_442_MOESM13_ESM.zip › Figure_2/Figure 2O/kop Sibling.tif]

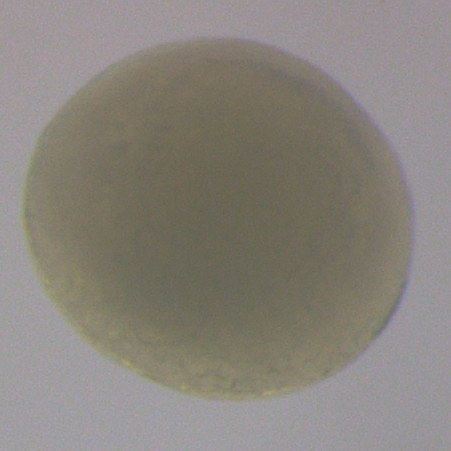

Supplement: Supplementary file 13 — Source data Fig. 2 [file 44318_2025_442_MOESM13_ESM.zip › Figure_2/Figure 2O/nanos3 Mrbm24a.tif]

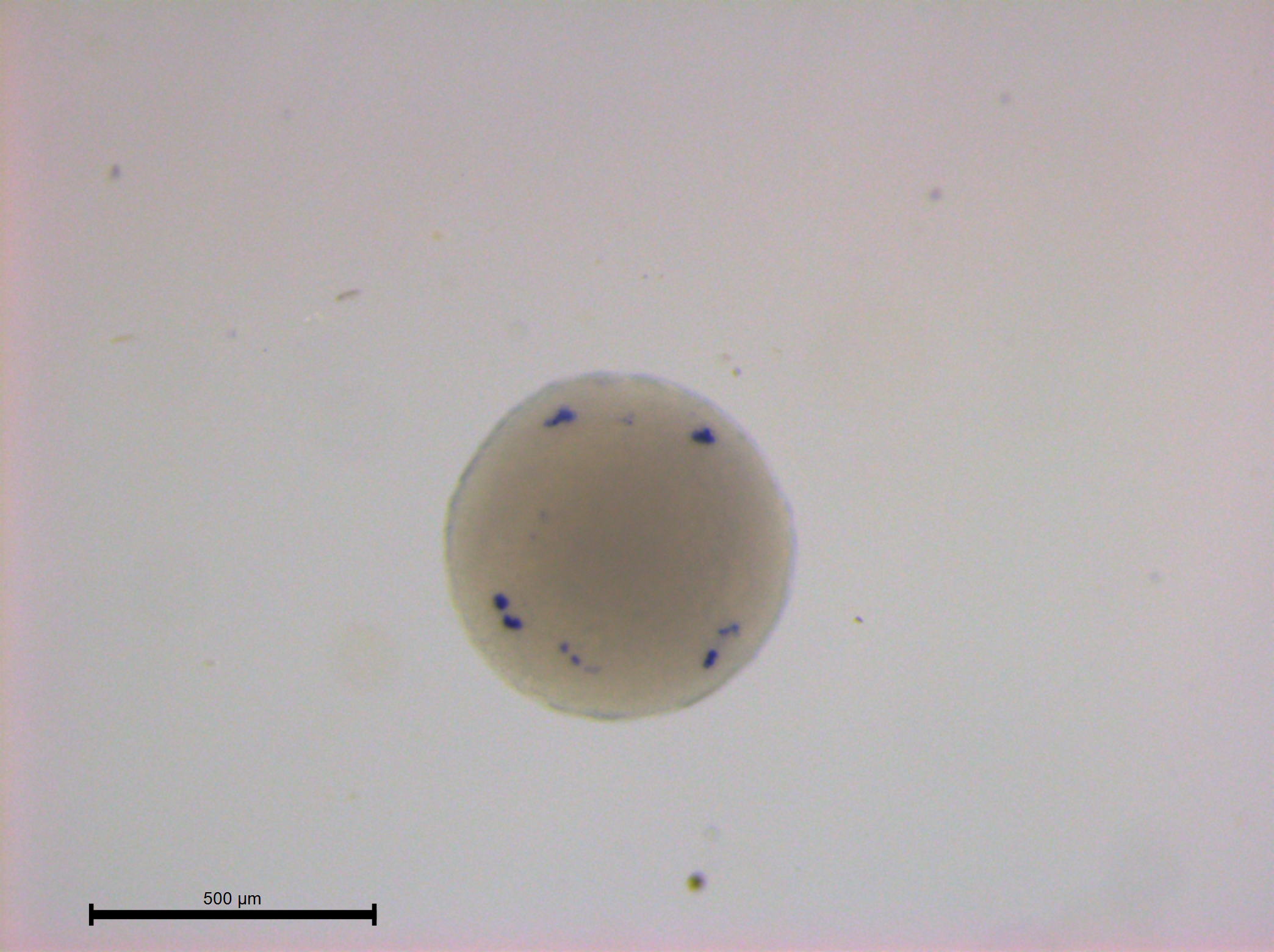

Supplement: Supplementary file 13 — Source data Fig. 2 [file 44318_2025_442_MOESM13_ESM.zip › Figure_2/Figure 2O/nanos3 sibling.tif]

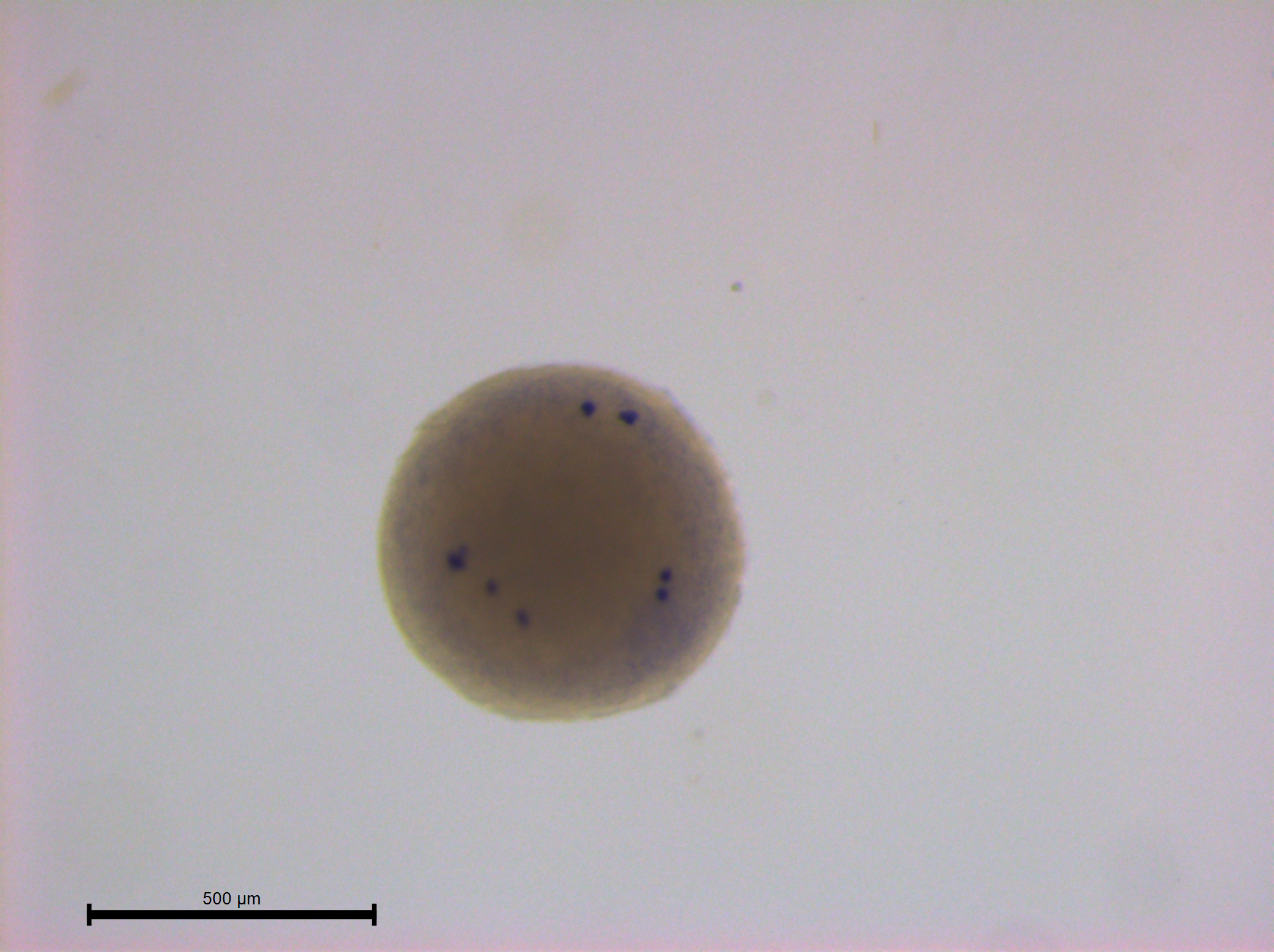

Supplement: Supplementary file 13 — Source data Fig. 2 [file 44318_2025_442_MOESM13_ESM.zip › Figure_2/Figure 2O/tdrd7a sibling .tif]

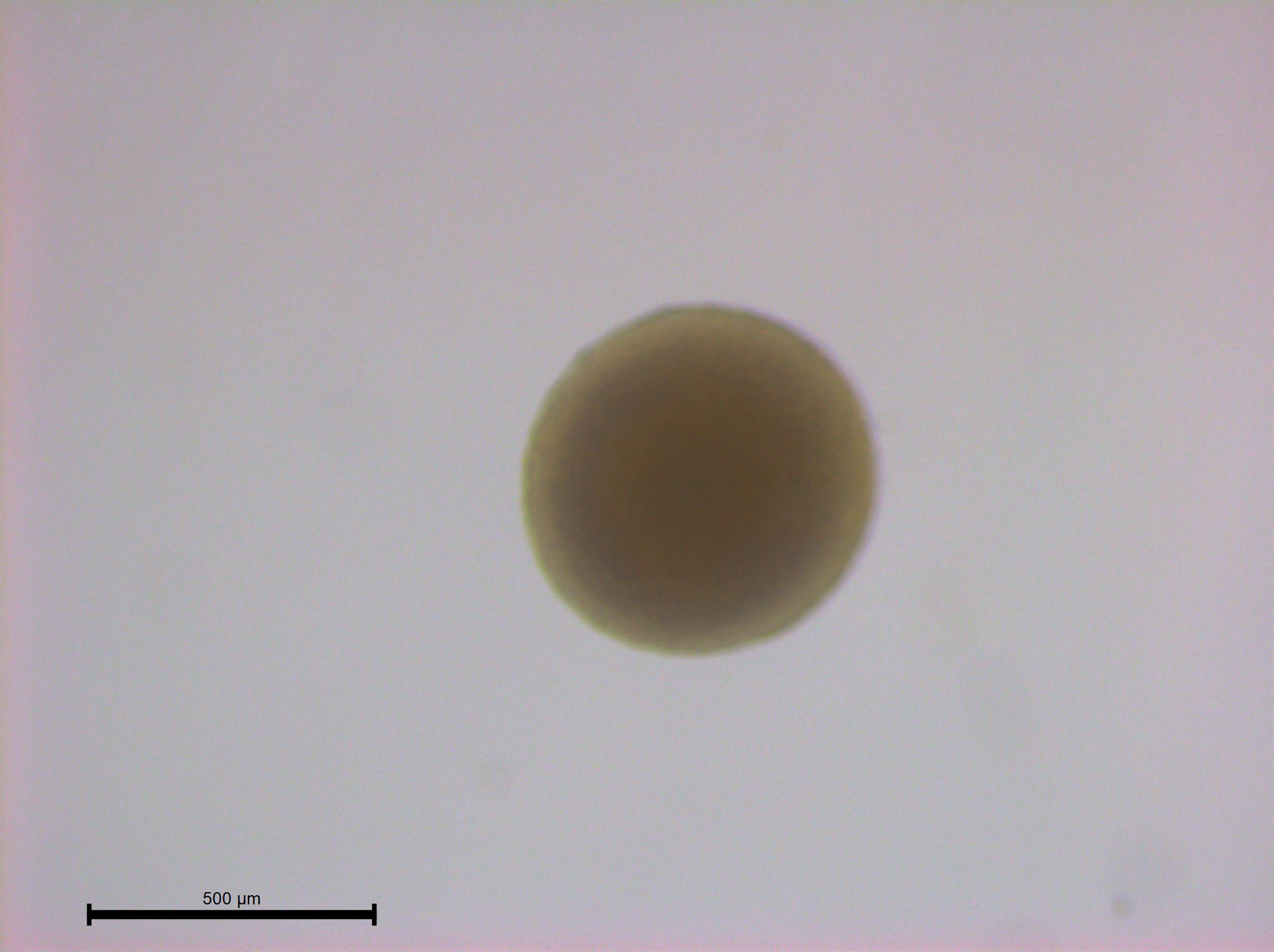

Supplement: Supplementary file 13 — Source data Fig. 2 [file 44318_2025_442_MOESM13_ESM.zip › Figure_2/Figure 2O/tdrd7a Mrbm24a .tif]

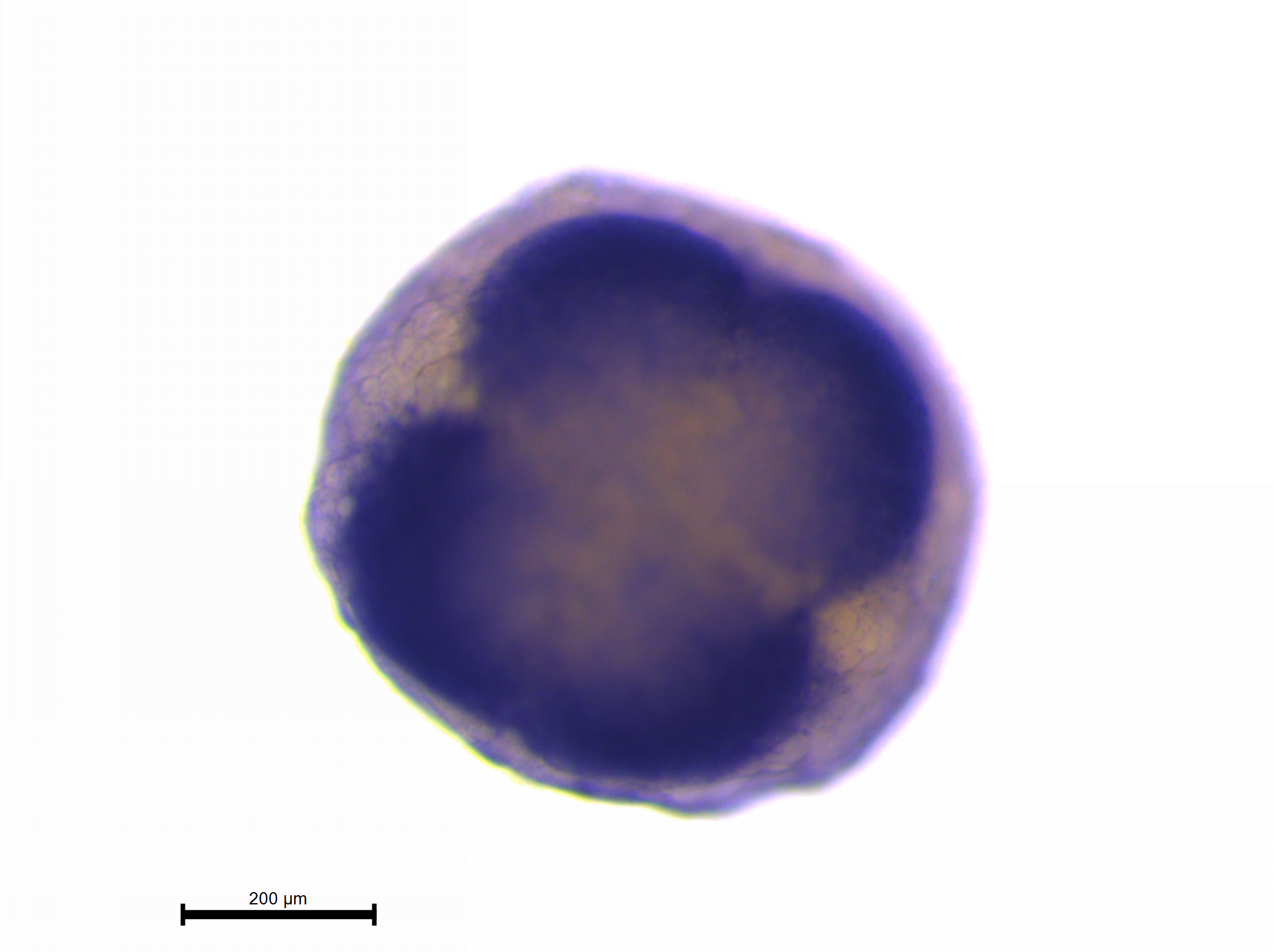

Supplement: Supplementary file 14 — Source data Fig. 3 [file 44318_2025_442_MOESM14_ESM.zip › Figure_3/Figure 3A/ca15b Mrbm24a .tif]

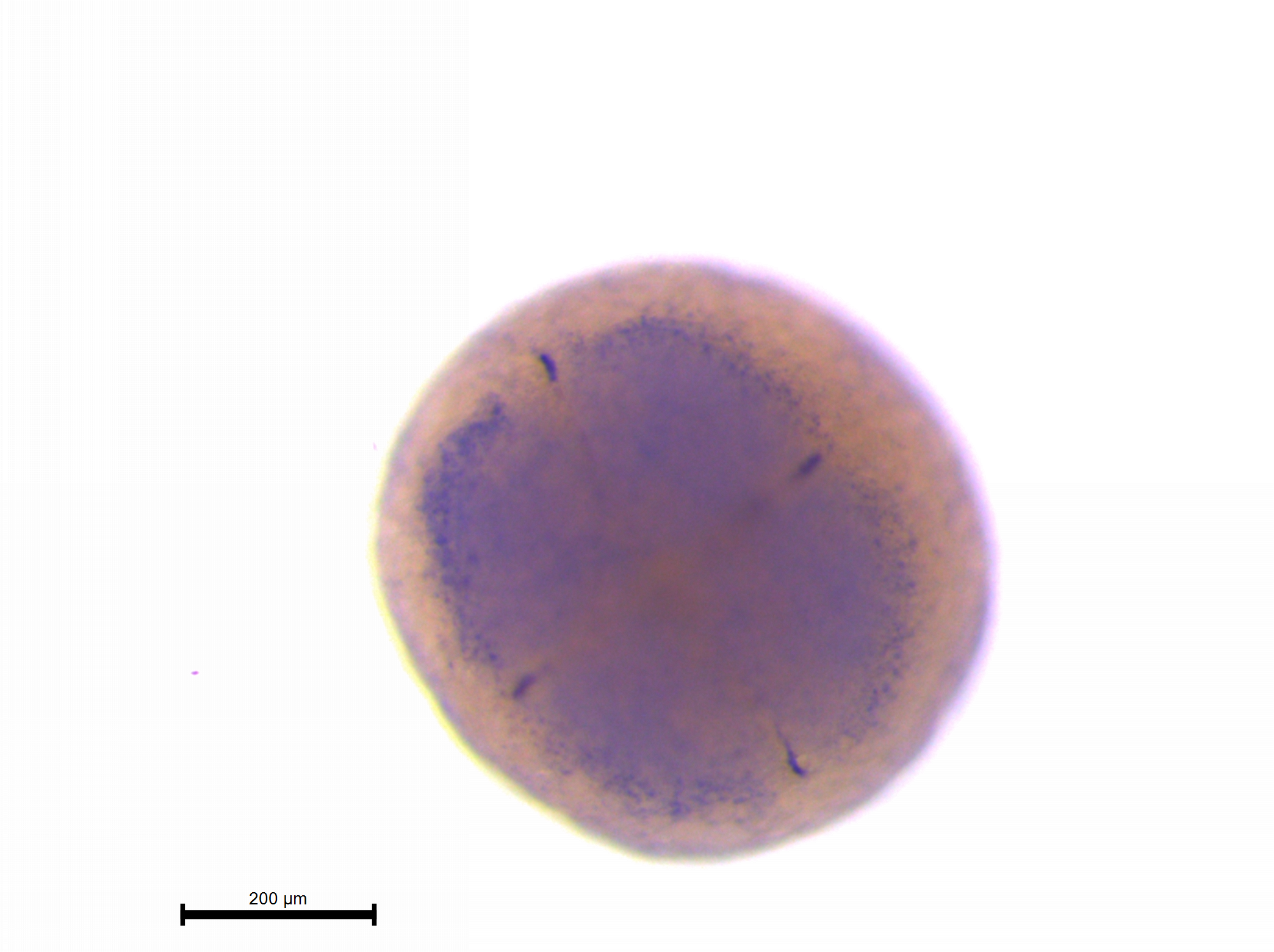

Supplement: Supplementary file 14 — Source data Fig. 3 [file 44318_2025_442_MOESM14_ESM.zip › Figure_3/Figure 3A/ca15b sibling.tif]

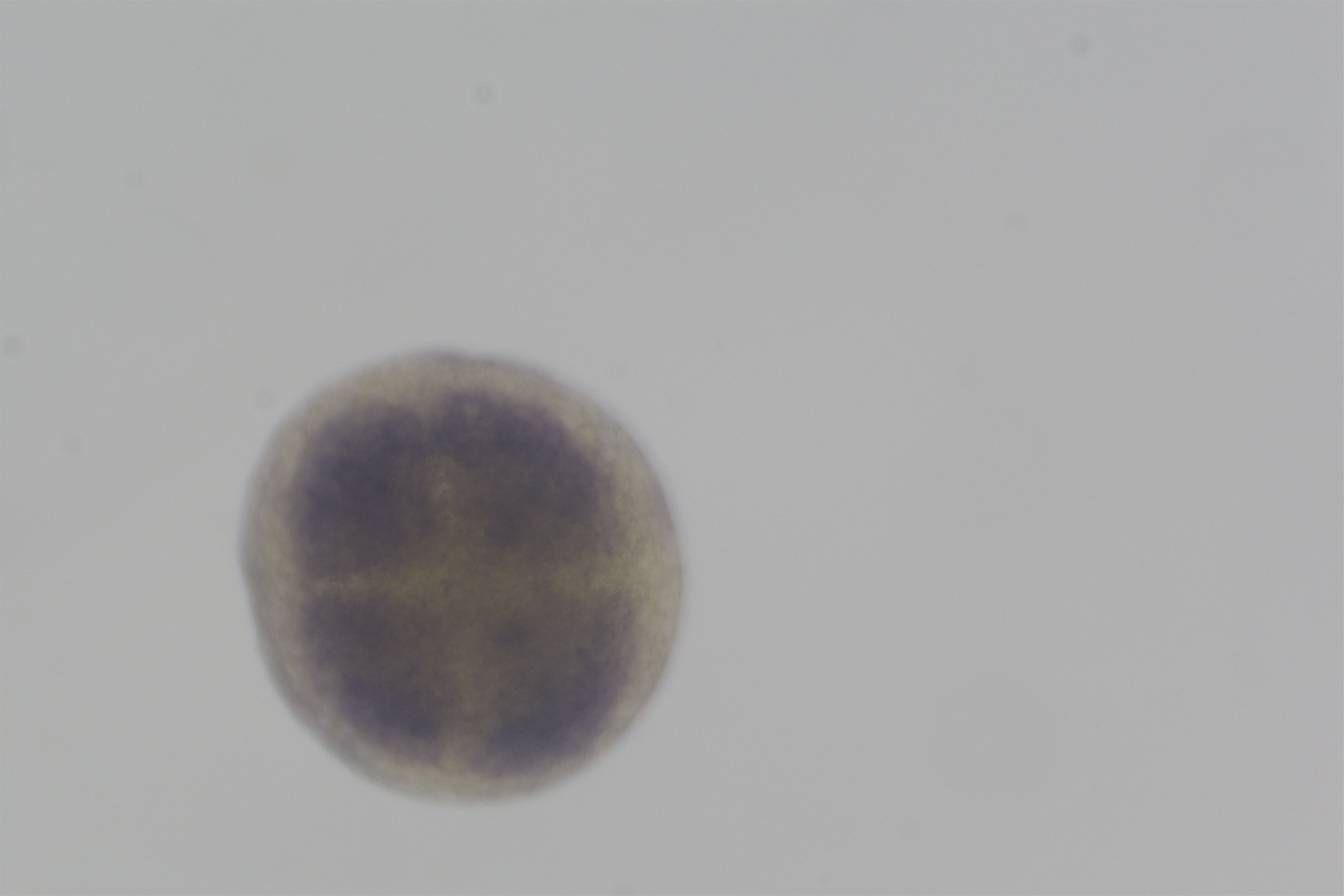

Supplement: Supplementary file 14 — Source data Fig. 3 [file 44318_2025_442_MOESM14_ESM.zip › Figure_3/Figure 3A/ddx4 Mrbm24a .tif]

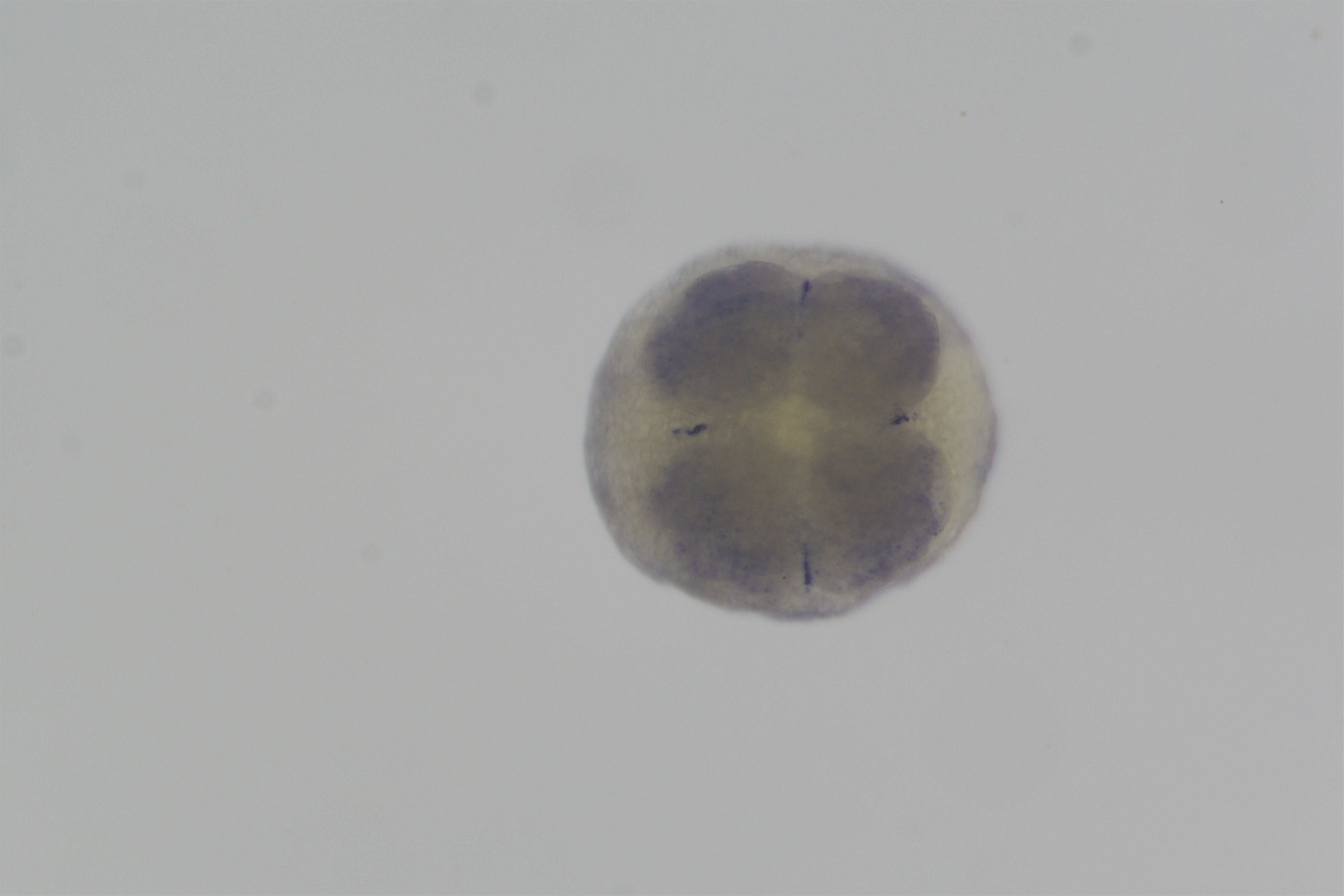

Supplement: Supplementary file 14 — Source data Fig. 3 [file 44318_2025_442_MOESM14_ESM.zip › Figure_3/Figure 3A/ddx4 sibling .tif]

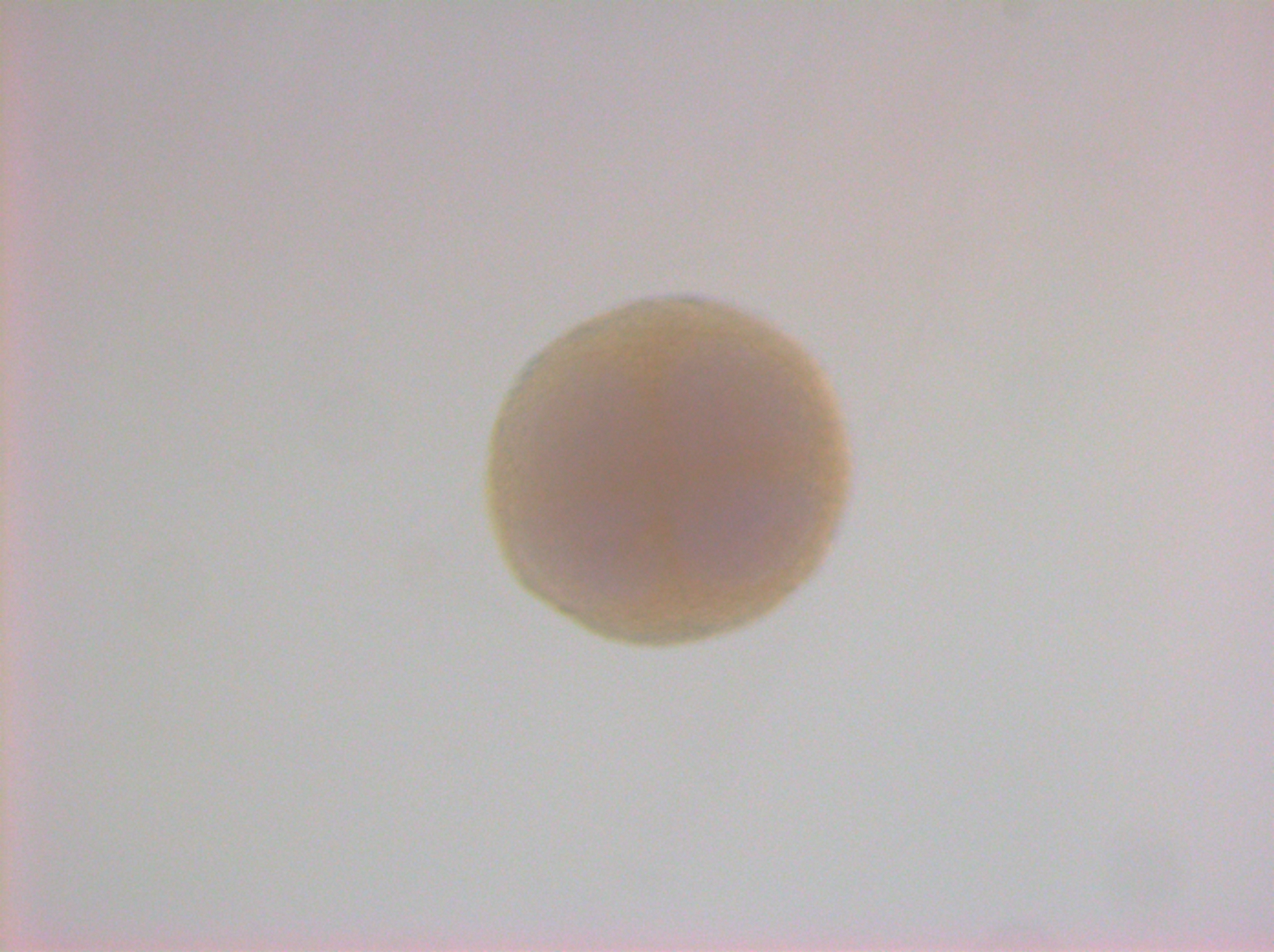

Supplement: Supplementary file 14 — Source data Fig. 3 [file 44318_2025_442_MOESM14_ESM.zip › Figure_3/Figure 3A/dnd1 Mrbm24a.tif]

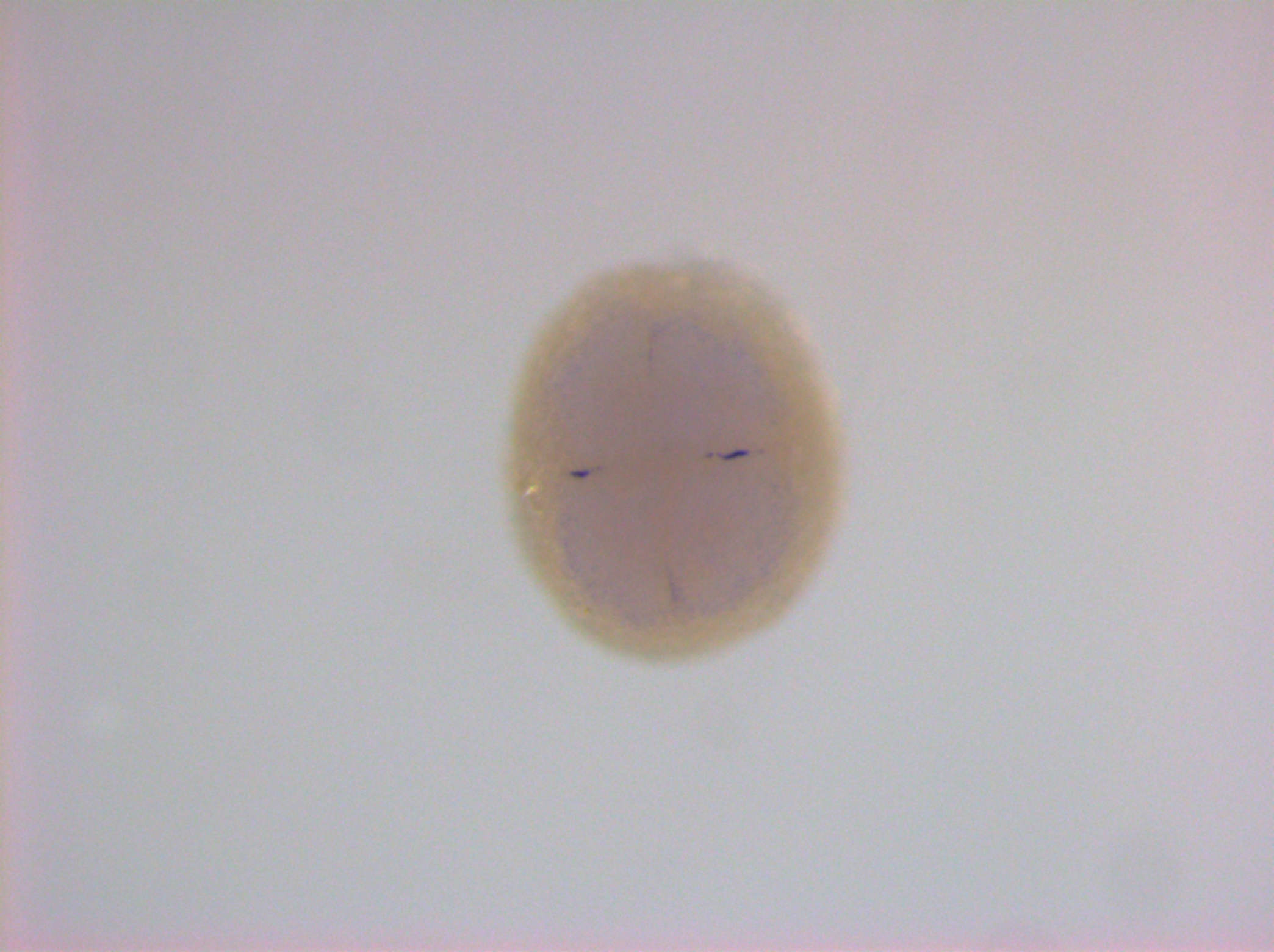

Supplement: Supplementary file 14 — Source data Fig. 3 [file 44318_2025_442_MOESM14_ESM.zip › Figure_3/Figure 3A/dnd1 sibling .tif]

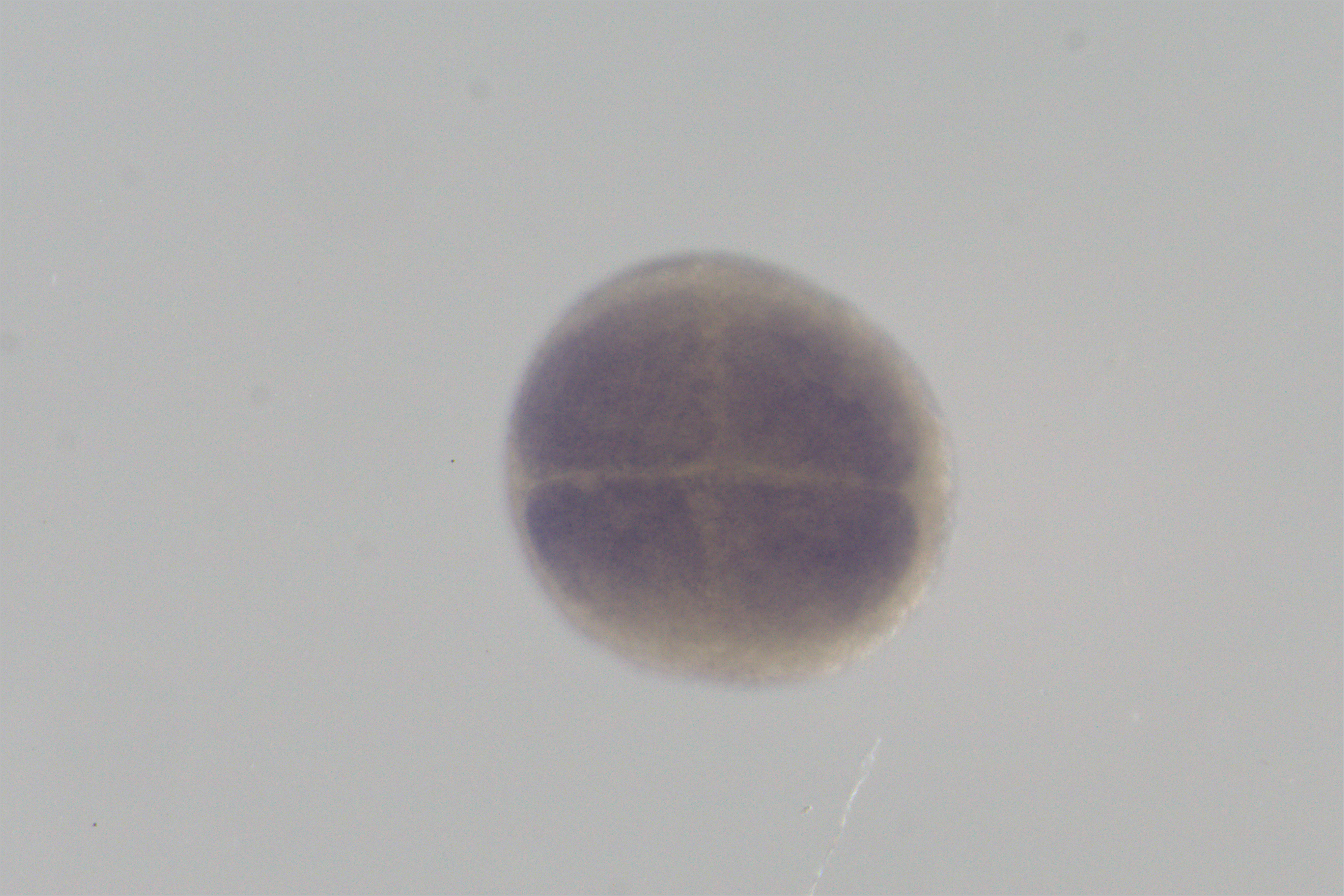

Supplement: Supplementary file 14 — Source data Fig. 3 [file 44318_2025_442_MOESM14_ESM.zip › Figure_3/Figure 3A/kop Mrbm24a.tif]

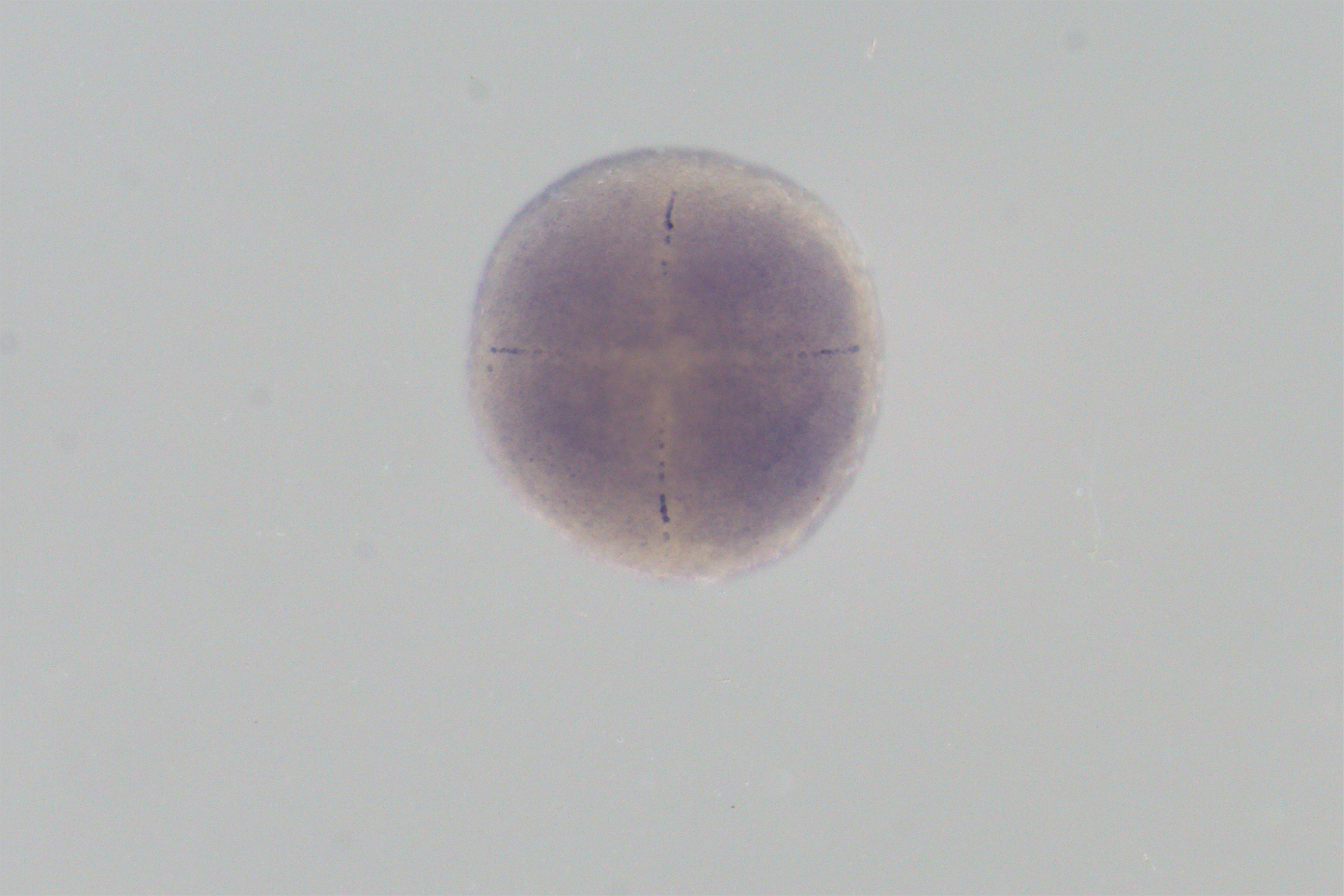

Supplement: Supplementary file 14 — Source data Fig. 3 [file 44318_2025_442_MOESM14_ESM.zip › Figure_3/Figure 3A/kop sibling.tif]

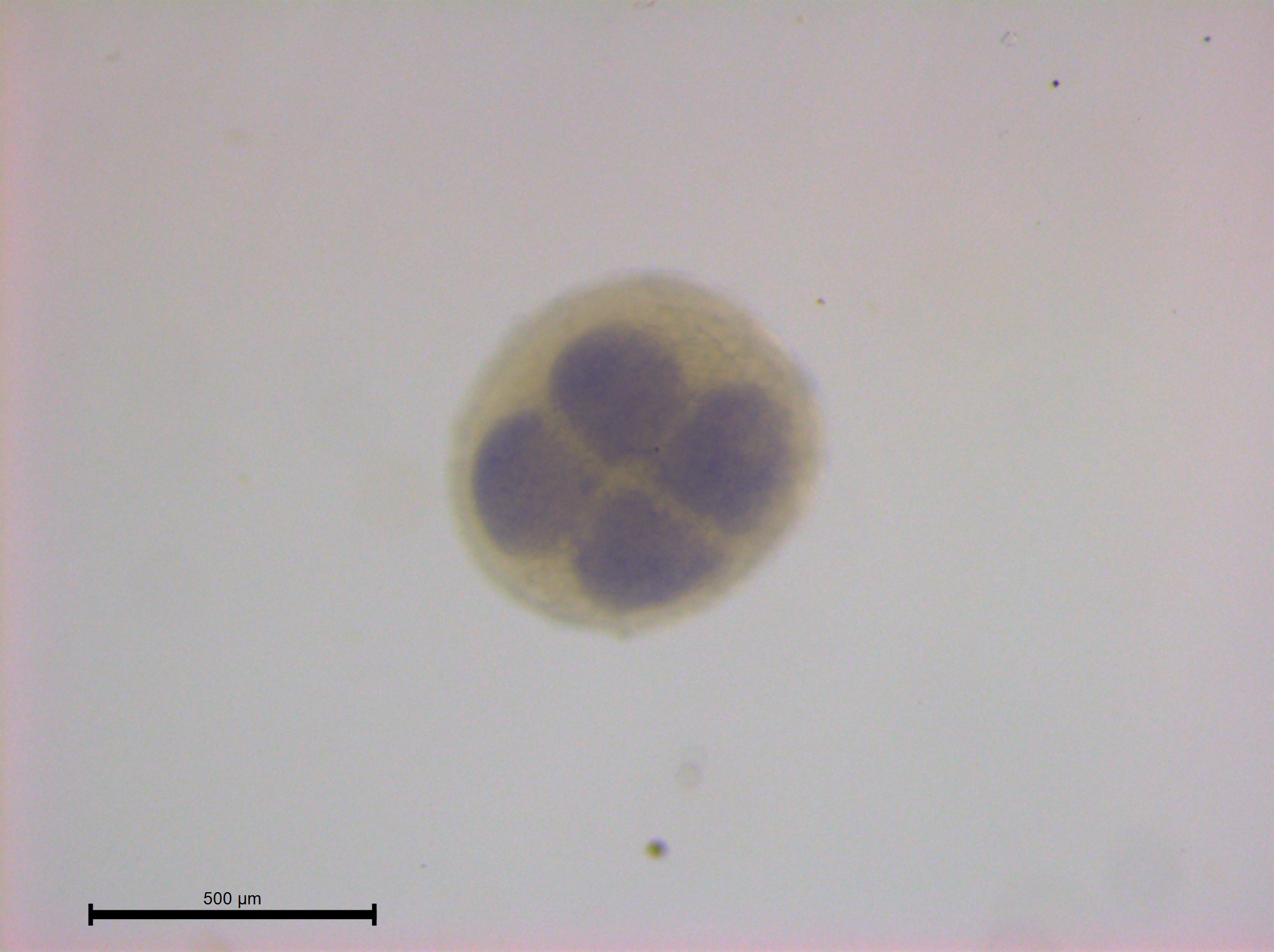

Supplement: Supplementary file 14 — Source data Fig. 3 [file 44318_2025_442_MOESM14_ESM.zip › Figure_3/Figure 3A/nanos3 Mrbm24a .tif]

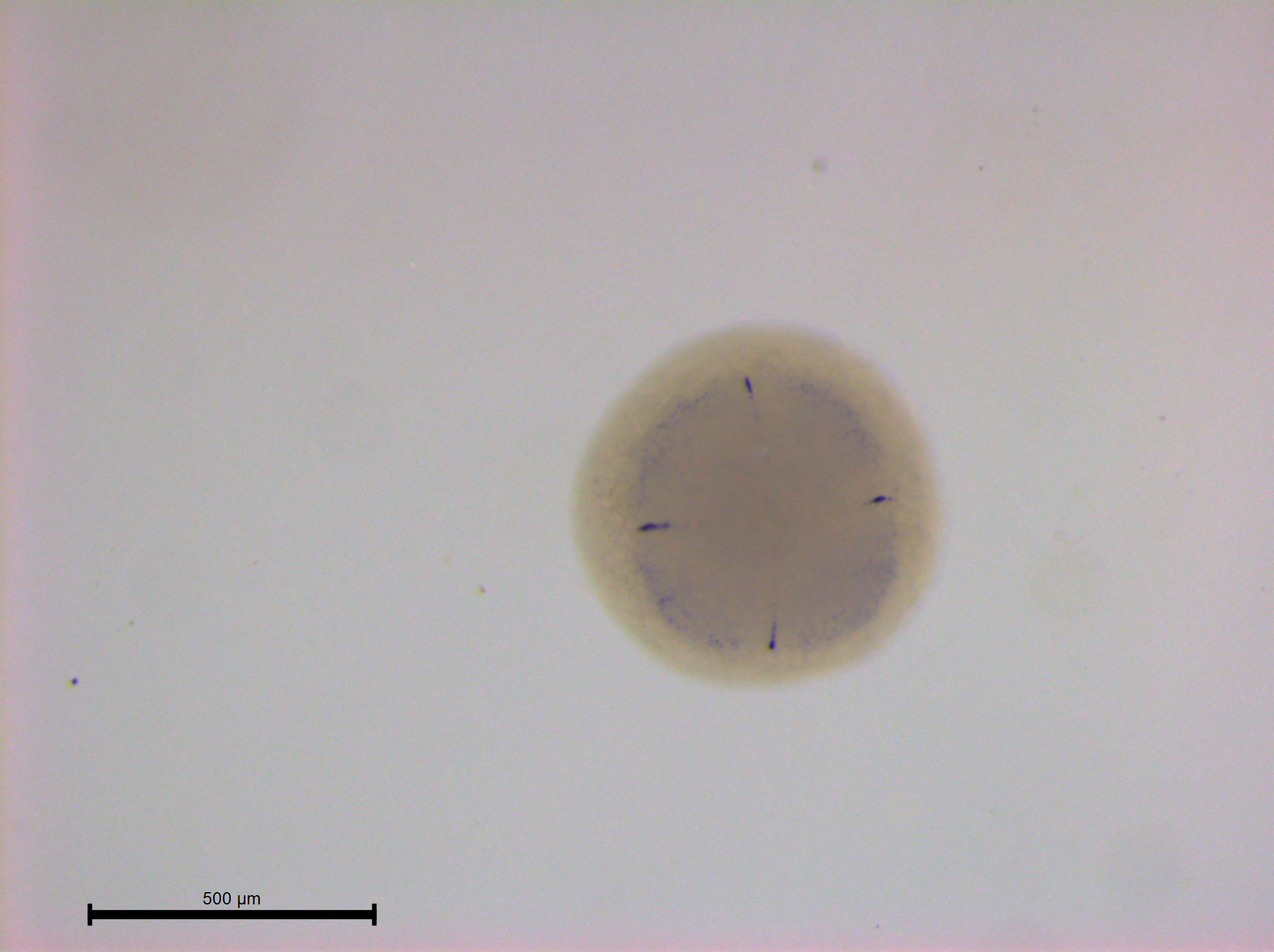

Supplement: Supplementary file 14 — Source data Fig. 3 [file 44318_2025_442_MOESM14_ESM.zip › Figure_3/Figure 3A/nanos3 sibling .tif]

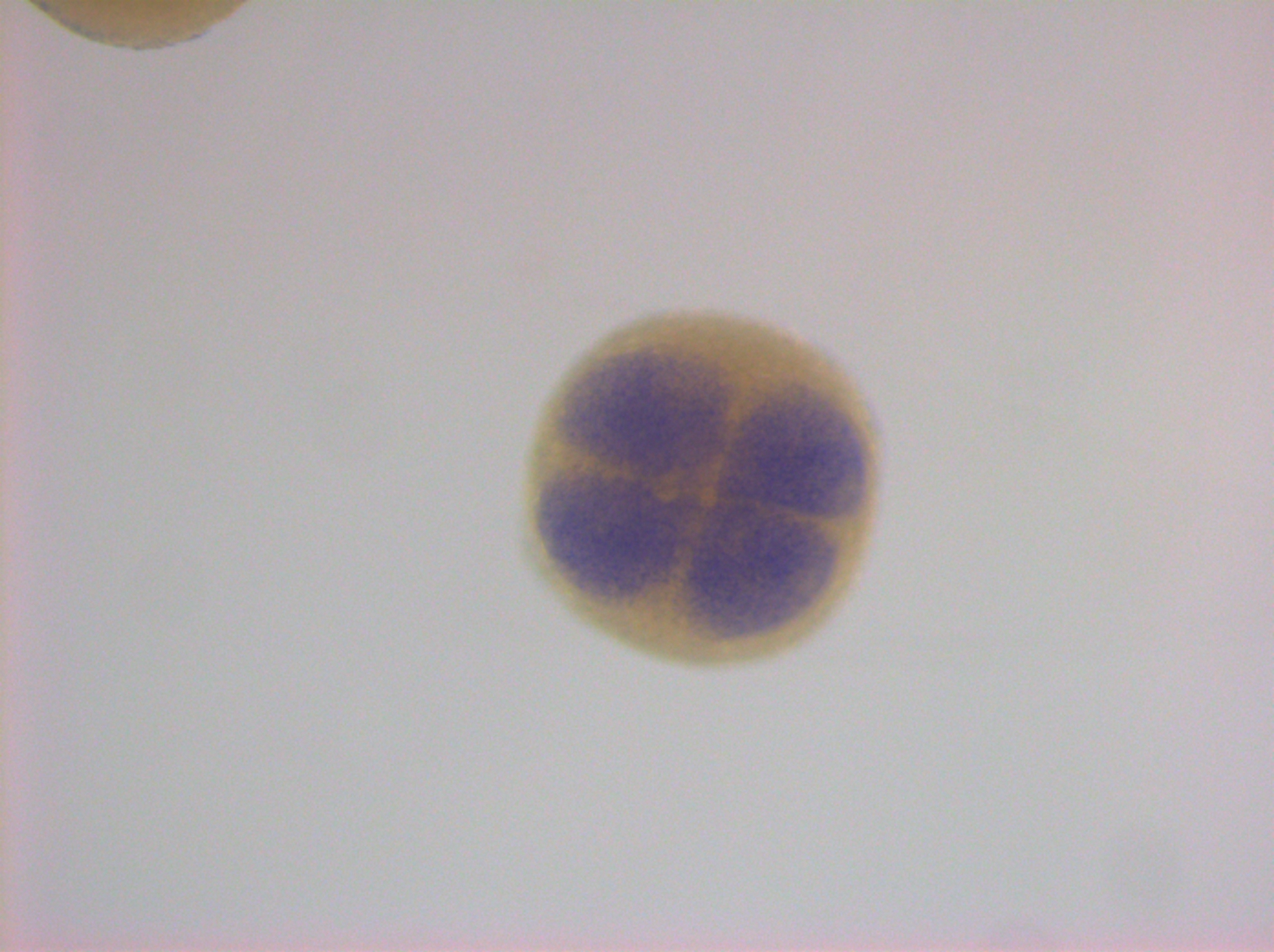

Supplement: Supplementary file 14 — Source data Fig. 3 [file 44318_2025_442_MOESM14_ESM.zip › Figure_3/Figure 3A/tdrd7a Mrbm24a .tif]

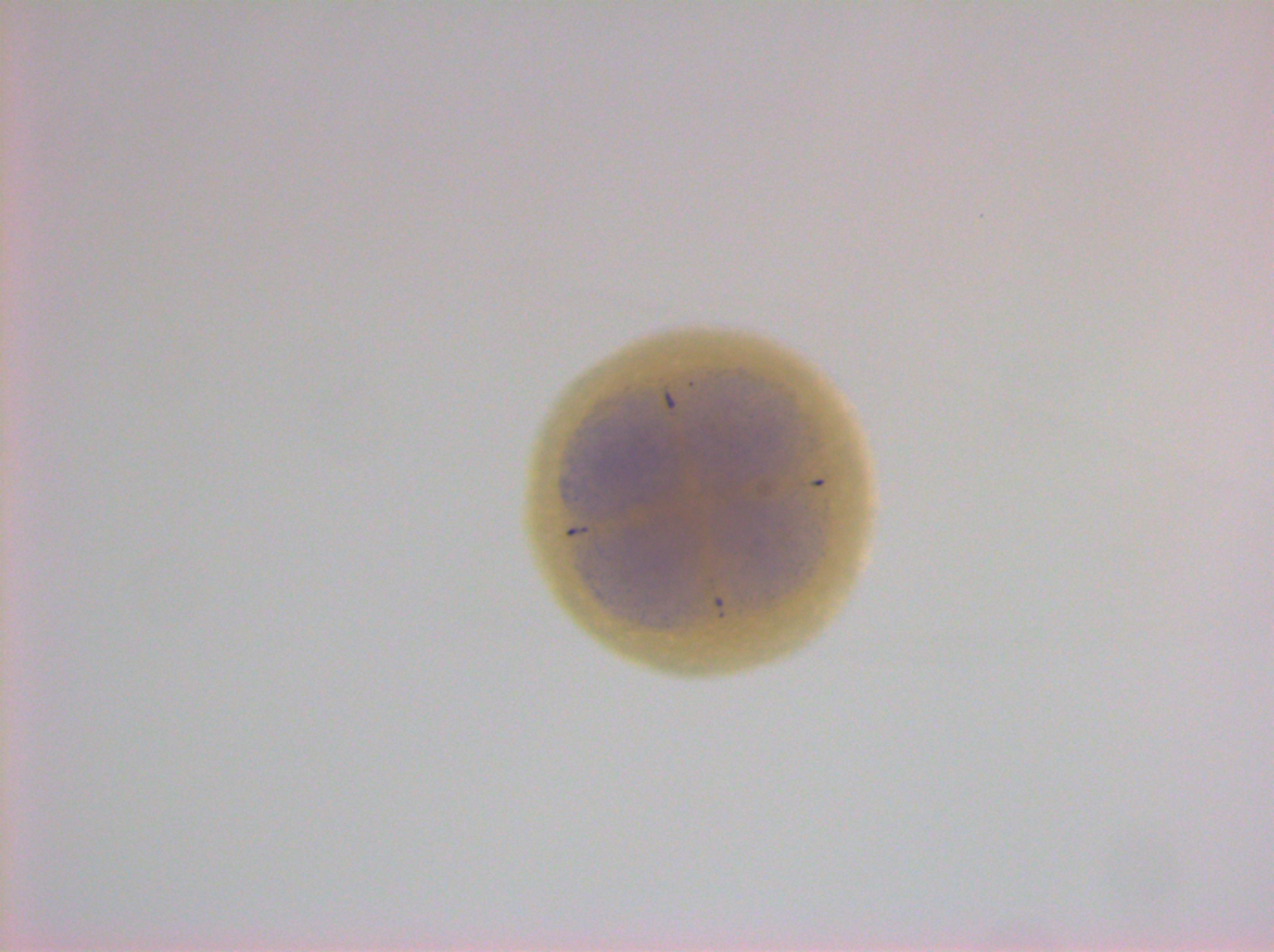

Supplement: Supplementary file 14 — Source data Fig. 3 [file 44318_2025_442_MOESM14_ESM.zip › Figure_3/Figure 3A/tdrd7a sibling.tif]

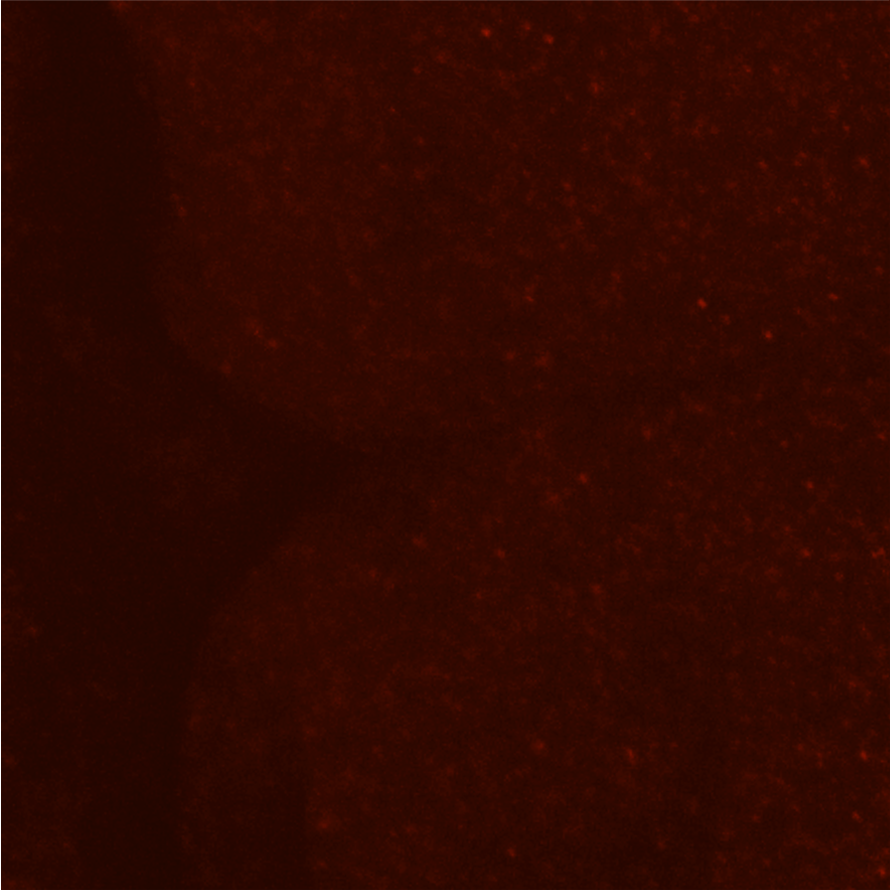

Supplement: Supplementary file 14 — Source data Fig. 3 [file 44318_2025_442_MOESM14_ESM.zip › Figure_3/Figure 3B/ca15b Mrbm24a.tif]

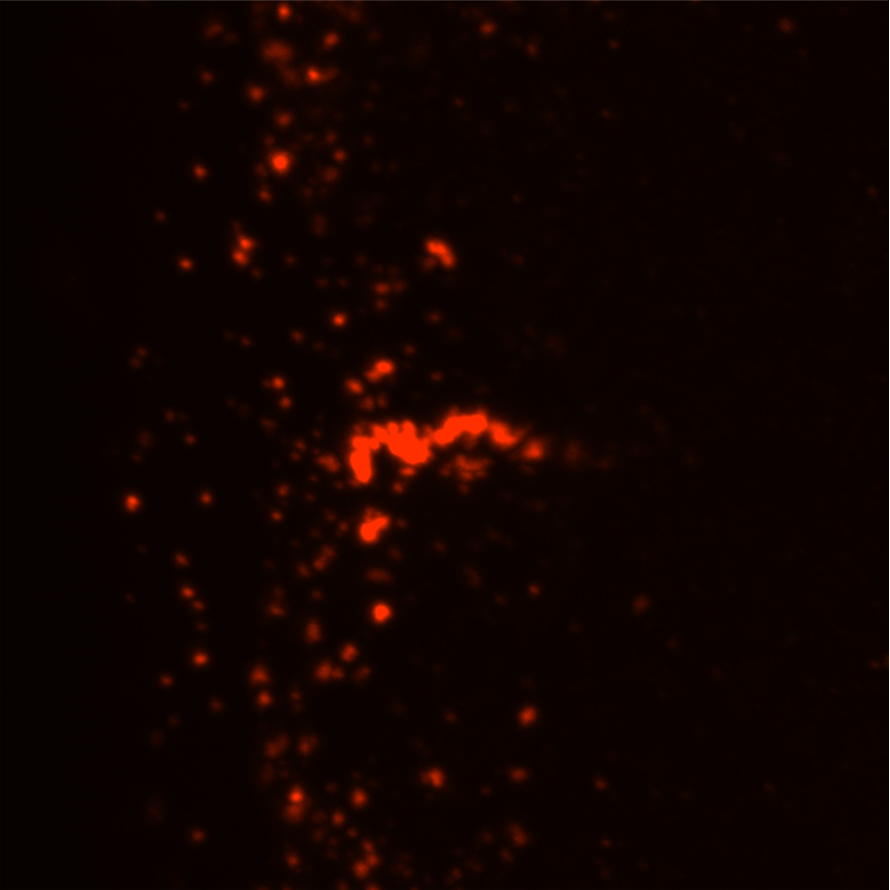

Supplement: Supplementary file 14 — Source data Fig. 3 [file 44318_2025_442_MOESM14_ESM.zip › Figure_3/Figure 3B/ca15b sibling.tif]

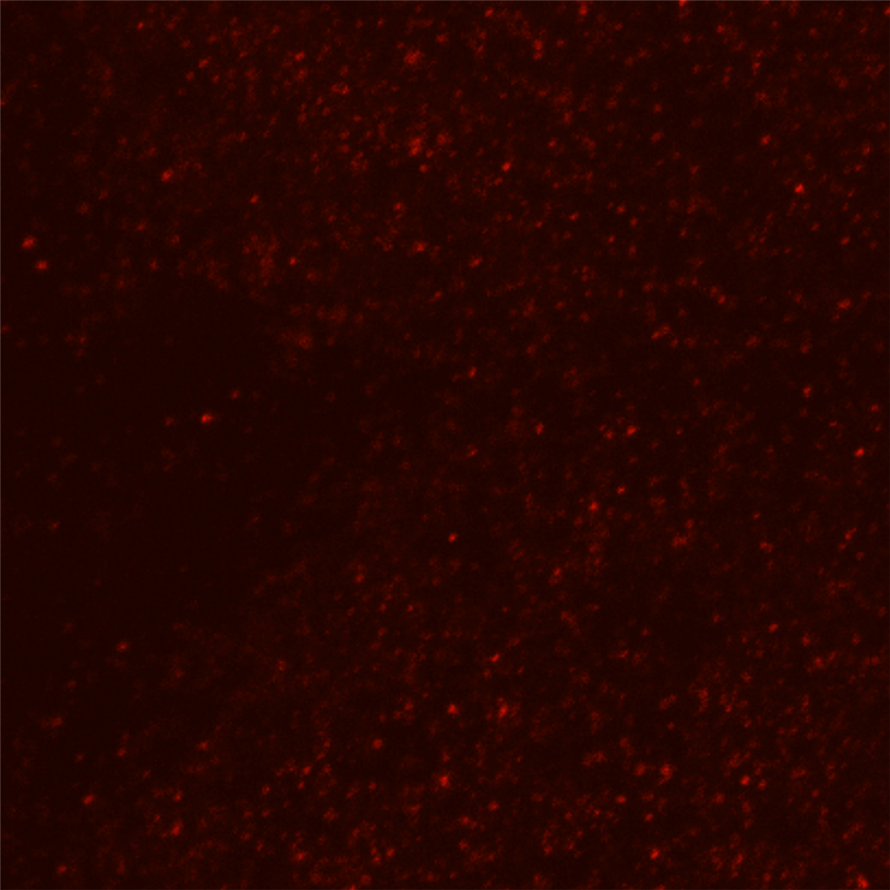

Supplement: Supplementary file 14 — Source data Fig. 3 [file 44318_2025_442_MOESM14_ESM.zip › Figure_3/Figure 3B/ddx4 Mrbm24a.tif]

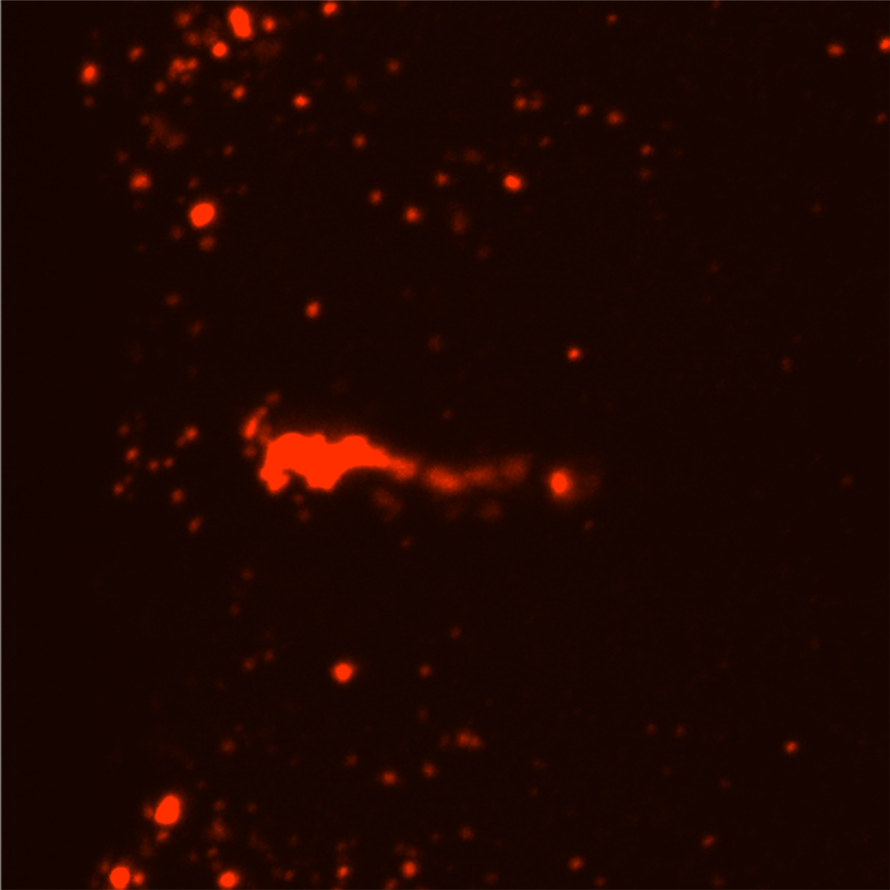

Supplement: Supplementary file 14 — Source data Fig. 3 [file 44318_2025_442_MOESM14_ESM.zip › Figure_3/Figure 3B/ddx4 sibling.tif]

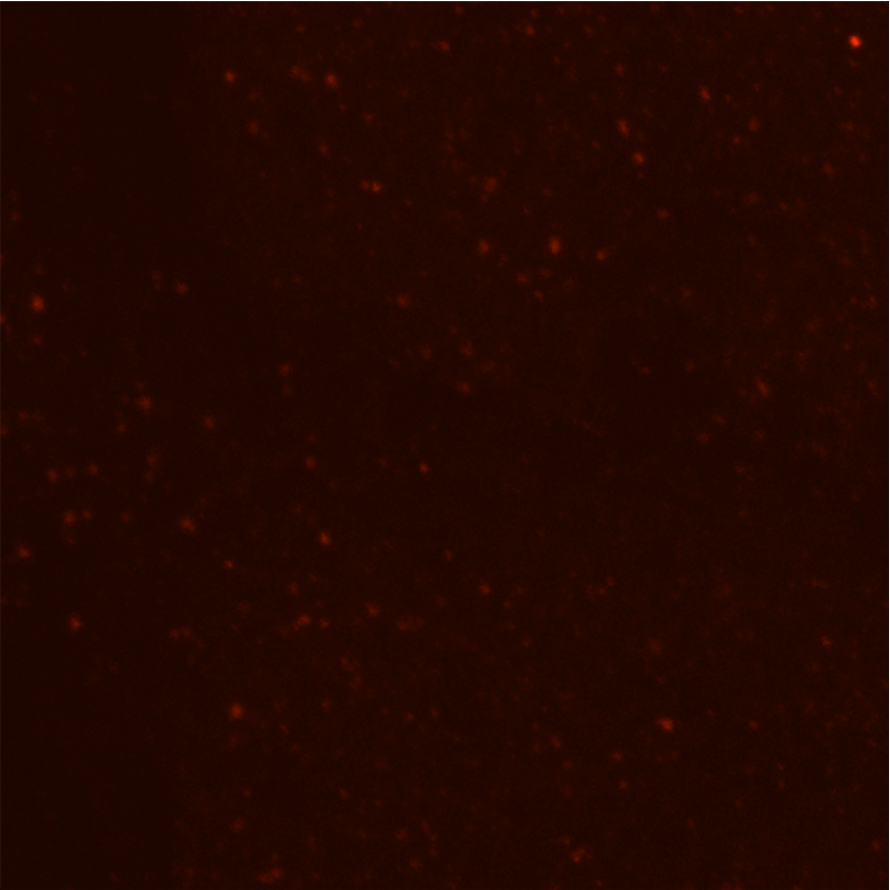

Supplement: Supplementary file 14 — Source data Fig. 3 [file 44318_2025_442_MOESM14_ESM.zip › Figure_3/Figure 3B/dnd1 Mrbm24a .tif]

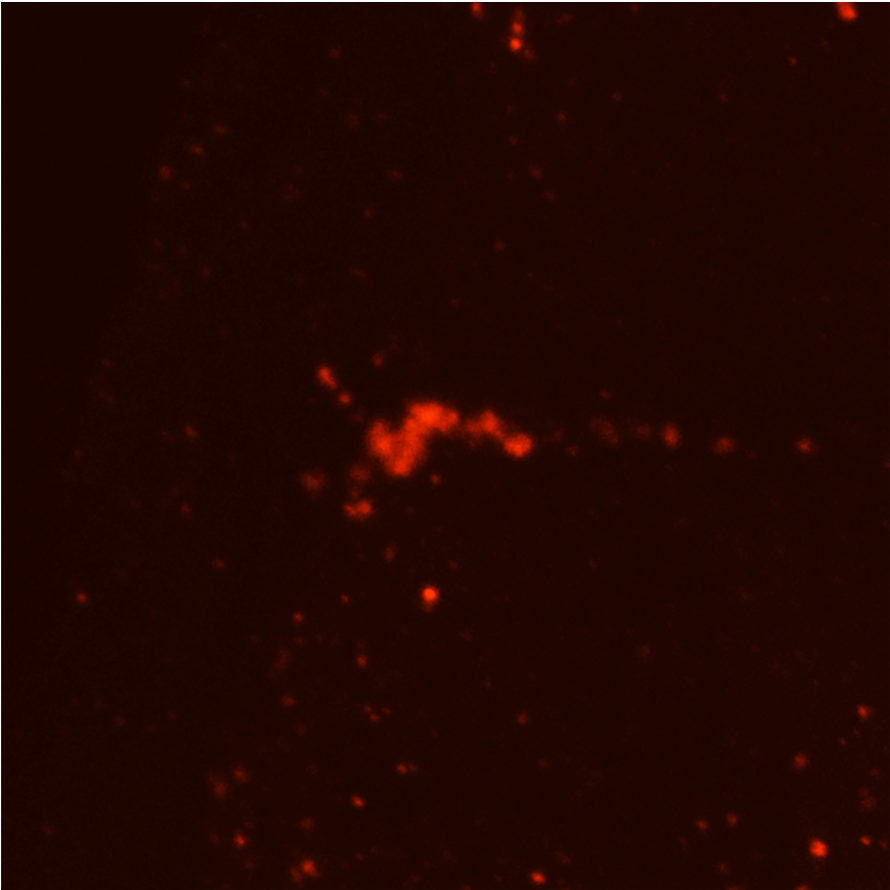

Supplement: Supplementary file 14 — Source data Fig. 3 [file 44318_2025_442_MOESM14_ESM.zip › Figure_3/Figure 3B/dnd1 sibling.tif]

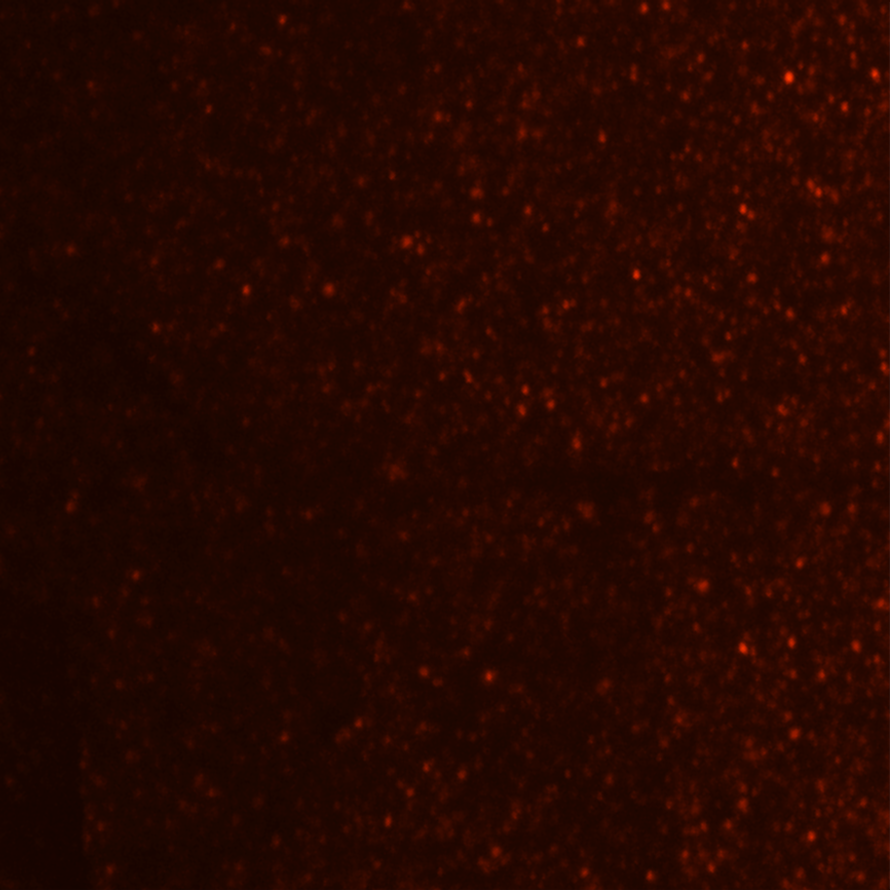

Supplement: Supplementary file 14 — Source data Fig. 3 [file 44318_2025_442_MOESM14_ESM.zip › Figure_3/Figure 3B/kop Mrbm24a.tif]

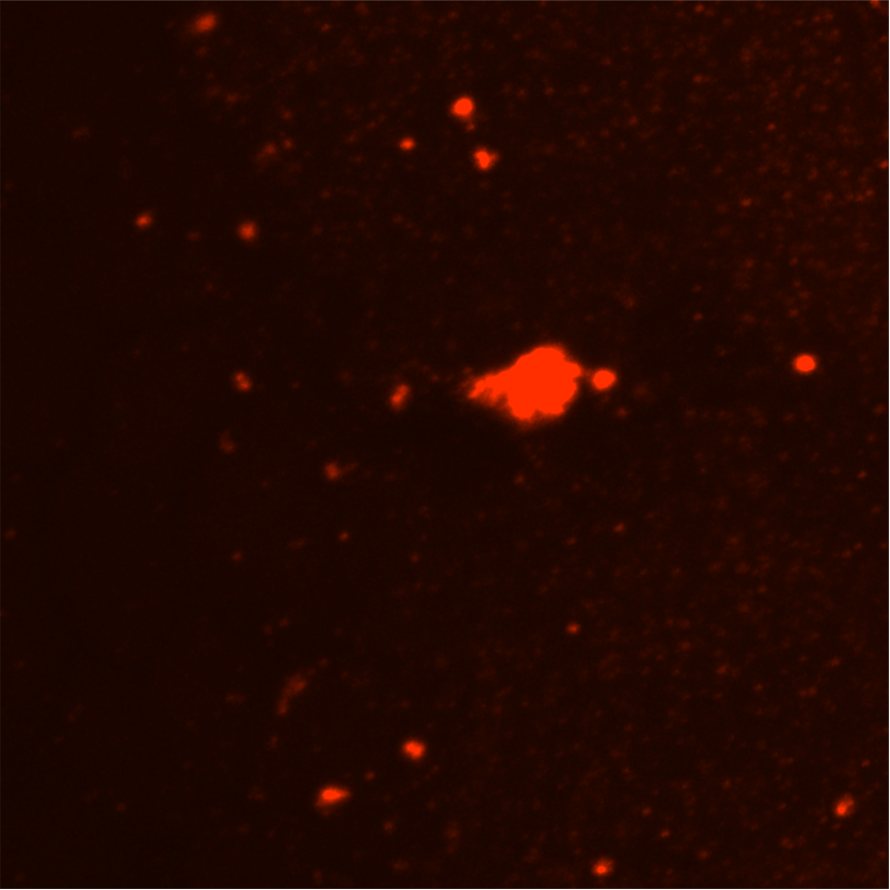

Supplement: Supplementary file 14 — Source data Fig. 3 [file 44318_2025_442_MOESM14_ESM.zip › Figure_3/Figure 3B/kop sibling.tif]

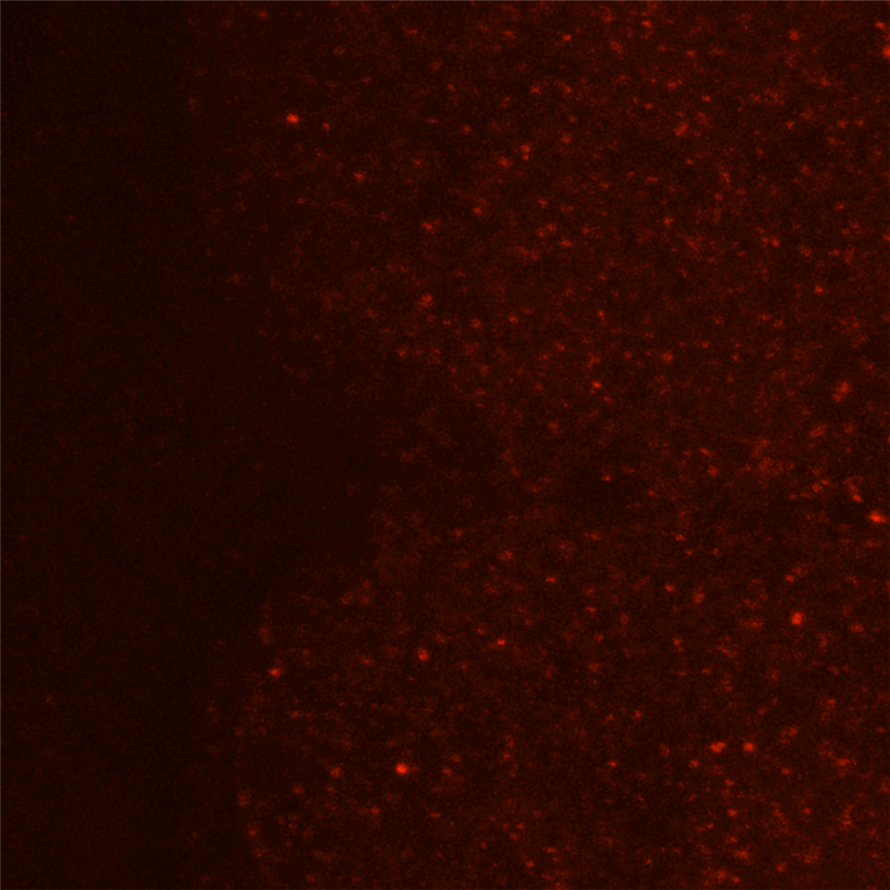

Supplement: Supplementary file 14 — Source data Fig. 3 [file 44318_2025_442_MOESM14_ESM.zip › Figure_3/Figure 3B/nanos3 Mrbm24a.tif]

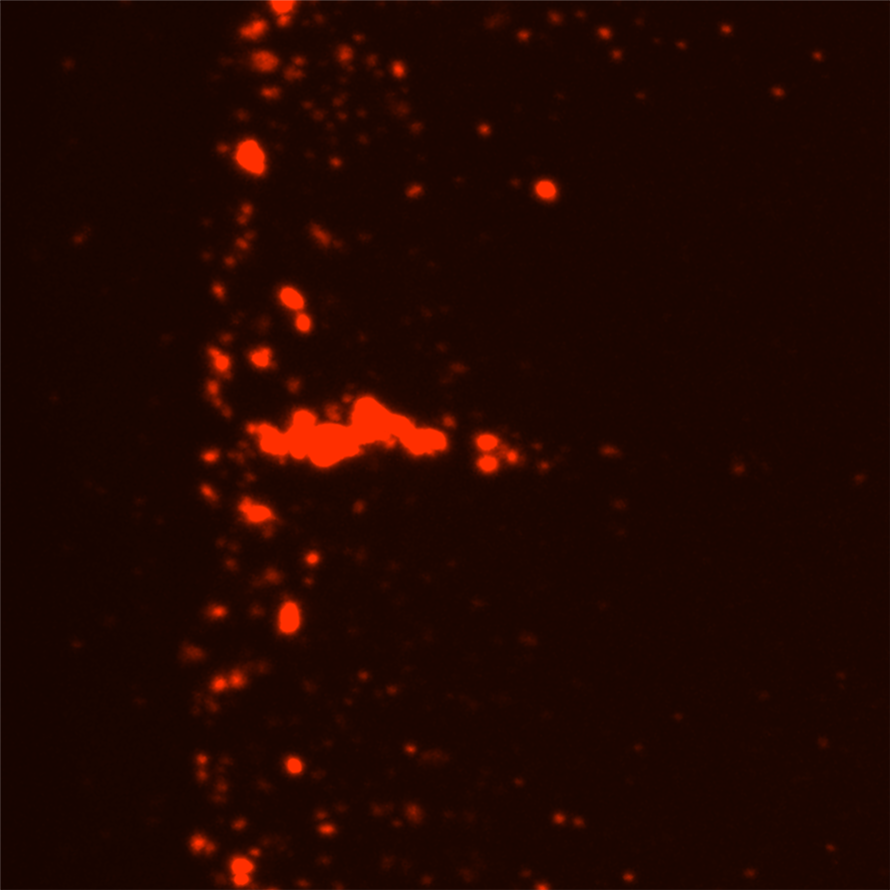

Supplement: Supplementary file 14 — Source data Fig. 3 [file 44318_2025_442_MOESM14_ESM.zip › Figure_3/Figure 3B/nanos3 sibling.tif]

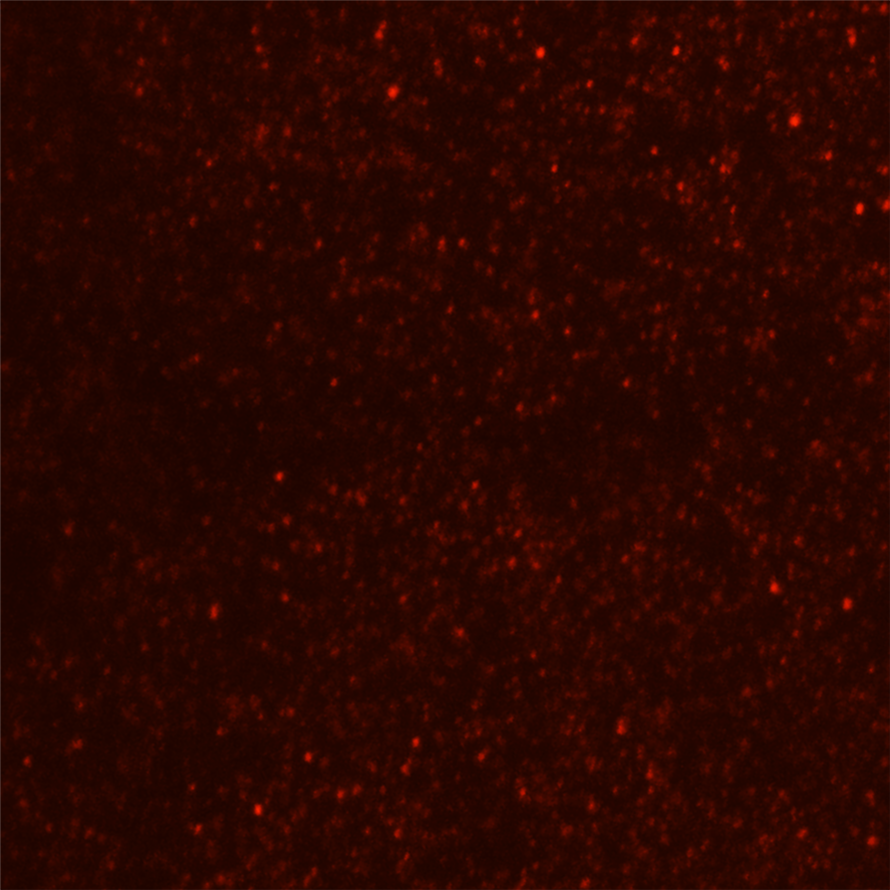

Supplement: Supplementary file 14 — Source data Fig. 3 [file 44318_2025_442_MOESM14_ESM.zip › Figure_3/Figure 3B/tdrd7a Mrbm24a .tif]

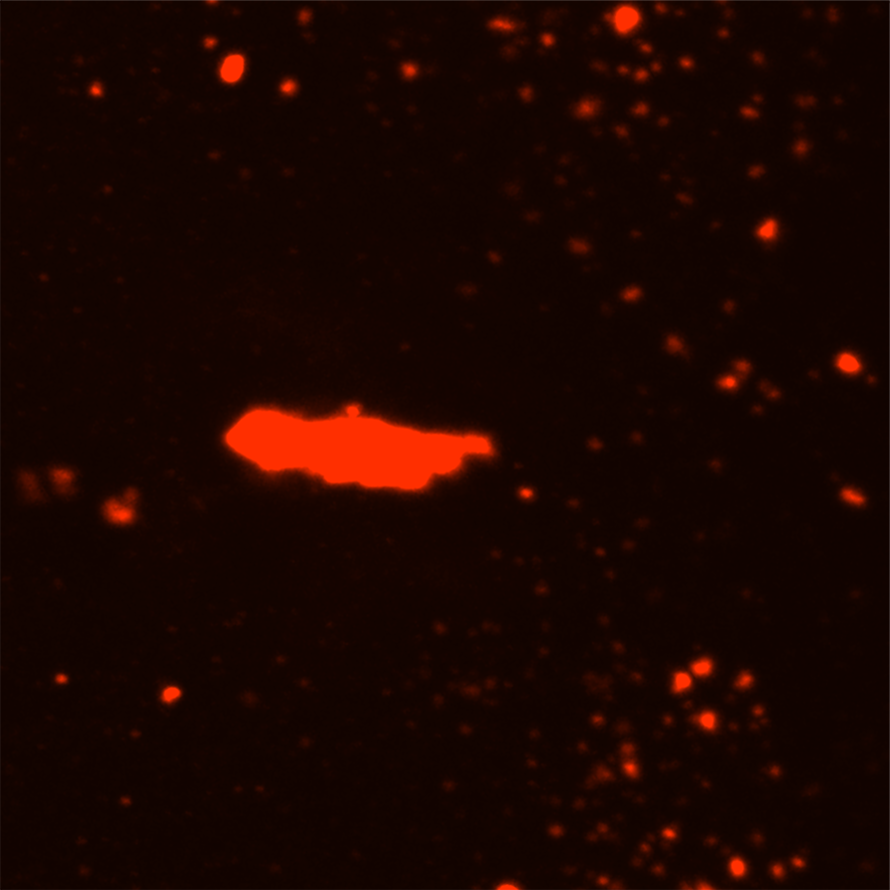

Supplement: Supplementary file 14 — Source data Fig. 3 [file 44318_2025_442_MOESM14_ESM.zip › Figure_3/Figure 3B/tdrd7a sibling.tif]

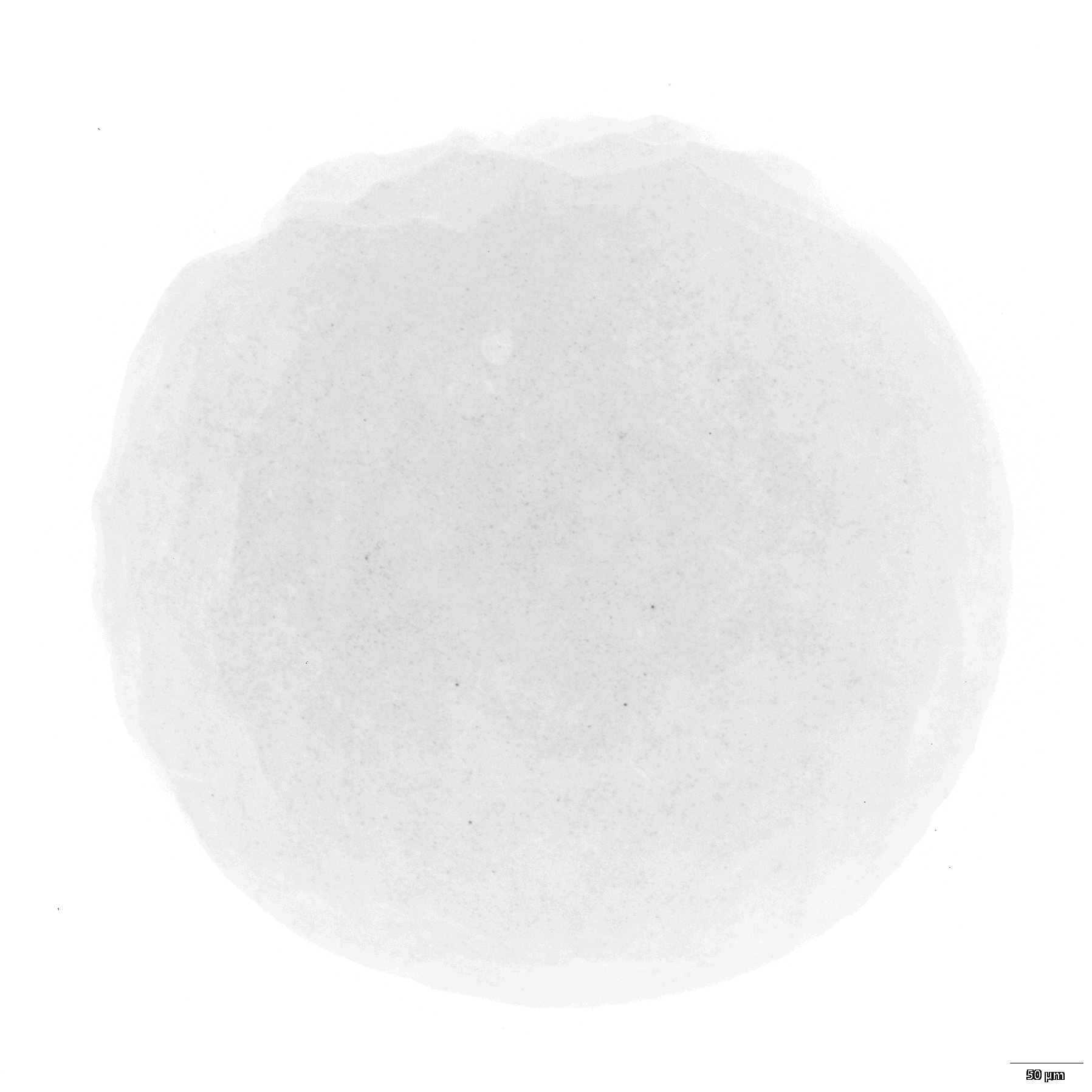

Supplement: Supplementary file 14 — Source data Fig. 3 [file 44318_2025_442_MOESM14_ESM.zip › Figure_3/Figure 3C/nanos3 Mrbm24a .tif]

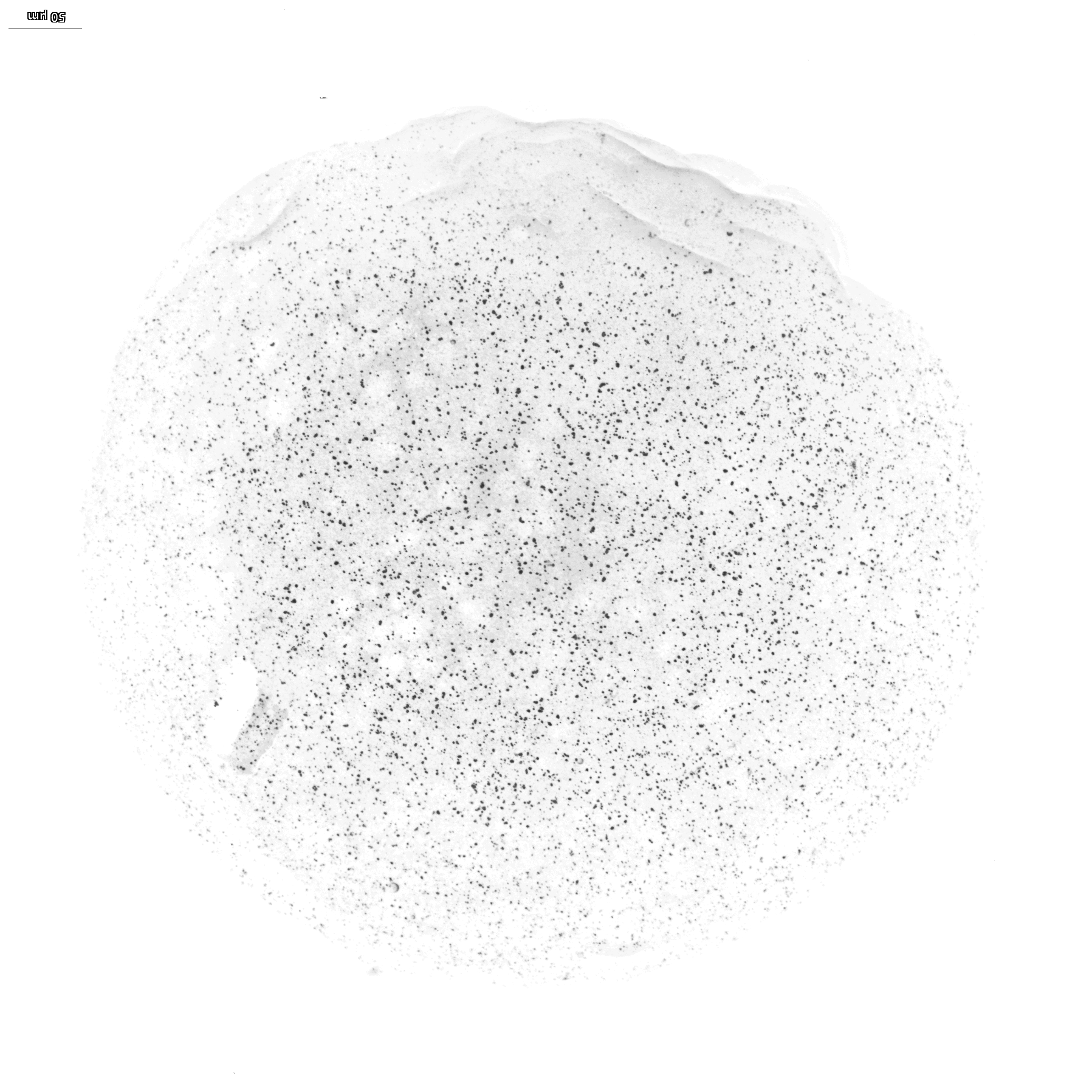

Supplement: Supplementary file 14 — Source data Fig. 3 [file 44318_2025_442_MOESM14_ESM.zip › Figure_3/Figure 3C/nanos3 sibling.tif]

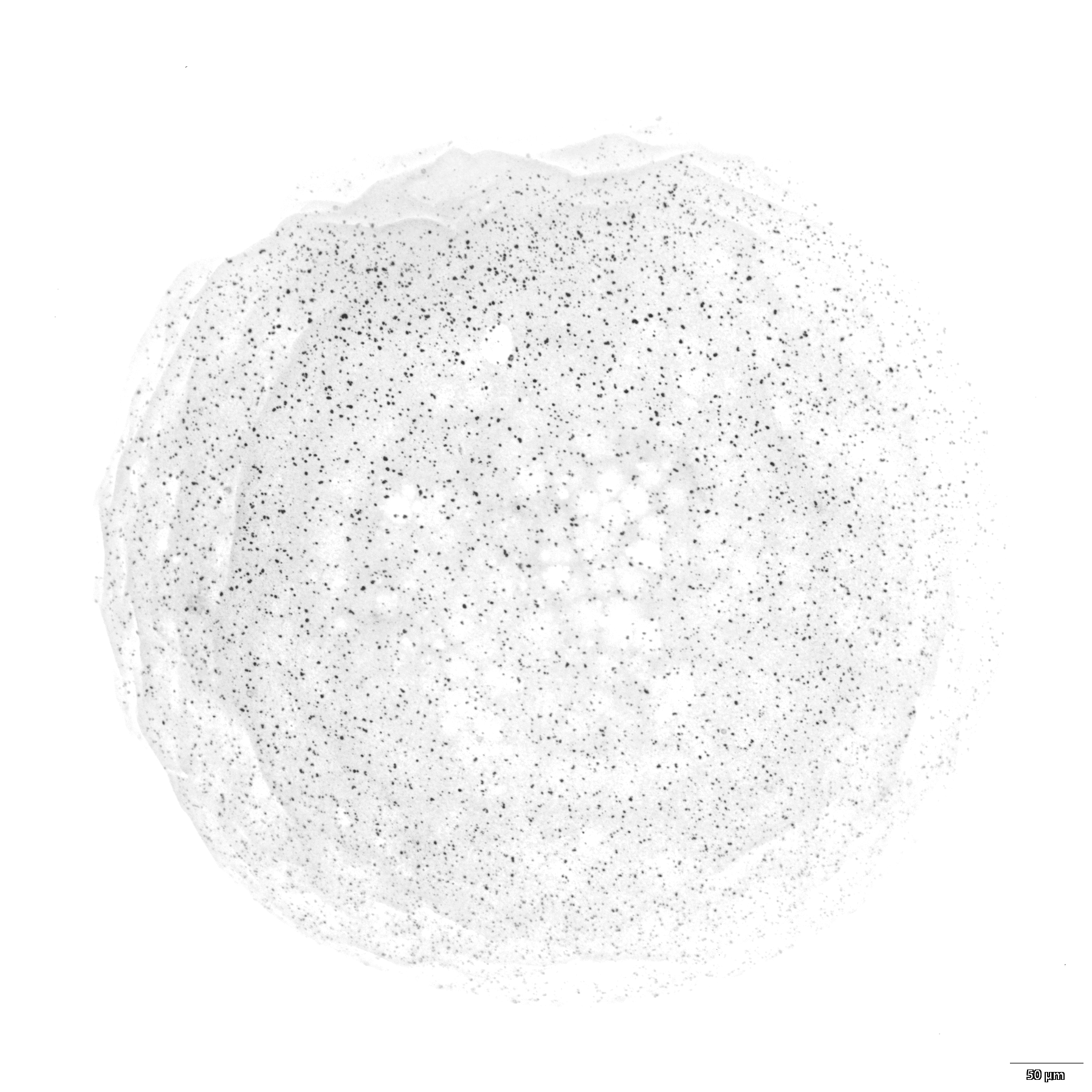

Supplement: Supplementary file 14 — Source data Fig. 3 [file 44318_2025_442_MOESM14_ESM.zip › Figure_3/Figure 3C/Piwil1 Mrbm24a .tif]

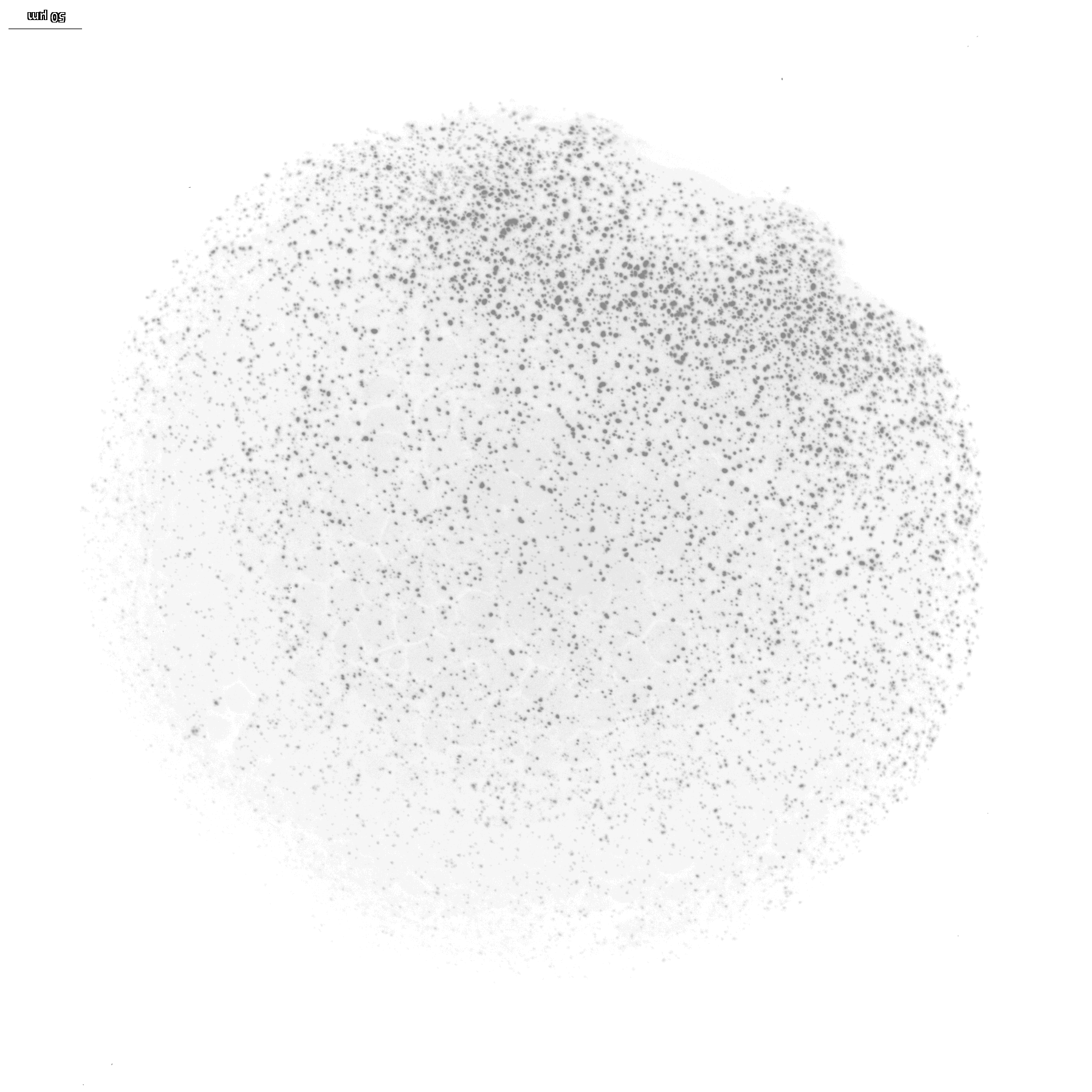

Supplement: Supplementary file 14 — Source data Fig. 3 [file 44318_2025_442_MOESM14_ESM.zip › Figure_3/Figure 3C/Piwil1 sibling .tif]

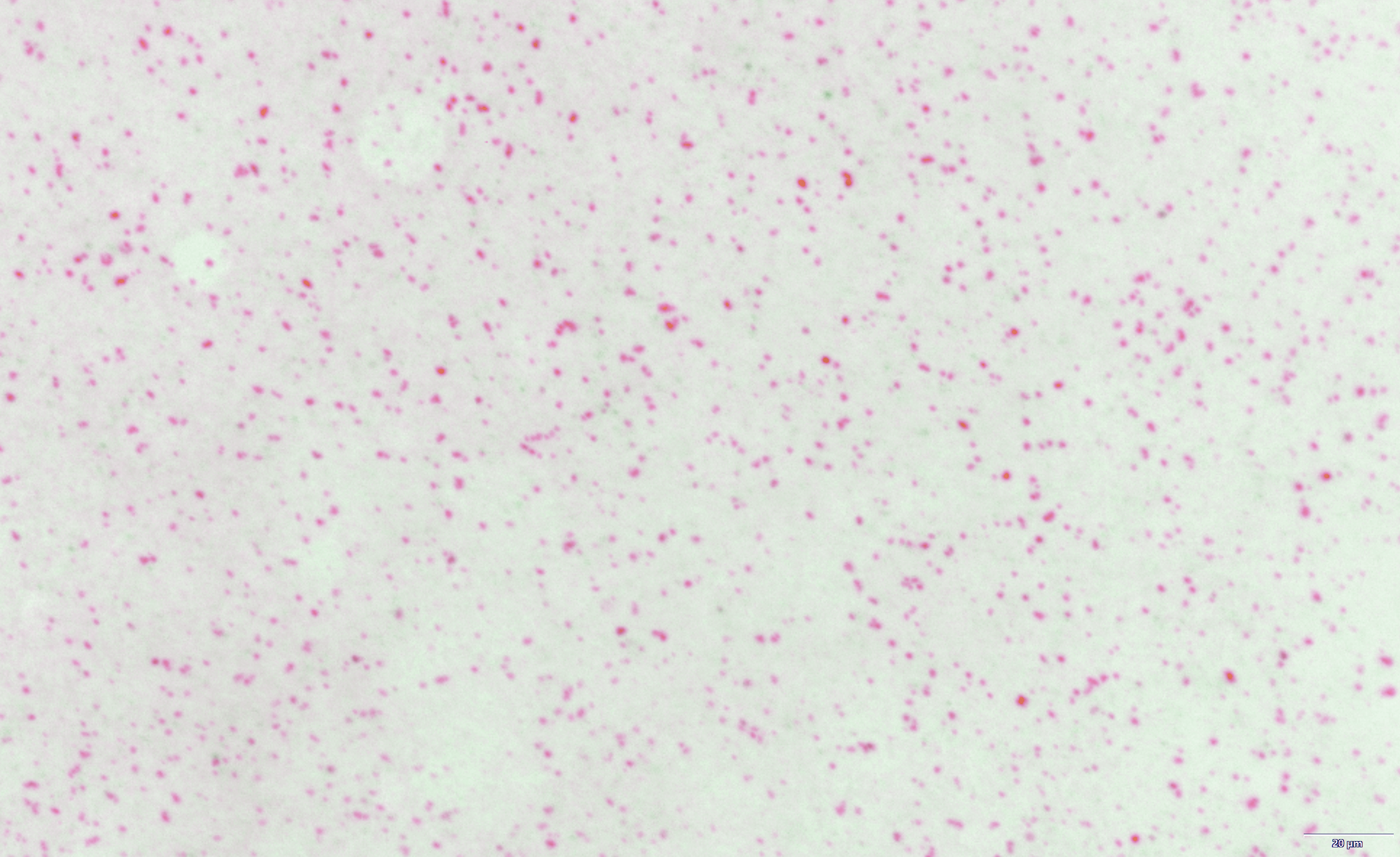

Supplement: Supplementary file 14 — Source data Fig. 3 [file 44318_2025_442_MOESM14_ESM.zip › Figure_3/Figure 3D/Mrbm24a merge.tif]

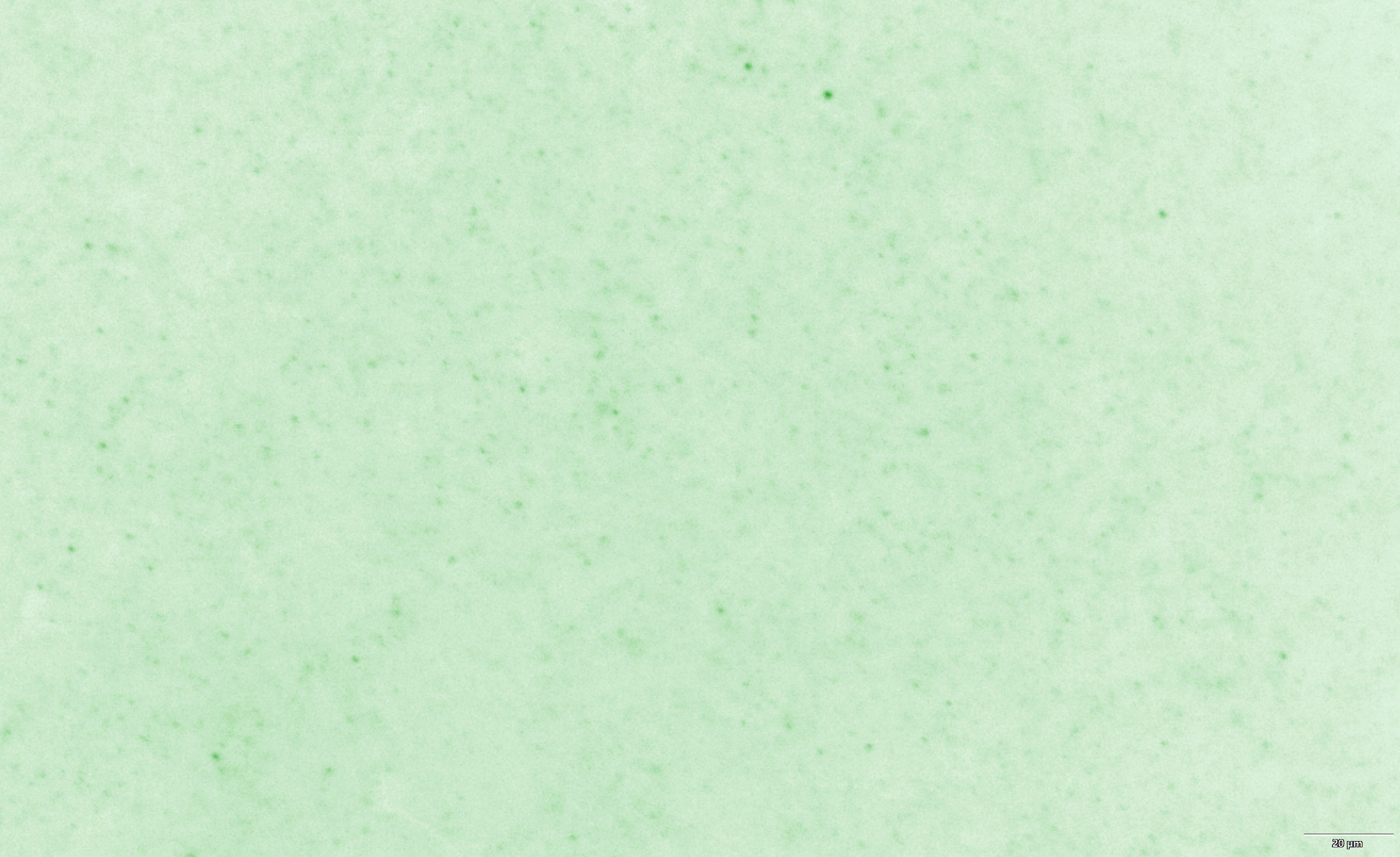

Supplement: Supplementary file 14 — Source data Fig. 3 [file 44318_2025_442_MOESM14_ESM.zip › Figure_3/Figure 3D/Mrbm24a nanos3.tif]

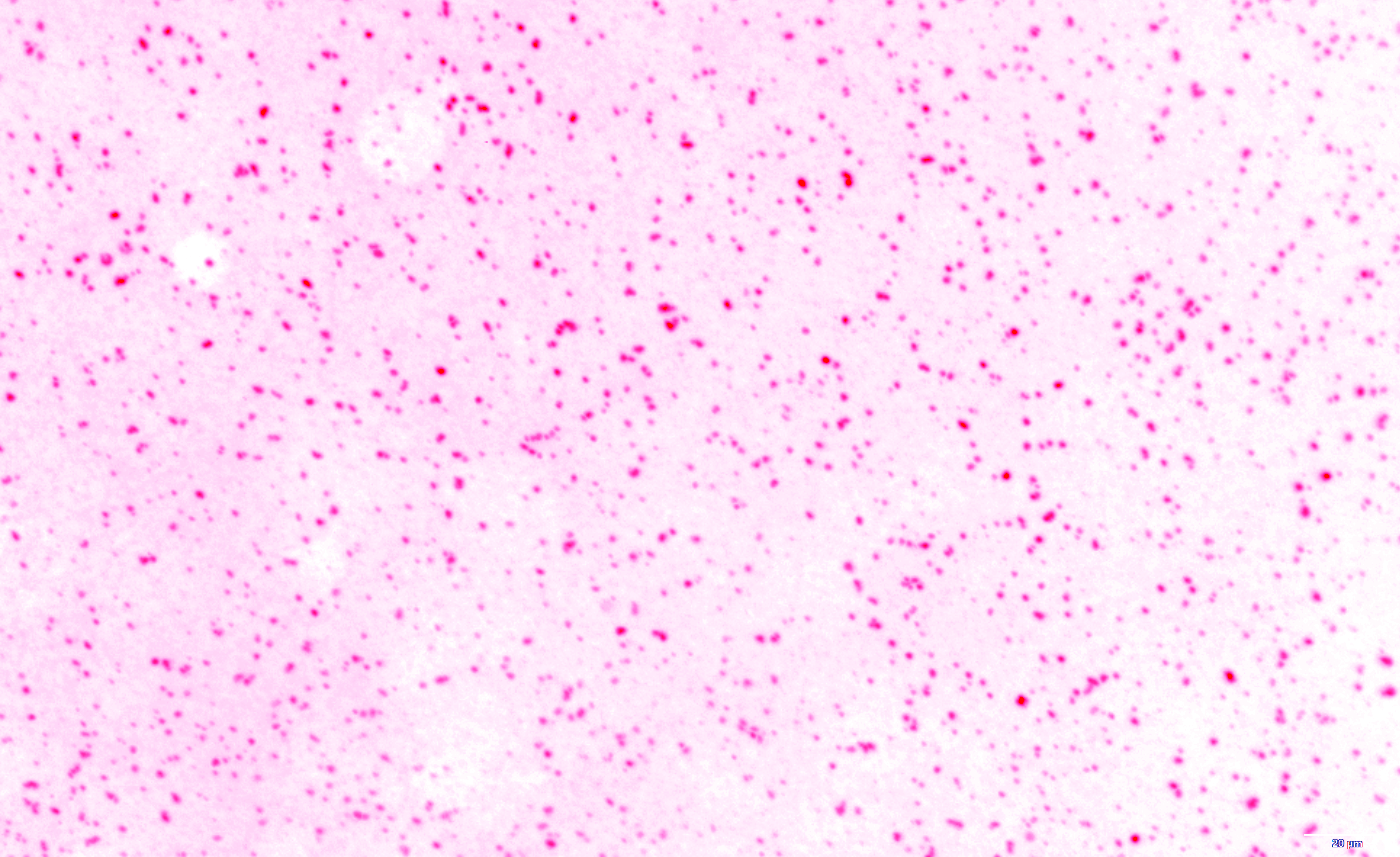

Supplement: Supplementary file 14 — Source data Fig. 3 [file 44318_2025_442_MOESM14_ESM.zip › Figure_3/Figure 3D/Mrbm24a piwil1.tif]

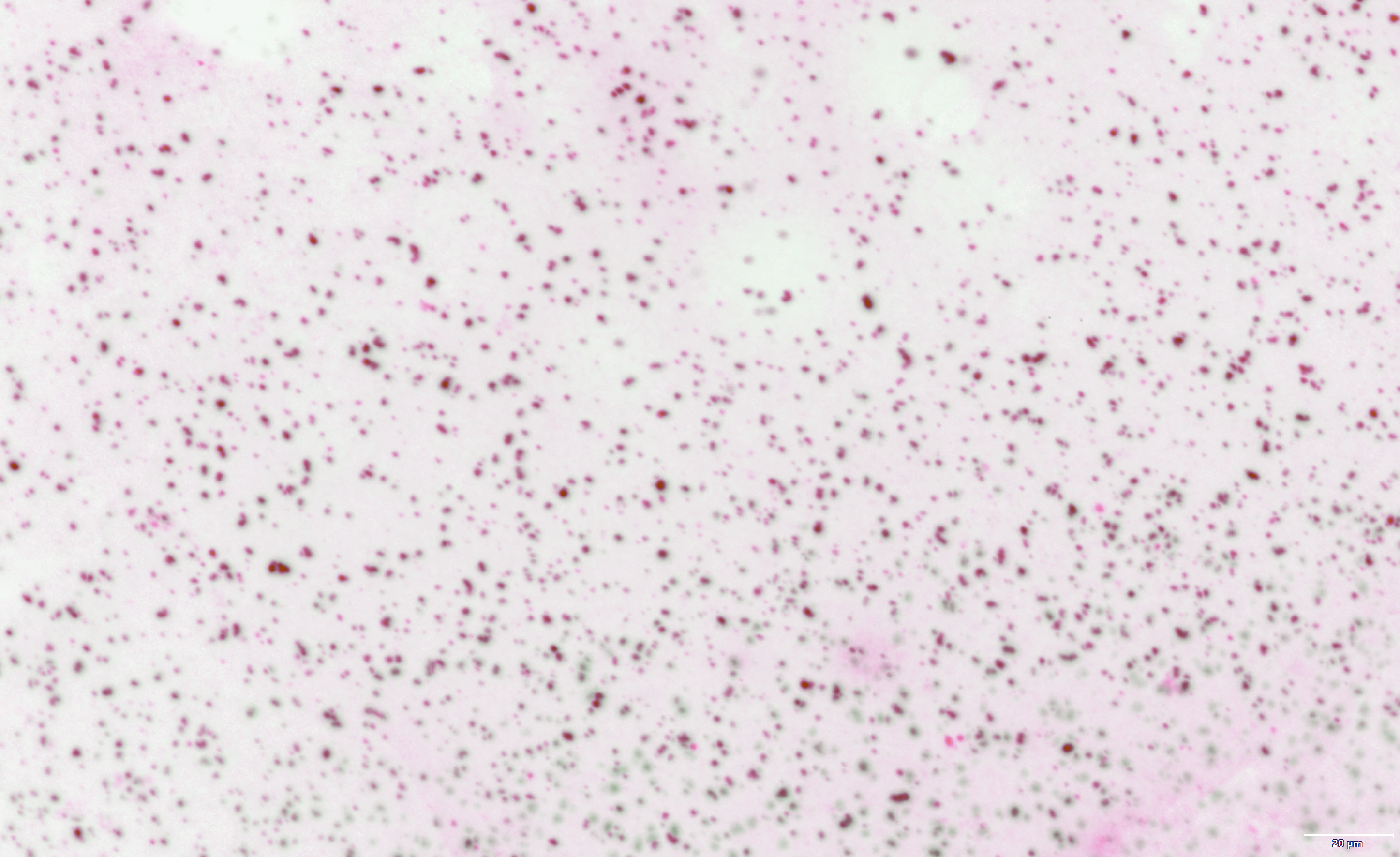

Supplement: Supplementary file 14 — Source data Fig. 3 [file 44318_2025_442_MOESM14_ESM.zip › Figure_3/Figure 3D/sibling merge.tif]

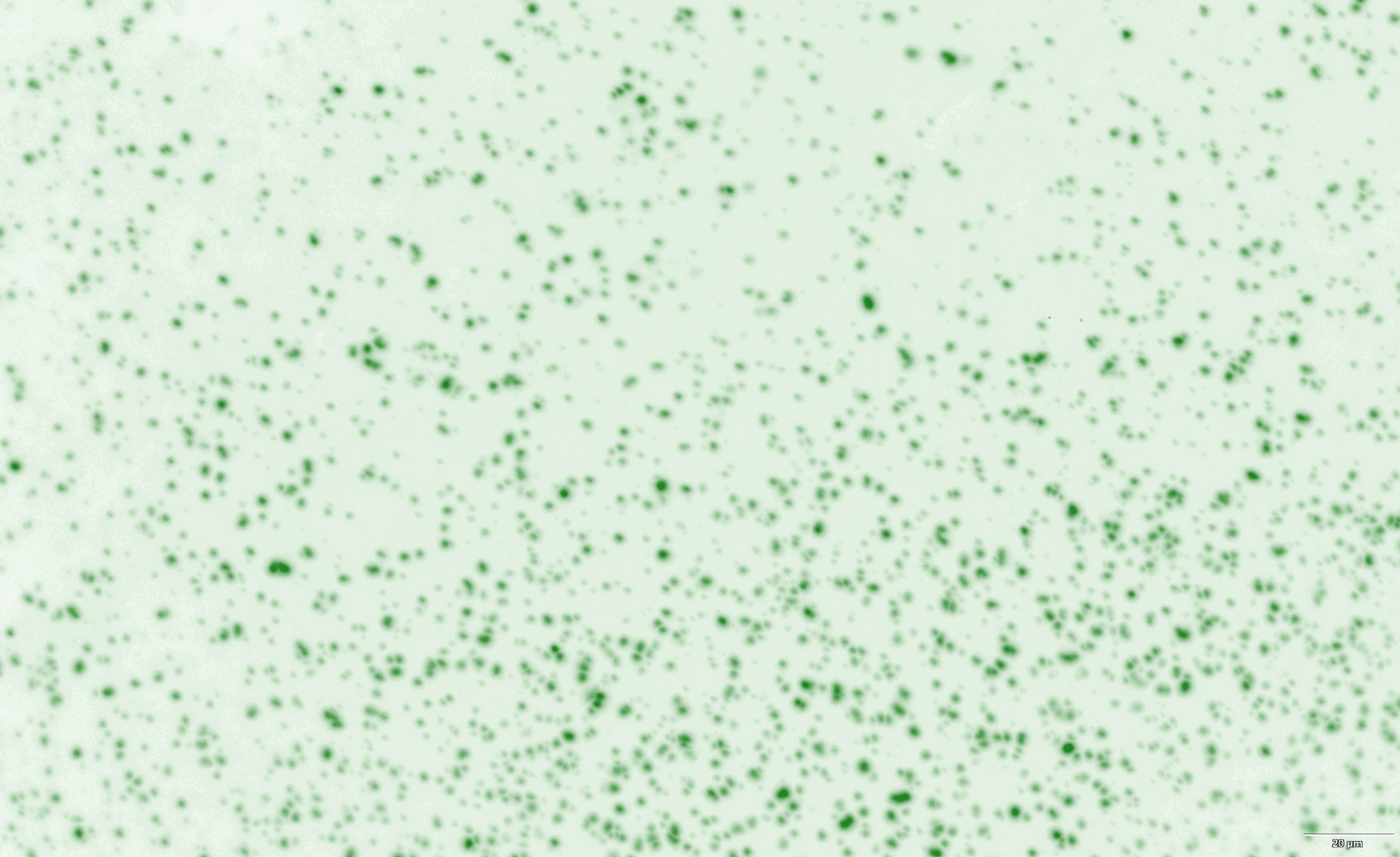

Supplement: Supplementary file 14 — Source data Fig. 3 [file 44318_2025_442_MOESM14_ESM.zip › Figure_3/Figure 3D/sibling nanos3.tif]

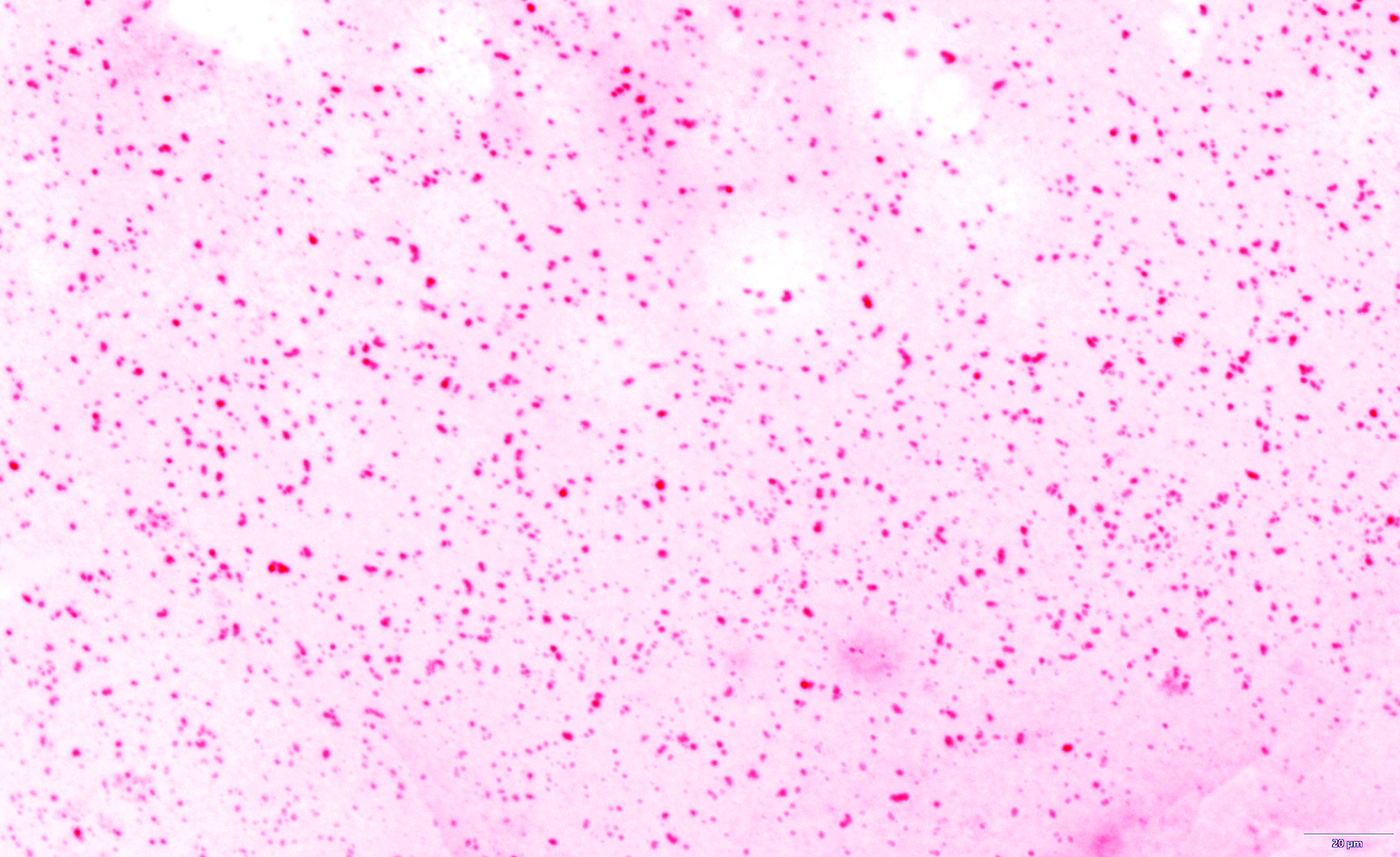

Supplement: Supplementary file 14 — Source data Fig. 3 [file 44318_2025_442_MOESM14_ESM.zip › Figure_3/Figure 3D/sibling piwil1.tif]

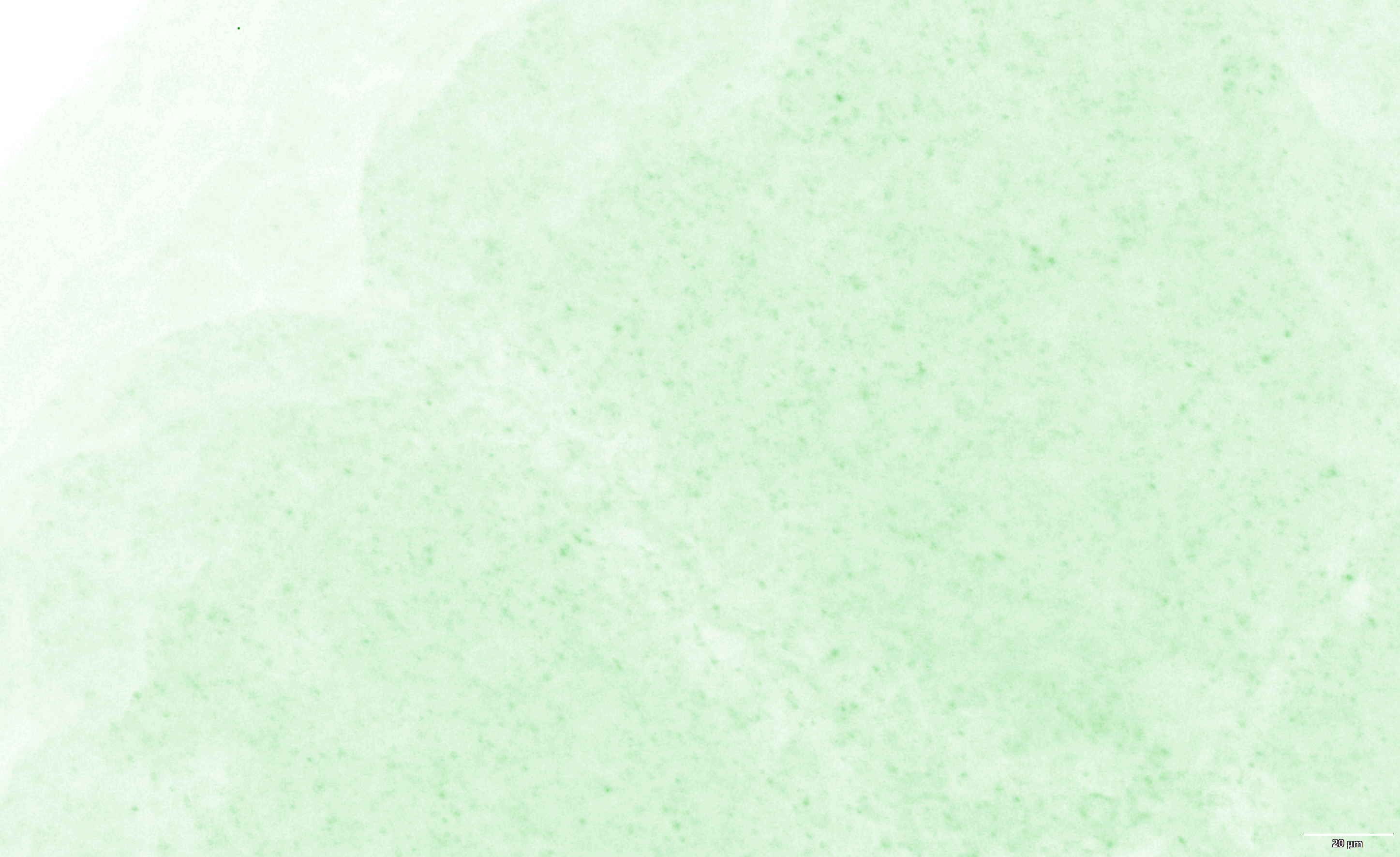

Supplement: Supplementary file 14 — Source data Fig. 3 [file 44318_2025_442_MOESM14_ESM.zip › Figure_3/Figure 3G/Mrbm24a nanos3.tif]

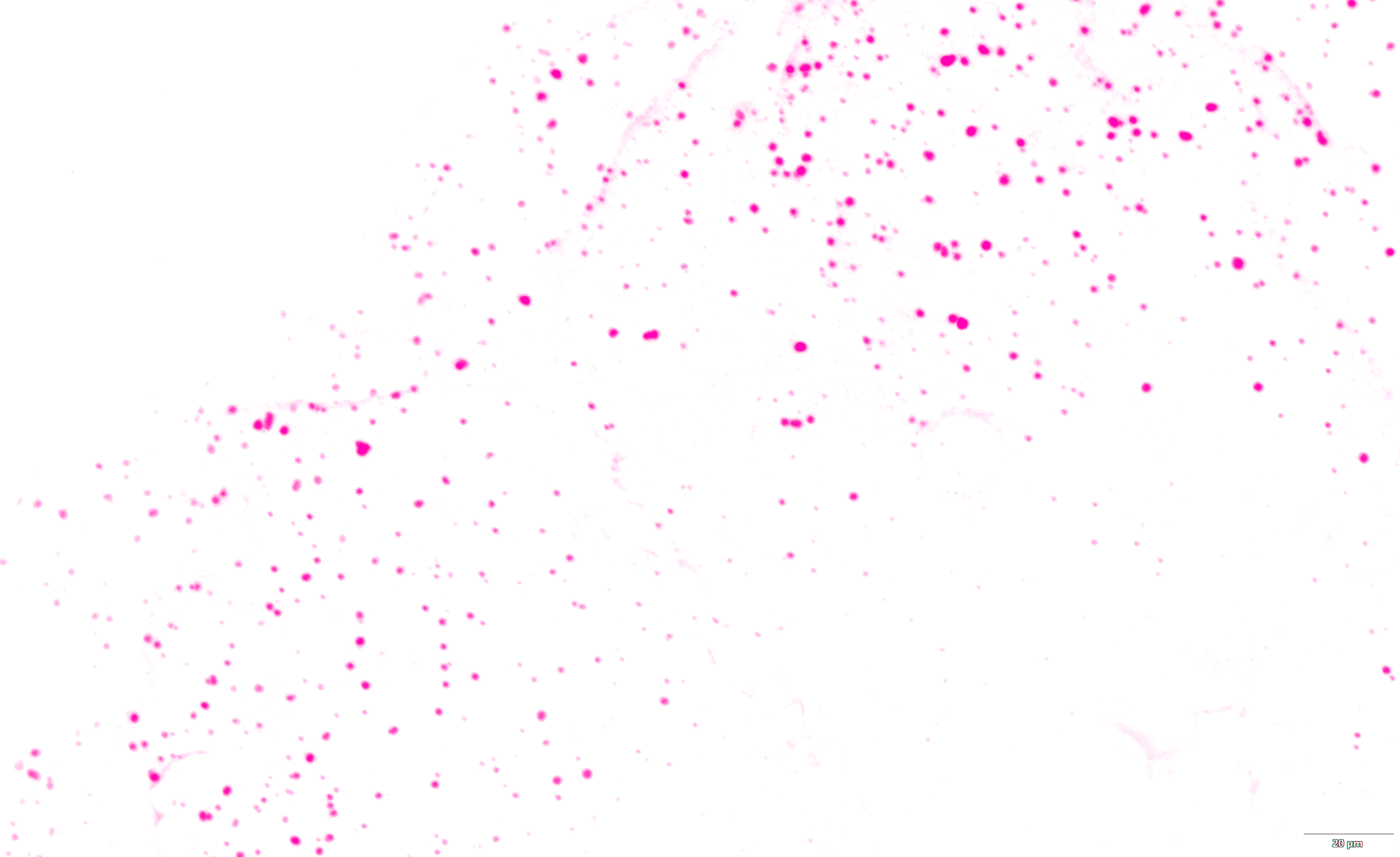

Supplement: Supplementary file 14 — Source data Fig. 3 [file 44318_2025_442_MOESM14_ESM.zip › Figure_3/Figure 3G/Mrbm24a piwil1.tif]

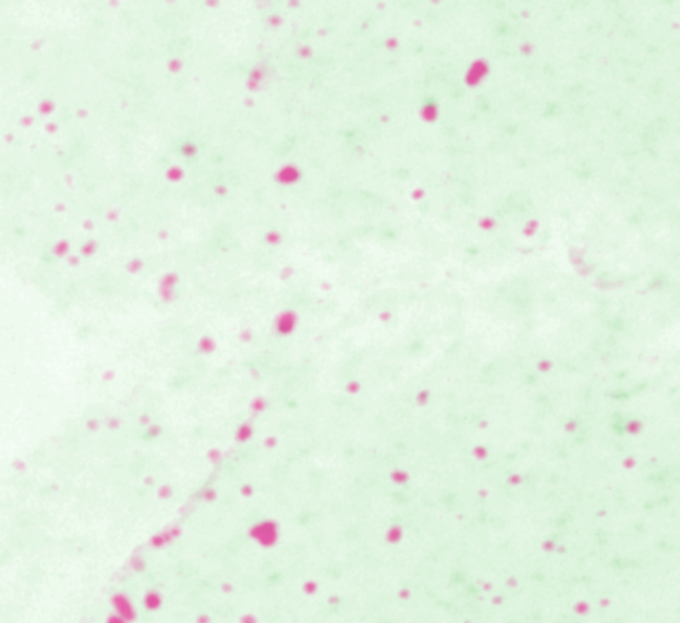

Supplement: Supplementary file 14 — Source data Fig. 3 [file 44318_2025_442_MOESM14_ESM.zip › Figure_3/Figure 3G/Mrbm24a Merge.tif]

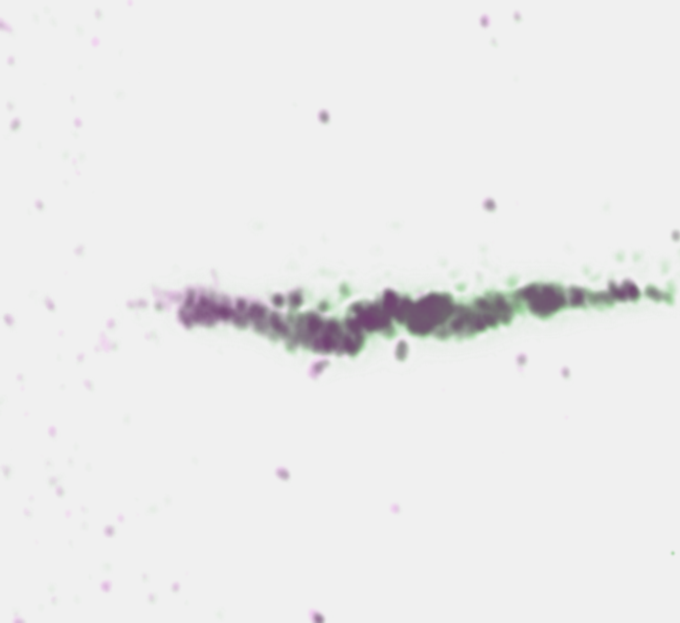

Supplement: Supplementary file 14 — Source data Fig. 3 [file 44318_2025_442_MOESM14_ESM.zip › Figure_3/Figure 3G/sibling Merge.tif]
