# Supplementary material for: Rbm24a dictates mRNA recruitment for germ granule assembly in zebrafish
Source: EMBO J. 2025 Apr 25;44(11):3121–49. doi: 10.1038/s44318-025-00442-z (PMC12130248; doi:10.1038/s44318-025-00442-z)
Supplement: Supplementary file 4 — Movie EV1 [file 44318_2025_442_MOESM4_ESM.zip › Movie EV1/Legend for Movie EV1.docx]

**Movie EV1: Rbm24a-GFP distribution and dynamics during early cleavage of a *rbm24a-GFP* KI embryo.**

The embryo is positioned with the animal pole to the viewer under a light-sheet microscope.
