# Supplementary material for: Rbm24a dictates mRNA recruitment for germ granule assembly in zebrafish
Source: EMBO J. 2025 Apr 25;44(11):3121–49. doi: 10.1038/s44318-025-00442-z (PMC12130248; doi:10.1038/s44318-025-00442-z)
Supplement: Supplementary file 5 — Movie EV2 [file 44318_2025_442_MOESM5_ESM.zip › Movie EV2/Legend for Movie EV2.docx]

**Movie EV2: Germ granule dynamics labelled by Rbm24a-GFP during cleavage and blastula stages.**

The *rbm24a-GFP* KI embryo was mounted with the animal pole towards the objective lens of a spinning disc confocal microscopy.
