# Supplementary material for: Rbm24a dictates mRNA recruitment for germ granule assembly in zebrafish
Source: EMBO J. 2025 Apr 25;44(11):3121–49. doi: 10.1038/s44318-025-00442-z (PMC12130248; doi:10.1038/s44318-025-00442-z)
Supplement: Supplementary file 6 — Movie EV3 [file 44318_2025_442_MOESM6_ESM.zip › Movie EV3/Legend for Movie EV3.docx]

**Movie EV3: 3D view of germ granule dynamics during early development.**

3D video of *rbm24a-GFP* KI embryos was acquired under a light-sheet microscope. The left and right parts of the video represent the animal pole view and lateral view of the same embryo.
