# Supplementary material for: Rbm24a dictates mRNA recruitment for germ granule assembly in zebrafish
Source: EMBO J. 2025 Apr 25;44(11):3121–49. doi: 10.1038/s44318-025-00442-z (PMC12130248; doi:10.1038/s44318-025-00442-z)
Supplement: Supplementary file 7 — Movie EV4 [file 44318_2025_442_MOESM7_ESM.zip › Movie EV4/Legend for Movie EV4.docx]

**Movie EV4: Live imaging of Buc-GFP recruitment to the germ plasm condensates in wild-type and M*rbm24a* embryos.**

The Buc-GFP protein was translated from injected *buc-gfp* mRNA.
