# Supplementary material for: Rbm24a dictates mRNA recruitment for germ granule assembly in zebrafish
Source: EMBO J. 2025 Apr 25;44(11):3121–49. doi: 10.1038/s44318-025-00442-z (PMC12130248; doi:10.1038/s44318-025-00442-z)
Supplement: Supplementary file 8 — Movie EV5 [file 44318_2025_442_MOESM8_ESM.zip › Movie EV5/Legend for Movie EV5.docx]

**Movie EV5: Germ granules fail to converge to cleavage furrows in M*rbm24a* embryo.**

Germ granules were labelled by transgenic expression of Buc-GFP.
