# Supplementary material for: Rbm24a dictates mRNA recruitment for germ granule assembly in zebrafish
Source: EMBO J. 2025 Apr 25;44(11):3121–49. doi: 10.1038/s44318-025-00442-z (PMC12130248; doi:10.1038/s44318-025-00442-z)
Supplement: Supplementary file 9 — Movie EV6 [file 44318_2025_442_MOESM9_ESM.zip › Movie EV6/Legend for Movie EV6.docx]

**Movie EV6: Trajectory tracking of germ granules during the formation of second cleavage furrow in wild-type and M*rbm24a*.**

Germ granules were labelled by transgenic expression of Buc-GFP.
