# Supplementary material for: Rbm24a dictates mRNA recruitment for germ granule assembly in zebrafish
Source: EMBO J. 2025 Apr 25;44(11):3121–49. doi: 10.1038/s44318-025-00442-z (PMC12130248; doi:10.1038/s44318-025-00442-z)
Supplement: Supplementary file 10 — Movie EV7 [file 44318_2025_442_MOESM10_ESM.zip › Movie EV7/Legend for Movie EV7.docx]

**Movie EV7: Germ granules fail to undergo directed transport toward the furrows after kinesin antibody injection.**

Germ plasm particles were visualized by endogenous expression of Rbm24a-GFP in the *rbm24a-GFP* KI background.
