# Supplementary material for: Rbm24a dictates mRNA recruitment for germ granule assembly in zebrafish
Source: EMBO J. 2025 Apr 25;44(11):3121–49. doi: 10.1038/s44318-025-00442-z (PMC12130248; doi:10.1038/s44318-025-00442-z)
Supplement: Supplementary file 11 — Movie EV8 [file 44318_2025_442_MOESM11_ESM.zip › Movie EV8/Legend for Movie EV8.docx]

**Movie EV8: Trajectory tracking of germ granules during the formation of the second cleavage furrow in uninjected and kinesin-antibody-injected *rbm24a-GFP KI* embryos.**

Germ granules were visualized by endogenous expression of Rbm24a-GFP in the *rbm24a-GFP* KI background.
