# Supplementary figures and images for: Rbm24a dictates mRNA recruitment for germ granule assembly in zebrafish (part 2 of 3)
Source: EMBO J. 2025 Apr 25;44(11):3121–49. doi: 10.1038/s44318-025-00442-z (PMC12130248; doi:10.1038/s44318-025-00442-z)

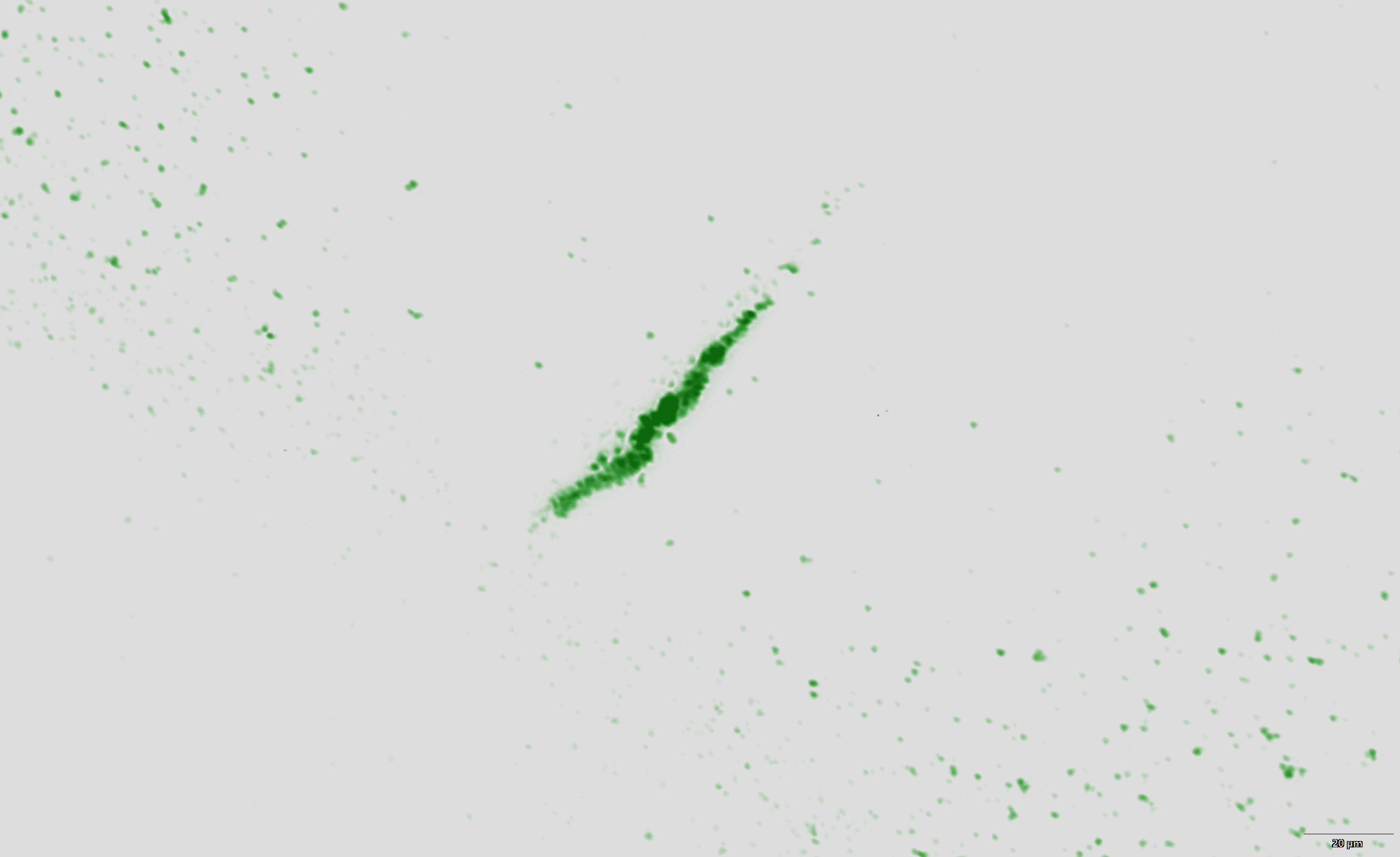

Supplement: Supplementary file 14 — Source data Fig. 3 [file 44318_2025_442_MOESM14_ESM.zip › Figure_3/Figure 3G/sibling nanos3.tif]

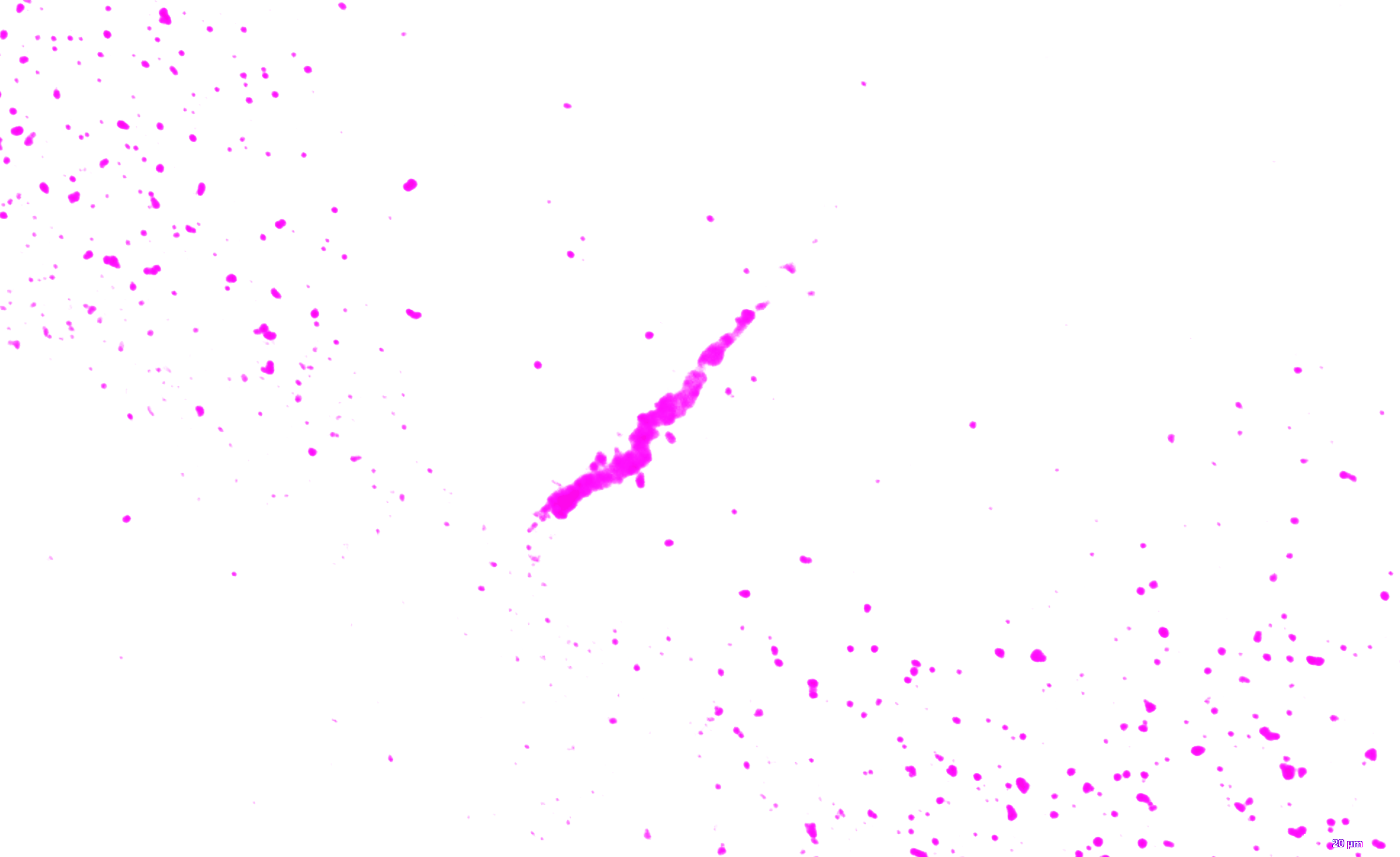

Supplement: Supplementary file 14 — Source data Fig. 3 [file 44318_2025_442_MOESM14_ESM.zip › Figure_3/Figure 3G/sibling piwil1.tif]

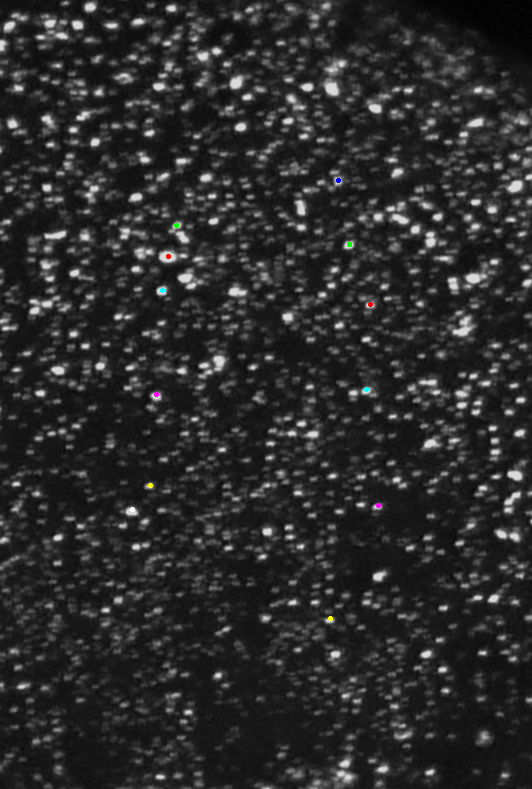

Supplement: Supplementary file 15 — Source data Fig. 4 [file 44318_2025_442_MOESM15_ESM.zip › Figure_4/Figure 4A/Figure 4A.tif]

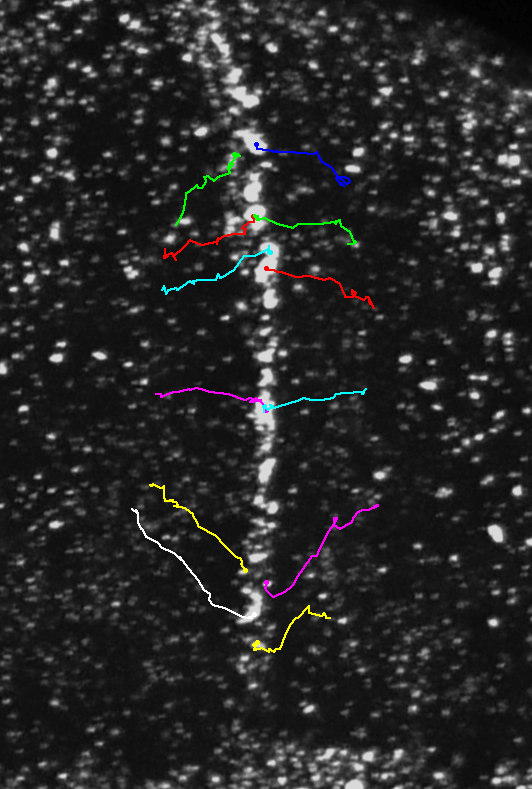

Supplement: Supplementary file 15 — Source data Fig. 4 [file 44318_2025_442_MOESM15_ESM.zip › Figure_4/Figure 4B/Figure 4B.tif]

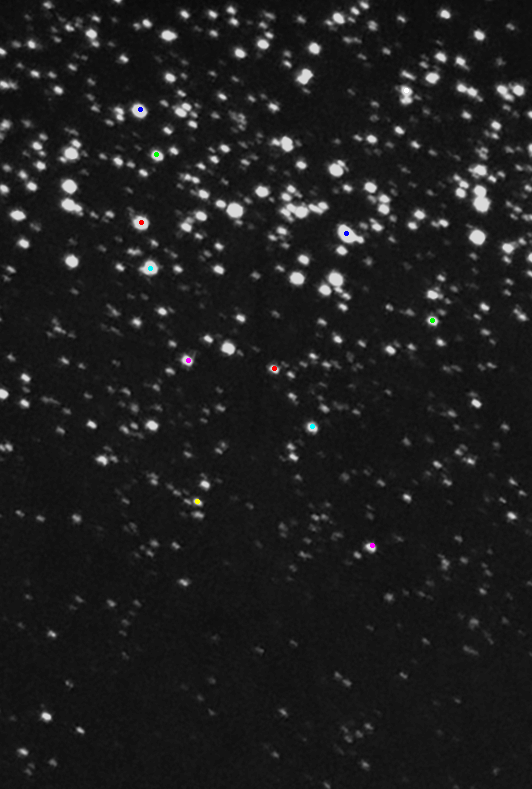

Supplement: Supplementary file 15 — Source data Fig. 4 [file 44318_2025_442_MOESM15_ESM.zip › Figure_4/Figure 4C/Figure 4C.tif]

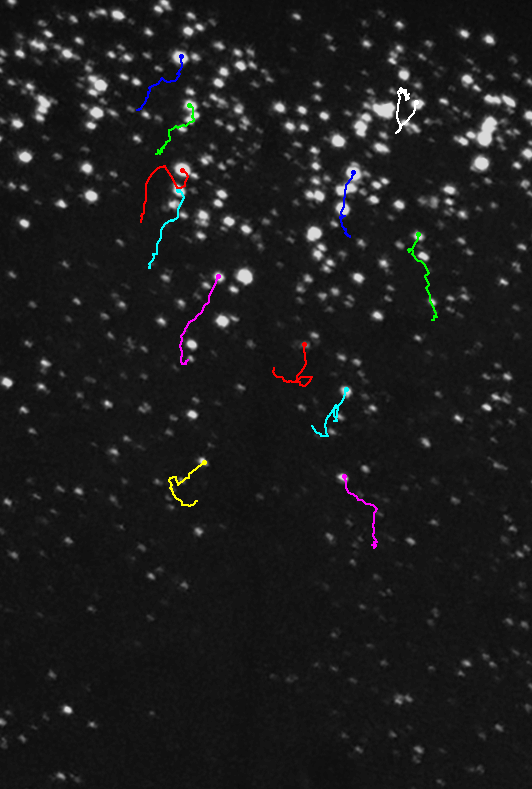

Supplement: Supplementary file 15 — Source data Fig. 4 [file 44318_2025_442_MOESM15_ESM.zip › Figure_4/Figure 4D/Figure 4D.tif]

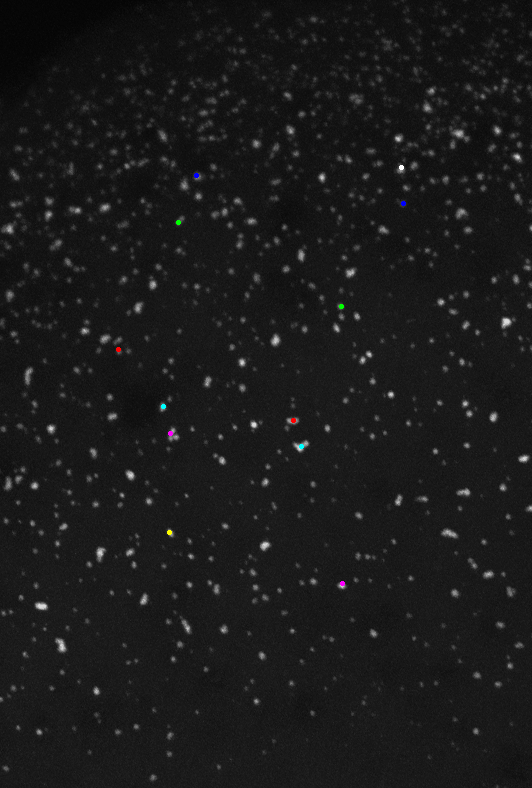

Supplement: Supplementary file 15 — Source data Fig. 4 [file 44318_2025_442_MOESM15_ESM.zip › Figure_4/Figure 4G/Figure 4G.tif]

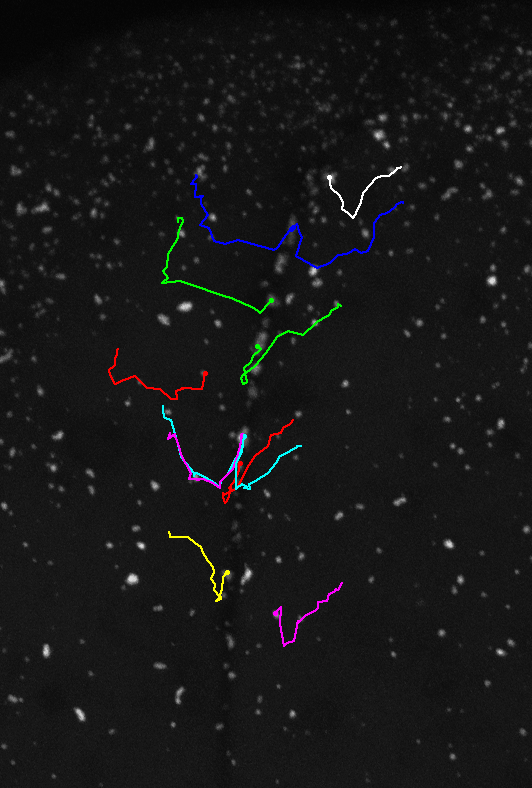

Supplement: Supplementary file 15 — Source data Fig. 4 [file 44318_2025_442_MOESM15_ESM.zip › Figure_4/Figure 4H/Figure 4H.tif]

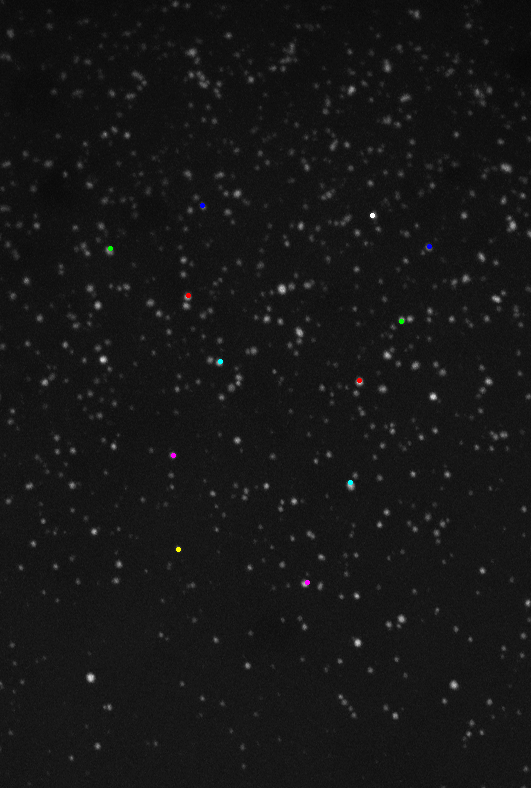

Supplement: Supplementary file 15 — Source data Fig. 4 [file 44318_2025_442_MOESM15_ESM.zip › Figure_4/Figure 4I/Figure 4I .tif]

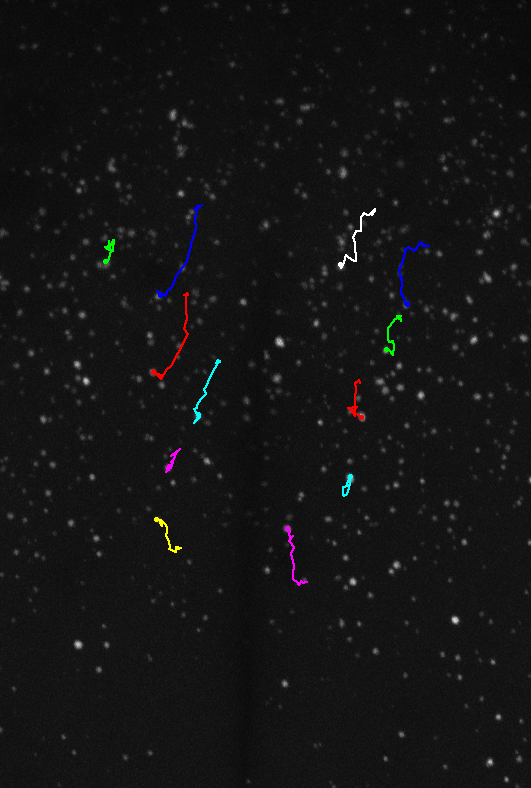

Supplement: Supplementary file 15 — Source data Fig. 4 [file 44318_2025_442_MOESM15_ESM.zip › Figure_4/Figure 4J/Figure 4J.tif]

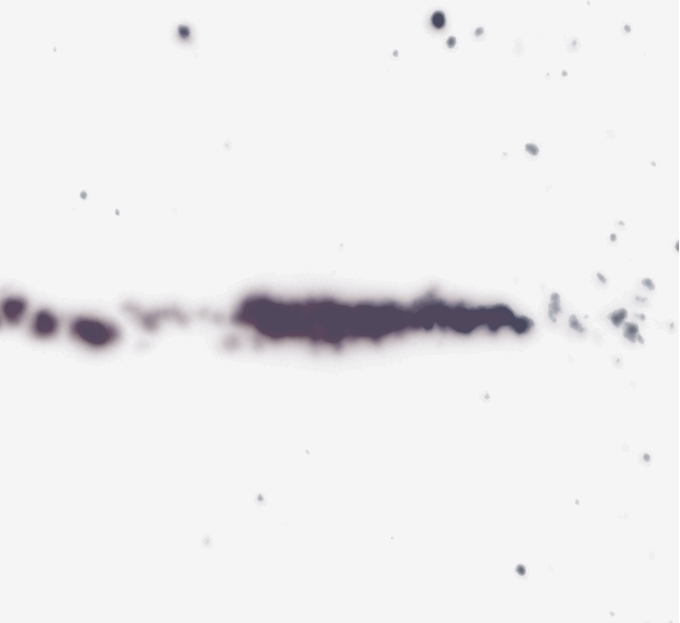

Supplement: Supplementary file 15 — Source data Fig. 4 [file 44318_2025_442_MOESM15_ESM.zip › Figure_4/Figure 4M/WT merge.tif]

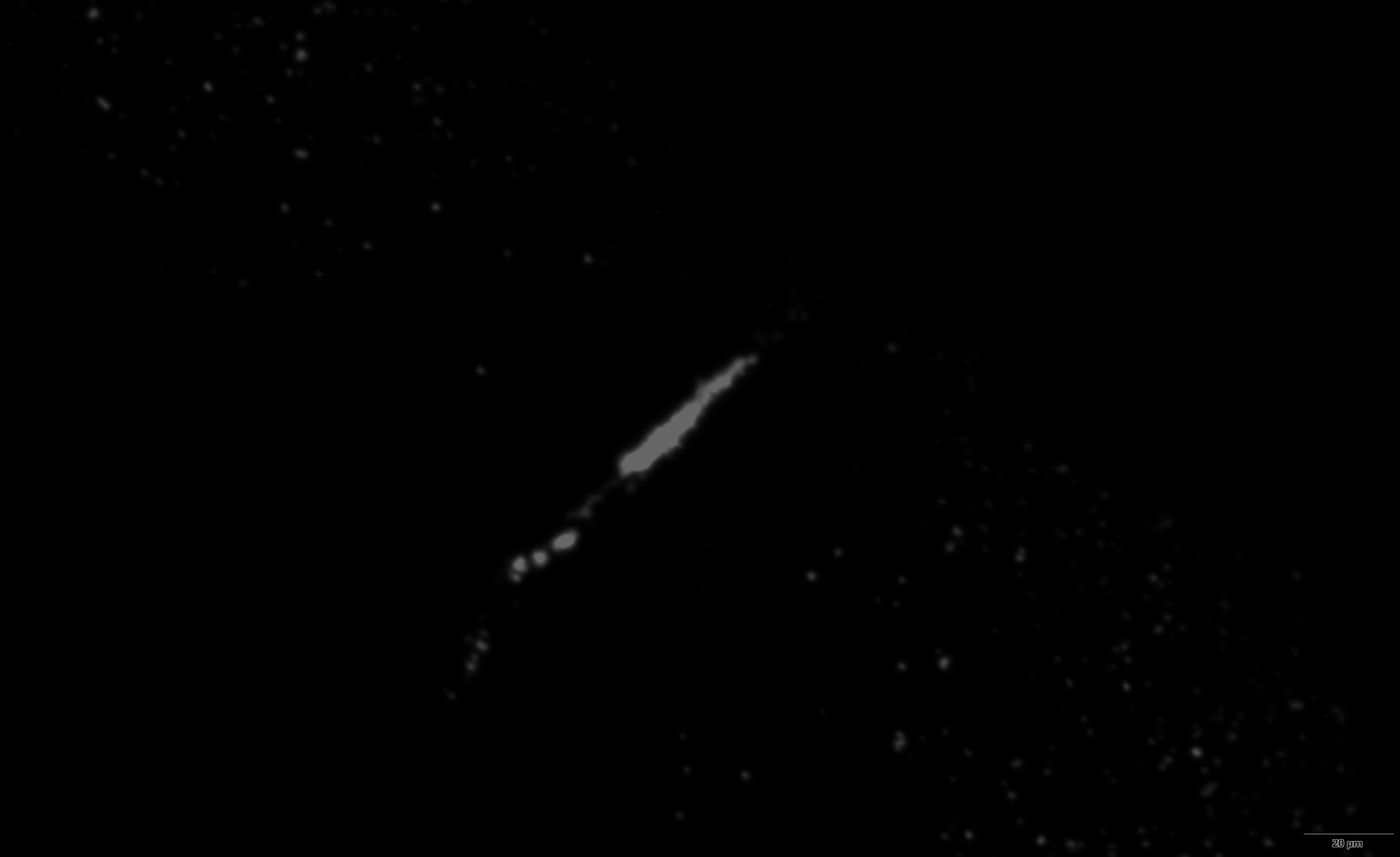

Supplement: Supplementary file 15 — Source data Fig. 4 [file 44318_2025_442_MOESM15_ESM.zip › Figure_4/Figure 4M/WT un nanos3.tif]

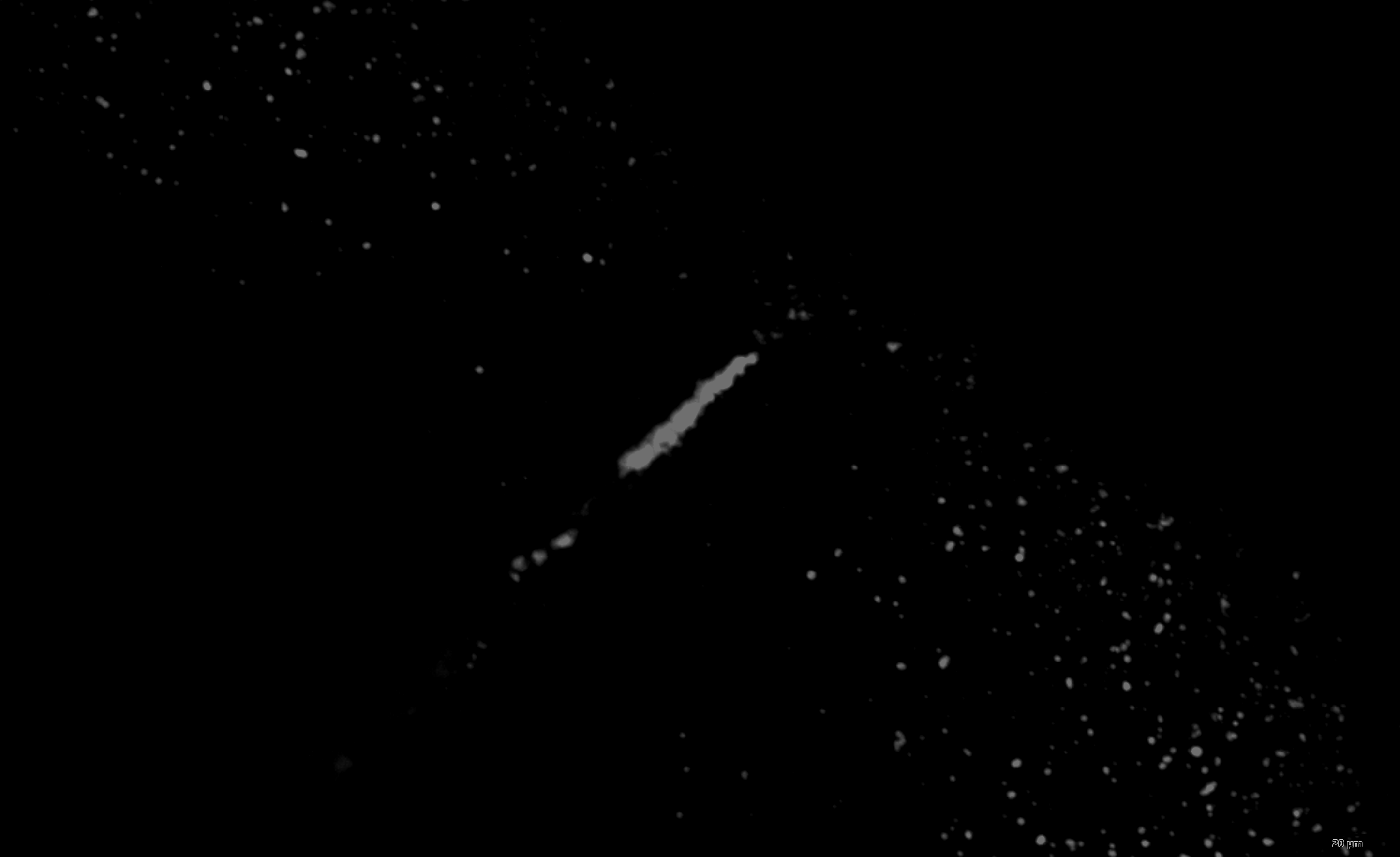

Supplement: Supplementary file 15 — Source data Fig. 4 [file 44318_2025_442_MOESM15_ESM.zip › Figure_4/Figure 4M/WT un Piwil1.tif]

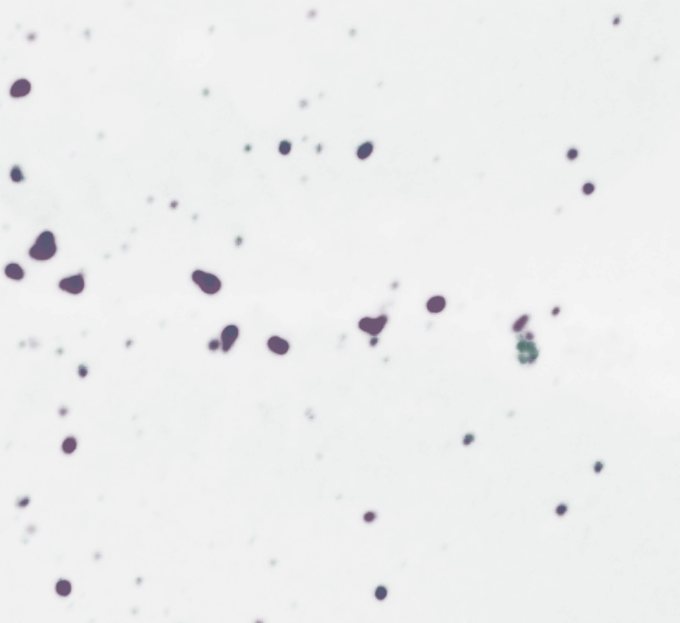

Supplement: Supplementary file 15 — Source data Fig. 4 [file 44318_2025_442_MOESM15_ESM.zip › Figure_4/Figure 4N/wt inj merge.tif]

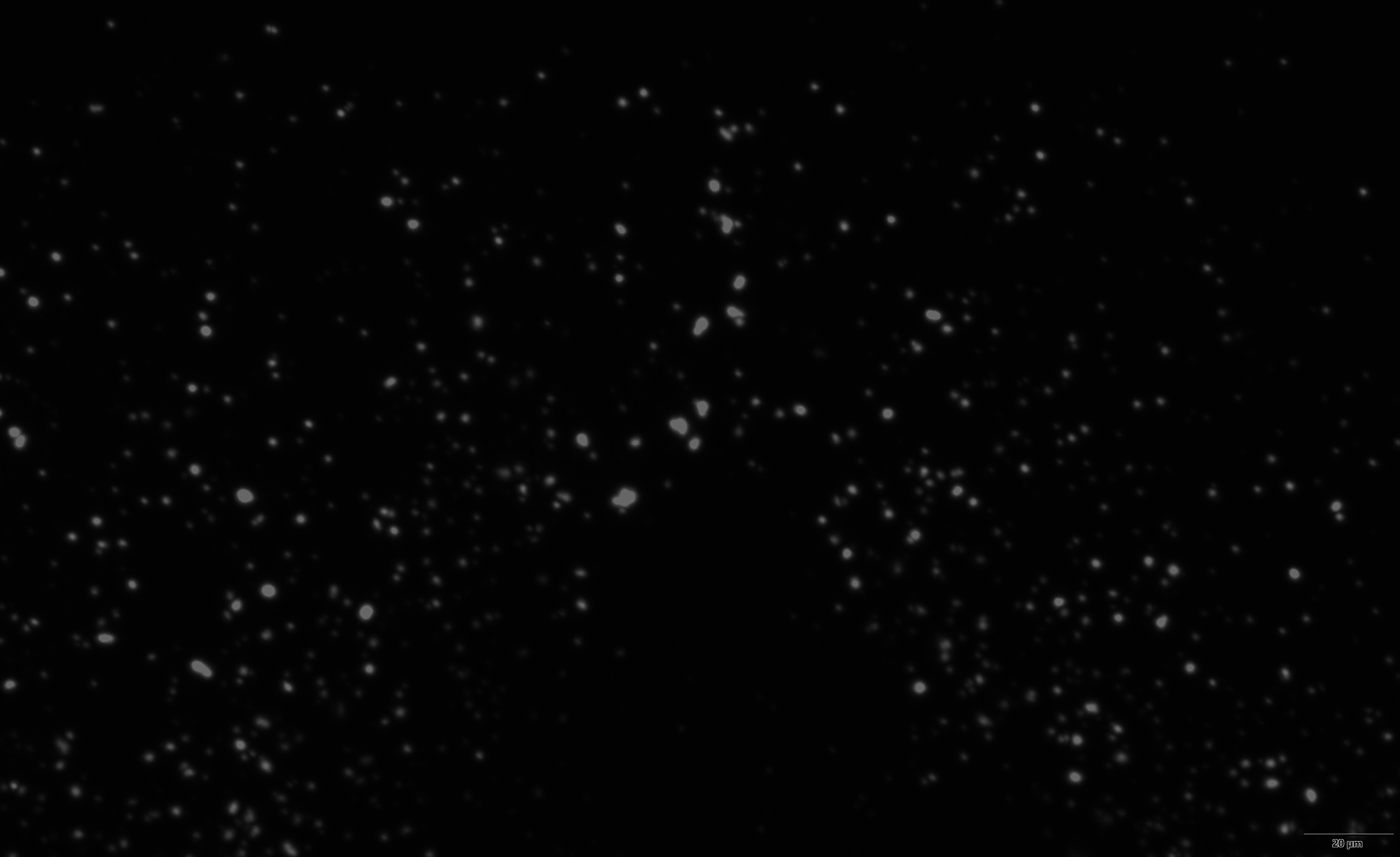

Supplement: Supplementary file 15 — Source data Fig. 4 [file 44318_2025_442_MOESM15_ESM.zip › Figure_4/Figure 4N/wt inj nanos3.tif]

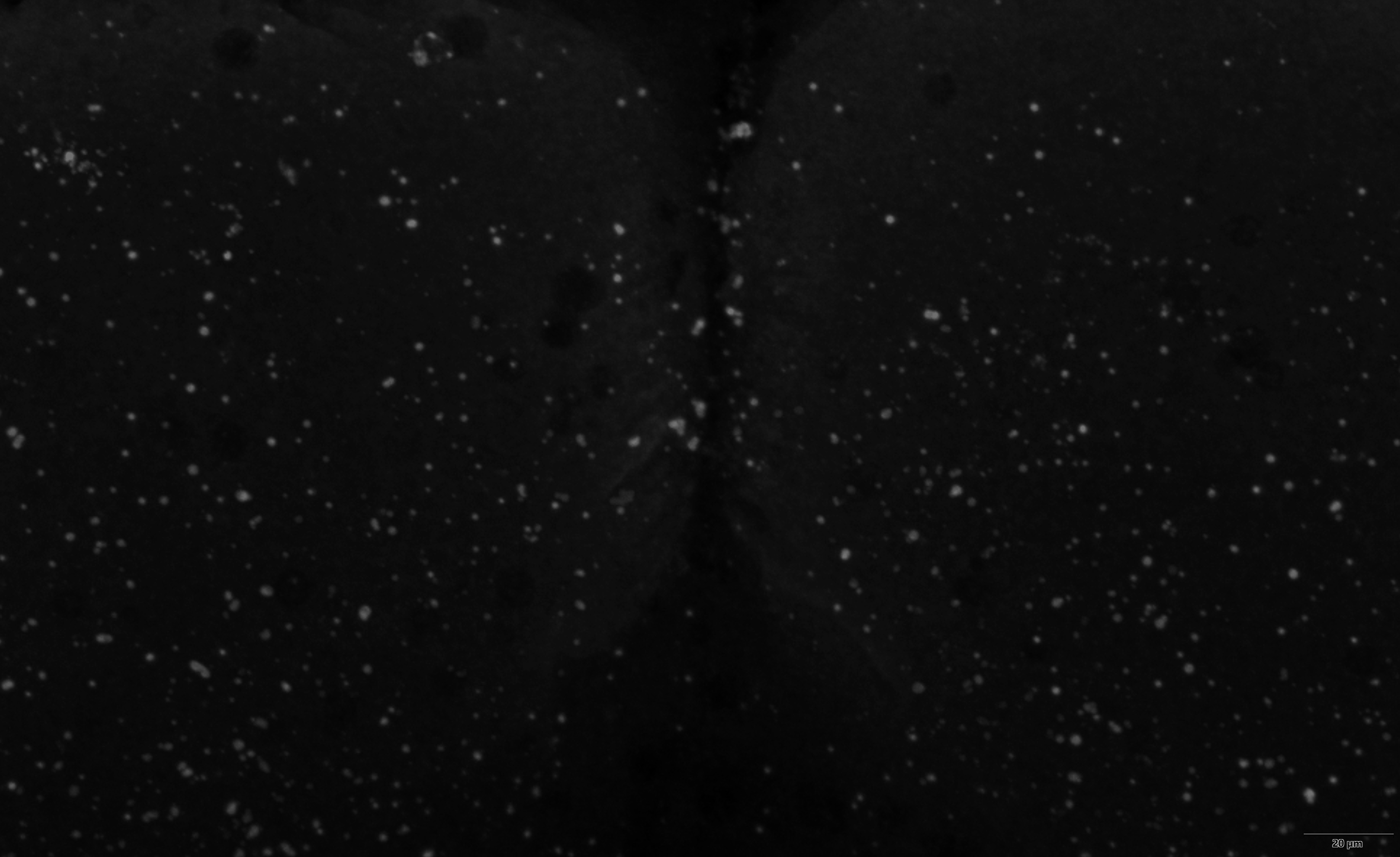

Supplement: Supplementary file 15 — Source data Fig. 4 [file 44318_2025_442_MOESM15_ESM.zip › Figure_4/Figure 4N/wt inj Piwil1.tif]

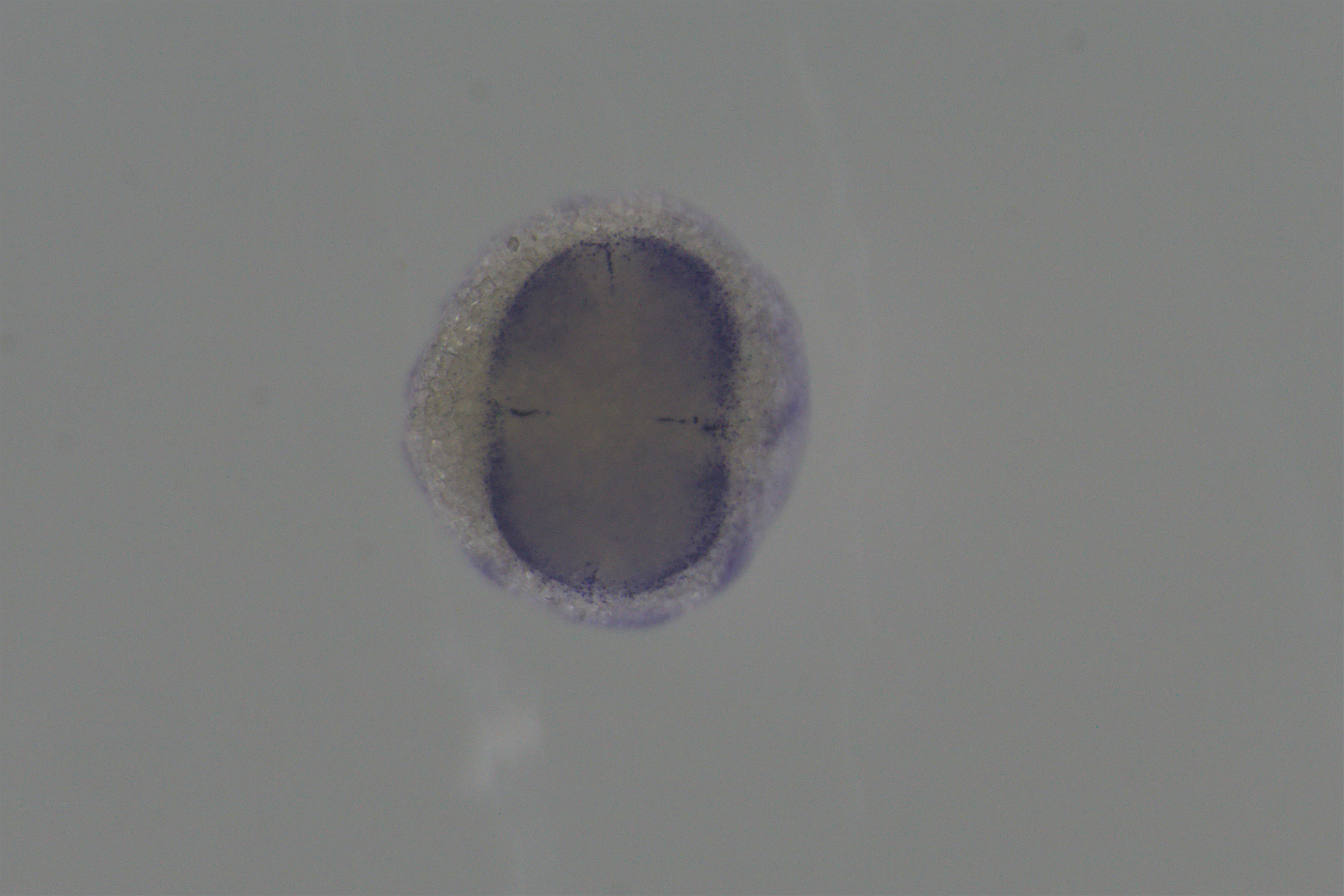

Supplement: Supplementary file 15 — Source data Fig. 4 [file 44318_2025_442_MOESM15_ESM.zip › Figure_4/Figure 4O/IgG ca15b.tif]

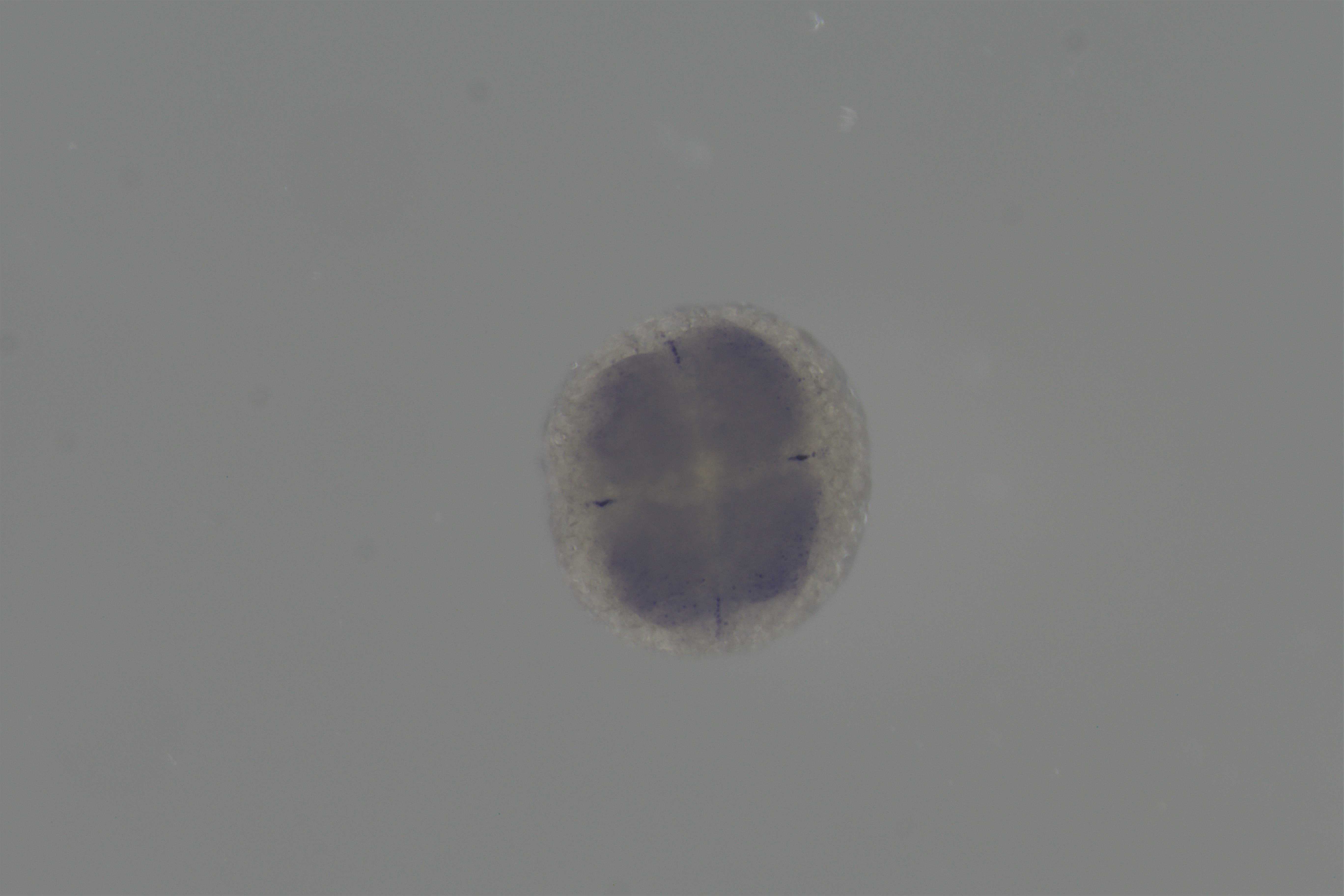

Supplement: Supplementary file 15 — Source data Fig. 4 [file 44318_2025_442_MOESM15_ESM.zip › Figure_4/Figure 4O/IgG ddx4.tif]

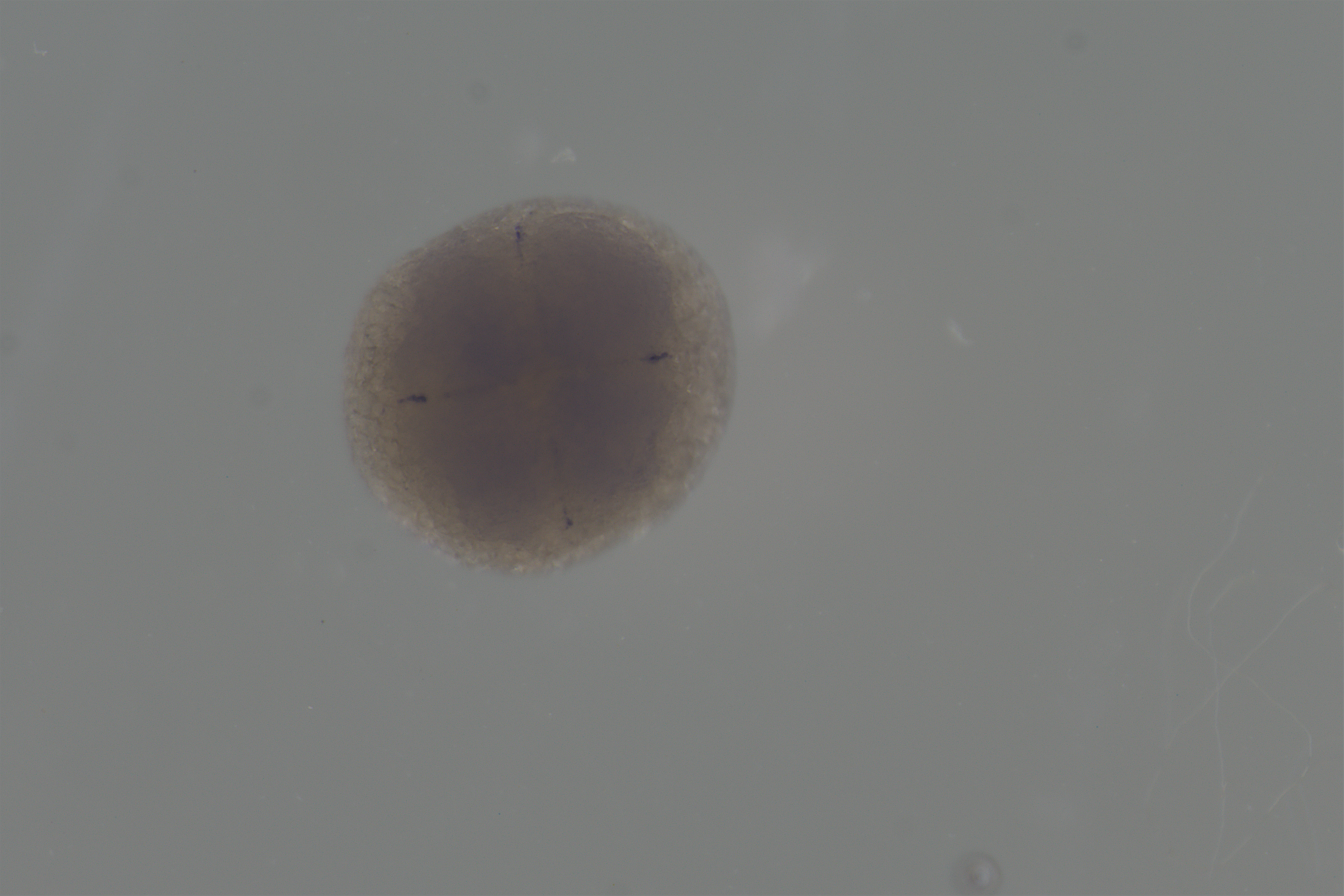

Supplement: Supplementary file 15 — Source data Fig. 4 [file 44318_2025_442_MOESM15_ESM.zip › Figure_4/Figure 4O/IgG dnd1.tif]

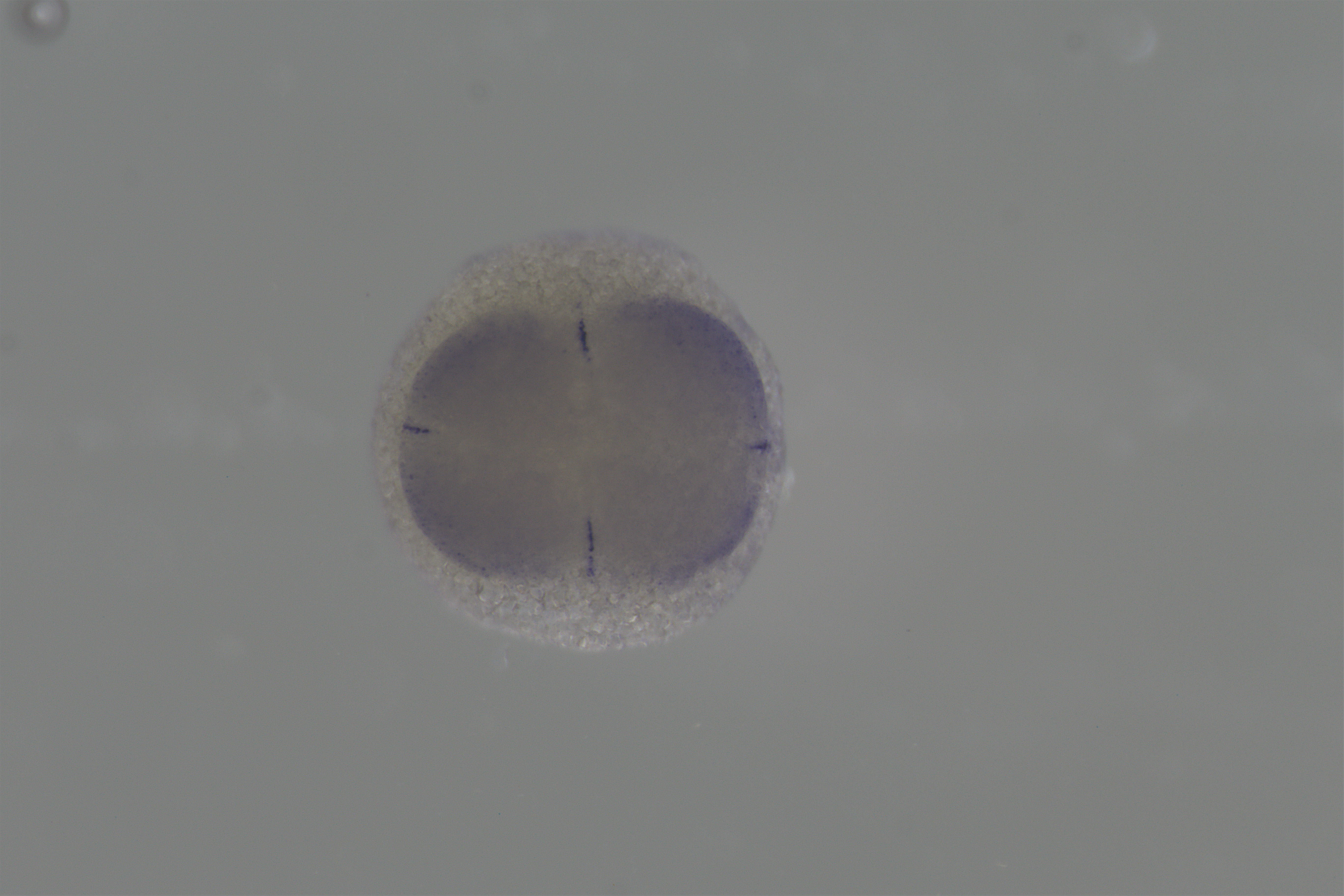

Supplement: Supplementary file 15 — Source data Fig. 4 [file 44318_2025_442_MOESM15_ESM.zip › Figure_4/Figure 4O/IgG kop .tif]

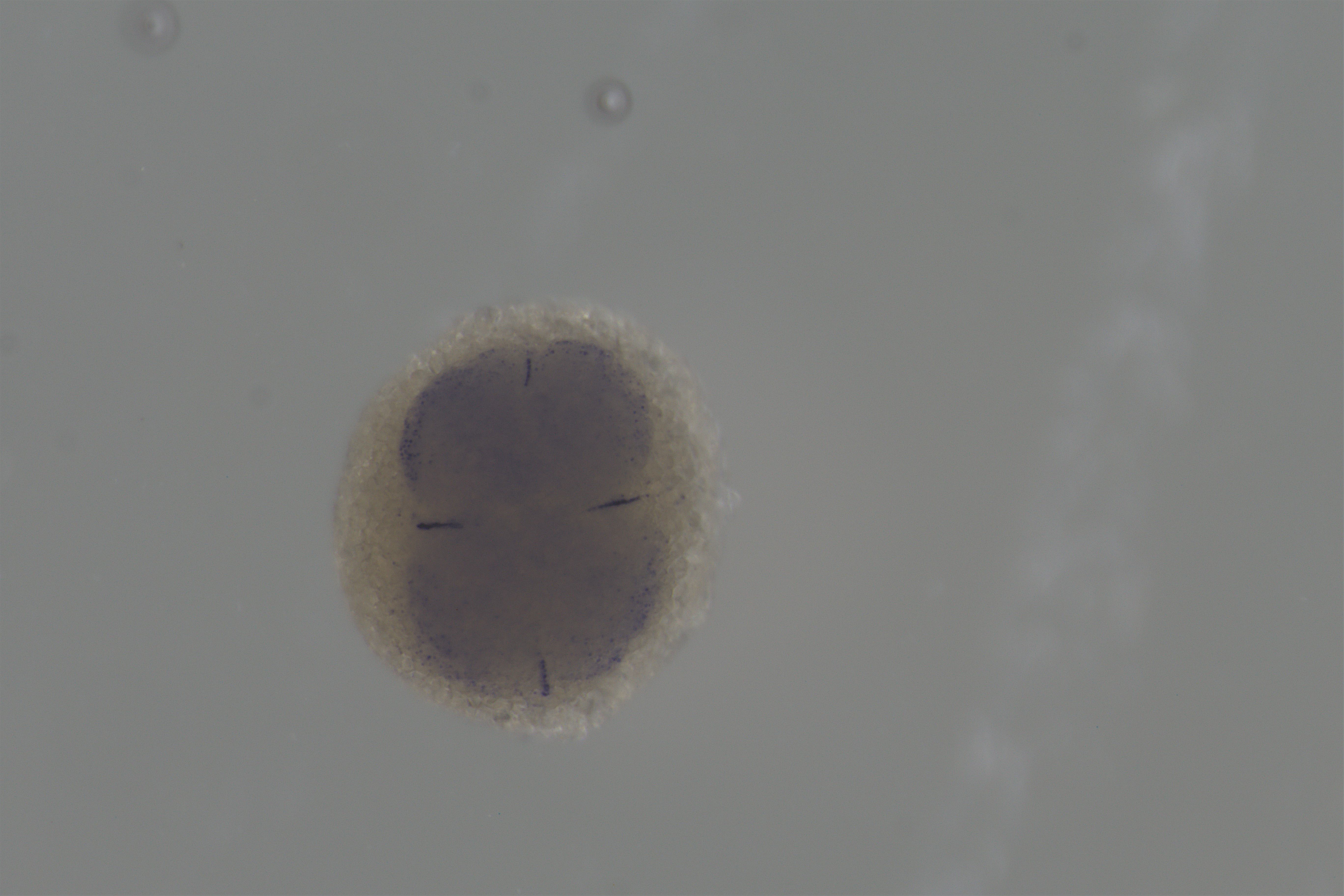

Supplement: Supplementary file 15 — Source data Fig. 4 [file 44318_2025_442_MOESM15_ESM.zip › Figure_4/Figure 4O/IgG nanos3.tif]

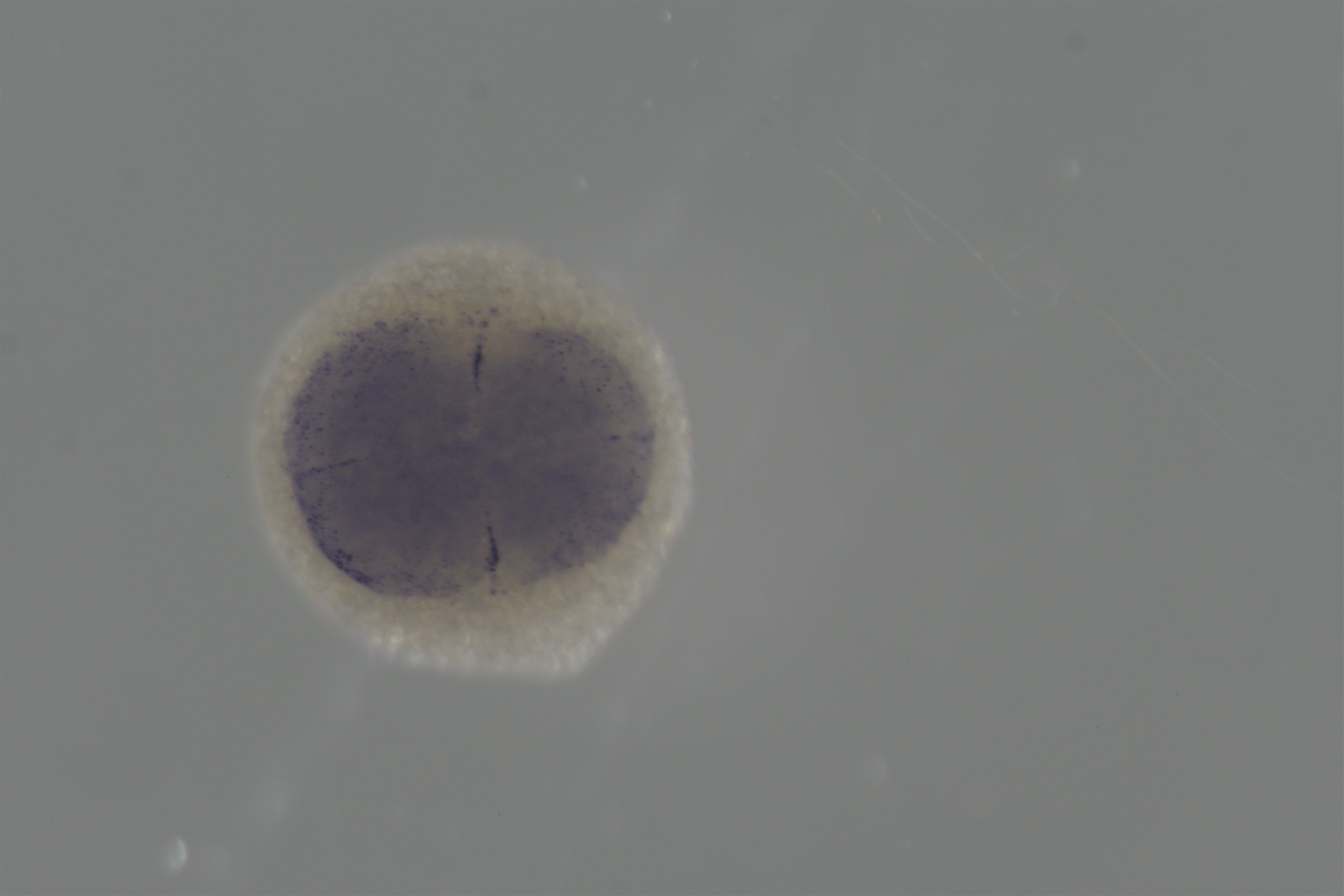

Supplement: Supplementary file 15 — Source data Fig. 4 [file 44318_2025_442_MOESM15_ESM.zip › Figure_4/Figure 4O/igG tdrd7.tif]

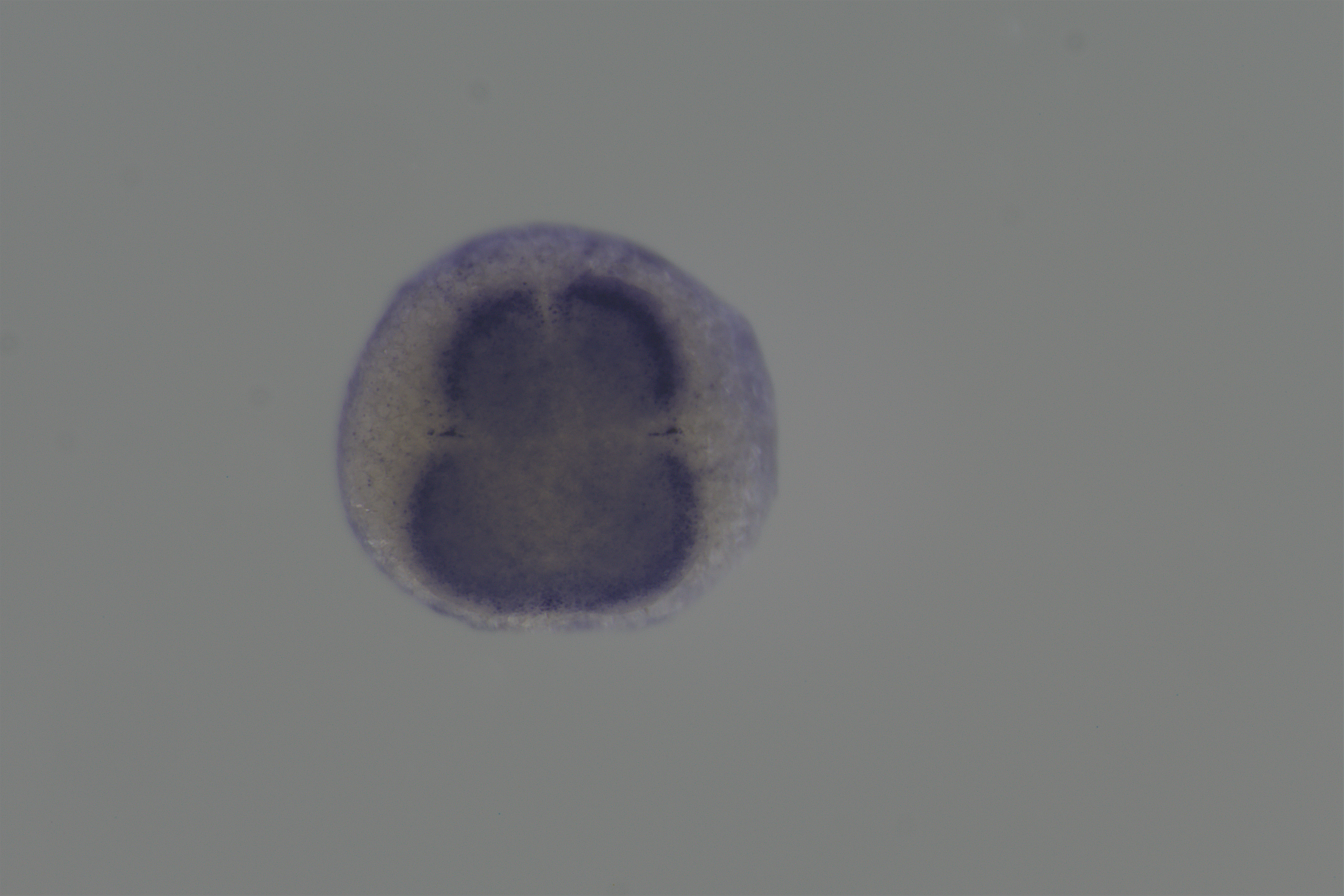

Supplement: Supplementary file 15 — Source data Fig. 4 [file 44318_2025_442_MOESM15_ESM.zip › Figure_4/Figure 4O/kinesin-1 antibody-inj ca15b.tif]

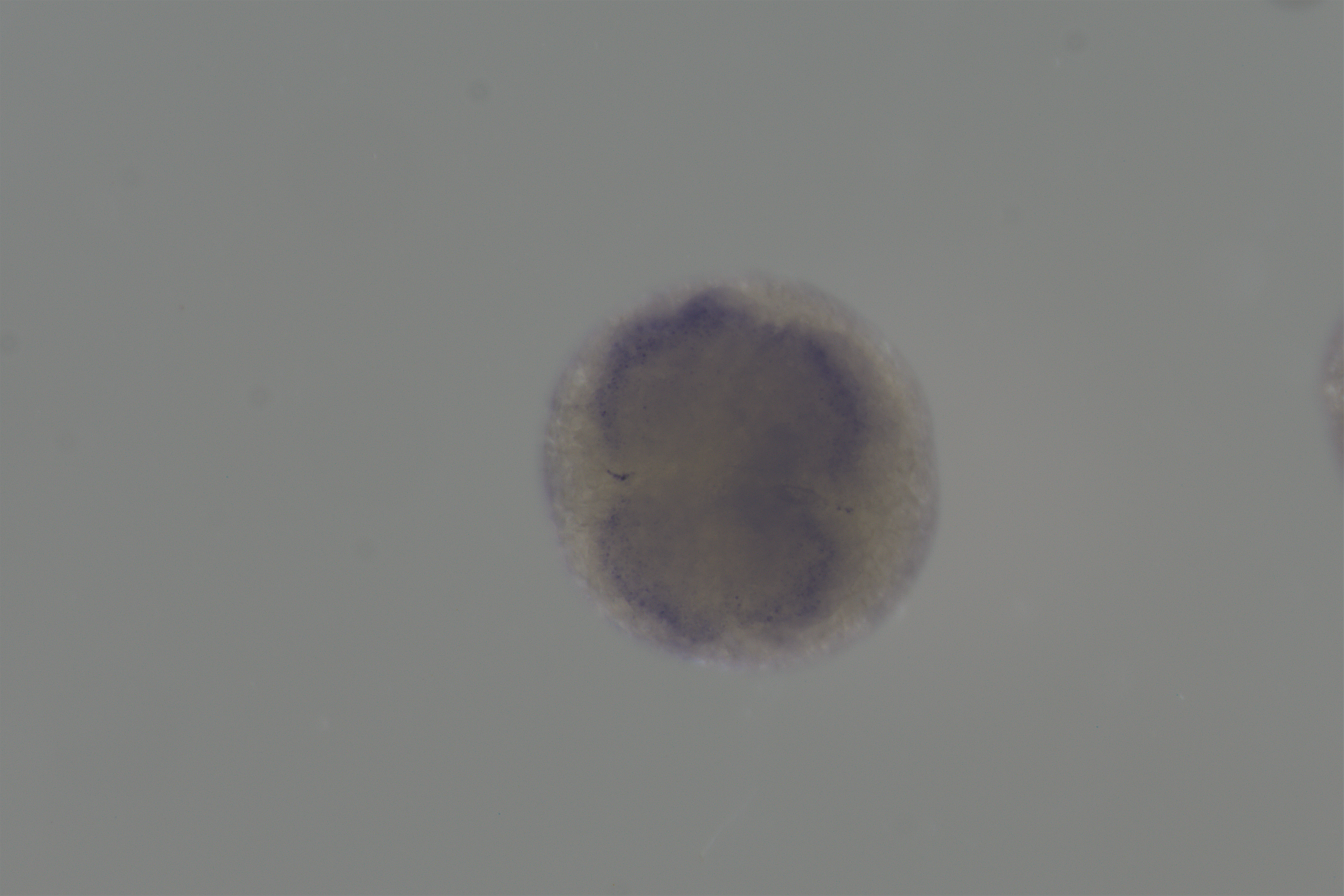

Supplement: Supplementary file 15 — Source data Fig. 4 [file 44318_2025_442_MOESM15_ESM.zip › Figure_4/Figure 4O/kinesin-1 antibody-inj ddx4.tif]

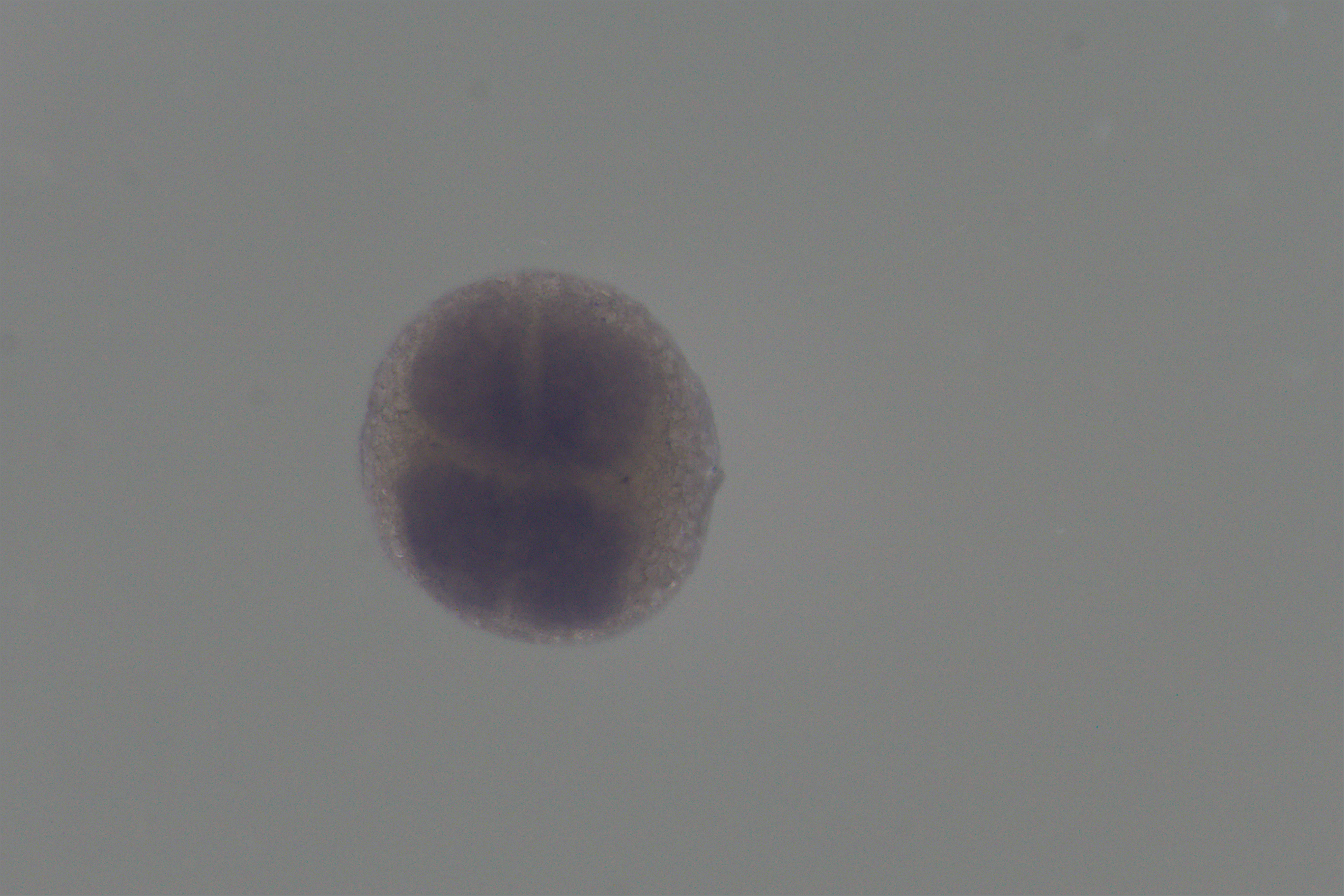

Supplement: Supplementary file 15 — Source data Fig. 4 [file 44318_2025_442_MOESM15_ESM.zip › Figure_4/Figure 4O/kinesin-1 antibody-inj dnd1.tif]

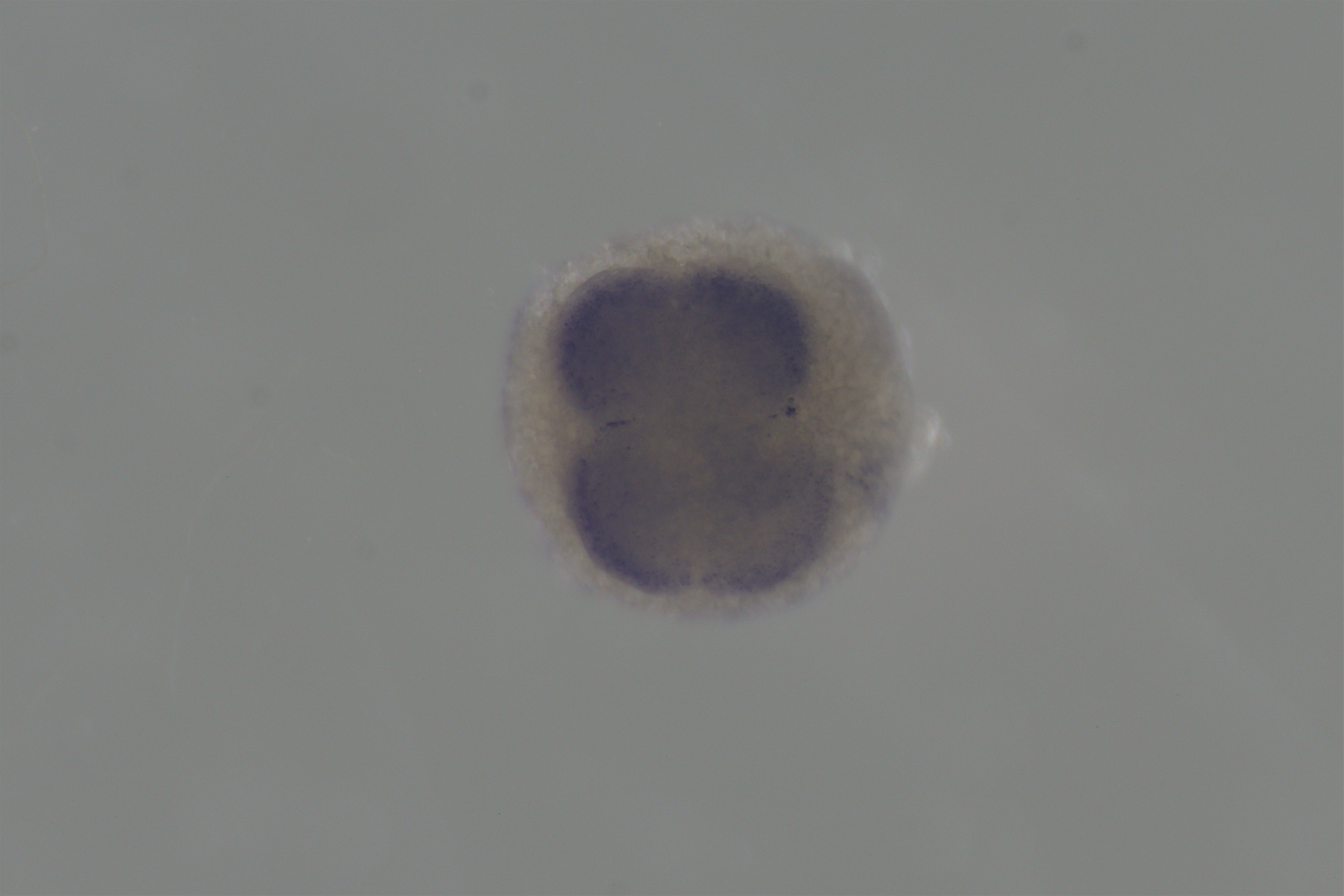

Supplement: Supplementary file 15 — Source data Fig. 4 [file 44318_2025_442_MOESM15_ESM.zip › Figure_4/Figure 4O/kinesin-1 antibody-inj kop.tif]

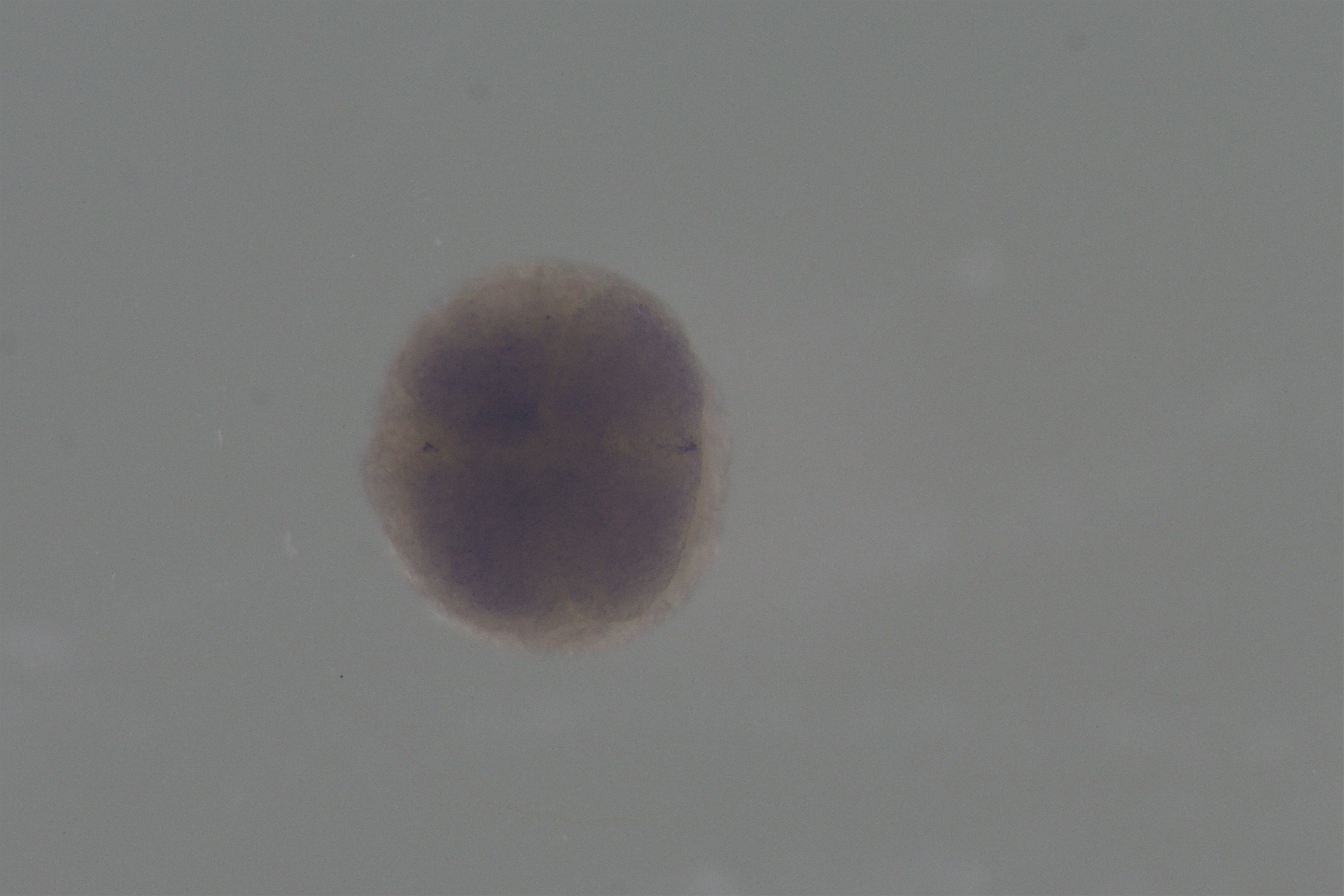

Supplement: Supplementary file 15 — Source data Fig. 4 [file 44318_2025_442_MOESM15_ESM.zip › Figure_4/Figure 4O/kinesin-1 antibody-inj nanos3.tif]

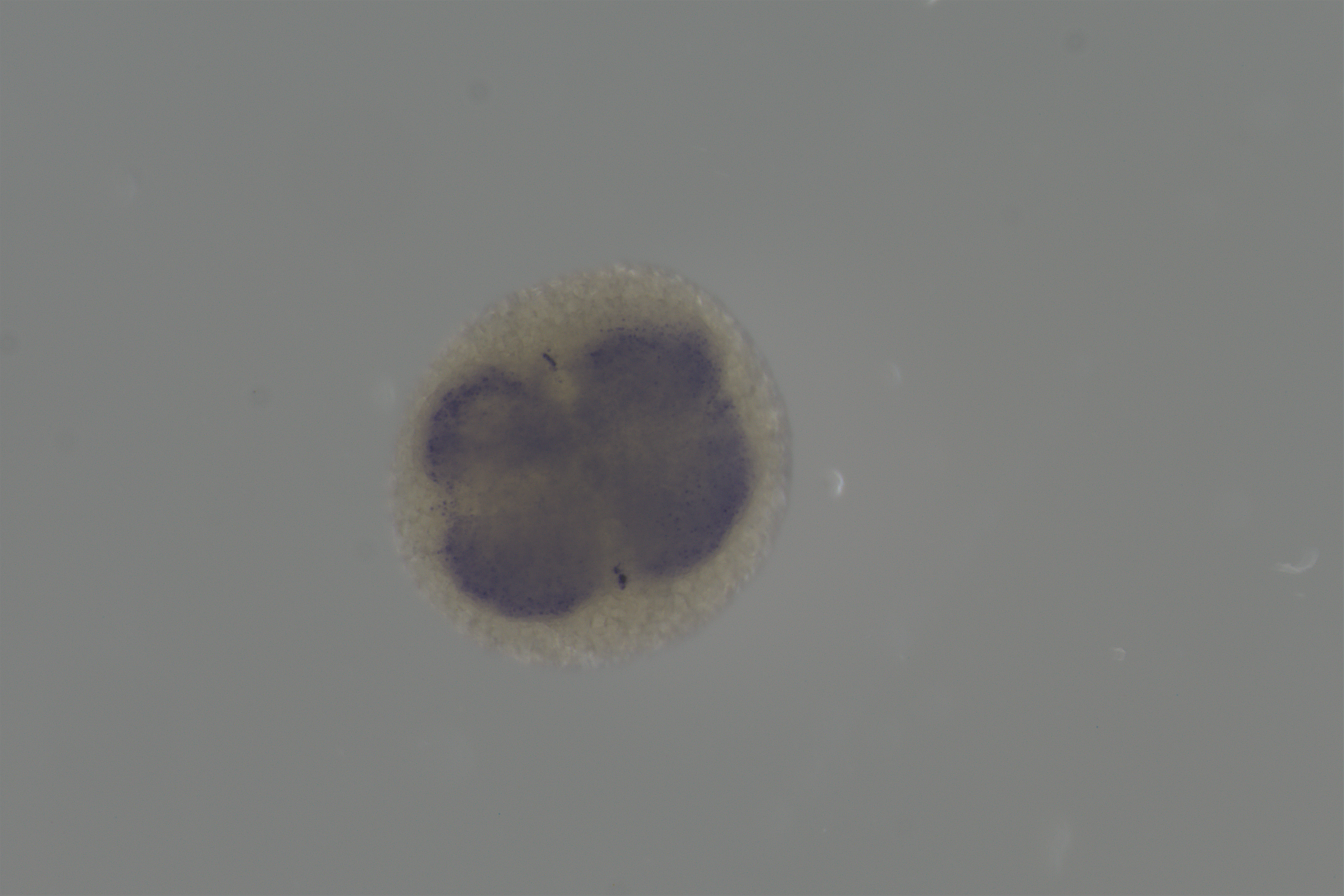

Supplement: Supplementary file 15 — Source data Fig. 4 [file 44318_2025_442_MOESM15_ESM.zip › Figure_4/Figure 4O/kinesin-1 antibody-inj tdrd7.tif]

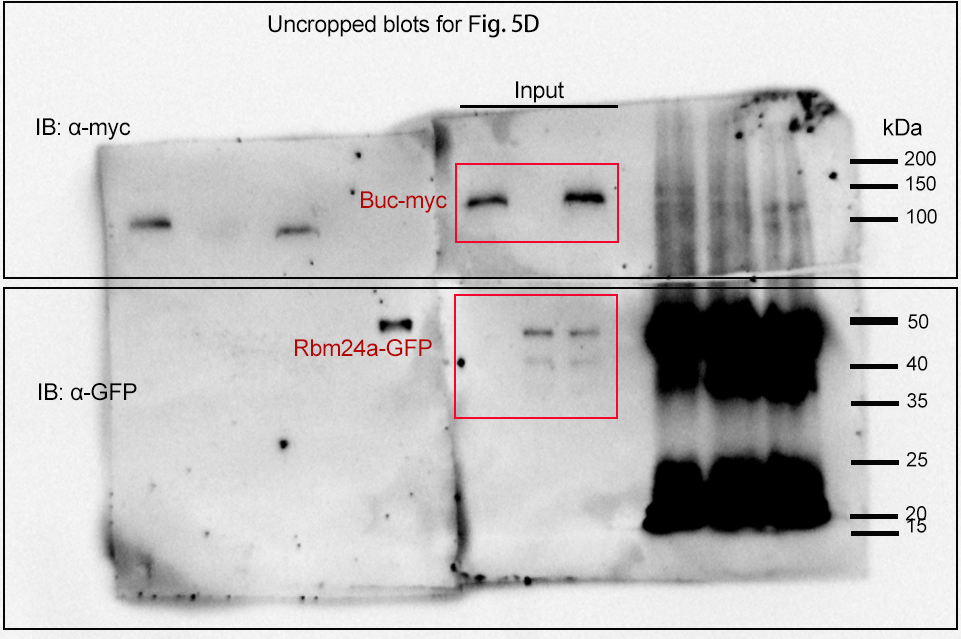

Supplement: Supplementary file 16 — Source data Fig. 5 [file 44318_2025_442_MOESM16_ESM.zip › Figure_5/Figure 5D/1 up and down rbm24a FL 1 .tif]

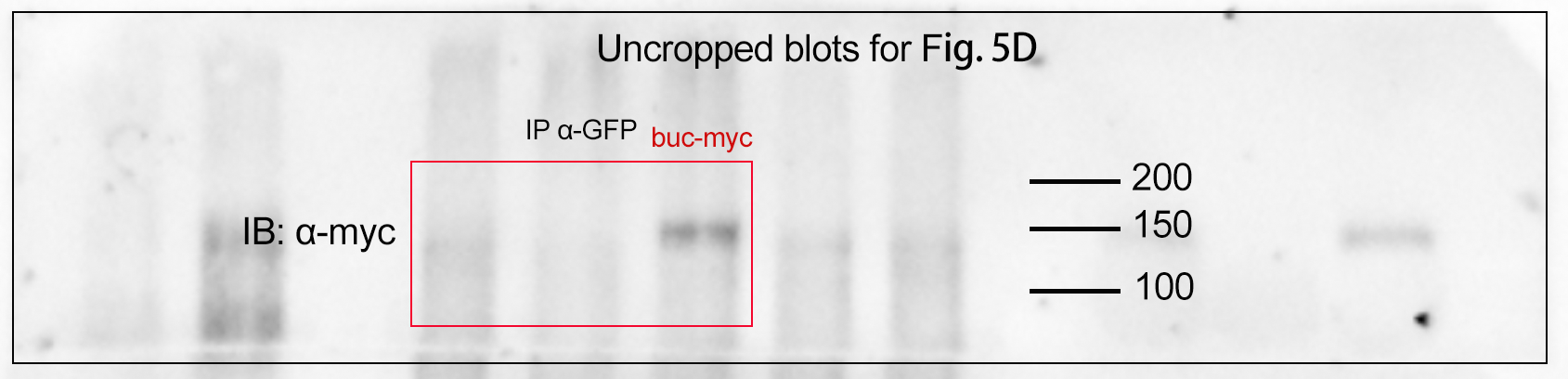

Supplement: Supplementary file 16 — Source data Fig. 5 [file 44318_2025_442_MOESM16_ESM.zip › Figure_5/Figure 5D/2' up buc FL IP .tif]

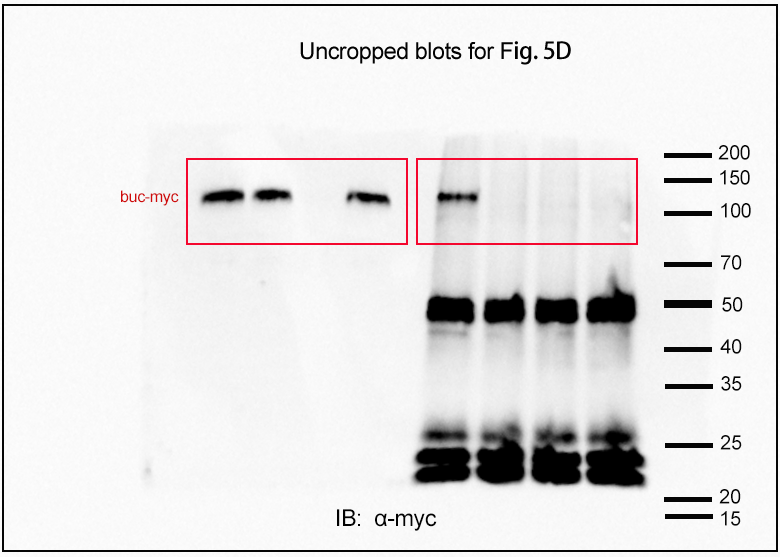

Supplement: Supplementary file 16 — Source data Fig. 5 [file 44318_2025_442_MOESM16_ESM.zip › Figure_5/Figure 5D/3- _4 up añC .tif]

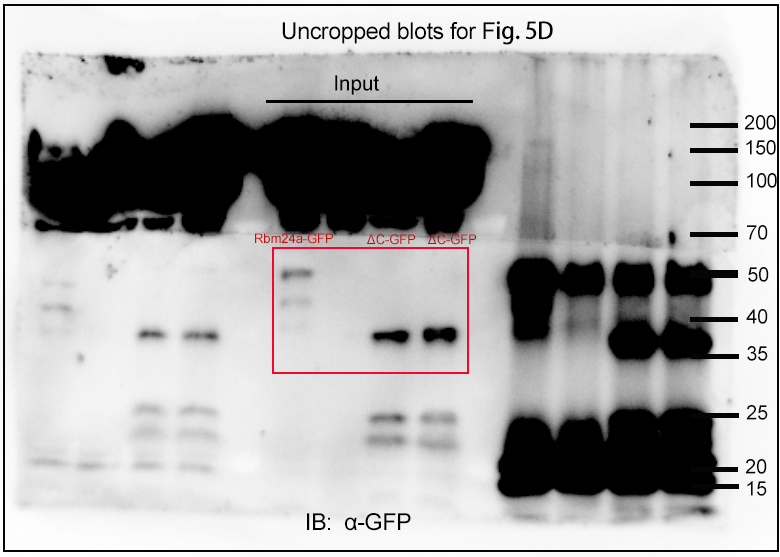

Supplement: Supplementary file 16 — Source data Fig. 5 [file 44318_2025_442_MOESM16_ESM.zip › Figure_5/Figure 5D/3 down añC a┴-gfp input .tif]

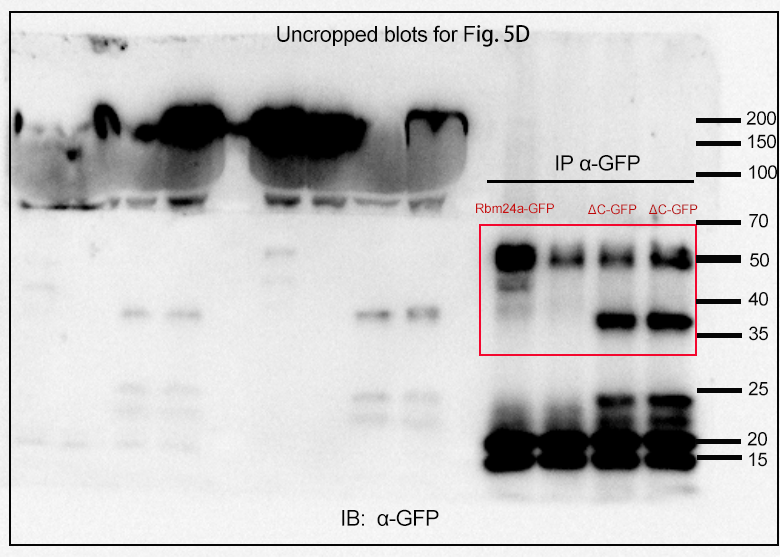

Supplement: Supplementary file 16 — Source data Fig. 5 [file 44318_2025_442_MOESM16_ESM.zip › Figure_5/Figure 5D/4 down añC a┴-gfp .tif]

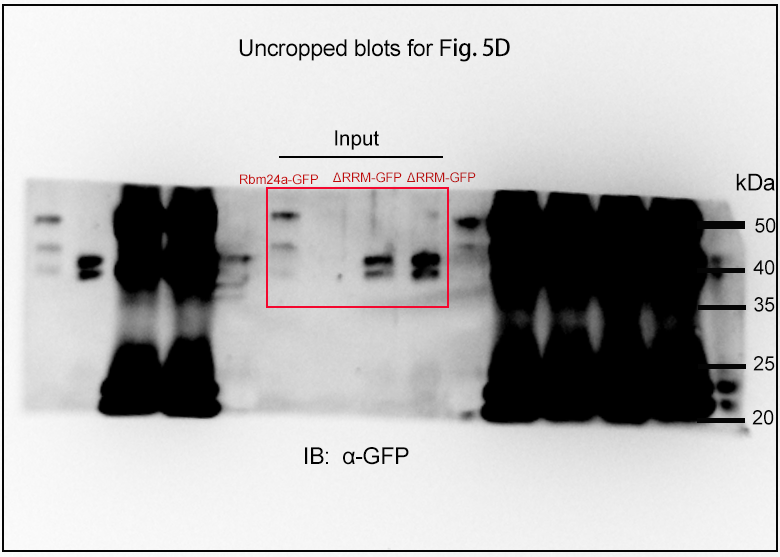

Supplement: Supplementary file 16 — Source data Fig. 5 [file 44318_2025_442_MOESM16_ESM.zip › Figure_5/Figure 5D/5 down añRRM a┴-gfp .tif]

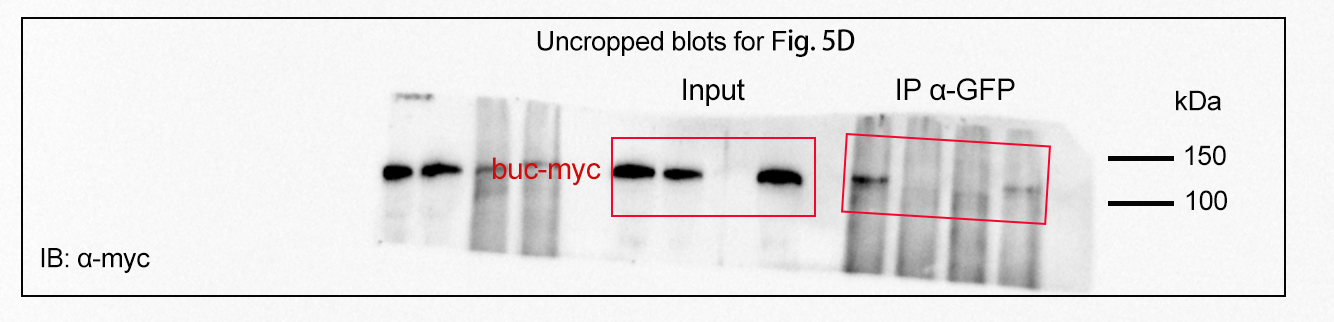

Supplement: Supplementary file 16 — Source data Fig. 5 [file 44318_2025_442_MOESM16_ESM.zip › Figure_5/Figure 5D/5_6 up añRRM a┴-myc .tif]

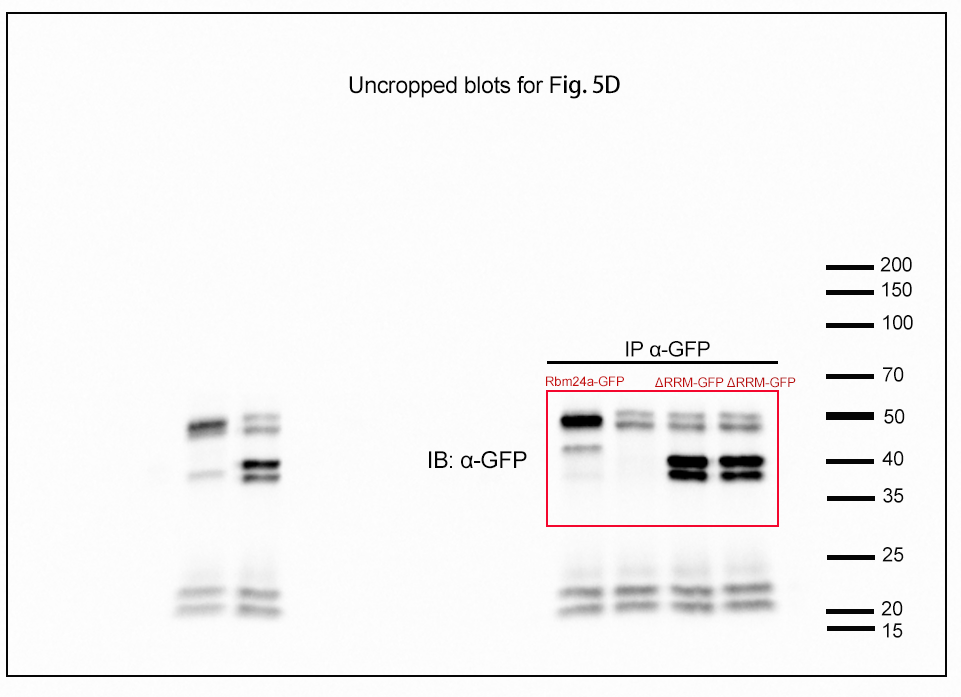

Supplement: Supplementary file 16 — Source data Fig. 5 [file 44318_2025_442_MOESM16_ESM.zip › Figure_5/Figure 5D/6 down añRRM a┴-gfp IP .tif]

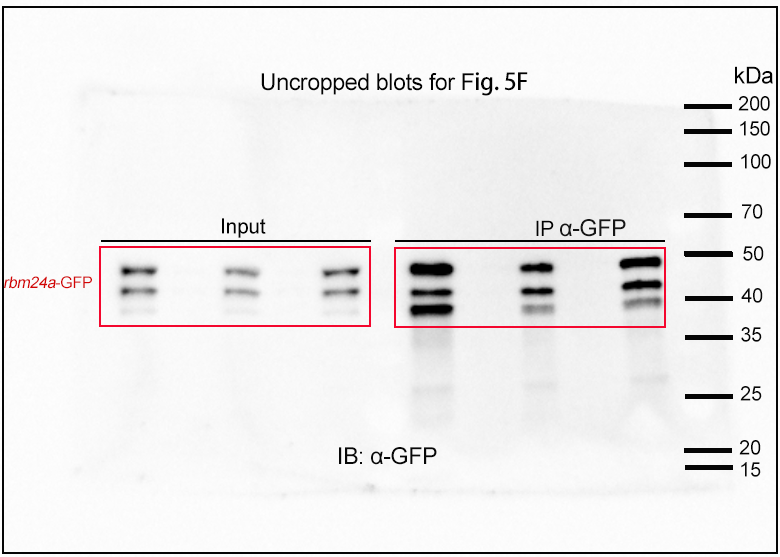

Supplement: Supplementary file 16 — Source data Fig. 5 [file 44318_2025_442_MOESM16_ESM.zip › Figure_5/Figure 5F/buc añ1 añ2 a┴-gfp raw .tif]

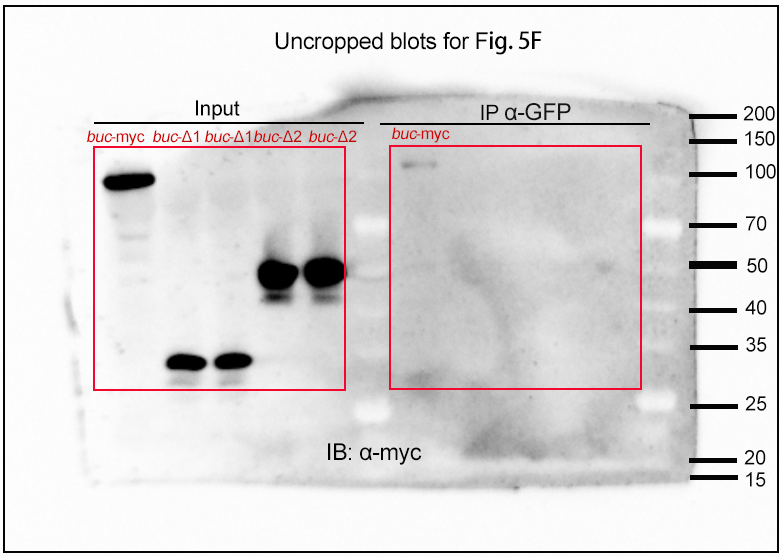

Supplement: Supplementary file 16 — Source data Fig. 5 [file 44318_2025_442_MOESM16_ESM.zip › Figure_5/Figure 5F/buc añ1 añ2 a┴-myc raw .tif]

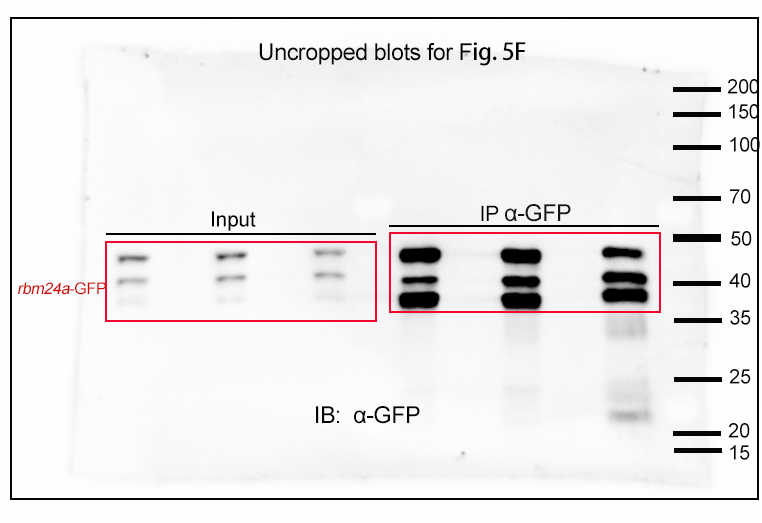

Supplement: Supplementary file 16 — Source data Fig. 5 [file 44318_2025_442_MOESM16_ESM.zip › Figure_5/Figure 5F/buc añ3 añ5 a┴-gfp raw .tif]

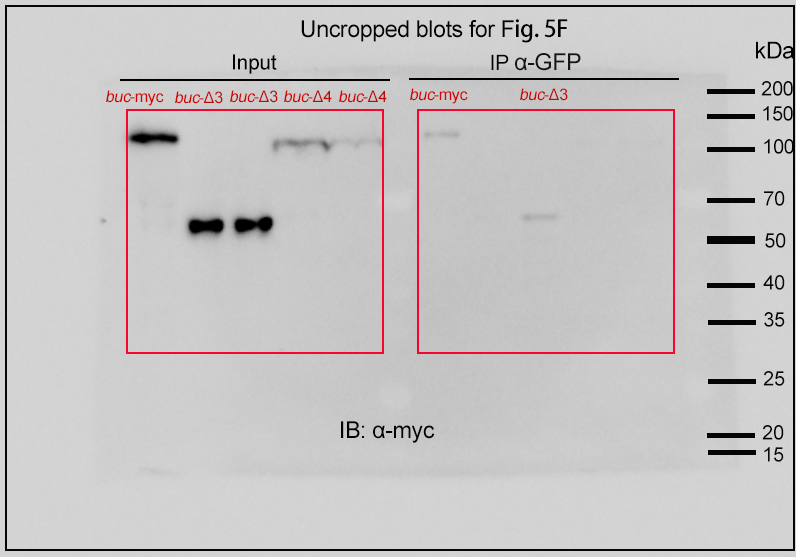

Supplement: Supplementary file 16 — Source data Fig. 5 [file 44318_2025_442_MOESM16_ESM.zip › Figure_5/Figure 5F/buc añ3 añ5 a┴-myc raw .tif]

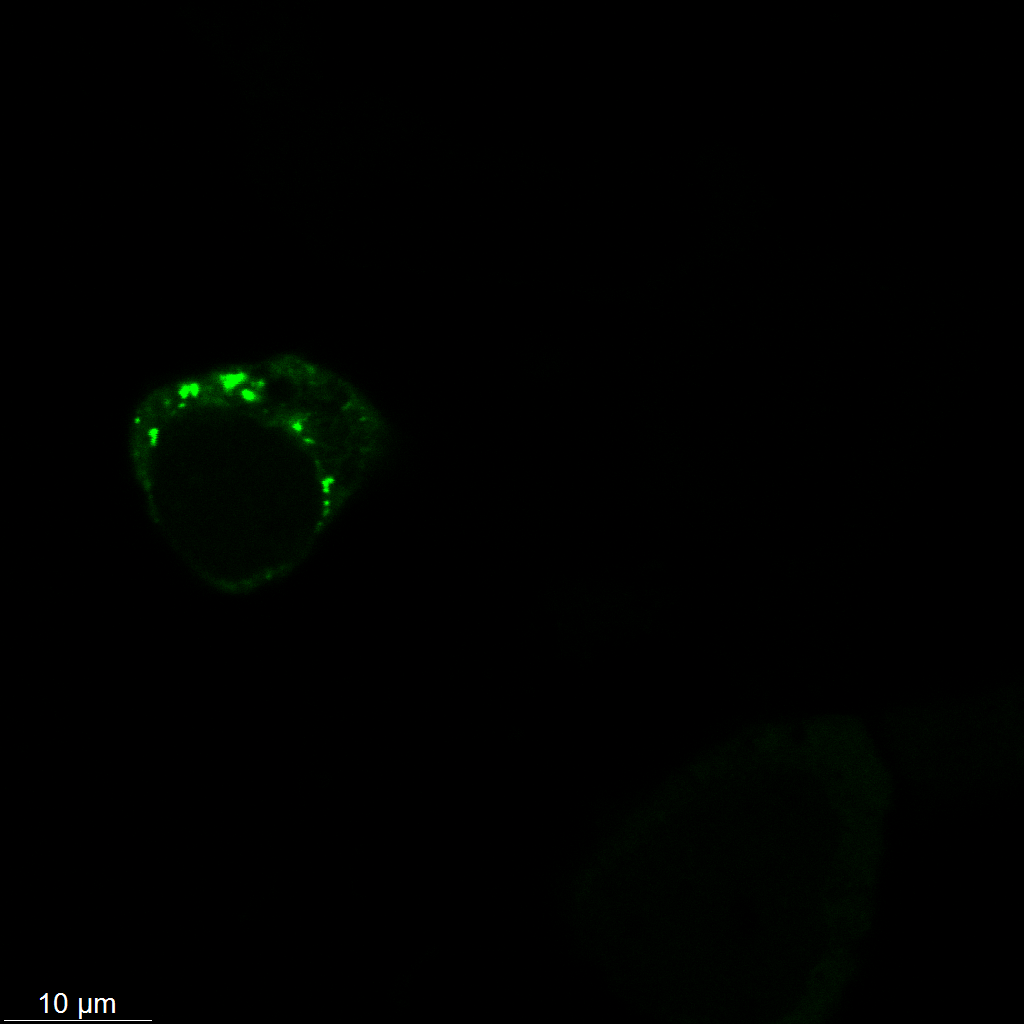

Supplement: Supplementary file 16 — Source data Fig. 5 [file 44318_2025_442_MOESM16_ESM.zip › Figure_5/Figure 5G/Buc-GFP.tif]

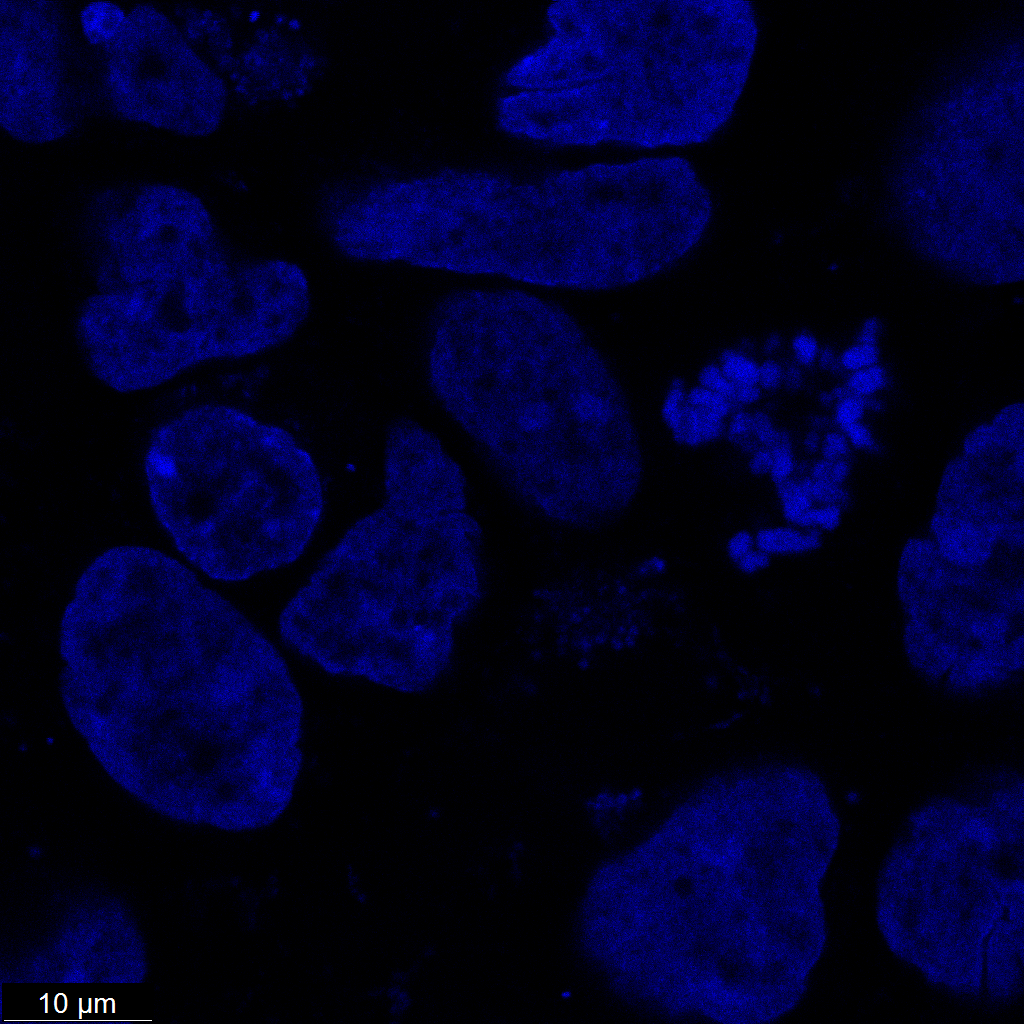

Supplement: Supplementary file 16 — Source data Fig. 5 [file 44318_2025_442_MOESM16_ESM.zip › Figure_5/Figure 5G/DAPI.tif]

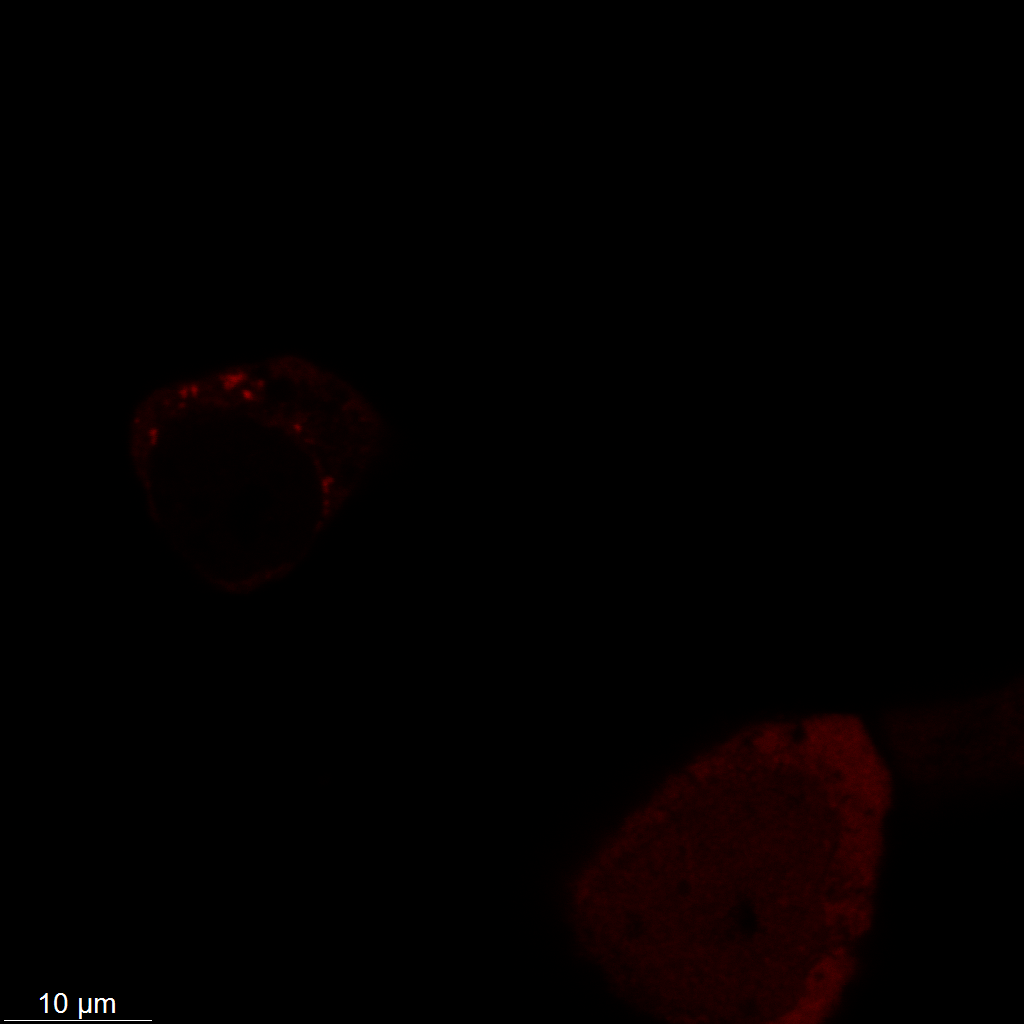

Supplement: Supplementary file 16 — Source data Fig. 5 [file 44318_2025_442_MOESM16_ESM.zip › Figure_5/Figure 5G/Rbm24a-RFP.tif]

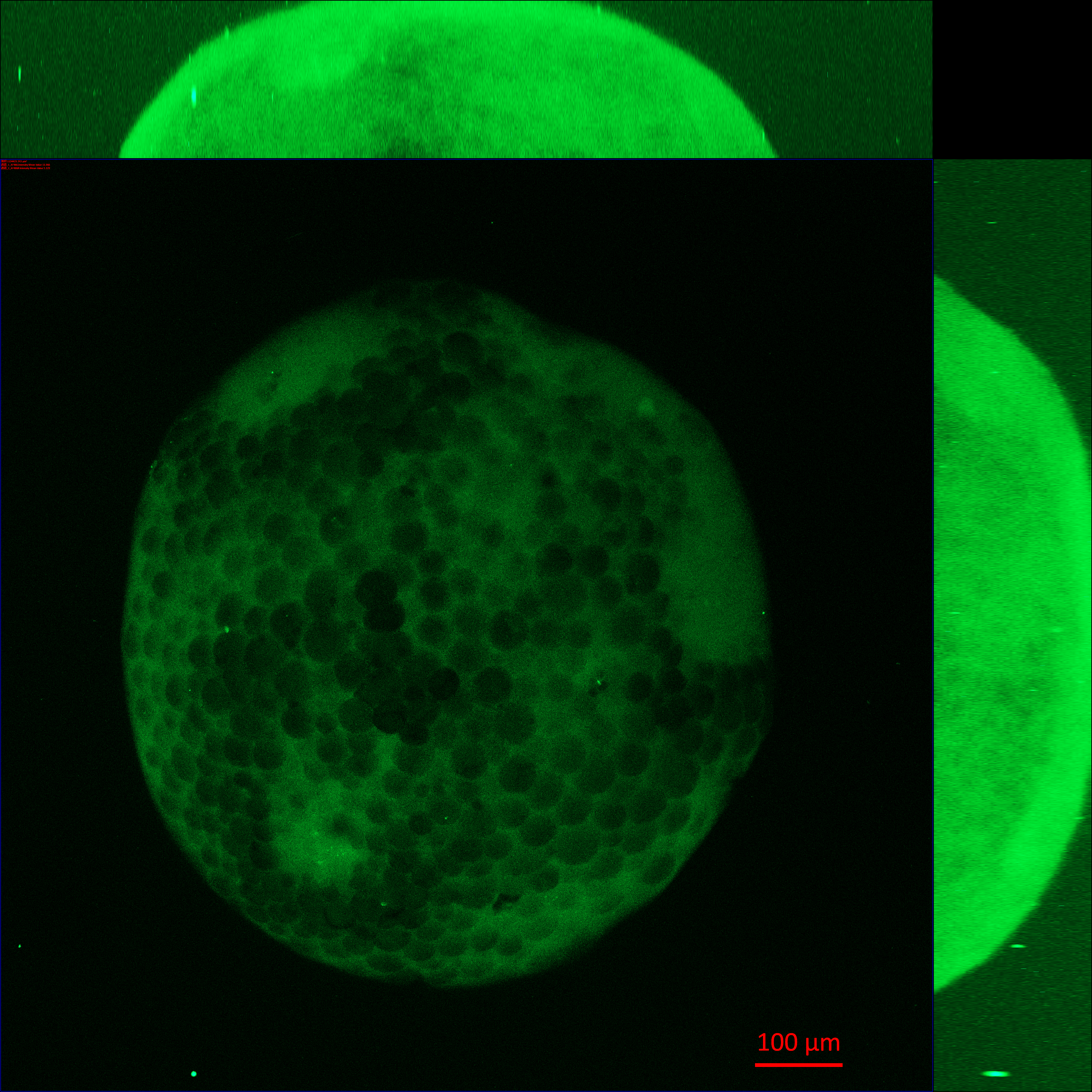

Supplement: Supplementary file 16 — Source data Fig. 5 [file 44318_2025_442_MOESM16_ESM.zip › Figure_5/Figure 5H/Mbuc 10x .tif]

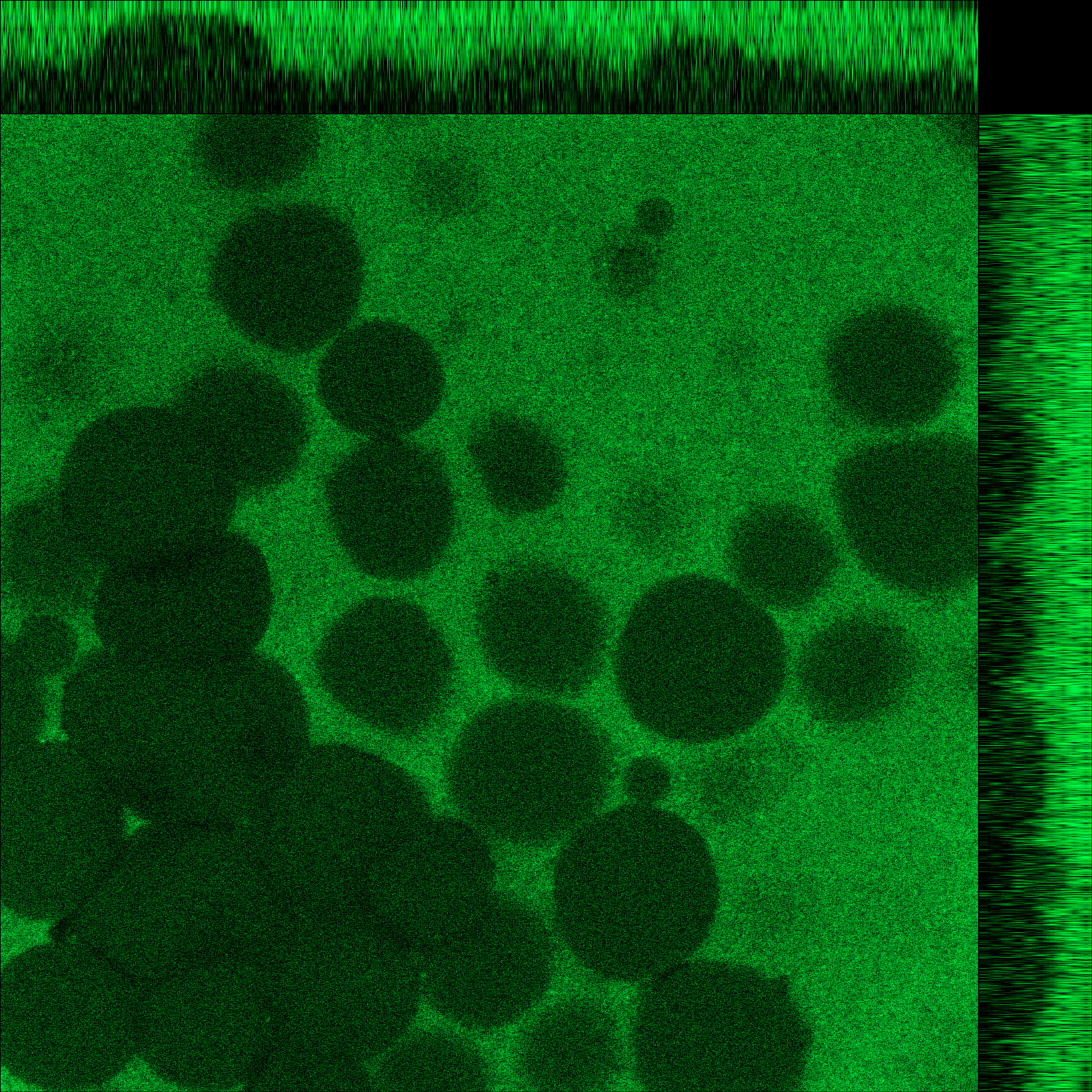

Supplement: Supplementary file 16 — Source data Fig. 5 [file 44318_2025_442_MOESM16_ESM.zip › Figure_5/Figure 5H/Mbuc 40x.tif]

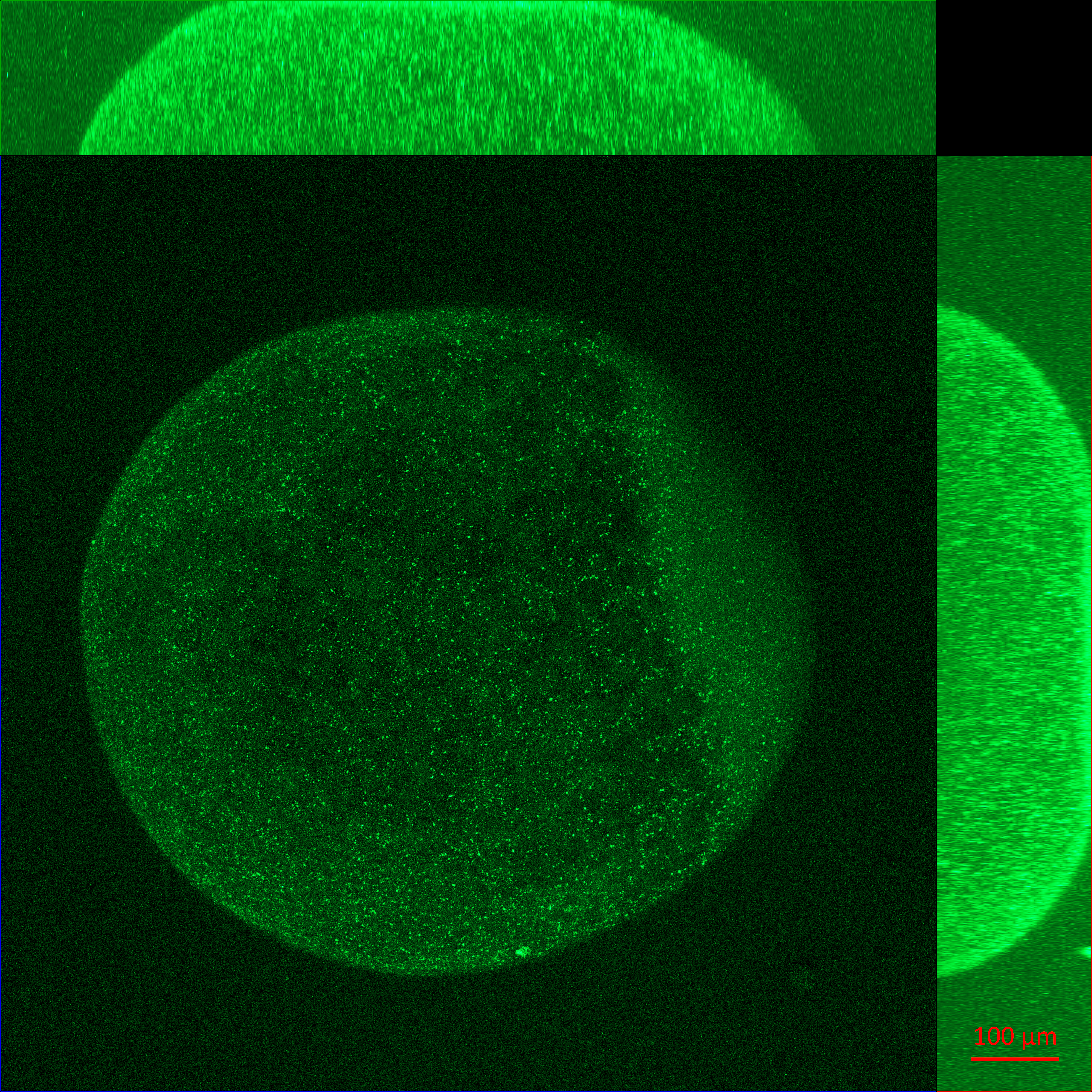

Supplement: Supplementary file 16 — Source data Fig. 5 [file 44318_2025_442_MOESM16_ESM.zip › Figure_5/Figure 5H/rbm24a-GFP KI 10X.tif]

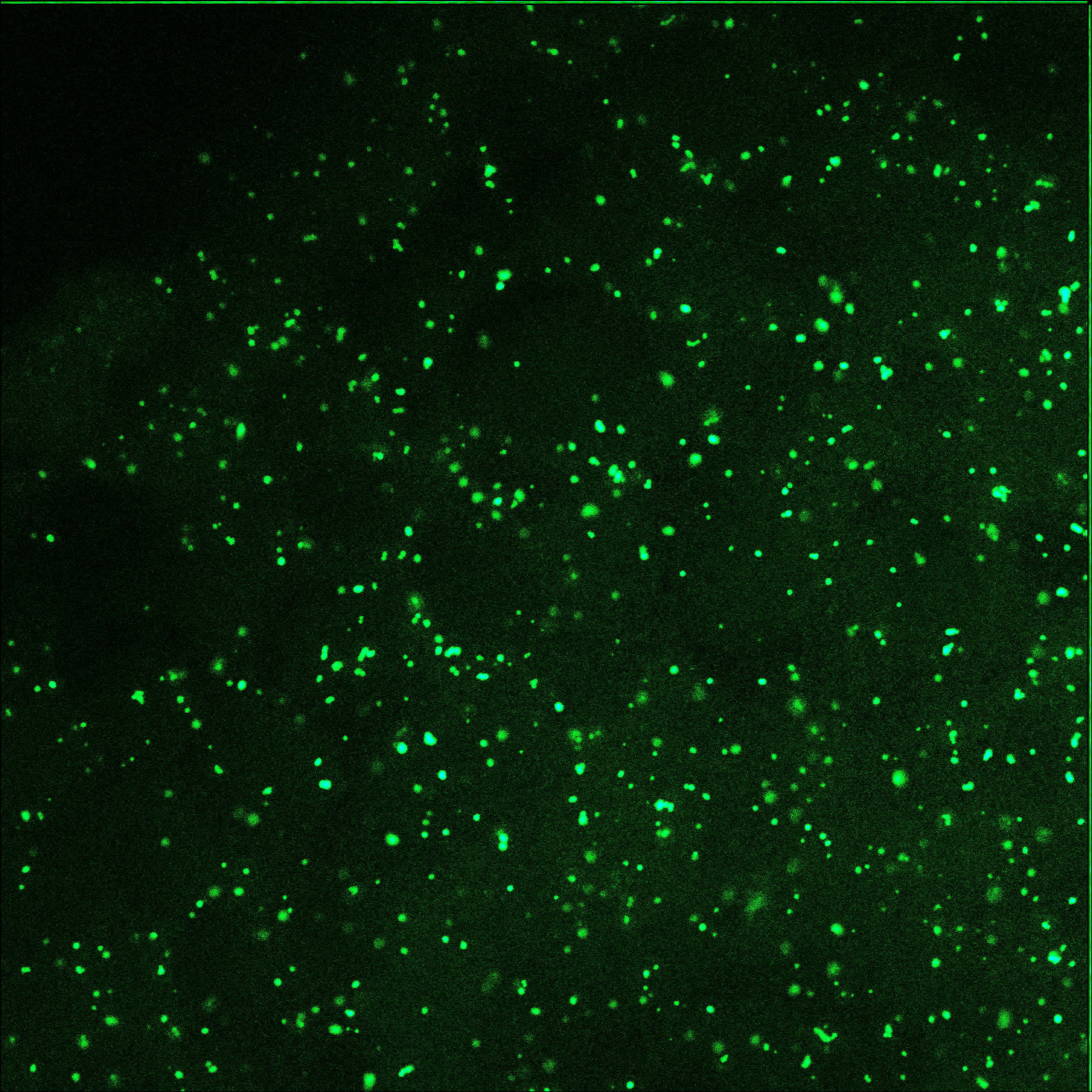

Supplement: Supplementary file 16 — Source data Fig. 5 [file 44318_2025_442_MOESM16_ESM.zip › Figure_5/Figure 5H/rbm24a-GFP KI 40X.tif]

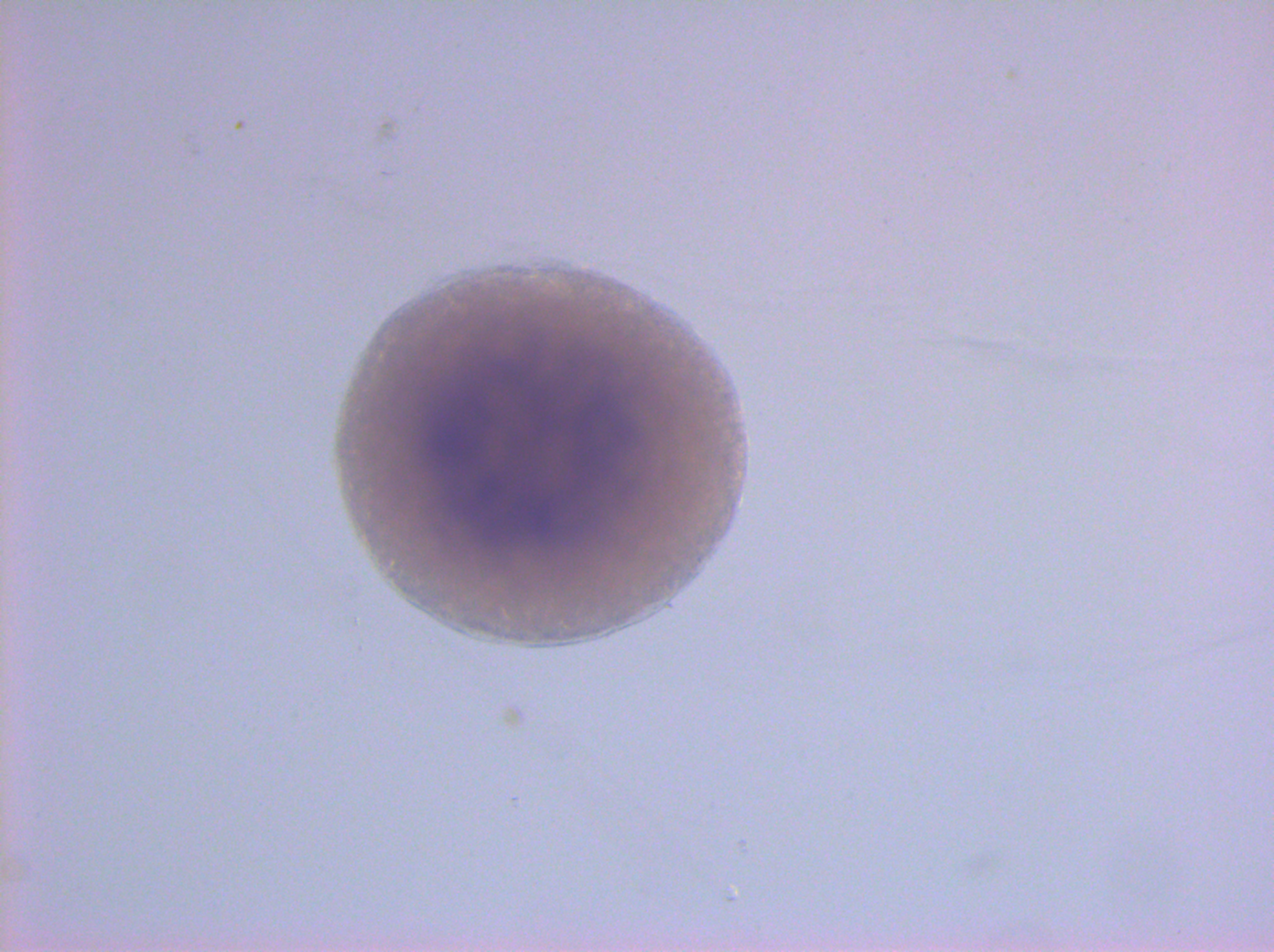

Supplement: Supplementary file 18 — Figure EV1 Source Data [file 44318_2025_442_MOESM18_ESM.zip › Figure_EV1/Figure EV1b/wt rbm24a 1 cell .tif]

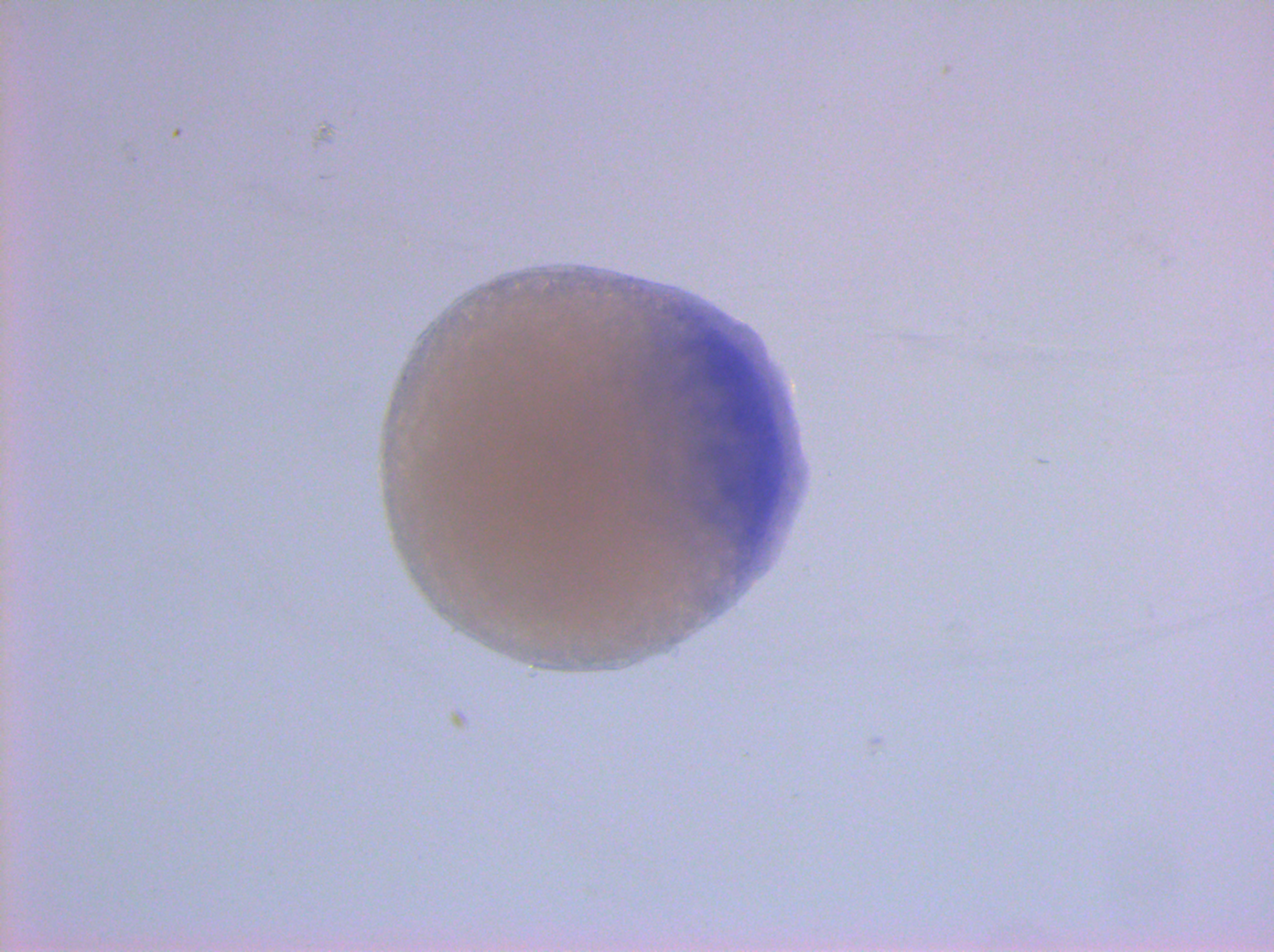

Supplement: Supplementary file 18 — Figure EV1 Source Data [file 44318_2025_442_MOESM18_ESM.zip › Figure_EV1/Figure EV1b/wt rbm24a 1-cell .tif]

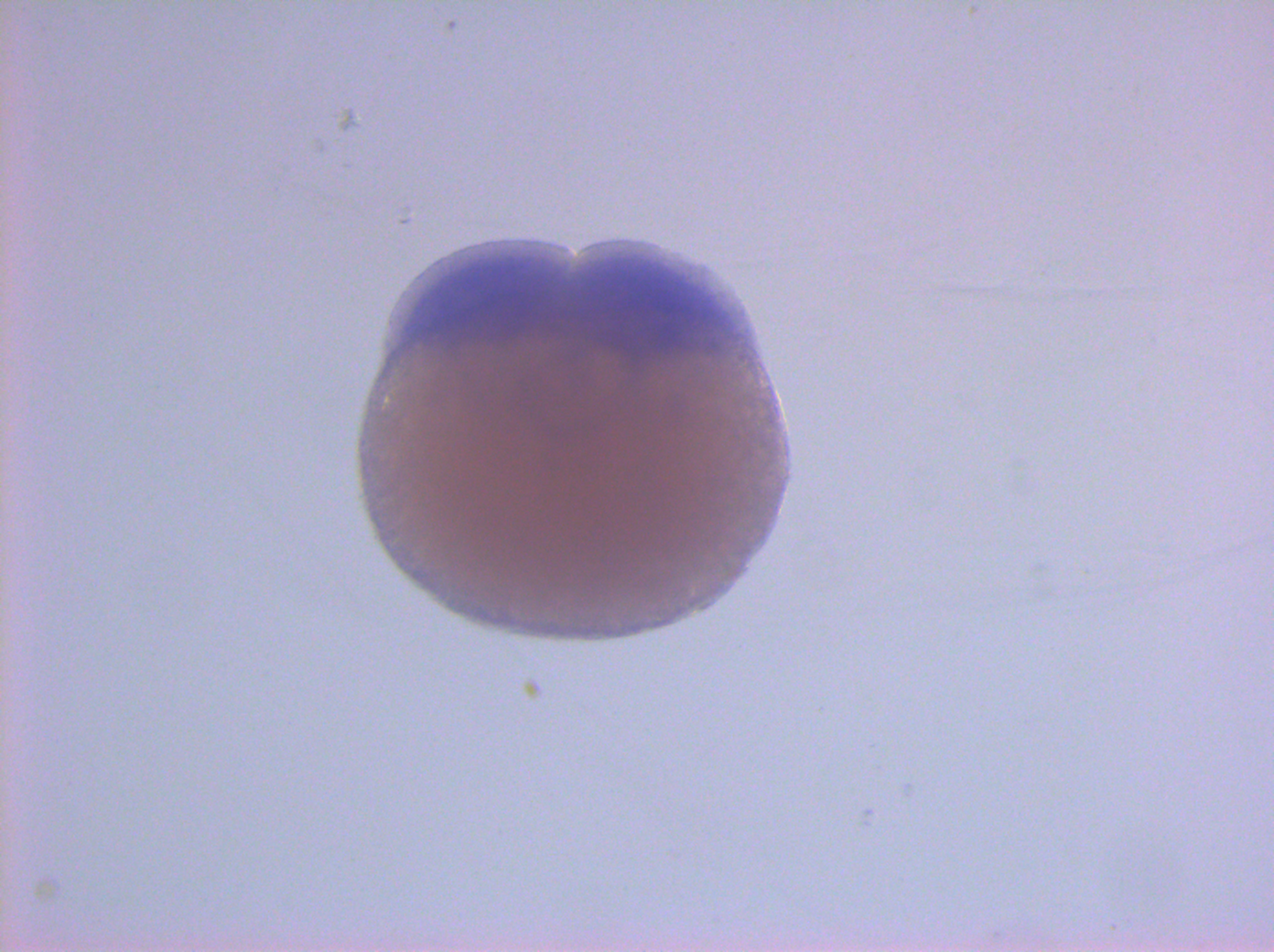

Supplement: Supplementary file 18 — Figure EV1 Source Data [file 44318_2025_442_MOESM18_ESM.zip › Figure_EV1/Figure EV1b/wt rbm24a 2 cell 0005.tif]

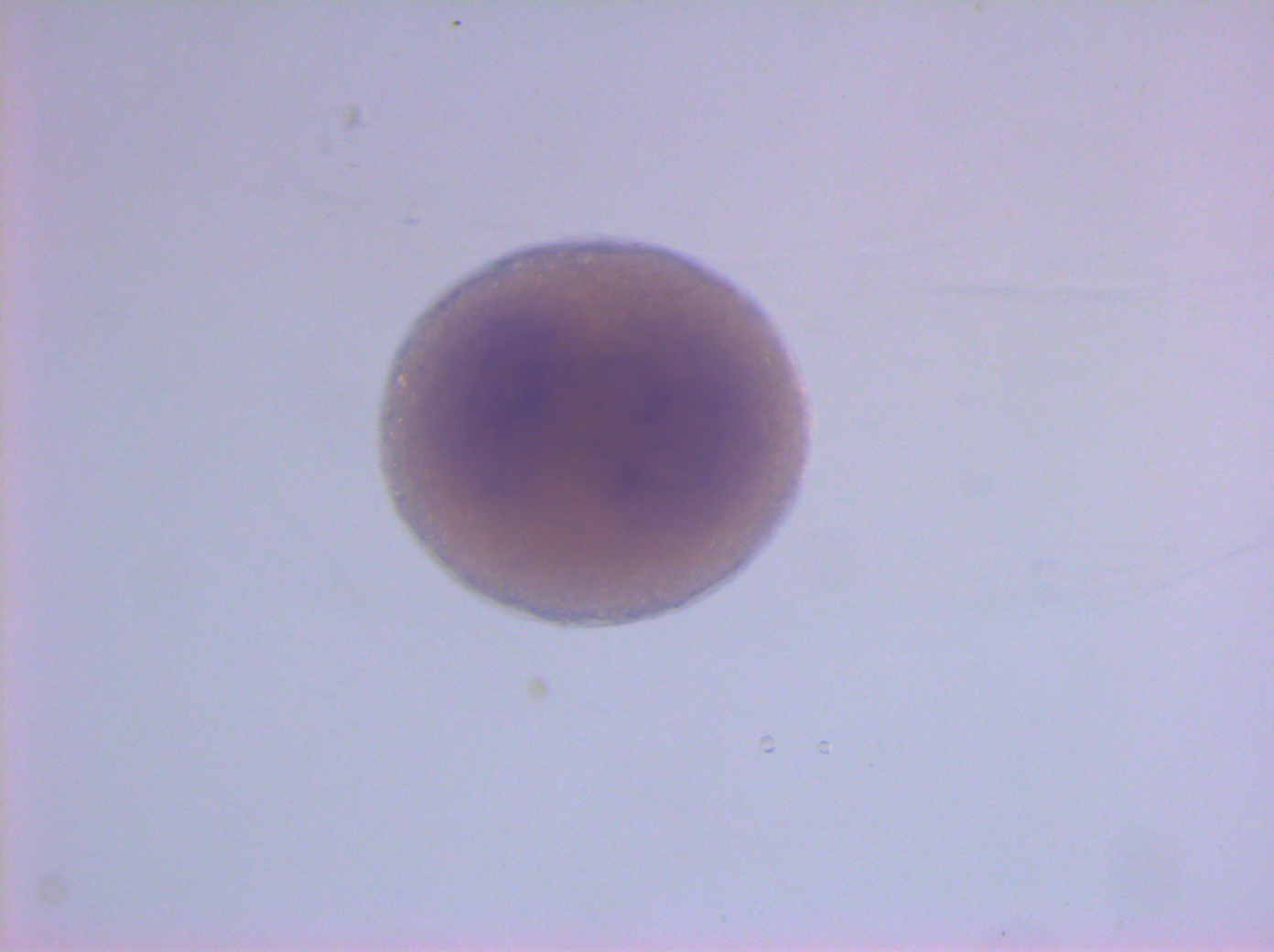

Supplement: Supplementary file 18 — Figure EV1 Source Data [file 44318_2025_442_MOESM18_ESM.zip › Figure_EV1/Figure EV1b/wt rbm24a 2 cell 0006.tif]

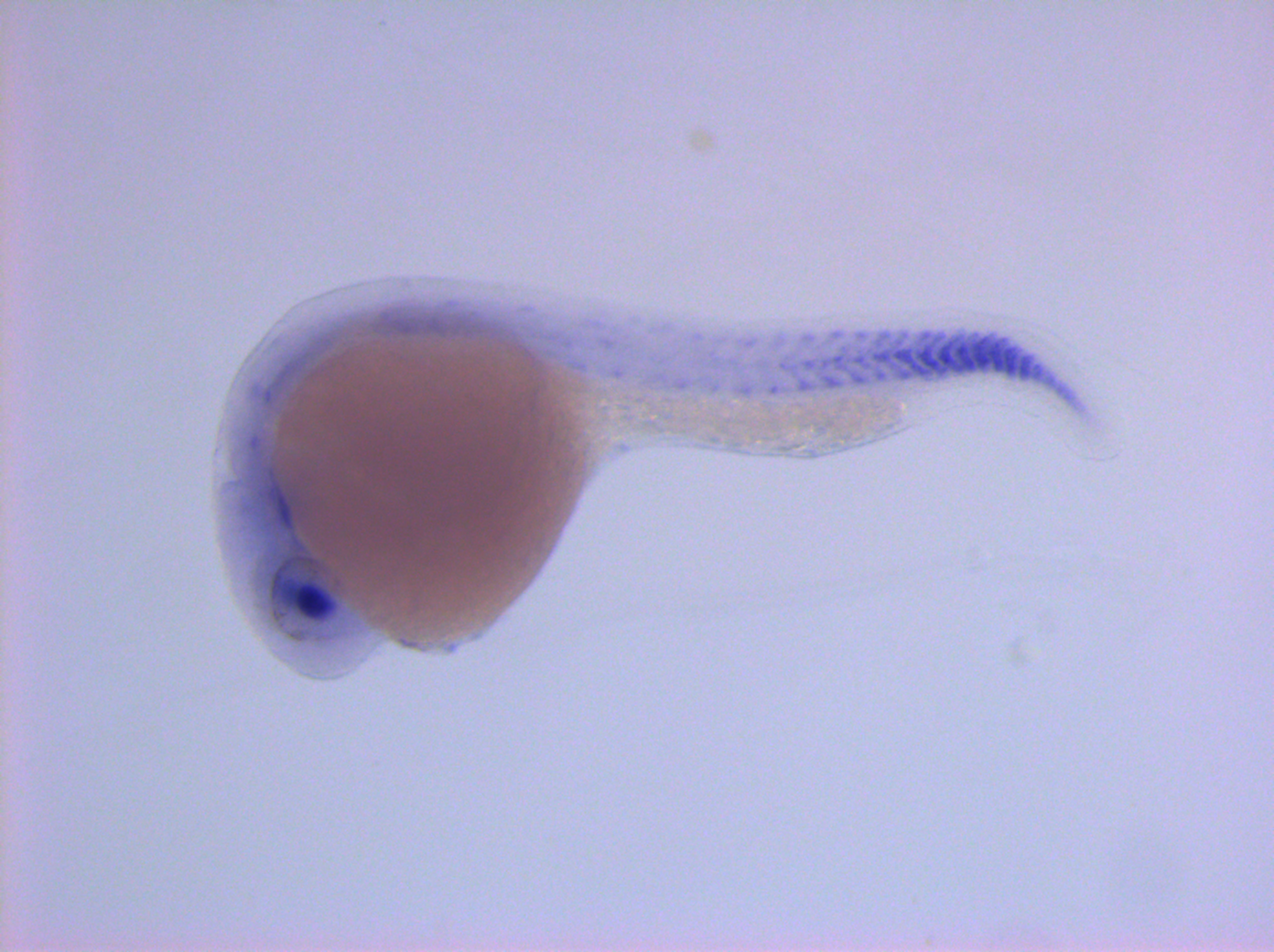

Supplement: Supplementary file 18 — Figure EV1 Source Data [file 44318_2025_442_MOESM18_ESM.zip › Figure_EV1/Figure EV1b/wt rbm24a 24hpf 0015.tif]

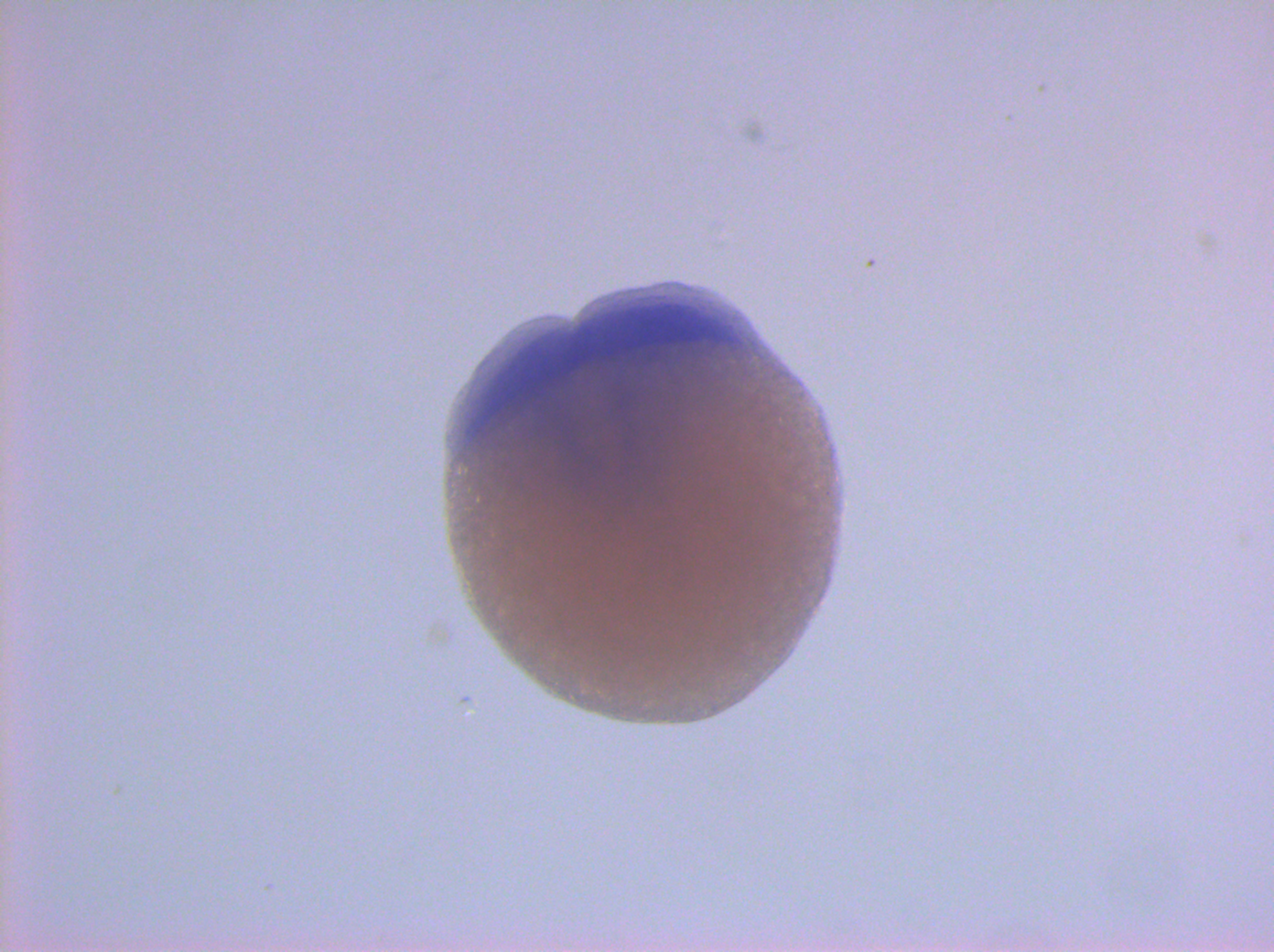

Supplement: Supplementary file 18 — Figure EV1 Source Data [file 44318_2025_442_MOESM18_ESM.zip › Figure_EV1/Figure EV1b/wt rbm24a 4 cell 0007.tif]

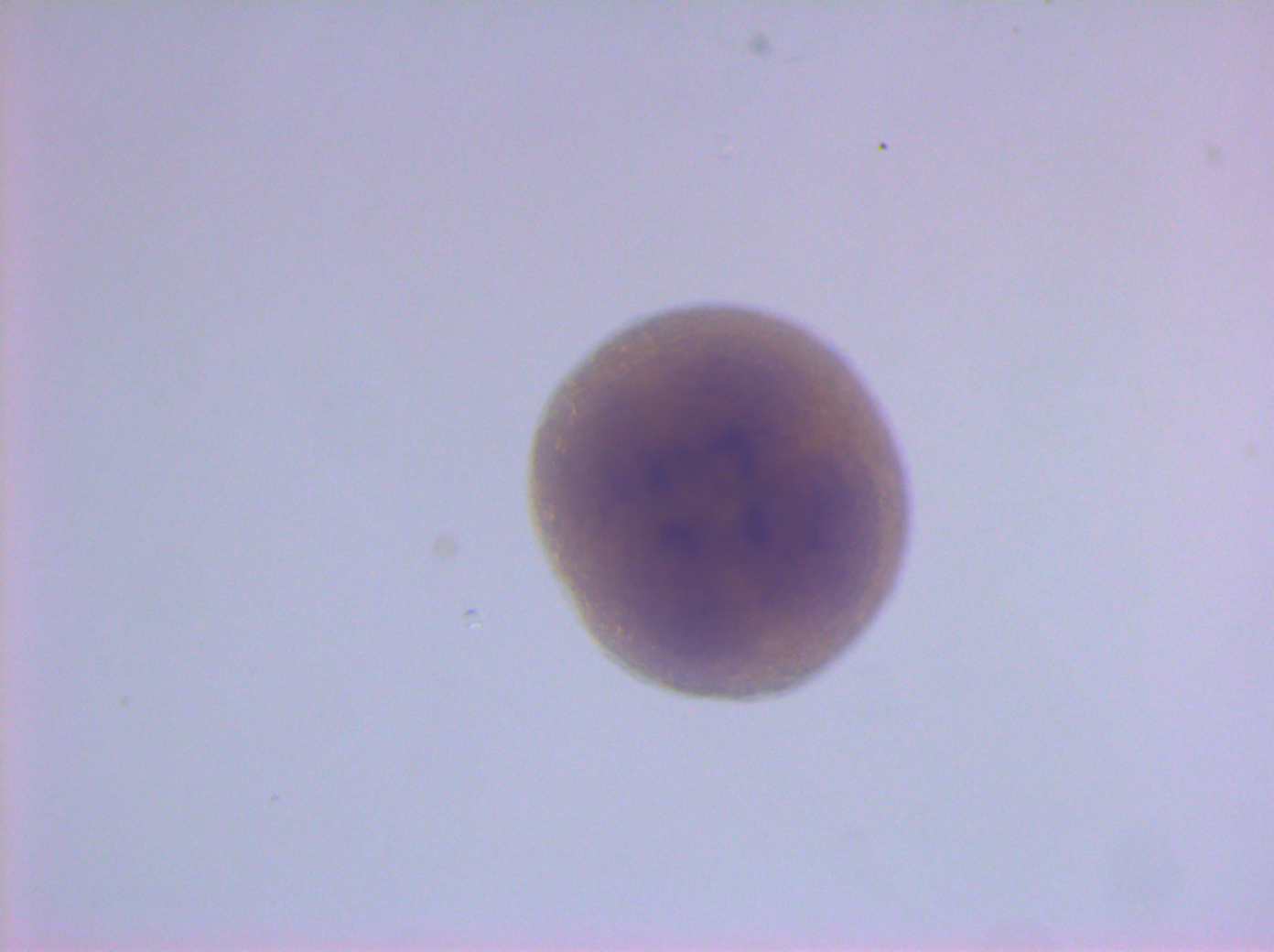

Supplement: Supplementary file 18 — Figure EV1 Source Data [file 44318_2025_442_MOESM18_ESM.zip › Figure_EV1/Figure EV1b/wt rbm24a 4 cell 0008.tif]

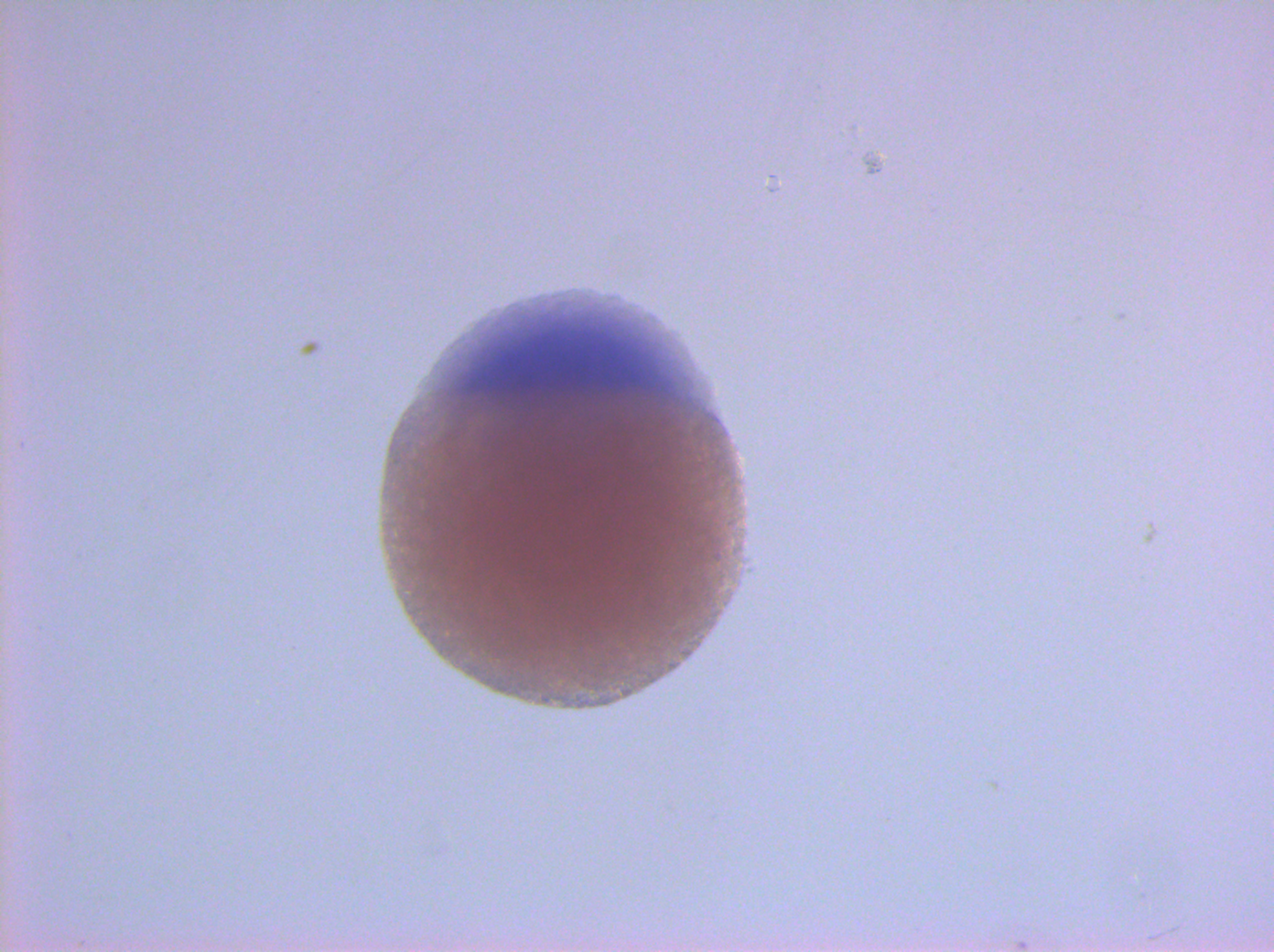

Supplement: Supplementary file 18 — Figure EV1 Source Data [file 44318_2025_442_MOESM18_ESM.zip › Figure_EV1/Figure EV1b/wt rbm24a sphere 00009.tif]

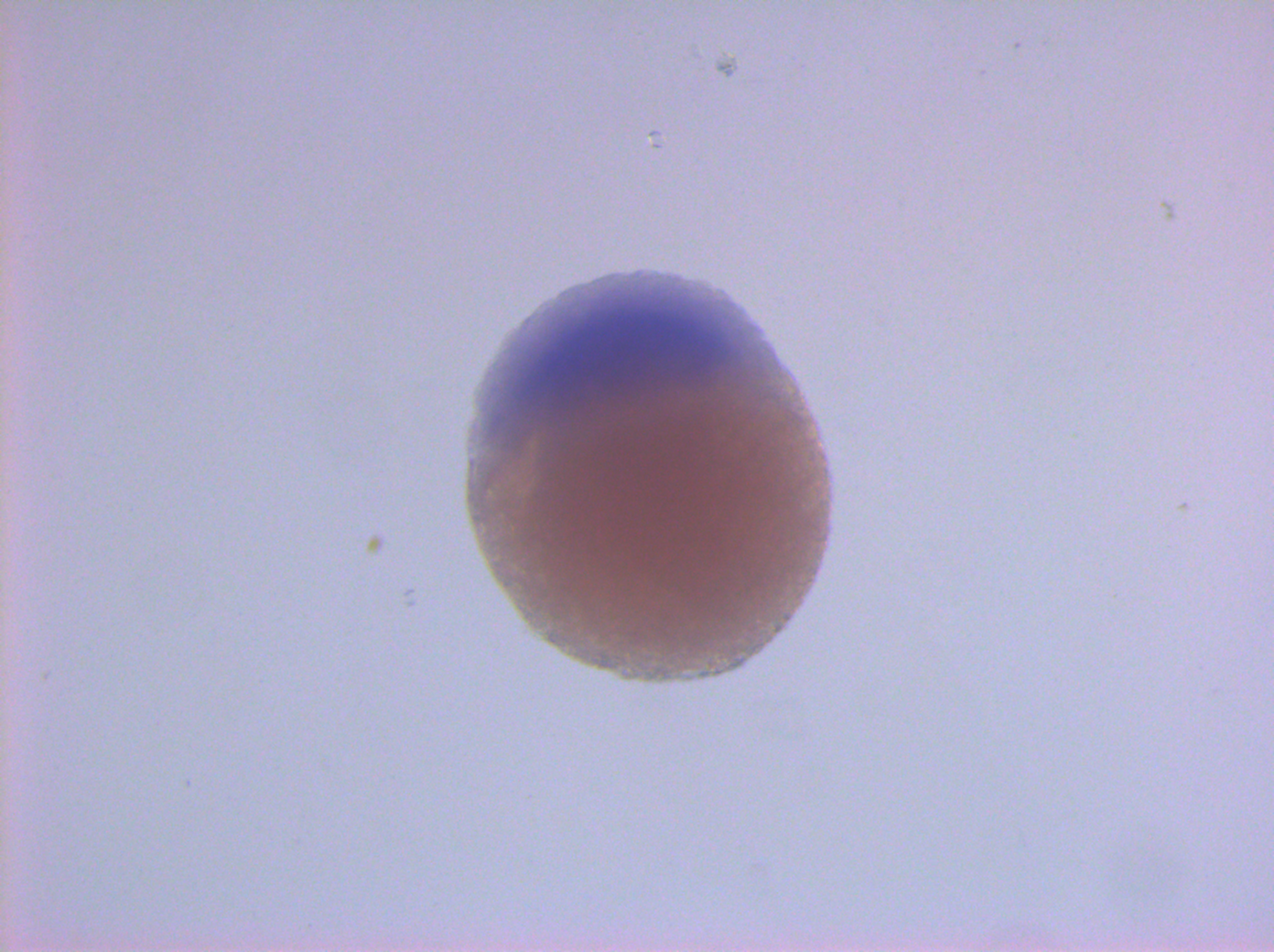

Supplement: Supplementary file 18 — Figure EV1 Source Data [file 44318_2025_442_MOESM18_ESM.zip › Figure_EV1/Figure EV1b/wt rbm24a sphere 0009.tif]

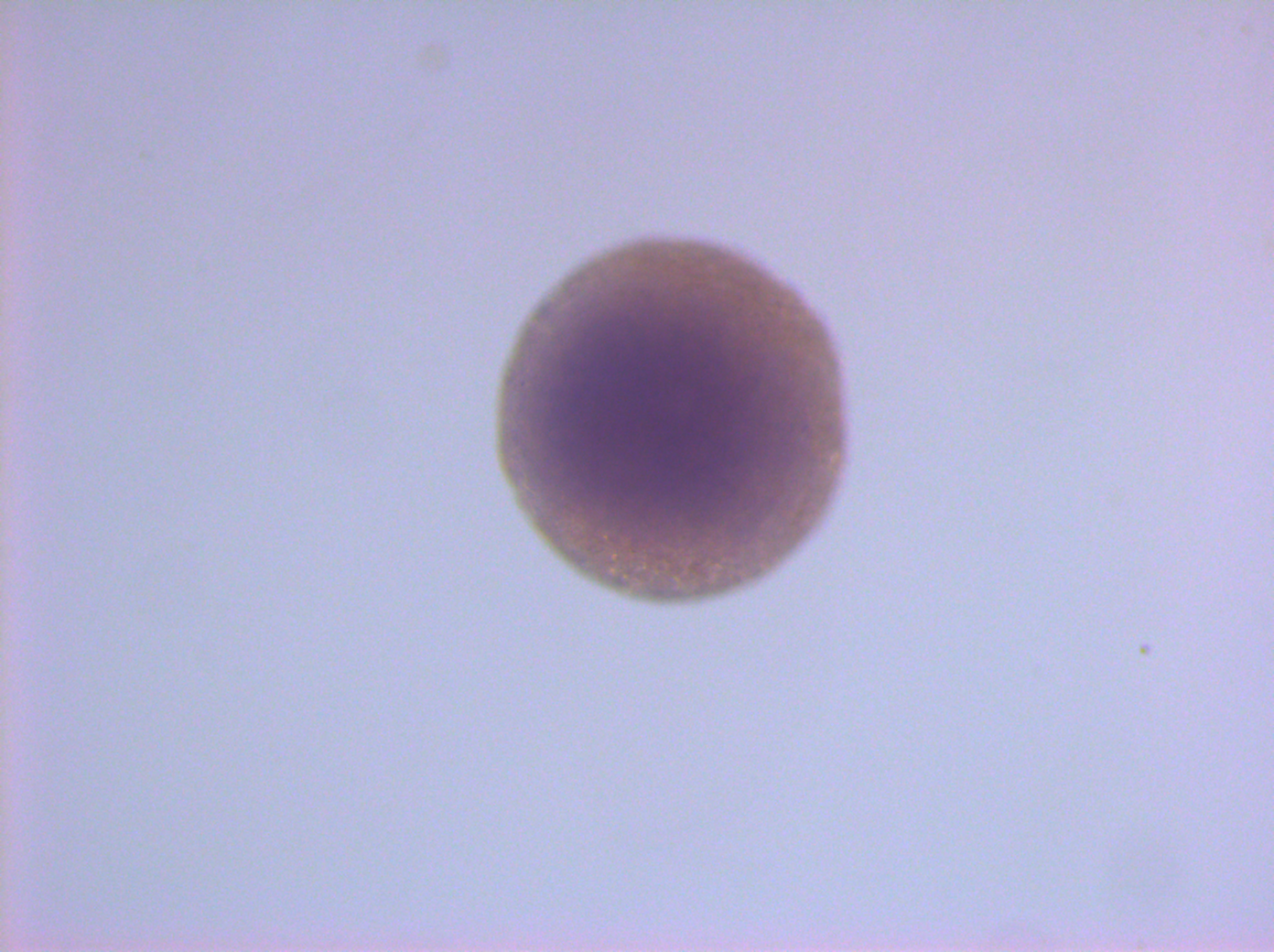

Supplement: Supplementary file 18 — Figure EV1 Source Data [file 44318_2025_442_MOESM18_ESM.zip › Figure_EV1/Figure EV1b/wt rbm24a sphere 0014.tif]

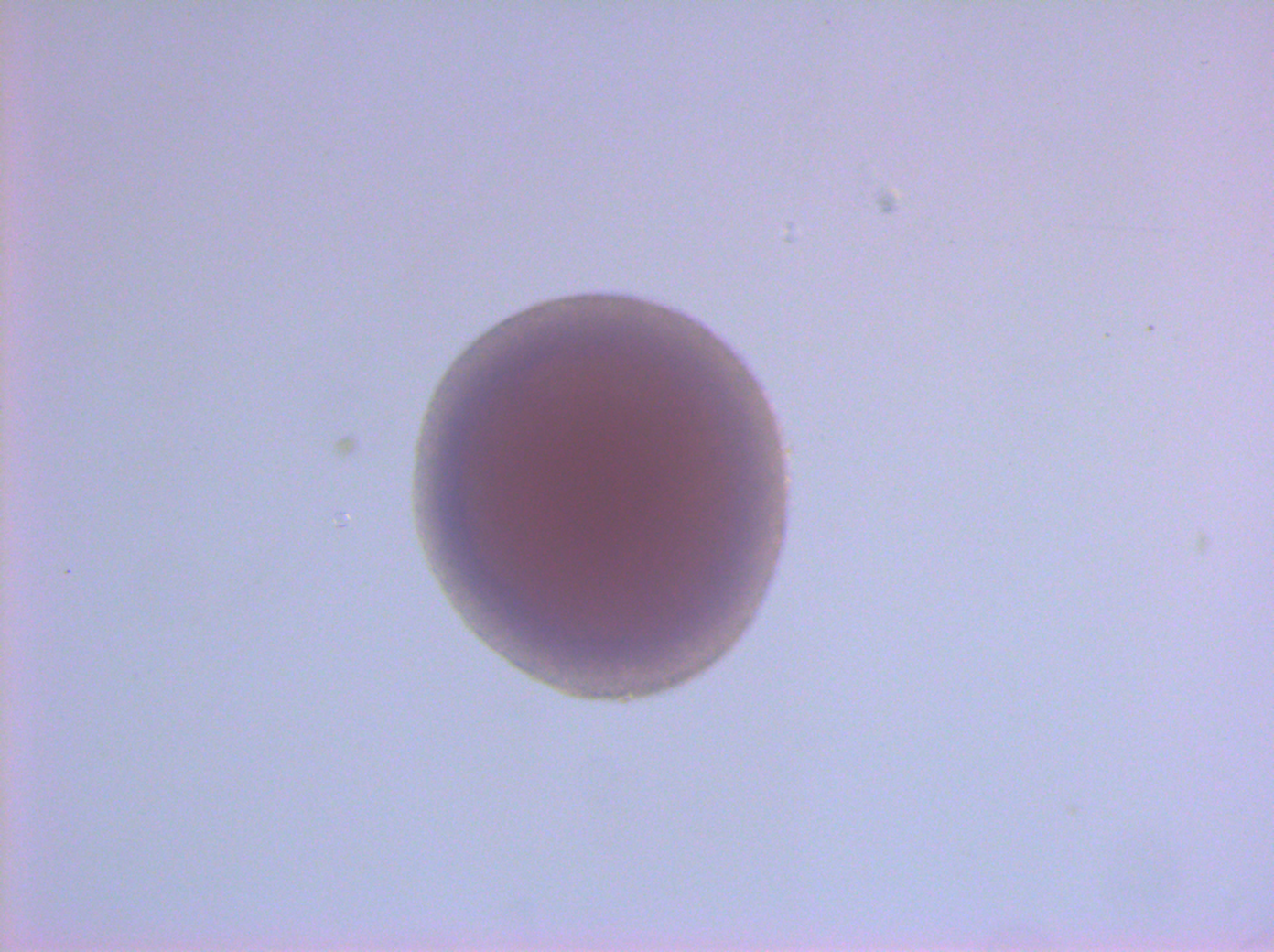

Supplement: Supplementary file 18 — Figure EV1 Source Data [file 44318_2025_442_MOESM18_ESM.zip › Figure_EV1/Figure EV1b/wt rbm24a50%0013.tif]

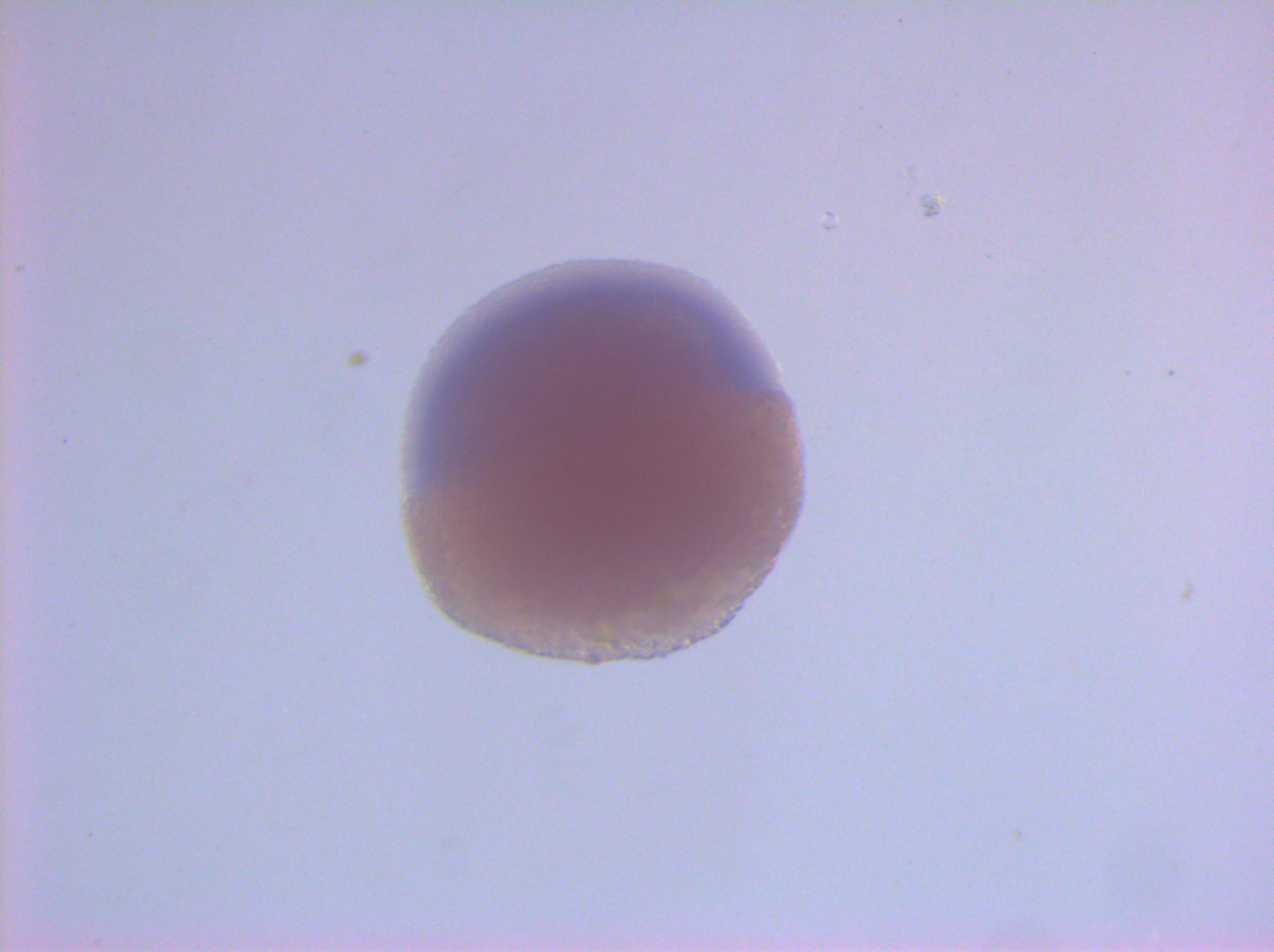

Supplement: Supplementary file 18 — Figure EV1 Source Data [file 44318_2025_442_MOESM18_ESM.zip › Figure_EV1/Figure EV1b/wt rbm24a50%0014.tif]

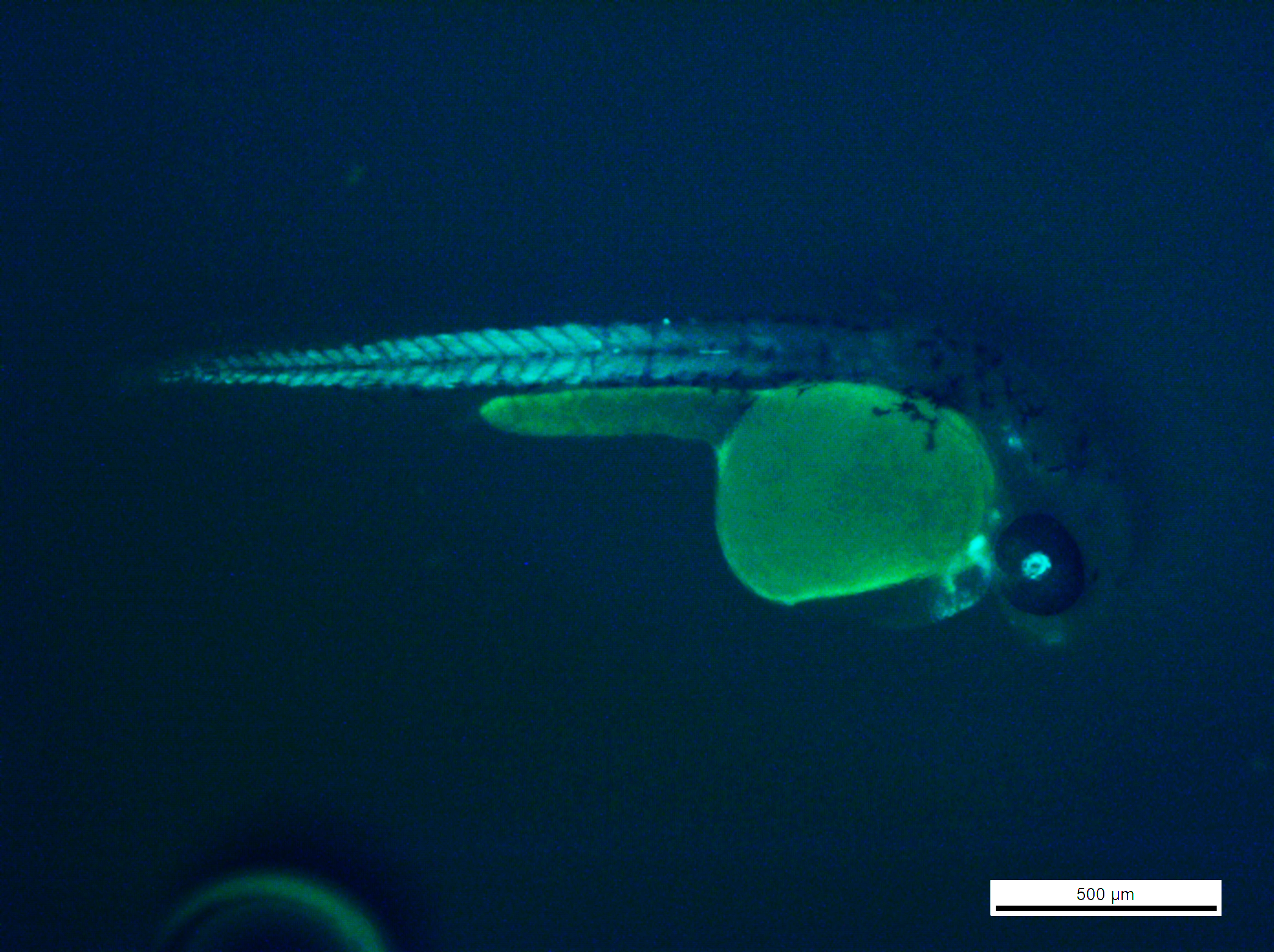

Supplement: Supplementary file 18 — Figure EV1 Source Data [file 44318_2025_442_MOESM18_ESM.zip › Figure_EV1/Figure EV1c/Extended Data Fig 1c.tif]

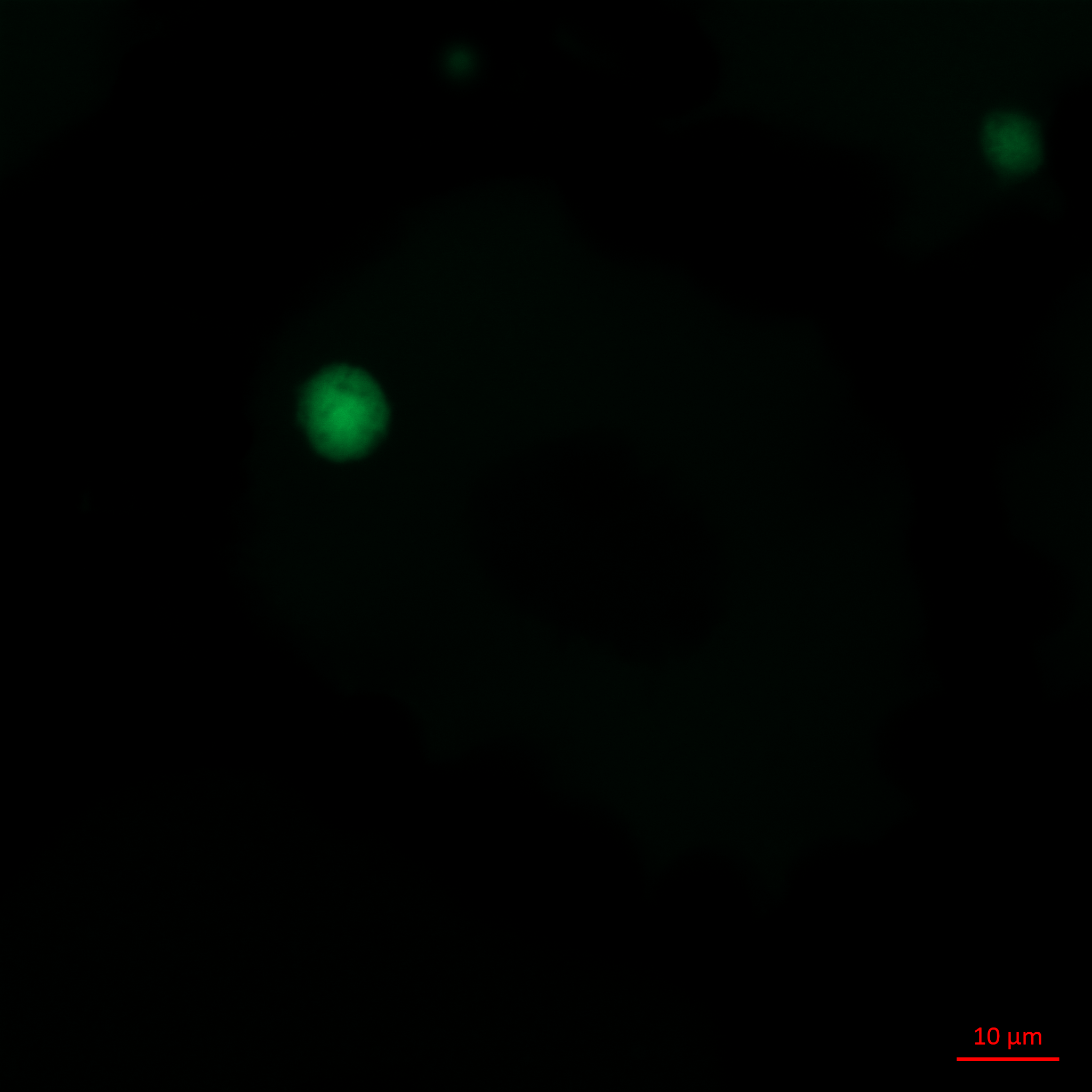

Supplement: Supplementary file 19 — Figure EV2 Source Data [file 44318_2025_442_MOESM19_ESM.zip › Figure_EV2/Figure EV2a/a Rbm24a-GFP.tif]

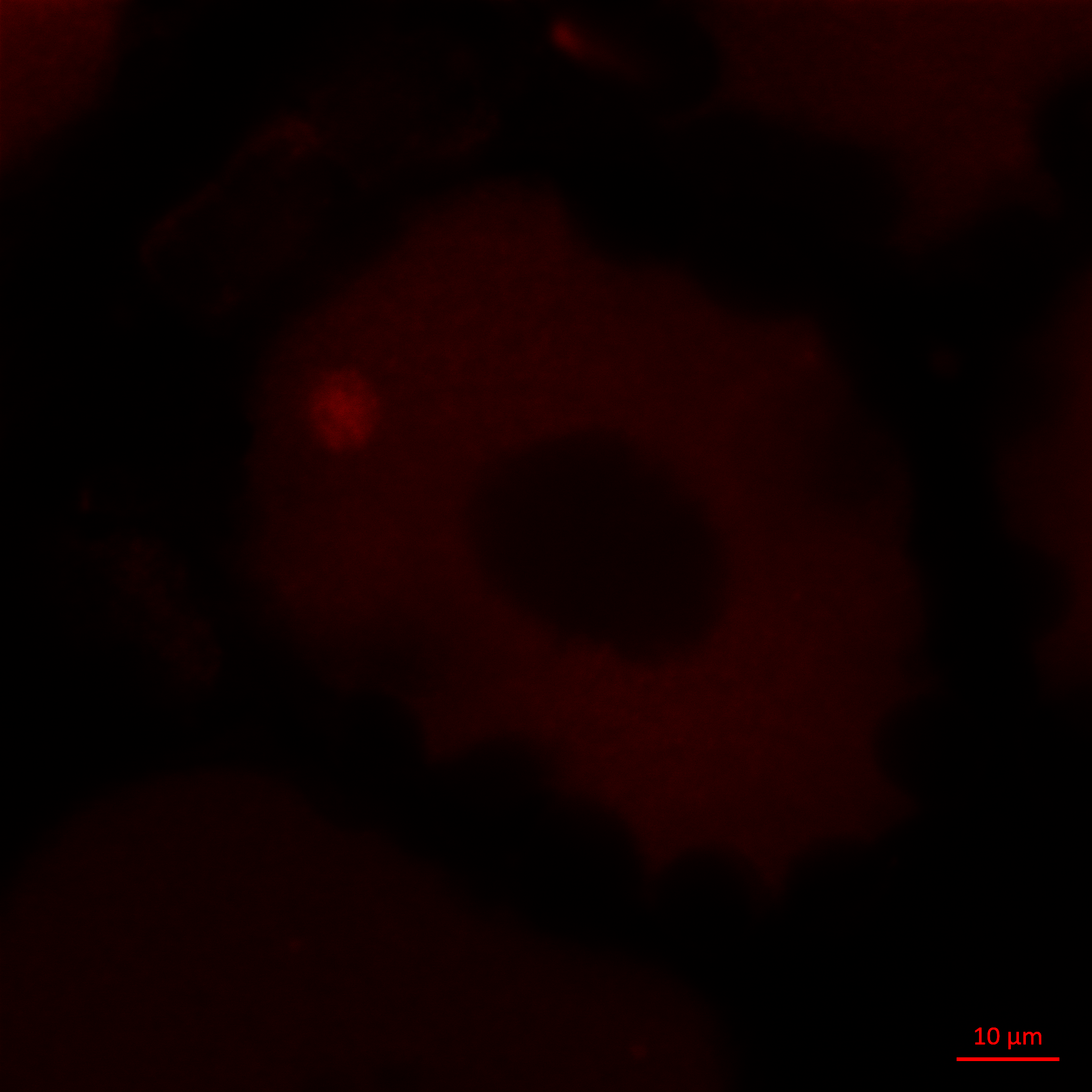

Supplement: Supplementary file 19 — Figure EV2 Source Data [file 44318_2025_442_MOESM19_ESM.zip › Figure_EV2/Figure EV2b/b ddx4.tif]

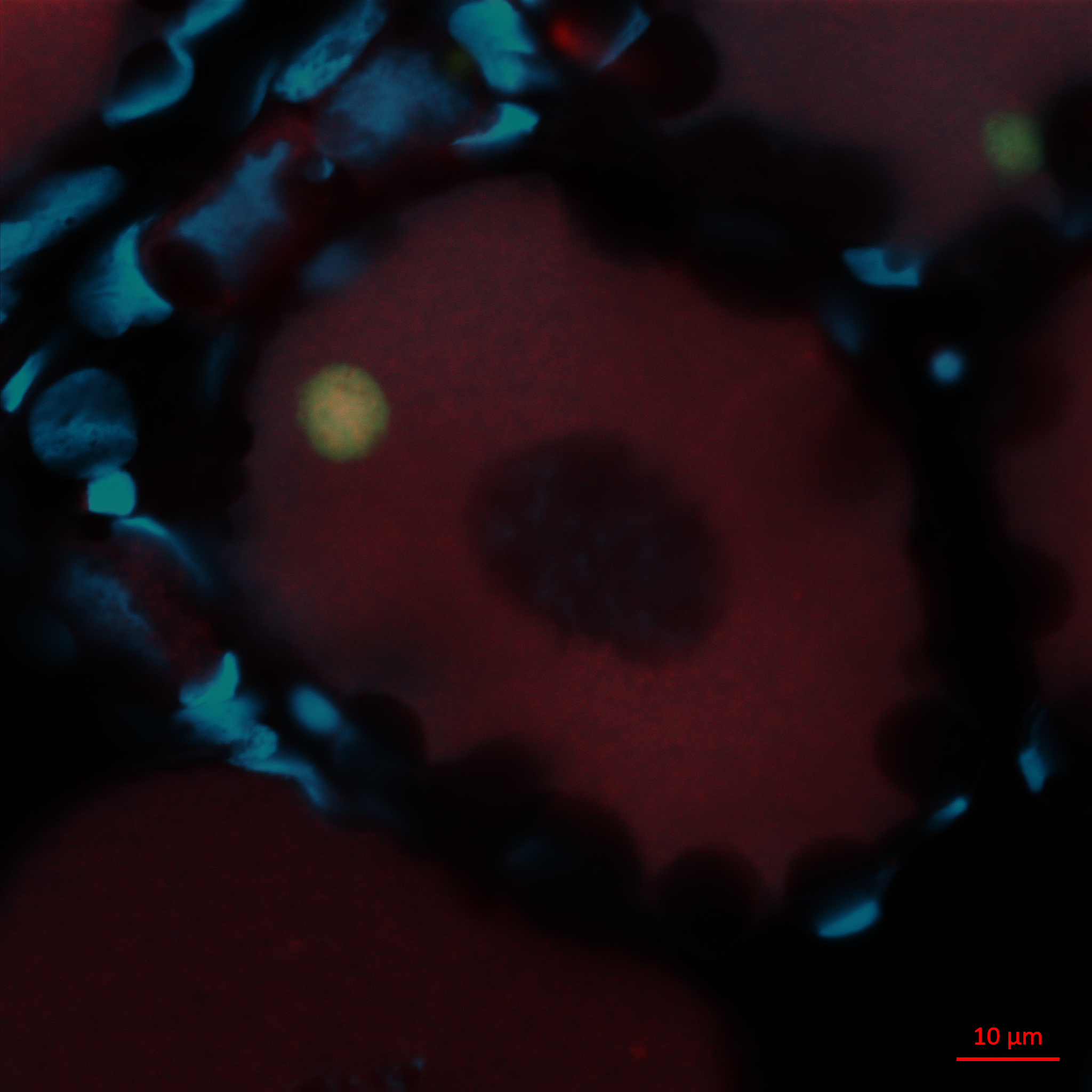

Supplement: Supplementary file 19 — Figure EV2 Source Data [file 44318_2025_442_MOESM19_ESM.zip › Figure_EV2/Figure EV2c/c merge.tif]

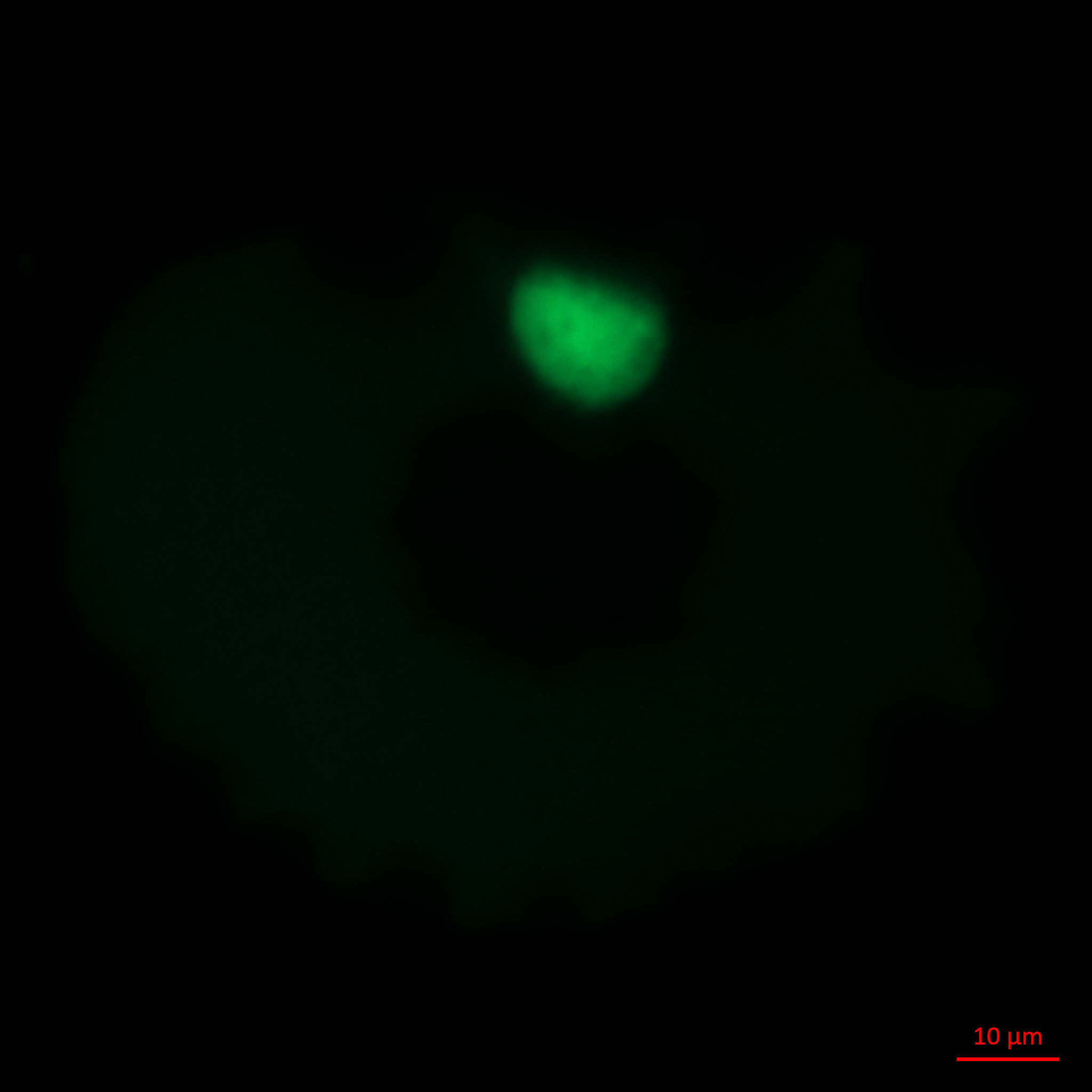

Supplement: Supplementary file 19 — Figure EV2 Source Data [file 44318_2025_442_MOESM19_ESM.zip › Figure_EV2/Figure EV2d/d Rbm24a-GFP.tif]

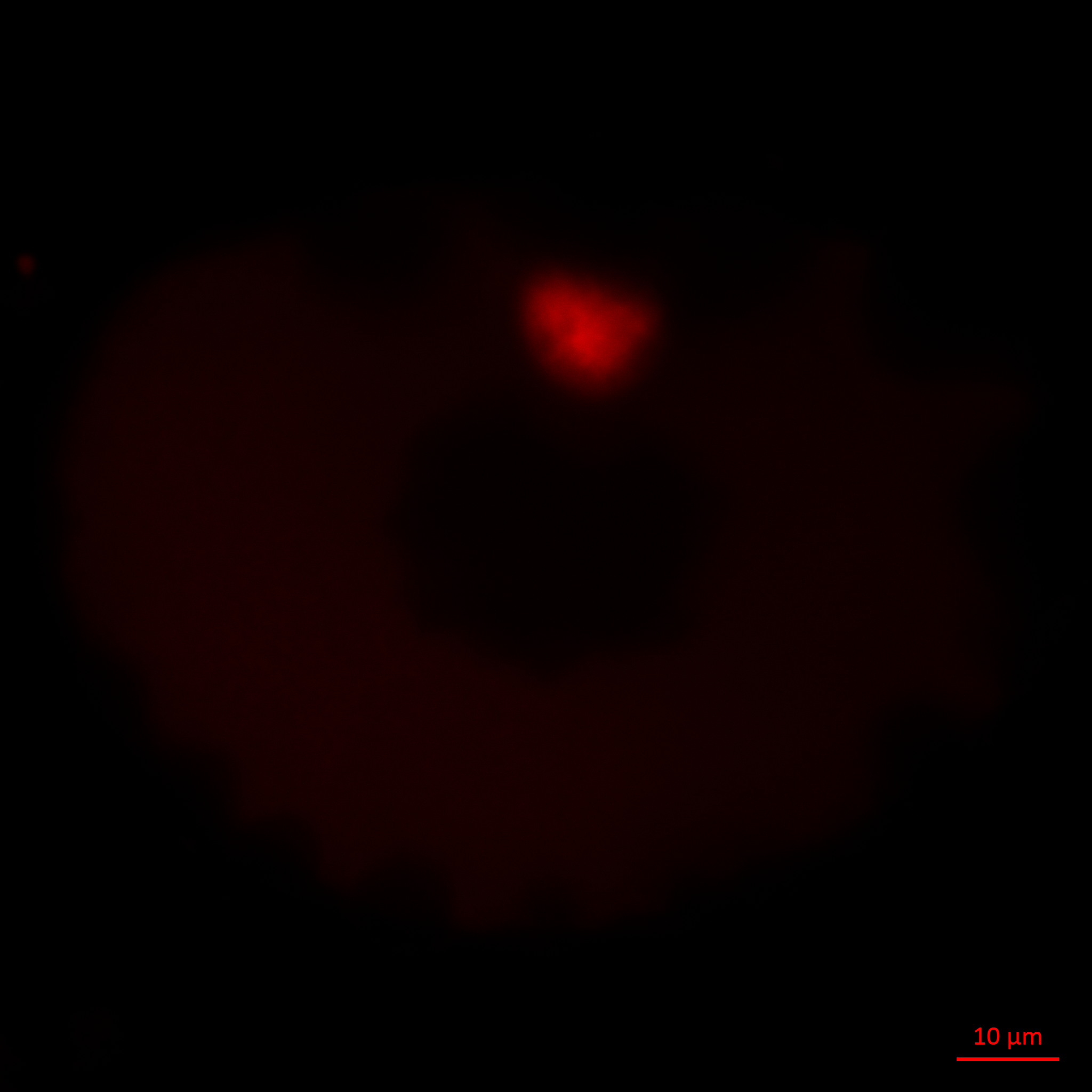

Supplement: Supplementary file 19 — Figure EV2 Source Data [file 44318_2025_442_MOESM19_ESM.zip › Figure_EV2/Figure EV2e/dazl.tif]

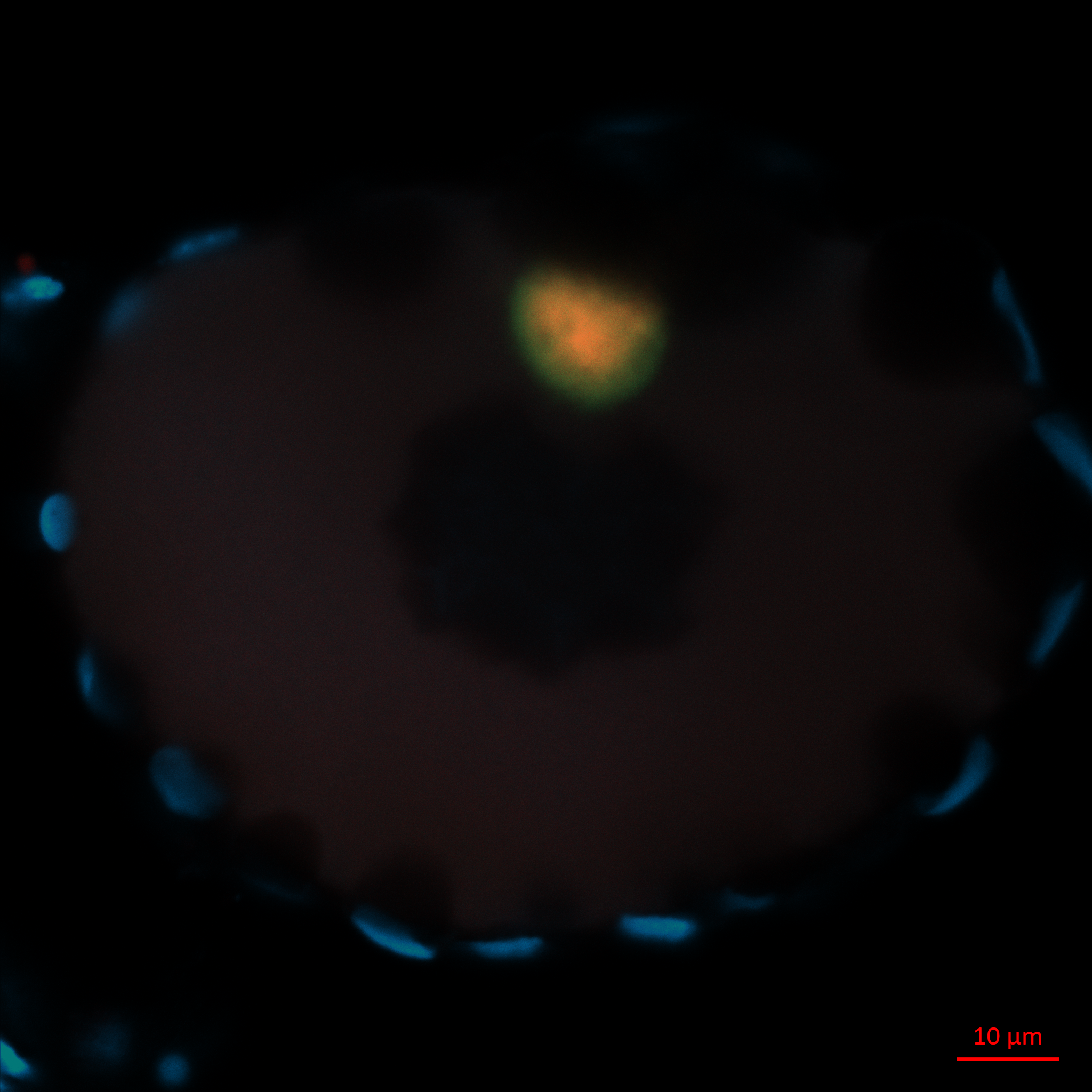

Supplement: Supplementary file 19 — Figure EV2 Source Data [file 44318_2025_442_MOESM19_ESM.zip › Figure_EV2/Figure EV2f/f.tif]

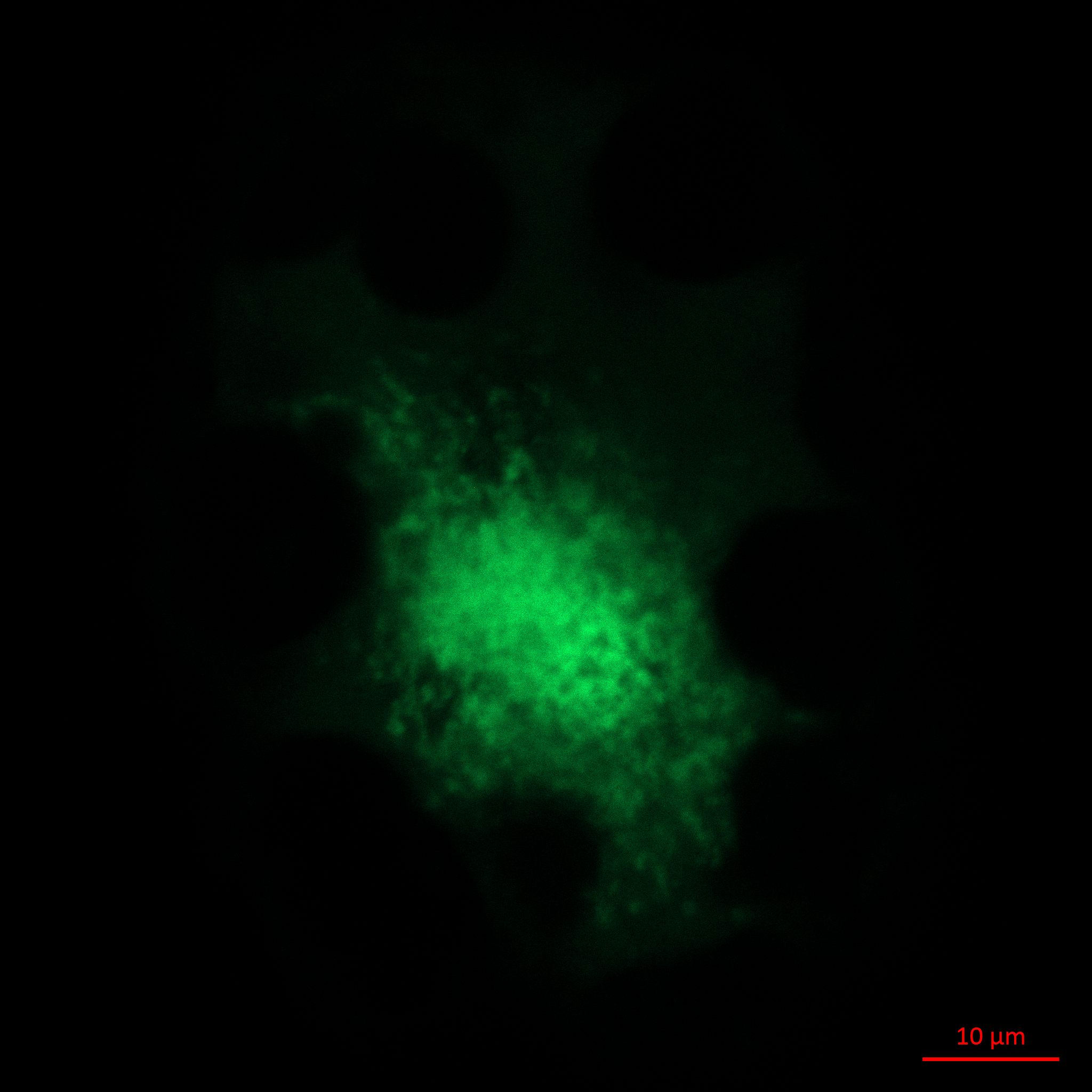

Supplement: Supplementary file 19 — Figure EV2 Source Data [file 44318_2025_442_MOESM19_ESM.zip › Figure_EV2/Figure EV2g/g Rbm24a-GFP.tif]

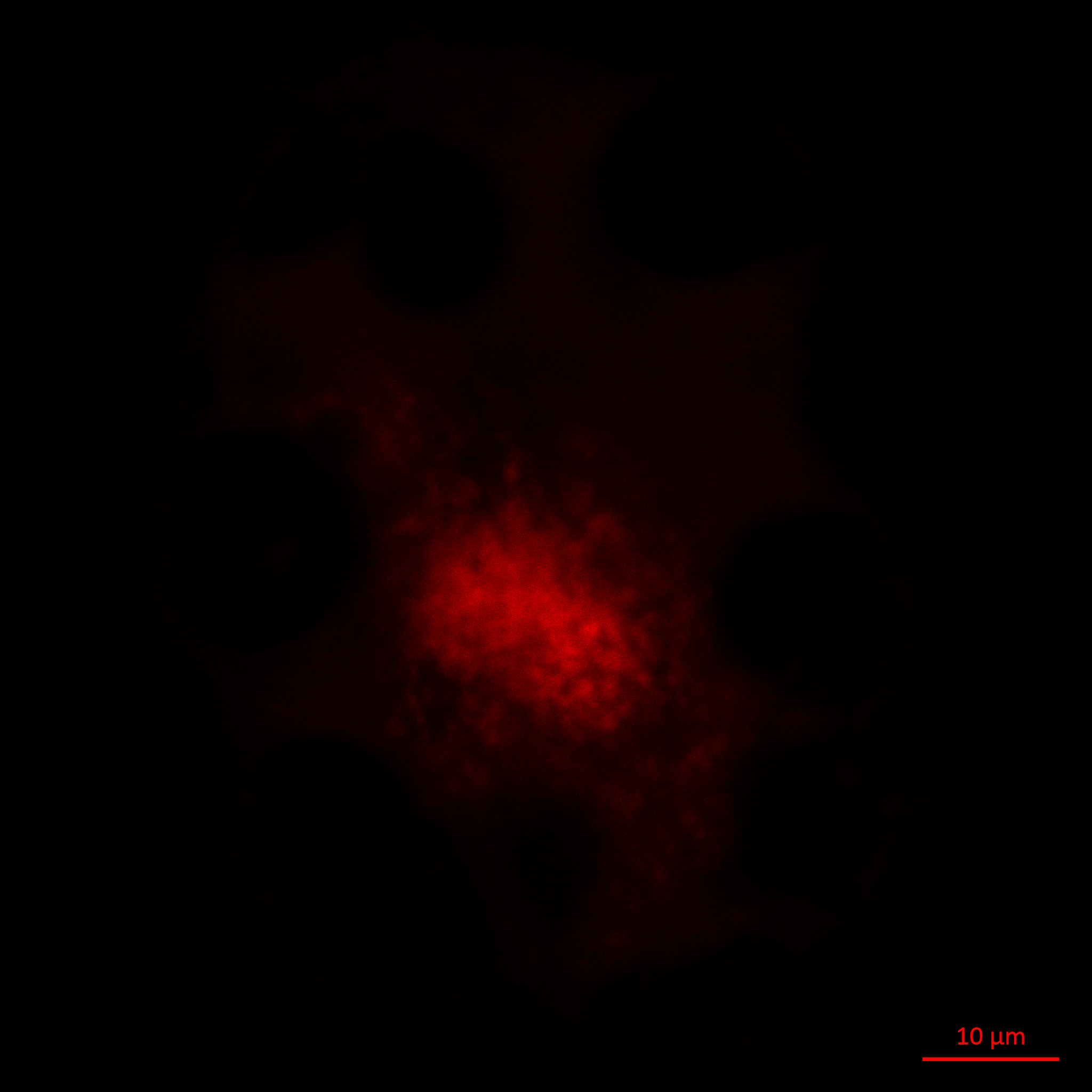

Supplement: Supplementary file 19 — Figure EV2 Source Data [file 44318_2025_442_MOESM19_ESM.zip › Figure_EV2/Figure EV2h/h dazl.tif]

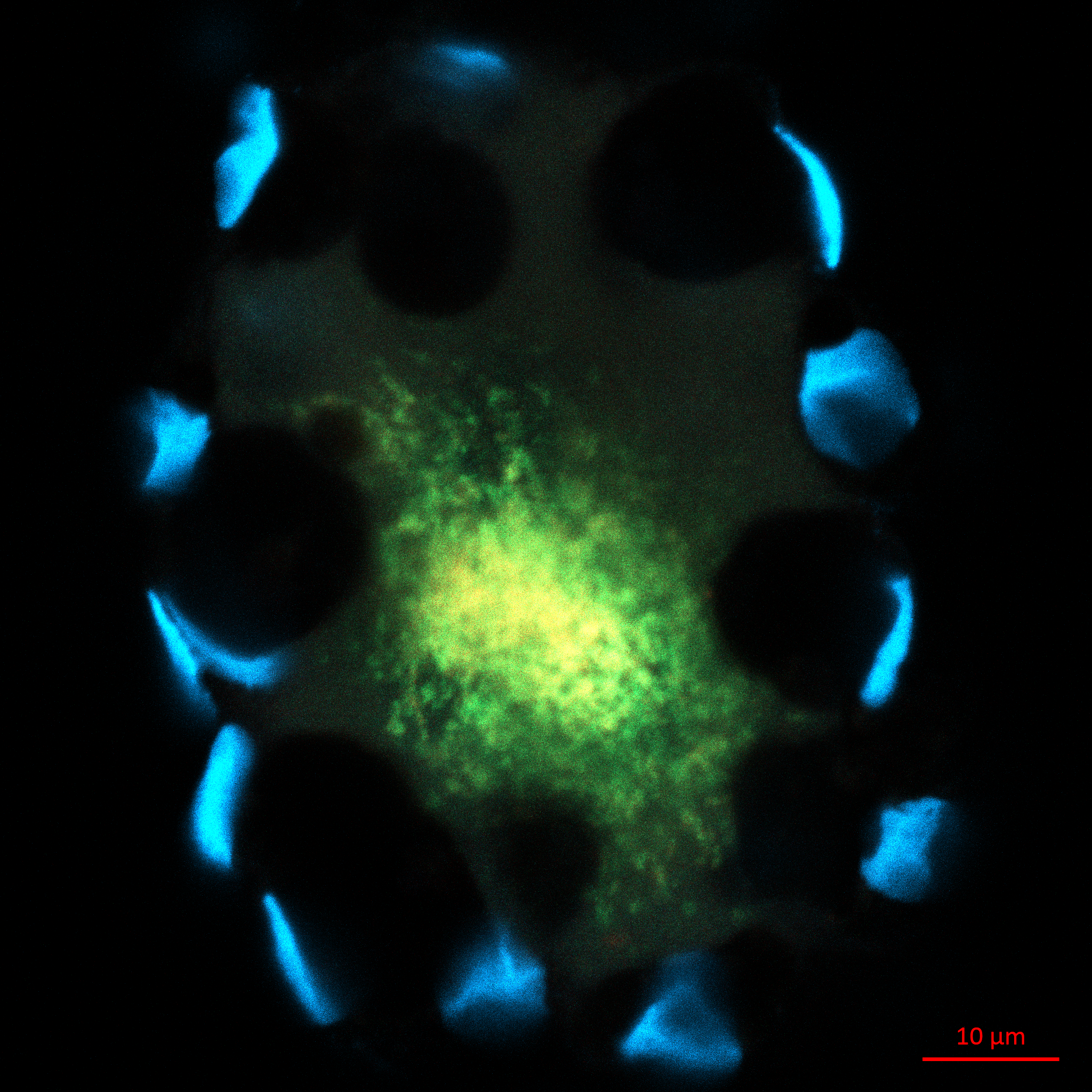

Supplement: Supplementary file 19 — Figure EV2 Source Data [file 44318_2025_442_MOESM19_ESM.zip › Figure_EV2/Figure EV2i/i.tif]

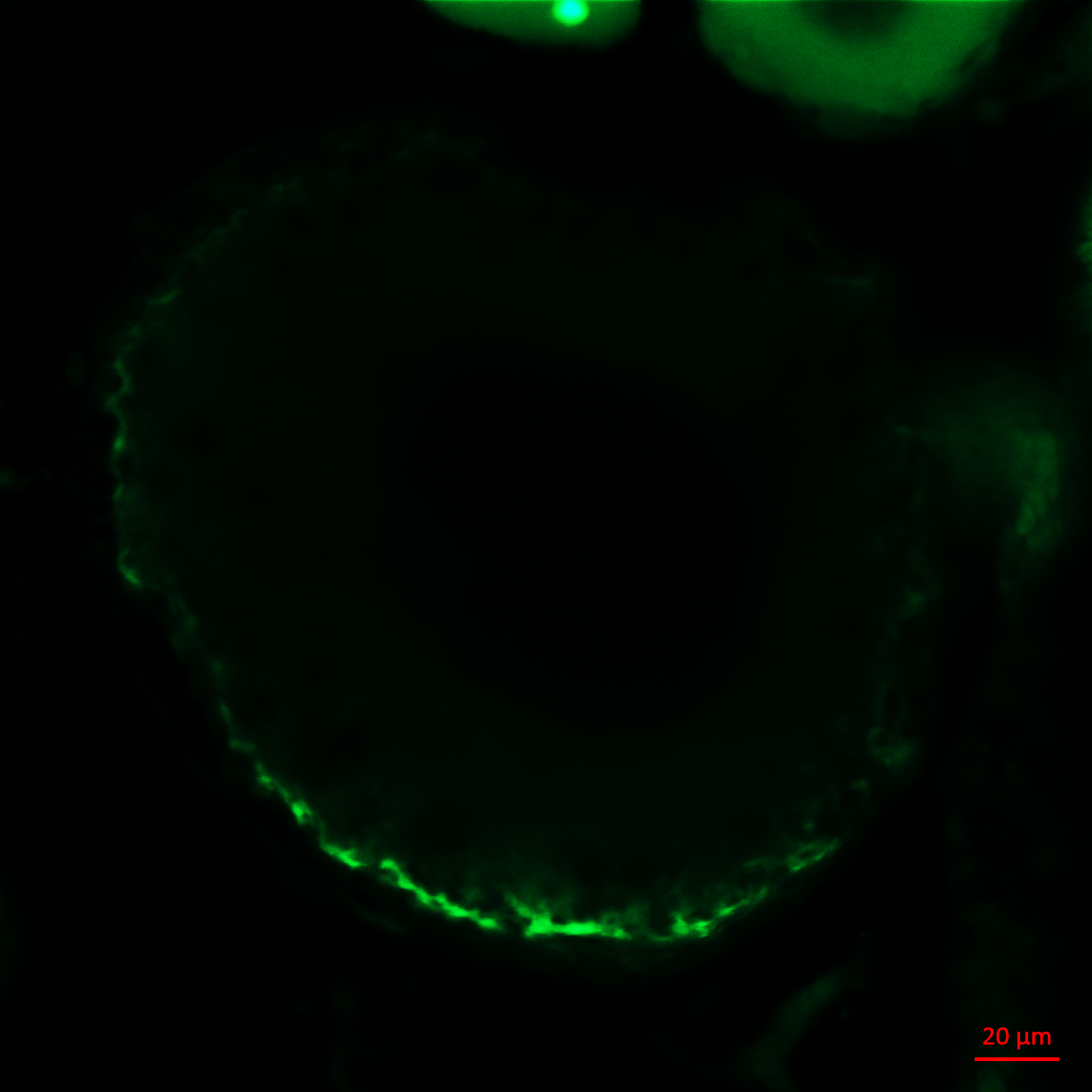

Supplement: Supplementary file 19 — Figure EV2 Source Data [file 44318_2025_442_MOESM19_ESM.zip › Figure_EV2/Figure EV2j/j Rbm24a-GFP.tif]

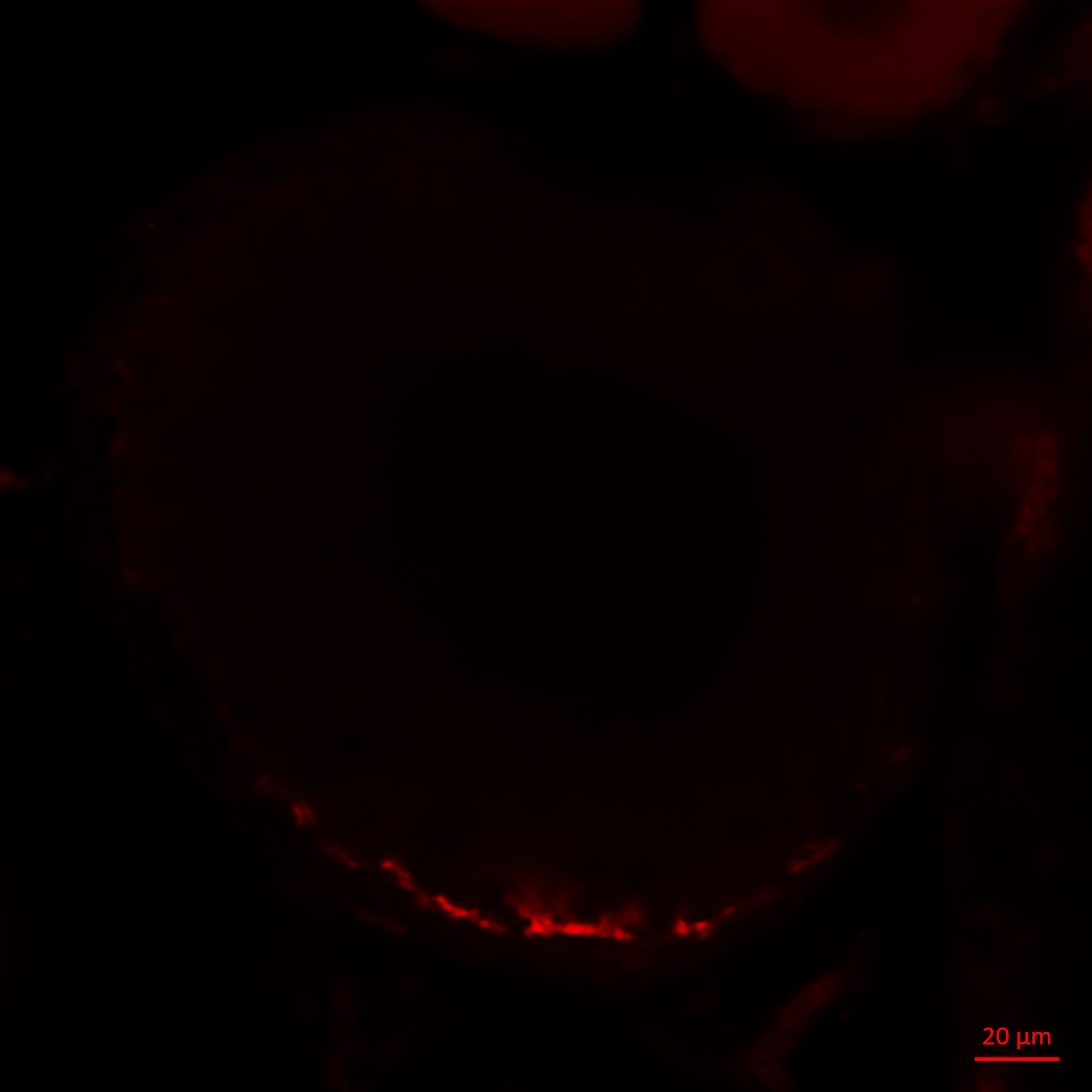

Supplement: Supplementary file 19 — Figure EV2 Source Data [file 44318_2025_442_MOESM19_ESM.zip › Figure_EV2/Figure EV2k/k dazl.tif]

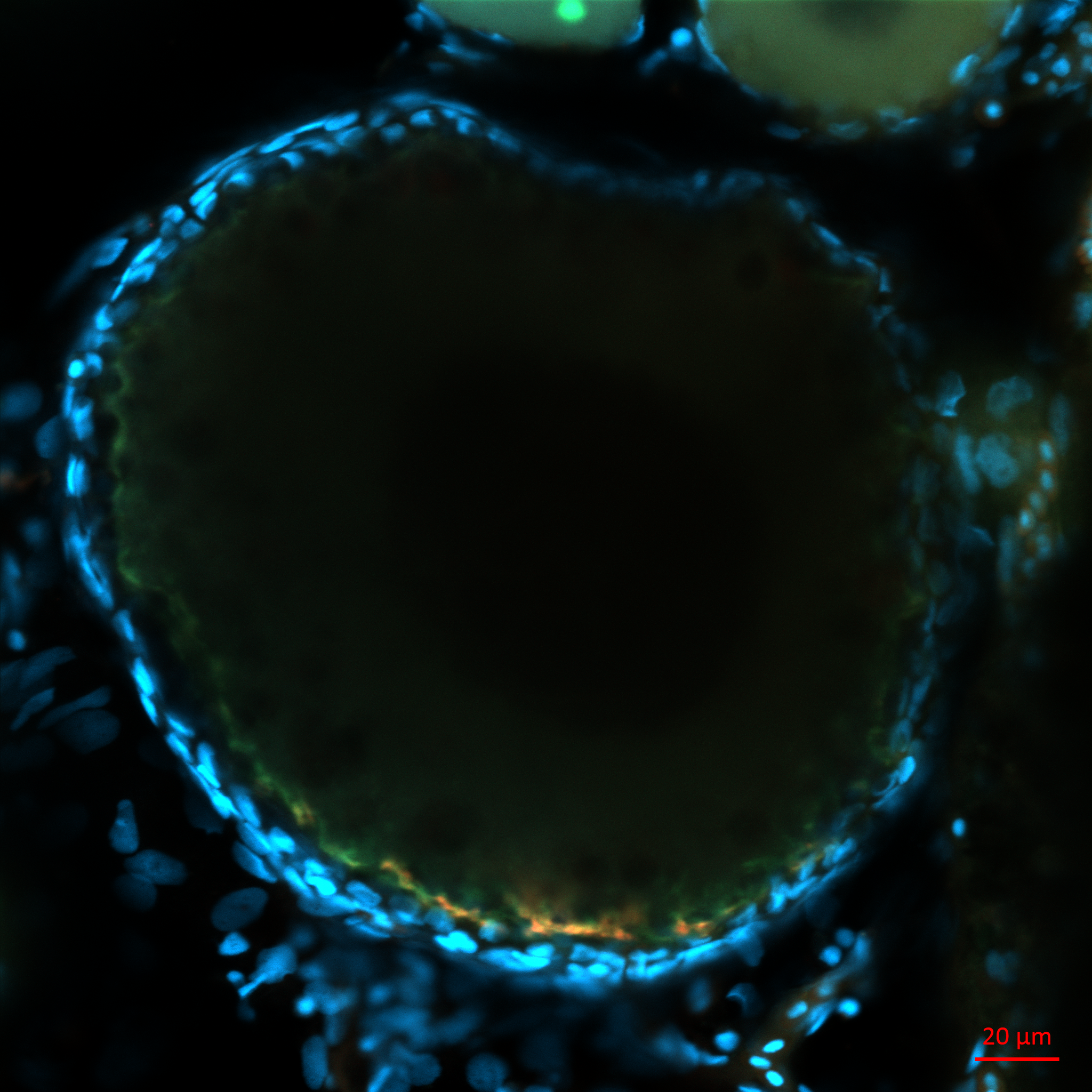

Supplement: Supplementary file 19 — Figure EV2 Source Data [file 44318_2025_442_MOESM19_ESM.zip › Figure_EV2/Figure EV2l/l merge.tif]

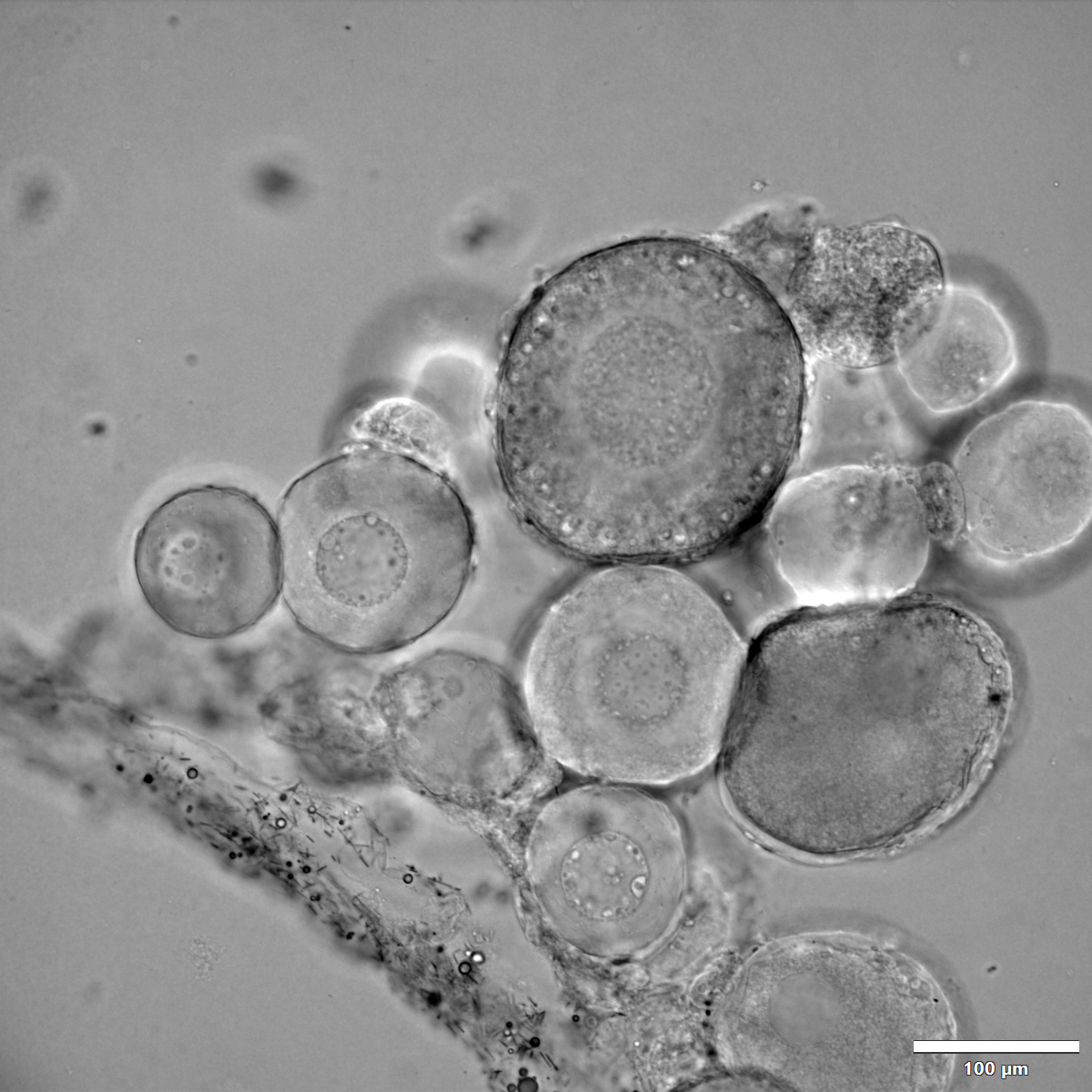

Supplement: Supplementary file 19 — Figure EV2 Source Data [file 44318_2025_442_MOESM19_ESM.zip › Figure_EV2/Figure EV2m/m oocyte DIC.tif]

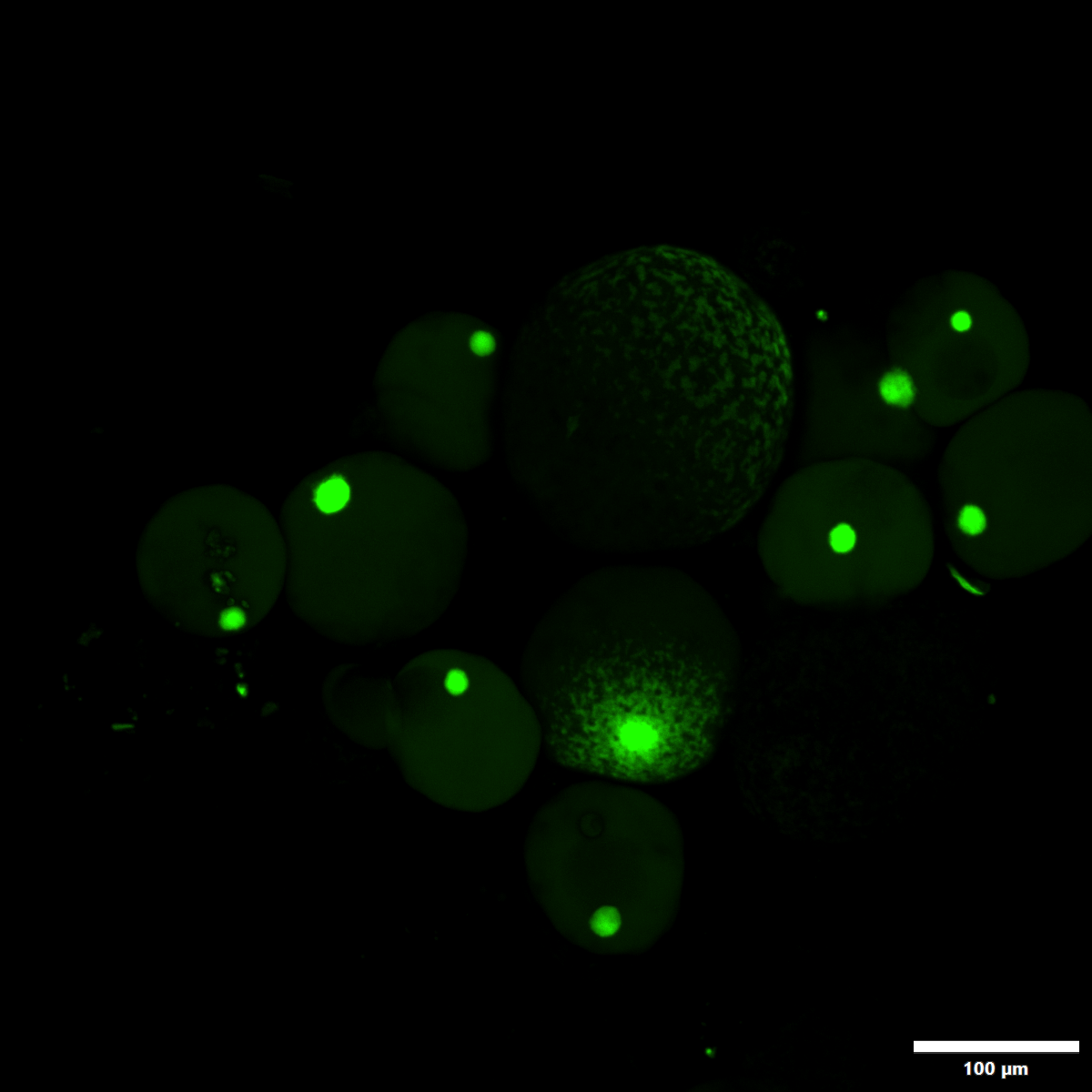

Supplement: Supplementary file 19 — Figure EV2 Source Data [file 44318_2025_442_MOESM19_ESM.zip › Figure_EV2/Figure EV2n/n oocyte Rbm24a-GFP .tif]

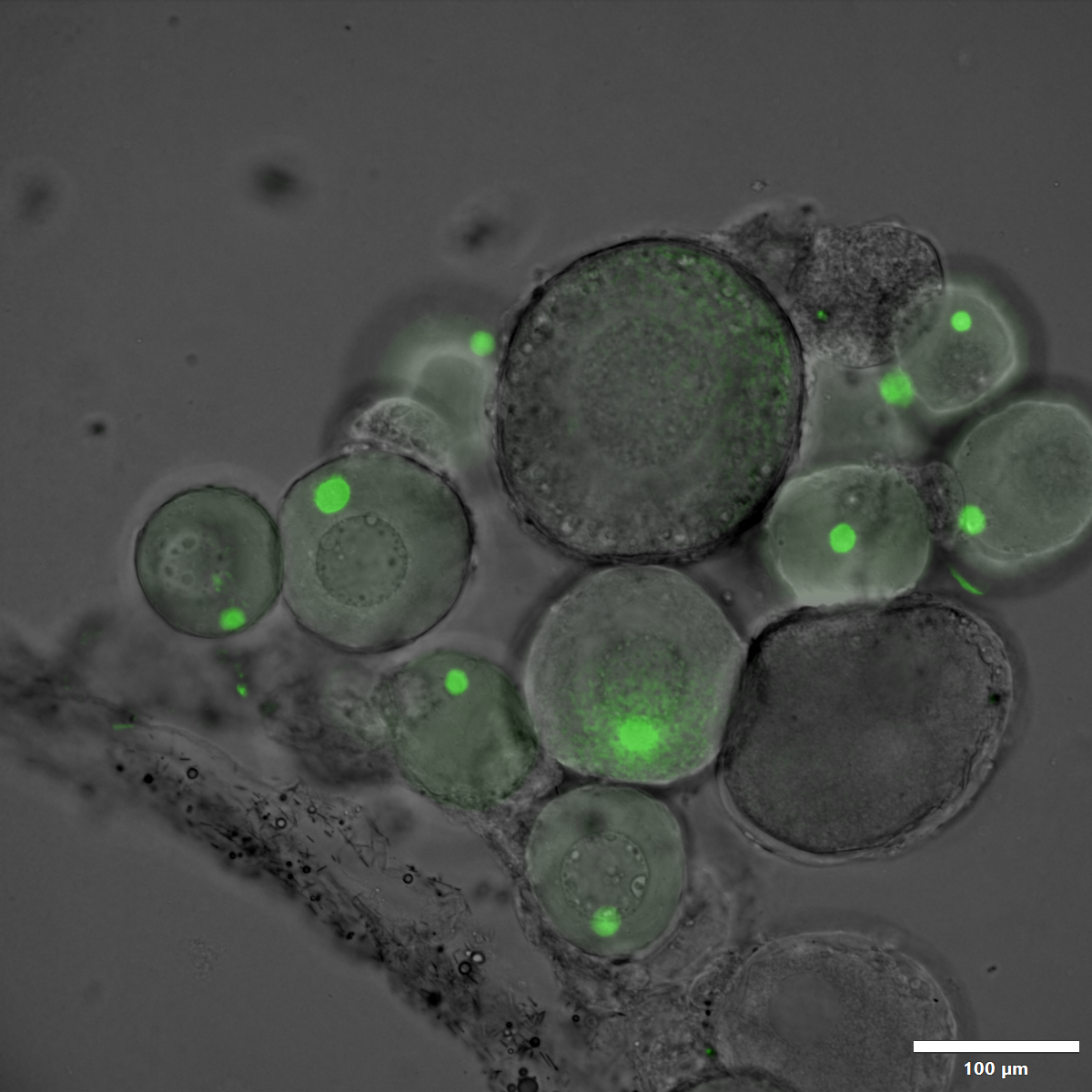

Supplement: Supplementary file 19 — Figure EV2 Source Data [file 44318_2025_442_MOESM19_ESM.zip › Figure_EV2/Figure EV2o/o oocyte Merge .tif]

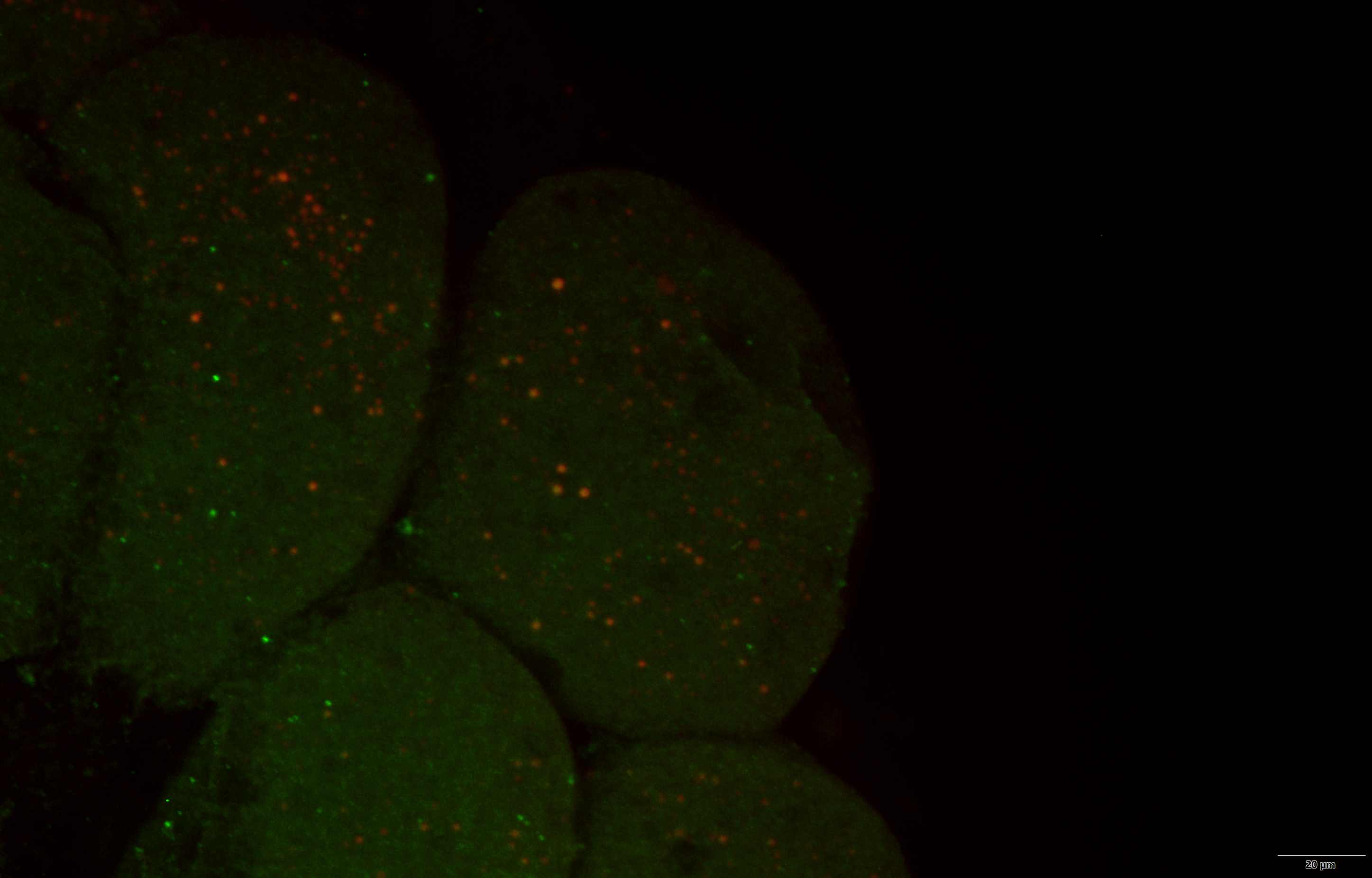

Supplement: Supplementary file 20 — Figure EV3 Source Data [file 44318_2025_442_MOESM20_ESM.zip › Figure_EV3/Figure EV3a/Extended Data Fig 5a M celf1-myc and piwil.tif]

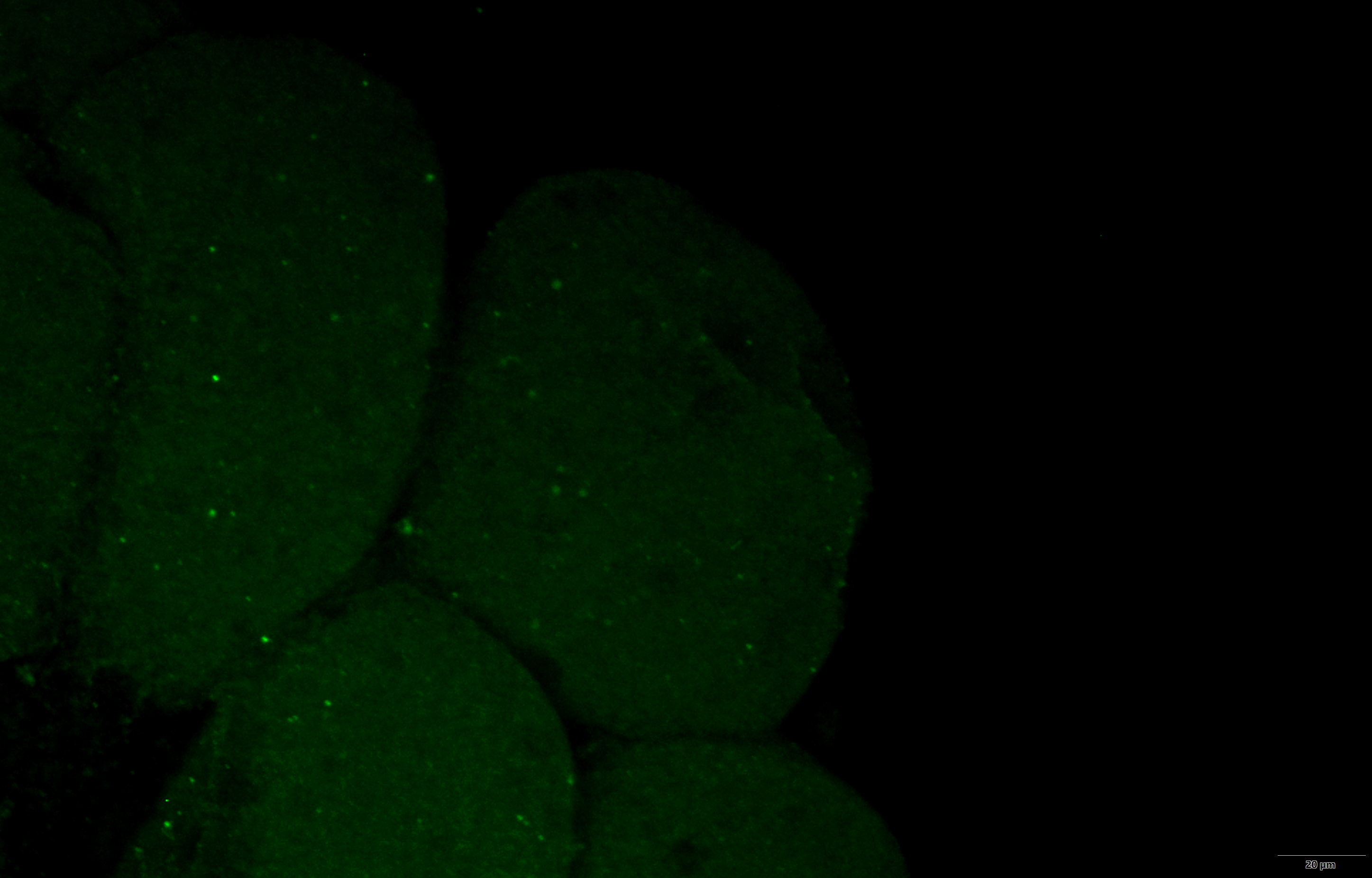

Supplement: Supplementary file 20 — Figure EV3 Source Data [file 44318_2025_442_MOESM20_ESM.zip › Figure_EV3/Figure EV3a/M celf1-myc.tif]

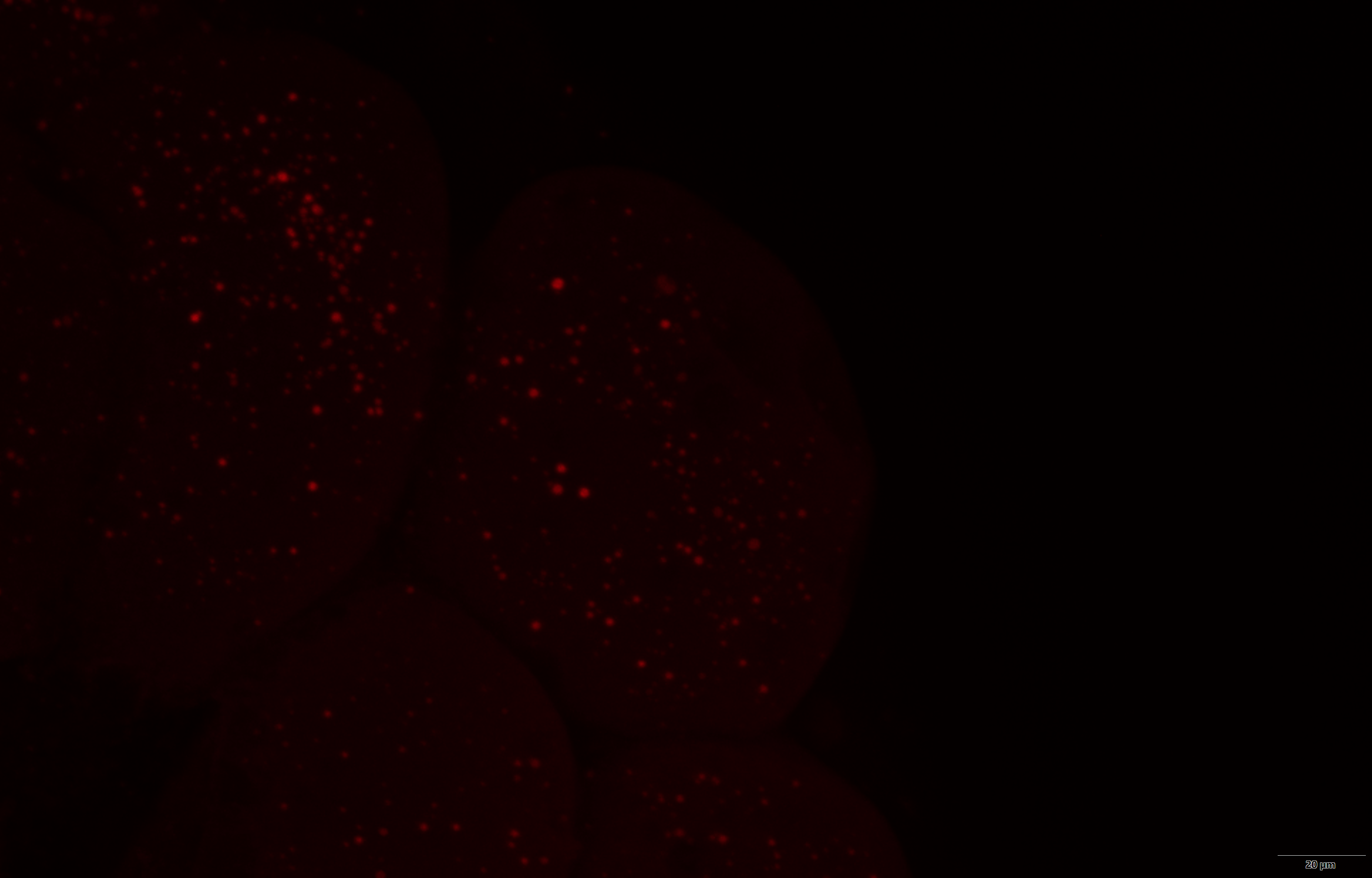

Supplement: Supplementary file 20 — Figure EV3 Source Data [file 44318_2025_442_MOESM20_ESM.zip › Figure_EV3/Figure EV3a/M piwil.tif]

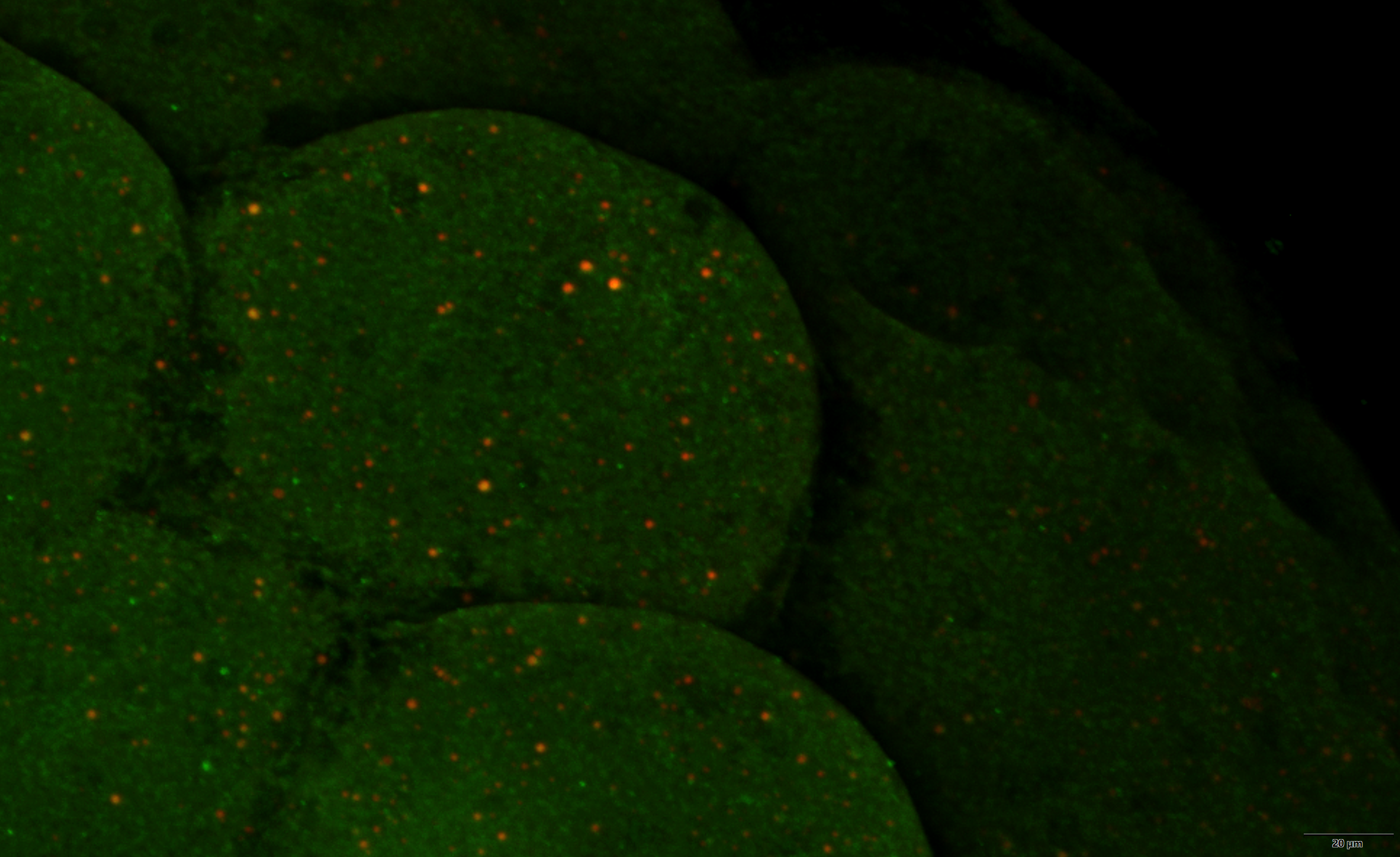

Supplement: Supplementary file 20 — Figure EV3 Source Data [file 44318_2025_442_MOESM20_ESM.zip › Figure_EV3/Figure EV3a/M tdrd6-myc and piwil.tif]

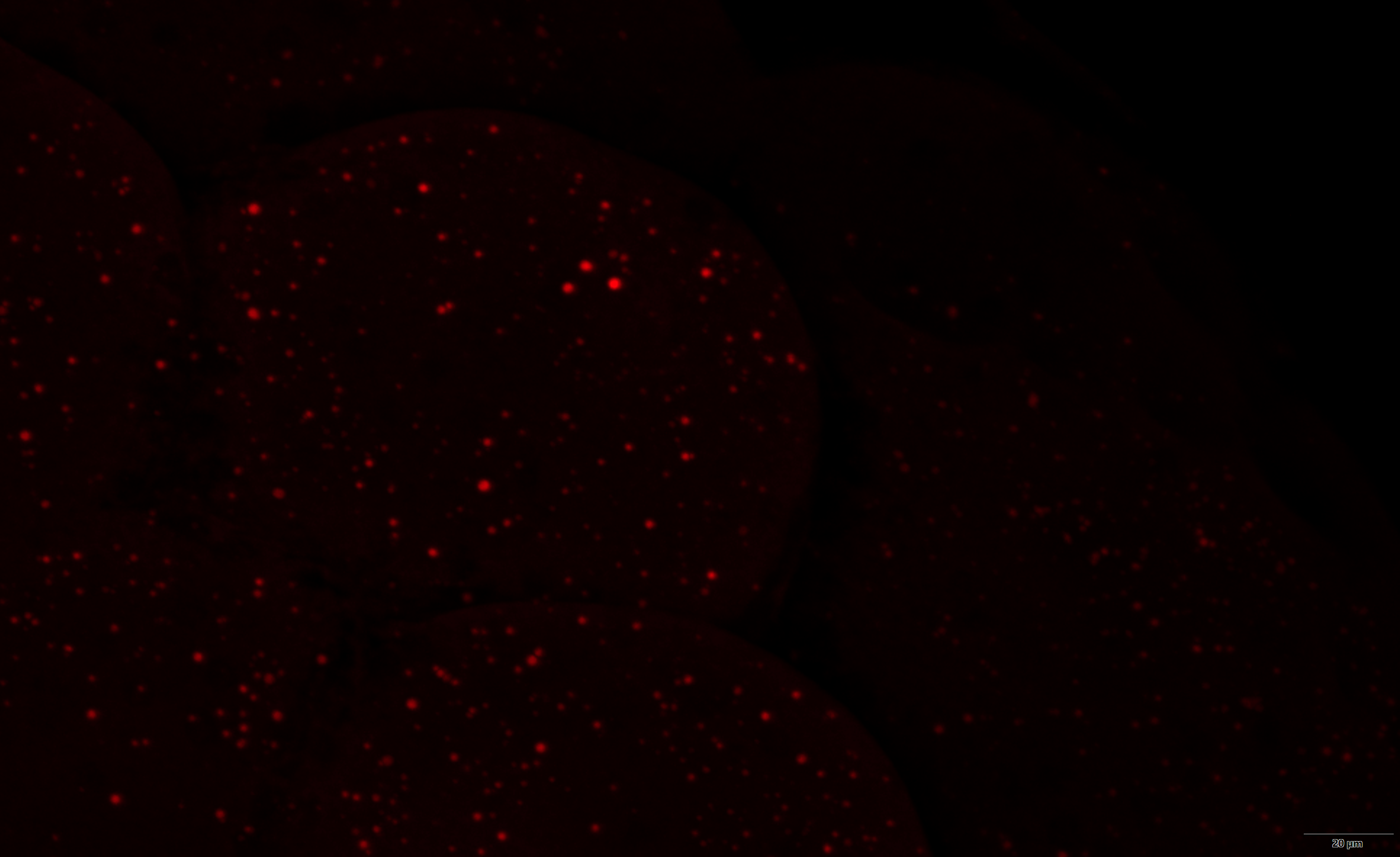

Supplement: Supplementary file 20 — Figure EV3 Source Data [file 44318_2025_442_MOESM20_ESM.zip › Figure_EV3/Figure EV3a/M tdrd6-myc rbm24a piwil.tif]

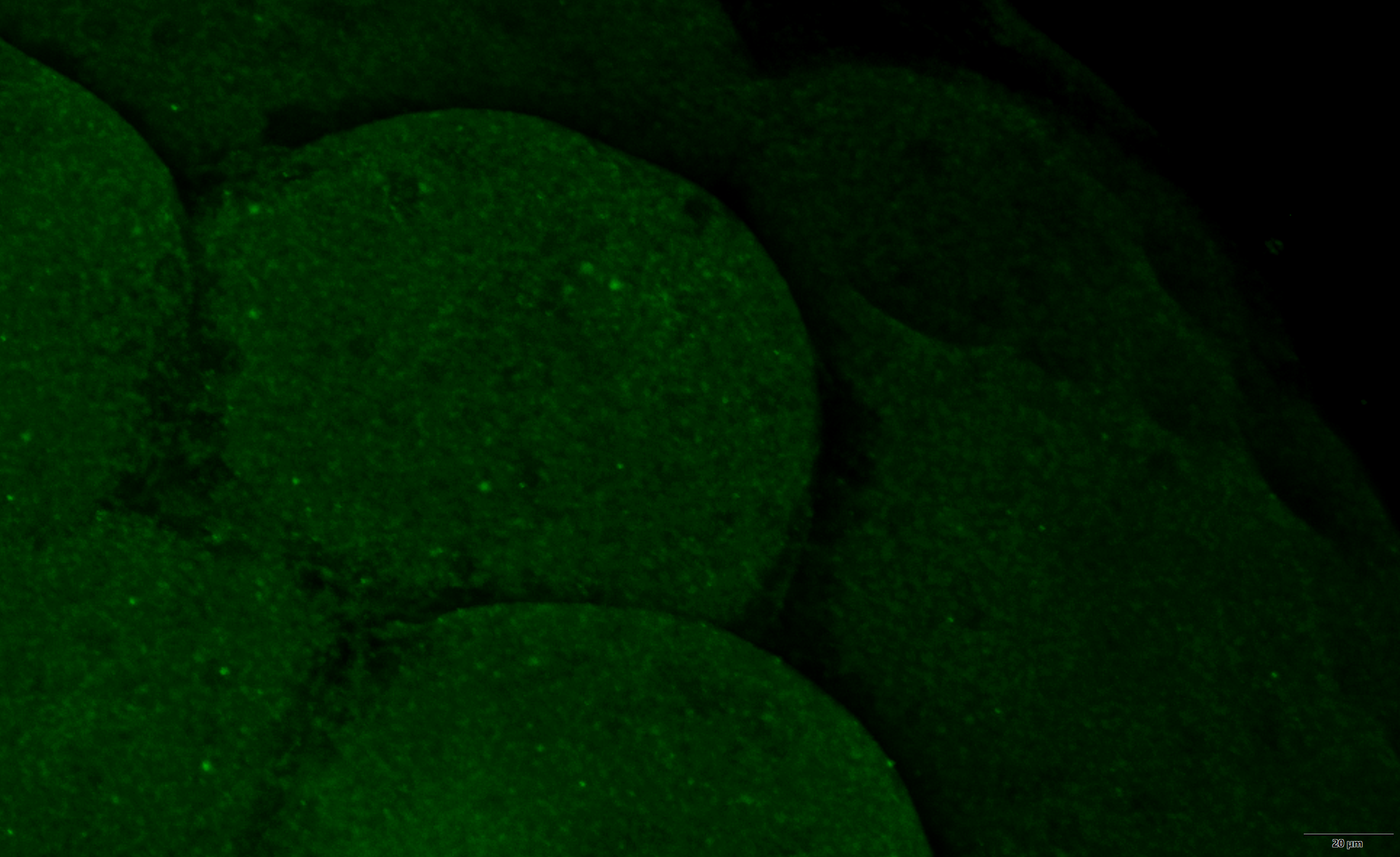

Supplement: Supplementary file 20 — Figure EV3 Source Data [file 44318_2025_442_MOESM20_ESM.zip › Figure_EV3/Figure EV3a/Mrbm24a tdrd6-myc.tif]

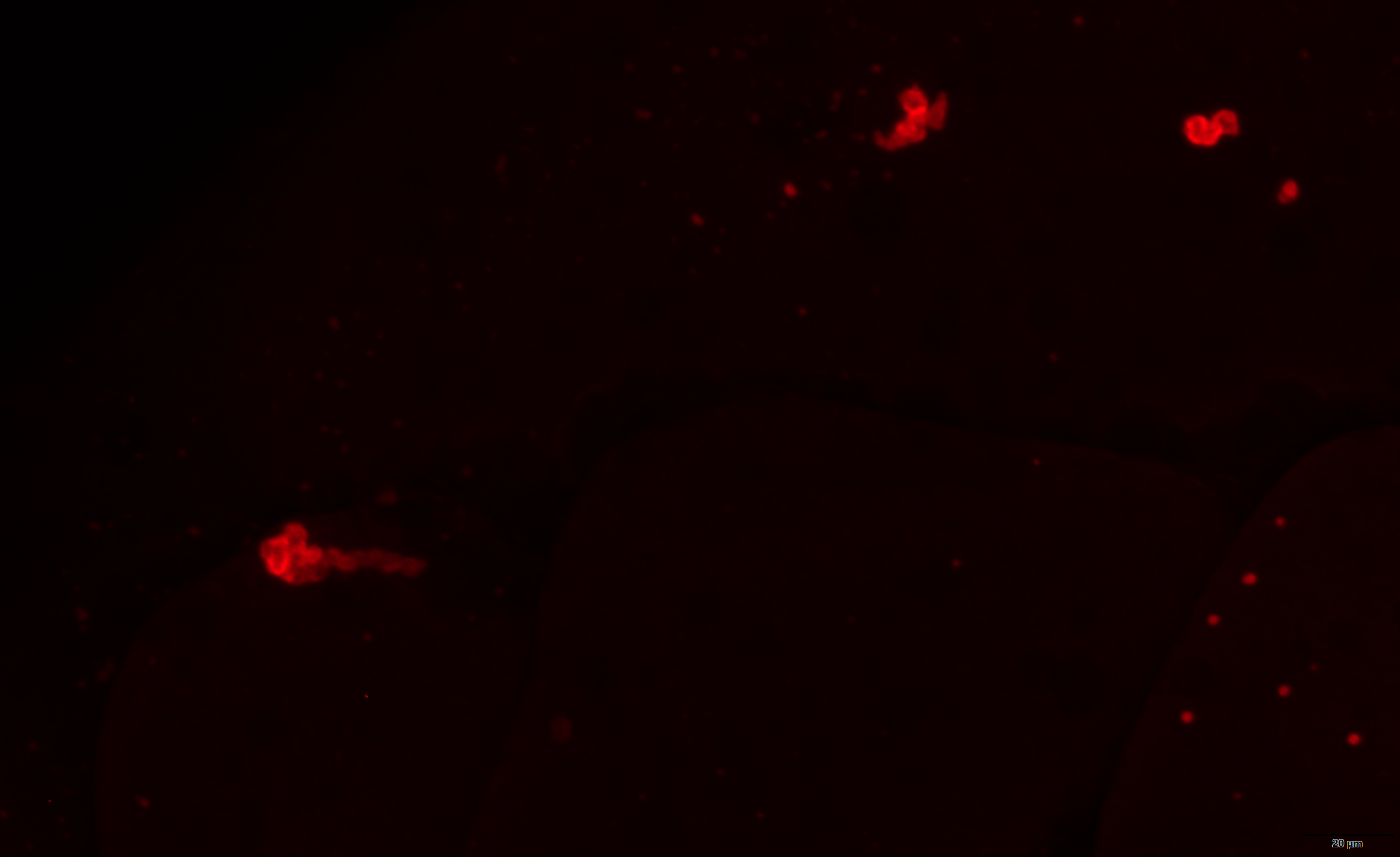

Supplement: Supplementary file 20 — Figure EV3 Source Data [file 44318_2025_442_MOESM20_ESM.zip › Figure_EV3/Figure EV3a/sibling tdrd6 piwil.tif]

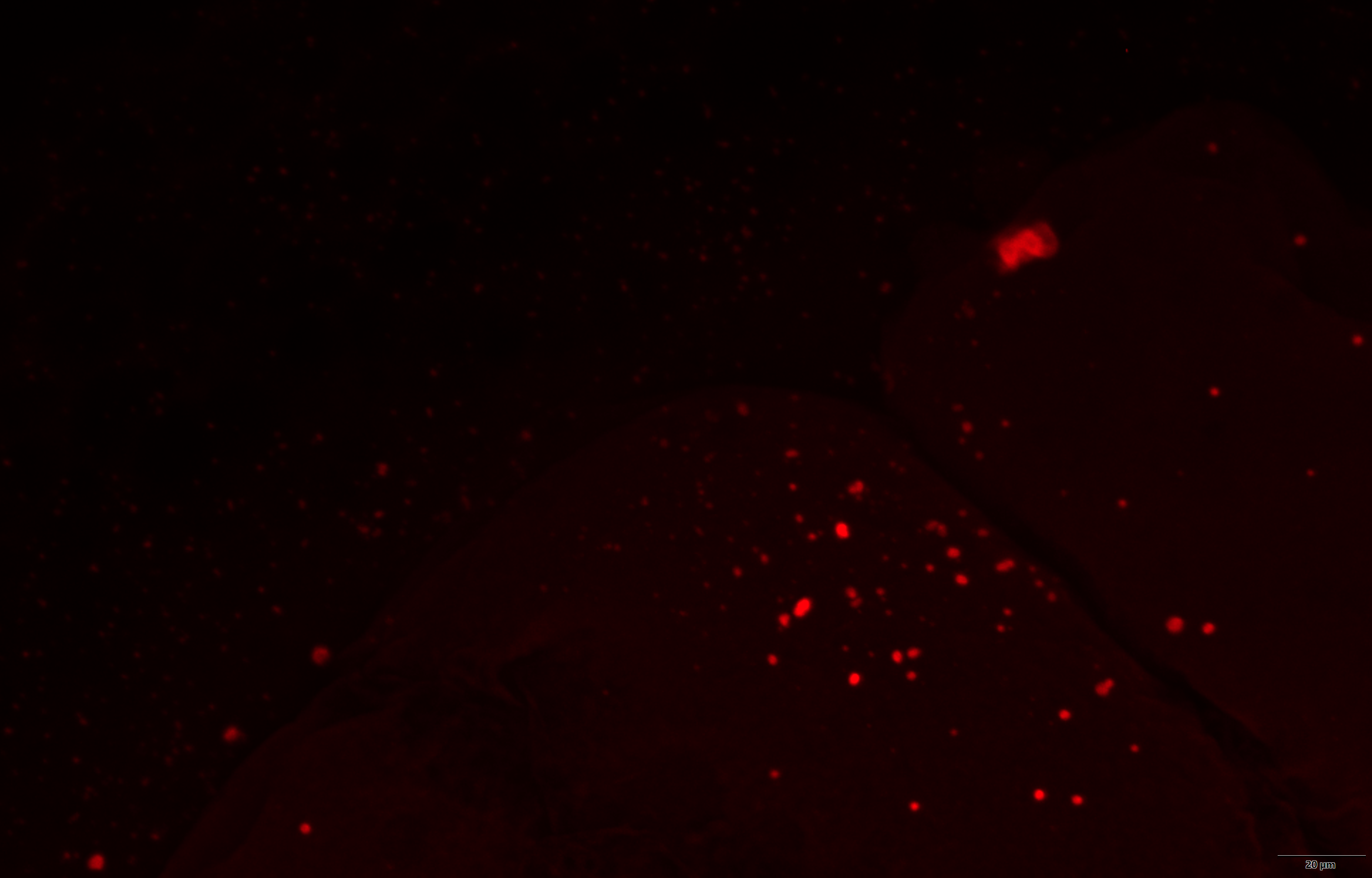

Supplement: Supplementary file 20 — Figure EV3 Source Data [file 44318_2025_442_MOESM20_ESM.zip › Figure_EV3/Figure EV3a/sibling celf1 piwil .tif]

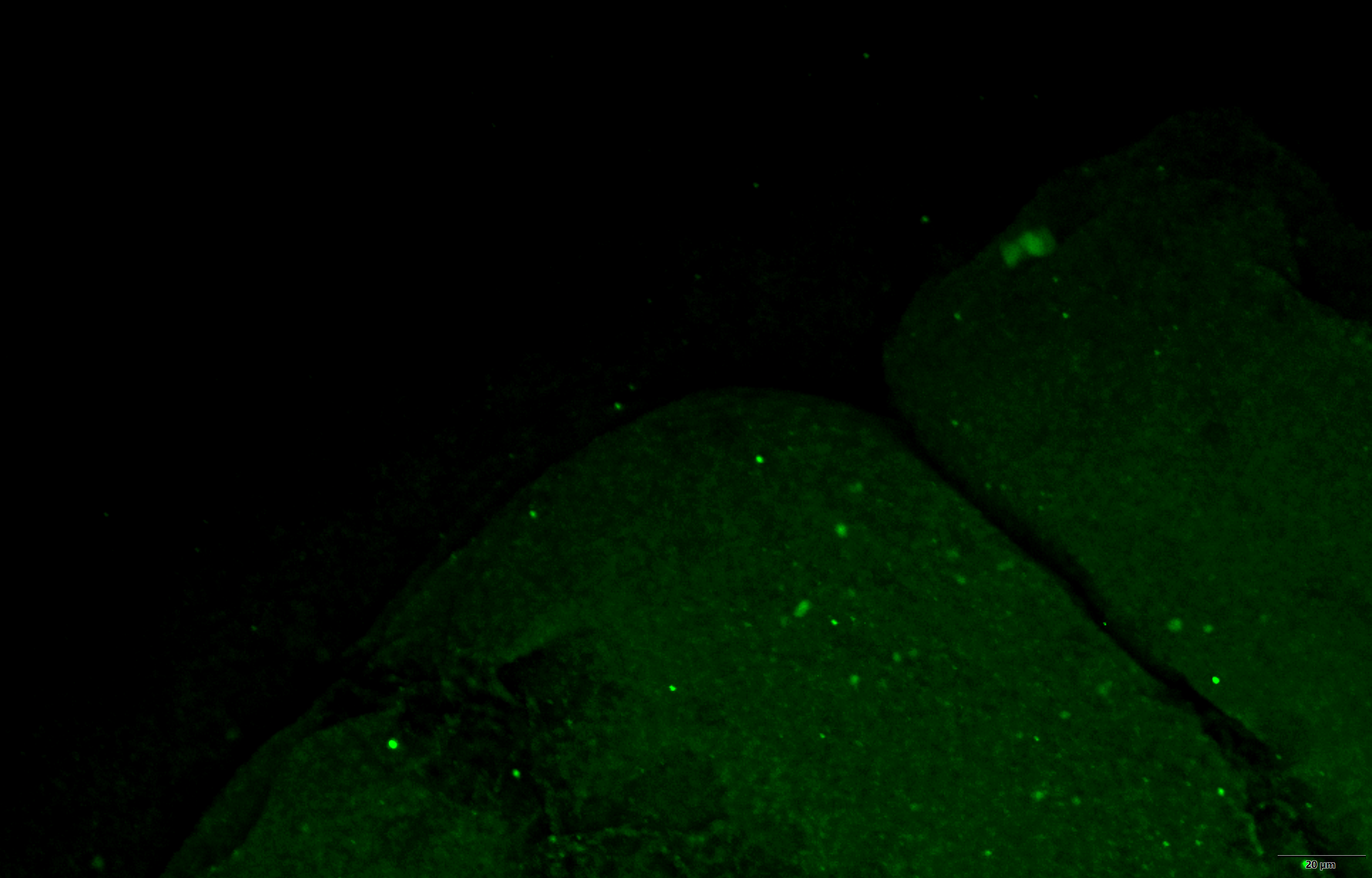

Supplement: Supplementary file 20 — Figure EV3 Source Data [file 44318_2025_442_MOESM20_ESM.zip › Figure_EV3/Figure EV3a/sibling celf1-myc.tif]

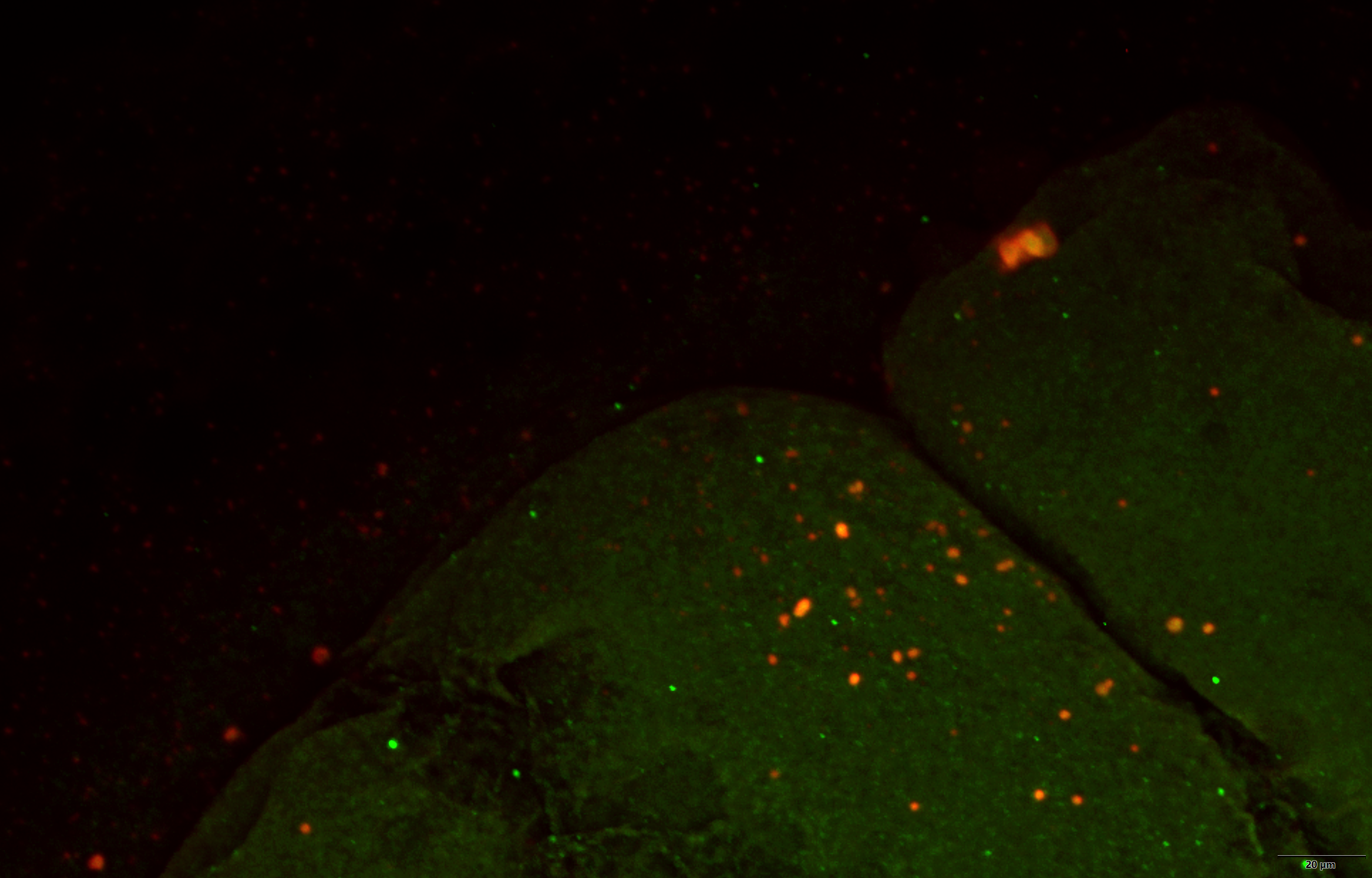

Supplement: Supplementary file 20 — Figure EV3 Source Data [file 44318_2025_442_MOESM20_ESM.zip › Figure_EV3/Figure EV3a/sibling piwil and celf1.tif]

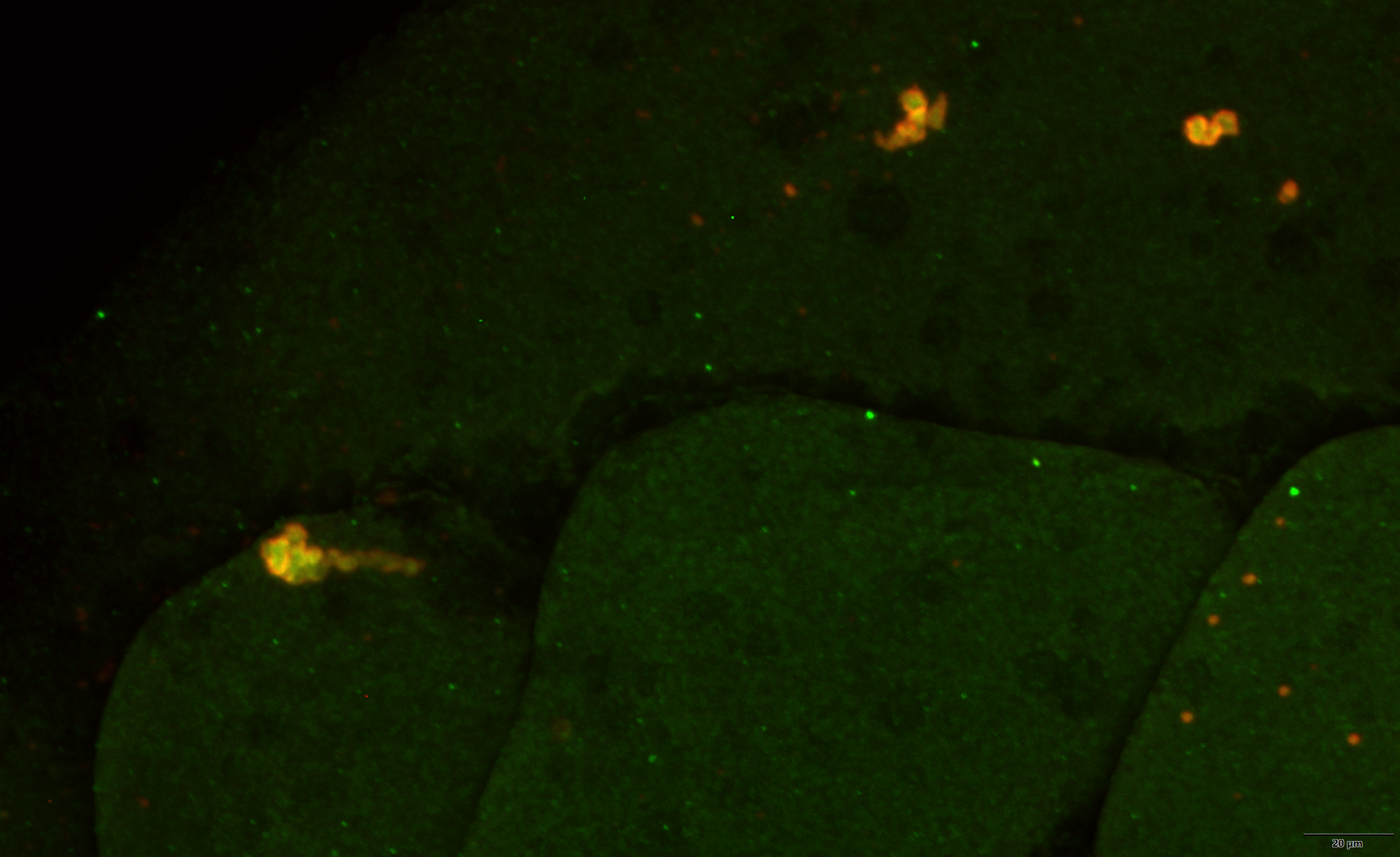

Supplement: Supplementary file 20 — Figure EV3 Source Data [file 44318_2025_442_MOESM20_ESM.zip › Figure_EV3/Figure EV3a/sibling tdrd6-myc and piwil.tif]

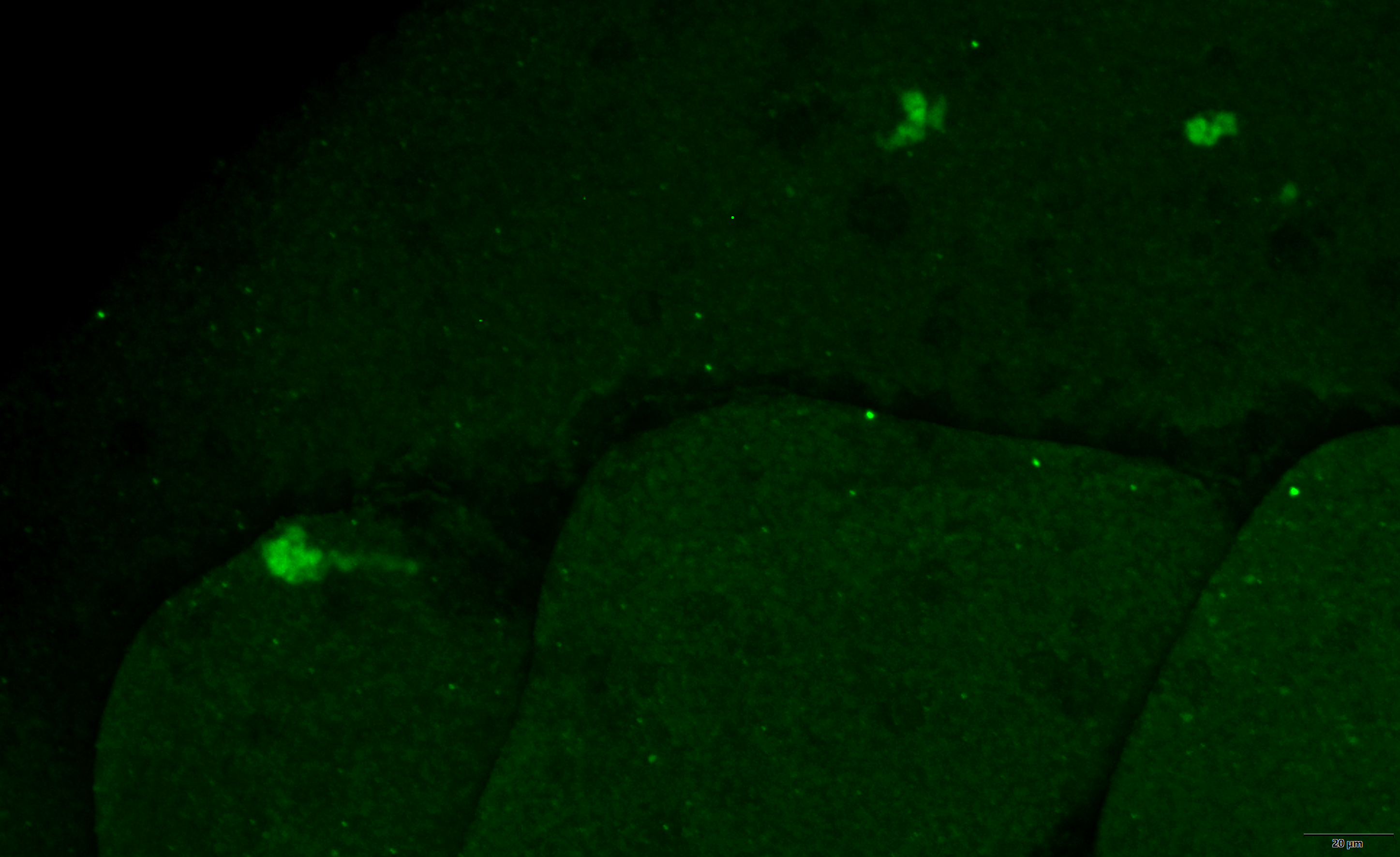

Supplement: Supplementary file 20 — Figure EV3 Source Data [file 44318_2025_442_MOESM20_ESM.zip › Figure_EV3/Figure EV3a/sibling tdrd6-myc.tif]

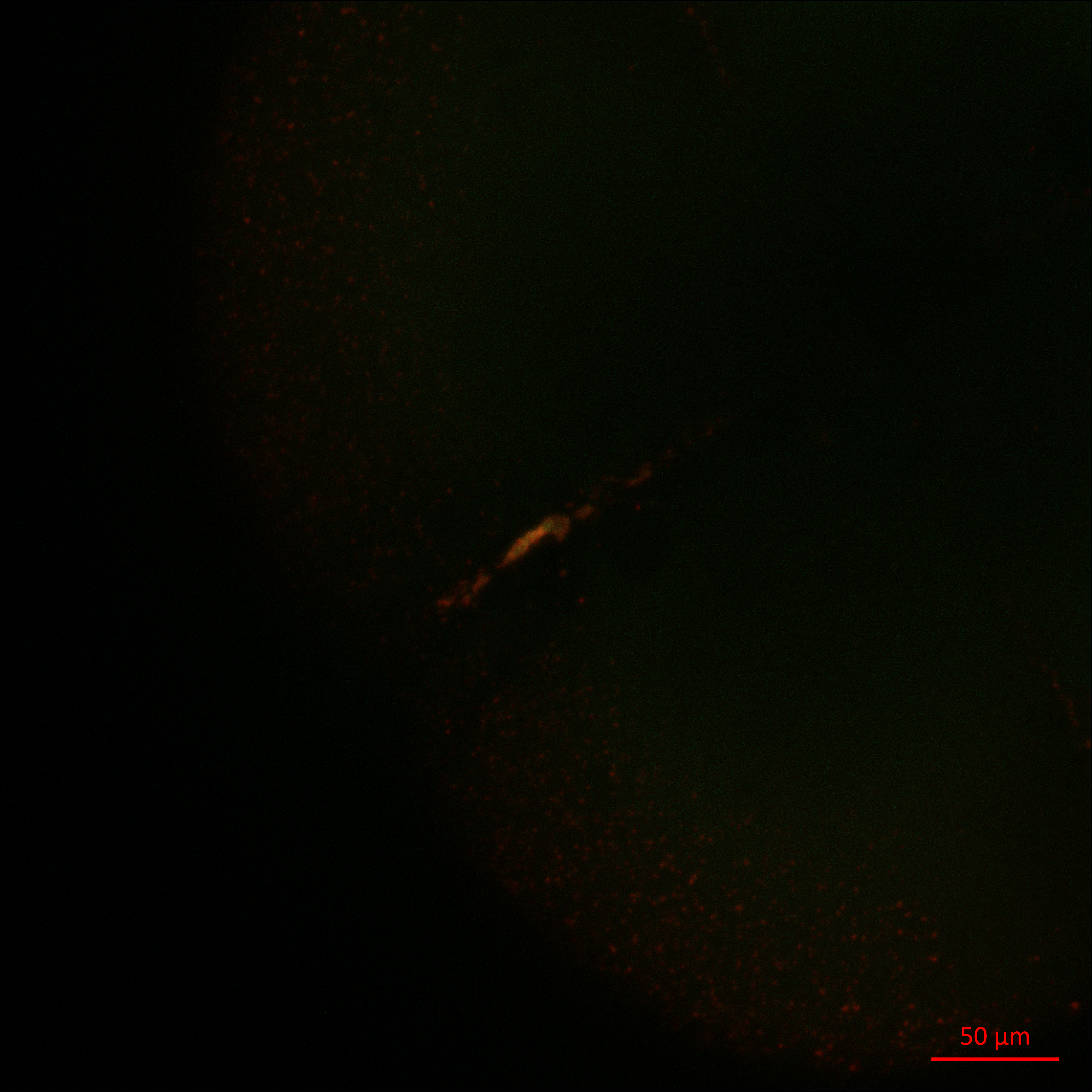

Supplement: Supplementary file 20 — Figure EV3 Source Data [file 44318_2025_442_MOESM20_ESM.zip › Figure_EV3/Figure EV3b/merge KI gfp myosin2 4-cell.tif]

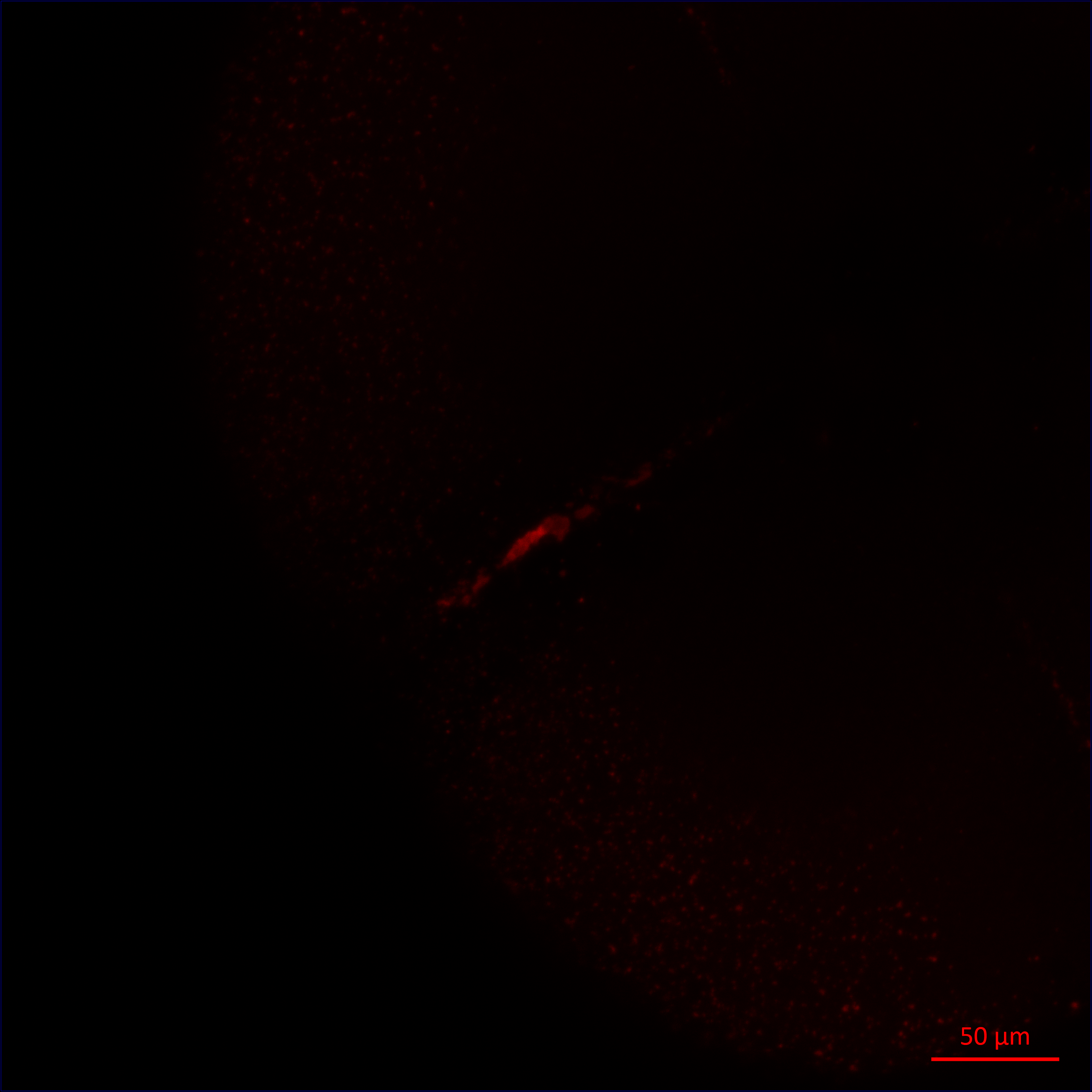

Supplement: Supplementary file 20 — Figure EV3 Source Data [file 44318_2025_442_MOESM20_ESM.zip › Figure_EV3/Figure EV3b/myosin2 4-cell .tif]

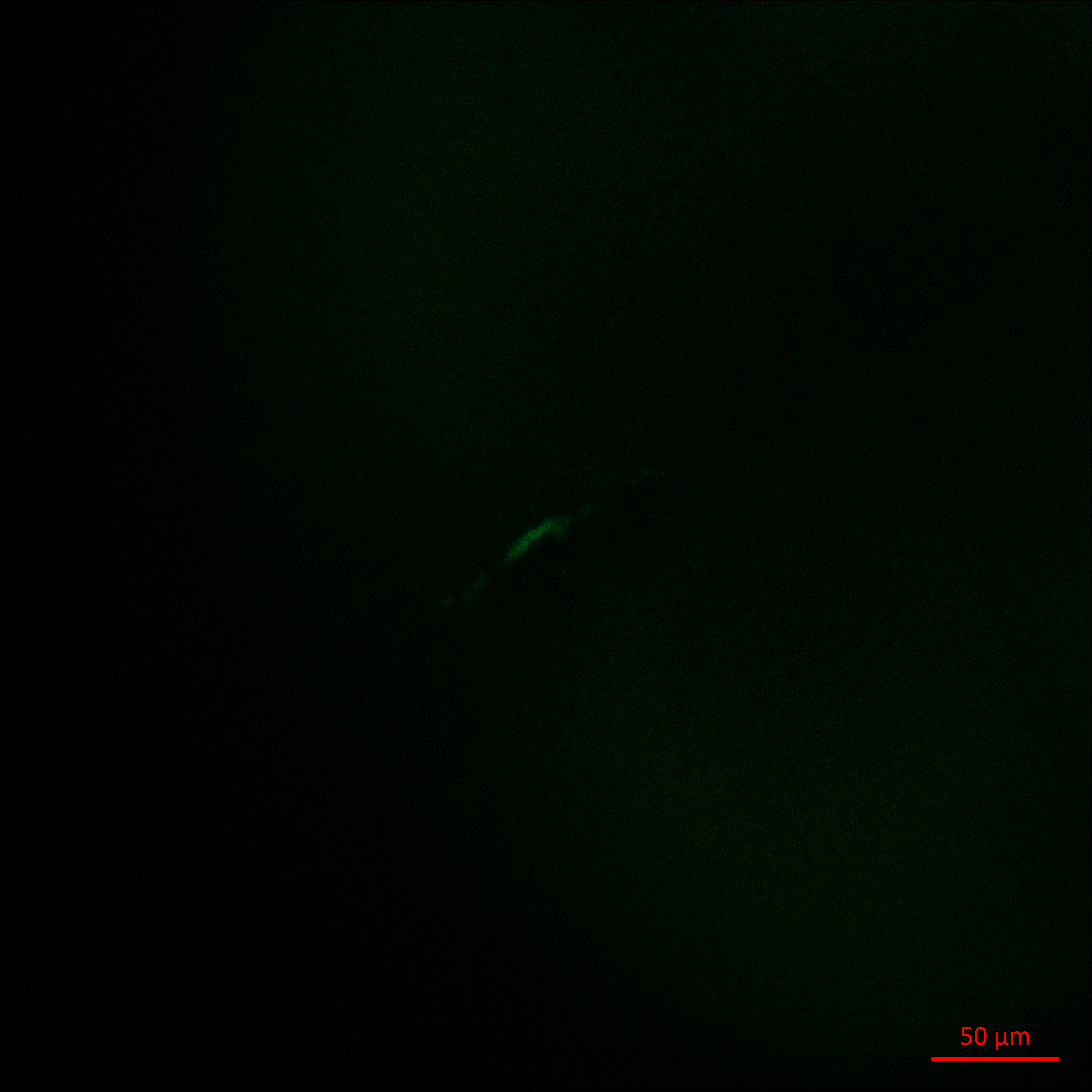

Supplement: Supplementary file 20 — Figure EV3 Source Data [file 44318_2025_442_MOESM20_ESM.zip › Figure_EV3/Figure EV3b/rbm24a KI gfp 4-cell.tif]

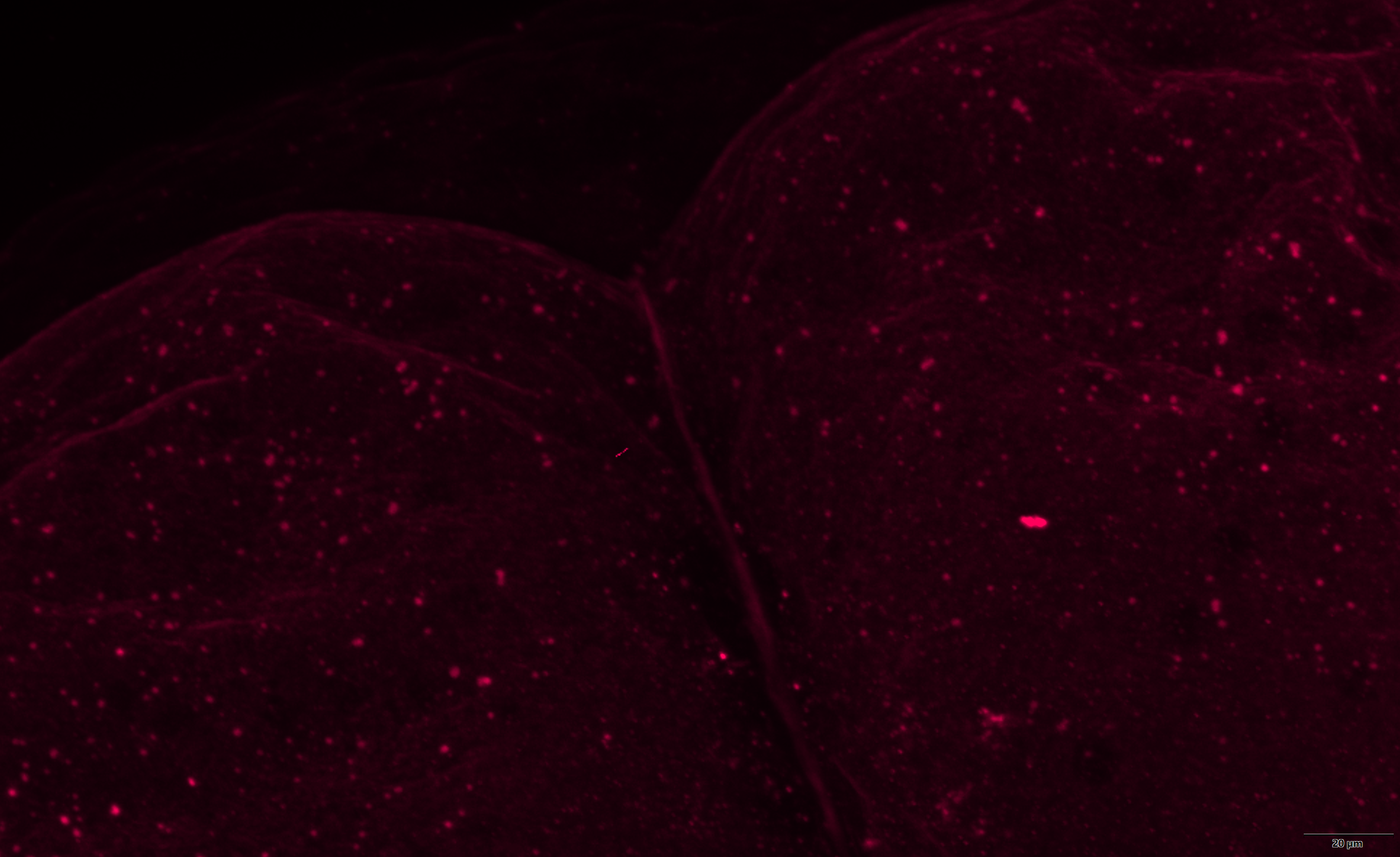

Supplement: Supplementary file 20 — Figure EV3 Source Data [file 44318_2025_442_MOESM20_ESM.zip › Figure_EV3/Figure EV3c/Mrbm24 p-myosin 2 60x.tif]

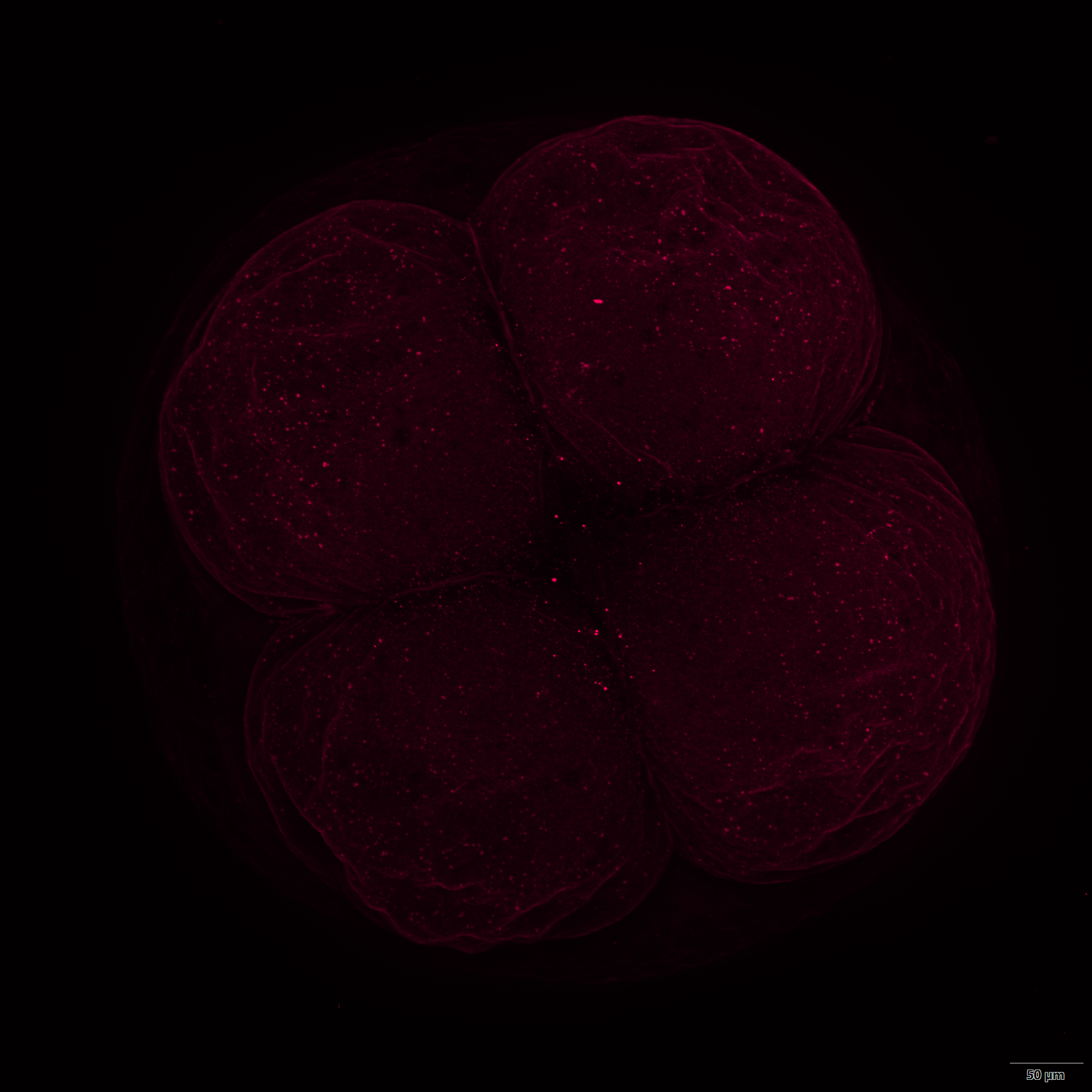

Supplement: Supplementary file 20 — Figure EV3 Source Data [file 44318_2025_442_MOESM20_ESM.zip › Figure_EV3/Figure EV3c/Mrbm24 p-myosin2.tif]

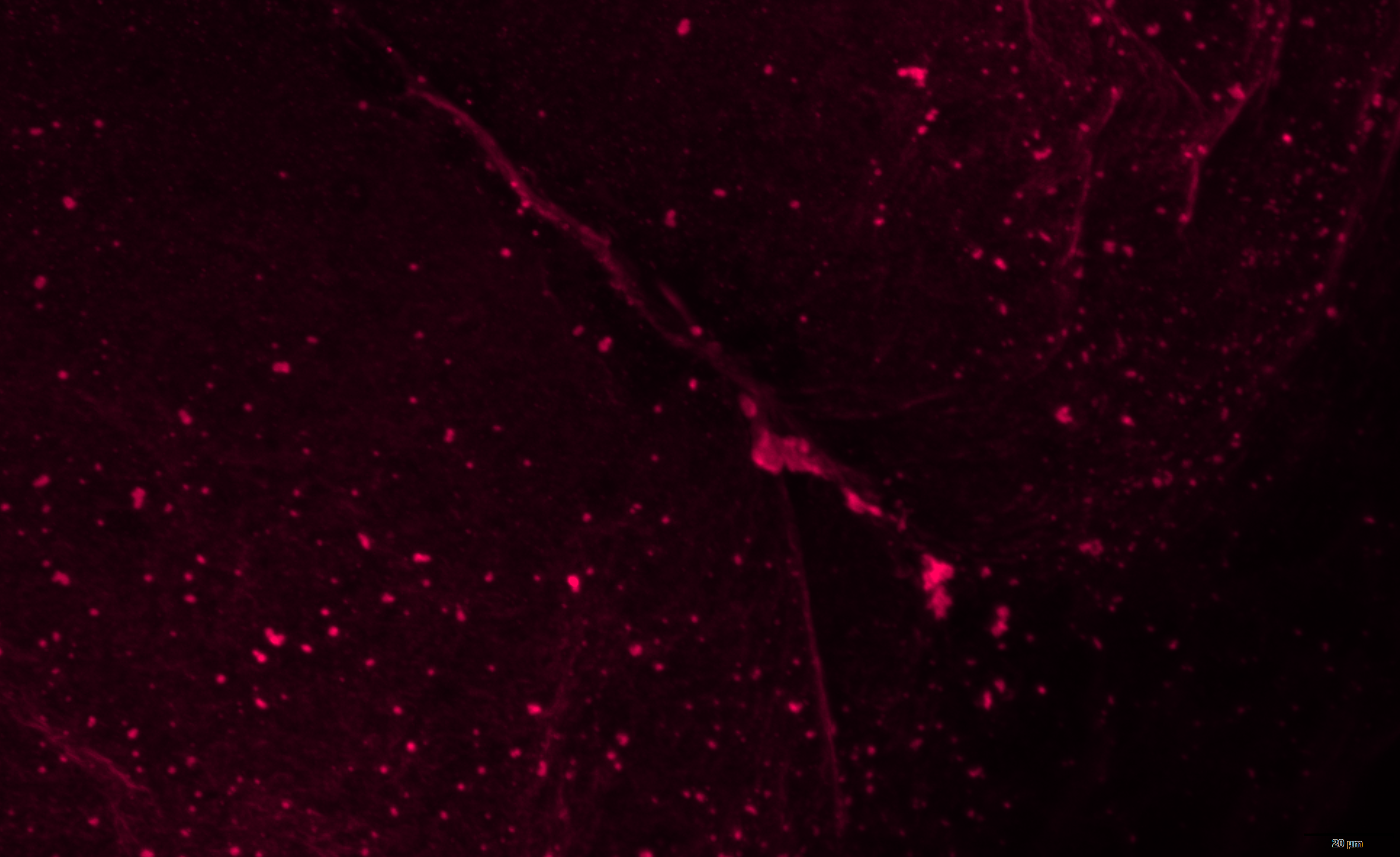

Supplement: Supplementary file 20 — Figure EV3 Source Data [file 44318_2025_442_MOESM20_ESM.zip › Figure_EV3/Figure EV3c/wt p-myosin 2 60x.tif]

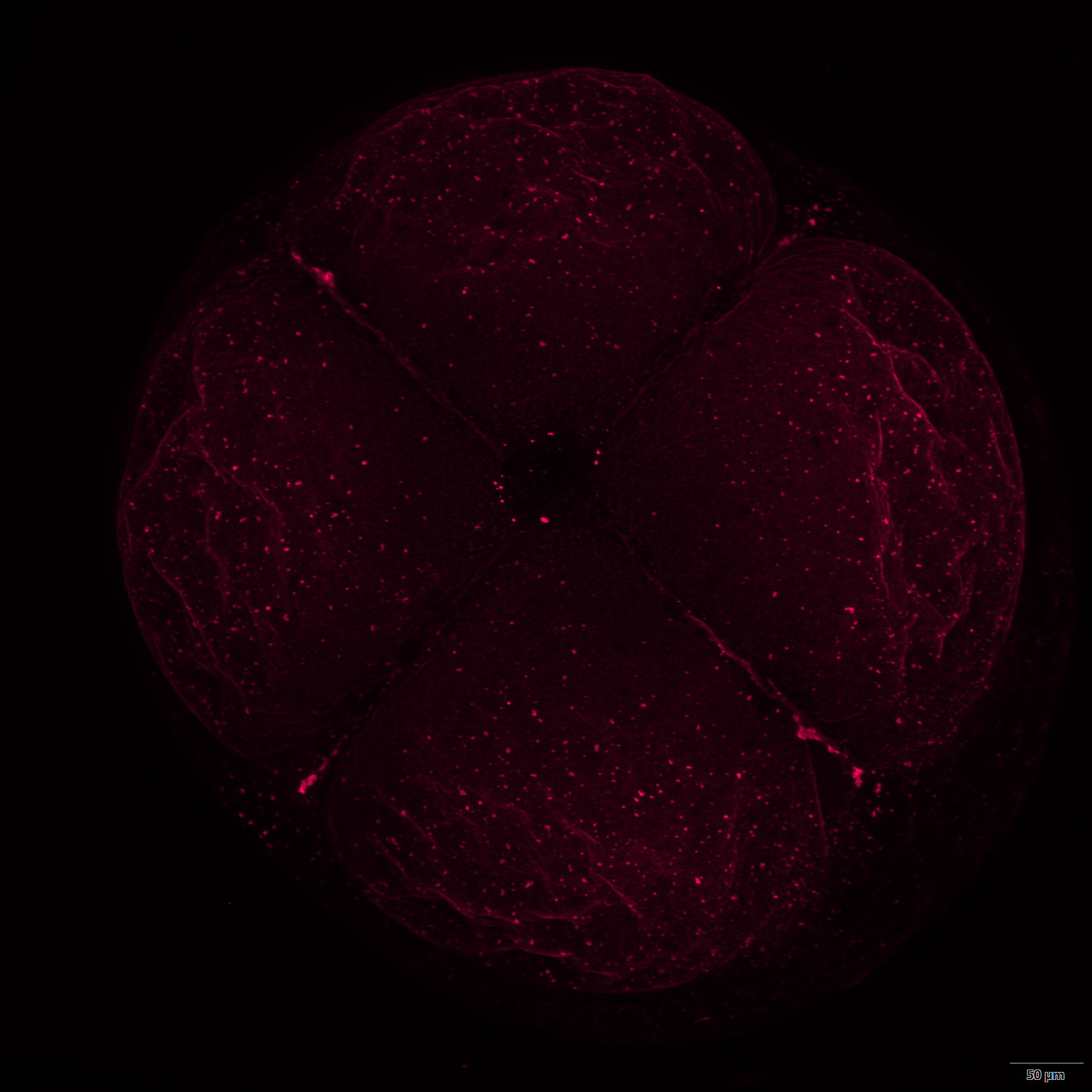

Supplement: Supplementary file 20 — Figure EV3 Source Data [file 44318_2025_442_MOESM20_ESM.zip › Figure_EV3/Figure EV3c/wt p-myosin 2.tif]

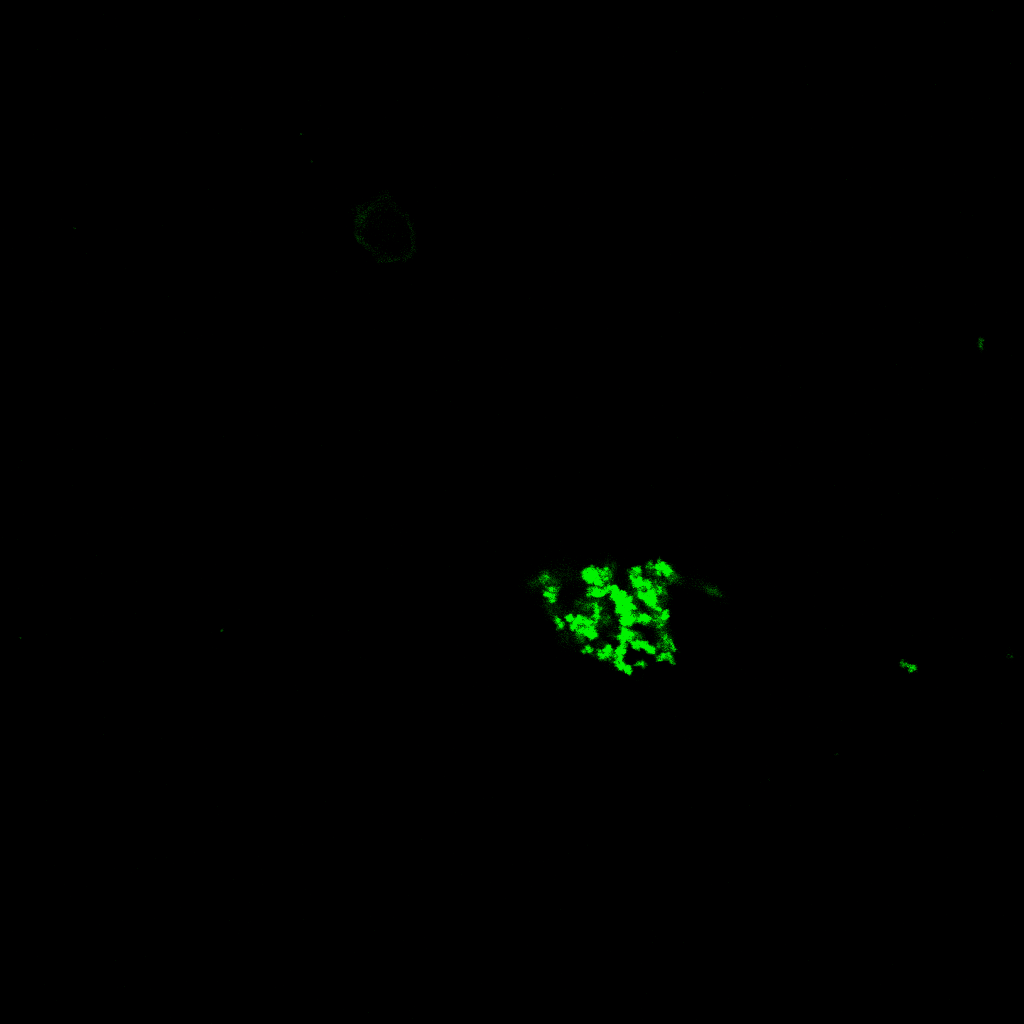

Supplement: Supplementary file 20 — Figure EV3 Source Data [file 44318_2025_442_MOESM20_ESM.zip › Figure_EV3/Figure EV3d/pellet.tif]

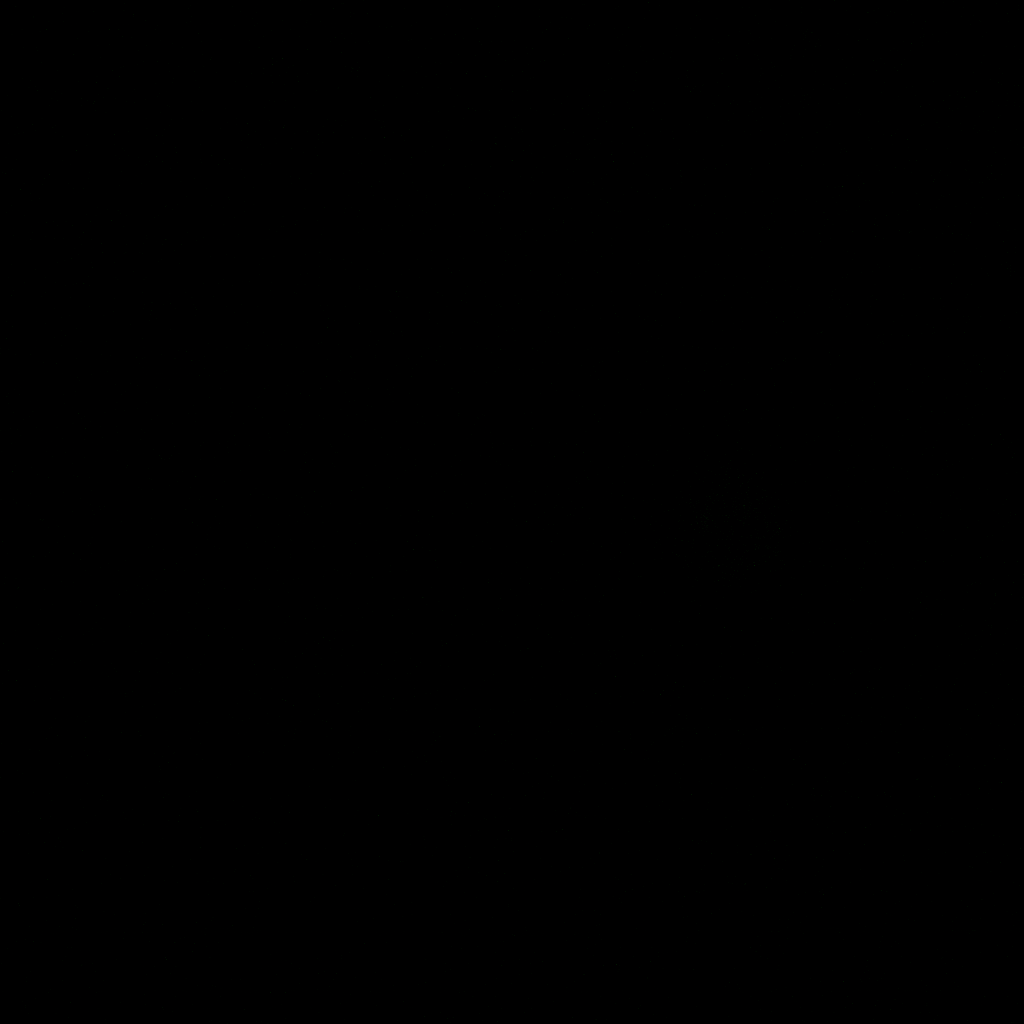

Supplement: Supplementary file 20 — Figure EV3 Source Data [file 44318_2025_442_MOESM20_ESM.zip › Figure_EV3/Figure EV3d/supernatant.tif]

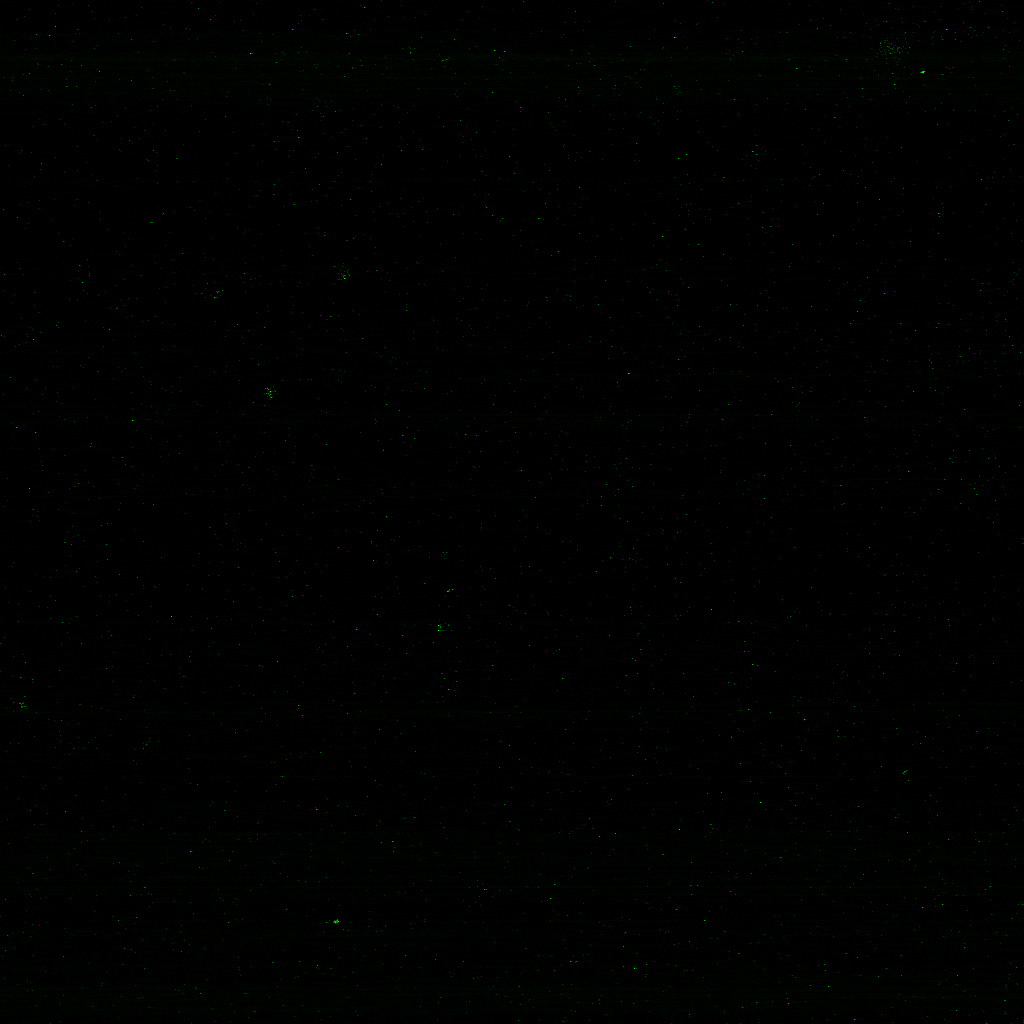

Supplement: Supplementary file 20 — Figure EV3 Source Data [file 44318_2025_442_MOESM20_ESM.zip › Figure_EV3/Figure EV3d/WT pellet.tif]

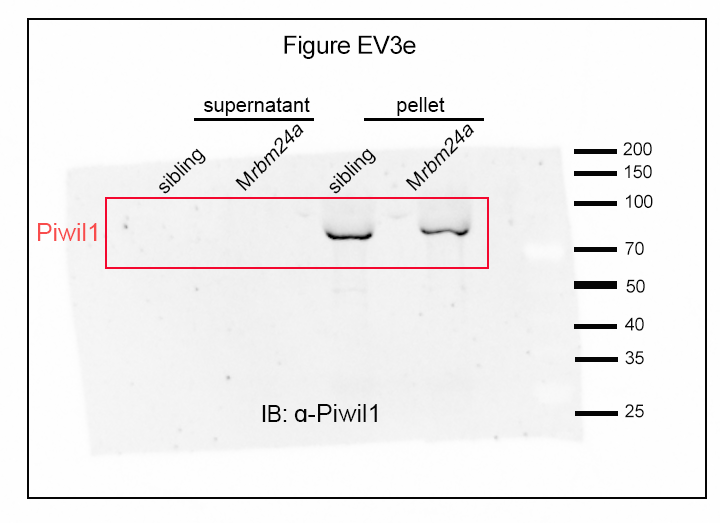

Supplement: Supplementary file 20 — Figure EV3 Source Data [file 44318_2025_442_MOESM20_ESM.zip › Figure_EV3/Figure EV3e/Figure EV3e.tif]

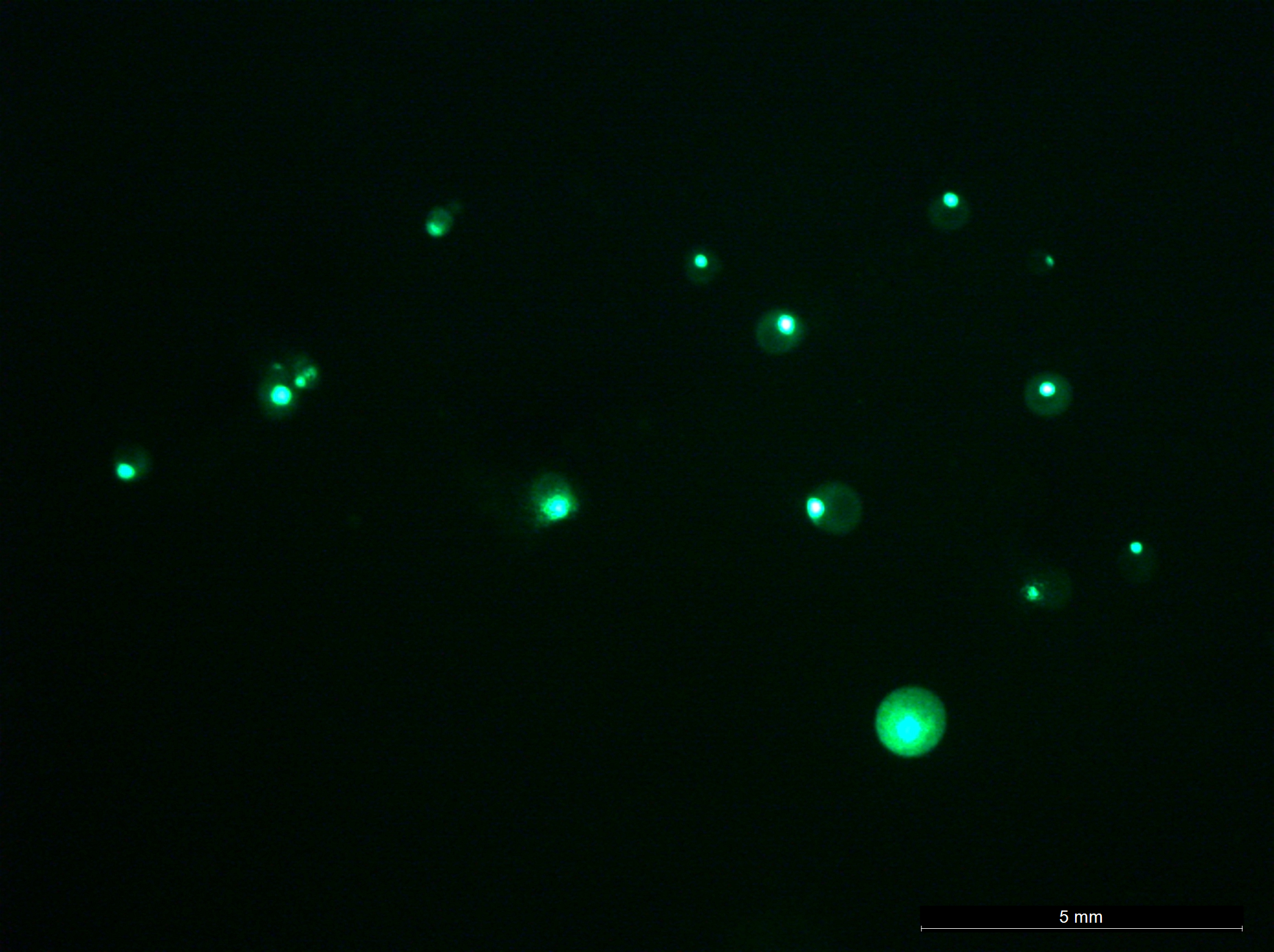

Supplement: Supplementary file 21 — Figure EV4 Source Data [file 44318_2025_442_MOESM21_ESM.zip › Figure_EV4/Figure EV4a/Rbm24a-GFP.tif]

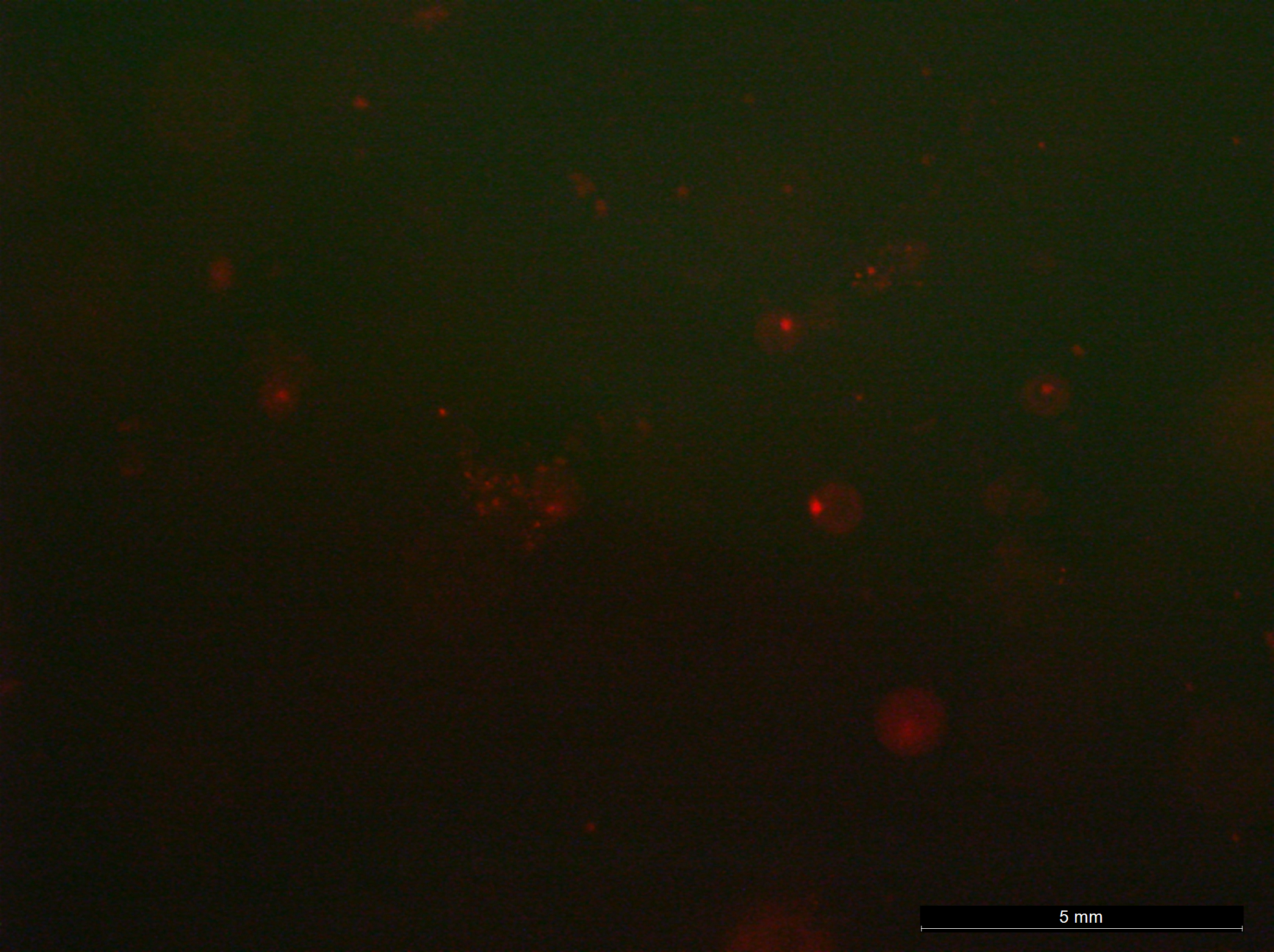

Supplement: Supplementary file 21 — Figure EV4 Source Data [file 44318_2025_442_MOESM21_ESM.zip › Figure_EV4/Figure EV4b/Rbm24a-RFP.tif]
