# Supplementary figures and images for: Rbm24a dictates mRNA recruitment for germ granule assembly in zebrafish (part 3 of 3)
Source: EMBO J. 2025 Apr 25;44(11):3121–49. doi: 10.1038/s44318-025-00442-z (PMC12130248; doi:10.1038/s44318-025-00442-z)

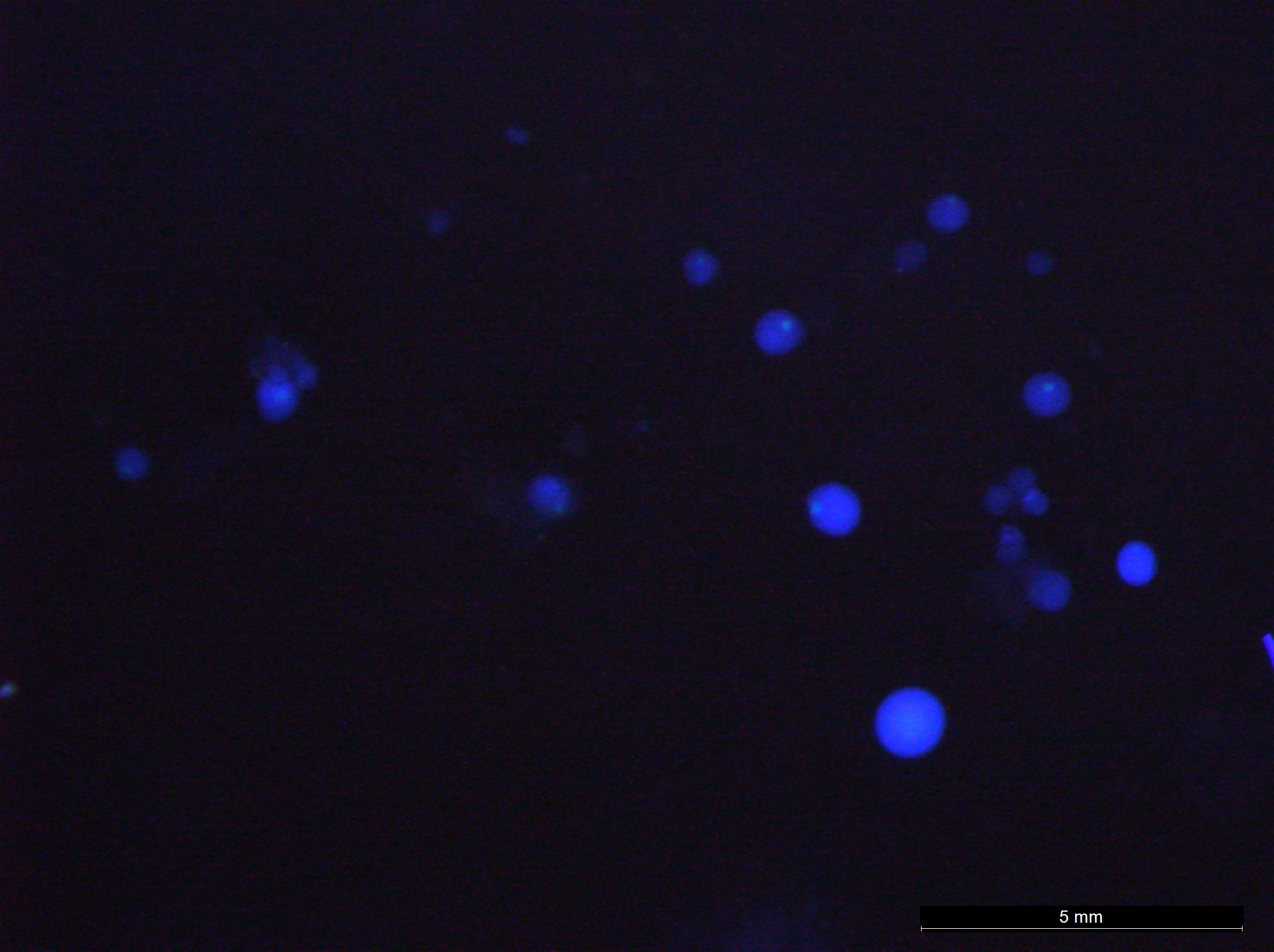

Supplement: Supplementary file 21 — Figure EV4 Source Data [file 44318_2025_442_MOESM21_ESM.zip › Figure_EV4/Figure EV4c/BFP.tif]

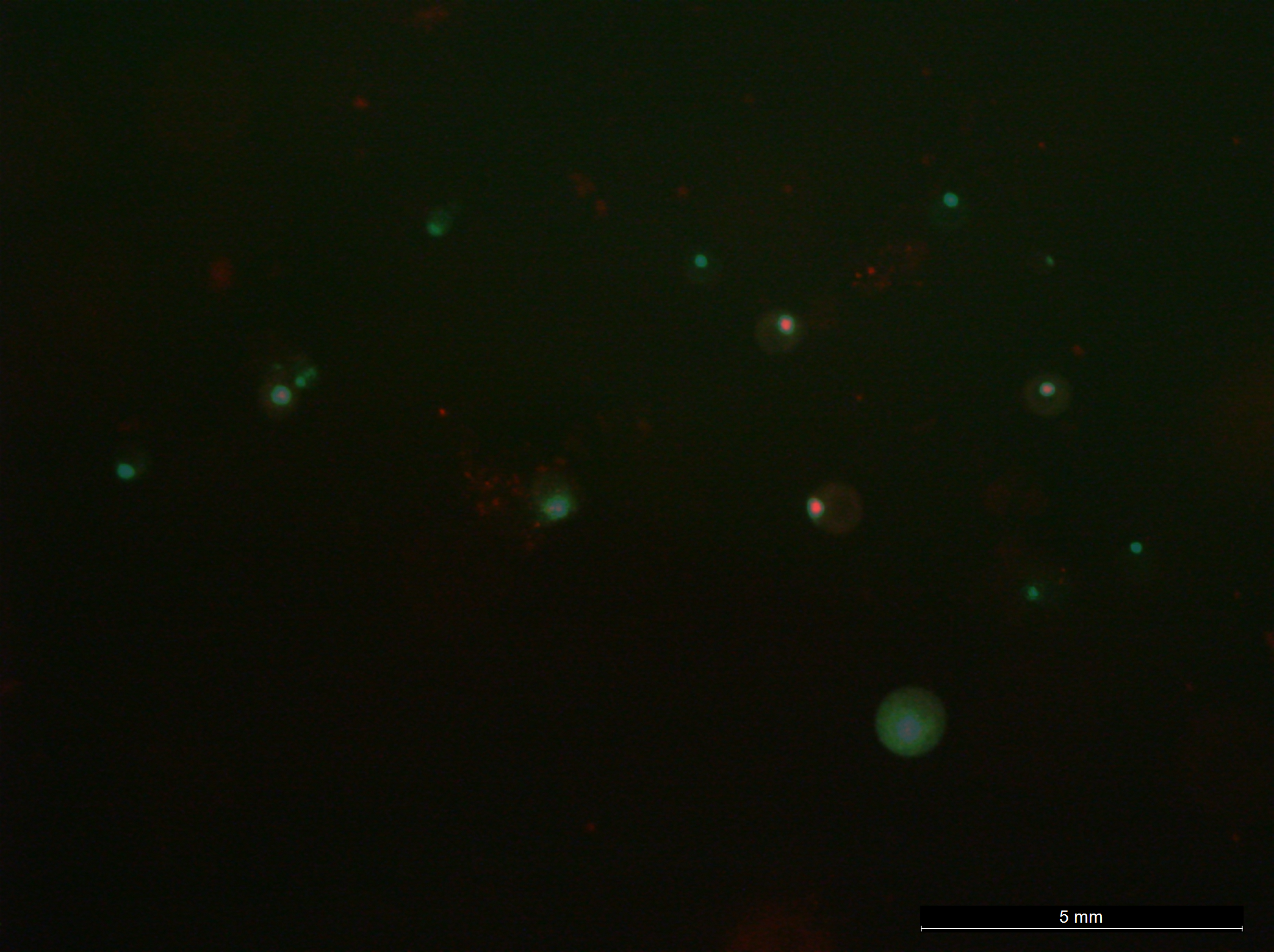

Supplement: Supplementary file 21 — Figure EV4 Source Data [file 44318_2025_442_MOESM21_ESM.zip › Figure_EV4/Figure EV4d/merge.tif]

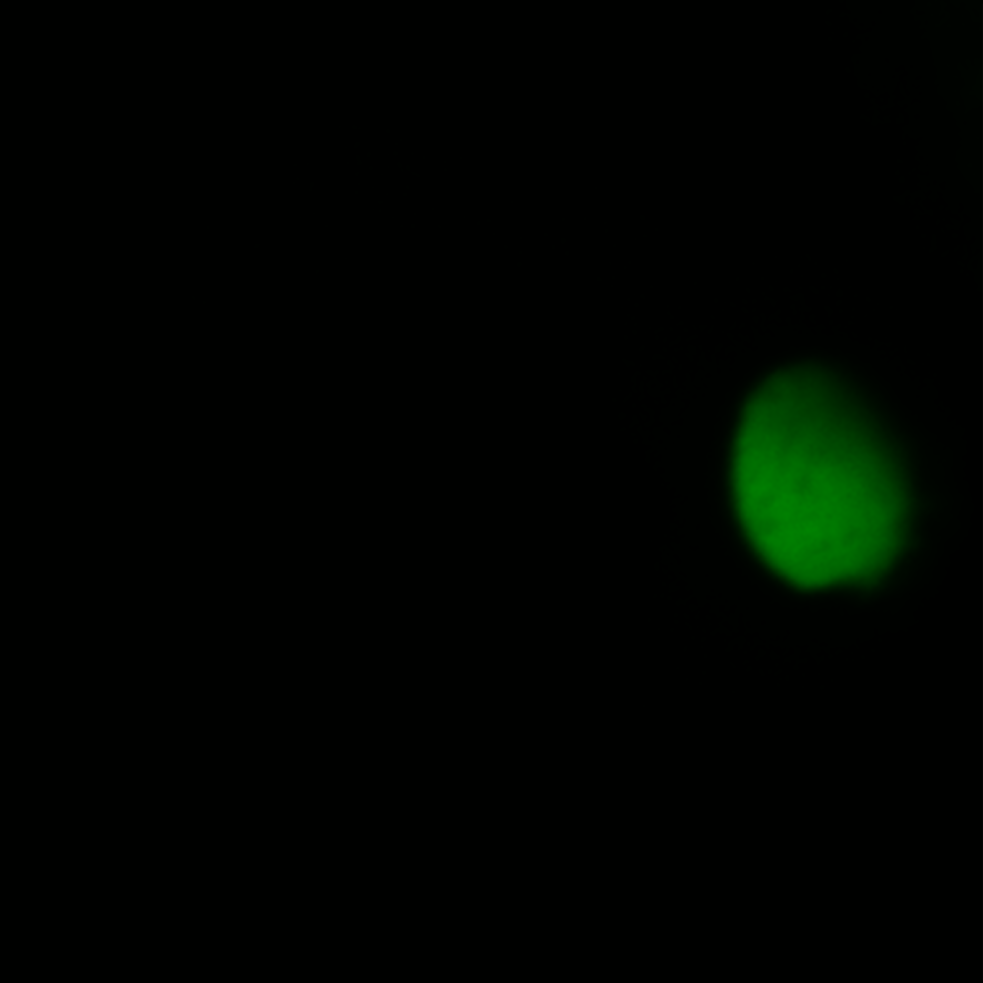

Supplement: Supplementary file 21 — Figure EV4 Source Data [file 44318_2025_442_MOESM21_ESM.zip › Figure_EV4/Figure EV4e/1 wt buc-gfp .tif]

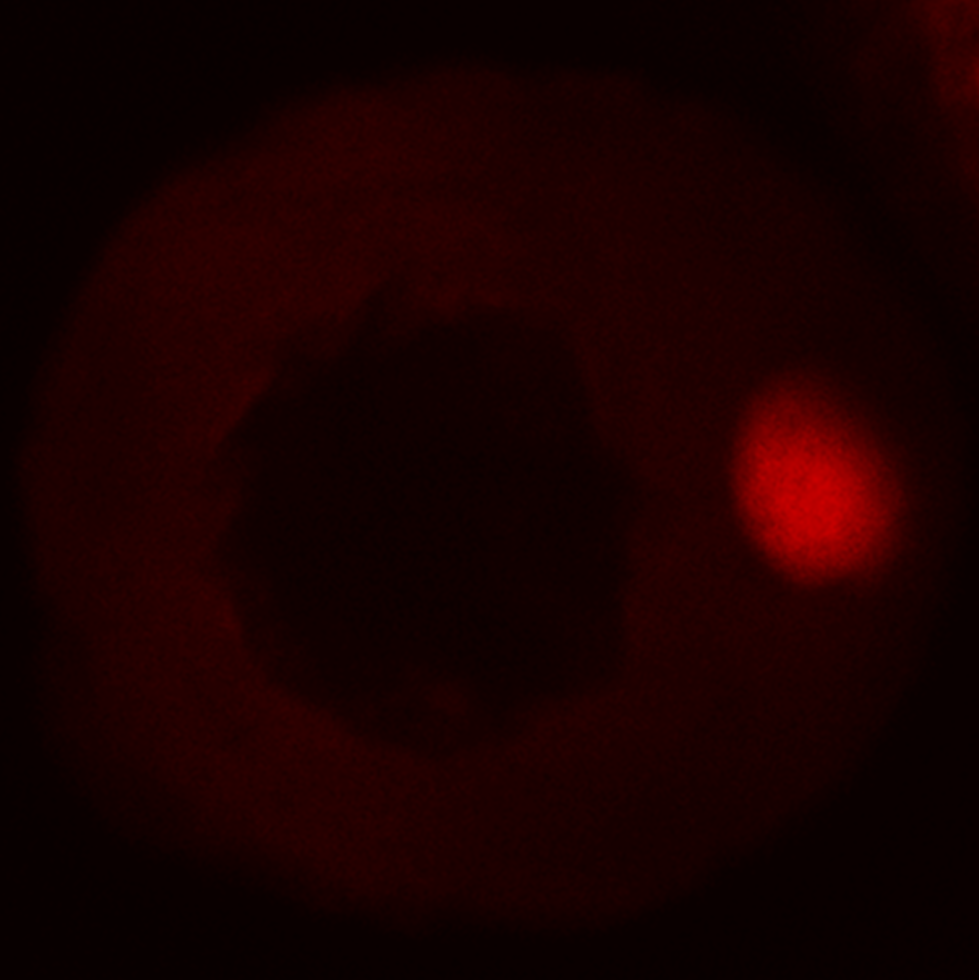

Supplement: Supplementary file 21 — Figure EV4 Source Data [file 44318_2025_442_MOESM21_ESM.zip › Figure_EV4/Figure EV4e/1 wt dazl .tif]

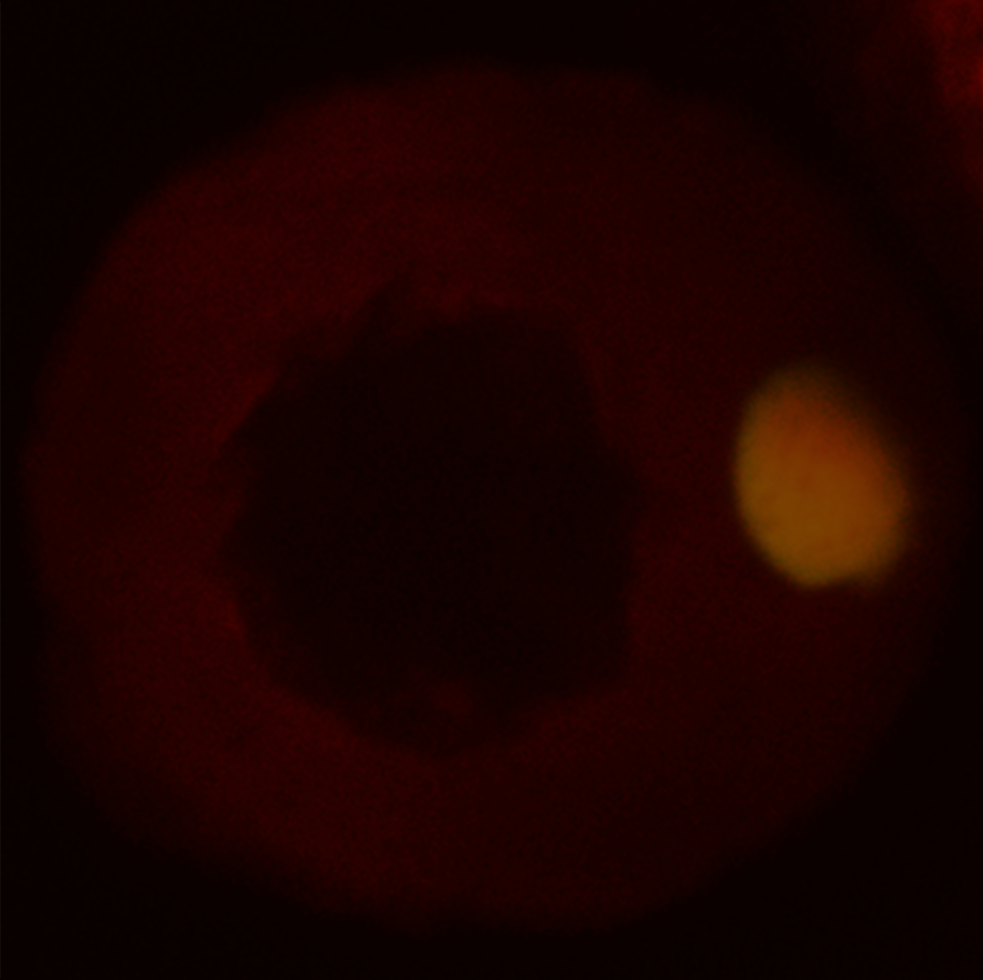

Supplement: Supplementary file 21 — Figure EV4 Source Data [file 44318_2025_442_MOESM21_ESM.zip › Figure_EV4/Figure EV4e/1 wt merge .tif]

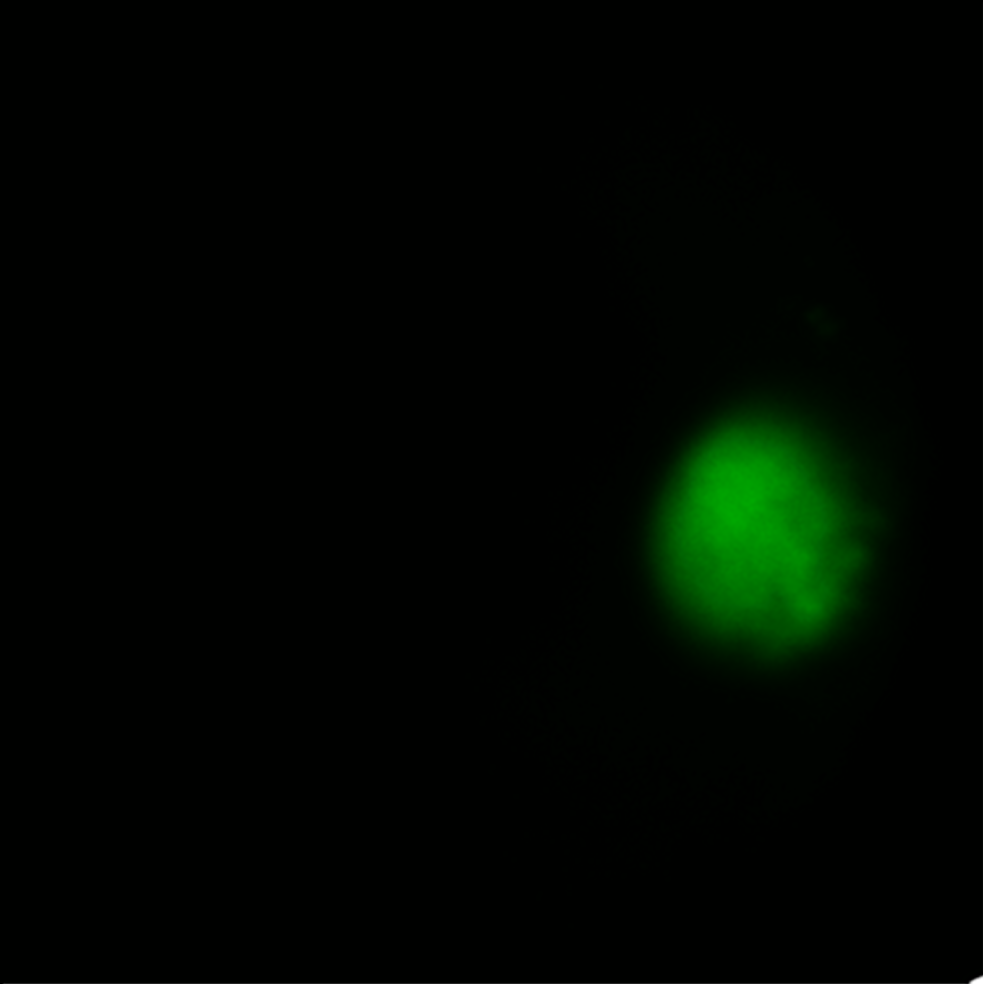

Supplement: Supplementary file 21 — Figure EV4 Source Data [file 44318_2025_442_MOESM21_ESM.zip › Figure_EV4/Figure EV4e/Mrbm24a 1 buc-gfp .tif]

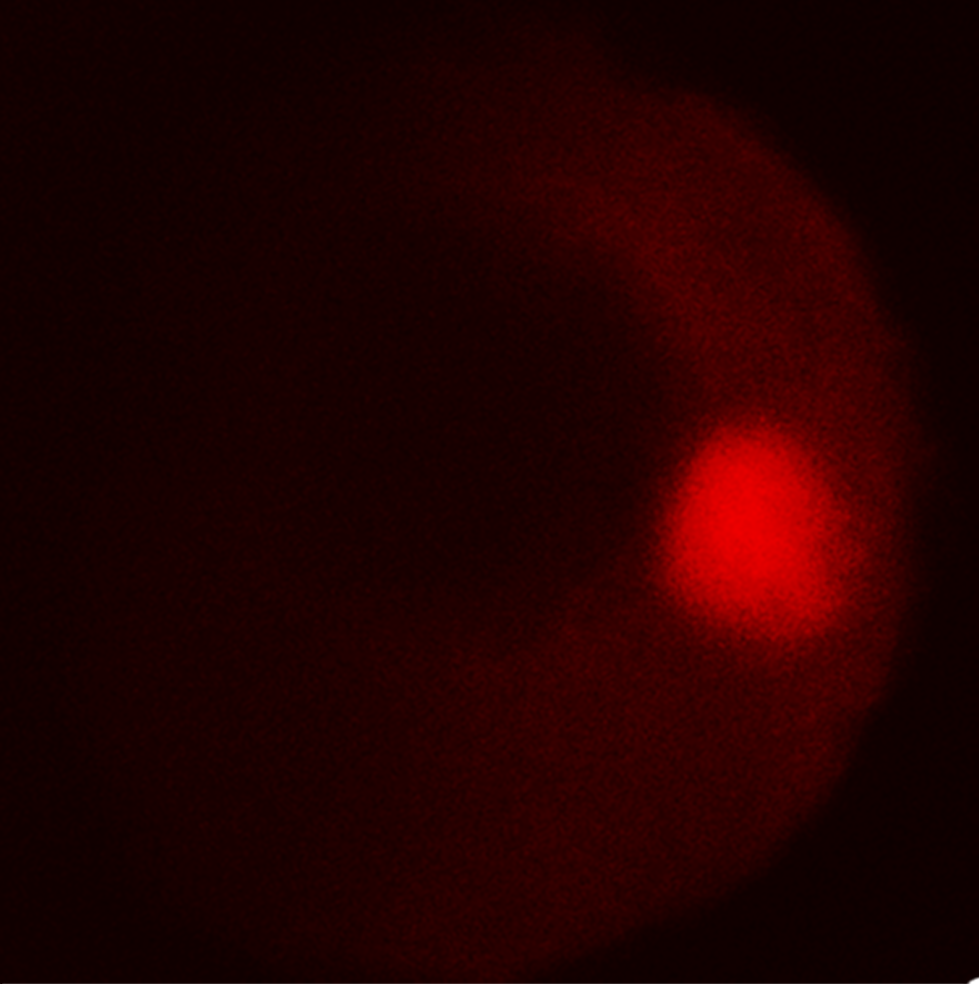

Supplement: Supplementary file 21 — Figure EV4 Source Data [file 44318_2025_442_MOESM21_ESM.zip › Figure_EV4/Figure EV4e/Mrbm24a 1 dazl .tif]

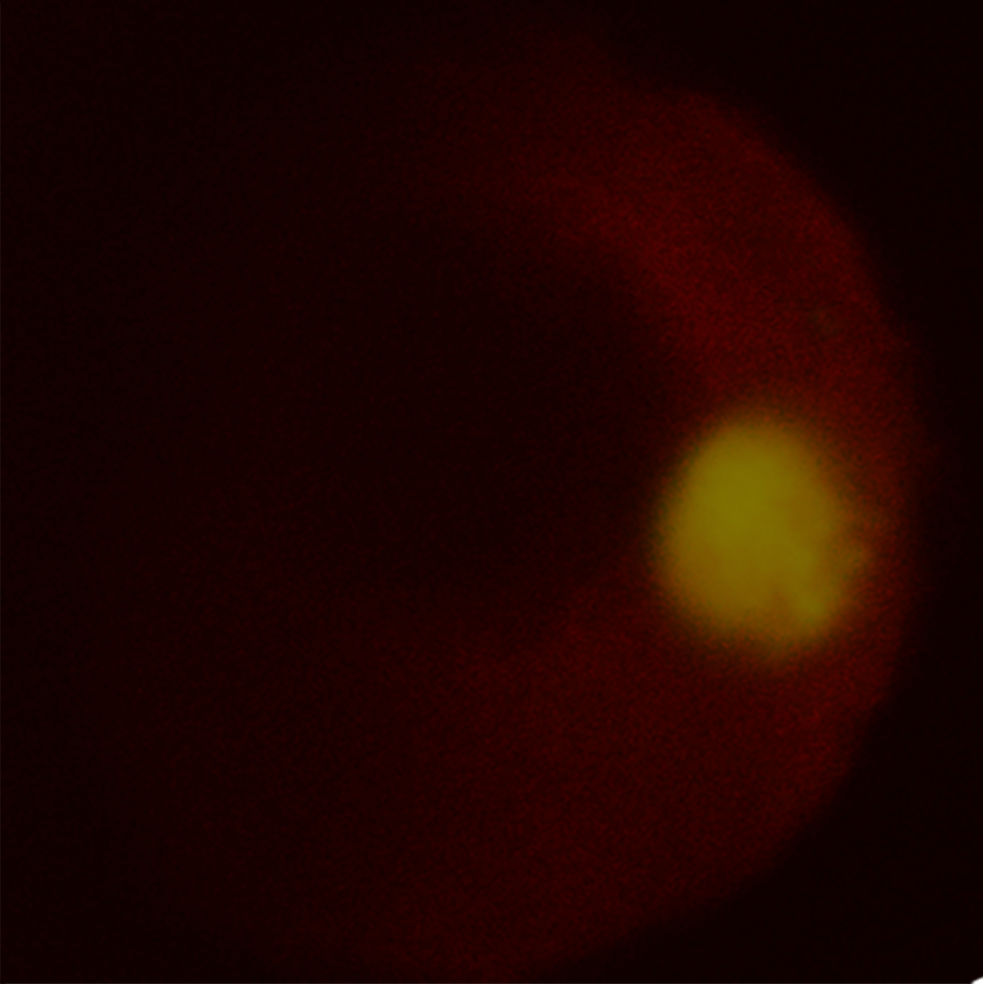

Supplement: Supplementary file 21 — Figure EV4 Source Data [file 44318_2025_442_MOESM21_ESM.zip › Figure_EV4/Figure EV4e/Mrbm24a 1 merge .tif]

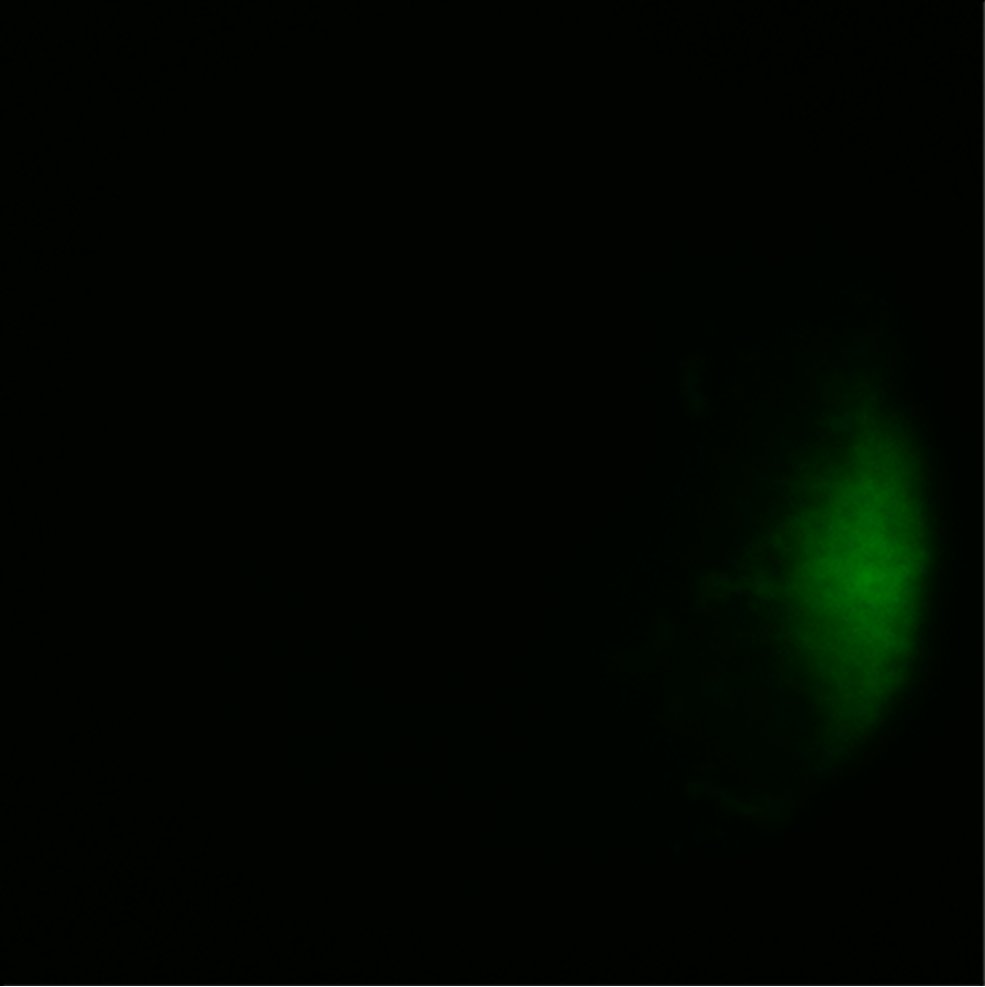

Supplement: Supplementary file 21 — Figure EV4 Source Data [file 44318_2025_442_MOESM21_ESM.zip › Figure_EV4/Figure EV4f/2 wt buc-gfp .tif]

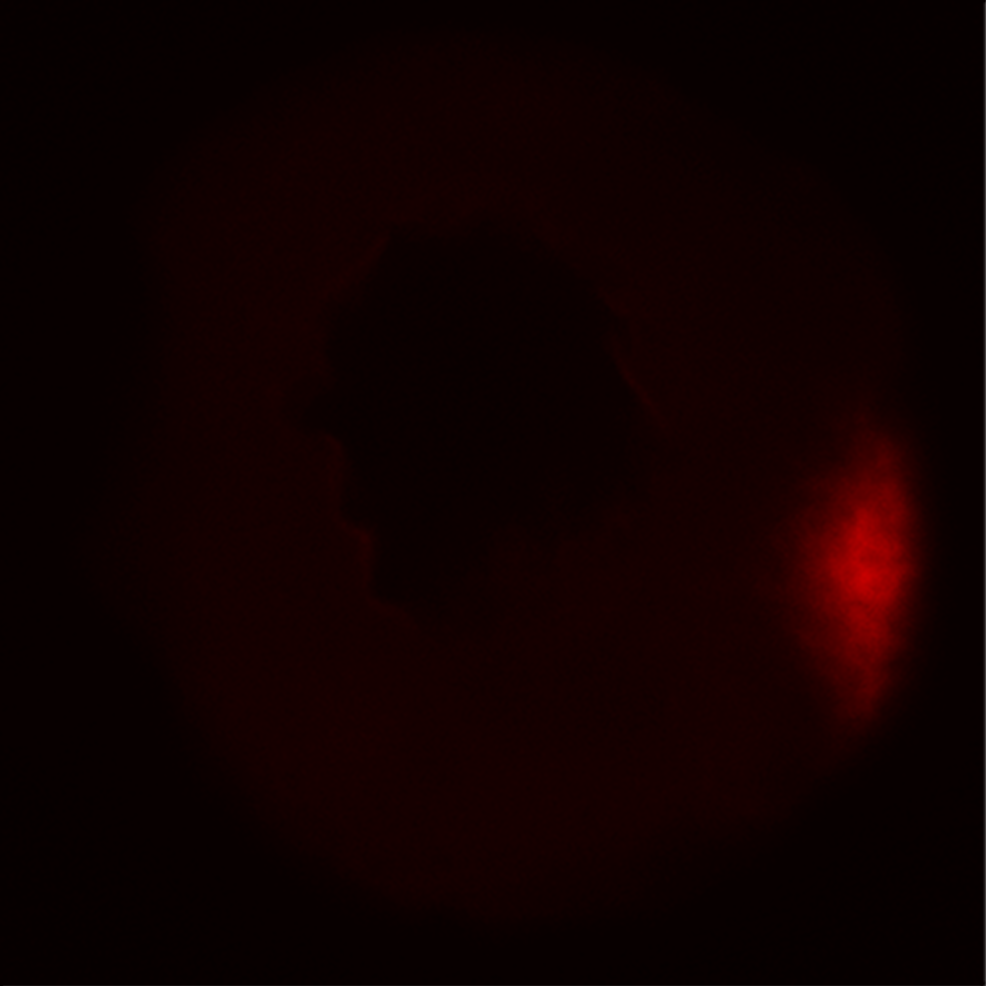

Supplement: Supplementary file 21 — Figure EV4 Source Data [file 44318_2025_442_MOESM21_ESM.zip › Figure_EV4/Figure EV4f/2 wt dazl .tif]

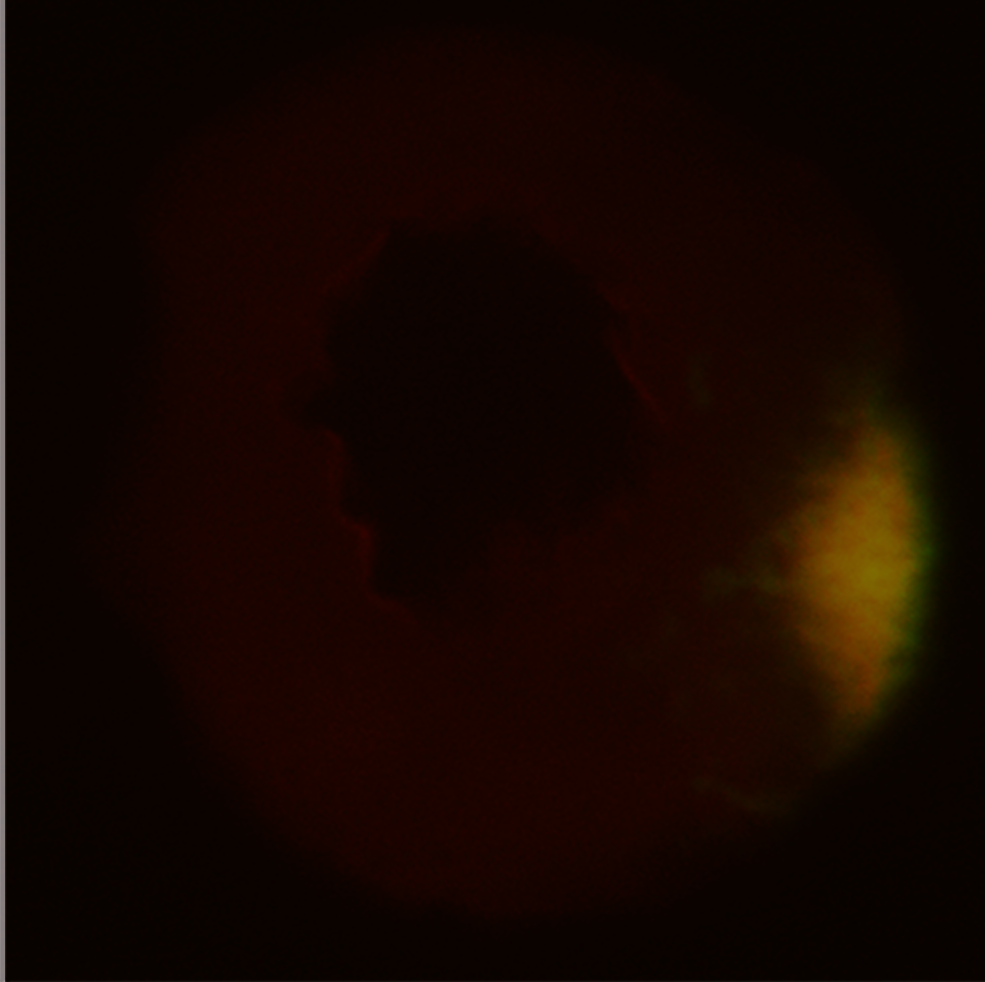

Supplement: Supplementary file 21 — Figure EV4 Source Data [file 44318_2025_442_MOESM21_ESM.zip › Figure_EV4/Figure EV4f/2 wt merge .tif]

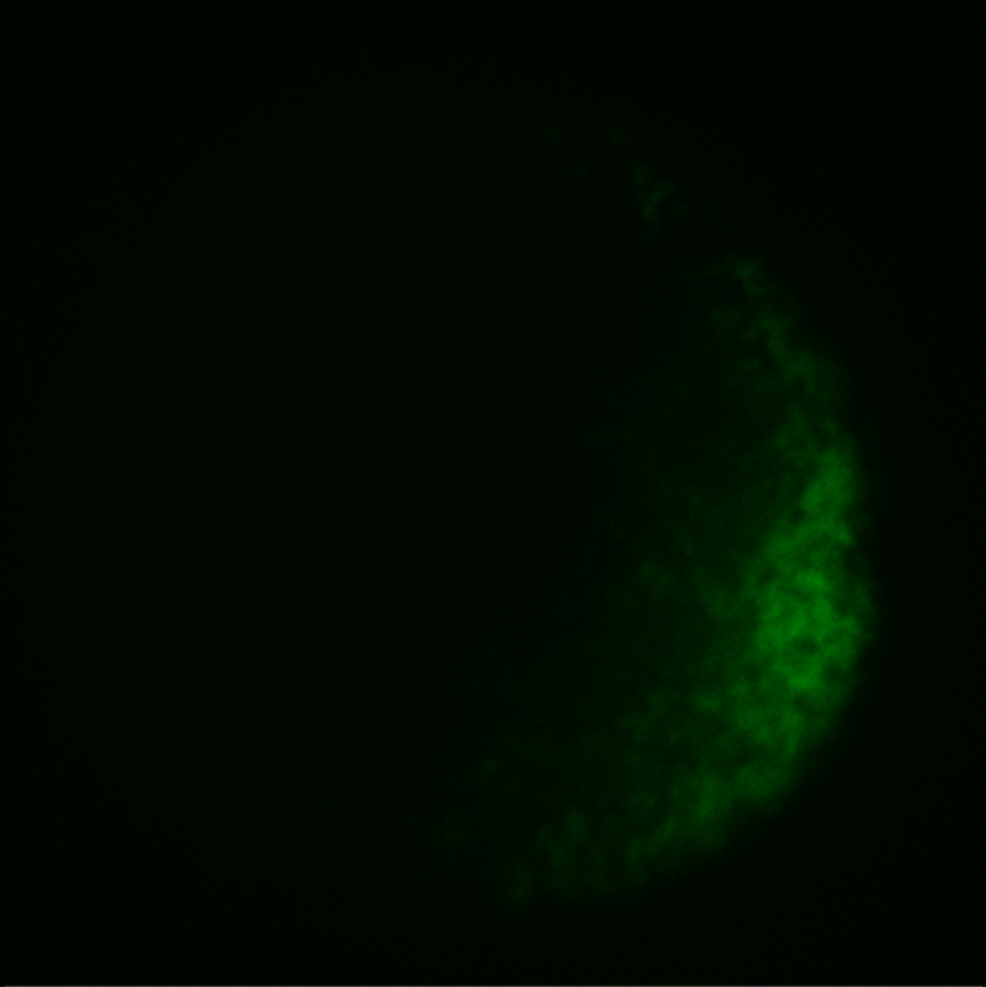

Supplement: Supplementary file 21 — Figure EV4 Source Data [file 44318_2025_442_MOESM21_ESM.zip › Figure_EV4/Figure EV4f/Mrbm24a 2 buc-gfp .tif]

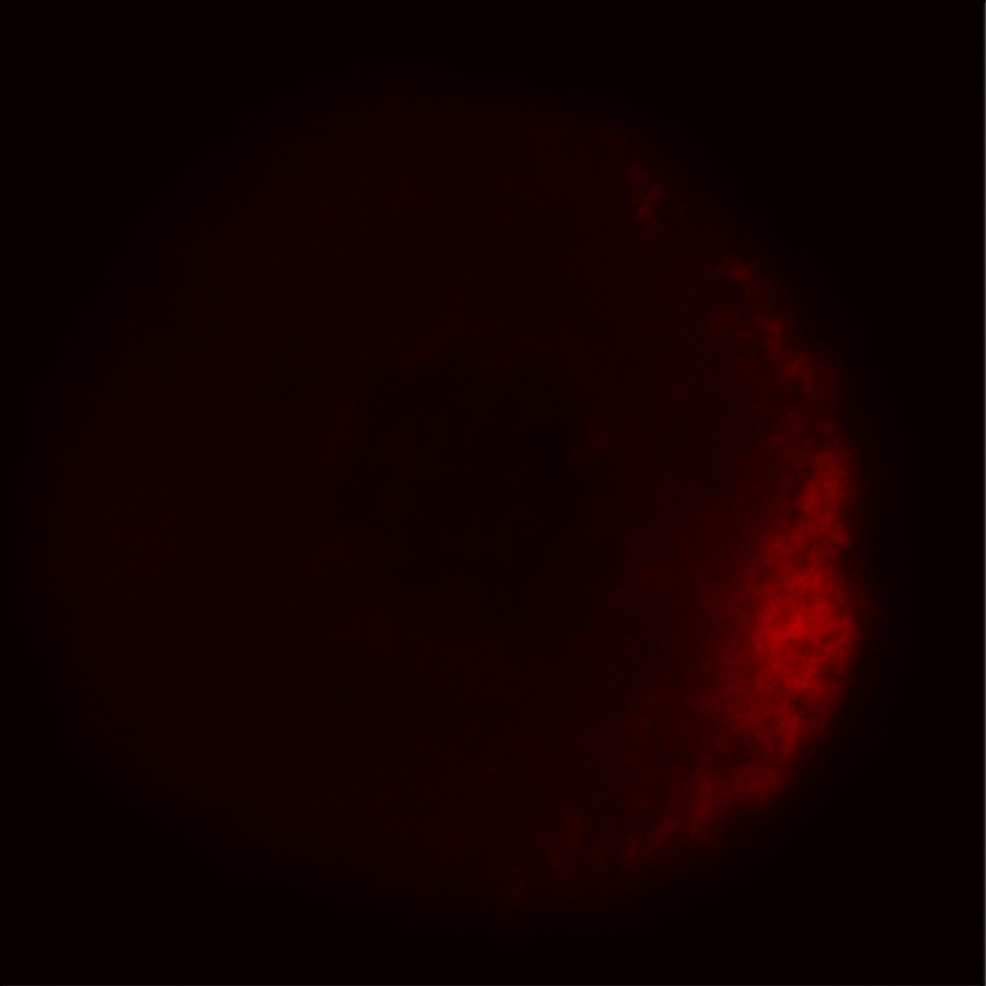

Supplement: Supplementary file 21 — Figure EV4 Source Data [file 44318_2025_442_MOESM21_ESM.zip › Figure_EV4/Figure EV4f/Mrbm24a 2 dazl.tif]

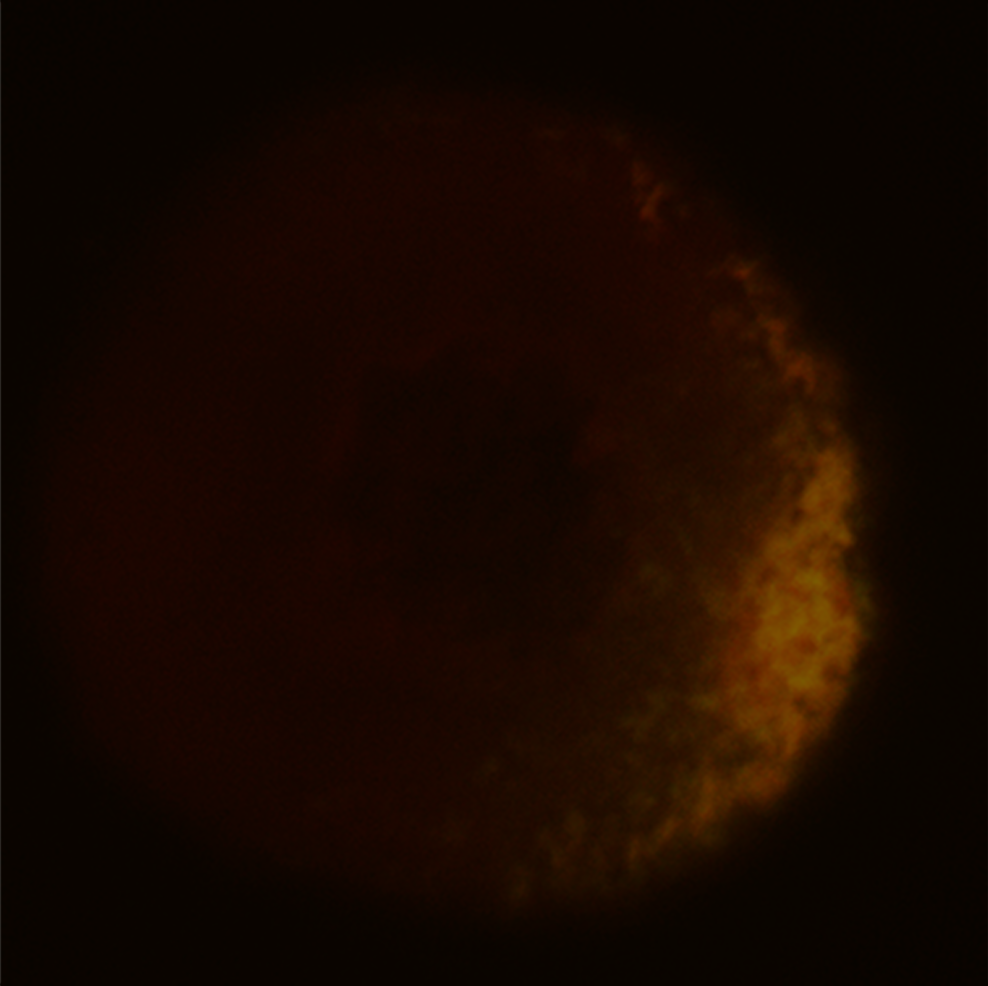

Supplement: Supplementary file 21 — Figure EV4 Source Data [file 44318_2025_442_MOESM21_ESM.zip › Figure_EV4/Figure EV4f/Mrbm24a 2 merge .tif]

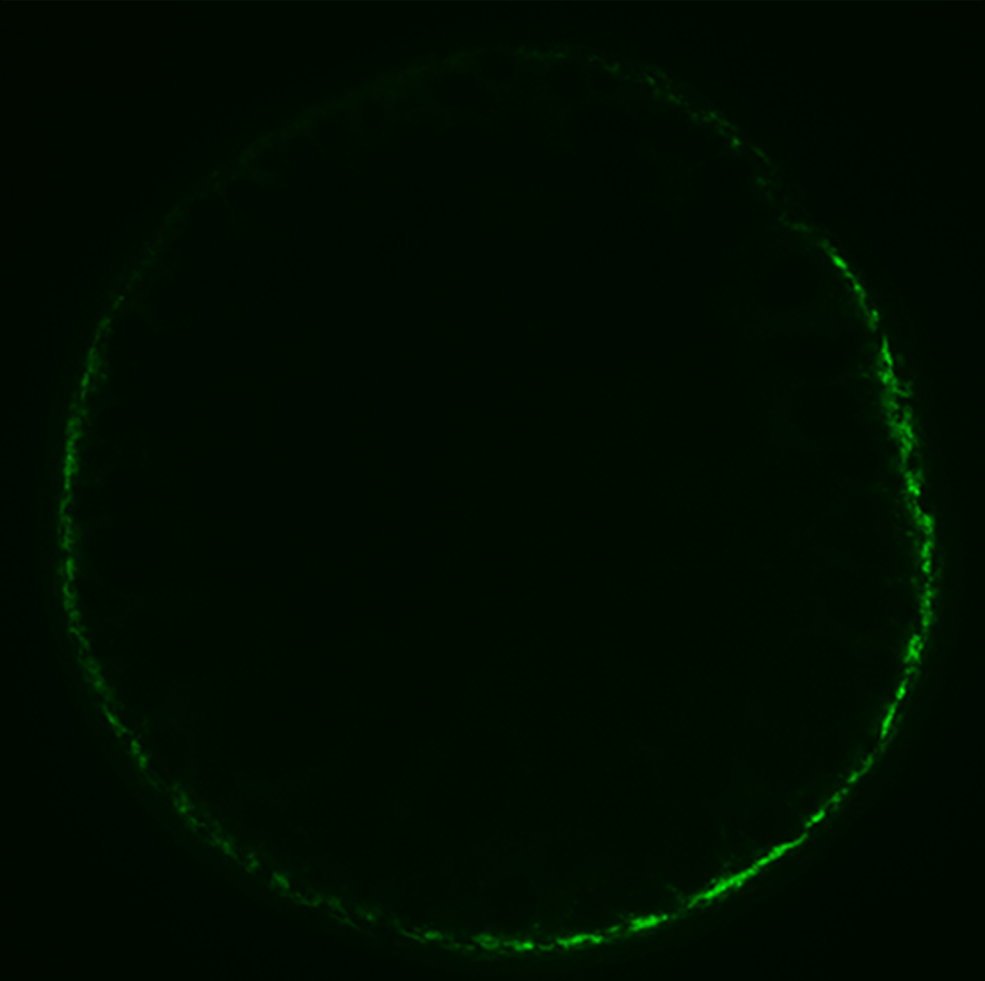

Supplement: Supplementary file 21 — Figure EV4 Source Data [file 44318_2025_442_MOESM21_ESM.zip › Figure_EV4/Figure EV4g/3 wt buc-gfp .tif]

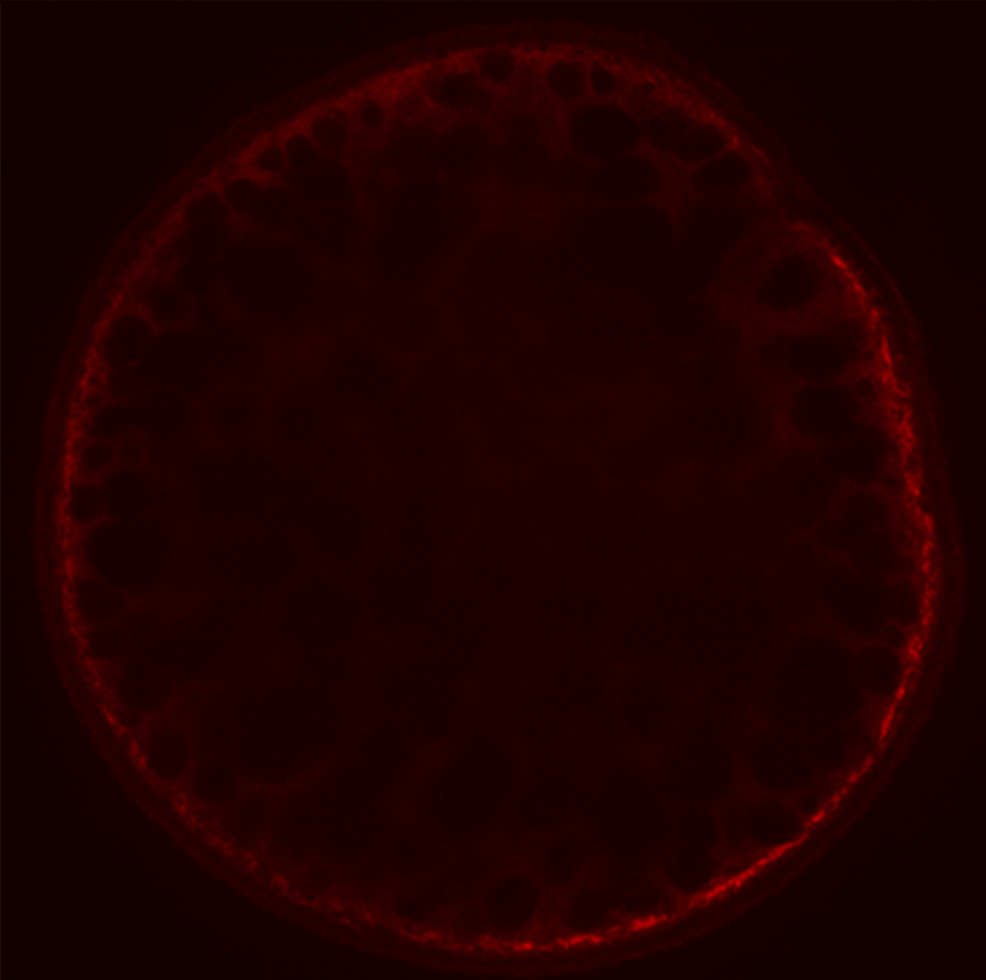

Supplement: Supplementary file 21 — Figure EV4 Source Data [file 44318_2025_442_MOESM21_ESM.zip › Figure_EV4/Figure EV4g/3 wt dazl .tif]

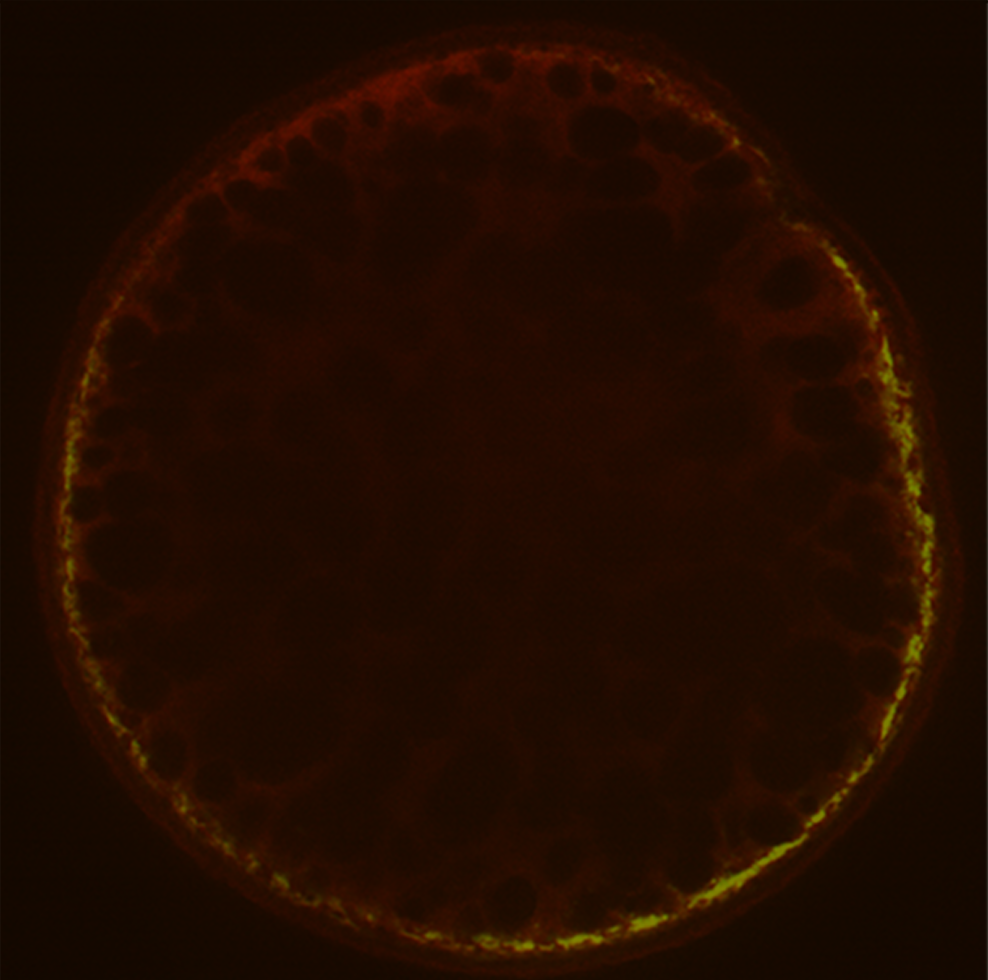

Supplement: Supplementary file 21 — Figure EV4 Source Data [file 44318_2025_442_MOESM21_ESM.zip › Figure_EV4/Figure EV4g/3 wt merge .tif]

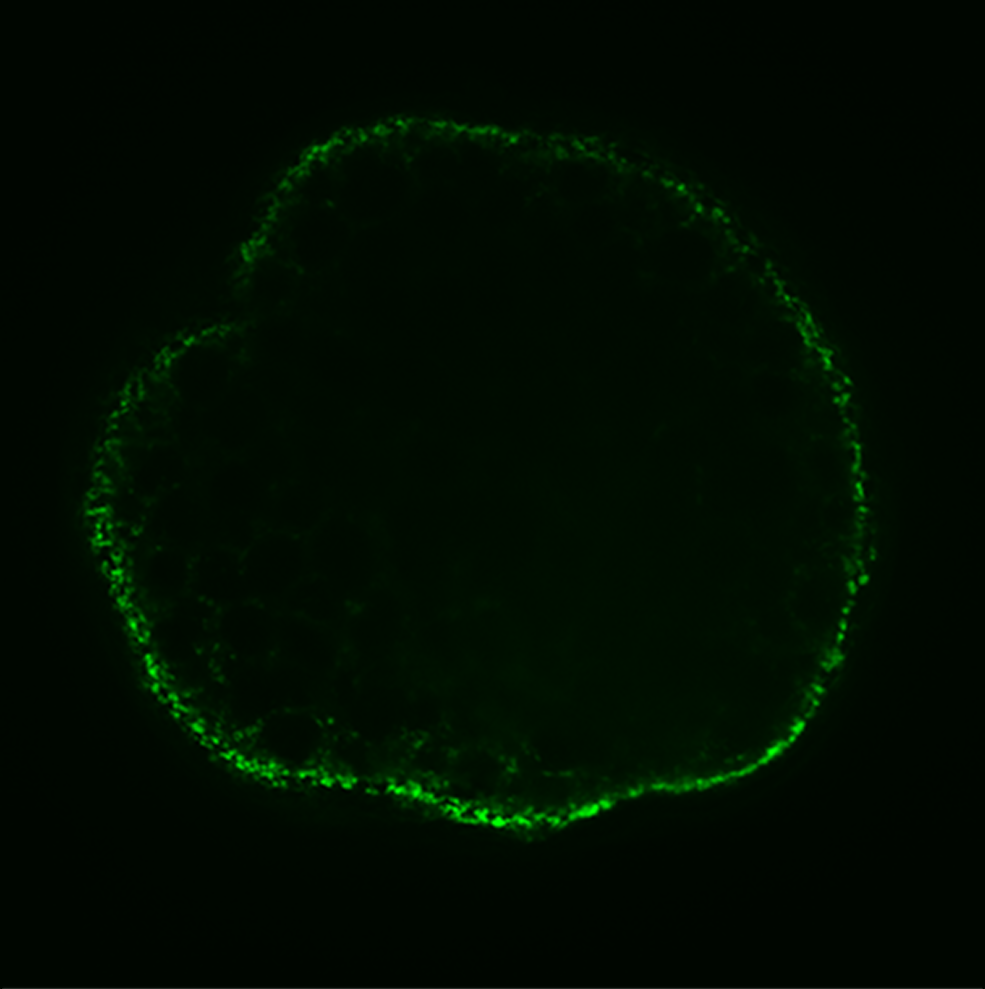

Supplement: Supplementary file 21 — Figure EV4 Source Data [file 44318_2025_442_MOESM21_ESM.zip › Figure_EV4/Figure EV4g/Mrbm24a 3 buc-gfp .tif]

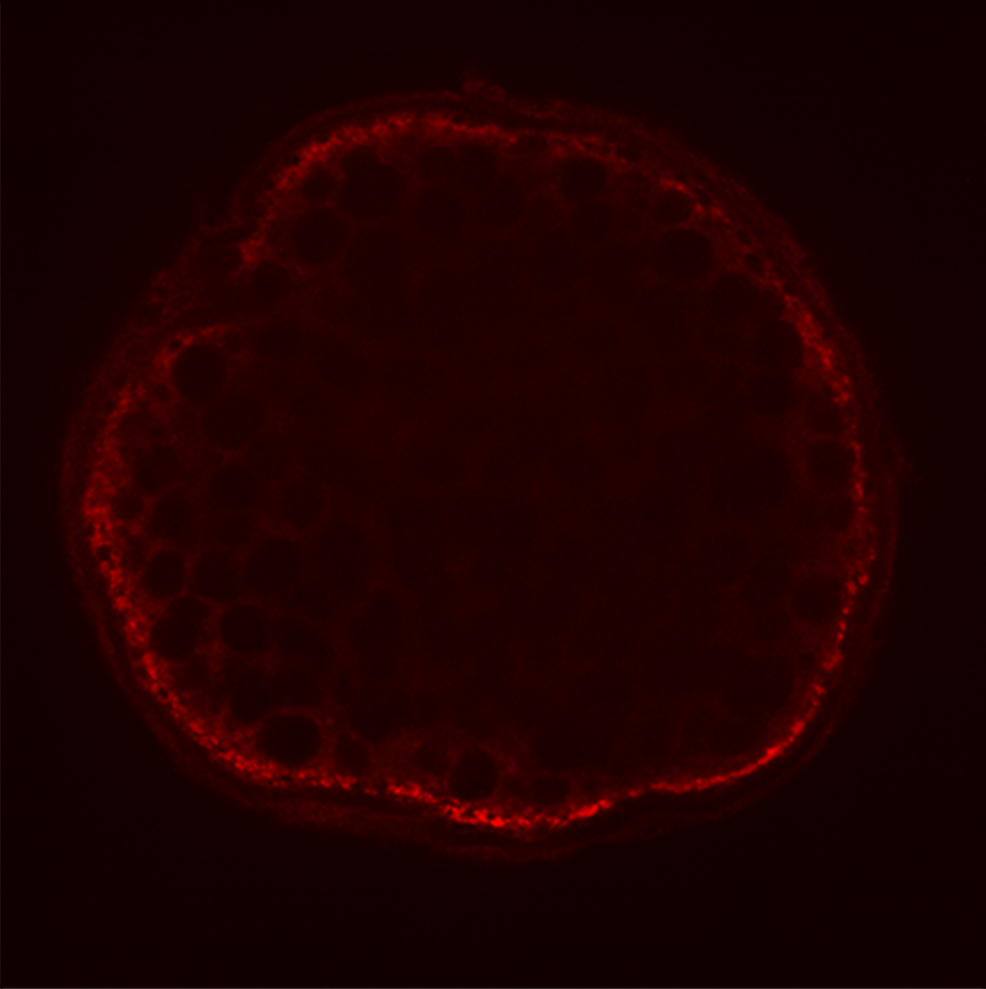

Supplement: Supplementary file 21 — Figure EV4 Source Data [file 44318_2025_442_MOESM21_ESM.zip › Figure_EV4/Figure EV4g/Mrbm24a 3 dazl .tif]

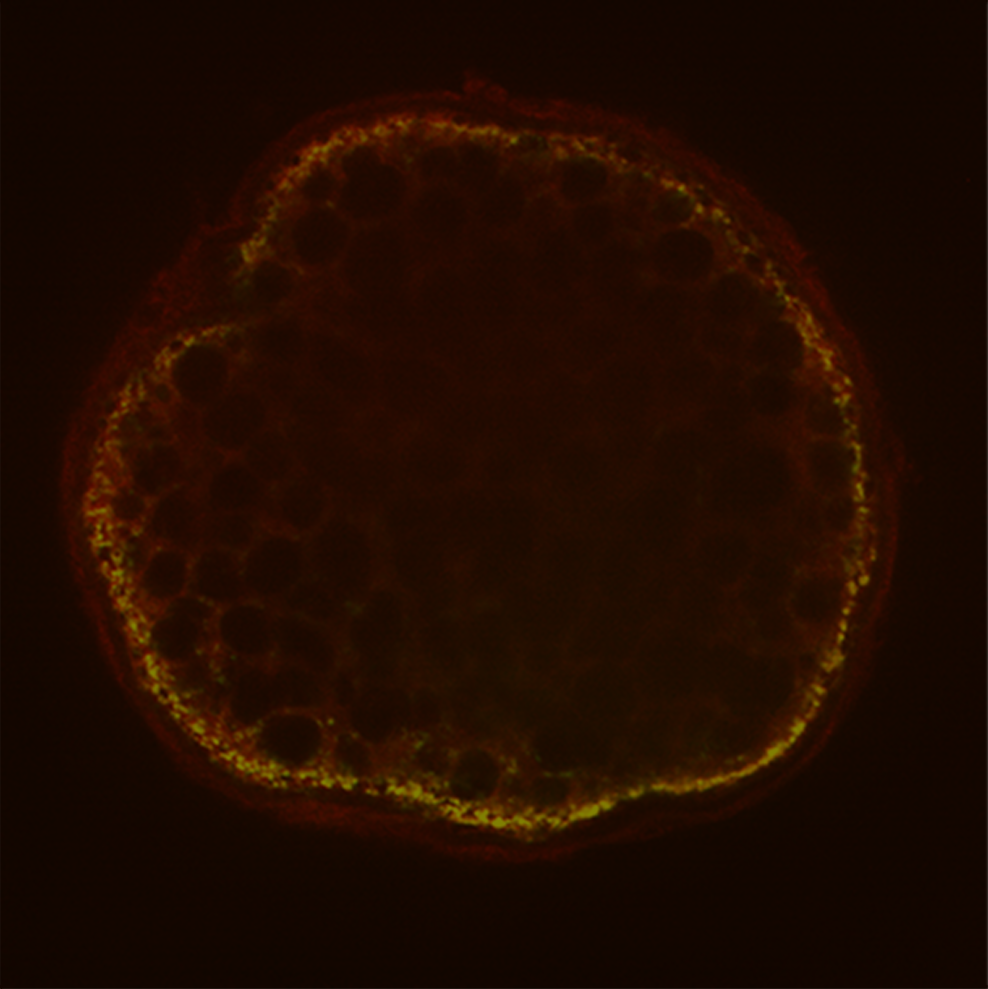

Supplement: Supplementary file 21 — Figure EV4 Source Data [file 44318_2025_442_MOESM21_ESM.zip › Figure_EV4/Figure EV4g/Mrbm24a 3 merge .tif]

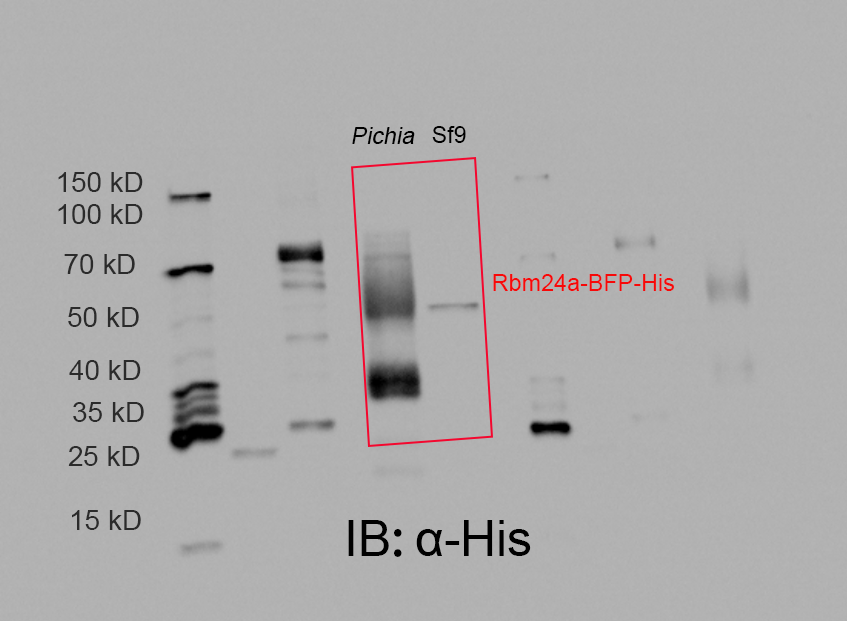

Supplement: Supplementary file 22 — Figure EV5 Source Data [file 44318_2025_442_MOESM22_ESM.zip › Figure_EV5/Figure EV5a/Pichia rbm24a sf9 rbm24a.tif]

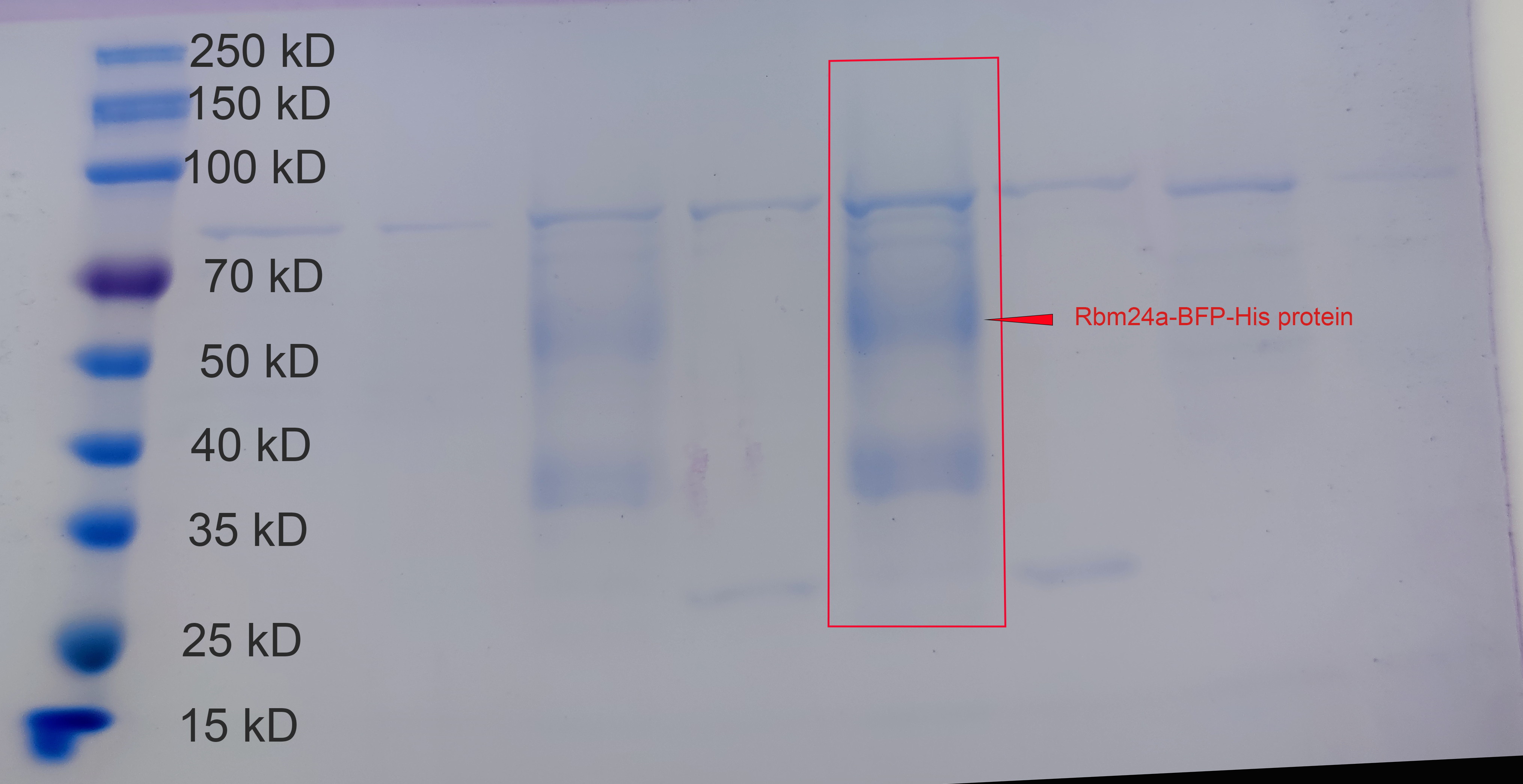

Supplement: Supplementary file 22 — Figure EV5 Source Data [file 44318_2025_442_MOESM22_ESM.zip › Figure_EV5/Figure EV5a/Pichia.tif]

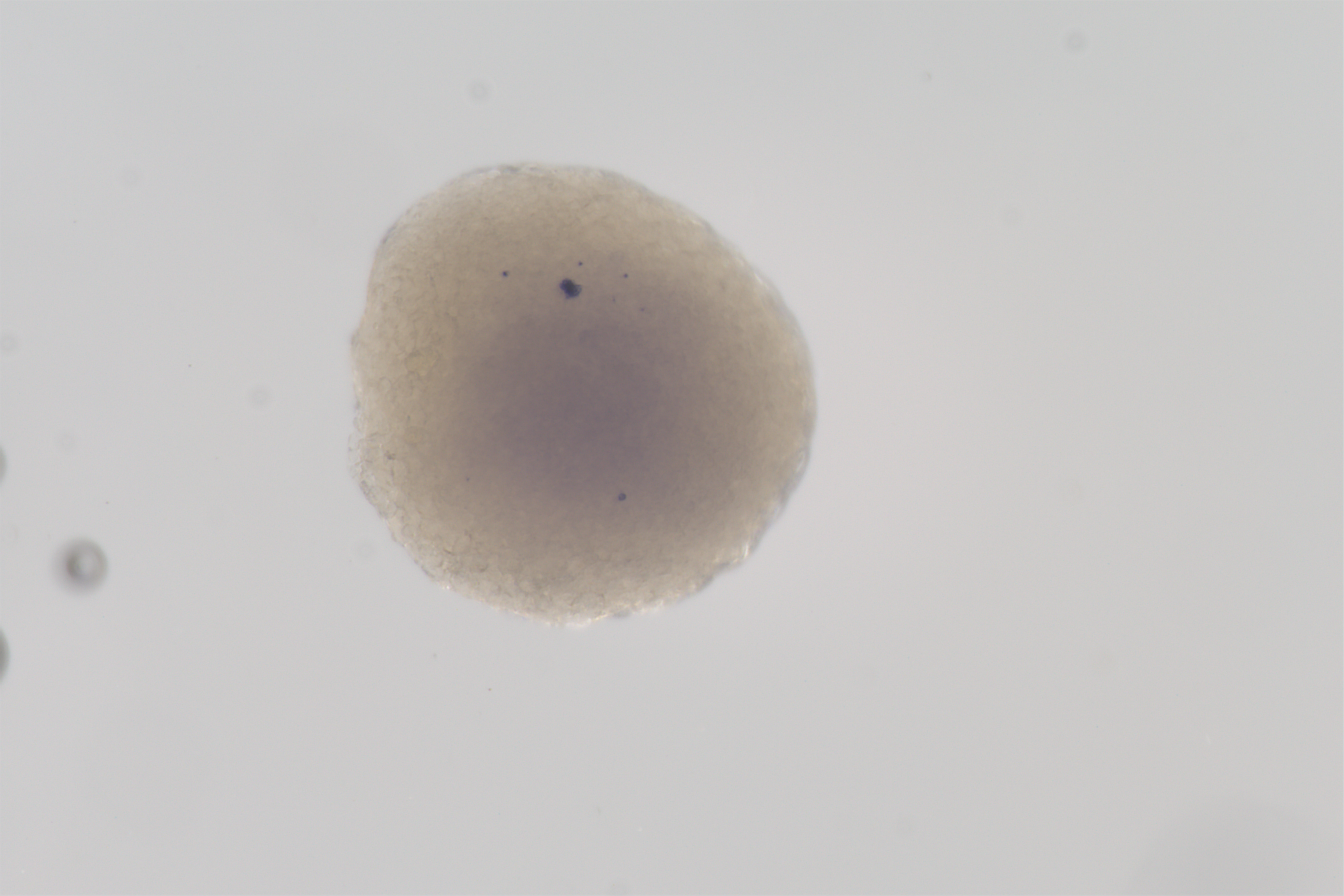

Supplement: Supplementary file 22 — Figure EV5 Source Data [file 44318_2025_442_MOESM22_ESM.zip › Figure_EV5/Figure EV5b/protein inj rbm24a.tif]

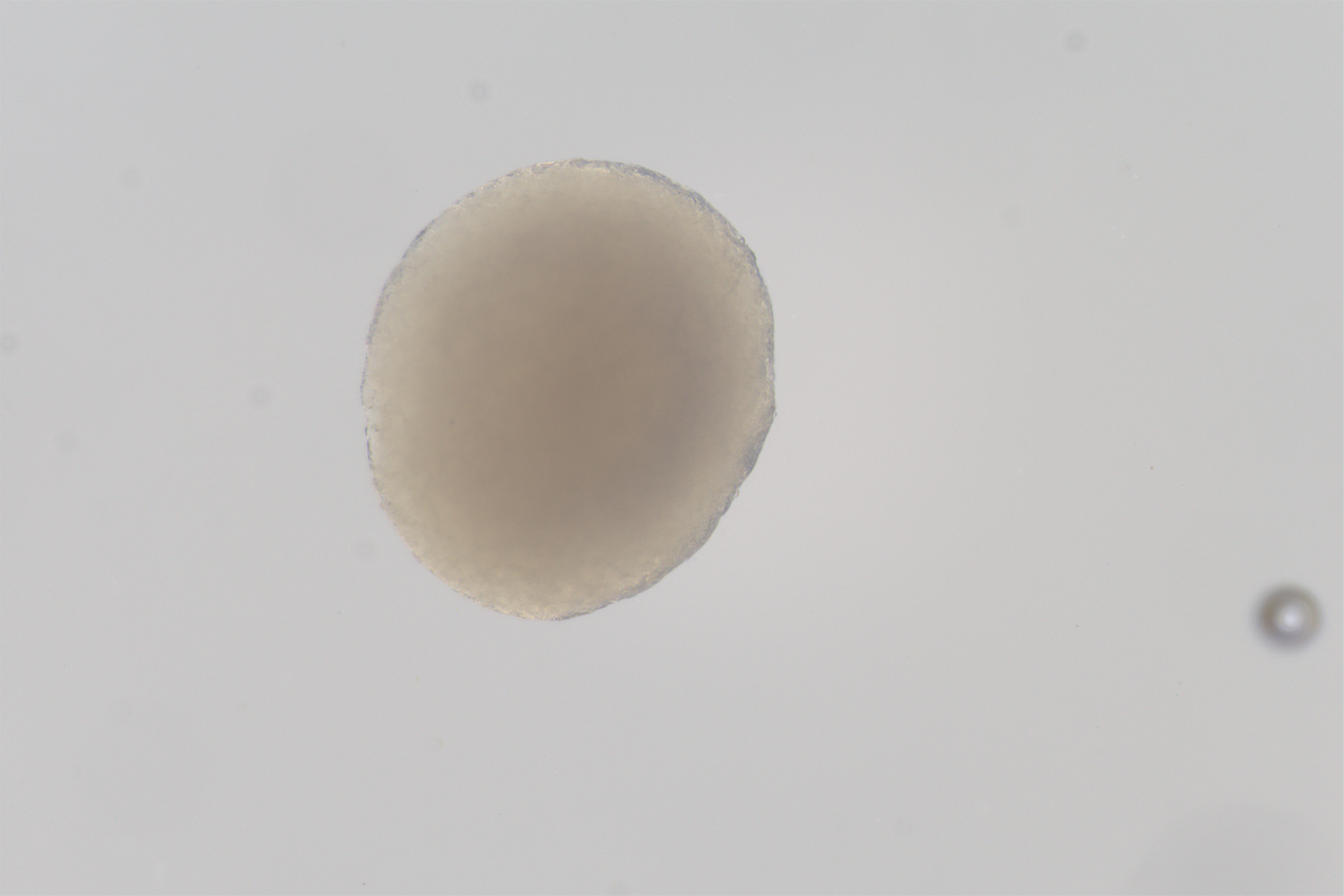

Supplement: Supplementary file 22 — Figure EV5 Source Data [file 44318_2025_442_MOESM22_ESM.zip › Figure_EV5/Figure EV5b/protein un .tif]

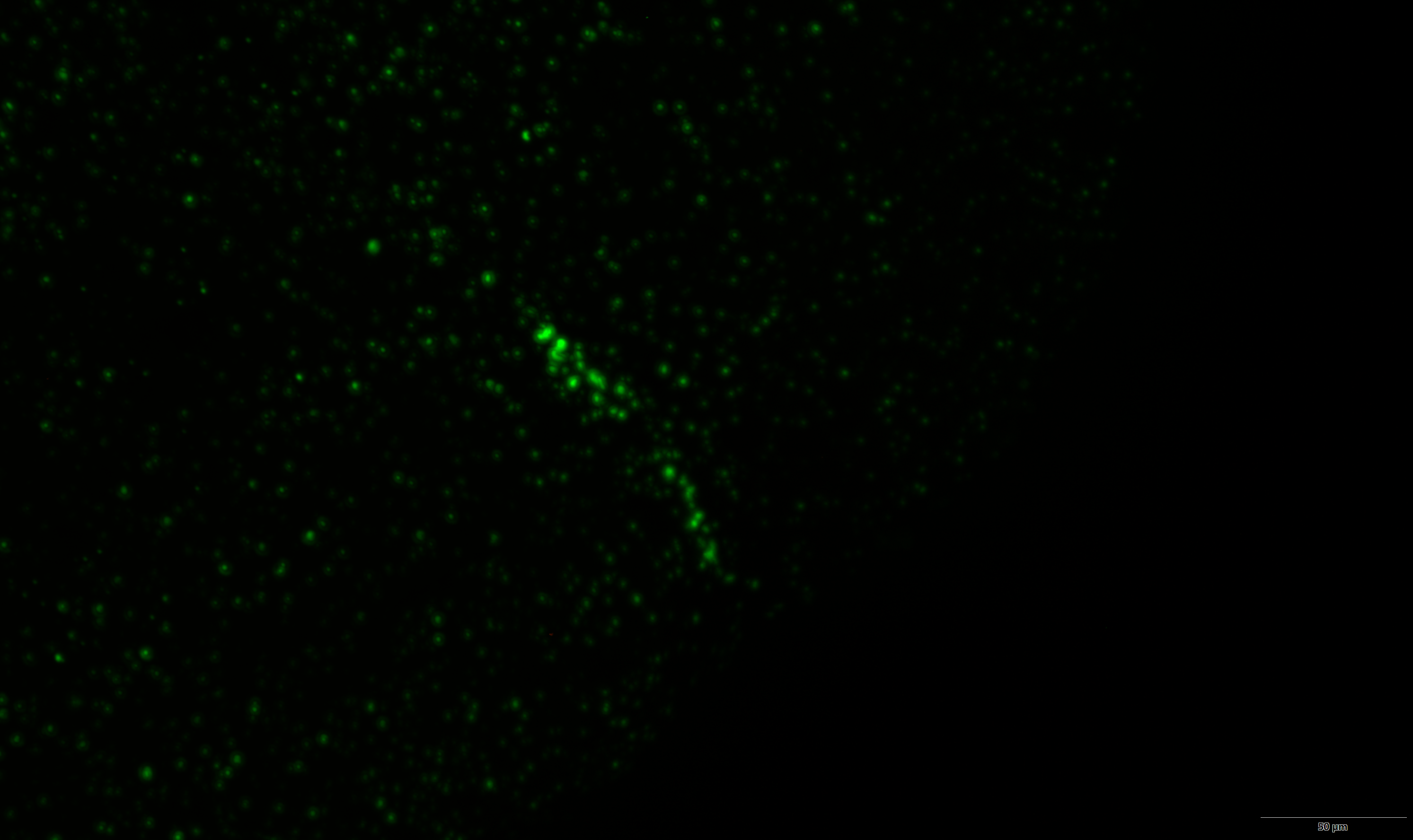

Supplement: Supplementary file 22 — Figure EV5 Source Data [file 44318_2025_442_MOESM22_ESM.zip › Figure_EV5/Figure EV5c/M inj Rbm24a protein.tif]

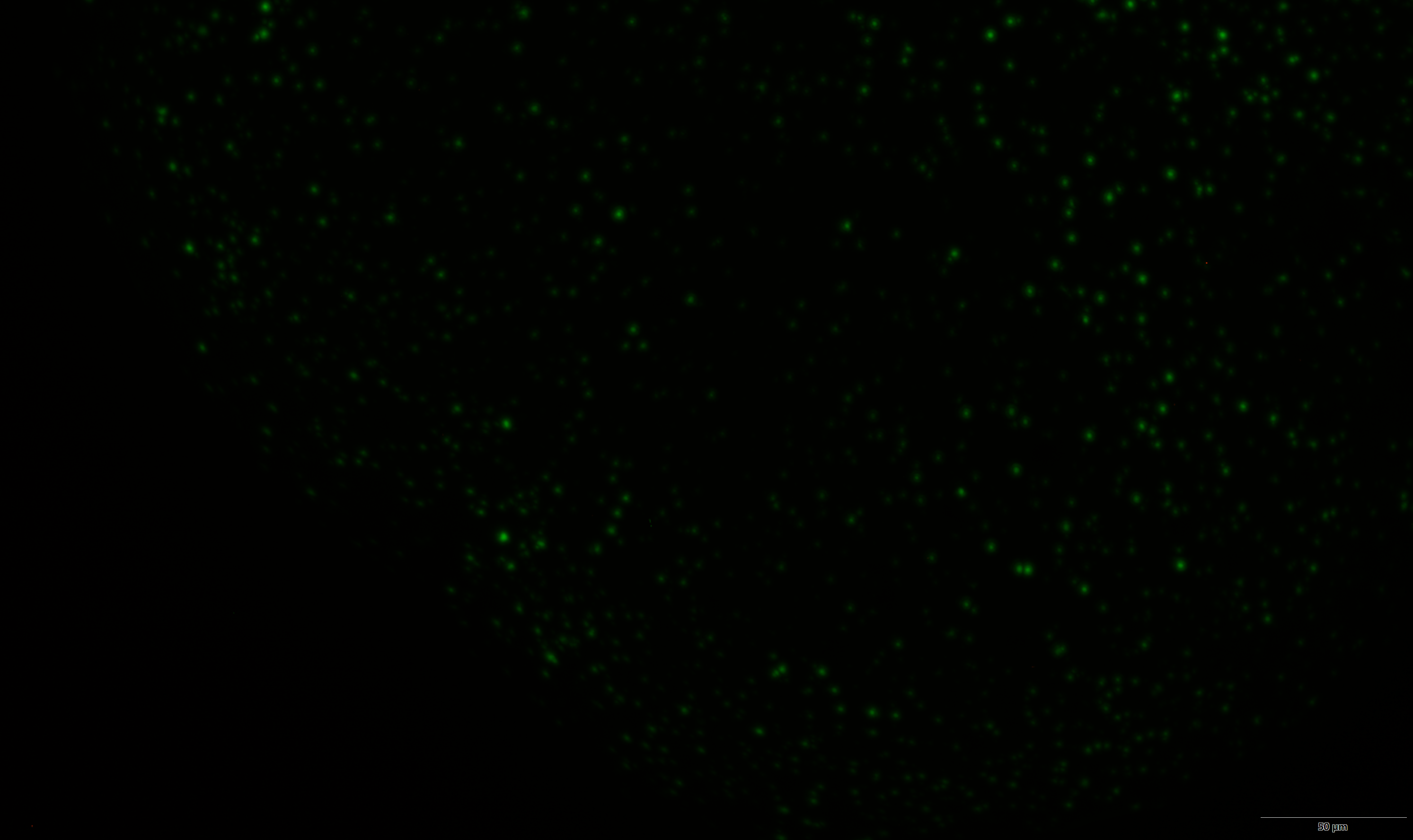

Supplement: Supplementary file 22 — Figure EV5 Source Data [file 44318_2025_442_MOESM22_ESM.zip › Figure_EV5/Figure EV5c/M un.tif]

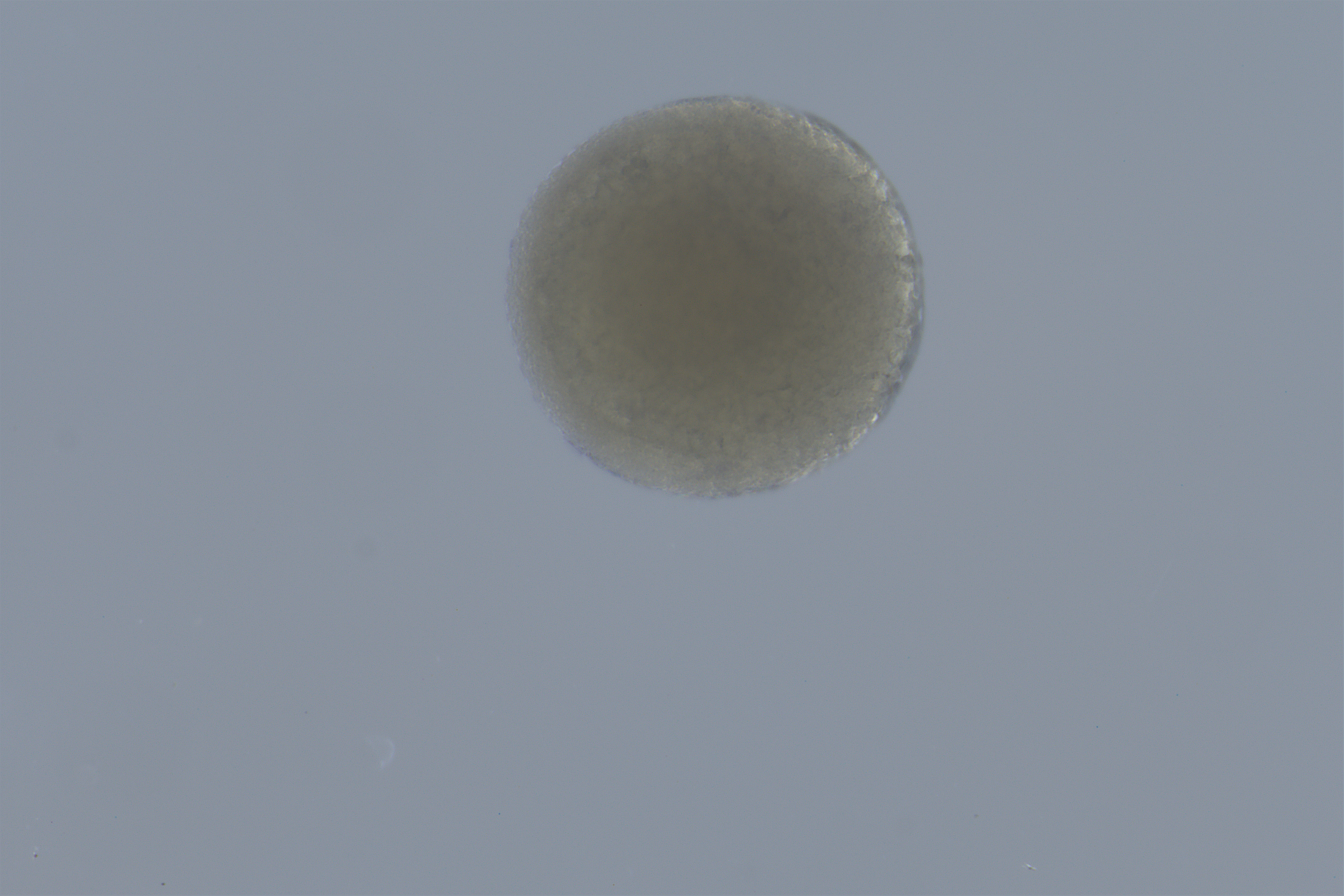

Supplement: Supplementary file 22 — Figure EV5 Source Data [file 44318_2025_442_MOESM22_ESM.zip › Figure_EV5/Figure EV5d/Mrbm24a inj buc mRNA.tif]

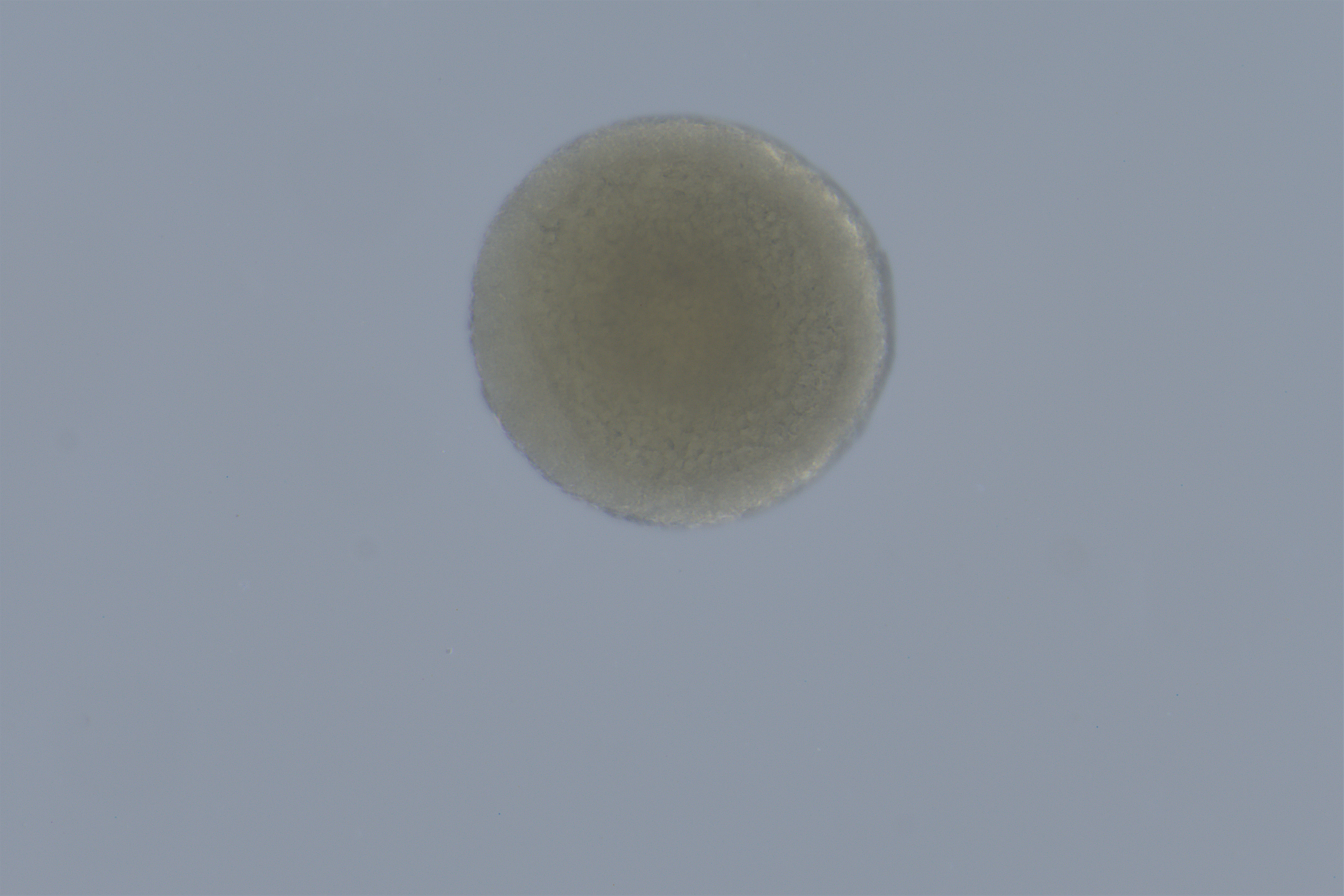

Supplement: Supplementary file 22 — Figure EV5 Source Data [file 44318_2025_442_MOESM22_ESM.zip › Figure_EV5/Figure EV5d/Mrbm24a inj rbm24a mRNA .tif]

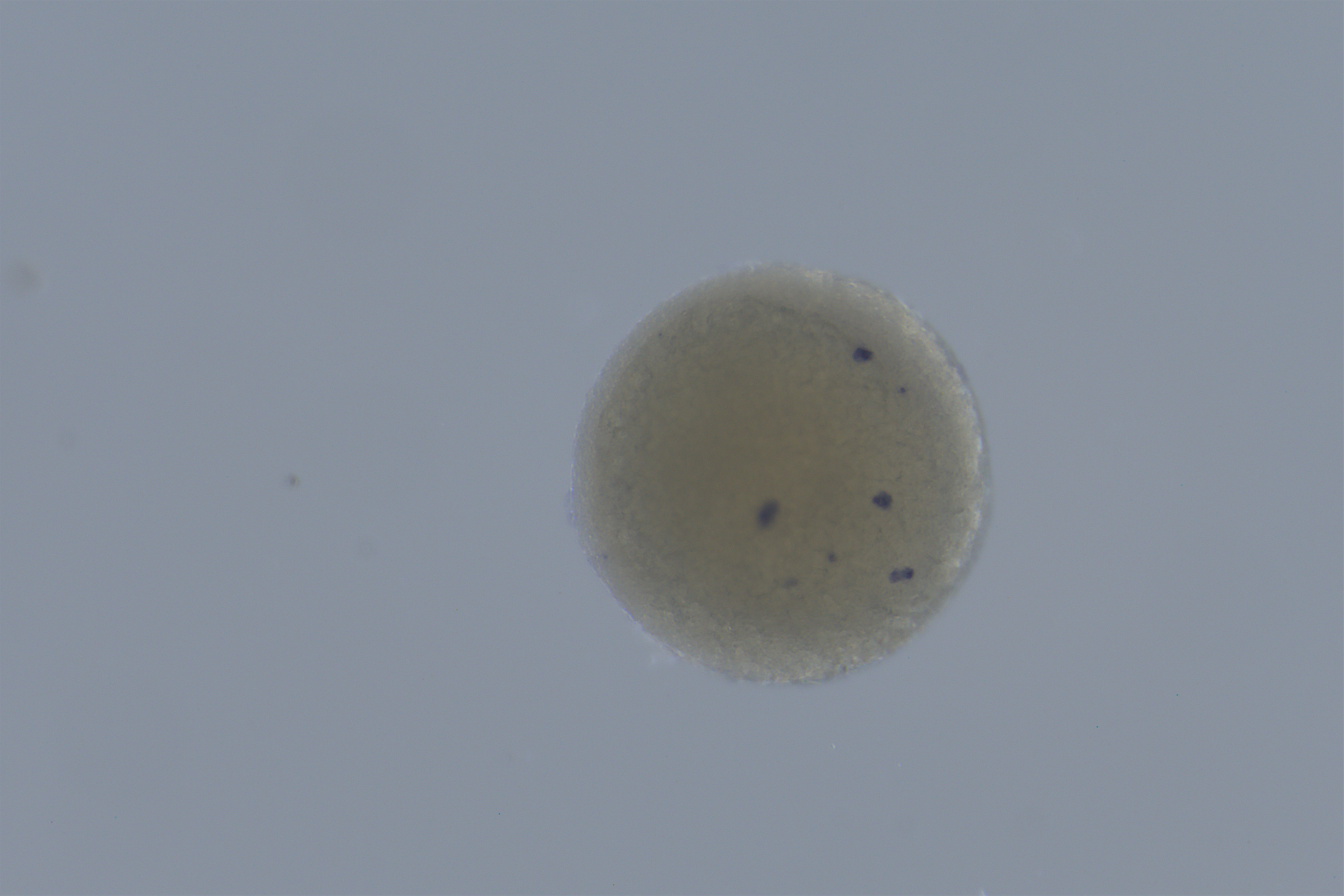

Supplement: Supplementary file 22 — Figure EV5 Source Data [file 44318_2025_442_MOESM22_ESM.zip › Figure_EV5/Figure EV5d/Mrbm24a inj rbm24a-gfp buc-gfp mRNA mRNA .tif]

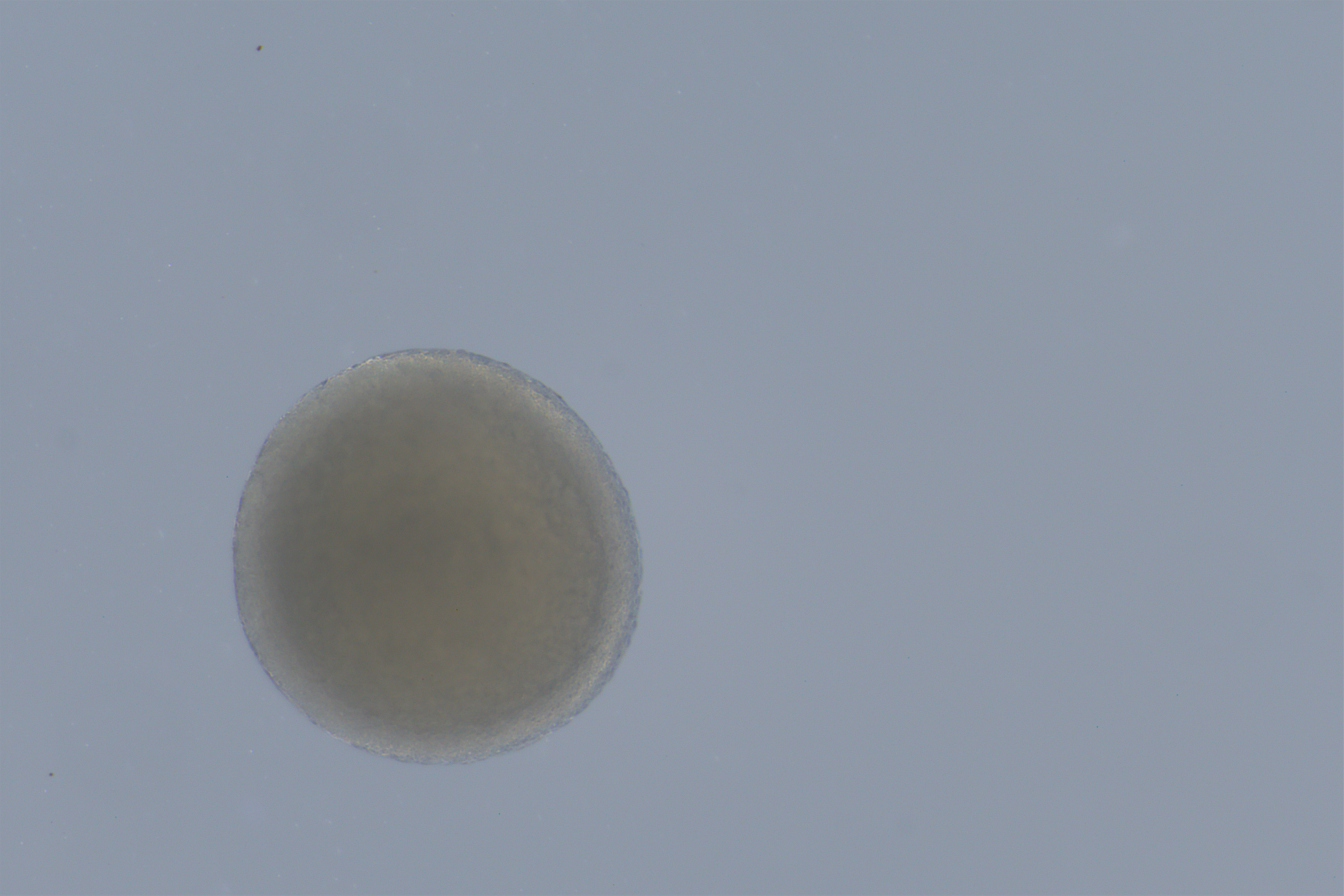

Supplement: Supplementary file 22 — Figure EV5 Source Data [file 44318_2025_442_MOESM22_ESM.zip › Figure_EV5/Figure EV5d/Mrbm24a un.tif]

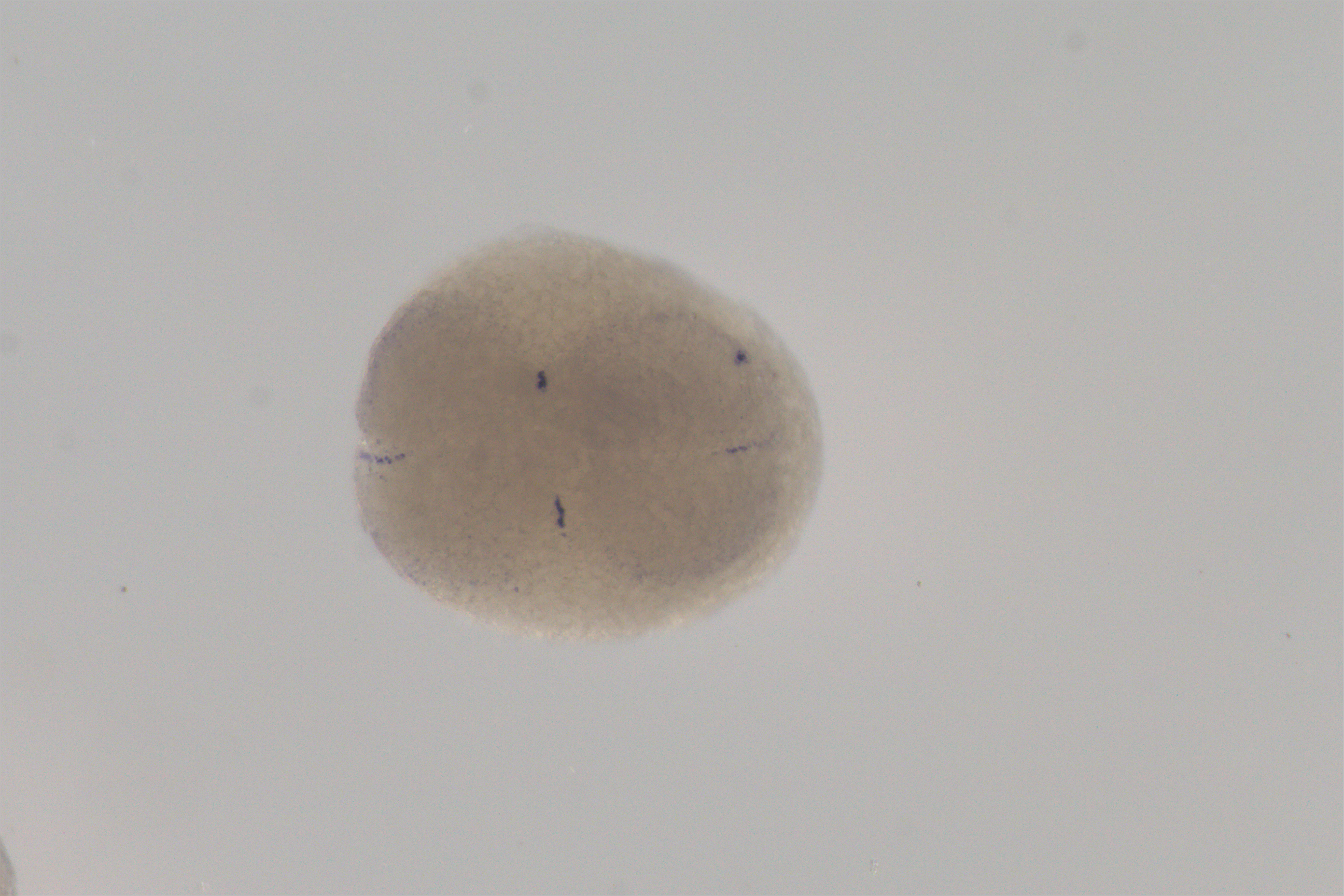

Supplement: Supplementary file 22 — Figure EV5 Source Data [file 44318_2025_442_MOESM22_ESM.zip › Figure_EV5/Figure EV5e/4-cell inj Rbm24a .tif]

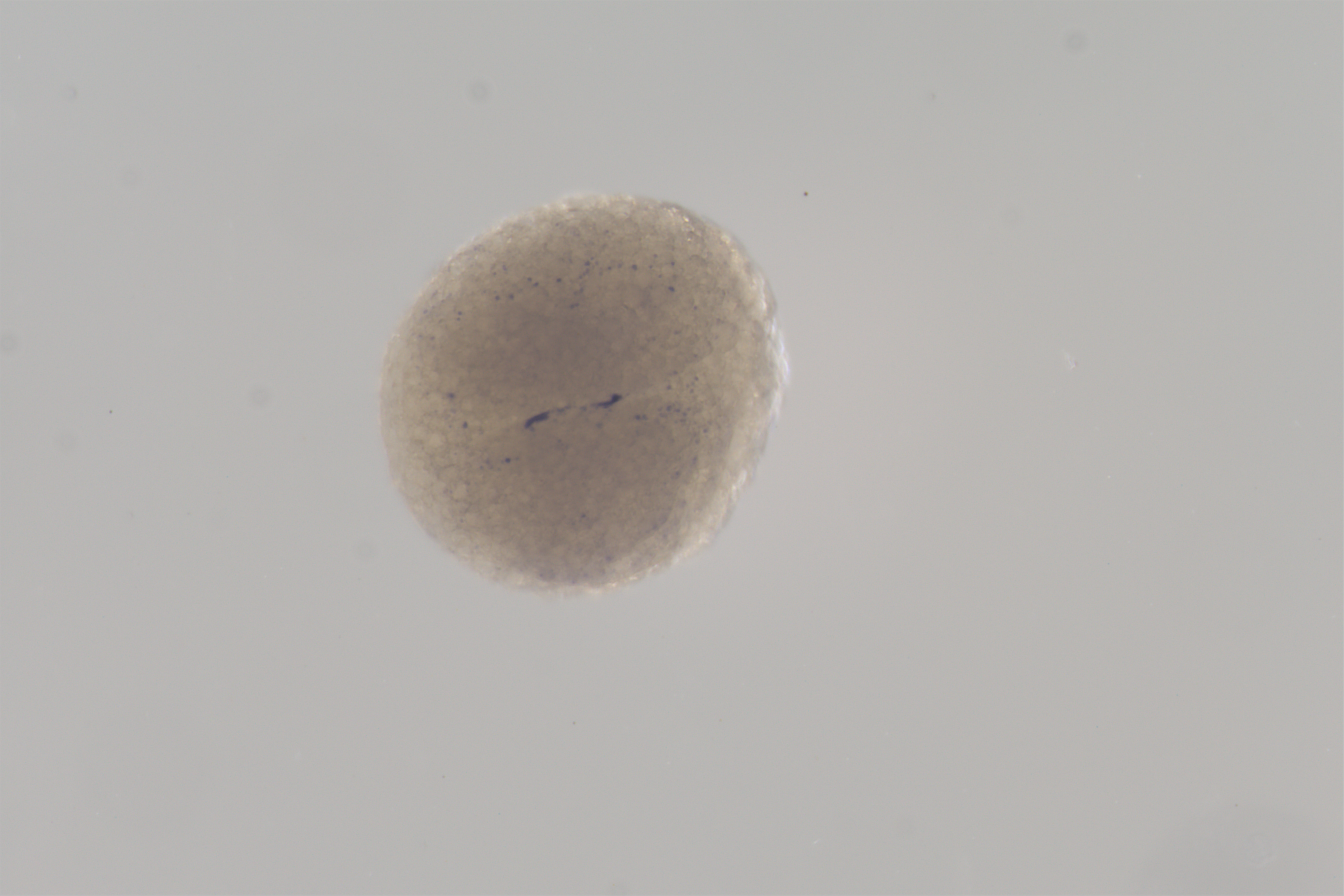

Supplement: Supplementary file 22 — Figure EV5 Source Data [file 44318_2025_442_MOESM22_ESM.zip › Figure_EV5/Figure EV5e/4-cell inj Rbm24a 2.tif]

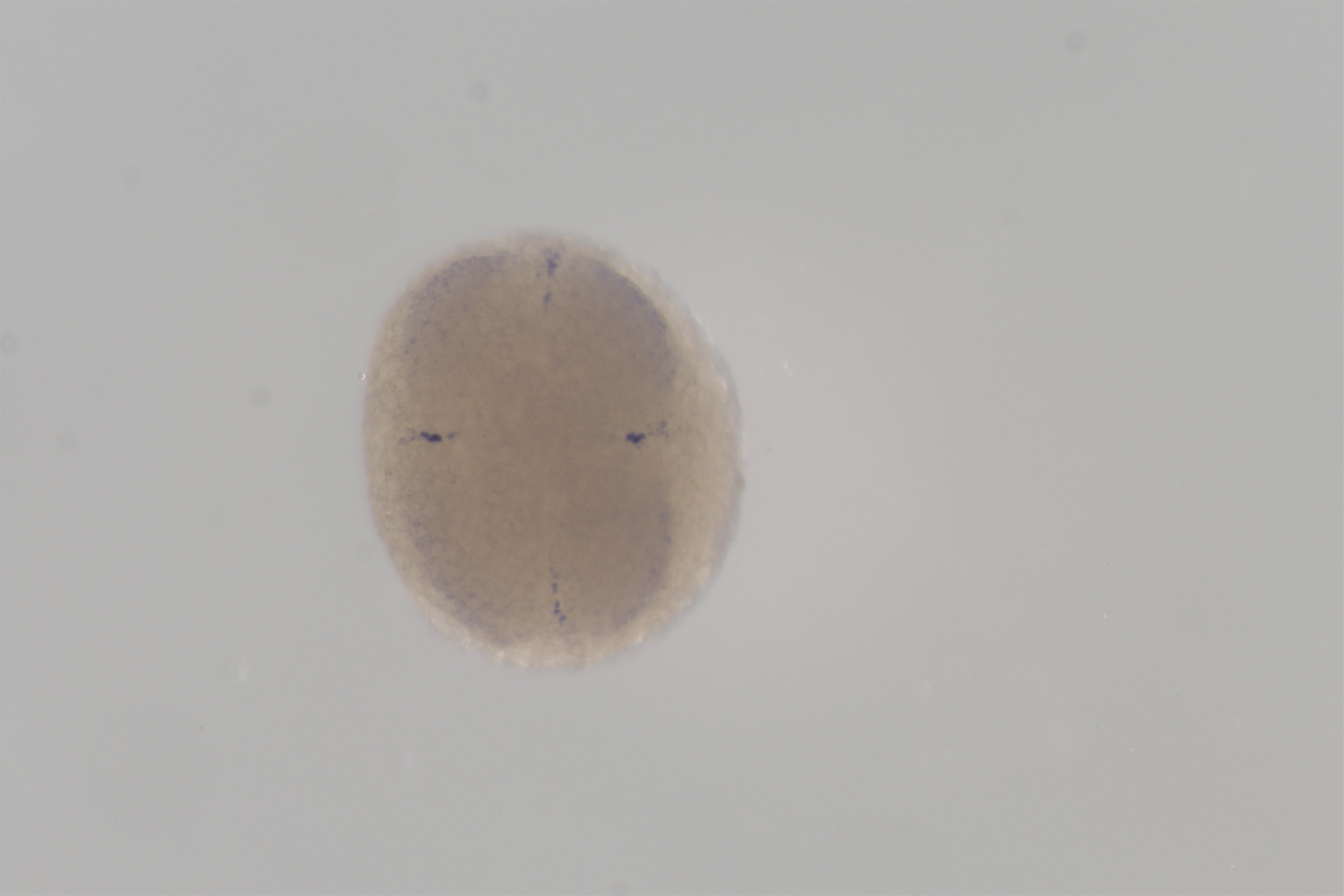

Supplement: Supplementary file 22 — Figure EV5 Source Data [file 44318_2025_442_MOESM22_ESM.zip › Figure_EV5/Figure EV5e/4-cell uninj.tif]

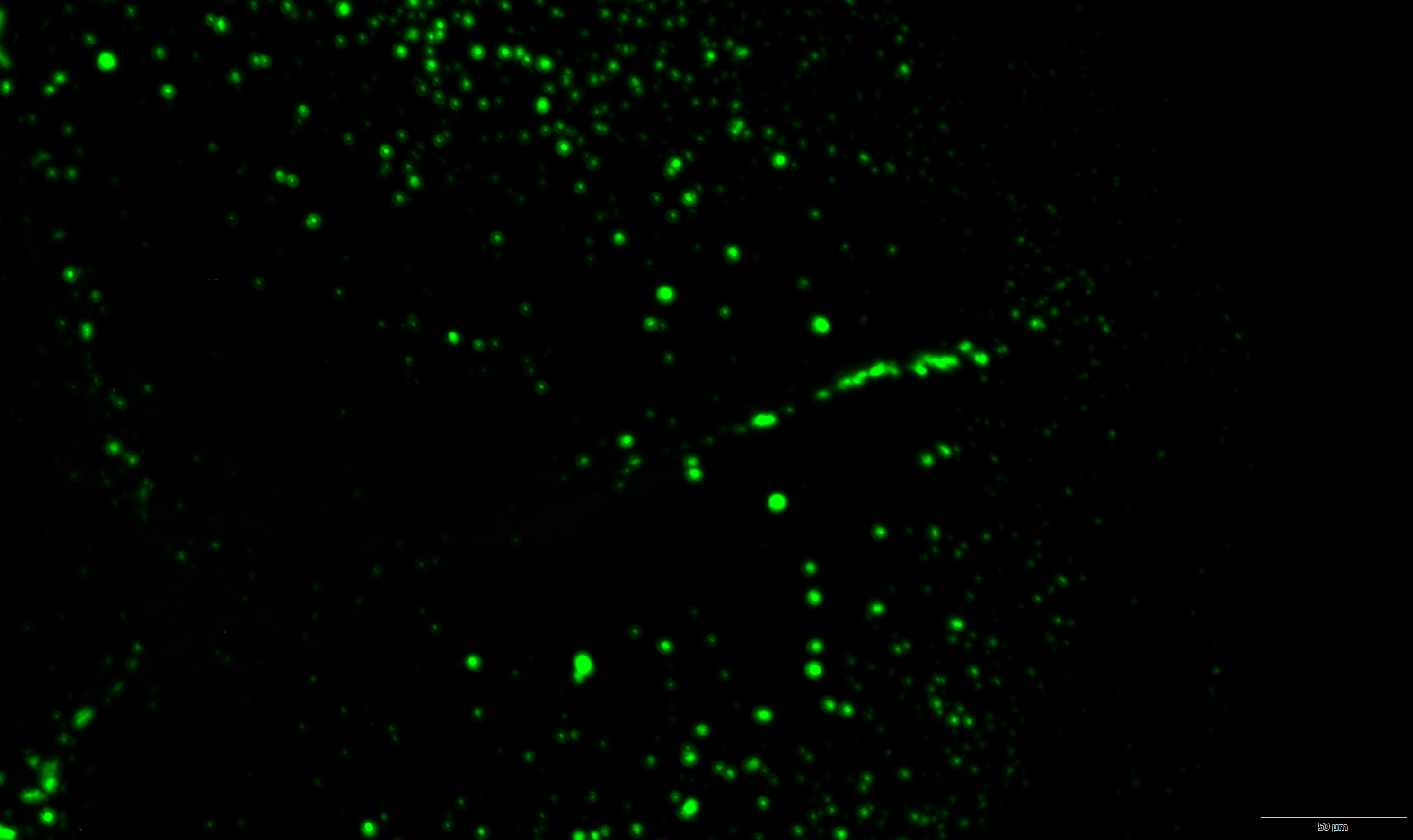

Supplement: Supplementary file 22 — Figure EV5 Source Data [file 44318_2025_442_MOESM22_ESM.zip › Figure_EV5/Figure EV5f/sibling inj Rbm24a.tif]

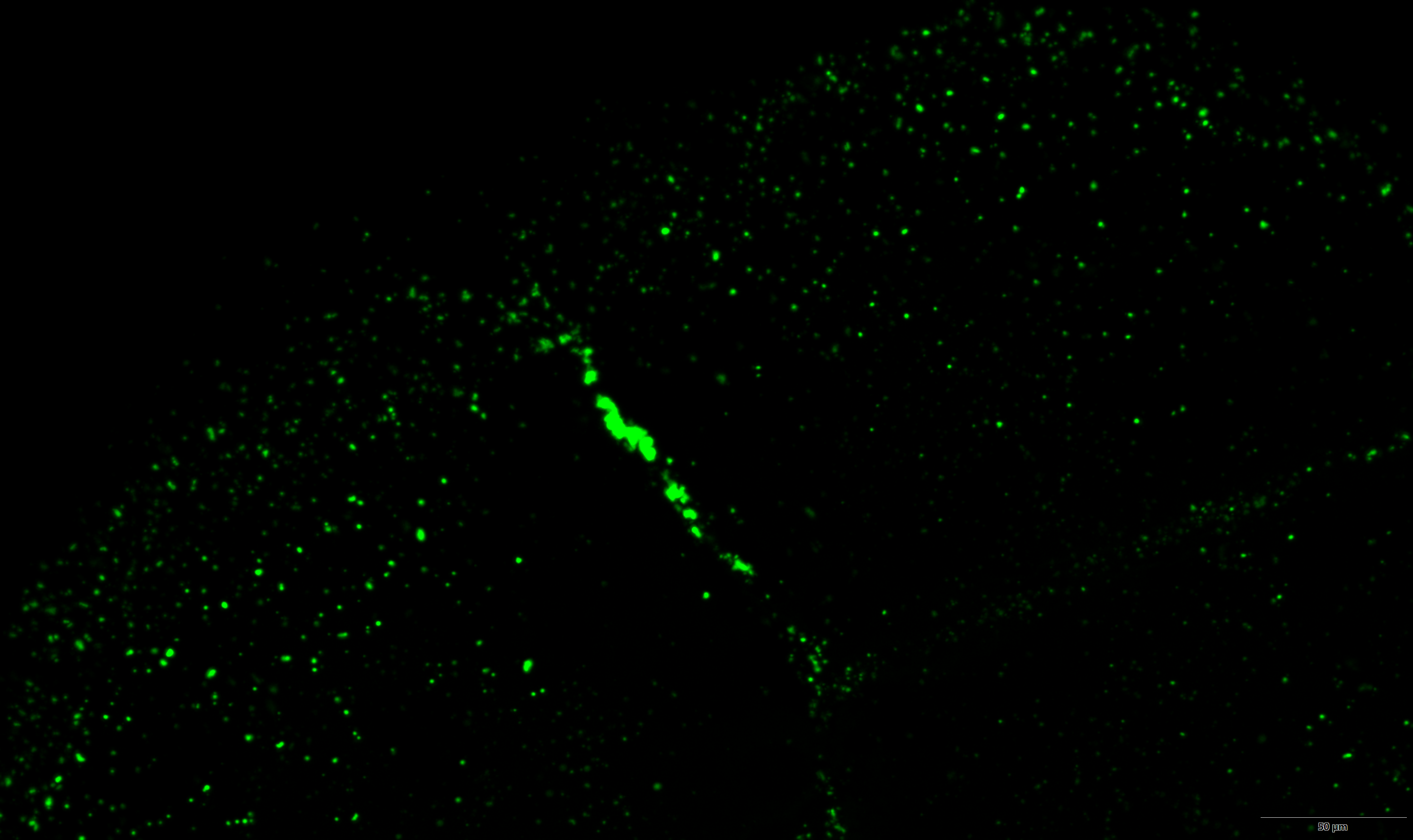

Supplement: Supplementary file 22 — Figure EV5 Source Data [file 44318_2025_442_MOESM22_ESM.zip › Figure_EV5/Figure EV5f/sibling uninj.tif]

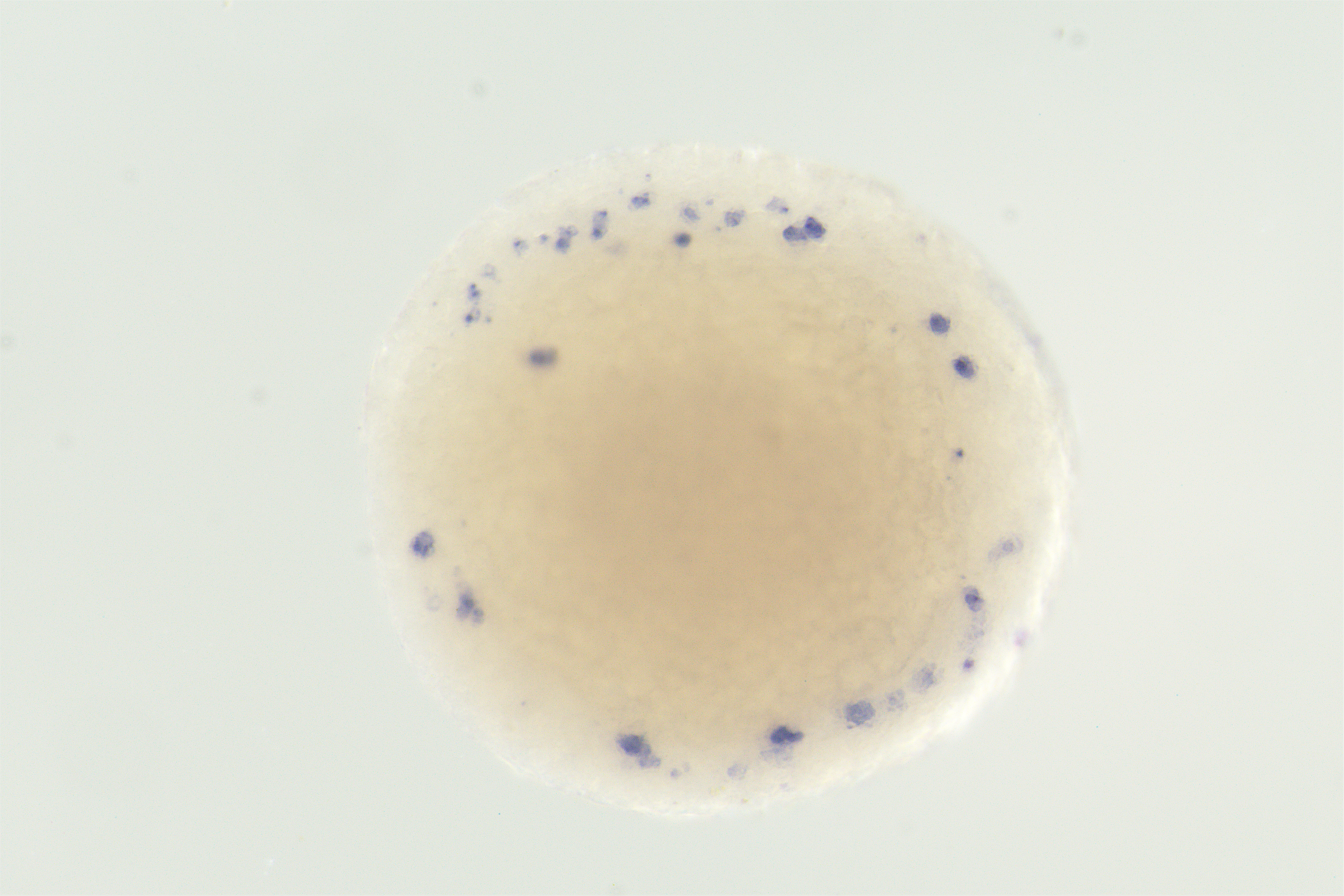

Supplement: Supplementary file 22 — Figure EV5 Source Data [file 44318_2025_442_MOESM22_ESM.zip › Figure_EV5/Figure EV5h/wt inj rbm24a mRNA .tif]

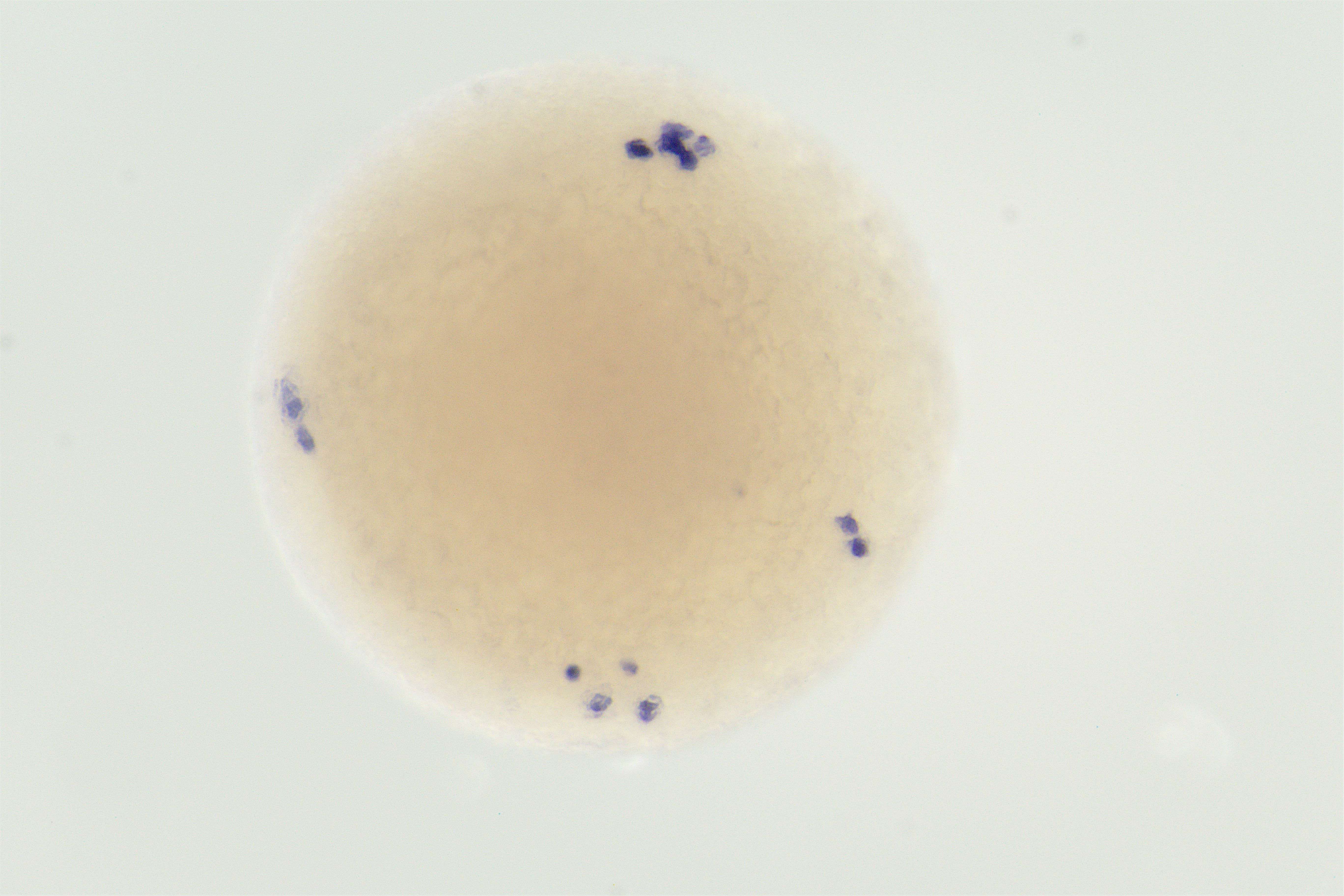

Supplement: Supplementary file 22 — Figure EV5 Source Data [file 44318_2025_442_MOESM22_ESM.zip › Figure_EV5/Figure EV5h/wt uninj .tif]
