# Supplementary material for: Rbm24a dictates mRNA recruitment for germ granule assembly in zebrafish
Source: EMBO J. 2025 Apr 25;44(11):3121–49. doi: 10.1038/s44318-025-00442-z (PMC12130248; doi:10.1038/s44318-025-00442-z)
Supplement: Supplementary file 23 — Expanded View Figures [file 44318_2025_442_MOESM23_ESM.pdf]

Expanded View Figures

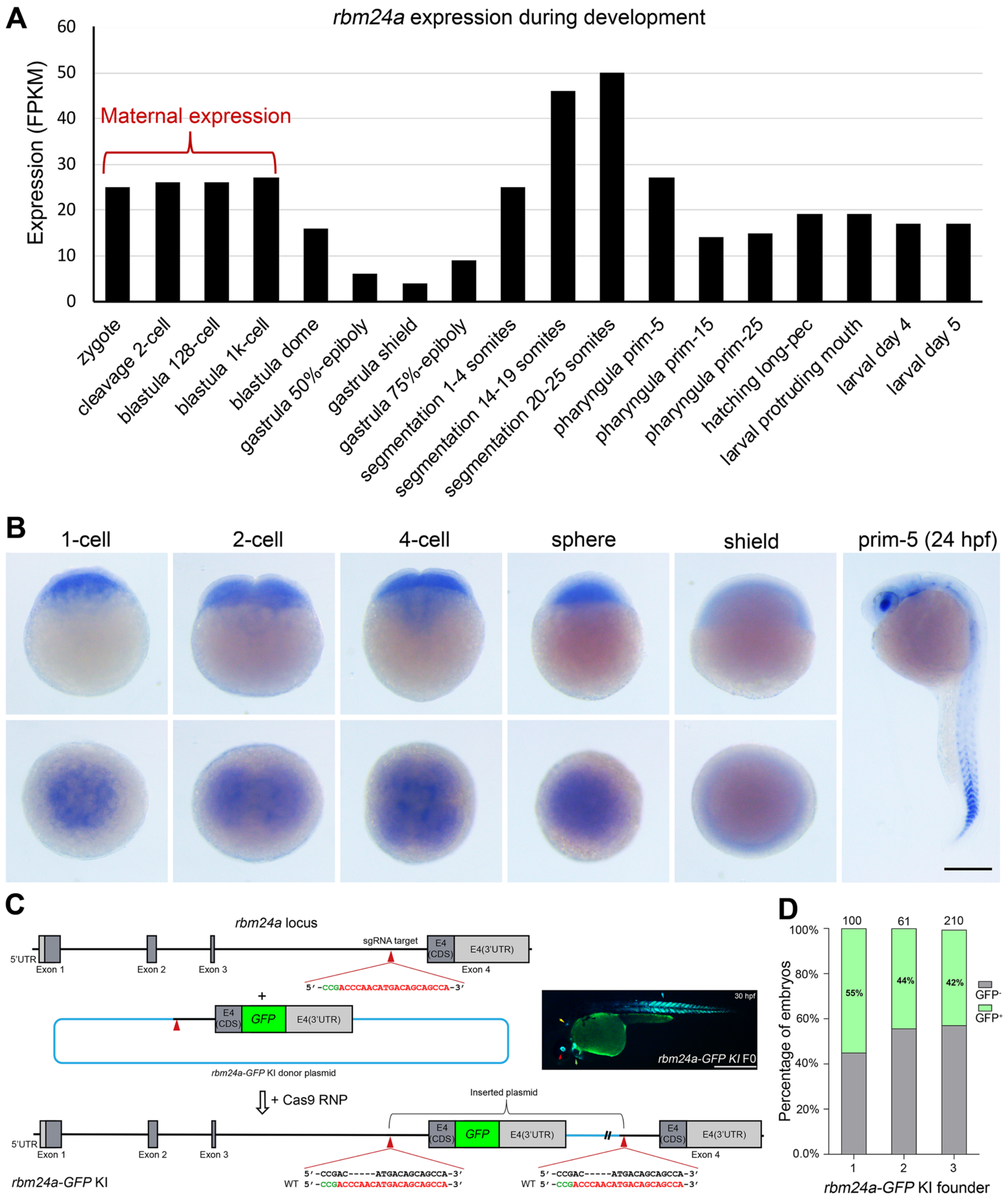

Figure EV1. Expression of zebrafish *rbm24a* transcripts and construction of *rbm24a-GFP* KI line.

(**A**) Expression levels of *rbm24a* transcripts at different developmental stages, replotted from a published resource (White et al, 2017). (**B**) Spatial expression of *rbm24a* transcripts. Scale bar, 250 μm. (**C**) Diagram illustrating the knock-in strategy to insert a GFP tag at the C-terminus of endogenous Rbm24a. The inset on the right shows an F0 embryo with early integration of the knock-in plasmid. Scale bar, 500 μm. (**D**) Germline transmission of three founders with early integration event. Numbers on top of each column represent embryos analyzed from each founder. Source data are available online for this figure.

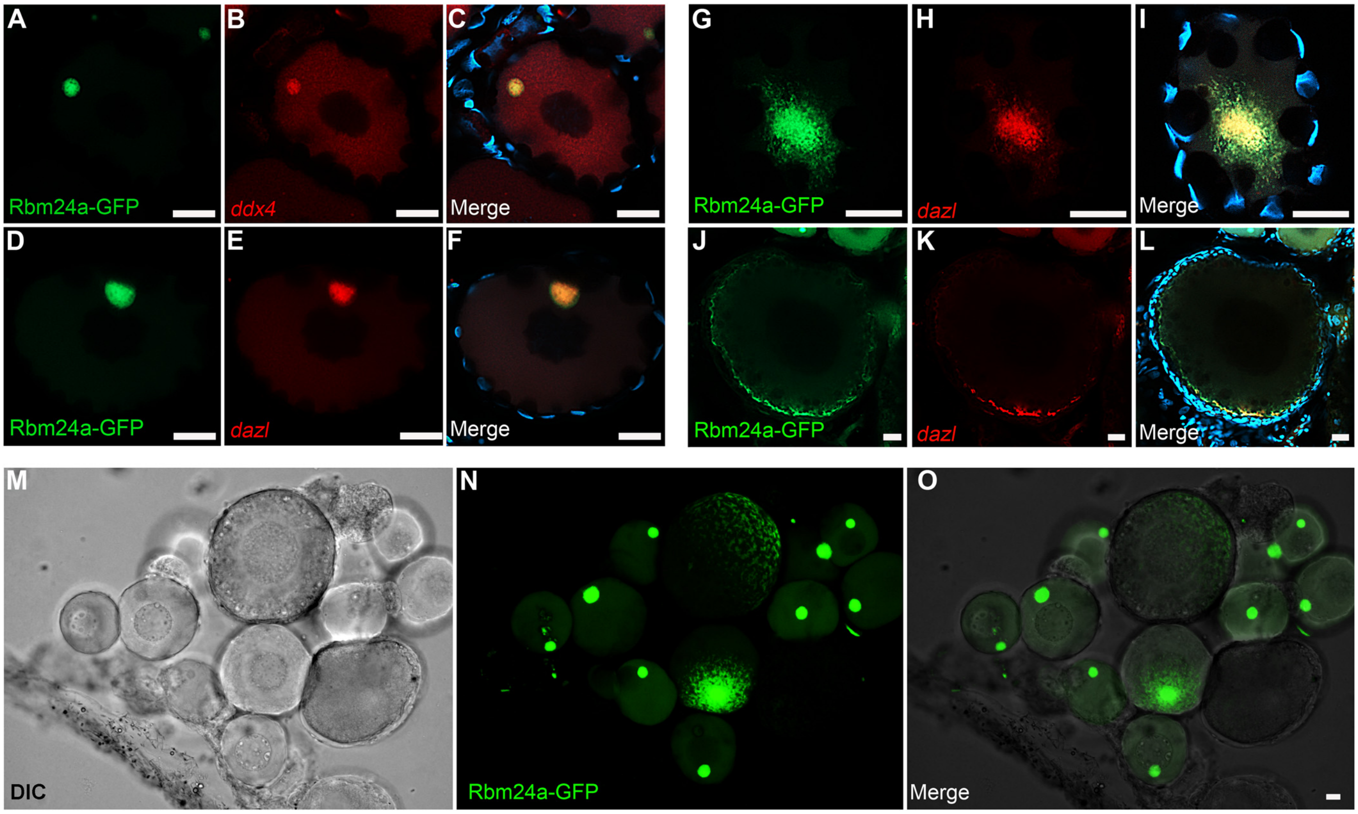

Figure EV2. Rbm24a localization during oogenesis.

(**A–F**) Colocalization of Rbm24a-GFP with *ddx4* and *dazl* in the BB of stage Ia oocyte. Scale bars, 20 μm. (**G–L**) Rbm24a is released following BB breakdown in the late stage Ib and stage II oocytes. Scale bars, 20 μm. (**M–O**) Live *rbm24a-GFP* KI oocytes at different stages. Note the BB localization of Rbm24a in stage I oocytes and a spread of Rbm24a as patched forms in larger oocytes. Scale bar, 20 μm. Source data are available online for this figure.

 © The Author(s)

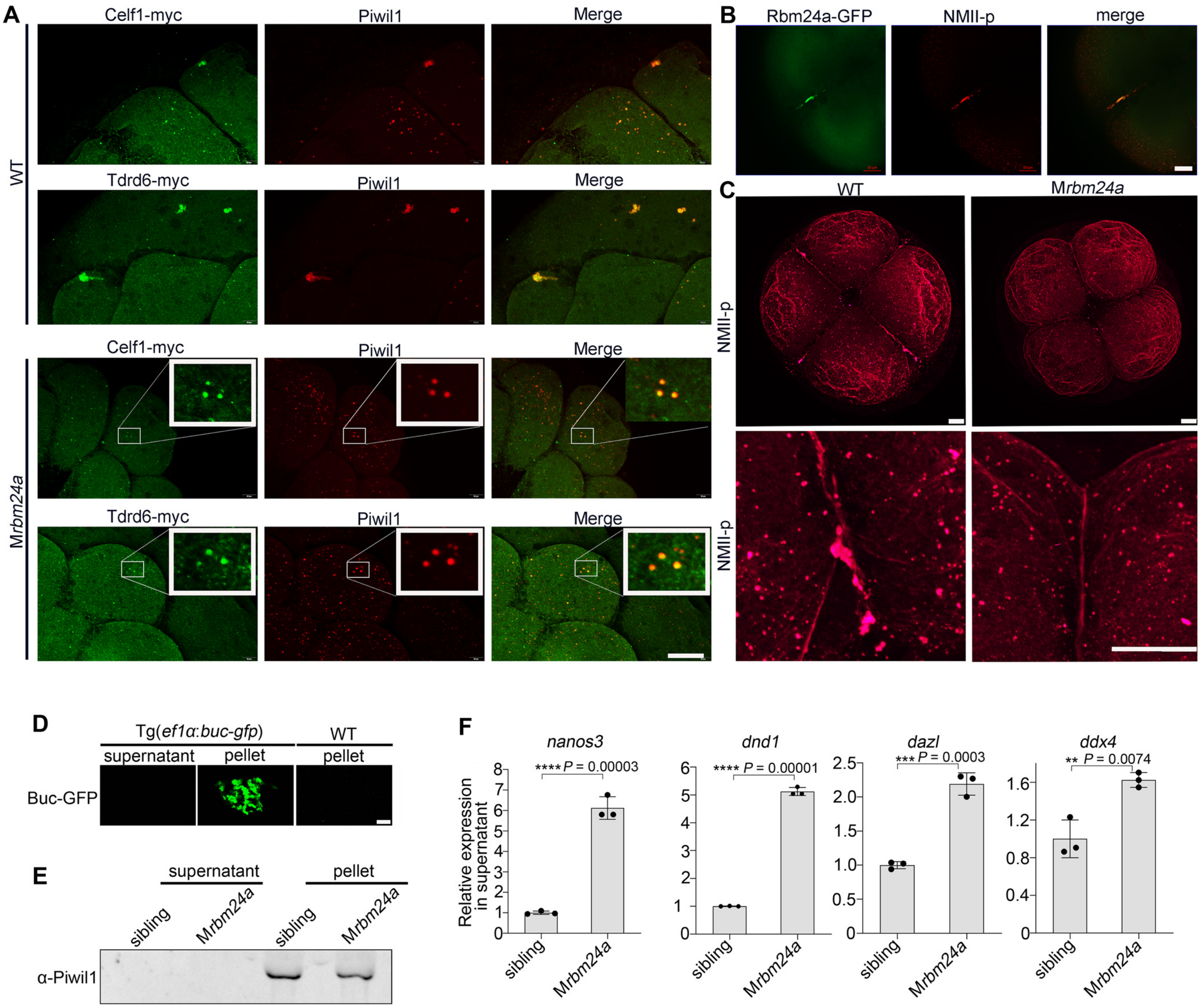

Figure EV3. Rbm24a is required for the recruitment of germ plasm mRNAs but not proteins into germ granules.

(**A**) Recruitment of Tdrd6-myc and Celf1-myc into germ plasm condensates in the presence or absence of maternal Rbm24a. Scale bar, 50 μm. (**B**) Colocalization of NMII-p with Rbm24a-GFP in the cleavage furrow. Scale bar, 50 μm. (**C**) NMII-p localizes in small germ granules in the absence of maternal Rbm24a. Scale bars, 50 μm. (**D**) Isolated germ plasm condensates examined by confocal microscopy. Scale bar, 10 μm. (**E**) Western blot analysis of Piwil1 in the supernatant and the pellet. (**F**) Expression levels of germ plasm mRNAs in the supernatants of siblings and *Mrbm24a* embryos ($n = 3$ independent biological samples). Data are presented as mean ± SD. $^{**}P < 0.01$, $^{***}P < 0.001$ and $^{****}P < 0.0001$, unpaired Student's *t* test. Source data are available online for this figure.

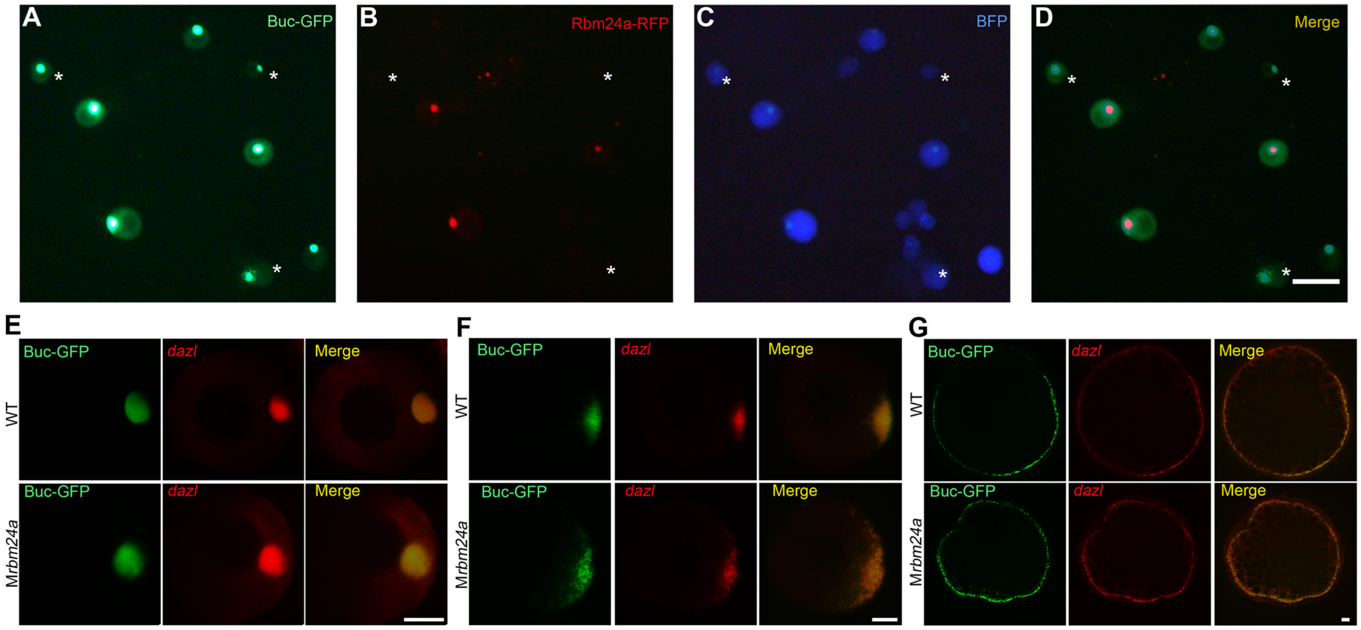

Figure EV4. Loss of Rbm24a function does not affect the formation or dissociation of BB.

(**A–D**) Identification of *rbm24a* mutant oocytes in a homozygous *rbm24a-RFP* KI$^{zpc:cas9}$;Tg(*U6:4xsgRNA*^{rbm24a});Tg(*ef1a:buc-GFP*) background. Notably, Rbm24a-RFP signal is absent in mutant oocytes (asterisks). Scale bar, 500 μm. (**E**) BB formation is normal after the loss of *Mrbm24a*, as revealed by the distribution of Buc-GFP and *dazl* mRNA in stage Ia oocytes. Scale bar, 20 μm. (**F**, **G**) BB dissociation is not affected by the absence of maternal Rbm24a. Oocytes in (**F**) are at late stage Ib, while oocytes in (**G**) are at stage III. Scale bars, 20 μm. Source data are available online for this figure.

 © The Author(s)

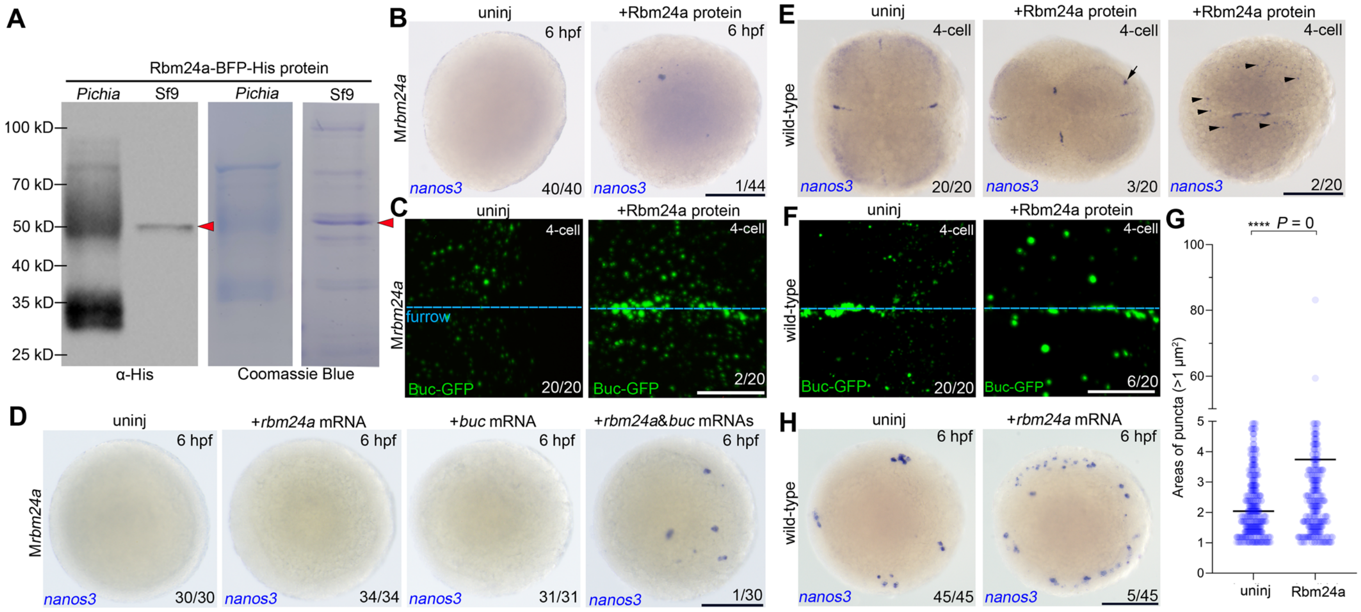

Figure EV5. Overexpression of Rbm24a induces germ granule aggregation and PGC formation with low efficiency.

(**A**) Purified recombinant Rbm24a-BFP-His protein from Pichia yeast and Sf9 cell lines. (**B**) Injection of recombinant Rbm24a protein rescues PGC formation in M*rbm24a* with low efficiency. Scale bar, 200 μm. (**C**) Supplementation of recombinant Rbm24a protein induces increased germ granule aggregation along the cleavage furrow. Scale bar, 50 μm. (**D**) Co-injection of *rbm24a* and *buc* mRNAs into M*rbm24a* embryos rescues PGC formation defect with low efficiency, while separate injection has no effect. Scale bar, 200 μm. (**E**) Injection of recombinant Rbm24a protein into wild-type embryos results in ectopic and large germ plasm aggregates. Scale bar, 200 μm. (**F**) Rbm24a promotes large germ granule formation outside the cleavage furrow, as monitored by transgenic Buc-GFP marker. Scale bar, 50 μm. (**G**) Scatter plot shows the size of germ granules outside the cleavage furrow (data are from three independent embryos for each group). Only granules with size >1 μm^2 were counted. The arithmetic mean was denoted by black horizontal lines. For uninjected group $n = 498$, for Rbm24a protein-injected group $n = 640$. ****$P < 0.0001$, unpaired Student's t test. (**H**) Increased PGCs in embryos injected with *rbm24a* mRNA at 6 hpf. Scale bar, 200 μm. Source data are available online for this figure.
